# Supplementary material for: Diversity and evolution of the emerging Pandoraviridae family
Source: Nat Commun. 2018 Jun 11;9:2285. doi: 10.1038/s41467-018-04698-4 (PMC5995976; doi:10.1038/s41467-018-04698-4)
Supplement: Supplementary file 1 — Supplementary Information [file 41467_2018_4698_MOESM1_ESM.pdf]

Title: Diversity and evolution of the emerging *Pandoraviridae* family

Authors: Legendre et al.

SI Materials and Methods

Virus amplification

For each virus the production was performed using cells cultured at 32°C in microplates with 1 mL of proteose peptone–yeast extract–glucose (PPYG) medium (2% proteose peptone, 0.1% yeast extract, 2.5 mM KH_2PO_4, 2.5 mM Na_2HPO_4, 0.4 mM $CaCl_2$, 4 mM $MgSO_4(H_2O)_7$, 50 µM $Fe(NH_4)_2(SO_4)_2$, 100 mM glucose pH 6.5) supplemented with antibiotics [ampicillin, 100 µg/mL, and penicillin–streptomycin, 100 µg/mL (Gibco); Fungizone, 2.5 µg/mL (Life Technologies)].

For P. macleodensis, cell lysis was observed after several passaging of the culture medium on fresh cells. The culture medium was thus recovered and centrifuged 5 min at 500 x g to remove debris. The supernatant was centrifuged 5 min at 16,000 x g and the pellet resuspended in 100 µl PBS supplemented with antibiotics and used to infect fresh Acanthamoeba cells.

For P. quescus, both the supernatant and the resuspended pellet produced cell lysis. In both cases, the culture medium was recovered and centrifuged 5 min at 500 g. The supernatant was recovered and centrifuged 30 min at 14,000g and the pellet was resuspended in 100 µl PBS supplemented with antibiotics which were then used to infected fresh Acanthamoeba cells. As for P. neocaledonia, and P. macleodensis, visible particles resembling pandoraviruses were visible in the culture media after cell lysis.

Virus cloning and purification

For each newly isolated pandoravirus, we cloned it[1] and amplified the clone prior purification, DNA extraction, proteome analysis, and cell cycle characterization by transcriptomic and electron microscopy.

Pandoraviruses were purified after cell lysis by centrifuging the culture media for 5 min at 500 × g to remove the cellular debris and the virus was pelleted by centrifugation at 6,800 × g for 45 min. The viral pellet was then resuspended, washed twice in PBS buffer, layered on a discontinuous sucrose gradient [50%/60%/70%/80% (wt/vol)], and centrifuged at 5,000 × g for 45 min. The viral layer was recovered, washed twice in PBS buffer, and stored at 4 °C.

Synchronous infections for TEM observations

For each time point, $3.5x10^7$ A. castellanii -adherent cells in 40 ml culture medium were infected with each pandoravirus with a MOI ranging from 50 to 100 for synchronization. After 1h of infection at 32°C, cells were washed 3 times with 30mL of PPYG to eliminate the excess of viruses. For each infection time (every hour from 1h to 15h pi), 2.5 ml were recovered for inclusions and the rest of the cells were centrifuged for 10min at 5,000 x g. The collected 2.5 ml A. castellanii cells were fixed by adding an equal volume of PBS with 5% glutaraldehyde and incubating 1 h at room temperature. Cells are than centrifuged 10 min at 5,000 x g and the cell pellets were resuspended in 1 ml PBS with 2.5% glutaraldehyde, incubated at least 1 h at 4 °C, and washed twice in PBS buffer prior coating in agarose and embedding in Epon resin. Each pellet was mixed with 2% low melting agarose and

centrifuged to obtain small flanges of approximately 1 mm^3 containing the sample coated with agarose. These samples were then prepared using the OTO (osmium-thiocarbohydrazide-osmium) method: 1h fixation in 2% osmium tetroxide with 1.5% potassium ferrocyanide, 20 minutes in 1% thiocarbohydrazide, 30 minutes in 2% osmium tetroxide, overnight incubation in 1% uranyl acetate, 30 minutes in lead aspartate, dehydration in increasing ethanol concentrations (50%, 70%, 90%, and 100% ethanol), and embedding in Epon-812. Ultrathin sections of 90 nm were observed using a FEI Tecnai G2 operating at 200 kV. The infectious cycle of the different pandoravirus strains were then scrutinized for potential differences.

mRNA preparation

A fraction of the different time points of infected cells used for the TEM study were pooled to retrieve the $poly(A)^+$ fraction of RNA and access the information on pandoravirus genes structure. RNA were extracted from the A. castellanii cells using the RNeasy Midi kit (catalog no. 75144; Qiagen) using the manufacturer's protocol. Briefly, the cell pellets were resuspended in 4 mL of RLT buffer supplemented with 0.1% β-mercaptoethanol and disrupted by subsequent −80 °C freezing and thawing at 37 °C for 10 min. For each pandoravirus infection, the total RNA were eluted with two successive additions of ∼200 µL of RNase-free water and quantified on the nanodrop spectrophotometer (Thermo Scientific). Two successive poly(A) enrichments were performed (Life Technologies, Dynabeads $oligodT_{25}$) in order to completely remove ribosomal RNA.

DNA extraction for sequencing

DNA was extracted from 2 × 109 purified particles. They were first centrifuged 3 min at 16,000 x g and the pellet was resuspended in 300 µl ultra-pure water to which 500 µl of CTAB buffer (20 g/L CTAB, 1,4 M NaCl, 100 mM Tris-HCl, 20 mM Na2EDTA, pH 8,0), 20 mg/mL Proteinase K and 3 µL de DTT 1 M were added. The sample was incubated 1 and half hour at 65°C and an additional 10 min after adding 20 mg/mL RNase A. 500 µL chloroform were then added and the sample was vortexed 30 sec prior centrifugation10 min at 16,000 g. The upper aqueous layer was recovered and centrifuged 5 min at 16,000 x g at 4°C. The supernatant is then mixed with an equal volume of chloroform, vortexed and centrifuged 5 min at 16,000 x g at 4°C. The aqueous layer was again recovered and mixed with 2 volumes of precipitation buffer (5 g/L CTAB, 40 mM NaCl, pH 8.0) and gently mixed. The sample was left at room temperature 1 h prior 5 min centrifugation at 16,000 g. The pellet was gently dissolved in 350 µl 1.2 M NaCl, 350 µl chloroform were added, the solution was vortexed few sec and centrifuged 5 min at 16,000 g at 4°C. The aqueous layer was again recovered and 0.6 volumes isopropanol were added and gently mixed until the DNA precipitated. The sample was centrifuged 10 min at room temperature at 16,000 g and the pellet was gently washed with 500 µl 70% ethanol. After cenfrifugation 10 min at room temperature at 16,000 x g, the pellet was left to dry prior being resuspended in 20 µl ultra-pure water.

Proteomic analysis

For P. salinus, P. dulcis, P. neocaledonia and P. quercus the virions produced from each clone were purified on Cesium chloride gradient. Proteins were extracted using a standard protocol[2] starting from 10^9 virions. Two biological replicates were produced for P. dulcis

starting with two independent productions. For the 2 other pandoraviruses, independant triplicates were produced starting from the same pandoravirus production. Characterization of the different clones and technical replicates were performed for P. dulcis. Peptides and proteins were identified and quantified as previously described[1] using MaxQuant software[3] (version 1.5.5.1) in independent runs for each virus. Spectra were searched against the corresponding pandoravirus protein sequences as well as A. castellanii protein sequences, and the frequently observed contaminants database embedded in MaxQuant. Trypsin was chosen as the enzyme and two missed cleavages were allowed. Peptide modifications selected during the searches were carbamidomethylation (C, fixed), acetyl (protein Nter, variable), and oxidation (M, variable). Minimum peptide length was set to 7 aa. The 'match between run' option was activated. Maximum false discovery rates were set to 0.01 at peptide and protein levels. Intensity-based absolute quantification (iBAQ)[4] values were calculated from MS intensities of 'unique+razor' peptides. For each pandoravirus, iBAQ values were column-wise normalized and the protein ranking in particle was deducted from to the sum of the normalized iBAQ values per protein. For characterization of particle protein contents, only proteins identified by MS/MS in at least 2 replicates and quantified in all three replicates were taken into account for further analyses.

Genome sequencing and assembly

Pandoravirus neocaledonia genome was sequenced using 2 SMRT cells of the Pacbio sequencing technology. Sequencing reads were pre-assembled using the HGAP workflow[5] from the SMRT analysis framework version 2.3.0 with default parameters, resulting in 38,592 corrected reads. The same workflow was then used to perform the final polished assembly resulting in a contig of 2,003,191 nt. Read coverage was uniform except for a region of 10 kb at one extremity of the genome showing a large increase in coverage (60x vs 1950x). This is characteristic of terminal repeats as was showed in previously published pandoraviruses' genomes[2,6].

The same approach was used to sequence the Pandoravirus quercus genome with 1 SMRT cell resulting in 72,468 corrected reads assembled in a single contig of 2,077,288 nt. Again, while read coverage was uniform in most of the genome, a peak was found in a 40 kb region at one extremity (400x vs 650x read coverage).

Pandoravirus macleodensis was sequenced using the Illumina MiSeq technology with large insert (5-8 kb) mate pair sequences. After read filtration for low quality sequenced bases and adapter cleaning, the dataset contained a total of 3,492,652 mate pair reads of 122 nt on average. The SPAdes assembler version 3.8.0[7] was used with the defaults parameters except for the "careful" option and the following set of kmers: 21, 41, 61, 81, 91, 101, 121, 127. A single contig of 1,838,258 nt was obtained. Read coverage was uniform except for a 30kb-long region at the end of the contig showing a higher coverage (220x vs 460x).

Stringent genome sequence annotation

For the 4 viruses for which we had transcriptomic and proteomic data available (*i.e.* P. salinus, P. dulcis, P. quercus and P. neocaledonia; see Table 1) we sought to stringently predict protein-coding genes. As summarized in Supplementary Fig. 2 the pipeline takes into

account RNA-seq data, Mass spectroscopy data of the particles as well as protein conservation data.

We first predicted gene models using 4 gene finders: GenemarkS[8], EMBOSS getorf[9], Braker[10] and GenemarkS-T[11]. The latter two were supported by RNA-seq transcripts (see below).

RNA-seq transcriptomic data from pooled time-course infection data points were exploited as follows. We first mapped the stranded RNA-seq reads to the viral reference genomes using Tophat version 2.0.12[12] with the following parameters: i=20, I=1500, b2-very-sensitive and no-discordant. We then performed the assembly of the RNA-seq reads into transcripts using Trinity[13] with the RNA-seq reads alignments (ie "genome-guided"). The following options were used: jaccard_clip, trimmomatic and genome_guided_max_intron=1500. Finally assembled transcripts were mapped to the reference genome using the PASA pipeline[14].

Peptides from the proteomic data of the virions particles were identified using MaxQuant[3] on a very permissive definition of ORFs. The reference ORF database was made using EMBOSS getorf allowing overlapping ORFs on the six frames. Once identified, significant peptides were mapped to the genomic coordinates using the CDSmapper script[15].

Protein conservation among the pandoraviruses was exploited as follows. We performed a protein clustering of the proteins predicted *ab initio* by GenemarkS[8] in all 6 Pandoravirus genomes using OrthoMCL[16] with default parameters. Excluding singletons we then aligned the resulting protein clusters with mafft-linsi[17] and built an HMM profile for each alignment using Hmmer[18]. Finally HMM profiles were aligned to the previously described permissive ORF database (based on EMBOSS getorf, see previous paragraph) using Hmmer. Matching regions were mapped back to the genomic coordinates using the CDSmapper script[15].

Subsequently, transcriptomic data, proteomic data and protein conservation data were combined in order to choose the best gene models using EvidenceModeler (EVM) [19]. The following weights were used to score the different gene models from the gene finders: Braker prediction=5, GenemarkS prediction=3, GenemarkS-T=5, EMBOSS getorf=1, and the external evidences: virion peptide=25, HMM match=2 and transcript alignment=10. After visual inspection of the gene models picked up by EVM we realized that a handful of probably genuine protein-coding genes were missed. We thus added potential protein-coding genes as long as: i) they showed transcription evidence (aligned transcript) and ii) an ORF >50 amino acids was predicted in the cognate strand. Finally, untranslated regions (UTRs) of predicted protein coding genes were defined using PASA[14] and the transcripts alignment.

For the two genomes for which we had no transcriptomic and proteomic data (*i.e.* P. inopinatum and P. macleodensis; see Table 1), we first trained an Augustus[20] model for gene prediction based on the stringently predicted genes in the other pandoraviruses (see above). We then predicted genes using Augustus[20], GenemarkS[8] and EMBOSS getorf[9]. EVM[19] was then used to choose the best gene models with the following weights: Augustus

prediction=5, GenemarkS prediction=3, EMBOSS getorf=1, and the external evidence of protein conservation HMM match=2.

Functional annotation of protein-coding genes was performed using a combination of sequence similarity searches (Blastp[21] evalue < 1e-5) and protein motifs detections (Interproscan[22]).

Non-coding RNA (ncRNA) genes were predicted for the viruses for which we had transcriptomic data (see Table 1). We defined ncRNAs as genomic region that exhibit transcription (ie transcript alignment) and no predicted ORF in the cognate strand. In order to separate potential transcriptional noise from genuine ncRNA transcripts, we defined a transcript as ncRNA only if the RNA-seq signal was of high enough. The threshold for RNA-seq signal was defined as follows: we collected the RNA-seq signal corresponding to the genomic regions of the peptides identified from the proteomic data of the virions. We then used a Gaussian mixture model to separate this RNA-seq signal from the one corresponding to the opposite strand (considered as potential transcriptional noise). Candidate ncRNA transcripts were finally classified based on this model.

tRNAs were predicted using tRNAscan-SE[23] with default parameters.

Miscellaneous bioinformatics analyses

Protein clustering

Clustering of pandoravirus protein-coding genes was performed using BlastP[21] with default parameters and Evalue < 1e-5, Bit-score>50 and filtering=F, followed by an MCL clustering[24] with an inflation parameter=1.5. A similar clustering was also applied on individual genomes of several giant viruses.

Genome rearrangements

Genomic rearrangements were estimated using Mauve[25] with default parameters in combination with the R package GenoPlotR[26].

Syntenic regions between pandoraviruses were also identified using the MScanX toolkit[27].

Codon Adaptation Index

Codon adaptation index was computed using the “cai” tool from the EMBOSS package[9]. Reference codon usage was calculated on the most expressed protein coding genes in A. castellanii (see Supplementary dataset S3).

Phylogenetic analyses

Phylogenetic trees were computed for each protein-coding gene cluster (aligned with Mcoffee[28]) using the protocol described in[29]. Orthologous gene pairs were defined using the Species Overlap algorithm from the ETE toolkit[30].

Estimation of synonymous (dS), nonsynonymous (dN) substitution rates and dN/dS ratios were calculated using the YN00 method from the PAML package[31]. In order to avoid saturation and outliers we only considered dN/dS if the number of substitutions per synonymous site < 2, the number of nonsynonymous substitutions > 1 and the number of substitutions > 1.

The phylogenetic tree of the pandoraviruses was computed from the codon super alignment of the 1:1 orthologous genes present in all viruses using PhyML[32] with the JC69 substitution model. Estimation of the dN/dS ratios of each branch was computed using the CodeML method from the PAML package[31] through the ETE toolkit[30]. The dN/dS ratio from the two branches (see Fig. 3) were found to be significantly different (pvalue< 1e-5 between models M0 and b_free).

The cladistic tree was computed using the Neighbour-Joining method from the presence/absence matrix of 5375 clusters derived from an OrthoMCL[16] clustering. The distance was the one used in[33]. Support values were estimated using bootstrap resampling (n = 10000). Besides the pandoravirus genomes, the following genomic sequences were included in the analysis:

NC_000852.5, NC_001659.2, NC_001824.1, NC_002188.1, NC_002520.1, NC_002687.1, NC_003038.1, NC_003389.1, NC_003391.1, NC_003494.1, NC_003663.2, NC_004002.1, NC_004003.1, NC_005309.1, NC_005336.1, NC_005337.1, NC_005832.1, NC_005946.1, NC_006549.1, NC_006966.1, NC_006998.1, NC_007346.1, NC_008361.1, NC_008518.1, NC_008724.1, NC_009233.1, NC_011183.1, NC_011335.1, NC_013288.1, NC_013756.1, NC_014637.1, NC_014649.1, NC_014789.1, NC_015326.1, NC_015780.1, NC_016072.1, NC_016924.1, NC_020104.1, NC_020864.1, NC_021312.1, NC_021901.1, NC_023423.1, NC_023848.1, NC_024697.1, NC_025412.1, NC_027867.1, NC_028094.1, NC_029692.1, NC_030230.1, NC_030842.1, NC_031465.1, NC_032108.1, NC_032111.1, NC_033775.1, NC_034249.1, NC_034383.1, NC_017940.1, AY318871.1, AF482758.2.

A maximum likelihood phylogenetic tree based on the DNA polymerase B sequence contained in each of these genomes is shown in Supplementary Fig. 14. A protein sequence multiple alignment was performed with MAFFT using default parameters[17]. We then used Prottest to estimate the best substitution model which was defined as the LG+I+G model. Finally PhyML[32] was used with the following parameters to compute the final phylogenetic tree: -c 4 -m LG -v e -a e -o lr -f d -d aa.

The analysis of potential horizontal gene transfer was performed on P. salinus genes that exhibit a significant BlastP match (Evalue<1e-5) against the NR database. Significant matches of each query were aligned using Mcoffee[28], trees were computed using PhyML[32] and analysed manually to identify HGT direction when possible.

Supplementary References

1. Legendre, M. *et al.* In-depth study of Mollivirus sibericum, a new 30,000-y-old giant virus infecting Acanthamoeba. *Proc. Natl. Acad. Sci. U. S. A.* **112,** E5327–5335 (2015).

2. Philippe, N. *et al.* Pandoraviruses: amoeba viruses with genomes up to 2.5 Mb reaching that of parasitic eukaryotes. *Science* **341,** 281–286 (2013).

3. Cox, J. & Mann, M. MaxQuant enables high peptide identification rates, individualized p.p.b.-range mass accuracies and proteome-wide protein quantification. *Nat. Biotechnol.* **26,** 1367–1372 (2008).

4. Schwanhäusser, B. *et al.* Global quantification of mammalian gene expression control. *Nature* **473,** 337–342 (2011).

5. Chin, C.-S. *et al.* Nonhybrid, finished microbial genome assemblies from long-read SMRT sequencing data. *Nat. Methods* **10,** 563–569 (2013).

6. Antwerpen, M. H. *et al.* Whole-genome sequencing of a pandoravirus isolated from keratitis-inducing acanthamoeba. *Genome Announc.* **3,** (2015).

7. Nurk, S. *et al.* Assembling Genomes and Mini-metagenomes from Highly Chimeric Reads. in *Research in Computational Molecular Biology* 158–170 (Springer, Berlin, Heidelberg, 2013). doi:10.1007/978-3-642-37195-0_13

8. Besemer, J., Lomsadze, A. & Borodovsky, M. GeneMarkS: a self-training method for prediction of gene starts in microbial genomes. Implications for finding sequence motifs in regulatory regions. *Nucleic Acids Res.* **29,** 2607–2618 (2001).

9. Rice, P., Longden, I. & Bleasby, A. EMBOSS: the European Molecular Biology Open Software Suite. *Trends Genet. TIG* **16,** 276–277 (2000).

10. Hoff, K. J., Lange, S., Lomsadze, A., Borodovsky, M. & Stanke, M. BRAKER1: Unsupervised RNA-Seq-Based Genome Annotation with GeneMark-ET and AUGUSTUS. *Bioinforma. Oxf. Engl.* **32,** 767–769 (2016).

11. Tang, S., Lomsadze, A. & Borodovsky, M. Identification of protein coding regions in RNA transcripts. *Nucleic Acids Res.* **43,** e78 (2015).

12. Kim, D. *et al.* TopHat2: accurate alignment of transcriptomes in the presence of insertions, deletions and gene fusions. *Genome Biol.* **14,** R36 (2013).

13. Grabherr, M. G. *et al.* Full-length transcriptome assembly from RNA-Seq data without a reference genome. *Nat. Biotechnol.* **29,** 644–652 (2011).

14. Haas, B. J. *et al.* Improving the Arabidopsis genome annotation using maximal transcript alignment assemblies. *Nucleic Acids Res.* **31,** 5654–5666 (2003).

15. Bringans, S. *et al.* Deep proteogenomics; high throughput gene validation by multidimensional liquid chromatography and mass spectrometry of proteins from the fungal wheat pathogen Stagonospora nodorum. *BMC Bioinformatics* **10,** 301 (2009).

16. Li, L., Stoeckert, C. J. & Roos, D. S. OrthoMCL: Identification of Ortholog Groups for Eukaryotic Genomes. *Genome Res.* **13,** 2178–2189 (2003).

17. Katoh, K. & Standley, D. M. MAFFT multiple sequence alignment software version 7: improvements in performance and usability. *Mol. Biol. Evol.* **30,** 772–780 (2013).

18. Eddy, S. R. A new generation of homology search tools based on probabilistic inference. *Genome Inform. Int. Conf. Genome Inform.* **23,** 205–211 (2009).

19. Haas, B. J. *et al.* Automated eukaryotic gene structure annotation using EVidenceModeler and the Program to Assemble Spliced Alignments. *Genome Biol.* **9,** R7 (2008).

20. Stanke, M. & Waack, S. Gene prediction with a hidden Markov model and a new intron submodel. *Bioinforma. Oxf. Engl.* **19 Suppl 2,** ii215–225 (2003).

21. Altschul, S. F., Gish, W., Miller, W., Myers, E. W. & Lipman, D. J. Basic local alignment search tool. *J. Mol. Biol.* **215,** 403–410 (1990).

22. Jones, P. *et al.* InterProScan 5: genome-scale protein function classification. *Bioinformatics* **30,** 1236–1240 (2014).

23. Lowe, T. M. & Eddy, S. R. tRNAscan-SE: a program for improved detection of transfer RNA genes in genomic sequence. *Nucleic Acids Res.* **25,** 955–964 (1997).

24. Enright, A. J., Van Dongen, S. & Ouzounis, C. A. An efficient algorithm for large-scale detection of protein families. *Nucleic Acids Res.* **30,** 1575–1584 (2002).

25. Darling, A. E., Mau, B. & Perna, N. T. progressiveMauve: multiple genome alignment with gene gain, loss and rearrangement. *PloS One* **5,** e11147 (2010).

26. Guy, L., Roat Kultima, J. & Andersson, S. G. E. genoPlotR: comparative gene and genome visualization in R. *Bioinformatics* **26,** 2334–2335 (2010).

27. Wang, Y. *et al.* MCScanX: a toolkit for detection and evolutionary analysis of gene synteny and collinearity. *Nucleic Acids Res.* **40,** e49 (2012).

28. Notredame, C., Higgins, D. G. & Heringa, J. T-Coffee: A novel method for fast and accurate multiple sequence alignment. *J. Mol. Biol.* **302,** 205–217 (2000).

29. Vilella, A. J. *et al.* EnsemblCompara GeneTrees: Complete, duplication-aware phylogenetic trees in vertebrates. *Genome Res.* **19,** 327–335 (2009).

30. Huerta-Cepas, J., Serra, F. & Bork, P. ETE 3: Reconstruction, Analysis, and Visualization of Phylogenomic Data. *Mol. Biol. Evol.* **33,** 1635–1638 (2016).

31. Yang, Z. PAML 4: phylogenetic analysis by maximum likelihood. *Mol. Biol. Evol.* **24,** 1586–1591 (2007).

32. Guindon, S. *et al.* New algorithms and methods to estimate maximum-likelihood phylogenies: assessing the performance of PhyML 3.0. *Syst. Biol.* **59,** 307–321 (2010).

33. Snel, B., Bork, P. & Huynen, M. A. Genome phylogeny based on gene content. *Nat. Genet.* **21,** 108–110 (1999).

Supplementary figures

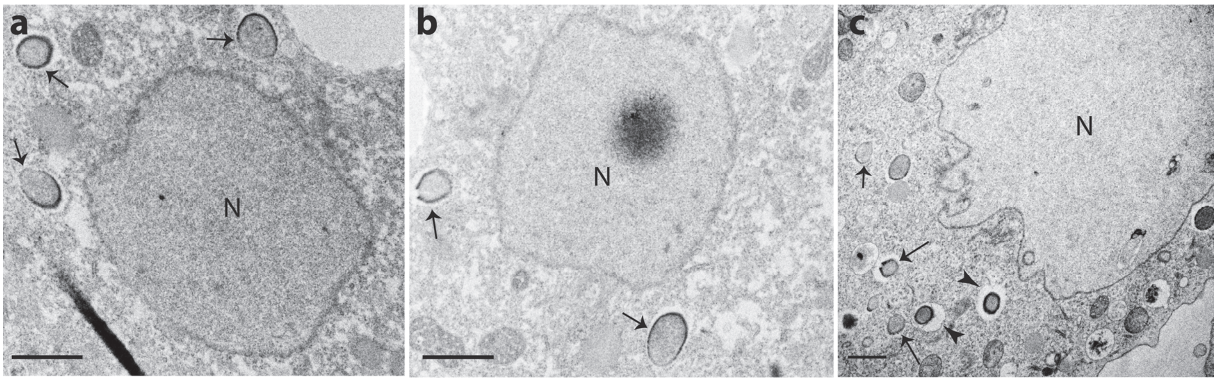

Supplementary Fig. 1. Nuclei in infected A. castellanii cells. Late stage of P. neocaledonia (**a-b**) (scale bar is 1 µm) or P. salinus (**c**) infectious cycle (scale bar is 2 µm). Maturing particles (arrows) co-exist with a nucleus-like compartment limited by a membrane. Mature particles in vacuoles are also visible (arrow-heads).

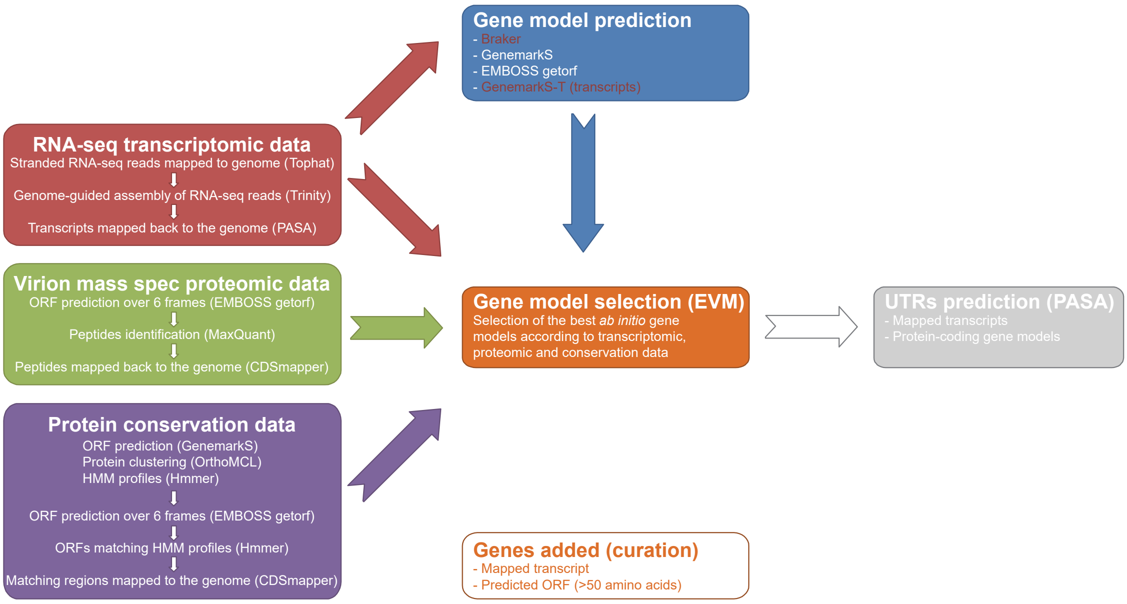

Supplementary Fig. 2. Diagram of the stringent gene annotation pipeline. *Ab initio* gene prediction (blue), transcriptomic data (red), proteomic data (green) and protein conservation data (purple) are combined to accurately predict protein-coding genes.

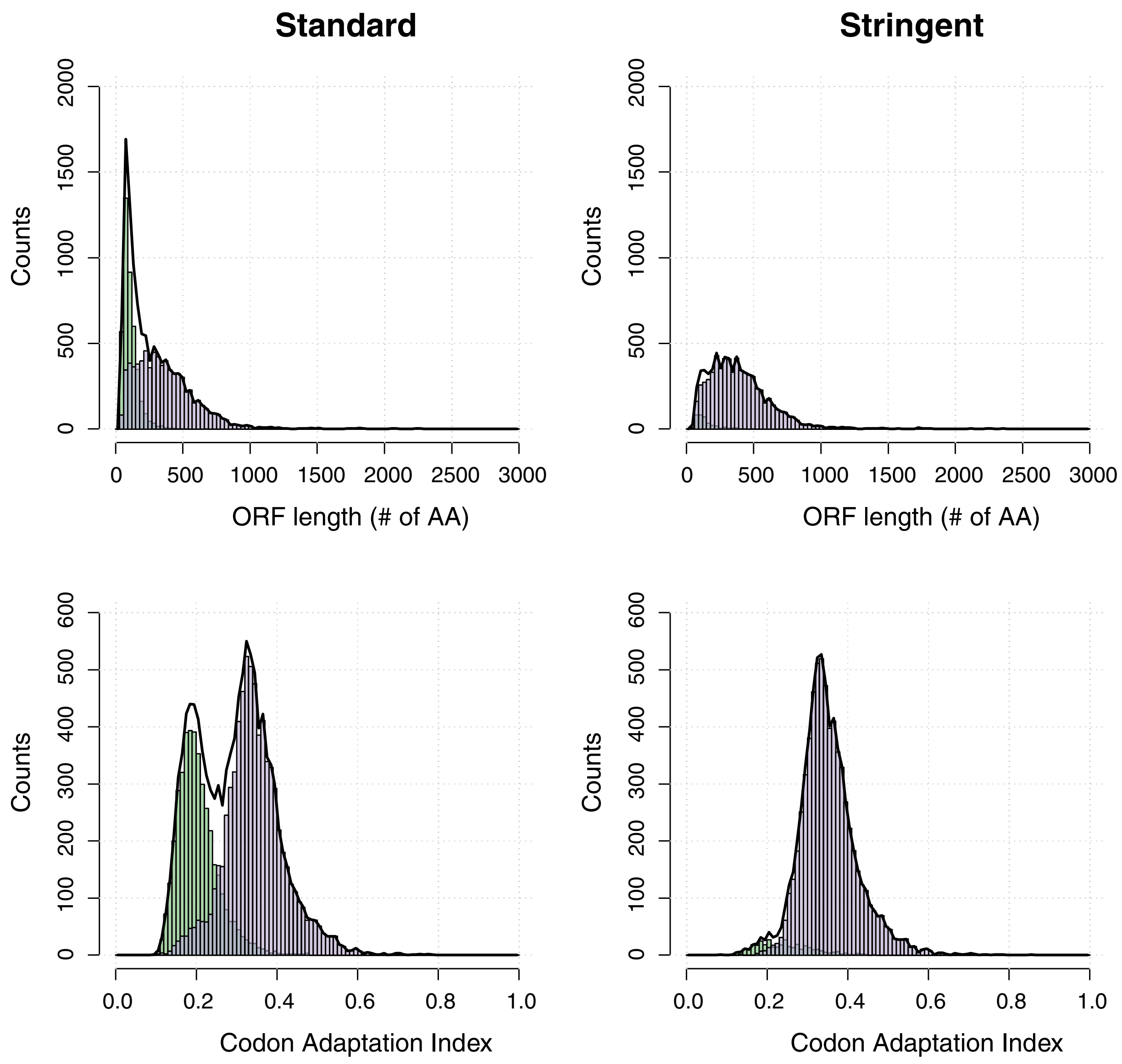

Supplementary Fig. 3. ORF length and codon usage bias distributions. Distributions are displayed for standard (left) and stringent (right) gene annotations. Genes matching other pandoraviruses (BlastP, Evalue < 1e-5) are shown in purple while genes without significant match are shown in green.

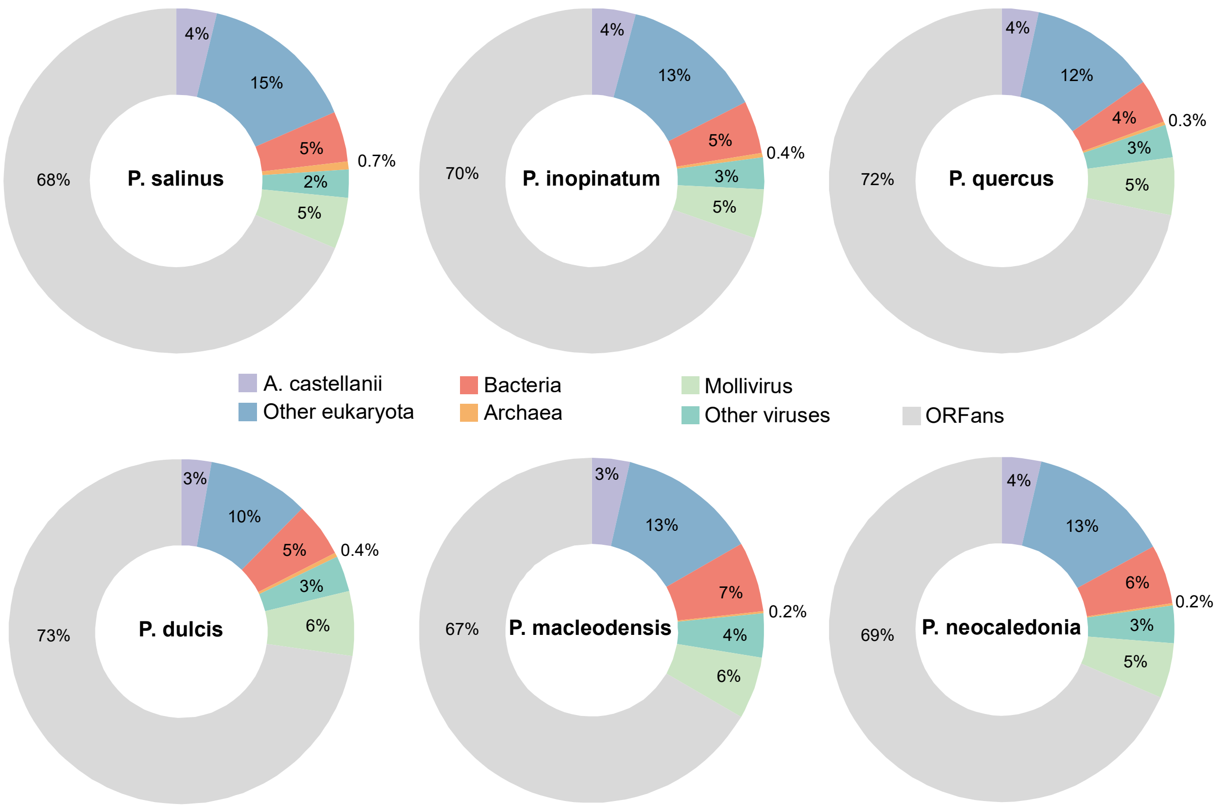

Supplementary Fig. 4. Taxonomic distribution of protein-coding genes best matches for the various pandoravirus strains. Searches were done using BlastP (Evalue < 1e-5) against the NR database excluding hits corresponding to the pandoraviruses.

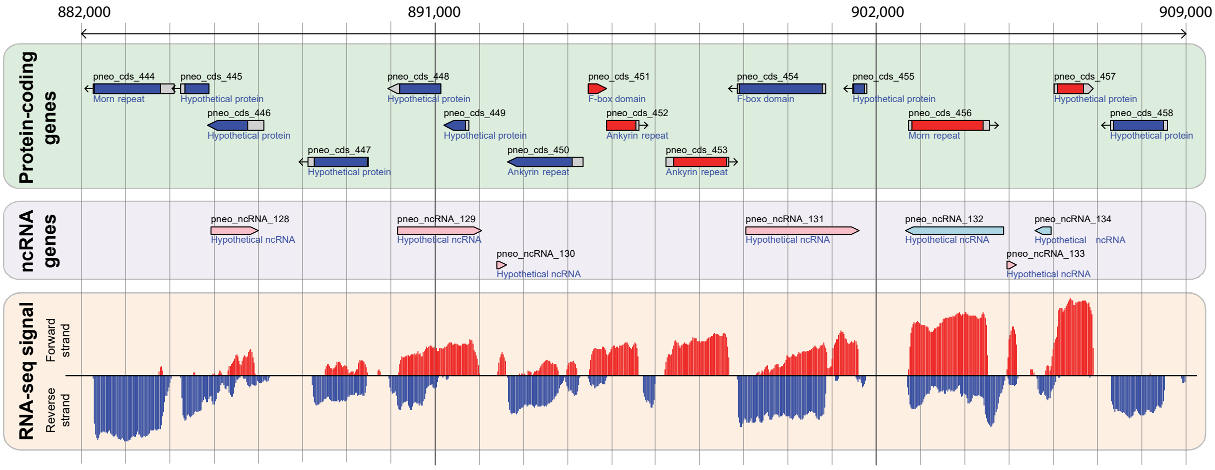

Supplementary Fig. 5. Protein-coding and lncRNA gene mapping using RNA-seq data. The genomic region of Pandoravirus neocaledonia between 882,000 and 909,000 is displayed. Protein-coding genes are shown in the green panel. The coding region is colored in red for genes encoded in the forward strand and in blue for genes encoded in the reverse strand. UTRs are shown in gray. Non-coding RNAs from the forward strand (in pink) and reverse strand (in light blue) are shown in the purple panel. Strand-specific RNA-seq read density from the forward (red) and reverse strand (blue) is shown in the orange panel.

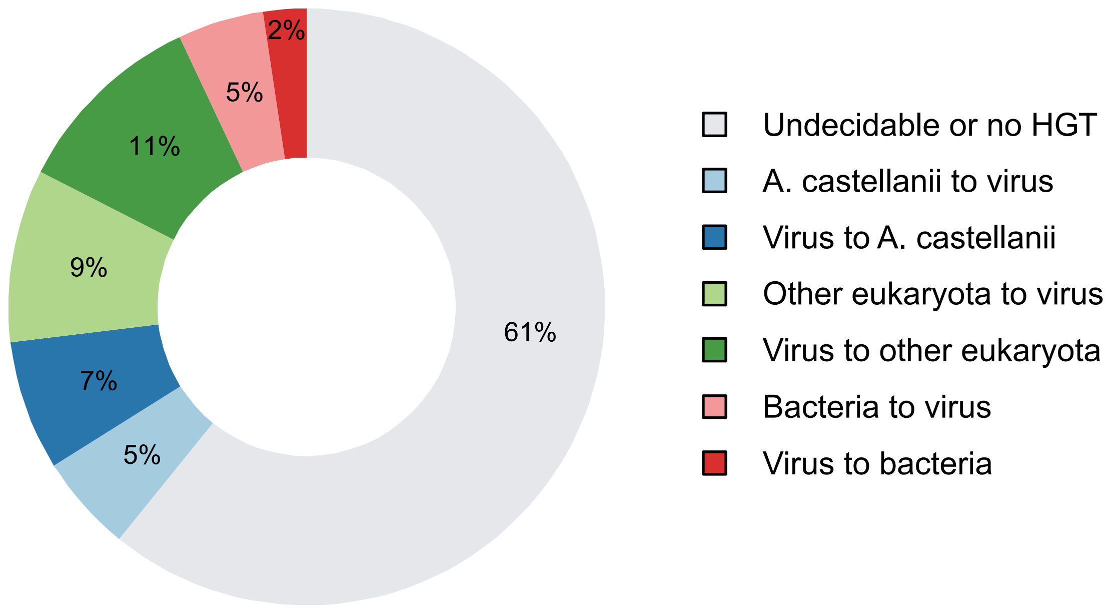

Supplementary Fig. 6. Likely direction of candidate horizontal gene transfers for genes with detectable homologues outside the proposed *Pandoraviridae* family.

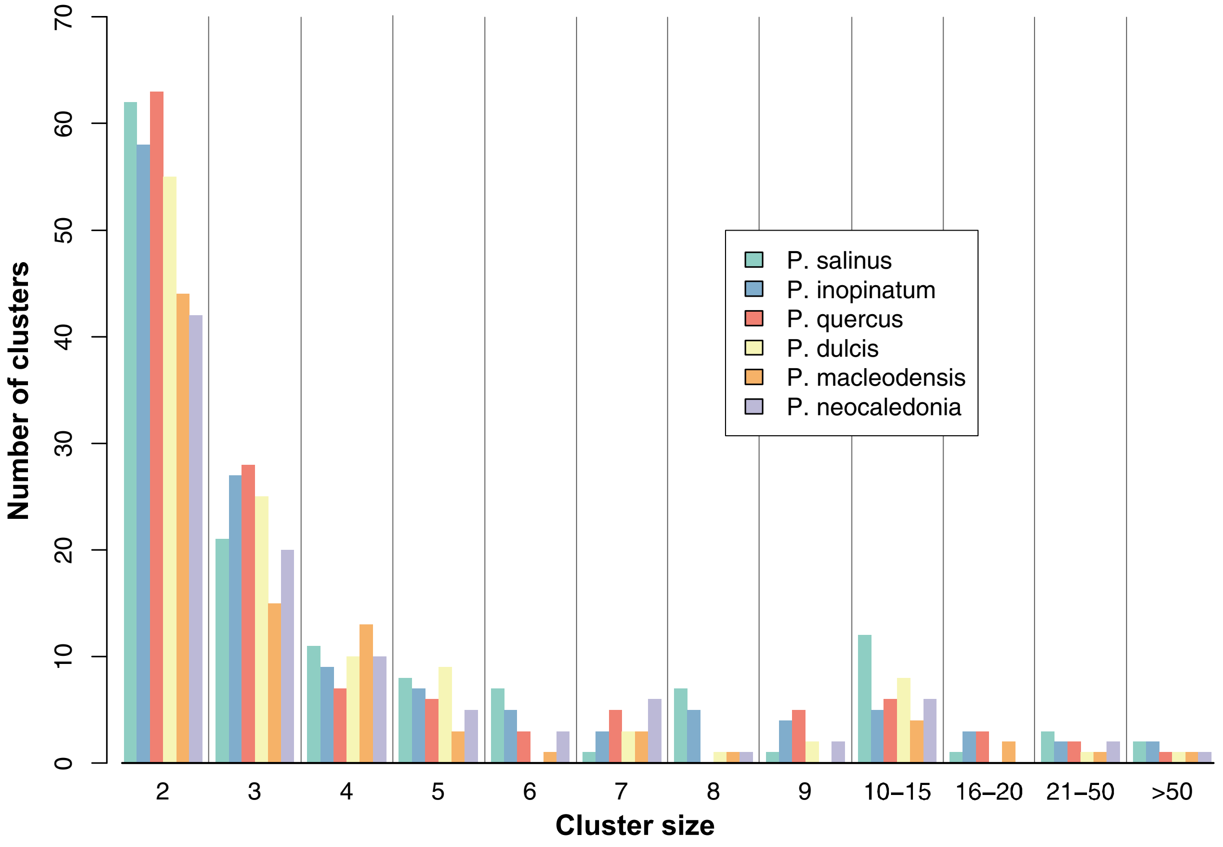

Supplementary Fig. 7. Number of gene cluster of a given size (>2) for the various pandoravirus strains.

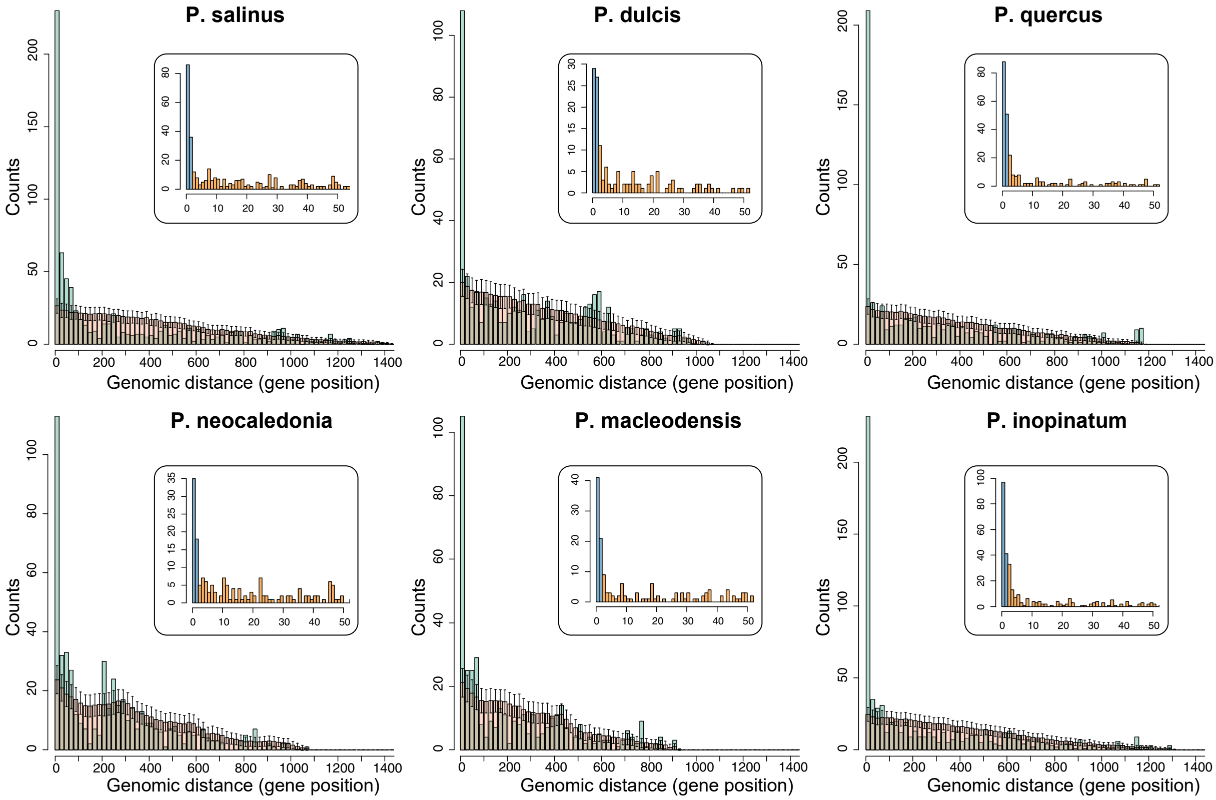

Supplementary Fig. 8. Distributions of genomic distances between the closest paralogs. For each pandoravirus strain, the genomic distance between pairs of within-strain BlastP best matches is shown in green. Genomic distances are computed from the gene order along the genome. A control randomization (n=1000) was performed (in red). The insets show close-ups of the distributions within the 1-50 range. Distances of 1 and 2 are colored in blue and other distances are shown in orange.

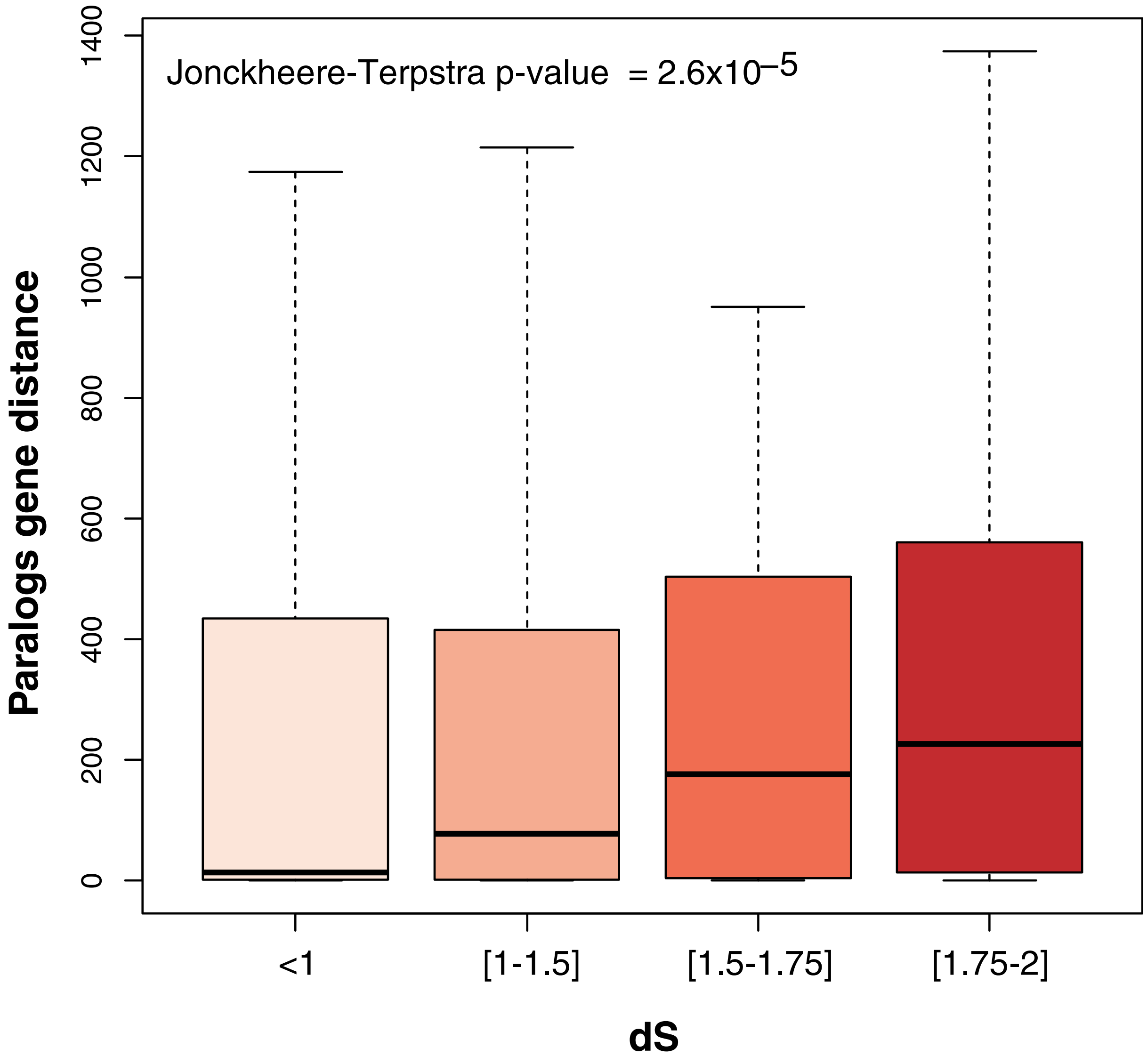

Supplementary Fig. 9. The divergence between closest paralogs increases with their genomic distance. The genomic distance is computed from the gene order along the genome. The sequence divergence is defined as the number of synonymous substitutions per sites (dS). Box plots show the median, the 25th and 75th percentiles. The whiskers correspond to the extreme data points.

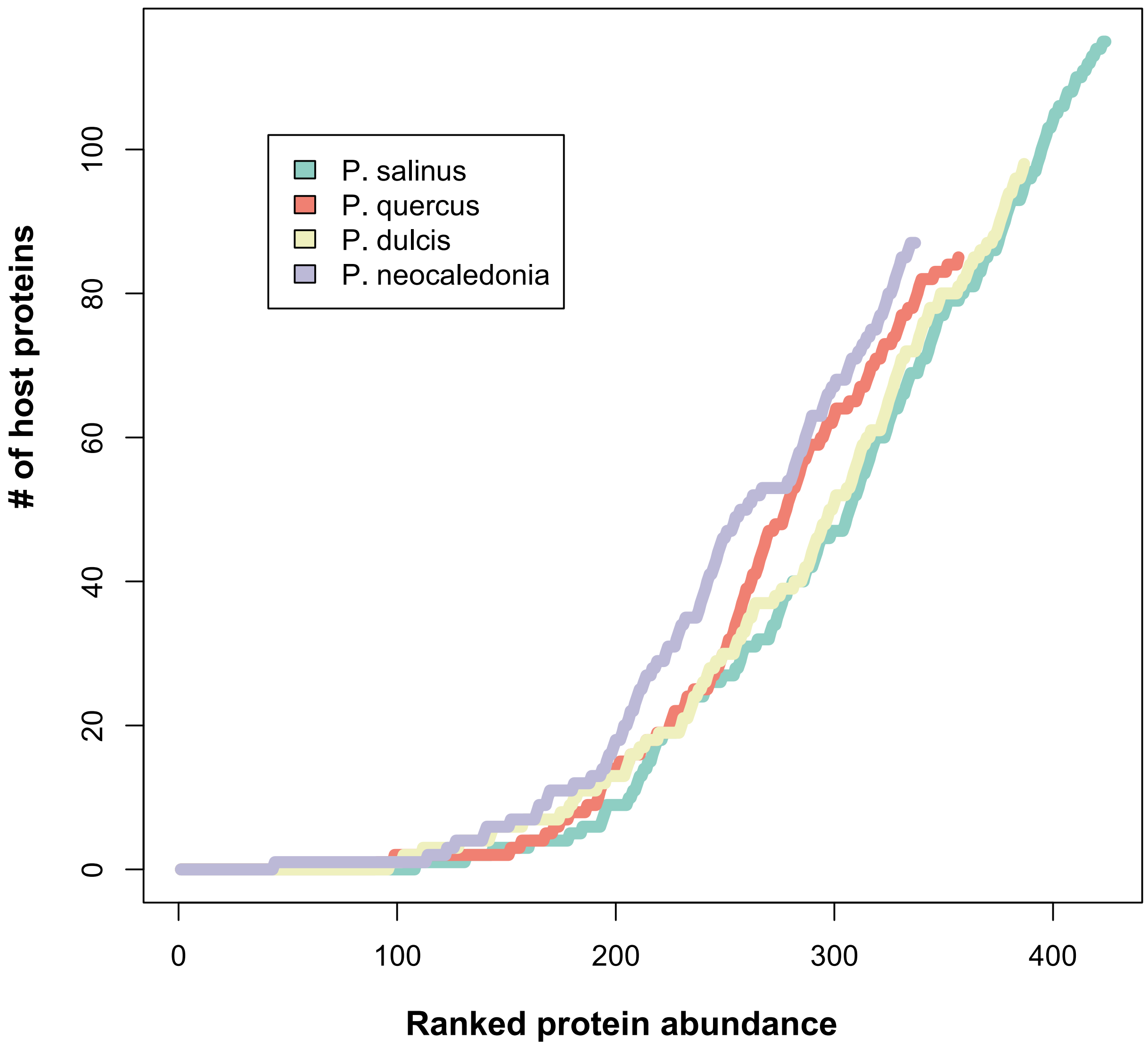

Supplementary Fig. 10. Increasing proportion of host proteins identified at low abundance. Cumulative distribution of the number of identified A. castellanii proteins in the virion as a function of their ranked protein abundance.

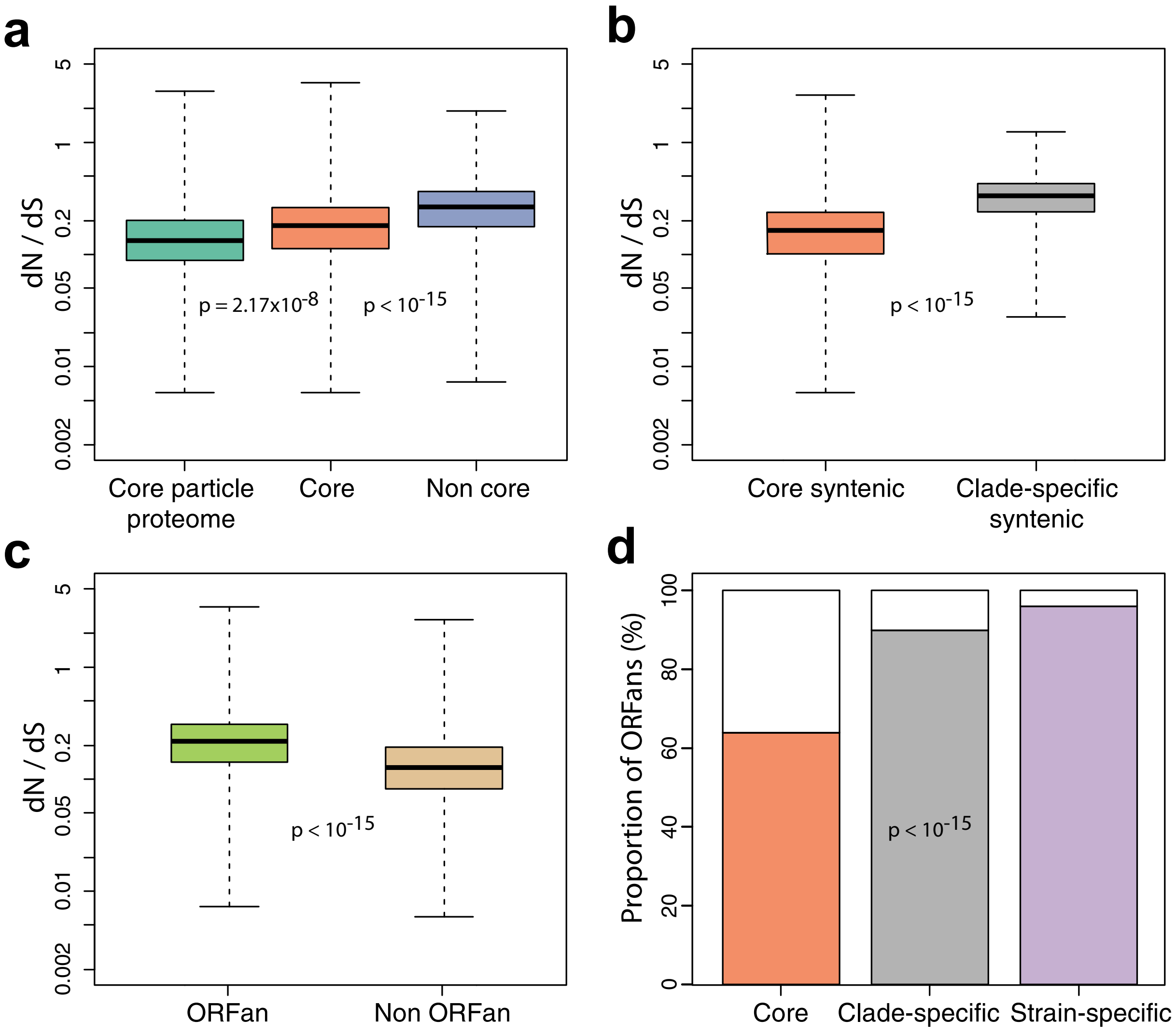

Supplementary Fig. 11. Selective pressure among different classes of genes estimated from dN/dS ratios. a, **b**, **c**) Box plots of the dN/dS ratios among different classes of genes. Pairwise p-values correspond to Mann-Whitney tests. **d** Proportion of ORFans, i.e. proteins without significant BlastP match in the NR database (Evalue cutoff = 1e-5) in different classes of genes. The pvalue is calculated using the chi-squared test. Box plots show the median, the 25th and 75th percentiles. The whiskers correspond to the extreme data points.

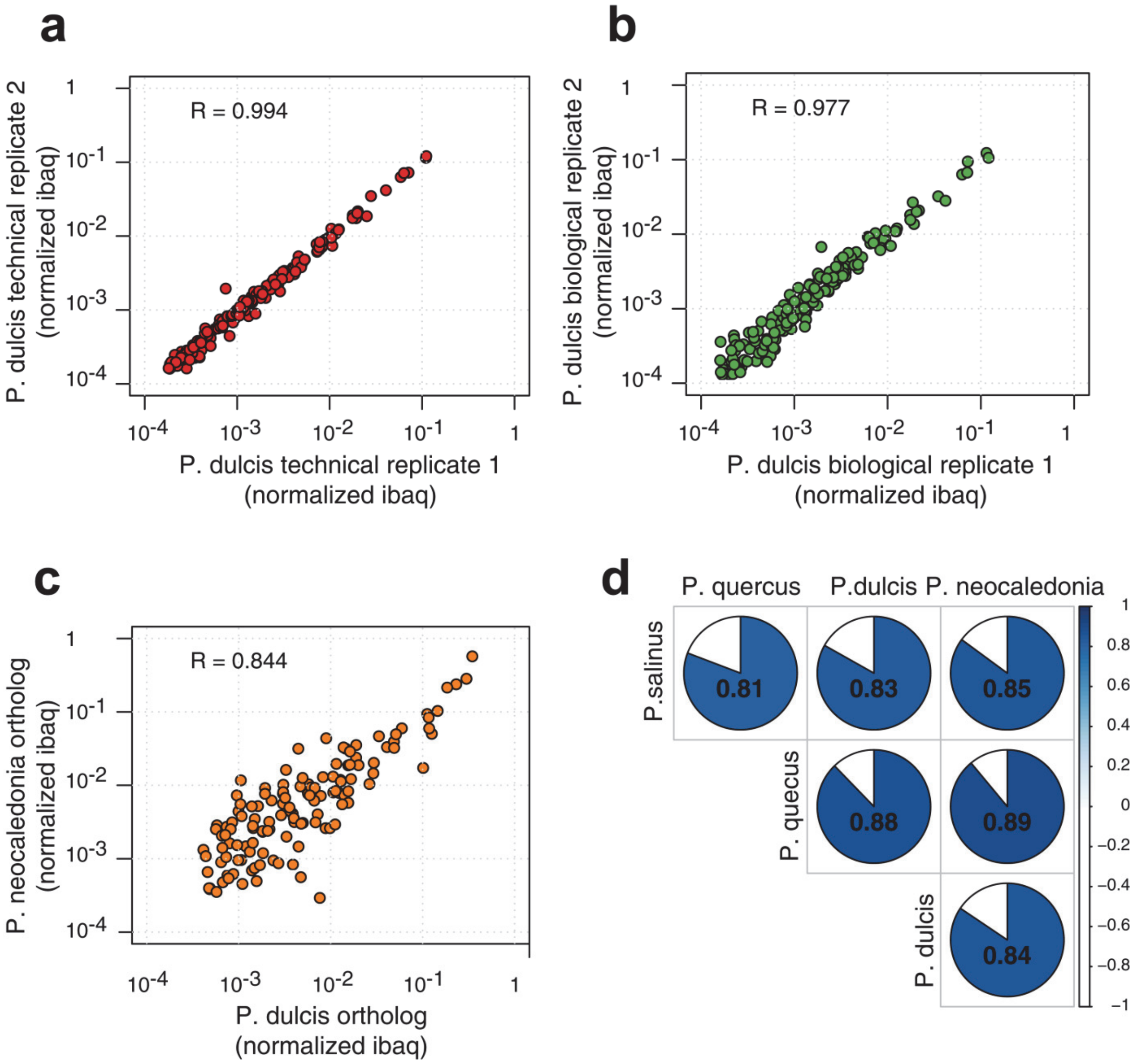

Supplementary Fig. 12. Comparative particle proteomics of four pandoravirus strains. a Correlation of two technical replicates of P. dulcis virion proteome. **b** Correlation of two biological replicates of P. dulcis virion proteome. **c** Correlation of P. dulcis and P. neocaledonia orthologous proteins in the virions proteomes. **d** Pearson correlation coefficients of all pairs of pandoravirus orthologs present in the virions proteomes.

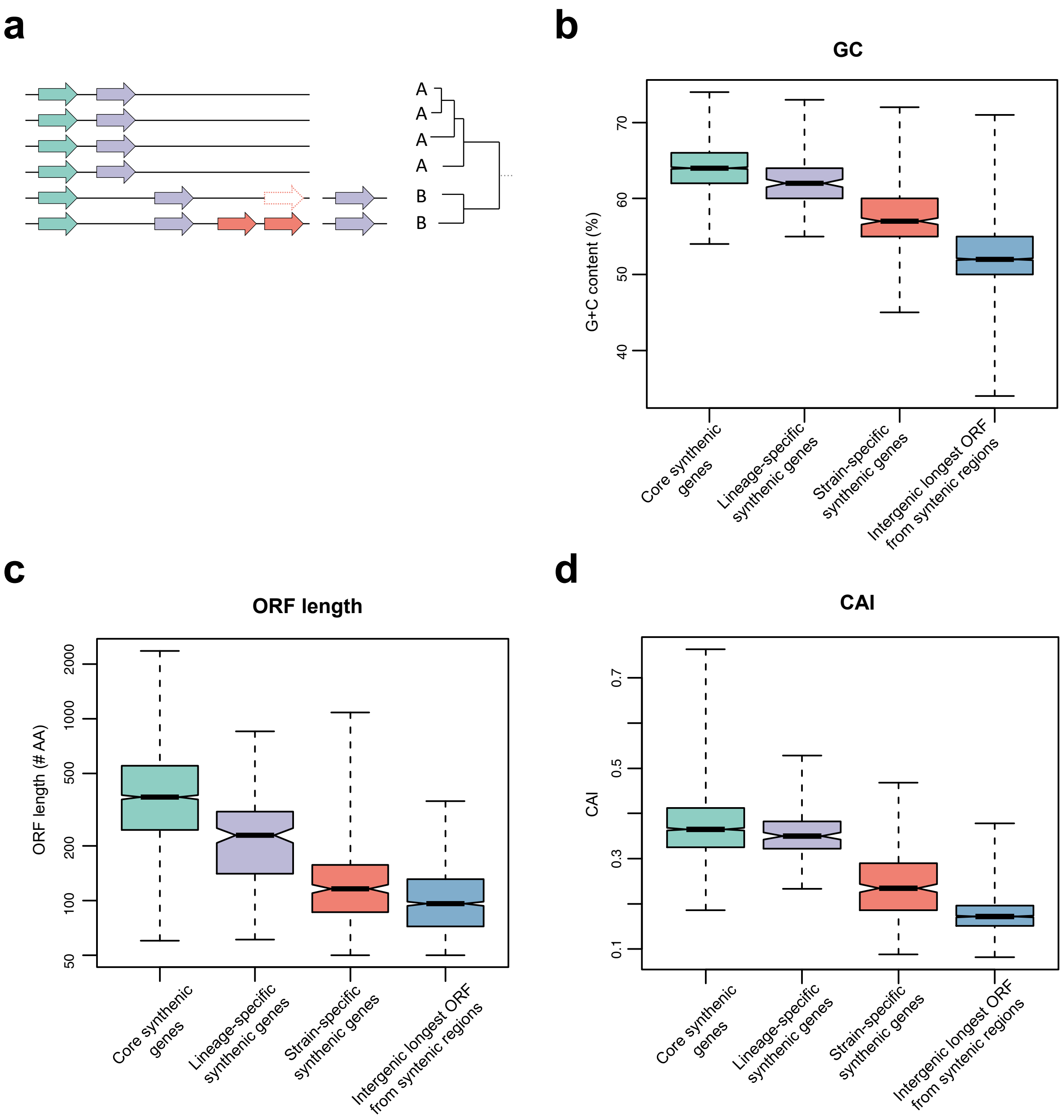

Supplementary Fig. 13. Different genomic features between core, clade-specific and strain-specific genes. a Diagram representing the different classes of syntenic genes under study: core genes (in green), clade-specific genes (in purple) and strain-specific genes (in red). **b** G+C content of the different gene classes. **c** ORF length of the different gene classes. **d** Codon usage bias calculated as the codon adaption index (CAI) of the different gene classes. Box plots show the median, the 25th and 75th percentiles. The whiskers correspond to the extreme data points.

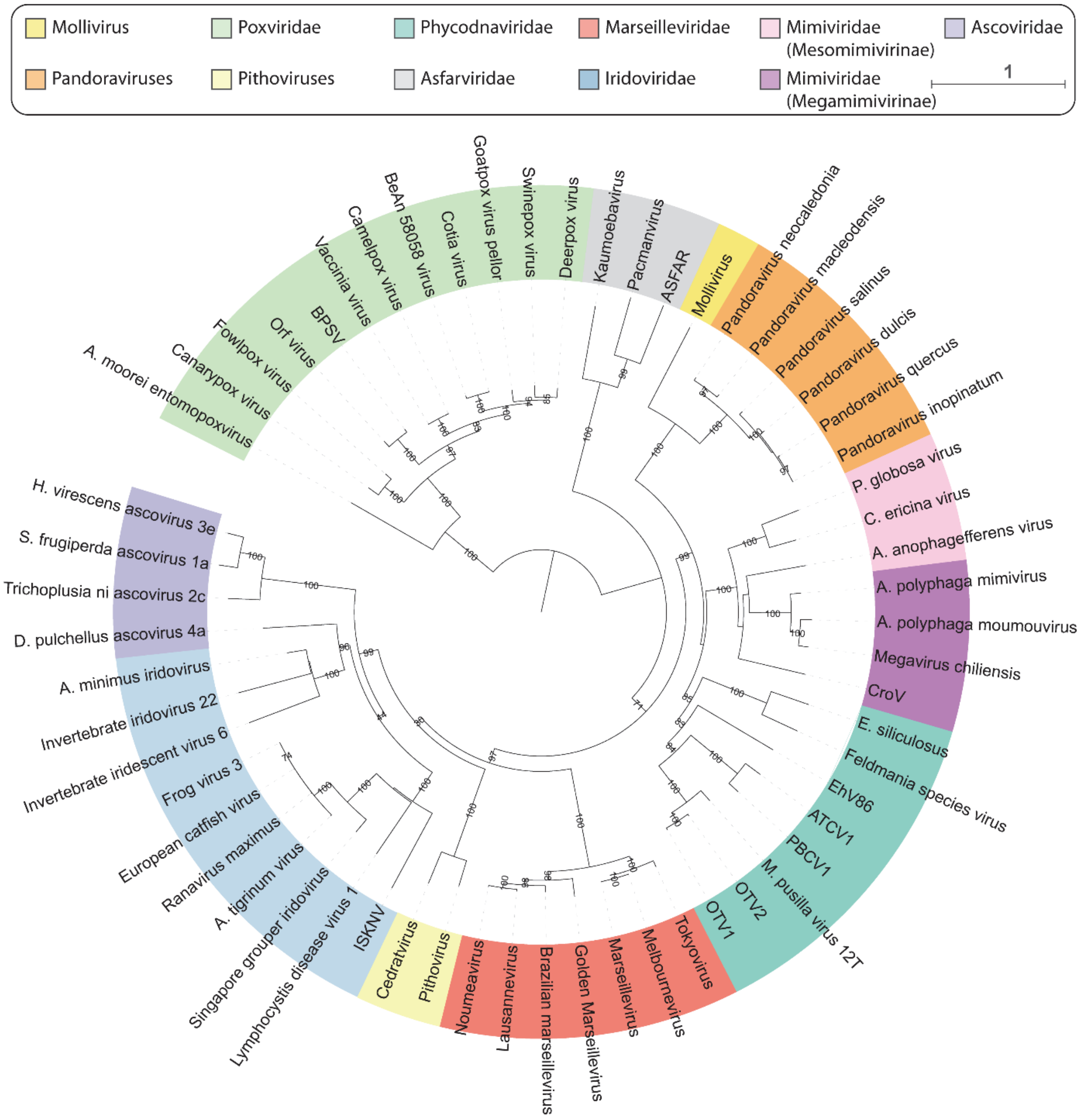

Supplementary Fig. 14. Phylogenetic tree based on the DNA polymerase B of large DNA viruses. Long virus names have been replaced by acronyms (from top, clockwise). OTV1: Ostreococcus tauri virus 1; OTV2: Ostreococcus tauri virus 2; EhV86: Emiliania huxleyi virus 86; ASFAR: African swine fever virus; CroV: Cafeteria roenbergensis virus BV.PW1; BPSV: Bovine papular stomatitis virus; ISKNV: Infectious spleen and kidney necrosis virus. Bootstrap values were estimated using the aLRT SH-like branch support method from the PhyML package[32].

Supplementary tables

Supplementary Table 1. Genome annotation statistics

Supplementary Table 1a. Gene structure statistics

	Protein-coding genes					Non-protein coding genes			
**Pandoravirus Isolate**	**N genes with intron**	**Intron size (nt)**	**Number of intron/gene**	**UTR size (nt)**	**Protein size (aa)**	**N anti-LncRNA**	**N inter-LncRNA**	**Total N LncRNA**	**LncRNA size (nt)**
P. salinus	108 (7.5%)	218 ±170	1.34 ±1.28	160 ±113	385 ±237	158	56	214	789 ±528
P. dulcis	113 (10.5%)	200 ±140	1.27 ±0.7	182 ±125	398 ±253	237	31	268	898 ±624
P. quercus	112 (9.4%)	174 ±123	1.31 ±0.86	152 ±125	398 ±243	150	7	157	1299 ±736
P. neocaledonia	140 (13%)	186 ±133	1.19 ±0.62	197 ±154	383 ±252	208	41	249	1135 ±741

Supplementary Table 1b. Genome structure statistics

Pandoravirus isolate	Transcribed genome fraction	Coding genome fraction	Theoretical genome span (nt)/ encoded protein
P. salinus	84.7 %	66.7%	1730
P. dulcis	87 %	66.8%	1784
P. quercus	84.4 %	68.2%	1753
P. neocaledonia	82.7 %	62%	1853
Megavirus chilensis	-	90%	1136

Supplementary Table 2. Pairwise global protein sequence conservation based on 1:1 orthologs super-alignment

	**P. salinus**	**P. inopinatum**	**P. quercus**	**P. dulcis**	**P. macleodensis**
**P. inopinatum**	73%				
**P. quercus**	74%	88%			
**P. dulcis**	70%	71%	72%		
**P. macleodensis**	54%	54%	55%	55%	
**P. neocaledonia**	54%	54%	54%	55%	76%

Supplementary Table 3. Core proteome protein clusters with detected functional motifs

Motif/function	P. salinus	P. dulcis	P. quercus	P. neocaledonia	Comment	Rank in P. salinus
Thioredoxin-like fold domain	psal_cds_383	pdul_cds_428	pqer_cds_374	pneo_cds_321	One-copy, very abundant	4
Disulfide isomerase	psal_cds_411	pdul_cds_453	pqer_cds_404	pneo_cds_343	One copy, abundant	12
Collagen triple helix repeat	psal_cds_145	pdul_cds_186	pqer_cds_134	pneo_cds_97	One copy, abundant	18
Nucleoporin_FG2	psal_cds_397	pdul_cds_436	pqer_cds_388	pneo_cds_326	One copy, abundant	21
Cupin domain	psal_cds_212	pdul_cds_262	pqer_cds_208	pneo_cds_174	One copy, variable	31
Endoribonuclease L-PSP	psal_cds_874	pdul_cds_721	pqer_cds_801	pneo_cds_634	One copy, abundant	32
PAN/APPLE-like domain	psal_cds_193	pdul_cds_240	pqer_cds_184	pneo_cds_153	One copy, abundant	36
ERV-family thiol oxidoreductase	psal_cds_384	pdul_cds_429	pqer_cds_375	pneo_cds_322	One copy, abundant	44
Acid phosphatase class b	psal_cds_162	pdul_cds_201	pqer_cds_150	pneo_cds_120	One copy, abundant	45
Casein kinase	psal_cds_194	pdul_cds_242	pqer_cds_185	pneo_cds_154	One copy, abundant	61
Trypsin-like serine protease	psal_cds_272	pdul_cds_320	pqer_cds_264	pneo_cds_225	One copy	65
Collagen triple helix repeat	psal_cds_92	pdul_cds_769	pqer_cds_723	pneo_cds_146	Many paralogs	77
NAD-dependent amine oxidase	psal_cds_628	pdul_cds_592	pqer_cds_534	pneo_cds_425	One copy	97
oxidoreductase	psal_cds_1260	pdul_cds_990	pqer_cds_1102	pneo_cds_908	One copy	101
FAD/FMN-containing dehydrogenase	psal_cds_1132	pdul_cds_867	pqer_cds_971	pneo_cds_812	One copy	121
Patatin-like phospholipase	psal_cds_323	pdul_cds_371	pqer_cds_312	pneo_cds_276	One copy	123
Lipase/esterase	psal_cds_1390	pdul_cds_1046	pqer_cds_1162	pneo_cds_964	One copy, low	130
AldR family transcriptional regulator	psal_cds_118	pdul_cds_758	pqer_cds_834	pneo_cds_654	Two copies	141
Ser/thr phosphatase 2c	psal_cds_235	pdul_cds_285	pqer_cds_229	pneo_cds_194	One copy, low	153
