# Supplementary material for: Diversity and evolution of the emerging Pandoraviridae family
Source: Nat Commun. 2018 Jun 11;9:2285. doi: 10.1038/s41467-018-04698-4 (PMC5995976; doi:10.1038/s41467-018-04698-4)
Supplement: Supplementary file 3 — Description of Additional Supplementary Files [file 41467_2018_4698_MOESM3_ESM.pdf]

Description of Additional Supplementary Files

File Name: Supplementary Data 1
Description: *Proteome compositions of the virions of P. salinus, P. quercus, P. dulcis and P. neocaledonia*

File Name: Supplementary Data 2
Description: *A. castellanii gene sequences used to compute the Codon Adaptation*

File Name: Supplementary Data 3
Description: *Reannotated P. inopinatum genome in Genbank format*

File Name: Supplementary Movie 1
Description: *Movie of pandoravirion exocytosis: The movie presents an amoeba infected by a pandoravirus 5H post infection at MOI 1000. Magnification factor is x1008. Speed is x4. The red arrow points to an exocytosis event.*
