# Supplementary material for: Diversity and evolution of the emerging Pandoraviridae family
Source: Nat Commun. 2018 Jun 11;9:2285. doi: 10.1038/s41467-018-04698-4 (PMC5995976; doi:10.1038/s41467-018-04698-4)
Supplement: Supplementary file 5 — Supplementary Data 2 [file 41467_2018_4698_MOESM5_ESM.rtf]

A. castellanii genes used as reference for the cai calculation

>g1007-20481-25408
ATGTCGGTCATTGGCATAGATCTGGGCAACAGAAATTCGATCATCGCCGTTGCCCAGCGA
GGCGGCATCGACATCGTGTTGAATGAGTGCTCCAACCGCCACACGCCGTCGATGGTGGGC
TTTGTGGGTCAGGAGCGCTCGATCGGCGAGGCGGCCATGGTTCAGTACGCACGGAACATC
AGAAACACTGTGGCCCAGGTCAAGCGCCTGATCGGTCGCAAGTGGAATGAGAAGGAGCTG
CAGGATGAGCTGCCTCTGCTGCCCTTCAAGGTGAAGGAAATCGGAGATGGCAAGATCGGA
ATCGAGGTGAGCTACAACGGGGAGCAGGTCACCTTCACCCCCGAGGAGGTGACGGCCATG
GTGCTGGTGCAGCTGAAGGCTATCTCGGAGAACTACCTCAGGACCAAGGTCAAGGACGTC
GTCATTTCCATCCCCGGCTTCTTCACCTCCGCGCAGAGGAGAGCTCTGCTCGACTCTACG
CAGATTGCGGGGCTCAACTGCCTGAAGCTCGTCAACGAGATCACCGCCACCGCGATCGCC
TACGGCATCTACAAAACCGATCTCCCCGAGAGCGACCCCATGCACGTCATGTTTGTGGAC
ATTGGCGATTCTCACATGAGCGTGGGCGTCGTCGCTTTCCAGAAGGGCAAGCTCCGGGTA
TTGAGCACGGCCTACGATAGAACGCTGGGCGGCAGGAATTTTGACCGGGCTTTGGCCGAC
CACTTCGCCAAGGTCTTCCAGGACAAGTACAAGATCGATGTCAAGTCCAACATGAAGGCC
TGGATCCGCCTCGAGACTGCTTGCGAAAAGGTGAAGAAGATTCTCTCCGCCAACTCGCAG
GCACCCCTCGCCATCGATTCTCTCATGGAGGACATCGACGTGAGCGCCATGGTCACCAGG
GAGGACTTTGAACAGTTCTGCGCTCCCCTCTTCGAGAGGCTGCAGGAGCCCCTCAAGCAG
GTTCTCGCGGAGACTGGTCTGTCCGGCAGCAGCTTGCACGCGATTGAGCTTGTCGGGGGT
GCCAGTCGCATGCCCCAGCTGGCCCCCATCATTTCCAAGCTGACCGGCAAGGAGTTCTCG
CGCACCATGAACGCCGAAGAGTCTGTTGCCCGAGGTGCGGCGTTGCAGTGCGCTATGCTC
AGCCCCACCTTCCGTGTGCGCGAGTTCAAGGTGGAGGACAGCAACCCCTACCCCATCAAC
CTCGTCTGGAAGGACCTCGACTCCGAATCCATGGAGACCGAAGAGCCCACGGAAATCTTC
CCGAAGAACTGCGTGGTGCCGGCCATGAAGATCATCACCTTCCCGCGAGGCAAGCCCTGC
GAGATCAAGGCCAGCTACGCGCCCACGGCGGATCTGCCCCCGGGCACCTCCGCCTTTATC
GGCAAGTGGGTCATCCCCACCGTCCCTCCCACGGAGTCAGGCGAGTCGGCCAAGGTTCGC
GTCAAGGTCAAGCTCGACGGGAATGGTATCTTCTCCGTGGAGTACGCACAAATGATCGAA
AACGTGGTCGCCAGCAAGGAGGAGGACAAGAAGGAGGCCGCCGCCGCCGCCGCCGCCGCC
CAGTCGCCCAAGGACGACGACGACAAGAAGGCCAAGGAGGGTGACGAGAACAAGAGCGCA
GAGAAGAAGGAGGACGCGAAGATCACGACGAAGCGGACGAACCTCTTTATCCAGGAGGTG
ACCGACGGCATGCCCCCCGCCCAAATCCAGGCCCTCGCCGCCGAGGAAAAGCAGCGTCTG
GCCAAGGACACCGAACTCCGGGAGACGGCTGAGGCAAGGAACGCGGTGGAGGCCTACGTC
TACGACACGCGCAGCGACCTGAACGGCAGCCTTCTTCCCTTCGTCCTCGAGGCCGACAAG
GACGCCTTCTACAGCCAGCTCAACGAGGCCGAGGATTGGCTCTACGGCGAAGGTGCCCAG
GCGACAAAGCAGGCCTACCAGGAAAAGCTGGCGCAGCTGAAGAAGGTCGGTGAGCCCATC
CGTATTAGGCGACGAGAGGCTGAGGATCGTGACGATGCCATCGAAAAGCTCCGCCAGGCC
ATGGAGAACTACCGCCTTCTCGCTCAGTCAACCGACCCCAAGTACGAGCACATCCCTCAG
GAGGAACGCCAGAAGGTACTCAACAAGGTCAAGGAAGCCGAGGACAGCGTGCTGCCAAAG
GCAGAGCAGCAGAAGACGCTTCCCTCCACCGCCGACCCTATCATCTGGGTTGCTGACATC
ACTCACACCAAGGAGAACTTGGACACCTTTGTCTCGACCATCATGAACAAACCGAAGCCC
AAGCCTGCTGCCCCTGCGCCCAAGGAAGAGCCCAAAAAGGAAGAAAGTGCCCCTAAGGAG
GAAACTGCTGAGCCAACTGAGAAGAAGGAGGCTGACATGGATGTGGAGCAGTGA
>g1007-25557-28438
ATGGGTCGTCGCCCTCTTTTCAGCGCGTGCCTCGTGGTCCAGTCGAATCGGGGAGCAGCG
GCCACGTTGTCTGGCCTGCGGGGAGTGTGCTCATCTTCTTCCCACAACGGAACGGGGCGA
ATTGTTGCCAGCCTTACGTCGCATTCGCGTAGTGGCCTCAATGTTCTTCCTAAGCGATTC
TACGCAACCGCCGAGGTCAAGACCCAAGCGGTGCCCCCCATGGGTGACTCGATTACAGAG
GGCGAGCTCAAGAATTGGTCCAAGGGGGTTGGCGAACACGTTGCCGTGGACGACTTGGTT
GCAGTGATCGAAACGGACAAGGTGGCAGTTGAGATTCGCGCCAAGGAGGCTGGAGTGATC
AAGGAGCACTTTGCCGAGGAAGGCTCGACCGTGTCCGTGGGTGCGCCCCTCTTCGCCTAC
GAGGCCGGAGCTGAGGCGCCCAAGAAGGCCGAAGTTCCCAAGGAGGAGCCGGCCAAGAAG
ACCGAGCAAGCGCCGAAGCCAGAGGCTGCCGCTCCTAAAGCGGAAGCTCCCAAGACCGCG
GCTGCCCCTGCTGCTGCTACCGAGGGCAAGGCTGCCCCCAAGGCGGAGGCCTCGAAGGCA
CCTGCCCCCAAGACCGCTTCCCCCGTCGCCGGAGGTGAGCGCAAGGTCAAGGTCACCCGC
ATGCGGGAGCGTATCGCGCAGCGCCTCAAGGACGCACAAAACACCTACGCCATGCTCACC
ACCTTCCAGGAGGCCGACATGTTCAACCTCATCAACATGCGGGAAGATTTCAAGGAGGAG
TTCCAAAAGAAGCACGGCGTGAAGCTGGGCTTCATGTCCGCCTTCGTCAAGGCCTCTGCC
GCCGCTCTCAAGGAGATTCCCGCTGTCAACGCAGTGTACGACGGCTCGAACAGAGAGATC
ATCTACCGGGACTATGTCGACATATCTGTTGCCGTGGCCACCCCCAGGGGCCTCGTGGTG
CCGGTGCTGAGGGACTGCGATCACCTCAGCTTTGCTGACGTCGAAAAGCGGCTGAGCGAA
CTCTCGGTCAAGGCCCGCAAGGACGAAATCACGCTCGAGGAAATGGCCGGCGGCACTTTC
ACGATCTCCAACGGTGGCGTTTATGGTTCGCTGATGGGCACGCCCATTATCAACCCACCG
CAGTCCGCCATTCTTGGCATGCACGCCATCAACAAGCGCCCCGTTGTCGTCAATGATCAG
GTCGTCATCAGGCCCATCATGTACCTGGCGCTGACATACGACCACCGTTTGATCGACGGC
AAGGAAGCGGTGACCTTCCTGAGGCACATCAAGCACAGCATTGAAGACCCTCGCCGCCTC
CTCCTTGAGCTGTAA
>g1007-28487-29308
ATGTCCACCCAAGTCAAGGTCAGCGCTGTCGTGAACGCACCCGTCGAGAACGTGTGGAAG
GAGCTCAGGGACTTCACCTTCCCCGCACGCCTCATCTCCACCGTCGAGAGCGCCGAGCTG
CTGGACGGTGCGCAGGGCACCGCCGTTGGCGGGGCCAGGAAGCTCAAGTGGAAGAGTGGT
GAATCTGCCACGCAGGTGCTGCTGGAGCTTTCCGACATCGAGCGCCGTGCTGTGTGGGAA
ACCACCCTCGCCGAGCCTGACACCGAAGTGGCGGCCACTATCTCCACCCTCCATGCCCTC
CGCATCACGGAGAACAACTCGACCCTTCTCACCTGGTCGGCTGAGTTCAGTGCCGACGTG
ACGCCTGACTTTATCAAGTTCCAGCACAAGGCCCTCCAGGACAACCTTGCCGAGATCAAG
GCCCACTTTGCCCACTAG
>g1007-33221-34277
ATGAAAATCCGTTCGTTCGTTTGTCGTGGTGCCGTGTGTCCGGTGCGCACCGGCCCCAGC
TCCGCCCCGCAAGAGTCAATCTGCCTCCGCGACACGCTGGATGTTTCTGTGGATCCCGCG
AGGCAGACCAACCGCCAGCTACGGCTCGCTGCAATCCGCATCCACAACCATCGCCCCCAT
GCAGAGACCAAGCTCGCCGCCCGCCTGGATTTATCCTCCCATGAACGAGAAACTGCCCCC
CCCCCGGCCGCCAATCCTATGATTTTGACAAAAGATAAAGGAAAATTTTTAAAAAAATTC
CTTGCTATCGCCAGGATTGATTCTAAGGTTGTGCAATCAACAGTAATGTCTCGCTCGCTC
ATCACCCGCAGCCTTCCCCTTCGTTCCTCTTCCCATGGTGCCCTTGGTGGTGCGAGGCCT
GTATTCTCCGAGGTGCCCCTGAAGGAGGTGCCGTCGTACGTGAGGCTCCATGTGCTCGGA
AACACCAGGCGCCTCACCAAGGAGTTCGTCCAGTGGTACTGGAGGACTTACTTCCACGCT
GGCAAGGCCGACCCCATCATTCACGGCATCCTCCTCGTCTCCGGCGTTGGCTATCTCCTC
CACAACCACTGCCGGTCTCACCGTACGAGTTACCATCTTGACACACAGGGCACGGATCGC
ACCCATAAGCGCTCAGCCCCGCGCACACGAGCATTTCTTTGCCGCGTAAGAAGGATGTCG
AGCCCCAACGTACAGTTATGGGGAGGAACTGCCTTCCTCCTCCCTAATAAACAAGTCCCC
TTTCCTCCTGCTGGCCCACGCGGCCGCAAGTTGTCGGTTCCAGACCGGGGGGGGGGGTAG
>g1007-46746-48880
ATGGCCGCCATTCCCGCGTCAACCAGCAGCAGCAGCAGCAGCAGGCGAGACCTCTATCCG
CCCATCGAGCCCTATTCCCACGGACGGCTGAGGGTGAGCGACATCCACGAGCTCTACTAC
GAGTTGTGCGGCAACCCCAACGGCAAGCCCGTCGTCGTGGTGCATGGCGGCCCTGGCGGC
GGCATCGCTGCCTACTACAGGCAGTACTTTGATCCGGAGGCCTACAAGATCGTCCTGTTC
GACCAGCGCGGTTCGGGCCAAAGCACCCCCTTCGCCTGTCTCGAGGAGAACACGACGTGG
GACCTCGTGGAGGACATGGAGAAGCTGCGGCAGCACCTGGCCATCGACAAGTGGCTCGTC
TTCGGCGGCTCCTGGGGCTCCACCCTCGCCCTCGCCTACGCTGAGGCCCACACCGAACAG
GTGAAGGGCCTGGTGCTGCGGGGCATCTTCGGGCTGCGGCGCAGCGAGCTCCTGTTCTTC
TACCAGGAGGGCTCCTCGTGGCTGTTCCCCGATGCCTGGGAAAAGTACCTGGCCCCGATT
CCGGAGGTGGAGCGGGGAGACCTGATGTCGGCCTACTACCGCCGCCTCACCGGCAACGAC
GACAAGGTCAAGCAGGAGTGCGCCACCGCCTGGAGCGTCTGGGAGATGACGACGAGCCGC
CTCTACGTGGACCCCGACTACGTGGCGCGGGCCGCCGAGGACGACAAGTTCGCCCTCGCG
TTCGCGCGCATCGAGTCGCACTACTTTGTGCACGGCGGTTTCTTCAAGGAAGACGGACAG
ATCATCAAGGAGGCCGCCAAGCTGGCCCACCTCCCCGTGACCATCGTGCAGGGACGCTAC
GATCTCGTGTGCCCCATGAAGACCGCGTGGGACCTGCACAAGGTGCTGCCGTCGTCCGAG
CTGGTGGTCGTTCCCGACGCGGGCCACAGCGCCAAGGAGCCCGGCATCGTGGACGGACTG
GTGCGCGCCTGCGACAAGTACCGCGACCTCTGA
>g1007-177784-179557
ATGGGTAAGGTGCGCGTACGTATGATCAGTGCGAGGAACCTCGTCTCGGCCGATACCAAC
GGCTATTCGGACCCATACGTCGTGATCGCCGTGGCCGGCGAGGAGAAGAAGAACTTCAAG
AAGACCGCCACCATCAAGAAAAGCCTGAACCCGAGCTGGAATGAGTCGTTCGAGTTCGAG
CTGGGCTCCACCCCCACCCACCGCCAGGTCACCTTCCACGTCTACGATTGGGACATGCTC
TCGTCGCACGACTCGCTGGGCAACATCAGCCTGCCGGTGGACGATCTGTTCAAGGGCGAG
GAGAAGCTGCAGTGGTACACCCTGCACAACACCAAGCACGGCGAGATCCAGGTCGGTTTG
ACTGCCCTTGACTTTGGCATCGACCGAGCGGCCCAACAGCACCAGCAGTTCCAGCAGCAC
CAGCAGGGCTATGCCCCCCAGCATCAGCAGGGCTACCCCCCGCAGCAGCAGGGCTATGCC
CCGCAGGGCTACCCCCCGCAGCCCCAGCACGGCTACCCCCCGCAGCAGCAGGGCTTCCCT
CCTCAGCAGGGCTTCCCTCCTCAGCAGGGCTATCCCCAGCAGCAGCAGGGGTACCCGCCG
CAGGGCTACGACCAGGGCTACGGGCAGCACCAGCAGGGCTACCCGGCCCAGGGACTCGCC
CCGGCGCCGGTCTATGGCCAGCCCGGCCACGGCGGCTCGCACTCGCCGGCCGTAGCACAC
GGCAAGGAGCACAAGAACAAGGACGGCAAGAAGAACAAGGACAAGAAGAAGAAGGACGGC
AAGGTGGGCAAGAAGGCCGGCAAGGCCGCCGGCACCTTCTTCAAGGGCGTCGGCACCGTT
CTCTCCGCCGCCGTCGAGATCGGCAGCGATGACTGA
>g1007-210596-212658
ATGGCGATGGATGAAAGAAAGCAGAAGGCGCTGCACGATTTCAGGGCCAAGCTACTGCAG
CACAAGGAAGTGAACGATCGAGTTCGTTCGATGAGGGAGGACTTCAAGACGCAGAAGAGA
GCCTATGACAAATCTGAGGATGATCTCAAGGCTCTCCAGAGCGTGGGTCAGATCATCGGT
GAAGTGCTGCGCCAGCTTGACGATGAGAAGTTCATTGTCAAGGCCTCGAGCGGTCCTCGC
TATGTGGTAGGTTGCCGCAAGCGCGTGGAGAAGAAGAAGCTTATCGCTGGCACCCGTGTG
GCTCTCGACATGACCACGCTCACCATCATGAGGGCGCTGCCCAGGGAAGTCGACCCTGTG
GTGTTCAAGATGTTGTCGGAGGACCCTGGCGAGGTGTCCTACTCCTCCATCGGTGGTTTG
AACGAACAGATTCGCGATCTTCGTGAGACGATCGAGCTGCCCCTCACGAACCCTGAGCTC
TTCCTGCGCGTGGGTATCAAGCCCCCGAAGGGAGTGCTCCTCTACGGTCCGCCCGGTACC
GGCAAGACCCTGCTCGCCAGAGCCATGGCGCACAACGTGGAGGCCAAGTTCATGAAGGTC
GTGTCTTCGGCCATTGTTGATAAATACATCGGCGAGAGCGCGCGTCTGATCCGAGAGATG
TTCGGCTATGCCCGTGAGCACCAGCCGTGCATCATCTTCATGGACGAAATCGACGCTATT
GGAGGCAGGCGTTTCTCGCAGGGTACCTCCGCCGACAGAGAGATTCAGAGGACGCTCATG
GAGCTTCTCAACCAGATGGACGGTTTCGACGAGCTGGGAATGGTGAAGATCATTATGGCC
ACCAACCGGCCAGACGTACTCGACCCAGCCCTGCTTCGCCCCGGTCGTCTCGACCGTAAG
ATCGAGATCCCTCTGCCCAACGAGTCGGCGCGTCTGGACATCATCAAGATCCACTCCGAG
TCGATCACCAAGCACGGCGACATCGACTTCGAGTCGGTGGTCAAGCTTTCCGACGGCTTC
AACGGCGCCGATCTGAGGAACGTCGTCACGGAGGCCGGCATGTTCGCCATCAGGGCCGAG
AGGGACTACGTCATCAGCGAAGACTTCATGAAGGCCGCGCGCAAGCTCGCCGAGAACAAG
AAGCTCGAGAGCAAGCTCGAGTACAAGAAGCTCTAA
>g1007-257770-259328
ATGACGTCAAAGGGCAGTAAGATGCTCCAGTTCATCAACTATCGTATGAAAATTACGATC
GAAGATGGACGGACGCTGGTGGGCAAATTCATGGCCTTCGACAAACACATGAACCTCGTG
CTCGGCGATTGCGAGGAGTACCGAAAGATCACGCCCAAGGGCAAGGGCAAGGAGGAGCGA
GAGGAGAAGAGAGTTCTCGGTCTGGTCTTGATTCGGGGAGAGAGCGTCATTTCGCTCAAC
GTCGAGGGTCCCCCTCCCATCGAGGAGAACCGATTGAAGGGCATTCAAGTCGGTATCCCC
GGCCCCGGCGTGGCCAGGCCCGCTGGACGAGGTATTGCCATGCCCCCGATGGAGGGCGCG
CCCGCTCCCGGCCTGTCCGGCGCCCCTGTGCGTGGCGTGGGTGGACCCGCTCCCTCGATG
ATGATGCCCCAGGGCGGTCGCGGCGGCCCCATGGGTATGCCCTTCGGTGCTCCCGGCATG
GGCAGAGGAATGCCCGGCATGGGTCCGCCTCCCGGCATGGGCGGCCCCGGTATGCCGCCG
ATGGGTCGCGGCATGCCCCCGATGGGTGGCCCGCCTCCCGGCATGATGGGCGGCCCGCCC
CCCGGCATGCCGCCGATGGGTCGAGGAATGCCAGGAATGGGTCCGCCTCCTGGCATGGGC
GGCCCCGGTATGCCGCCGATGGGTCGCGGCATGCCCCCGATGGGCGGCCCGCCGCCTGGT
ATGCCGCCGATGGGCCGAGGCATGCCTCCGATGGGTGGTCCGCCGCCTGGTATGCCGCCG
ATGGGCCGAGGCATGCCCCCGATGGGTGGTCCTCCTCCCGGCATGCCCCCATACCGCCCC
TCGTAA
>g1007-288057-288731
ATGAGCGACGCCAAGCCCGAGAAGAATGAGGAGGAGGAGTTCAACACTGGACCCCTCTCG
CTGCTGGCCGAGTCGGTGAAGACCAACTCGCAGGTCCTCATCAACGTGCGCAACAACAAG
AAGCTGCTCGGCCGTGTCAAGGCGTTCGATCGTCACTGCAACATGGTGCTGGAGAATGTG
AAGGAGATGTGGACCGAGGTGCCCAAGAAGGGCAAGGGCCAGAAGAAGGCCAAGCCCATC
AACAAGGACCGGTTCATCAAGAATATGTTCCTCCGAGGGGACAGCGTCATCGTCGTCCTC
CGCAACCCGCTGGCCACCTCTGCCGCCCCCAAGCCCATCACCGCCGCTTCCTCCTAG
>g1007-330056-330760
ATGGCATCCAAGTTTGGCGGAGCTCCCAAGTGCCCTCGGTGCGCCAAGTCGGTGTACCAC
GCCGAGCAGATCATCAGCGCCGGCCGAGAGTGGCACAAGAACTGCTTCACCTGCCGCGTG
TGCAACAAGAGGCTCGATTCGACCACCGCCACCGATCGCGAGGGCGACGTCTACTGCAAG
GGCTGCTACGCCACCAACTTTGGCCCGCACGGATTCAGGGGCGGCAACGCCGGCGGCATC
ATGCACACTCAGGGCAAGGAGGACGTGATCAGCTTTGGTGGCTCGGCCCCTGCGCCGGTG
GCTGCTGCTGGCGGCGGCGGCGGCGGCGCTGCGTTCTGCTCGGGTTGCGGTACCAAGGCG
AGCGGCGGTCGGTTCTGCTCCGGCTGCGGCAACGGTCTCTAA
>g1007-388106-392098
ATGAAGTCCATCTTTTCCCTACTTCTTGTGGCCCTCTTCGTGGCCGCCGCCTCGGCGCAG
AGCGATAAGACCATCGTGGAGGTGCTGGCCCAGCAGCCCCTCTACTCGCAGGTGAACGCC
AGCCTCCAGGGCAAGCAGATCGAGGCCTTCCTGAACAACGTCAACGTCACCGCTACTCTG
CTTCCCCCCGTGGACCAGAAGACTCCCCTCAATCTCACCGACGCCCAGATCAGCTACCAC
GTGCTGAACGGCACCGCGCTCGACCTGACCCAGGTCAAGCAGGGTGAGCTCTTCGTCACT
GCTCTCAGCCTTGCTTCCCTCAAGGACGGTTTCCAGCGCGTCAAGGCTTCGCTTGGTTCC
AACGACACTCGCGTCTTCCTCGGCCCCAACTCGGTCGCGGTTAACGCCACCGCTCTGGAT
GCGTCCAACGGCGTCATCCACGGCATCGAGAAGCCCATGGAGCTGCCTGGTGACCTCGAG
AGCCTCACCGCTTCGGTCGATGTTCTCAAGACTCTGTCCACTGTCCTCGCCTTGGCCAAC
ATCACGCTCGACGACGCGAACGCGGCTGGCGTCACCGCCTTCGCCCCCAATGACAACGCC
TTCAAGACGCTCCAGACCACCAAGCCCCTGGTGTACAACTACCTCACCGCCGCCGCCGCC
GGCATCCCGGATCTCAAGTCTGTGCTCAACCTCCACATCGCCACCTCTGTCGTCTACTCC
AACGAGCTCGCCGCCACCCAGAACGTGCCCACCCGCAACGGAGAGCTCACCGTGAGCGTC
AATGGCTCCGTTGTGACCGTCAGCAACGCCGGTAGCTCGGCTACTGTCGTCAGCGCCGAC
AACCTGGCCAGCAACGGTGTCGCTCACGTCATCGATCAGGTGTTGATCCCCAGCGACTTC
ACGTTCAGCCTCCGCAAGGCCCTTCTCGGCCTCAAGCTCAACAAGTTCGAGAGCGCTCTG
GCCAGCTTCAACCTGACCCAGTATCTGGACAACACCACTCCGTTCACTCTCTTCGCGCCC
ACTGACGCCGCTCTCGCCGGCAAGACGGTCACCGCTGACGTCCTCCAGTACCACATCATC
GAGGGCAGCAAGACCACCTTCGTCACCGGGCTCCTGCAGTCCCAGCTCGCTCTCAACTCG
AACAACAACGCCTTCCAGCAGCTCAGCTTCAAGGTTGAGAACACCACCAAGTACGTCGCG
ACCGTGAACAGCGTCGTTCTCCCCGCTCCTCAGGATGCCGGTGCCACCATCGGTGTGATC
TACGTCCTGGACACCGTCCTTGCTCCCCCCAGCAAGAACATCGTGCAGACCGCTGCCAAC
GGCGGCTTCTCCAGCCTCGTCTCCGCTGTCAACTTTGCCGGTGTCGCTGATGTGCTCCAG
GACACCACCGTCTTCGCTCCCGTCAACGCCGCCTTCACCGGACCCGTCGCCGACTATCTT
CTCCTCAACACCACCCAGTCCAACGCTGATCTGGTTAAGGTGCTTCAGTACCACGTGGCT
GCCGGCAAGAACCTCTACTACGCCGATGGTCAGCCCGCGCTCCCCGCCACTGTTGAGACC
CTCAACGGCAACGTCACCGTCACCGTCGTGAACAACACCGTGCTCCTCAACGGTAAGGCC
AAGGTCGTCGAGGCCAACATTCTGGCTTCCAACGGTGTGGTCCACGCCATCGACAGCGTG
CTCATCCCCAGCGACGTTACCTTCGATAACGACAAGCTCGTCAAGGGCTTCAAGGCCACT
GATTTCCTCTCGCGTCTGCAAGAGGCCAACCTCACCAAGGTGCTCACGAGCACCACTGCC
TACACCATCTTCGCCTTCACCGACGCTGCCTACGATAGCGCCCCTAAGTCGCTTACCAGC
AACCCCTCCAAGTGGCCCACCGTGATCCAGACCCACATCTTCAACGGCACCATTGCTTCC
CTCGTCGCTGGCCGCAACTACACCATGTTGTCTGGTGAGGTCCTGCAGGTGGCCAGCTCC
ACCAGCGTTCAGGTTGTGGGCGCCGAGAGCGCCGGTAAGCCCTCCGTCGTGGGCGGCCCC
GTCGCCACCGACAACGGTGTGGTCTATCTTATCGATGGCATCCTCTCCGTCAAGGCTGTG
AACCCCGATGACGATGGTCTCTCCGACACGGCGATCGGCTTCATCGTCATCGGCTGCATC
ATCGGCGTGCTCCTCATCATCGGTGCTGCCGGCGGCGGCTACTGGTACTACAGGAGGAGG
GCCGGCTACGAGCAGATCGGAGACAACTCCTTCTAA
>g1007-400846-401434
ATGAAGTTCCTTCTTGCCATGCTCGTCCTCCTTGCCGCCCTGGTGTTCTGCGCCGCCGCG
TCGGAGCCCAAGATCGCCGCCCCTCAGGACAGCGACTGCAACCTTGAGCTCGGCCTCCAG
TGCGCTGGTGCCCTCGCCCAGTGCTTCCCCCAGTGCAAGGAGTTCAAGTCGAACCCCGAC
GCCTGCTTCTCGTGCCTCGGCGGCAGCGTCGCCACGTGCTGCCCCTGCCTCAAGAAGGCC
TTCGGCCCCAACTTCCCCTGCTAA
>g1007-410536-413510
ATGTCAGGACACGTGCAGGAGGACTACCCACTCGTGGGTGGCTATGGCGAGAACCGCTCG
AAGTGGTTCTACGTGGGTGCCATCGTCTTCGTCTGCTGGAACATCCTACTCTTCTCTCTG
GCTGTGGCTGCCATCGCTAAAGTGAACAACATCAGCGACCCGAAGCAGCCCGAACCAGCA
ATCTTTTCACAGAGCGTCTCAGCTACTGCCAACAACACCGTTCCCGTGATGAACGTGGTG
TCCCTTCTTCCCAACGGCAGCGTCAGGAGCGGCGCCGGTACGACCGTCTTCTTCAAGACC
AACCCGCCGCCGCCCTTCGGCGTGGGGTCAAAGAACGTGCGCGTCGCTCGCTTCGTCGAG
AACACCTCGGACGATCACGTGATCGTGGCGTGGACCAACAACACCGCCTCGTACGTTGTT
CTGGCCACCCTCTCGCTTGACAAGCAGAGCGTCACCGGTTGGTACACGCCTGTGAAGATG
CAGTTCGCGGAATTCTACGACCTGATCACCCTGGCCAACAACCACTTCATCATCGCCGCC
GATAAGGCCGTGTGGGCCGGCACTGCCAACACCAACCCTGGCGCCATGACCATCAGCGAG
CTGGGCAGCGTCAACTACATTGATCAGAATGTTTTTGTCTGGAACGACATCCGCCTGACC
GCTATGGGCCTGAGCAACTTCCTGATGACCTTTAACACCCCGTTCATCGACGTCGATGCC
GGCACCACCTTCGGTGCTATCGTCGGCAGCGTCGTGTGGGACATGGTCAACAAGGTCCCC
AACATCACCTTCGCCACTACAGTCCAGAAATGGGCCGGGAGCTTCCCAGTGCACGACGCC
TTCGCGCTCTCCGATACCACGTTCGTCGTTGCCTACGCCACCAACTCTACGGCGAGGCAG
CTGGCGTGCATGTTCGGCAGCTTCAGCCTCGACAACAAGGCCATCACCTTCACAGACCCC
ACTGTCTTCGAGAGCATCGCGCCCGAGTACCACCTCTCCGCTGTTGGCATCGACGCCAAC
ACTGGCATGATCGTGCTGGTCGACAGCAACAAGGACTTCGCCCTCCGTGCCGTGCTTGTG
CACAGGAGCGACTTCAAGATCAACGGCGTTCACTTCGGTGACGTTCTGACGATCGAGGAC
GGCGTGGCGGACAACATCTACACCAACACGTTCAAGAACGGCATTCTGCCCTTCATCGAC
TCTGCCAAGGTGTCCTCCAACAAGGTGGTCGTCGCGTGGTCGGACTACAGCAACAACGGC
AGGCTCACCACGGCCATCATCACAGTGGACCAGGCGGCCAACCTCCACTCCTCGCCGCTC
TACGTCATCGGCCAGGCCCTCGCGCTTAAGCAGCTTAACGGCTACCACGTCAGCGTCGCC
GGCCTTTCGATCGACGCCTTCGGCCAGTACGGCGCCGTCGTGATCGACCGCAACAACGCC
GCCGACGCCGCCAGCGCGGGCCACGTCGCGCTCGTCGAGGTCGGCCCCAAGGCCTACGGC
GTGGCCGCCCACACCGCCTTCGCCGGCCAGTCGTCCAAGGTGGTCGTCAACGGCCTGCTC
GACCTGCCTTCCTCGTTCCCCACCGACCTCAAGCCCGGCCACCTCTACTTCGGCCGTACC
GACGGTTCGCTCGAGGCCGGCCCGCTGGCCGGCACCATCGACAAGGCCATCCAGTACATC
ACCCTCGACGACGGCAGCCTCGTCACCGTCGACTCGGCCGTCGGCGTCGCCGTCGGCAAG
CGCCAGCTGTACGTTCTCCCTTCGTTCACCCACTAG
>g1007-463237-465718
ATGTTTGCGATGGATGACACGGGCTCGCCCTTGACCGCCCGCTCTGACGCGACTGCAGGC
CCCGAGCTGTGCTTCTCGCCGCCGCGGCAGACCGTGTCGTACCCCAAGAACAAGATCAAG
ATCCTCCTGCTCGAAGGCGTGTCCGACGAGGCTGTGGGCATTCTCAAGCAGGAAGGCTTC
AACGTGGAAGCGCACAGGGGGAAGCTGCCCGATGACGAGCTCGCCGAGAAGATCCGTGAC
GCTCACTGCGTGGGCATCAGGAGCGGCACCAAGCTGACGAAGGAGCTGTTGGCCTTGGGC
CAACGCCTGCTGTGCGTGGGCTGCTTCTGCATCGGTTCCGAGCAAGTGGACCTCCAGTCT
GCCCAGTCGCTTGGCGTGCCCGTGTTCAACTCGCCTTTCTGCAACACTCGTAGCGTTGCC
GAGTTGATTGTGGCCGCCGTCATCAGCTTGGCGCGTCGTCTTGGCGACAAGAACAAGGAA
CTGCACTCGGGCCACTGGAACAAGTCGGCCAAGGGTTGTCACGAAGTCCGTGGCAAGACC
CTGGGCATCGTCGGGTACGGCCACATCGGCTCGCAACTGTCGGTGATGGCGGAGGCCCTG
GGCATGCGCGTGATCTTCCACGACATCGTCCCCAAGCTGCCGCTCGGCAACAGCAGGCAA
GTTGCCGATCTCAACACCCTGCTGGAGCAGTCTCACTTTGTCACCATGCACGTACCGTCT
ACGCCGCAGACCAAGAACATGATCGGCGAGGAGCAGATCAAGCTGATGAGGCGAGGCTCG
TTCCTGCTCAATGCGAGCCGAGGTAGCGTGGTCATCATCCCCGACCTCGTCAAAGCGCTG
AAGAGCGGCCACTTGGCGGGAGCGTACGTGGACGTGTATCCGCATGACATGGAGCCCGGC
AGCGTCTCCGACAACTGGGAATGTGATTTGCGCGGCTGCCCCAACACGCTTCTCACACCG
CACATTGGTGGCTCTACCGAAGAGGCGCAGGCGGCAATCGGCGTAGATGTCGCGAACAAG
ATCATCAACTACATCAACGCCGGTGCTTCGATCGGCGCCGTCAACTTTCCTCAGATCGGC
CTGCCCTACGGCGGCCCACAAACCCACCGAATCCTCAGCATTCATCGCAACAGGCCTGGT
GTGCTTAGGGATATAAACTTGATCCTTGCGGACTTCAACGTGACCCGACAGGTACTCGGA
ACGCAGGGCGACGTCGGTTATCTGGTGGTGGAGGTCGACCGTGCTGCCTCCAAGCCCATC
AAGGAAGCCATCATCAAACTGGAGGCCAGCATCAAGACGCGCATCTTGTTCTGA
>g1028-206767-208058
ATGGGATTCGTCAAGGTGGTCAAGAACAGGTCGTACTTCAAGAGGTACCAGGTCAAGTAC
AGGCGAAGGCGTGAGGGCAAGACGGACTACCAGGCCCGTAACAAGATGATCATCCAGGCC
AAGAACAAGTACAACACGCCCAAGTACCGCCTGGTCGTCCGCGTGACCAACAAGGACATC
ATCTGCCAGATCGCCTACGCCAAGATCAAGGGTGACATGATCATGACCGCTGCCTACTCT
CACGAGCTCCCCCGCTACGGCCTCAAGACTGGTCTGACCAACTACGCCGCTTGCTACGCC
ACCGGCCTCCTGCTCGCCCGCAGGCATCTGACCAAGCTCAACCTGGCCGAGAAGTACCAG
GGTAAGACCGAGGTCACCGGTGAGGACTGGTCGTACAACCTCGACGAGATTGAGGGTCCC
AAGCCCTTCAAGGCCACCCTCGATTCCGGCCTTGCCCGCACCTCGACTGGTGCCAAGCTC
TTCGCCTCGCTCAAGGGCGCCTGCGATGGTGGCCTCGACATCCCCCACTCGGACACCCGC
TTCGCCGGCTACTCCGCTGAGAAGAAGAAGCTCAACGCTGAGGTCTTCAGGAAGTACATC
TTCGGTGGCCACGTTGCCGACTACATGAAGCAGCTCGCCGAGGAGAACCCCGAGAAGTAC
GCCAAGCAGTTCTCGCAGTACATCAAGAACGGCGTCAAGCCCGAGGATGTCAAGACCCTC
TACGAGAACGTCCACAAGGCCATCCGTGCCAACCCCGCCCACCAGAAGAAGGCCGCCCCC
GCTCAGCCCGTCGTCGCCAAGCGCTGGGCTCCCGCCAAGAGGTCGCTCAAGCAGCGCCAG
GCCCGCGTTGCCCAGAAGAAGGCCAGCCACGCCAAGAAGCTCGAGGCCTAA
>g1042-3322-5648
ATGAGATCCTGCCCACAGCGTCGCCTCCTAACTCTTGGCTTCCCCAGCGTTTCGCGCACC
CTTGGCGGTAGAAGCTCAGCAGTGCTGGTGCAGCGCGGCTATGCGTCGTCAGTATGGTCG
CACGTGCCCAAGGGTCCTGAGGACCCCATCCTGGGCGTTACGCTGGCCTTCAACAAGGAC
ACCAGCCCCAAGAAGATGAACCTTGGTGTCGGCGCCTACCGGGACGACAACAACAAGCCC
TACATTCTGCCCACCGTTCGCCAGGCCACTAGCGACCTCCAGAAGCGCCTCGATGAGGGT
AAGGAGGACCATGAGTACCTGGGCATCGTGGGCGACCCCAGCTTCAACAAGGCCGCCATC
AAGCTTGCCCTCGGCGAAAACTCGCAGCACATCATCGACAAAAAGGTGGTGACTGTTCAA
GCGCTCTCCGGCACCGGTGCGCTGAGGATTGCGGGAGACTTCCTGAACCGTTTCTACACG
CATAACAAGCAGATCTTTGTCCCCACCCCGACATGGGCCAACCACATTCCCCTCTTCACC
GACGCCGGGCTCGAGGTCAAGTACTACCGCTACTACGACAAGGCCGCCAACGGTCTCGAC
TGGAAGGGGCTCATCGACGACATCAACAGCGCTCCCAACAAGTCGATCATCCTTCTGCAC
GCGTGCGCTCACAACCCGACGGGTCAGGACCCCAAGCTCGACCAGTGGAAGGAACTGGAG
AAGCACATCAAGACCAAGGAGCATCTCGTGGTCTTTGACTCAGCCTACCAGGGCTTTGCC
AGCGGTGATCCTGAGAAGGATGCTGCGGCTGTCCGCTACTTCGTGGAACAGGGCCACAAC
ATCGTCCTGTGCCAGTCCTTCGCCAAGAACTTTGGTCTCTATGGCCAGCGCATTGGAGCT
GTGTCTTTCCTTACCTCAACTCCCGAGGAAGCTATCAACGTGGAGTCCCAACTGAAGATC
CTCGTCAGGCCCATGTACTCCAACCCCCCGAAGCAGGGCGCTAAGATTGTCTCCGCCATT
CTCAATAACTCTGAGCTCGCCACTGAATGGCGCAAGGAGGTAAAGGGAATGGCCGACCGG
ATCATCACCATGAGGGATCGCCTTGTACACGGCCTTAAGGAAGCTGGCTCGACTAGGGAC
TGGACCCACATCACTGACCAGATTGGCATGTTCTGCTTCTCTGGCCTTTCTCCTGAACAG
GTGGACAGGCTGGCCAACGAATTCCACATTTACATGACCAAGAATGGCCGCATCTCCATG
GCAGGTGTTACCTCCCACAATGTGGACTACCTTGCCAAGGCCATCCATGAGGTCACCAAG
CAGTAA
>g1042-48157-50458
ATGCACGCCCGCACTACCACAACGACCACGACCTGCTCCATGCACAACAATGGCGTGCGG
CGCCCAGTTCAGAAGTACCAGGTGGCCATCATCGGATCCGGGAACTGGGGTAGCGCTATA
GCCAAGGTGGCCGGCGAGAATGTGGCCCAGCAACCCGAACTCTTCGAGCCTCAAGTTAAA
ATGTGGATGCAAAATCGAGACTTGGCGGACATCGTCAACACCCGGCACGAGAACACCAAG
TACCTGCCAGGCATAAAGCTGCCCACCAACGTGATTGCCGTGCCCGACCTGCTGGCGACG
GTGAAAGACGCGCACCTGCTTGTTTTTGTAGTCCCCCACCAGTACGTGGAAGAGACGTGC
AGGAAGCTCCGGGGCCACCTCCGACCCGACGCCAAGGCTGTATCGCTCATCAAAGGCATG
TCCGTGGAGGACGGCGAACCGCAACTCATCTCCGAGCTCATCAATGGCCTGCTTGACATC
GACGTGAGCGTCCTCTGCGGAGCCAACATTGCGAGCGAAGTCGCCAAAGGCGGGTTCAGT
GAGGCCACCATCGGGTACAAGGACAGACAGGCGGGAGAAGTTTGGCGGAAGCTGTTCCAC
AACCCGACCTTCCGAATCAACACGGTGGACGACGTTGCGGGAGTTGAAGTGTGCGGCGCT
CTCAAAAACGTGGTGGCGCTGGGTGCTGGTTTCTGCGACGGCCTGGGATTGTCCTCGAAC
ACGAAGGCAGCTGTGATTCGATGCGGCCTGGCTGAGATGCGAAAGTTTGCCCAGACCTTC
TTCAGCGACATCAACATGCAGACCTTCTTCGAAAGCTGCGGCGTGGCTGATCTGATCGTC
ACCTGCTTCAGCGGGAGGAACCGTAAGGTGTCAGAGGCCTTCGTGGTCACAAAGAAGAGC
TTCGAGGCGCTCGAGAAGGACATCCTCAACGGACAAAAGCTGCAGGGCACCCTCACAGCT
CAAGAAGCCCCACGAGGTCCTGGAGAAGAAGGGACTCACCGACGAGTTCCCGCTGTTCCG
GACCATCTACCAGATCGCGTTCGAAGGCTTGGCGCCCTCCCACATCACCGCCTTCTGAGT
TACCTCAAGTTCCAAAAAGAAAAATCAATAAATGCCGAATGCACAACACTCATCACGCAC
CGTGGAGCAAGGGACGACGCAGAGAACGAAGGGGTTATCGCCCACGACGCATAA
>g1042-50926-52181
ATGTCAACCTGCACACTCCCCGCAAGGACCACTTCATCTTCCGGCGCCACCTACGGACGA
AGTATGAACATGGCCGACGTGAAGGTTGTACACTTCATTCGTCACGGAGAAGCCGAGCAC
AACGTCGCGGCGCGTCGCCACGGCTGCCAGGAGTACCGCAACTGGGCCTATCTCGACGCG
CCCTTGACAGAAAAGGGAAGAGGCCAAGCCAGAGAAGCCCAGAAGGTCGTGCTGGCCCAA
ATGAAGCCGCAGGTGGTGCTGGTGTCGCCGCTCACGCGAACGCTGCAAACAGCCGAGGAA
GTCTTCCAACCGCTGATGGACAGCAGCGAAGGCAAGCCACGGTTCGAGGTGTGCGAGGGC
GTGCGGGAGCGAATCGGTCACCACCCCTGCGACAAGCGACGGACGGTGTCGGAGCTCAAG
CCCCAGTTCCCGCAGTTCTCCTTCGATGCCATCCTCGACGAGGACGATTGCCTCTGGAGC
GAAGCCCGGGAGCCCACCGAGGACATCCTGCAGCGCGCCAAAGCTTTCCTAGAGGTTCTG
CGACAACGCTCAGAGAATTGCATCGGCGTGGTGTCTCACAGCGCGTTCTTGACGGCCATG
TTCGTGGTGCTGACGACGGAATGCGGCCTGCGGAGCGACGGACCCGATGGCCCGCCCGAC
ATCACCTCTGCGTCATCGCCCGATGCCGTGGCCAACGGTGGACCCATGAACGGCGAGTCC
AAGCCCTACTTTGCCAACGGCGAGGTCAAGACCGTTGTCATCCTCCCGCACTCCTCGTGA
>g1042-52213-54138
ATGGCCACCATCACCGCTGTGCACGCTAGGCAGATCTTCGATAGCCGCGGCAACCCCACC
GTTGAGGTCGACGTTACCACCGAGAAGGGCCTGTTCCGCGCTGCCGTGCCCTCTGGTGCC
TCCACTGGCATCTACGAGGCCATCGAGCTCAGGGATGGCGACAAGTCCAAGTGGCTCGGC
AAGGGGGTCACCAAGGCCGTCTCGAACGTCAACGAGGTGATCGCCCCCAAGATCATCGGC
AAGGACGTCACCAAGCAGGAGGAGCTCGACAACCTGCTGCTCGAGATCGACGGCACCGAG
AACAAGGGCAACCTCGGAGCCAACGCCATCCTCGGCGTGTCGATGGCCGTGTGCAAGGCC
GGCGCTGCCGTTAAGGGCGTGCCTCTGTACCGCCACATCGCCGACCTGGCCGGCAAGAAG
GAGGCCGTCACGCTGCCCGTGCCCGCCTTCAACATCATCAACGGCGGCTCGCACGCCGGC
AACAAGCTGGCCATGCAGGAGTTCATGATCCTCCCCGTCGGCGCCTCGTCCTTCACCGAG
GCCATGCGCATGGGCGTCGAGGTGTACCACAACCTCAAGAACGTCATCAAGGAGAAGTAC
GGCCAGGACGCCACCAACGTCGGCGACGAGGGCGGCTTCGCCCCCAACATTCAGGGTAAC
GACGAGGGTCTCGAGCTCTGCAAGGTCGCCATTGAGAGGGCTGGCTACACCGGCAAGGTC
AAGCTCGGCATGGACGTGGCCGCCTCTGAGTTCTACAAGGAGGGCAAGTACGACCTCGAC
TTCAAGAACCCCTCCAGCGACGCCAGCCAGCACAAGACCGGCGCTGAGCTCGCCGAGCTG
TACGCCTCGTTCACGCGCAACTACCCCGTCATCTCCATCGAGGATCCCTTCGACCAGGAC
GATTGGGAGTCCTACACCACGCTCACCGAGGCTCTCGGCAAGGATGTGCAAATCGTGGGT
GACGATCTCCTGGTGACGAACCCCAAGCGCATTGAGACCGCTCTGGAGAAGAAGGCCTGC
AACGCGCTTCTCCTCAAGGTCAACCAGATCGGTTCCGTCACGGAGGCCATCAAGGCGTGC
CTGGTCTCGCAGGCCGCCGGTTGGGGCGTGATGGTGTCGCACAGGAGCGGTGAGACCGAG
GACACCTTCATCGCTGACCTCGTGGTCGGCCTCGGCACCGGCCAGATCAAGACCGGCGCC
CCGTGCAGGTCCGAGAGGCTCGCCAAGTACAACCAGCTCCTCAGGATCGAGGAGGAGCTC
GGCGCCTCCGCCAAGTACGCCGGCGAGGGCTTCAGGAACCCCCAGTAG
>g1042-121486-122422
ATGTCTGAGACTGAGAGCAAGGCCACCACCAAGTCCGCCAAGGCCACCAAGGCCGACGCC
GGCGAGACCAAGAAGAGGAAGGCCAAGACTGTTGCCCCCACCGCGGCCTCATCCCGCCCC
GAGCGAACGCACAGGAAGCCCAACCGTTTCGAGTTCGGCGCTGAGGAGAAGAGCGTTGTC
ACCTCGAAGAGGAAGAGGGCCGCCTCCAAGGAGCGTGCGCCCGCCAAGAAGAGGAGGTCC
GTGAGCAAGTCGCCGTCGAGGAGCAAGAAGTCGACCAAGAAGGGCGGCAAGAAGAAGAAG
GATCCCAACGCCCCCAAGAAGGCCCTCAGCGCCTACATGTTCTTCGCCCAGGCCAACCGC
GACAAGGTCAAGAAGGAGAACCCCGACGCCACCTTCGGCGAGCTGGGCAAGCTCCTCGGC
AAGCAGTGGAGCAAGGCCAGCAAGTCGGACAAGGCTAAGTACGAGGCCAAGGCCAACAAG
GACAAGGAGAGGTACGAGAAGGAGAAGGCCAAGTACGACAAGAAGCGTGAATCCTCAAGC
GAAGAGGAGGAAGAGTCCTCCGATTAA
>g1042-138094-141058
ATGTCGAGGGACCCCAACGTTATTCGTATCAAGCCCTACCACTACATTCACGTTCTGGAC
AACAACACGAACGTGACTCGCGTGGAGGTGGGTCCTAACACATACACCAGGCAGGAACAT
GAGAAGATCGTGTTCGGCCCTGAGTCGATGATCATGATCCCCCCTCGCCACTACTGCATC
ATTTCGAACCCGGCGACCAAGGATGCCAAGAACCAGCCCGTCTTCGATGGCCACCACAAC
GTCAAGGTGCGCTTCGGCGATGAGGAGATTCGCTTCGAGCAGGAGCCCTTCCCGCTCTAC
CCGGGCGAGTCGGTGCTCGGCAAGGTGAGCGCGCTCCAGGTGGTGGGTCCCGATGCCGCG
CTTCGCCTGCGCGCGATCCGCGATTTCGAGGACAAGGTCGCCGGCGACGAGTGGCTCTTC
CGTGGCCCGGCCACCTACAAGCCGCGCGTCGAGGTGCAGGTCGTCGAGATCATCCGCTCG
ACCATCGTCAAGCCCAACACGGCGCTCAAGCTGCGCGCGCGCAAGGCCGCCGTCGACTCG
GCCGGCACCAAGCGTGAGGCCGGCGAGGAGTGGCTCGTGCGCACCGTGGGCGCCTACCTG
CCCGGCGTCGACGAGGAGATCGTCCAGACCGTGCCCGCCAAAGTGCTGACCGACAAGAAG
GCCCTCCACCTGCGTGCCACCAAGACCTTCGTCGACGTGTTCGGCACCAAGCGCAAGGCC
GGCGAGGAGTGGCTCGTGACGCTCGACGACGCCGAGACCCACATCGCCGACGTCTACGAG
GAGCTCGTGGGCGAGGTGCGCATCACCACGCTGTCCAACCGTCAGTACACGGTCGTGCTC
GACCCGCTCGACAAGAACGGCCACCCGCAGCTGGGCCTCAAGCACCTGCGCGTCGGCCCG
GCCTCGTTCTTCCTCCGCCCCGGAGAGAGGCTCGAGAACGGCATCCAGAACATCCACGTG
CTCGGCGCCGAGGAGGCCCTCCTCCTGCGCTCGCGCGAGGCCTACCAGGACGGTGCCCTG
GCGCGCAAGCCCGGCGACCGCTGGATGATCGTCGGCCCGTGCGACTACGTGCCGCCGATC
GAGGTGGACGTGCTCGAGACCCGCCGCTCGATCCCGCTCGACGAGAACGAGGGCATCTAC
GTGCGCGACCTCAACACCGGTAAGGTGCGCGCCGTCATCGGCGAGTCCTACATGCTCAAG
GCCGACGAGGAGCTCTGGGAGAAGCAGCTGCCCCAGGTCGTCGAGGACCTGCTCTCGCGC
GAGCGCACCTCGGGCGAGGTCCGCTCGGCCACCTGGTCCAACGCCCGCGGTGGCGTCCAG
CCCGCCGTGTCGGCCCGCGACAAGACCCGTCTCGTCACCTACCGCGCGCCCCACGGTGCC
GCCGTGCAGATTTATGACTACAAGGAGAAGAAGGCCCGCATCGTGTTCGGCCCCGAGCTC
GTCACGCTCGGCCCCGAGGAGCAGTTCACCGTGGCCGACCTGTCGGGCGACGTGCCCAAG
CGCCCCAACGTGATCAAGTCGCTCGCCCTCCTGCTCGGCCCCGACTTCATGACCGACATC
GTCATCGTCGAGACCTCGGACCACGCGCGTCTCTCGCTCAAGCTCTCCTACAACTGGTTC
TTCGAGGTCGACAAGGCCAACCCGCAGTCAGCCGCCTCGATCTTCCGCGTGCCCGACTTT
GTCGGCGATGCCTGCAAGGCTATCGCCTCGCGCGTGCGTGGCGCCGTCGCCGCCGTCTCC
TTCGACAAGTTCCACAAGATGTCGTCGGACGTCATCCGCGCCGCCGTGTTCGGTATCGAC
CCCGAGACCGAGCAGGTCAGGAACAGGTTCTTCTTCTCGGCCAACAACCTGGTGATCACC
AACATCGATATCCAGTCGGTCGAGCCCGTCGACCAGCGCACCCGCGACGCCCTCCAGAAG
TCGGTCCAGCTCGCCATTGAGATCACCACCAACTCGCAGGAGGCCTCGGCCCGCCACGAC
GCCGAGCGCATGGAGCAGGAGGCCCGTGGCCGTCTCGAGCGTCAGAAGATCCTCGATGAG
TCCGAGGCCGAGAAGGCCCGCAAGGACCTCCTCCAGCTGCAGGCCCAGAGCGCCGCCGTC
GAGGCCACCGGTCAGGCCACCGCCGAGGCCAAGGCCCGCGCCGAGGCCGCCCAGATCGAG
GGCGAGGCCGCCGTCACGCAGTCGCAGCTCAAGGCCGAGGCCACCAAGATCAAGGCCTTT
GCCGACCTCGAGCAGCTCAAGAAGAAGCAGTTCCAGGAGATCGACCACCGCAAGGCCATG
GCCGAGCTCGAGGTCAACAAGGCCAGAGAGCTGGCTGCCATCGAGTCCAAGAAGTTCTCG
GAGATCGTCGACGCCATCGGTGCCGAGACCATCAAGAAGATCGCCCAGGCCGGCCCCGAG
ATGCAGGCCAAGCTGCTCCAGGGCCTGGGCCTCAAGAGCCTCATGATCACCGACGGCTCG
TCGCCCATCAACCTCTTCAACACCGCCTCTGGCCTCATCGCCCAGCACCAGTAA
>g1042-144136-144843
ATGGCCGACAAGCAGCAAGCGACGACACCCGCCAAGGAGCACGTTATCGAGGAGGACGAT
GAATTTGAGGAGTTCGAGAATGAGGAATGGAAGGAGGATCAGGAAGATGCTGACGATGCC
AAGCAGTGGGAGGACGATTGGGATGATGAGGAGGTAGATGACGATTTTTCTAAGCAGTTG
CGGGCTGAGCTTGAGAAGCAGACCACGGCGATGCAGACGTCGTAG
>g1067-36876-38664
ATGGGTAAGGAGAAGACTCACATCAACCTTGTCGTGATCGGTCACGTCGATGCCGGTAAG
TCGACCACCACTGGCCACTTGATCTACAAGTGCGGTGGTATCGACAAGCGAACCATCGAG
AAGTTCGAGGCTGAGGCCAAGGAGATGGGCAAGGGCTCGTTCAAGTACGCCTGGGTGCTC
GACAAGCTGAAGGCCGAGCGTGAGCGTGGTATCACCATCGATATCGCTCTCTGGAAGTTC
GAGACTGCCAAGTACTACTTCACCATCATCGATGCCCCCGGTCACCGTGATTTCATCAAG
AACATGATCACTGGTACCTCGCAGGCTGATGTGGCTATTCTCGTCATCGCCTCCGGTGAG
GGTGAGTTCGAGGCCGGTATCTCGAAGAACGGTCAGACCCGTGAGCACGCCCTGCTCGCC
TTCACCCTCGGTGTCAAGCAGATGATCGTGGTCTGCAACAAGATGGACAACGTCAACTGG
GCCGAGAACCGTTACAACGAGATCCAGCGTGAGGTGTCGGGCTACCTGAAGAAGGTCGGC
TACAACCCCAAGAACATCCCGTTCGTCCCGATCTCCGGCTTCCACGGTGACAACATGGTG
GACCGCACCGACAAGATGCCGTGGTACAAGGGCCCCACGCTCCTCGAGGCCCTCGACGAC
ATCAAGCCCCCCAAGCGCCCCATGGACAAGCCCCTCCGCGTGCCCCTCCAGGACGTCTAC
AAGATCGGCGGTATCGGTACCGTGCCCGTCGGCCGTGTCGAGACTGGCGTGCTCAAGCCC
GGCATGGTGGTGACCTTCGCCCCCGTGAACGTGACCACTGAGGTCAAGTCCGTCGAGATG
CACCACGAGGCCCTCCCCGAGGCCGTGCCCGGTGACAACGTCGGCTTCAACGTCAAGAAC
GTCTCCATCAAGGACATCCGTCGCGGTAACGTCGCCGGTGACTCCAAGAAGGACCCGCCC
CAGGAGACTGAGGACTTCACCGCCCAGGTCATCATCCTCAACCACCCTGGTCAGATCCAC
GCTGGCTATGCCCCCGTGCTCGATTGCCACACCGCCCACATTGCCTGCAAGTTCAAGGAG
CTCCTCGAGAAGGTCGACCGTCGTTCGGGTAAGAAGATGGAGGACAACCCCAAGGCCGTC
AAGTCTGGAGACGCCGCCATGGTGCTCCTCATCCCCTCGAAGCCCCTCTGCGTCGAGACC
TTCACCGACTACCCGCCCCTCGGCCGCTTCGCCGTCCGTGACATGAGGCAGACCGTCGCC
GTCGGCGTCATCAAGGCCGTCACCCGCAAGGACCCCAAGGCCGGTAAGGTTACCGCCTCG
GCCAAGAAGGCCGGCAAGAAGTAA
>g1067-68166-69042
ATGTCGGAAGAGGCTGAAGTTGCGCTTTTCGATCCTACGCAAAAGAAGAAGAAGAGGACG
AGGAAGGTCGTCGCGGACGCCGCCACCGAGGAGGTAAAGGCTACCGATGCCGCCCCCGAT
CAGGCCACATCCGACGAGGTCGCCCCTTCCACATCCACCAATGGCGAGGATGCCTCTGCG
GCTGCCAAGGCCGACATCGAGGACTACTCGTACGAGACTCTGCTCCATCGCGTGTTCGAG
CTCGTTGGCGATAAGCCCGATCAGAAGCGATCCAAGCGCGTGCCGCTGCCCGAGGCCTAC
CTCGTGGGTACCAGCAAGACCCTGTGGTCCAACTTCCCGGCGATCGTGAAGGCCCTCAAT
CGCAACCCCCAGCATCTCCTGACCTACGTCATCGTCGAGTTGGGAACGACGGCCAATTTG
GACGGCAGCGGCCGCGTCGTTATCAAGGGTCGCTTCATGCCCAAGCAGCTCGAGTCGCTG
CTCAAGAAGTACATCGACGAGTACGTCGTGTGCAAGACCTGCAAGGGACGAGACACCGTG
CTCAAGAGGGAGAACCGTCTCCACTTCCTTGTCTGCCAGTCTACCGTCTGCGGATCGTCT
CGCTCCGTGCCGCCCCTCAACAAGGGTTACGTCCACAACATCCGCCGTAAGAAGGGCAAC
TAA
>g1067-70146-70730
ATGTCGGCTGCCAGAGGAGTGCGCACGATTATCAGGCCGGTGAACAAGGGCACCTACTTC
GGCCGGTTCAACCAGTTCTGGAAGGAGGTCACTCTTGAGGACCCTGCCATCTCCATCTCC
TTCTTCCTTGGTGGCCTTGGCGTGGCCATTACCTTCCTCTCCATGCCTAATGTCCGCGGC
ATTCTCGTCCCCTTCAAGTCACCCAAGCAATCCTGGTTTGGCGAGCACTACTAG
>g1067-115527-116868
ATGTCCGACTCGCTCACTCTCAAGGGTGTCCTCCGGGGCCACAGCGGCTGGGTTAACTCC
ATTGCCACCACCACCGAGAACAATGACATGATCCTGTCCGCCTCCAGGGACAAGACCCTG
CTCGTGTGGAACCTCACCCGCGATGAGGACAACTACGGTCAGCCCAAGAGGGCCCTCAAG
GGTCACTCCCACTTCGTCAATGACGTCGTCATCTCGTCGGACGGCCAGTTCGCCCTCTCC
GCTTCGTGGGATAACACCCTCCGCCTGTGGGACCTGAACGCCGGCACCACCACCCGCCGC
TTCGTGGGCCACAGCAAGGACGTCCTGTCCGTCGCCTTCTCGGCCGACAACAGGCAGATC
GTGTCGGGCTCGCGCGACAGGAGCATCAAGCTGTGGAACACCCTGGGTGAGTGCAAGTAC
ACCATCGATGGTGAGGGTGCGCACACCGAGTGGGTCAGCTGCGTGAGGTTCTCGCCCAAC
GCTGCCAACCCCGTCATCGTGTCGTCCGGCTGGGACAAGCTGGTCAAGGTGTGGAACCTC
CACAACTGCCGCCTGAGGACCAACCTCCGCGGCCACACCGGCTACGTCAACACCGTGACC
GTCTCCCCCGACGGCTCGCTCTGCGCCTCTGGCGGCAAGGACGGCACTGCCATGCTCTGG
GACCTGAACGAGGGCAAGCACCTGTCCTCGCTCGAGGGTGGTGAGATCATCCACTCGCTC
GTCTTCTCGCCCAACCGCTACTGGCTCTGCGCTGCCACCACCACTGGCATCCGCATCTGG
GATCTCGAGAGCAAGAGCGTCGTCGCCGAGCTCAACAAGAACAACTGCGCCGGCTTCTTC
GGTGATGACGACACCCCCAAGGACAAGAACCCCACCCCCATCTCCCTTGCCTGGTCCGCC
GATGGCTCCACCCTCTTCGCCGGCTACACCGACAGCGTCATCCGTGTCTTCGAGACCTCG
CTGTAA
>g1067-125456-128230
ATGGGACGGAACAAGATCAAGATCGAAAGGATCACCAATGAGAGGACTCGCTTGGCCACG
TTCAACAAGCGCAAGAATGGTCTGGTAAAGAAGGCCATGGAGCTCGCCATCTTGTGCGGC
TGCGAAGTCGCCCTGATCGTCATCGGTAACAACAAGCTGACGCAGTACAGCAGCAGCGAC
ATGGATCAGCTGCTCCTGCGCTACACCGACGGCGGCTTCGAAGAACCCCACGCCGAGCCC
CTCACCAGCGACGACTACCCCAAGCTGTTCGGCAAGAAGAACAAGGCCCTCGTGACCGGC
ACGTGCGCCAGCAGCAAGGAGTCGCCCGCCAAGAAGGCCCGCGTTGAGAGCCCCGCCCCC
CTCCAGGCCGCCTCCGCCGCCGCCCCCTCTCACCCCGTCGTCGCGGTCAAGCCCCTCGCC
CCCGCCGCGCACCACGACGGCCGCTCTAGCCCCTCGGCCGCCCCCGTGCCGACGCTCGTG
CCCCGCGGACCTTCGCCTCCTCTCGGTGTCAAGCCCGTGTCGCCCACCGCCGGCTTCGCC
CGCCTCTCTGCGCCCCAGCAGCAGTTGGCCGCGAGCTACCCCGCGCCCTACATGGTGGGT
GGCGGCTACCCGGCCACGCGCACCGGCCAGTTCCCGTCGTCCCAGCAGCAGTCCTCCTCG
TCCCCGTCCCGTAGCAACATCATCACCCCCTCCCTCTACTCTGCCCCGAGTACCTCCGCC
AACAACGTCAACGCCAACAGCAGCACCCCCGGTGGCCGCATCTCGCCTCCGAGCCACCGT
GGTAGCTTCGCCGCGCCCGGCTCTGTGTCGCCCACGTTCGAGGGCGCCAAGCCCCAGTCG
CCCCACTCCAACCCGCCGACTCCCCCCGGCAGCGGCGGCCAGTACGCCGAGGAGATGCGC
AGGGCCTCGTCGGCCGGCCGGCTCCACCAGGAGCAGCAGTTCGCGCAGAAGCTCAACAAG
AAGAACCTCTCGCTGGCCATCCCCGACGCCTCGTCGGTCTCCTCGGCCAGCTTCCCCGGC
TCCTCCTCGTCGGGCCACTCGCGCCACACCACCTACACGCCGGGCTTCACCGGCCTCAAC
GTGCCCTCGAGCATGCCCTTTGGAGCCGAGCACGACCACATGTCGCAGCACTCGGCCGTC
CTGCCGTCGCCCTCGACCTTCTTCGCCGGCGAGACCCCCGAGCCTGCCAGCTACGCCATG
CCCGCGTGGGGCAGCTGGATGGCCTCGTCCTCGCCCAGGCACCTCCATGACTCACTGCCC
CAGGAGCGCTTCCTCTCGCGTGACCTCAACATGGCCCTCCACGACGGCACCCTCCACCAG
CAGCTCCAGCTCGGCAAGCAGCTCCCCTACACGGTCGGCGACAAGCGCAAGGCCCAGGAC
CTCCTCAAGGTTCTCTGA
>g1107-5327-8677
ATGTGTTGCATTCTTGTAGGGGGGGATACTAGGCCGTCACCAGTCTCCTTCGCCAGTCTC
CTTCGCCACCCATCGCCGCCAGTCGCCGCCCCGCACTTCGCCGCCGGTCGCCGCCCCCAG
TCCACCGCCGCCCCGCACTTCGCCGCCGGTCCACCGCTGCCAGTGGCCCCTGTCCTTCAC
TGTTGGTCGCCGCCAGTCGCCGCTCCCAACCTTCGCCGCCGCTCGCCGCTTCGCCGCCAG
TCGCCACCAGTCGCCACCCCAGCCTTTGCCGCCGGTCGCCGCCGCCGGTCCACCGCTGCC
AGTGGCCCCGTCCTTCGCCGTTGGTCGCCGCCAGTTGCCGCCCCCCGCCCTTCGCTGCCA
GTCGCCGCCCCCCGCCCTTCGTCGCCAGTCGCCGCCCCAGCCTTCACCGTTGGTCACCGC
CAGTTGCTGCCCCCCGCCCTTCGCTGCCAGTCGCCGCCCCCCGCCCTTCGTCGCCAGTTG
CCGCCCCCCGCCCTTCGTCGCCAGTTGCTGCCCCAGCCTTCGCCGTTGGTCGCCGCCAGT
CGCCGCCCTTCGTCGCCAGTCGCCGCCCCAGCCTTCGCCGTTGGTCGCCGCCAGTCGCCG
CCCTTCGTCGCCAGTCGCCGCCAGTCGCCGCCAGTCGCCGCCCTTCGTCGCCAGTCCTTT
ATTGCGCGAGTGCCGCGTGTGCTGTGGGTGAGTGTGTTCGTGTGCCTCATGGAGGAGAGC
GAGTTCGGGAAGCACGGGAAGCACTACTTTGACGTCGCCAAGTCCATGCTCGACTCCCGA
CCCTCCGACTTTGTGCAAAAAATCGCAAGAGCTGCGATACGTGCACCACCCGATACCCGA
TCGCATGGTCGGCTCCGACGATGCCACGCTGGTCCTTGTCGAAAGGATCCTCGCCTTCCT
CCGGGCAGGCGAGGTCGTGTACATTCACTGTTATGGCTGCCACGGGCGCACGGGCATCTT
CGCGTCGGTGCTGCTTGGCCGGATACACGGCATCGACGCCGAGAAGGCTCTGCAGCTGTG
CAAGCTCTACCACGATTGCCGGGCCCGACGTGGAGGGCATCAGCGCCAAGTCCGTGCCCT
CGCCCCAGACCCACGACCAACGCGCCCAAGTGGTCCGCCTTCTTCGCTGAGCGCTGCGAA
AGAAGAATTGTTTATTTATTTCTTTTTTCAAGCCTGGGGCGTGGAGGGTCGCCGCCAGTC
GCCGCCCTTTGTCGCCAGTCGCCGCCAGTCGCCGCCAGTCGCCGCCCTTCGCCGCCAGTC
GCCGCCAGTGGCCCCGTCCTTCGCCGTTGGTCGCCGCCAGTGGCCCCGTCCTTCGCCGTT
GGTCGCCGCCAGTCGCCGCCCTTCGCCGTTGGTCGCCGCCAGTGGCCCCGTCCTTCGCCG
TTGGTCGCCGCCAGTTGCCGTCCTTCGCCGTTGGTCGCCGCCAGTCGCCGCCCTTCGCCG
CCAGTCGCCGCCCCAGCCTTCGCCGTTGGTCGCCGCCAGTCACCGCCCCTGGTCTCCTCT
GCCAGTCGCCGGTCGCCTTCTCCTCCGCCTCAGTCTTGGGCTGTGGCTGCCTCGGTGCTC
ACTCCGCGACATGTGTTGAGGAGCGAACGAATGCAGCAAGACGGGGAGACAAGTCGAGAG
CGGACTGCGCCCAGGATGGGTTTGCCCTCTTCTTTTTCCTGCGGCTCTCGCCCCATCTTG
CGTGTCATGGAGTTGTTGTCGAATGTGGCCCTCGAGTTGTTGCTTTGCAGCTGTGACCAG
GCAGTGGCGAGCCTTGTTGGTGCTGCGATTGTACCGGGCCTTGTTCTCGGCATGTTTCGC
CTCGAGTGCAAAGGTCGCGAACTTCTCGATGCCGTGCCAGGCCTCCTCAGTGATGCTGAC
GTCCGCCTGCATCAGGAGCGTGGCCAGTTCGGCAAAGTGATCCCACAATGCCGCGATCGA
CCGGTTCCGGCCTTCCTGCTCCGGCAGGTGGCGTTCGAGGATCTCGATGAAGCAGGCTCG
ATGACGGAGGATCGCCAGAGCCTGAGGCCATGTCGGACCTGCGTTCTCCCACCGCTGGAA
CAAGTGCATCGAGTCCGGGATTATCTGTTGGATTGGGAGCTTGAGAAGGCGCCGGCCAAG
GACTCCACCAAGCCGGCGAGCATGCTTCTTCCGCGTATCGATGGTGCAGTGTGCCCGCTG
CATTGCCTGAAGCAGCACTTGACGTTCGCCTTTGGTCGATACACCTCTGTTAGGTTCAGC
ACAGCCGCGAGGCAGGCCATGTCAACACACAGAAGTGGCAGCACATGCTGTTCCACCTGC
TTGAAGGACTGGCCAAACTCTTCTTGA
>g1107-9036-10060
ATGCCACAACTCACATTGACAACACAAGTGGTCGGGCACTTCACCAGCGAGGTACTGTTG
ATAGAGCTCCTCATGGCGTTGCTTGGCTTTGCGTCTGGTGGCCTCATCCTCAAGGCGTGG
GTGCACCCAGTGGGGCACTCGTGGCGGACATCTTTCTCATGCTCGTCTCGATTTCCGTTG
CTTGCGAACTGTCGTGGGCAGTTGGAGTGGCGACATGGCGTGGTGGTAGGTGGTGCGCTC
GTGCTCATCCCGTGCTGGTGCTGTCTTGTACCTGTGGCGCTGGCCGTTCACCAGGTGTGT
GCAGCACTGGTGTCGACAGGGGAAAGTCATGTAGGTGTGTTGGGCGCTCATGGATGA
>g1185-95706-98155
ATGAAGACCACACAACGCTCGCAGCTGCGCCTCGCCGCCAGGGCGCCCCAGGGACGTGTC
TCCGGTCTCCGCTCTGCGTCGGTACTGCGCCGTAGCGTGCACTCGAAGGCCGCCCATGGG
TCGTCGCCGTCGGTGGTGCAGCACGACGTGGTGGTGGTTGGCGGCGGTGCCGCGGGTCTG
TCGGTGAGCCACCAGCTGCGGCGCGCCATGAAGGGCCTCGACGTGGCCATCGTGGAGCCC
TCCTCCAAGCACTACTACCAGCCCGGCTGGACCATGGTCGGGGGCGGCGTCATCAACAAG
CGCGAGACCGAACGGAACGAGGCTGACCTCATTCCGGCGGGCGCCAAGTGGATCAAGTCG
GCTGTGGATCAGTTCGTGCCGGAGGAGAACGCGGTGGTGACCAGCGACGGCCAGACCGTG
CACTACAAGTACCTCGTCGTGTGCCCCGGCATCCAGATCAACTGGGACAACGTCAAGGGC
CTGCGGGATGCGCTGGGCAAGAAGGGGGTGTCGTCCAACTACAGCTACGAGTACGCCGAC
AAGACCTGGGACCATATCAAGCATTTCAACGGGGGTAACGCGATCTTCACCCATCCGGCC
ACGCCCATTAAGTGCGGTGGCGCGCCTCAGAAGATCATGTACCTGGCCGAGGAGAACTGG
AGAAAGCGCGGCGTCCACGCGGACGTCTCCTTCTACACCGGCGGACCCGCCATCTTCGCC
TCCCCCTACTACGGCAAAGTGCTCACCAGGATCTGCCAAGAGCGAGGCATCCACACCCAC
TTCAAGCACGACCTGGTCGAGGTGAGGGGTGCCAACCAGGAGGCCGTCTTCAAGGACCTC
AACACGCAGGAGCTGGTGACGGTGCCATACGACTTCATGCACGTGACGCCGCCCATGGGC
CCGCCCAGCTTCGTGAAGAGCAGCCCCCTTGCCGACGCCGGTGGGTGGGTTGATGTCGAT
AAGCACACACTCCGACACAAGAAGTACGCGAATGTGTTCGGTCTGGGCGACGCGTCGAAC
CTGCCCACGTCCAAGACTGCGGCCGCCGTGAGCTCGCAGGCGGCGGTGGCCACGGCCAAC
CTGCTGGCCACCATCCGCGACCCCAACGCGGCCCTCCCCGAGAGCTACACCGGCTACACC
TCGTGCCCCCTCGTCACCGGCTACGGCAAGCTCGTCCTCGCCGAGTTCGACTACTCTCTC
CAGCCCTGCGAAACGTTCCCGGTGGACCAGTCAAAGGAGCGGCGGTCGATGTACCACCTA
AAGAAGGACGTGATGCCGCCCATGTACTGGCACGGCCTCATCAAGGGCCACTGGAAGGGT
CCCCGCCACCACGCCACCACCCGCCTCCTCAAGGACCTGGTCAACAAGCCCCAGCAACAG
CAGCAGTCGTGA
>g1185-98157-99558
ATGGCGAGGAAGAAGAGAGTGCTTACGGTCAATGAAAAGAGCTTCGGCATTTACATCTAC
AAGGTGCTGAAGCAGGTGCACCCCGACACGGGCATCTCCCAAAAGGCCATGGCCATCATG
AACTCGTTCGTTCATGACATTTCGAATCGTATTCAAGCGGAAGCTGCGGCCCTTGCTGAA
ATCGGCGCCAAGAAATCCCTCACGAGCAGAGAGATCCAGACGGCCGTGCGCCTTCACCTT
CCCGGTGAACTGGCCAAGCATGCCGTGTCGGAGGGCACTAAGGCCGTCACCAAGTTCAAC
TGTTTTGAAGGCGGCAGGCAAAGCAAGTCCGCCCGTGCCGGGCTGCAGTACCCGGTGGGC
TTCTTTCACCGCTACCTCAAGGAGAAGACCAAGCTCAGGGTCGGCAAGGGCGCCCCGGTC
TACCTCGCTGCTGTTATGGAGTATCTCTCCGCCGAAGTTCTAGAGCTGGCGGGTAACGCA
GCGCGTGACAACAAGAAGGCACGCATCATCCCGAGACACCTCATGCTGGCCATCCGCAAC
GACGAGGAGCTCAACAAGCTGTTGAAGAACGTCGTCATCGCCAGTGGTGGAGTGCTACCC
AACATCCATTCGGTCCTTGTTCCGAGGGCCGCGGTGACCAAGCTCCCGGCGTTTGGCGGT
GACGACGAGGGCCTCAACCAGGAGTACTGA
>g1185-112487-113838
ATGTCGCACATCAAGGTCGGTATCAACGGTTTCGGCAGGATCGGTCGTCTCGTGTTCCGC
GCGGCCGTGGAGAGCGGTAAGGTTGACGTGGTGGCGATCAACGAGCCCTTCATGAGCGTC
GACTACATGGTGTACAACCTCAAGTACGATTCCGTGCACGGTGCCTTCAAGGGCGACGCC
CACGAGAAGGACAAGAACACCCTCGTCGTCAACGGCAAGGAGATCAAGGTCTTCGCCGAG
AGGGACCCCGCCAACATCCCGTGGGGCTCTGCTGGCGCCGACTACATCGTCGAGTCGAGC
GGTGTCTTCACTGATGCCGACAAGGCTGGCCTTCACTTGAAGGGTGGCGCCAAGAAGGTC
ATCATCTCGGCCCCCTCGAACAATGCTCCCACCTTCGTGATGGGCTGCAACGAGTCGAGC
TACAAGCCTGAGTACAACGTCATCTCGAACGCCTCGTGCACCACCAACTGCCTGGCGCCC
CTCGCCAAGGTCATTCACGACAACTTCGGCATTGAGGAGGCCCTCATGACCACCATCCAC
GCCATCACGGCCACCCAGAAGACCGTCGATGCCCCCGCCGCCAAGGACTGGAGGAGCGGT
CGCGCTGGTCTCACCAACATCATCCCCGCCACGACTGGCGCCGCCAAGGCCGTCGGCCTC
GTGCTGCCCGAGCTCAACGGCAAGCTCACTGGCATGGCCTTCCGCGTGCCCACCATCGAC
GTCTCCGTCGTCGATCTCACTGCCCGCCTGAAGAAGGAGGCCTCGTACGACGAGATTAAG
GCCGCCATCAGGGCTGCCGCCGACGGCCCCCTCAAGGGAATCCTCGCCTACACTGAGGAC
GAGGTTGTGTCCTCCGACTTCATCCACTCCTCTGTCTCCTCGACCTTCGACGCCAAGGCC
GGTATTGCCCTCAGCAAGACCTTCGTCAAGCTCATCGCGTGGTACGACAACGAGTGGGGC
TACTCCAACCGCGTCCTCGACCTCATCGTCTACATCAACGGCAAGCAGTAA
>g1185-190706-192730
ATGGACCTCACCACCAGCAATTTGGCGAATGTTGTGCGAATCCCACAGCGTGAGGAGCAG
TACCTGAAGGCCACAGGCATCGATCAGCTGCTTTCCAATCTCGTGTCGCAGCTGTACATA
CACAAGCCCGAGGATGCCATCAACTTCATGATCAACTACCTTCAGCACACCAAGCTCGAG
CGAAACTCGGTTTCTTTCTCTGGTGCTTCGTCATCTTCTTCATCTTTCTCCTCCTCCTTC
TCTTCTTCGACTTCATCGCCTGTCGGCAGCTCGTCCACCGTTACCACTCTGCAAGTGAAT
GGCAGCCCACAGTCAGCCGGTGGTGAAGACGAGGGTAATGGTGCCGTGGAAATGGAGCAA
GACAAGAAGGGCAACGGCAATCACCTCGATCAGAGCGGCGAGTATGGTCGCGAGGACATG
CAGGTCACCTCGCCCGACGGCGGCCCGGTCGACATCGATCCTACCCTCAGCGGCAACCGC
TCCCTGCAGCGCCGAAGGATGGCCATCTCTTCCGAGCCTGTGGACCTGAGCGGCTTCGAC
GCAGATATGCCAGGCAATGGCGTGCCCAACACGCCCAAGAGCCCCGAAACTCTGCAAGCT
CTCGAAGAAGCGCTGCGCACCAACGTTCTCTTCGCCCATCTCGAGGAGGACGAGCGTAGG
CAGGTGTTCGATGCCATGGTGGAGGTCAAATTCAACGCCAACGACATCATCATCCAGCAG
GGCGACGAGGGCGACAACTTCTACGTCGTCGAGTCGGGCGAATGTGAGATCTGGATCGCC
AAGGAGGGCCAGCCCCCTCAGCGGGTCAGCGTCGTGAGGGAGGGCGGCAGCTTCGGCGAG
CTTGCACTCATCTACGGCACTCAGAGAGCCGCCACTGTCAAGGCCGCTACCGACGTGACG
CTGTGGGCTATTGACCGCGTCACCTACAGGAGGATCCTGATGGGAGCCACCATCAAGAAG
CGAAAGATGTACGAGGGCTTCCTCGAGAAGGTCCCCATTCTTGCGCCGCTCAACCACTGG
GAGAGGCTCACTGTCGCCGACGCGCTGGAGCCGGAGGTCTACCACGACGGCGAGGTGATC
ATTCGTCAGGGAGAGAGGGGCGACTCTTTCTTCATCATCGTCGACGGCGAGACGAAGGTG
TCGCAGGTCAACGAGCAGGGCGAGGTGGAGGTCGCTCGCCTGTATCCCTCTTCGTATTTC
GGTGAGATTGCCCTTCTCACCGACAGGCCGAGAGCCGCCACCGTAACCGCCATCGGCAAT
GTCAAGGTGGTGAAGATGGACAGGGACCGCTTCAACCGGGTGATGGGTCCGTGCGAAGAA
ATTTTGAGGAGGAACATGGAGATCTACAACCAGTACATCTCCACCAAGATCTAG
>g1185-291087-292078
ATGACTACTACCATGACCCTTACCACTTTCAACGCCCTTTTCAGCAACTCGAACCCCACT
TCACCGCCCAGCCGATACGCCACCACCCCGCAGACCAAGAAGGCGAGGCACCTCGAGGCC
CGGTCTAAGAGGACCCCGCGCGGAGGAGAGCGAAAGGAGCCCATCAAGCAGCCACAGACC
ACCTCGGCCTCGCGGTCCGTGGCCAGCAGCGAGAGACAGCAACCAAGGACGCCCCCGTCC
AAGAGCGCTCCCACGACCCCCGAGAGGTGGGCGAACGGCCTGGTGTCGCCTCCTCCTTCC
GCCCTCCCGATGCCCTGCTTCGAAGATGACGACGATGTCGTGATCCCGTCCGCCCGACCC
GCCTCCTCCGCGCAACGCCAACTCGTGTTCGCCTAA
>g1185-371366-373850
ATGATGTTGCGGTCCAGCGTTCGGGGTGGCAGAGCCGTGGCGAGTGGTGCCTGCGTAAAG
GCCACCGAGGTGATGGGGTCCAGCCTCGTCTCCGCGCGTACTCTTGCCACTGCTGCGGCC
CCGGCCGCCGCGGCGACGGCGCCGTCCACACAGCCTCGTTCCTCCGCCCTCTTCGACTAC
AGACGCCTGAAGAACAGGAAGGGCTGGAAAAAAGTGGTGCCCGATGAGGACCTCGATTTT
GTGGTGTTCCCTCGCGAGAGGCTGGGAAAGACCTACGAGCTCAACTGGACCATCTGCAAG
TACGCCGTGATCCCTAACAAGGACGGCGAGGCCTTCCACAATCTCCATTCGCGAGGCCTC
CAGATGCTGTCCAACGCCGCTGCCGACAAGAACAAGGCGCTGCACGTCACCGTGCCCGAG
GAAGAGTACGCCTTCAGCCACTACTTCGTGCTGCCCTCGCCCCCGCCCGCTCCCGCCGAG
GGCCAGGCTGCCCCCGTCGACCTCATGTTCGTCCCCGAGAATGCCAGCGTCGTCAACGAC
GGTGTGGCCAAGAACGTCTCCAAAGAGGTGCGCCGCTTCCTCAGCGAGGGCAGGTACCTT
TTCGTGCACGATGGTGCCGTCGGCTCGCACTCGGCCGCCGAGGAGAAGGTCCGATTCATC
GTCTCCAGCGCCACCACTGCCCTCCTCCTCAAGCACTTGATACCCAAGCAGCCCACCGTC
AACATTCAGGACTTCGAGGAGAGCATCACTGTATTTGTCGCCCCTGAGCTCAAGGCCACT
GCTGAGGCGCTTGGTGTGTCGAGTGACAGGTTCACCCTGGTCAACTTCAAGACGAACCAG
GTCTTCGTGGCCGGCACCAACGCTCCTGAGGCCGTCCAGCAGGCCGTGTCTTCCCTGGCC
ACCTACTTGCTCGGCAAGAAGGGCGCGCTTCCGCTGCAGTGCGATTCGGCGCTCACCAAG
GATGGCAAGTCGGTTCTTTTCTTCGGCCCCGGCCTTCTCCAACAGAAGCCCCGCAACGAT
TTGTTCGGTGCTCACGGCCACTTCTGGACCAACGAGGGCGTCAGCCGCATGTATGACGGT
GCTTCCGTCGCCAACCCTGACGCCTCCCTTGCGCTGAACAGAGGAGACCTTGTGTTCCAC
GCCACCAAGGGCGGCAAGACGACGAAGACAAGCCTTAGCGTGCCGCTTAGCCTGCAGGGC
CACCAGGCTCCTCATCCCTCTGCCATCGTCTTTGTCTCTCCGAACTCTGCGGCCGGTTTG
ACCAAGATCTCTGCCGAGGAGGCGGCCAAGCACTACCTCGCCTCGTTCCAGTACCCTGTC
GCCGTCAAGCCCGAGCTGCTCAAGCAGAGATTCGCATACCTGCTCTCCAACAAGAACGTG
ACTGCCTACCAGGTTGGCGCCAAGAGCGGGAAGGTGAACGTGGACGAGATCAACGCCCAG
CTGCACACCGCCCTCAAGCAATAA
>g1185-403037-406298
ATGGCATCGTTTCTGGCCTCTCCCCTTGCTGGCGTTGCCGCTGTCGTGAGTGCCTACGTG
GCCTCGATCTTCGTCGTTGGTGGCTCCTTTGCTACCTTTGGCCTTGTTTTGGCGCTCTAC
GTGTACTACTACCTTGTTGGCTGGCACAAGAAGACCAATGAGGTGAGCAGCTACGAGGTC
GGTTCGGCTAAGTCTGGCGAGGGCGCTGTGAGGAGGAAGCTTGGCGTGCAGGAACTCGCC
ATTAGCCCCTACCCTGGCGTTACTACCCTCTACGAGAACTTCCAGCGTGGTGTGGAGAAG
TTTGGTGATAAAAACTGCCTCGGCACGCGTACGTTCGAGAAGAATGGCACGCGTGGTGCC
TACAAGTGGGAGACCTACAAGCAGGTCAGCAAGCGCATCAGGAACTTTGGTGCCGGCTTG
GTTGGCCTTTTCGATTTGCCCACCGGCGCTCGCGTTGGCTTCTACGCCAAGAACAGTGCC
GAGTGGGTCGTTGGTGCCGAGGCCTGCAACGCCTACAGCCTTGTTAATGTTGCCCTCTAT
GACACTCTCGGCGAGGAGAACAGAGTCTTCATTGTGCAGCAGGCCGAAGTTTTGGTCATT
ATCACCACCCCGGACTTGGTGAAGAACGTCGCGTCTCTCGTCAAGGAATGCCCTACCCTC
AAGGCTATTGTCGTGATCGGCGAGCTTAACAACGAGCAACAGGCCCTCGCCACCGAGGCG
GGCCTCGAAGTCCGCACTTTCGCTGACGTCGAGAAGGCCGGCGAGGCGGCGCCTGTCGAC
CTTCGTCTTCCTAAGCCCGACGACTTGGCTATTCTCATGTATACCTCCGGCACCACCTCT
ACCCCCAAGGGCGTGCTCATCACCCACACGAACCTCGTCTCCGCCGTCGCCGGTGTGCTC
CAAGCTGTGATGCCCATCTCTTCCGATGACGTCTTCCTTTCCTACCTGCCGCTTGCCCAC
ATCCTCGAGCGCGCCTCTGAGGCTGCCATGTTCTCCAGCGGTGCTTCCGTCGGTTTCTAC
CAGGGCGACGTCCGCAAGCTGGATGACGACATCAGGACTCTGGCTCCCACTCTGTTCGTC
GGCGTGCCCAAGGTGTATCAGCGCGTGATGCTCGGTATCCAGAAGAAGGTCGCTTTGTCC
GGCCCCATCGCCCGCTTTGTTTTCTACACCGCCTTCGCCATCCAGCAGAAGGCTATCGAA
AGTGGTTTCACGATCGGCCTTTTGAACAAGATTGTCTTCGCTAAGGTCCAGGAGGGCCTT
GGTGGTCGTCTGAGGCAGGCTCTCTCCGGCGGAGCCCCCATCTCCGCTGAGTGCCACCAG
TTCATTCGCATTTGCTTCGGCTGCCCCATCATGCAGGGCTACGGTCTTACCGAGACCTGT
GGTGGCACTGCTGTCACTCCCTATACTATGCCCAACCCCTACGGTCGTGCTGGCGTCCCC
ATCTCCTCCTGCGAGGTCAAGCTTGTCGACGCTGGAAACTACAAGACGGCTTCCAACCCG
CCTCGCGGCGAGGTTTGTGTCTCTGGCCCCTGTGTGACCAAGGGCTACTACAAGATGGAG
GAGAAGACGAAGGAGGACTTCCGCGAGGAGGAGGATGGTCGCGTCTGGTTCCACACTGGC
GATGTGGGTCAGTGGAATGAGGATGGATCGCTCAGCGTCATCGGCCGCACCAAGGACATC
TTCAAGCTCGACGGCGGCGAATACATTGCGCCCGAGAGGCTCGAGACCATCTTCGCCGGT
TGCAAGTACGTGGGTAACATCTTCATCTACGGTGACAGCACCAAGTCGTTCATCGTCGCC
GTCGTCGTGCCCGAGCCCATCGCTGCCAGGCACTGGGCTTCCGAGCAGGGCCTCAAGTAC
AAGGACGAGGACTTCACGGCCACGAGCGTGCCCGAGGACCTTTGCGAGAACGCGCAGTTC
AAGAAGGCCATCGCCGATGACCTCGCCAAGGTCGCCAACACGGCCAAGCTCAACAGGTTC
GAGTTCGTGACCGCGCTCCACCTCAGCGCCCACATGTGGACGCCCGAGTCTGGCCTCGTC
ACCGCTGCCCTCAAGAACAAGCGTCCCTCGCTCCAGCAGGCCTTCCAGAGCCAGATCGAC
GCCCTCTACGCCTAA
>g1185-436105-436798
ATGCCGCACTCCTTCGGTTACAGGGCCAGGACCAGGAAGCTGTTCGCGCAGCCCTTCAGG
AAGCACGGTTCCGTTTCCCTCGCCAAGTACCTGACCACCTACAAGGTCGGCGATTATGTG
GACATCAAGGCCAACGCCTCGATCCACAAGGGTATGCCGTTCAAGTTCTACCACGGCAGG
ACCGGCGTCATCTGGAACGTGACTCCCCGCGCTGTCGGCGTCGAGATCAACAAGCAGGTC
CGTAACAAGATCATCAAGAAGAGGATCCACGTCCGCATTGAGCACATCCAGCCCTCGAGG
TGCAGGGAGGACTTCCTCAAGCGCGTGAAGGCCAACGACCTCGCCAAGAAGGAGGCCAAG
GCTAAGGGCGTCAAGGTGCACCTCAAGAGGATGCCCGCCCAGCCCAAGCCCGGCAGGATT
GTCAAGGCTAAGGCCACTTCCGCCACCACCATTGCCCCCCTCAAGTACGAGCTTCTTGTG
TAA
>g1185-436847-438218
ATGAGCGCCAGGCGATTCTTTGTGGGCGGCAACTGGAAGGCGAATGGAGGCCAGAGCTTC
GTCCGCTCCCTCGTAGCGGACCTCAACGCAGGAAATGTCCCTGAGGACGTTGAGGTGGTG
ATTTCCCCGCCCTCGCTCTACCTGGCCCAGGTGCAGGAGCAGGTGCGCAAGGAGATCGCT
GTGTCCGCACAGAACTGCTACCACCAGGACGGTGCCTTCACGGGCGAAGTCACCGCTTCG
ATGGTGAAGGACCTTGGTCTCCCGTGGGTCATCCTGGGCCACTCCGAGAGGAGGAGCATC
TTCAAGGAGAGCGATGAGCTGATTGGCACCAAGGTCGCCAAGTGCCTGGGTGTGGGCCTG
AAGGTGATCGCCTGTGTGGGCGAGCAACTTTCGGAGCGTGAGGAGGGCAAGACCACCGAG
GTCGTCTTCTCTCAGCTCAAGGCCATTGCGGCCAACGTGACTGACCCCAAGGGCTGGGAG
AACGTCGTTATCGCGTACGAGCCAGTGTGGGCTATCGGCACCGGCCGCACGGCGACCCCC
AAGCAAGCCCAAGAGGTGCATGCCGAGATCCGTAAGTGGCTTCACGATAGCGCTTCCGCT
GAAGTGGCCGCCACCACCAGGATCATCTACGGCGGTTCGGTCAAGCCCGACAACAGCGAC
GAGCTGGCGAGCGAGCCCGACATTGACGGTTTCCTTGTTGGTGGAGCCTCGCTCAAGGCG
GCCGACTTCTTCAAGATCGCTTCCGCTGCCAACGCCAAGAAGAGCGCCAAGCTGTGA
>g1185-477296-479799
ATGAAGGTCGCGGCGTTGGTGCTTTTGGGCATCGCCCTCCTGTTCGCGAGCGTGCACGGT
CAGGATGAGAACAACAAGTTCGAGGGTCCTATCATCGGTATCGATCTCGGCACGACCTAC
TCCGTGGTAGGCATCTGGAAGAATGGCCGAGTGGACATCATCGCCAACGACCAGGGTAAC
CGAATCACCCCGTCCTACGTGGCGTTCTCGGACAGTGAGCGCCTCATCGGTGACGCCGCC
AAGAATCAGGCCGCGCTCAACCCTGAGAACACTGTCTTTGACGTCAAGCGTCTGATCGGT
CGCAACTTCGCCGACAAGGAGGTCCAGGCCGATGCCAAGCTCCTGCCGTACAAGATCATC
AACAAGAACGGCAAGCCCTACATCGGCGTCGAGTACAAGAACGAGGCCAAGGAGTTCGCC
CCCGAGGAGATTTCCGCCATGGTCCTTACCAAGATGAAGGAGATCGCCGAGGCCTACCTT
GGCAAGACCGTCAAGAACGCCGTCGTCACCGTCCCGGCCTACTTCAACGACGCCCAGCGT
CAGGCCACCAAGGATGCCGGTGTCATCGCCGGTCTCAACGTCGTCCGCATCATCAACGAG
CCCACCGCCGCCGCCATCGCGTACGGTCTCAACGAGGTCAAGGGTGAGAAGAACATTCTC
GTGTTCGACTTGGGTGGTGGTACCTTCGATGTGTCGCTCCTCCAGATCGACGATGGTGTG
TTCGAGGTGCTCGCCACGAGCGGAGACACTCACCTCGGTGGTGAGGACTTCGATCAGCGC
GTGATGCAGTACATGCTCAAGCAGTTCAACAAGAAGACTGGTCTCGACGCCGGTAAGGAC
AAGCGCGCCATCCAGAAGCTGAGGCGCGAGGCCGAGAACGCCAAGAGGACCCTCTCCACC
ATGAAGGAGGTGTCGATCGAGATCGAGAACTTCTTCGAGGGCCAGGACCTCAGGGAGAGG
CTCACCCGCGCCAAGTTCGAGGACCTCAACTTGGACCTCTTCAAGAAGACGCTCAAGCCC
GTCCAGAAGGTCATGGAGGACTCCGGCCTCAAGAAGACCGAGATCGACGAGGTCGTGCTC
GTCGGTGGTTCGACCCGTATCCCCAAGGTGCAGGAGCTCATCAAGAACTTCTTCGACGGC
AAGGAGCCCAACAAGGGCGTCAACCCCGATGAGGCCGTCGCCTACGGTGCCGCCGTCCAG
GGAGGCATCCTCTCGGCCGACAAGAAGGACGACGAGCTGAACGACATCGTGCTGCTCGAC
GTCACGCCGCTCACCCTCGGCATCGAGACCGTCGGAGGCGTCATGACCACCATCATCGAG
AGGAACACCCTCATCCCGACCAAGAAGACCCAGACCTTCACCACCTACCAGGACCAGCAG
GAGGCCGTCACCATCCAGGTCTACGAGGGCGAGCGCGCCATGACCAAGGACAACCACAAC
CTCGGCAAGTTCGACCTCAAGGGCATCCCGCCCGCTCCCCGTGGCGTGCCTCAGATTGAG
GTCACCTTCGACGTCGACGTCAACGGCATCCTCCACGTCACCGCCGAGGACAAGGGCTCC
GGCACCAAGAAGTCCATCACCATCACCCCCGAGAAGGGCCGCCTCACCGAGGAGCAGATC
AAGCGCATGGTCCAGGAGGCCAAGGAGCGCGAGGAGGACGACAAGCTCATCCGCGAGCGC
GTCCAGGCCCGCAACGGTCTCGAGAGCTACGTCTACCAGATCCGCAACACCATCCAGGAC
AAGGAGAAGATCGAGGACAAGCTCAGCGAGGACGACCTCGAGACCCTCGAGACGCTCGTC
AAGGACTCCCTCGAGTGGCTCGACGACCACCAGGAGGCCGACAAGGACGACTACGAGGAG
AAGAGGAAGGAGATCGACCAGGTCGTCTCCCCCATCTTCACCCGCATGTACGGCGCCGCT
GGCGCTGAGGGCGGTTTCCCCGGTGGCGCCGACGGTGCCGACGAGGACTTCGGTGCCTAC
GACGAACTGTAA
>g1185-508426-509120
ATGGCATCTGACGGCGACAAGCACGGCGCCACCGCCGCTGCCTTGAAGGTCGAGCCCAAG
GACACGATCTTCGCTAAGATCGTGGATGGCACCATCCCCGCGAAGAAGGTCTTCGAGGAC
GACACGACGATTGCCTTCCATGACATCGCTCCCCAAGCGCCCACTCACATCCTCATCATC
CCCAAGAAGCCCATCGGCGGAATTGGAGACGTGAAGCAGGACGAGGAAGCGCTCGTGGGC
CACCTCATGTTCGTGGCTACCCAAGTGGCCCGCGAGCAGGGGCTGGGCGACGATGGCTAC
AGACTGGTCATCAACGAGGGCACCAATGGACAGCAGTCCGTCAGGTGGCTCCACATCCAC
CTCCTCGGCGGCCGCAAGCTCACCTGGCCTCCCGGCTAA
>g1185-648266-652142
ATGAAGAGCCGCAAGCACTCAGGATCGATCAAGAACAGGAGCGAGAGCTTGGCACAGCCC
TTCCTCAACATGACCGTGGCGAAGGTCGAGCAGCCCGAGAAGGAGGGTTTTCTCCACAAG
AACGGCGAGAAGCGCTGGTTCGCTCTCAAGGACGCGCGACTCTACTACTTCAAGAACAAG
GATGCCCAGACGCCACTCCAGGGCGTTATCGAGCTCGACGCTGCGACCGTCATCGACAAG
AAGGAGGGAGACAAGAAGCACGGCTTCGATGTCACGCCTCACGACGCCAAGGCCTTCCAG
CTTCGCGCTCACGATGAGCAGGACAAGGAGGCCTGGATTGCCGCGATCGAGAAGGCCGCT
GCCAAGACGCCCGCTGCCGCCAAGGACAGCGATAAAGAGCCCGAGAAGCAAGCCGAGGTG
AAGGGCAAGCAGGCCAAGCGCAACAGCAAGAAGGAGAAGAACAAGAAGGCGTCGAAGGAT
GACTCCGACTCTTCATCTTCTGACTCGTCCTCCGACGAGTCCTCCTCCGACGACGAGAAG
GAAGACCACAAGAACAAGGCCAAGCCCGAAGAGAAGGCCGCGAAGTCCGAGGAGAAGCCC
AAGGAGGAGGCCAAGCCCGAGGAGAAGGAGAAGGAGGAGCCCGCCAAGGAGGAGGTCAAG
GCTGTCGCTAAGCCTGACGACAAGAAGGCGAAGAAGGAGGAGAAGAAGAAGCCCGAGAAG
GAGGAGAAGAAGCAGGAGCGACCCACTTCGCCGCAGCCCAAGAAGAATGCGTTCGGCTTC
ATGAGCCTGAAGAGGCACAAGAAGCCCGCCGACGACGCCATCGAGAAGCCCGAGGAGAAG
AAGGCCACTCAGGAGAAGAAGCACGAGGAGGCCAAGCCCGAGAAGAAGAAGGAGGCCAAG
CCCGAGAAGAAGAAGGAAGCCAAGCCCGAGAAGAAGAACGAAAAGAAGGGCAAGAAGGAG
GCCGCGGCAGTCAAGCACGAGGAGGAGGAAAAGTCCGAGGAGAAGGAGCCCGCTAAGGAG
GAGGCCAAGCCCGAGCCCGTCGTCGAGAAGGCTGCAGTGCCCGCCGAAGAGCACAAGGAA
GCTCCCAAGGAAGCCGAAGCCGAGAAGCCCGAGGAGAAGGAGGAGGAGCCCGCCAAGGAG
GAGGCTAAGCCCGTCGAAGCCGAGAAGGCCGCCGCCGTCGCTGTCGTCGTTGTGCCTGCC
GAGGAGCACAAGGAGAAGGAGGAGAAGGATGACTCTTCGTCTGATTCCGACTCTGACTCT
TCCTCCGACTCTGACAGCGACGACGACGACAAGAAGCCCAAGGAGGAGGCCAAGCCCGAG
GAAAAGAAGGAGGAGGCCAAGCCCGCCGAGGTCGAGAAGCCCGCCGAGGAGAAGAAGGAG
GAGGCCAAGCCCGCCGAAGCCGAGAAGGCCGCCGCCGCCGCGCCCGCTGCCGAGGAGCAC
AAGGAAAAGGAGAAGAAGGACGAGAAGAAGAAGCCCGAGAAGAAGGAGAAGCCGGCCAAG
GACGAAGTCACTTCGCCTCGGAGCGGCAGGAAGGCCGGCAGCATCTTCAGCATCTTCGGA
AAGAAGGGCAAGAAGGACGAGAAGCACGAGGAGAAGAAGGAGGAGAAGAAGGAAAAGAAG
CACGAGGAGAAGAAGGAGGAGAAGAAGGAGAAGCACGAGGAAGAGAAGCACGAGGAGAAG
GAGAAGGTGGCCGAGAAGGCCGAAGCTCACGAGGAGAAGCACGAGGAGGTCAAGGCTGTC
GTCGCCGCCGAGGTCGCGGTCGAGGCTGAGGAGAAGGAGGCGCCCGCCGCCAAGGCCGAG
GAGAAGGCCGAGGAGGACAAGCCCGCCGCCGCCGTGGATGCCGATTCCGACTCGTCCTCC
GACTCTTCCTCTGACTCCGATAGCGACGACGACGACAAGAAGCCCAAGGAGGAGGCCAAG
CCCGAGGAGAAGGAGGAGGCCCCCAAGGCCGAGGAGCAGGCTGCTCCCGTCGATGCCCCC
GCTGCCGAGAAGGAGGAGGCTCCCAAGGAGGAGGCCCCCAAGGCTGGCGACGAGCACCAC
GAGGCTAAGGAGGAGGAGGAGGCCAAGCCCGCTGAGCCCGCCGCCGCCGTGGACGCCGAC
AAGGGCAAGGAGAAGGACGAGGAAGAGGTCAAGCCGCAGCTGACCCGAGGAGCCATCGTC
GGTCTCTTCACCGCCCAGGCCGCTTTCGTGCCCGAGGACAAGCTCCGCGAGTGGTGGACC
AAGGCCTCCGCCGAACTGTCTGAGCTCGAGGACGAGGAGCTCAAGAACAAGCACGAGGAG
ACGCCTAAGGAGAGGAAGGAGAGGCTCAAGAGGGAGGCCGCCGAGAAGAAGCGCCAGGAG
CAGGAGAAGAAGAGGAAGGAGAAGGAAGAGAAGAAGAAGAAGGCCGCCGAAGAGAAGGAG
AAGAAGAGGAAGGAGGCCGAGGAGAAGAGGAAGGAGGCCGAGGAGAAGAAGAAGAAGGCT
GATGAGGAGAAGAGGAAGCACCTCGAGGAGAAGAAGAAGCTCGAAGACGAGAAGAAGAAG
GAGGCCGAGGAGAAGAAGAAGCTCGAAGACGAGAAGAAGAAGGCCGCCGAGGAGGAGCAC
AAGAAGAAGGTCGCCGAGGAGGAGCTCAAGAAGAAGGCCGACAAGACCAAGTCCCAGGAG
GAGAAGGAGGTCGCCGACATCAAGAAGAAGGCCGACAAGACCAAGTCCCAGGACGAGAAG
GAGGTCTCCGACTCAAGCAGCTCCGACGACTCTGAGTCTGACTCAGACGACGAGGAAGGC
GAGAAGGCCGCCAAGACCGTCGTTGCGAAGGAGAAGGTCGCCGTCGAGGTCAACGCCGAG
AAGAAGGTTGAGACCAAGAGGGAGAGCGACTCGGACGACAGCAGCTCCGACTCAGACGAC
TCATCGGATGACGACAAGAAGAAGAGCGACAACGACTCCTCCAGCGACAGCGAGAGCGAG
AGCGACTAA
>g1185-664408-669318
ATGGAGGGACAAATCGAGAAATCGACAGAGCAGACCGCGCAGCAGCCGGAGCAGAAGCCC
CAGCAGCAGCCGAAGGCCAAGAAGCAGCCCGCCCCGAAGAAGCAGCCTGCCCCGAAGAAG
GCCGCGCCCGCTGCTGGCGAGAAGAAAGCCAAGAAGGATGAAATCCGTCTGAAGAAGGAA
TTCCACAATACTACTCCGAAGGGTGAGAAGAAGGATATGTCACAGCCCATGCTGAATGAG
TACGATCCGCCGGCCGTCGAGGCCGCGTGGTACGACTGGTGGGCCGCACAGGGCTTCTTC
CGCGCTGATGAGAACGATCAGACCAAGGAGAAGTTCGTCATCGTCATCCCGCCGCCGAAC
GTCACCGGCAGCTTGCACATGGGCCATGCCCTTACCAACTCCATTCAGGACTCTCTGTGC
AGATGGCACCGCATGAGCGGTAAGAACGTCCTGTGGGTTCCGGGCACAGATCATGCCGGT
ATTGCCACGCAGGTCGTTGTCGAGAAGAAGCTGAAGAAGGAGCGCGATCTTTCGCGTCAC
GATCTCGGCCGCGAGAAGTTCATCGAGGAGGTTTGGCGGTGGAAGAACGATTATGGTGCT
CAGATCTGCAATCAGCTTCGGCGATTGGGATCTTCCCTCGATTGGTCCCGCGAGGTTTTC
ACCATGGATGAGAAACTGTCCGTTGCGGTCGTCGAAGCGTTCGTTCGCATGTACGAAAAC
GGTCTCATCTACCGTGGCACTCGCCTGGTCAACTGGTGTACCGAGCTCAAGACCGCCATT
TCCGACGTCGAGGTCGAGCACAAGGACCTCAAGGGCCGCCACAAGATGAAGGTCCCCGGC
TACGGCAAACAGGAGGTCGAGTTCGGTGTCCTCACTTCGTTCGCGTACCCCATCGAGAAC
TCGGAGGAGCAGATCGTCGTCTCCACCACCCGTATCGAGACCATGCTCGGAGATACCGCC
ATCGCCGTTCACCCGGATGACGAGCGCTACAAGCATCTCCACGGCAAGTTTGCGGTCCAC
CCTTTCAACGGCCGACGGATTCCCATCATCACCGACTCGATCCTCGTCGACAAGGAGGTC
GGAACTGGTGCTGTTAAGATCACACCCGCTCACGATCCCAAGGATTACGAGTGCGGTAAG
AGGCACAGCCTGGAGATGATCAACATCTTCACCGATGACGGCCTCATCAACGAGGAGGGC
GGTGCTCCCTTCACGGGCATGAAGCGCTTCGACGCTCGCGTTGCCGTCACGCAGGCGCTA
GAGGAGAAGGGTCTGATCAGGGAGAGCCAGGACAACCCCATGATCATCCCCATCTGCTCG
CGTACCAAGGACGTCATCGAGCCTCGCCTCAAGCCCCAGTGGTGGGTCCGATGCAAGGGC
ATGGCCGACGAGGCCGTCAAGGCGGTTAGGGAGAAGCGCCTGGAGATCGTTCCCTCGATG
CACGAGGCTGTCTGGTACAGATGGCTGGAGAACATCCAGGATTGGTGCATTTCTCGCCAG
CTCTGGTGGGGCCACCGCATCCCTGCCTACCTCGTCCACATCGACGGACAGCCCACCCCT
GATGCCAAGGCCAAGCACGCCGACGTCGCGCCGGAGAAGATCAAGCTCGAGCAGGACCCC
GACGTCCTCGACACGTGGTTCTCTGCGGGCCTGTTCCCGTTCTCAGTCATGGGCTGGCCC
AACGAAACTGCCGACCTCAAGGCCTTCTACCCCACTTCTCTCCTCGAGACGGGCCACGAC
ATCCTGTTCTTCTGGGTCGCCCGCATGGTCATGATGGGCCTCAACCTCACCGGCCAGCTG
CCCTTCTCGCAGGTCCTTCTGCACGCGATGGTCCGCGATGCTCACGGCAGGAAGATGTCC
AAGTCGCTGGGCAACGTCGTTGATCCGATCGACGTCACGGAAGGCATCCGCCTGACGGAT
ATGCACCAAAAGCTCCGCGAGGGTAACCTCGAGGCCTCCGAAGTCGAGAAGGCCATCAAG
GGCCAGCAGAAGGACTTCCCGAACGGCATCAGCGAGTGCGGCACCGACGCCATGCGCTTC
GCCCTCTGCGCCTACACCTCGCAGGGGCGCGACATCAACCTCGACATCAACCGCGTCGTC
AGCTATCGTCACTTCTGCAACAAGCTCTGGAACGCCACCAAGTTCGCGCTCATGAACCTC
GGCGCCGACTACGCGCCCCTCGCCTCGCCTGACGTCACCGGCCAGGAGAGCATCATGGAG
AAGTGGATCCTCAGCCGCCTGCACTCAGCCGTCAAGGACGCCGACCAGGGATGGAAGTCG
TTCGAGCTTGCCCAGTGCACCACCGCCATCTACAACTTCTGGCTCTACGAGCTGTGCGAC
GTCTACCTGGAGGCCATCAAGCCCGCCATGCGCAACAAGGGCAGCCCCGAGCAGAAGTCG
GCTCAGCACACGCTCTACACGTGCCTCGACTATGGCCTCAAGCTGCTCCATCCGTTCATG
CCCTTCGTCACTGAGGAGCTCTACCAGCGCATCCCCAGGCGCCCCGGCGACAACATCTGC
AGCATCATGGTCTCCCCCTACCCCAAGCCGGCGTACACCCAGGCGTGGGCCAACGAGAAG
CTGGAGGCCGACGTCAAGCTGGCACAGGACATCATCCGTGCCTCGCGCGCCCTGCGTGCC
GACAACGGCCTTCCGCCGAGCTCGAAGCCTACCTTCTTCCTCGAGTTCCACTCGGACGAG
CTGAGGAACACCTTCCGAGGCTTCGCGCCCATCATCCTCACCCTCTCTGGTGCTTCGGAG
GTCAACGTCCTCGAGGGCGACGCCAAGCCGCCCAGCGGCTGCGCTGTAAATATTCTCAAC
GAGAACGTTGAAATCCACATCTTCCTCAAGGGCTTGATCGACCCCGACGTCGAAGTCAAG
AAGCTGGAGAAGAAGCTCGGCGAGTCGACCACGCAGCTCGAGACTCTCGAGAAGAAGATG
AAGGCGCCCATGTACGAGGACAAGGTCCCCGCCGCCGTCAAGCAGTCCAACACCGAGCGC
GTCGGCAAGCTGAACCAGGAGATCGAAGCCATCAAGCAGGCCATCGAGAAGTTCAAGGCC
ATGAAGGCCAACTAG
>g1185-738627-740358
ATGGATCACGGCAGAGTTCAGGTCATTGGATCTCAGGCAGAGTACGCGCAGGCGCTGCTC
GTTGCCACAGCCGCAGGCCGGTTGGCCGTGGTCGATTTTACCGCCAAGTGGTGTGGCCCC
TGCAACGCGATCGCGCCCGTCTTCGTTCAACTGAGCAACAAGTACCCCACAGTCGACTTT
GTCAAGGTGGATGTCGATGAGCAGGCAGAGATCGCCGCCGCGCACGGCGTGAGCGCCATG
CCCACGTTCATATTCATCAAGAACGGCCAACGTATCGACGAGCTGAGGGGCGCCAACGCA
GCCAGGCTCGAGCAGCTCATCCAGAAGCACAGCAGTGCCGGTGGAGCCGAGGAAGAGGAG
AGCGACCTGAAGCTTCCCGCTGGCCACAGCGACGTGACCCAGAACGTGGACAAGGCGCAG
GGCGAGTGCCTCAACCAGAGCACCGAACACAAGTGGGACAACATCTTCAAGAAGGACGAT
TCCTTCCTCGAGAGCGACACCGACGAGCAGCTCCTCCTGTATATCCCCTTCAACCAGCCC
ATCAAGATCCATTCGCTCTGCTTTCAGGCGCCCGATGATGGCCGGGCTCCGCAGACGGTG
AAGCTCTTTGTGAACAAGCGAGACATGGACTTCCAGTCGGTTGACTCTGCGCCCGCCACC
CAAGTGATCGAGCTGAAGGCCGACGACGTCAAGGGCGACAAGCTTGTCCCTCTGCGGTTC
GTCAAGTTCCAGAACGTGAGCTCGATCACCATTTTTGTAGAGAGCAACCAGGGTGGGGAG
CCTACCACCGTCATCCAGCGTCTGCGCTTCATCGGCCAGGCCCAGTCCGCCACCAACATG
AACGAGTTCAAGAGGGTGGCTGGCGAGAAGGGCGAGACGCATGGCTAA
>g1185-746077-748318
ATGCCAGTAGAGACGCGGCTGTACGATGTGCTGGGAGTGGGTCCCGATGCATCGCTTGAT
CAGATCAAGAAGTCCTACAAGCGGCTAGCGATGAAATATCATCCGGACCGTAATCCCAAT
GCAGAGGACAAGTTCAAAGAGATCAGCCTGGCCTACGAAATCCTGAGCGATGAGGAAAAG
AAGAGGGCCTACGATCGACACGGCGAGGAGTACCTGAAGCAGGGCGGACCCAGCCACGCC
GGCCCCAGCGATCTGTTCTCCCACCTGTTCGGCATGGGCGGCGGACGTGCGCGGCAGCGT
AAGGGCGAGGATCTCGTCTTCCCGCTCAAGGTCACCCTCGAAGATCTCTACAACGGCAAG
ACCACCAAGGTGGCCCTCAAGAAGAAGGTCATCTGCGACGAATGCAACGGAAAGGGGACG
CCTGTGCCTAATGCGCTGCGGACATGCGAGAGCTGTGACGGCCGGGGCATCAAGCTGACC
TTGCGTCAGCTCGGGCCTGGCATGGTGCAATGCCCCGATTGCGGTGGCGAGGGGCAGGTG
ATCAGGGAGAGGGACAGATGTAAAAAGTGTTCGGGGTTCAAGGTGGTACAGGAACGCAAG
ATCTTGGAGATCTTTGTGGACAAGGGAATGAAGCACAAGCAAAAGATCGTGTTTACGGGC
GAGGGAGACCAGGAGCCCGGCGTCACCCCCGGTGACGTCATCATCCTCCTCAACCAGGAA
GACCACCCGGTGTTCAAGCGAGACGGCAAGAACCTGTTTATGGAGAAGGAGATATCGCTC
TTTGAAGCGCTCTGCGGTTTTTCCTTCACCCTCAAGCACCTCGACGGCCGCACGCTGCTG
GTCAAGTTCGGCAACGGGCAGGTGGTCAAGCCGGGCGACCTCAAGGAGATCCCCGACGAG
GGCATGCCCACCTGGAAGCAGCCCTTCGACAAGGGTCCCCTCGTCATCAAGTTCAACGTC
AAGTTCCCCGACTACGTCAACCCTCAATCCAAGCCCATGCTGGAGCAAGTGCTGCCGGGC
GGACCGGAGCCGATGGACTTTGCGGCGAGCGGGGCGGTCGAGGTGGAGGAGGTCACGATG
CGCGACTACCGACCGGAGGCCCGCAACGCGCGCGGCGGCGCCAACGGCCAGCAGCGGCGA
GAGGCCTACGAGACGGGCAGCGACGACGAGGACCACCCGTACGGCGGCGGTGGCGGATCC
GGCGTGTCGTGCGCCCAGCAATGA
>g1185-770357-772028
ATGGCAGCAAGATCACCTGTGAAGCCTACTGGCAGCCCGTCGGTTGCCTCCAAGCAGAAG
GAGGCGAAGCCTAGCCTCGACAAGGCCGATGGCAGCAACCTCGACGTGGAGATCATCGAG
GTGCCTGACGTCGACCTGAGCCTCGAAGAGTGGAGCATGTGCGCGCACAAGCTCAAGCCA
GGGCAGAACATCGACATCCTCGTCACGGTGCGCAATCTCAGCAGCCGTGCCATCACCGAG
AGCCTCTCCCTCCGCGTGGACAGGGACGCCTTCTTCCAGTCGGTCGCCGTCAGGCTGCCG
TCGTCGGTACCGGCCAACGGCGAGGTCAAGGTCAAGGCCACACTCAGGGTGCAGGGCGGC
ATCACCAACCTCAATAAGCTGCCCGCCAACAAGGAGATCATGCTCGTGAAGGAGTGCGGT
CCCGGCAAACTGGCCAAATTGTCGTCCATGTTCATCGTCATCCCCATCGATCCCTTCATC
CGCTACCTCAAGGAGGAGTTCCGCCCGATCCGGCTTCAGCTGTGGGGCCTCTTCGGCACG
GGCAAGTCGTCATTCATCAACTCGATGGCCACGCTCTACAACGAGGACCCCCGGTTCCCG
AACAGGAAGCTCACGCCCGCATTCTCGAGGCCCTCGGGGGTGTCGGTGACGACCGAACTC
AAGCAGTACGACTTCAACAACGTGAGCCTCTGCGATTCGTGGGGCTGGGAGGAGGCGCGC
AAGGACCTCTACTCCGATCTCCTGTTCCAGCTCATGCTCGGCGGTCGGCTGGGCAGAGAG
TTCGCCATGGACAACGCCAAGACCCTCAGCGTGCTCAAGCTGCCCAACGTCGAGACCGCG
CCCGTCGACCTGGTGCTGTTCTTCATCACCGTGGGCGCCGTCGAGAACAACACCTACCTG
GAGCAGATGAACCACTTTATCGGCGAGGCGCGCAAGCTCGGCGTGCCCCTGCTCGTCCTG
TTGACCCAGATCGACAAGGTCGACCCTAGCCTGCGCCAGGACCCCTACAGCGACAACCCG
ACCGTGCAGGACCTTGTCACGCAGGTGTCGCTCAAGGCCGAGCTCGACGAGTCCTTCATC
TTGCCCGTGGTCAACTACACGCGCGAGACCGAGAGGACTTGGGCGATGGACCGAGCCGCC
TTCGTCACCCTCTACCGGGCCTTCCAGGCACGTGAAGTCGCTGTCGGCAACGCCTAA
>g1185-881499-883150
ATGGCCACCACTACACCTGCAGGCAATGGGGTTGAGAATGGACGCGGATCGTTCAAGGTG
AAGGCGGGACTGGCGCAGATGCTCAAGGGCGGCGTGATCATGGACGTCATCGATGCCGAG
CAAGCGCGCATTGCCGAAGAGGCCGGCGCCGTTGCCGTCATGGCCCTTGAGAGAGTGCCC
GCAGACATCAGGCGAGACGGCGGAGTGGCCCGCATGAGCGACCCGTCGATGATCAAGAGC
ATCATGGAGGCGGTGTCGATCCCGGTCATGGCCAAGGTGCGCATCGGCCACTTTGTCGAG
GCGCAGATCCTGGAGGCCGTCGGCGTCGACTACATCGACGAGAGCGAGGTCCTCACCCCG
GCCGATGACGAGAACCACATCAACAAGCACAACTTCGGCGTCCCCTTCGTCTGCGGCTCA
CGCAACCTCGGCGAGGCCCTGCGTCGGATCAGCGAGGGCGCCGCCATGATCCGAACCAAG
GGCGAGGCCGGGACGGGCAACGTGGTGGAGGCGGTCAGGCACGCCCGAACTGTGAACGGC
GAGATCCGCAAGCTGACGACCATGGACGAGAACGAACTCTACACCTACGCCAAGACCATC
GGCGCCCCGATCGACCTGGTGCAGCAGACGGCCAAGCTGGGCCGCCTGCCCGTCGTCAAC
TTCGCCGCCGGCGGTGTGGCCACCCCGGCCGACGCCGCCCTCATGATGCAGCTCGGAATG
GACGGCGTGTTCGTGGGTTCGGGCATTTTCAAGAGCGGCAACCCGGCCAAGCGGGCGCGA
GCGATCGTGCAGGCGGTGACCCACTACAAGGACCCGCTCGTGCTGGCCAAGGTGTCGGAG
GACCTGGGCGAGGCCATGGTGGGCATCAACTGCGACCAGATGGGCGAAAAGTGGGCCAAG
CGGGAGAGCGGCGCCGCCACCCCATCCAACGCCCACCAGTAG
>g1185-888926-890580
ATGGAGCGCGTGAATAACCTCATGGTGTTTGATATTCTGCCCGAGCGCAAGGTGGTGTTC
CTCGAACATAACATCAACGTGCAGGACACCCTCTCCATCCTTGACGACAAGCGCATCTCC
TCCGCCCCCGTGGTGAACGTGAGCGAGGGCAAGTTCCTGGGCATCGTGGACATGTTCGAC
ATCGTCACCTGCATGCTGGGCGTGCTGCCGGAGCTGAAGGAGGGCGACGACCCCAGCGCC
GTCGAGTGGGCTGGCGAGCACTTTGCCAAGACCACCATGGGCCAGGTCATCGAGGCCACC
AACACGTACGAGGGCTTTCCCATCGCCTCCAACCCGGTCAAGCGCGAGACCTTCCTGCCC
AAGGTCATCGAGCTCTTCTGGCTGGGACTGCATCGGCTGCCGGTGGTGAACATGGAGAAC
AACATCATCAACGTGCTCACGCAGAGCGACCTGCTGGCGTTCATGGCGCAGAACATGCAC
CTCATCGGCGCCATCGCACGCAAGACCCTCGACGAGTGCAACATCGGCCGCGTCGCGCCG
CAGATGGCGCGGGCCAACGAGCAGACTAGCGTCGTCGTCAAGCGCCTCCACGACAAGCGC
ATCACCGCCCTCCCCGTCGTCGATGACGAAGGCAAGATCGTCGCCAACTTCAGCATCAGC
GACCTCAAGGGCATCACGAGCAAGAACTTCAAGGACCTGCTGCTGCCGGTGAAGGCCTTC
CTGGACAAGCGGTCGTCGCAGGAGGAGAACTTCCGGTGCGAGCGGTCGCTCCACCCGCTC
ACGGTCCAGCGACACGACCCGCTCGAGGAGACCATCTACAAGATGGTCGCCACGCGAGTC
CACAGGCTCTGGGTCGTTGACGACAGCAACCGACCCATCGGCACTGTCTCTACCACCGAC
CTCATGCGCGCCTTCCTGCAGCTCTGA
>g1185-901627-902408
ATGGGTTCTGGCAAGCCTAGGGGTATCCGCGCTGCGCGCAAGCTCAAGAACCACCGCAGG
GTCGAGAGGTGGTCCGACAAGCAGTACAACAAGGCTCATTTGGGCACCCAGCTGAAGGCT
AACCCCTTCGGAGGCTCTTCGCACGCCAAGGGCATCGTGCTCGAGAAGCTCGGCATTGAG
GCTAAGCAGCCTAACTCTGCCATCCGTAAGTGCGTCCGTGTCCAGCTCATCAAGAACGGT
AAGAAGATCGCTTGCTTCGTCCCCCGTGACGGTTGCTTGAACTTCATCGAGGAGAACGAC
GAGGTGCTCGTGGCTGGCTTCGGTCGCAAGGGTCACTCCGTGGGTGATATTCCCGGCGTC
CGTTTCAAGGTGGTCAAGGTCTCGAACGTGGGTCTCTACGCCCTCTGGAGGGAGAAGAAG
GAGAAGCCCAGGTCTTAA
>g1185-910386-914107
ATGTCTGAGGGGGATCACACCAACGATGCCACAGAGCACACGACCACCGGCGAGGAGAAT
CTCGCCAACCAGGGAAGTGAGAACGTTGAGGGAGGGGAAGGTCTGAAGGCCGGAGCGGGT
GCCGAAGGAGGAGATGTTCACGGGGACGAGGCATCGGACGAAGTGAAAATCAATGAGGAC
GGACAGAAGCGAGCCTTCGGCGAGCAGGAGGAGGGAGAGGAGGATGGCGAGGAGTACGAT
GATGAGGAGGAGTACGACGACGATGAAGACGATGACGGCGATGATGATCAAGATCAGGAC
CTCTCGGCCAAGCTCAACGAGCTTCGCGGCATGATGGGCAGCTTCTACCCGTTCGGTACT
CAGAACCAGTACACCTCGGTTGACCTGGTCCTGAGCAAAGAGGATTTCGAGCTTGAGGAG
CTGTTGGAGCTCGACGACTTCCTTCCCGATCTGCGAAGGGGCAAGTCGAACCTTCTCTCC
TACCTGGTTCAGCCTGAGGTCTTGGAGCAGCTCGTTGCCTATATTTCCTCCGAGCCCAAG
GAGGACGAGGATGAGGATGAGGCGGAGCAGGAAAAGCAGAAGCGGTTCGCTCATCTTGCC
TACGAGGCCATCCAGTCTGACATCCTCAAGGAGGCGATGATCAACGACCACAACATGCTC
GACAAGTTCTTCGATTTCTTGGACATAACGACCGAAATGGATCCCCTCCGCTGCGCCTAC
TTCTCGCGGATCGTCGAGCTGCTCTTCGAGCGCTACCCCAAGGAGCTGATCTCCTACCTC
AAGAAGAAGAACTGCGTCGTGGCCAAGTTCGTGTGGCACATCAACAACCAGTACATCGTC
GAATCTCTCTTCCGGCTGATCGATTCGCAGATCTCCCACCAGTGGCTGCTCGACGAGGAC
CTCATCCCGCTGCTCATGTTCAAGATGGCCGAGTGCGAGGACCCCGAGACCCAGGAGAAG
ACGGCCAACACCTTCGTCGAGATCCTCGACCTCTTCGTCCCCTTCCCCTACTCTATTCTC
ACCACCCAGATGTTCCACTCGGAAATCGTCGCCGAGCAGTGGGAGCAGTTCACTCTCTCC
GAGCCGGCCAACTTCAACAAGATCCAGAACGGTTTGAACGTGGCCATCGGCATTCTCAAC
CTCTCGTACGAGGGCAACATGGCCGATGAGACGCCCATCTTCATGCGCACAGTTGTTGGG
AAGCTCGGCTTCTTCCTCCAGCTCCTCAAGACTCCTCAGGACTCGGAGCGGTTCTTCCAG
ATGACCTACGGCAAGCTGAGCCCGCCCTTCGGCTTCCTGAGGGTGAAGATCCTCGAGTTC
CTCAGCGCTCTGTTGAACACGAGGTACCAGTCCGTGGTGGACGCCTTCTGGAAGACCGAG
CCCAACGTGTTCGAGACGCTGTTGGACATCCTCTTCCACTACAAGTGGAACAACACTGTG
CATTACTATATTGACCAAATCATCGCCACCATCATGTCGCTCGACAACGGAGACAACATC
ATCGCCGTCCTCATCGAAAAGTGCAAGTTCATCCAGCGGTTCTCCGACGCGTTCAAGAAG
GAGAGCGACAGGACCGTGGGCTACCTGGGCCACCTGGTCCACCTCTCGAACGAGCTCACC
AAGGCCTCCACCTACCACGAGCCCCTCGCCAGGCTCCTCGACGAGAACGACCTCTGGAAG
GAGCTCGTCAACGGCAGGCTCAAGGAGATCAACGACAGAAACAACACCCCCATCGGTGCC
CCTCCCGCCGGCGGCTCTCCCATGATGGGCGACTCTGGCCTGGGCGAGATGGGTCGTGGT
GACAGCGTTTTCTACGAGGAGCAGGGAGTACAATACGAGGAGGACGGCTCCGATGAGGAG
GTCGACGATGACGGAAGGATTGAGCTTATTGACGAAAGCGAGAGAGCGGAAGAGGTTGAG
CTCACGTCCGAGGAGCCCCTCCCAAGCGGCGAGGAAGCTGCGTCGAGCTAA
>g1185-932177-934308
ATGGCGAACACAAGCGGATTGTATGGCTATTATGACGTCAACCCGATGAGGAGGTCCATC
ATGACCTTCAACATCGATGACGGCTTTCCTGAGGCCATCGTTCGCGGCTATCGCTCCGGC
ATTCTCACCCCCGCCGACTACGCCAATCTTACCCAGTGCGAGAGCCTCGAGGACATGAAG
CTGCACTTGGCGTCCACGGACTACGGTGATTTCCTCCAGAATGAGCCCTCCCCCATTCAC
ACCACCACCATTGCCGAGAAGTGCACCCAGAAGCTCATCGAGGAGTTCCAGTACGTGCGG
GCCAACTGTTTCGAGCCCCTCAGCACTTTCCTCGACTACATCAGCTATGGCTACATGATC
GATAACGTCGTGCTTCTCATCACCGGCACCCTCCACGAGCGTGACCTCGGTGAGCTCCTT
GAGAAGTGCCACCCGCTCGGCATGTTCGAGTCCATCTCTTCCCTCTCGTCGGCCCGGTCC
GTCAATGACCTCTACGATTACGTGCTCGTCGATACCCCCCTCGCCCCCTACTTCCTGGGC
TGCCTGTCGCAGGAGGATCTGAACGAGATGAACATCGAGCTCATCCGTAACACACTGTAC
AAGGCCTACCTCCAGGACTTCTACAGGTACTGCCAGGTCCTCGGCGGCGACACTGCCGAG
GTGATGGGCGAGATCCTCCAGTTCGAGGCGGACAGGCGCGCCATCAACATCACCATCAAC
TCCTTCGGCACGGAGCTCAGGAAGGAGGAGAGGGAGGCGCTGTACCCCAACTTCGGTCTG
CTCTACCCGGAGGGCACCAAGAAGCTCGGCCAGGCCGACCGAGTCGACCAGGTCAAGGAC
ATTGTCGACACCTTCAGCAACTACGGCCGGCTGTTTGACGAGTCGGGCCACAACAGCGAC
AAGTCGCTCGAGGACGCCTTCTTCGCCTACGAGGTGAAGCTCAACCAGCTCTCCTTCGAC
AGGCAGATGCAGTACGGCGTCTTCTACGCCTACATCAAGCTCAAGGAGCAGGAGATCAGG
AACCTGGTGTGGATCGCCGAGTGCATTTCGCAGCAGCTCAAGTCCAAGATCAACAACTAC
ATCCCCATCTTCAAGGACTAA
>g1185-948715-950868
ATGGAAAAGGACGGCAAGAGCGGAGCGGGCAAAGAGATCGCGCAGGAGGGGCTGGCCGCC
GACGCCGAGGGCTACAGCCCGTACGTGAACAGCGAGCACGGTGTGTCGCTCGTGTATCCG
GAAGAATGGAAGAAGAGCGAGGGCTACATGGGCACGATCGTGTCCTTCCTCTGCCCTGAC
GACGCCACGGGCCGCACGTCGCTCAATCTGCTGGTGCAGGAGGTGCCGCCCGGTATCACC
CTCGAGGACTACTCGGCCGAATCGCTGGGCCAGATGAAGCAGATGATGGCCGACACGGAG
TTCAAGGTGTGGGACGGCGACATGTTCGCGCAGGCGGGCAAGCGCATGCAATTCGTGGCG
GACATGGGTCCCTTCACCGTCAAGGTCTTCCAGGCCTGGACGGTGGCCAAGGACCAGGCC
TACATCCTCACCTACTCGGCGCCCGCCGACACGCACGACCTGCACCGCGAGCACGTCGAG
AAGGTGATCGCCTCCACCAAGTTCGTCAAGGCCGAGCCCAAGGAGAAGCCCATCGAGCTC
CTCTGCCTCAAGCACTACGAGAACCCCACCCACGCCTTCCGCCTCCGGTTCCCGCGCACG
TGGACGGTGAAGGAGCCCGAGCGCGAGGGCGGCCCCGTGGTGCAGGCCAAGTACGAGGAC
GCCCTCGCCGTCCTCGACACCCAGACCTACACCCTGACCGCTGACGTGACGATTTCGACG
CTGCCCAACGAGTCGTGGGGCGTGGAGGAGTACAAGGACATCGCGCTCATGCGGCTGGGC
GATCTGGCGGGCGGAGAGGAGAACATCCAGCTCAAGGACACCAAGATGGGCGGCCAGCCG
GCCATCCAAGCCCGCTACGCCTCGCCCTCTGCTGGCACCACCAACCTTCACGTCTTCACC
GTCAAGGACGGCAAGGCCTACAACATTGCCTTTTCCACCAACACGCCCAAGAACGACACC
CGTCCGTACCCCATCTTCGCGCGCATCCTGAGCTCCTTCACCATCTTGTCGCCCGGGTAC
ACGCCCAAGGCCAAGATCCTGGCCCTGGAGCACCTCGTCCACAAGGTCTCGTTCGAGTTC
CCCGAAGACGAGTGGCTGCCGAGGGAGGGCATGATGATGACCACGGTGTCCTTTGAGCAC
GGCATGACCGACGAAGGCGCGCCTCGTTCGAACGTCAACCTCGTGATCACGGACCTCAAC
ACTGCGCCCATCAACGTTAGCTCGCTCGACGAATTCGCCAGCATTGTGCGCGAGCAGCTC
GCCATGGCCGTCGACTCGCTCAGCTTCGTGCACGAGAAGAAAGTCAAGATGGGCGGCAAG
GACGCTCGCGAGCTGCGCTACAAGGGCACCATCTCCGACAGGGAGTTCGGCTTCCAGCAA
ACGTTCACAGTCCTCGACAACAACGCCTACGTCATCTCCTTCTCTTCGCTCCTCGGCGAC
TTTGACACCGAGATCGAGCTTGCCCTTCCCATCATCGAGTCCTTCCACTTCCTGTGA
>g1185-962496-964078
ATGAGAAGAACGATCGCCACCCACTGCAACCTGGCCGCCCTCCCCATGCGCCGCGGCTTT
GCCTCCGCTGTCCCTGCGGCACGCCGCCTCGAAGGCAAGGTGGCCATCATCACTGGCGCC
GCCAACGGCATCGGTCGCGAGGCCAGCCTCATTTTTGCGCGCCAGGGCGCCAAGATCGTG
GCCGTCGACCTCGGCGATGCGCGCGAGACCGTCGAGGGAGTGAAGGCCGAGGGCGGCGAG
GCCGTGTACGTCAAGGCCGACGTGTCGAAGGCGGGCGACTGCGAGAACATGGTGCAGGTG
GCCGAGAAGACCTTCGGCAAGGTCAACATCCTCTTCAACAACGCCGGCATTATGCACGGC
GACGACAATGGCGCCACCGACACCGAGGAGAAGATCTGGGACCTCACCATGAACGTCAAC
GCCAAGGGCGTGTTCTTCGGCTGCAAGTACGGCATCCCCGCTCTCCGTCGGGCCGGCGGC
GGTTCCATCATCAACACGGCCTCGTTCGTGGCCATCCTCGGGGCTGCCACCTCGCAAATC
GCGTACACGGCGAGCAAGGGAGCCGTTCTTTCCATGAGCAGGGAGCTGGCGGTGATCCAC
GCCCGTGAAAACATCCGCGTCAACGCGCTCTGCCCTGGTCCGCTCAAGACGGAGCTGTTG
ATGTCCTTCCTCAACACCGACGAGAAGAAGCAGCGTCGCCTTGTCCACGTGCCAATGGGC
CGCTTCGGCGAGGCACAGGAAATCGCCCAGAGTGCCCTCTTCCTGGCCTCCGACGAGTCG
TCCTACATCACGGGCTCCACCTTCCTTGTGGATGGTGGCATCACGGCCGCCTATGTCACC
CCCCAGTAG
>g1185-1086077-1087318
ATGATGACGCGAAGGACCACTTTTGTGCTCGTGGTGCTGGTCATTGGCCTGGTGCTGGGC
GTGCATGGAATCAAATTCGACATCAGCCCTAACACGGAGCGTTGCCTCAAGGAAGAGCTC
AGCAGGGATGTGCTCGTACTGGGTAACTACTCAGTCGAACAGCAGCAGCCCTCACTGCGC
CTCCACTTCACCATTATGGATCCGAGCAAGGGCGAGATCTTTGCGAAGGATGCTGATGAG
GCGCACTTCTCGTTCACTACCGAGAATGGAGGGGAGTTCCTGTTCTGCTTCAACGACATC
ACCGATCAGAGCCGACTGGCCTACGGTTACTCCAGGAGGGTAGCTCTTGAGCTGAAGACC
GGTGTCGATGCCAAGGACTACACCGAGCTGGCTGGCAAGGAAAACTTGTCCAAGCTCGAG
GTCGAGATGAGGCGAATTGAGGATGGCGTCGCTGAGATCCTCAACGACCTCAAGTACCTG
AGGAGGAGGGAGGAGAGGATGCGTGACACCAACGAGTCGACCAACAGCCGGGTGGCGTGG
TTGTCCATCTTCTCGGTGGTCACGATGGTGGCCGTGGGTGCGTGGCAGATCTACTACCTC
AAGCGCTACTTCCAGCAGAAGAAGATCCTCTAA
>g1185-1095716-1097600
ATGCGCAGAATCACCGCTCGCTCTGTCACTACGCCCCTCTTCACATCGAAGACTGCTTCG
CTCGCCGGCTCCGCCCGCCCCCTCTTCGGCATCGTTTCTGCGGCCGACAAGGTCGTTCCC
AAGGATGGCCTCGTGACCGTGGAGGTGTACCGCTGGTCCGCCGATGAGAAGGAGAAGCCC
TACATCCAGAGCTACAAGCTGGACGTCAACAACTGCGCGCCCATGGTGCTCGACGCGCTG
CTCAAGATCAAGAACGAGCAGGACCCCACCCTCTCCTTCAGGAGGTCGTGCCGCGAGGGC
ATCTGCGGCAGCTGCGCGATGAACATCGACGGCACCAACACGCTGGCCTGCCTCAAGCCC
GTCGCCGAGTCCGTCACCAACGGCAAGATCAAGATCTACCCGCTCCCCCACCTTCCGGTG
GTGAGGGATCTGGTGCCGGACCTGAGCAACTTCTACGAGCAGCACAAGTCGATCCAGCCG
TACCTGCAGGTGGACGACCACGCCGCCCTGGGCTCGAGCAAGACGGAGCAGCTGCAGACG
CGGGAGGACAGGAAGAAGCTCGACGGCCTCTACGAGTGCATCCTGTGCGCGTGCTGCTCC
ACGTCGTGCCCCTCCTACTGGTGGGCCGGCAACGAGAACAAGTACCTCGGCCCCGCCATC
CTCCTCCAGGCCTACAGGTGGATCGCCGACTCCCGCGACCAGCACACCAACCAGAGGCTC
AGGGCTCTGGCCAAGGAGGACATGAAGGTGTACGCGTGTCACACCATCATGAACTGTACA
AAGGTGTGCCCCAAGGGGCTCAACCCGGCCCTCGCCGTCGCCAAGGTCAAGAAGGCCACC
CTCGAGCTCGACGTGCAGCAGAAGAAGCAGGCCCTCGCCCACTAA
>g1185-1175416-1176770
ATGATAAACGAGATTAGGGCAGCGGTGGAGTGGCTGACGCGCACCATGGAGGTCAACGAC
CTGAGCGAGGCCCAGCTCCAAAGCTTCGAGTGGGCTCTGGTCGAGGCCCTCACACGCAAG
TACACCGACCACTGGTACCAGGATATGCCGTGGCGAGGTCAGGGCTATCGCTCGATTCTG
TCCGAGGACTGCGCGCACGTCGTCGATCACGTCCTGCGCCATGCCGCGCGCGAGGCCCAC
ATCACCGACCTGCCCTACAGGATGCCTGCCGAGGCCTTCGTGATCTGGGTCGATCCTGGC
GATGTCGAAGTCAAATTCCTCAAATCGCATCGAACCGAGGTGCTCTACCACCAGCCCTCC
TACACGATGGTGCCCGATTCGAGCGCTGCGAGCCACAGACGCAGCATGGCTGCCACGTCC
TCGTCGAGCACGCGCAACAGCTACGGCCATGGCCGCGTTCGGTCAATCGAGGAGGTGGAC
GTCGATGGCGCTTCATCTCTGCTCATGCCCGACGATGACCGCAGCATGCGCTACAAGAAC
GGGCTGCAACAGCAGCACGACTACCTGAACGCCTCGGACAGCGCCGTCTTCGTCCAACGC
GGCCATGCCAGCCCGCCTCGCGCCTCGACTGGCGCGTGGAACGCTGGCAGCGGCACCTAC
ACCAGCAAGTCGCCATCGAAGTTCAACCTCAACGCTCGCCCCTGGGAGCGGCCTTCGAAT
GGACCTTCATCTTGGGGCGCCAAGCCTGGCTTTCGCTCACCGAGCCCAGTCGGCCTGGCT
GGCGTCGTGTAA
>g1185-1260978-1262560
ATGTTCTACAAGTACGGCCGCATTATCCGCTGTGATGTCAAGGTCGGTTTCGGATTCGTA
GAGTACGAGGACCGACGTGATGCCGAAGATGCCGTCCGCGATCTGGATGGTGCTCACCTT
ATGGGCAAGCGCATCGCTGTCGAGTGGGCCAAGGGTGAACGGAGGGCTACGGGCACGCGC
AGCGATGCCTGCTTCCGTTGCGGAGAGGAGGGTCACTGGGCCAGGGACTGCACCCGCGGT
GGCGGTGGCGGTTACGGTCCCGGCGGCGGCGGCGGCGGCGGCTACGGCGGTCGTGGTCGT
TCTCCCCCTCGCTACGGCCGTGGATACAGCCGCAGCCCTTCGCCCCGTCGTGGACGCTAC
AGCCCTTCGCCCAGGCGCGGTCGCAGCCCCTCGCCCAGGCGTGGACGCAGCCCGTCCCCC
AGGCGTGGTGGTCGCTACTCGCCCTCGCCCAGGCGCGGAGGCCGCAGCCCTTCGCCCAGG
CGCAGCCCCTCGCCCCGACGAGGCCGATCGCCTTCGCCGCGCAGCCCCGCCGAGAAGAGG
AAGCTCTCCCGCACGCCCTCGCCGGGCGGCAGGCGTTCGCCGTCGCCTCGCCGCGGCAGC
CCGTCGCCGCGTCGCAGCCCCTCGCCCAGCAGGGGAAGCCCGTCGCCCAGGAGGAGCCCC
TCGCCCAAGCGCAGGAGGACGAGCGGCTCGCGCAGCCCGAGCCCCGCCACCAAGCGCGGC
AGCCGCTCGCCCTCGCCCTCGCCCGCCCCGGCCAACGGCGACGTCCCCGCCGATAGGGAA
GAGTAA
>g1185-1420566-1421308
ATGGTCGATAAGGTAGCGCTCGCTTCGCAGTACATCAAGGCTCGCCTTGCTGAGGACAAC
GACACCGTCCTCTCGCTCGTTACGGATGACATTGTCCTCGTCAGCCAGAGGGATGGCACC
CATGAGGGCAAGGAGGCTTTCACCAAGTACCTCAAGTCGGTGAAGCCCACTGGCAAGTGG
CAGGACCCCGTCCTGGAGGGCGACCAGGTCGTCATCAAGGGCACCGTCACCGTCATATTC
ATCAACTGGAGCGTGGCCTCTTTCCTCGACTTCAACGACGACGGCAAGATCACCAAGATC
GAGATCAAGAGGGTGTAA
>g1185-1429926-1432860
ATGGAGAGCACGAACATGGCTACCAACAAGTCTGGAAGTGTGGAGTCCATCCGCGCGCAG
CAGGTCATCGCCATCATGGGCGCTCAGTGGGGCGATGAGGGCAAGGGCAAGCTCGTCGAC
GTCCTCGGTGCAGACTACGACATCATTGCCCGCTGCGCCGGAGGTTCCAACGCCGGCCAC
ACGGTGATCGTCGAGGGCAAGAAGTTCGCCTTCCACCTGATGCCCTCTGGTATCCTGCAC
GAGAGCGTCATCTGCGTGCTGGGCAACGGCGTGGTCATCCATGTGCCCACCTTCTTCGAC
GAGGTCAAGTCCCTCGAGGACCAGGGCATCCCCTACACCGGCAGGATCAAGATCTCCGAC
CGGGCGCACCTGGTGTTCGATTTCCACCAGGAGCTCGACGGCAAGCTCGAGGACGAGGCC
AAGCGCGGTACCAAGGACCTCTCCATCGGTACCACCAAGAAGGGCATCGGACCGTGCTAC
ACCGCCAAGATCGCCCGCATGGGTATCCGCGTGGGCGACCTGGCCTTCCCGGCCAAGTTC
GAGGAGAAGCTGACGGCCCTCGTCAACTACTACAAGCGGATCTTCCCCGACATGGCCCCC
GTCGACATCCCCAAGGAGGTCGCCAAGTACACGCAGGACTACTACGGCAGGATCAAGCCG
CTGATCGTGGACACCGTGCACTACCTCAGCCAGGCCATCAAGACCGGCAAGCGCGTGATG
GTCGAAGGTGCAAACGCGACCATGCTCGACATCGACTTCGGTACCTACCCCTACGTGACG
TCGTCCAACGCTTCCATCGGTGGCGCTTGCACCGGCCTCGGCCTGGCGCCCACCAAGATC
GGCGCCACCGTCGGTATCATCAAGGCCTACACGACCCGTGTGGGCGCTGGTCCGTTCCCC
ACAGAGCTGAATGACGACCTCGGCGAGAAGCTGCGCGCCGAGGGACACGAGTTCGGCACC
ACCACTGGTCGTCCCCGCCGATGCGGTTGGCTGGACATTGTGGCCATGCAGTACGCGCAG
GCCATCAACGACTTCACCAGCCTCAACCTCACCAAGCTCGACGTGCTTTCCTCGTTCGAC
GAGGTCAAGCTCGGCGTGGCCTACAAGGTTGACGGCGAGGTCCTCCCGTTCTTCCCCGCC
AGCCTCGAGGTGCTGGGCAAGGTCGAGGTCGTGTACGAGACCTACCCCGGCTGGAAGTGC
GACATCACCAAGGCCAAGTCCTTCGAGGAGTTGCCCAAGGAAGCTCAGACCTACGTCAGA
CGTATTGAGGAGCTTCTCGAGTGCCCCATCGCCTGGATCGGCGTGGGTCCCGGCCGTGAG
GCGATGGCGATCAAGACGCCCCAGTGA
>g1185-1434216-1436442
ATGGTAGTACTCAAGGTCACGTACGGTTCCGAGATCAGGAGGATCACGGTCGACGACACC
CAGGCCTTCTCCTACAAGGACCTGCGCGTCCTCCTGAAGAAGCTCCATCACAACACTCTC
CCGAACCACTTCGAGATCAAGTACCTCGATGATGAGAACGACAAGGTGACCATCTCCAGC
GATCGCGAGCTCGCCGATGCCCTCCAATTTGTCAAGACGGCCAAGCAGCCTCTCCTCAGG
ATCATGCTCTCGGATCCAGTCAAGAAGGCCGCCCCCGCCCCTGCGCCCGCCCCGGCTCCC
GCTGCCGAAGCTCCCGCTGGCAAGGAGAAGGAGTCCCAGCAGGCTACCGGTGCCTTCAGC
CCCACGCAGCTGTTGGAGGTGATCGAGGCCGCCCTCAGCTCGCCCGAGGTCCAGCAGACC
TGGGAGAACGTCCAGGACACCTTCACCACCTGCGTCGACCAGGCCGTGCAGGAGGGCCGC
ACCGCCGCGCACTACGTCCAGCAGCACCCGCAGTTCTCGCGCGTTCAGCACCTCCTGACC
GAGCGCCTGCCGCAGGCCATCCACAACGACTTCAACACCACCCTCCCGCGGCTCATCAAC
GACATTCAGGCCATCTTTGCCAACATCCAGATCCCCATCGCGCCGGCCGCCCCCGCGCCT
CCCACCACGCCTCAGGTGCACCACCACGCCATCTGCGACGCGTGCGAGAGCCGCATCGTG
GGCATCCGCTACAAGTGCACGAGCTGCCCCGACTACGACCTGTGCGAGGCGTGTGAGGCC
AAGACCCCGGCCGTGCACGACTCGACCCACTACTTCATCAAGCTGCGCACCCAGCTGCCG
TGGGGCATGGAGCACGTCCGTCTCGCCTCGTCGCCCACCAACACCAGCCCCCTCGGCCGT
GCCGCGCGCTGCCCCTACTTCCAGGTGCCCCAGCAGCGCCACCAGACCTTCCAGCAGCAG
CAGCAGCAGCCGCAGCCCCAGAGCGATCCGCAGCACCACCAGGGTCCTCGTGTGCGCCGC
CACCCGTGCCGCTTCATGCTGTCAGCCAGCTTCGTCTCGGACGTGAGCATCGATGACGGC
ACCGTGCTGTCGCCCAACACGCCATTCGTCAAGGTGTGGCGTCTGCGGAACGACGGCGAG
CGCGCCTGGCCCGAAGGCACCACGCTCACCCACGTCTTTGGCCCGCGCCTGTCCGACGTC
CAGTCTGTCTCCGTGCCCGCCCTGCCCGCCGGCGAGGAGGTCGACGTGGCCGTCAACATG
GTCAGCCCCGCCGACGCCGGCCACTACGTCAGCAACTGGCGCCTGACCGGCCCGCGCGGC
TACAAGTTCGGCCACCGCGTGTGGGCCGACATCACCGTCCAGCCCGCCACCACCGTCACC
AACTCCGTTCCCGCCGCCGCCGAGCAGCCGCAGGAGGAGTCCAAGGTCGAGGAGGCCGAG
CAGCAGACCACCACCTCGACCCCGACCGTCGAAGAGCCCGCCTCGTCCGATAGCGAGTGG
GAACAGATCATCGAAGAAGAGGAGGAGAAGAAGGTGGAGGAGGAGGCCGTCGTCGAGCCG
GTCGAGGAGCCCAAGGTGGTGCAGATCGTGATTGAGGATGCCCCAGTCCCCGTCGTCGCT
GCTCCGGCCGCCACGCCTGCTCCCGAGCCGGCCCAGGCCGCTCCTGCTCCTGCTGACGAG
GACGACGGTGTGAGGATCGTGCTCGATCAGCTCGCGCAGATGGGCTTCCACGATCGCGCG
CTCAACAAGAGGATGCTGGCCAAGAACAGGGGCAACGTGCTCGCCACCATCCACAAGCTC
CTCGACATGTAA
>g1185-1473757-1474488
ATGTCGTTCACTCCCCCCGCTGGTATGGACAAGGCCTGCATCCCCGGTGATGTGGACAAC
GGTGAGAAGCTCTTCAAGGCTCGCTGCACCCAGTGCCACACCATCAACAAGGGTGGCGGA
AACAAGCAGGGTCCCAACCTGTACGGTCTGATCGGCCGCAAGTCTGGCATGGTTCCCGAC
TACAAGTACTCGCCCGCCAACAAGAACGCCGGCGTCACGTGGACCAACGACACTCTCTAC
AACTACCTTGAGAACCCCAAGAAGTACATCCCCAAGACCAAGATGGCCTTCGCTGGTTTC
AAGGCCCCCCAGGACCGTGCCGACGTCACCGCCTACATCGTCAAGGCCGCGCAGGAGTAA
>g1185-1530393-1534204
ATGGGAGAGCTCTTCCGTTCGGAGCCTATGCAGCTCGTCCAGCTGTTCATGTCGCTCGAG
GCAGCGCGTGACACCGTGGACGAGCTCGGTGAGATCGGTCTCATCCAATTCAAGGATCTC
AATCCTGAGGTAAATGCCATCCAGCGTAACTTCGTGGCCGAGGTCAAGCGCTGCGACGAG
ATGGAGCGCAAGCTCCGCTTCTTCGAGGACCAGATTGAGAAGCAGAACTTCGCCGAGGAG
GAGCTTGAACACCTCCAGCTCGGGCTCAGCATCGGCTCGTCGAAGAAGACTCTCGTCCCC
GAGATGGACGAGCTTGAGGCCCGCTTCGAGGACCTCGAGAAGGAGCTTACTCAGATGAAC
AGCAACCAGGAGAAGCTGAAGCGCAACTACAACGAGCTCATTGAGCTCAAGCACGTGCTC
GAGAAGGACAGCGTGTTCTTCGAGTCGTCCGGCGGTGCCGAGAGGGACCGCTACGACGAG
GAGGCCGACGTCGGCAGCAGCGAGGTGGCCGGCCTTACCTCGTTCGGCGTCAAGCTGGGC
TTCGTCACCGGTGTCGTCGAGCGTTCCAAGATGGTCACCTTCGAGAGGGTGCTCTGGCGC
GCCACCCGCGGTAACTTGTTCATGAGGACTGCCCCCATCGAGGAGCGCATTGAGGATCCC
AAGACCAACGAGTTGGTGGACAAGCTTGTCTTCATCATCTTCTTCCAGGGTGACCGTGCC
GAGTCCAAGGTCAAGAAGATCTGCGAGTCCTTCGGCGCCAACCTCTACCCGTGCCCCGAC
AGCGCGCAGGAGAGGCGTGAGATGTTCAACCAGGTGGAGACCCGCCTCGACGACCTCGAC
GTCGTGCTCGAGAGGTCGCTCGACCACAGGAAGAAGGTCCTCCTCGACATCGCCACCCAC
ATCGAGGACTGGAAGACCCAGGTCGTCAAGGAGAAGTCCATCTACCACAACATGAACCTC
TTCAACTACGATGTCGGCCGCAAGTGCCTCATCGCCGAGGGCTGGTGCCCGCTCACCGCC
ACCGAGGACATCCAGGACGCCCTCAAGCGCGCCAACGAGCGCTCCGGCACCCTCGTCCCC
TCCATCGTCAACGTCGTCAAGACCAGGGAGCAGCCGCCCACCCACTTCAAGACGAATAAG
TTCACCAAGTCCTTCCAGGGCATCGTCGATGCCTACGGTATGGCGCGCTACAGGGAGGTC
AACCCTGGTGTGTTCACCATCGTGACCTTCCCCTTCCTGTTCGGTATGATGTTCGGTGAT
GTCGGCCACGGTATCATGCTCTTCATCTTCGCCGTGTACCTGTGCATCAAGGAGGACACC
TTCTCCAAGATGAAGCTCAACGAGATGGTCAAGACTTGCTTCGACGGTCGCTACCTGCTG
CTCCTCATGGCCCTCGGTGCCATCTACTGCGGTGCTCTCTACAACGAAGTCTTCTCCGTC
CCGCTCGACATCTTCGGCTCGCGCTGGCAGTACTTCGAGGGCGAGCAGTTCGCCGAGTGG
ACCAACCCCGGTATCGCCTACCCGTTCGGTGTCGATCCCGCGTGGAAGGGTGCCAAGAAC
GAGCTGCTCTACTACAACTCGATCAAGATGAAGCTGTCCATCATCTTCGGTGTCACCCAC
ATGGTCTTCGGTATCCTCCTCTCCGCGCTCAACGGCATCTACTTCAAGAAGCCCTACAAC
ATCTGGTTCGAGTTCGTGCCCCAGCTTTGCTTCATGATGTCCATCTTCGGCTACATGGTG
TTCCTCATCTTCTTCAAGTGGAGCTACGAGTTCTCGGCGCCCCAGGACGCCCCCAACTTG
CTCAACCTCATGATCAGCATGTTCCTCAAGCCCTTCAAGCTCCAGCCCATCGACGATCTC
TTCCCCGGCCAGCTCTACCTCCAGTGGGTCCTGATCGCCGTCTGCGCCATCTCCGTCCCC
ATGATGCTCCTCCCCAAGCCCCTGCTCCTCAGGCGCGACCACAAGCGCGGCTACAAGCGC
CTTGCCGAGTCTCACGAGGAGGACGGTGATGAGGAGGAAGAGGAGTTCGACTTCAACGAG
ATTTTCATCCACCAGATCATCCACACCATCGAGTTCGTGCTCGGAGCCATCTCCAACACC
GCCTCGTACCTGCGTCTGTGGGCCTTGTCGCTCGCCCACTCCGAGCTCGCCACCGTGTTC
TGGGAGCGCGTGCTCGTCCTCACCCTCGAGAAGAACAACTTCTTCCTCATCTTCGTCGGC
TTCGCCATCTGGGCCGGCGCCACCTTCGGTGTGCTCTTGGTTATGGAGTCGCTCTCTGCC
TTCCTCCACGCCCTCCGTCTCCACTGGGTCGAGTTCCAGAACAAGTTCTACATGGGTGAT
GGCTACAAGTTCCAGCCGTTCTCCTACCAGCAGATCCTCTCCGGCGAGGAGGAGGGAGGC
CTCTAA
>g1185-1546317-1547604
ATGGTGTCCCTCAAGCTTCAGAAGCGCCTCGCGGCCAGCGTGATGAAATGTGGCCAGAAG
AAGATCTGGCTCGACCCGAATGAGGTCAGCGAGATCTCTATGGCGAACTCTCGCCAGGCT
GTCCGTAAGCTCGTCAAGGATGGCTACATCATCCGAAAGCCCCCCGTGATGCACTCGCGC
GCTCGCGTAAGGAAGAACCTCCTGGCCAAGCGCAAGGGTAGGCACATGGGTGTCGGTAAG
AGGAGGGGTACCCGTAACGCCCGTATGCCCGAGAAGGTCATCTGGATCAGGCGCATGCGT
GTGCTCAGGAGGCTTCTCCGCAAGTACCGTGAGGCCAAGAAGATCGACAAGCACTTGTAC
CACGAGCTCTACATGAAGGTCAAGGGTAACGTCTTCAAGAACAAGCGTGTGCTCATGGAG
TACATCTGGAAGGCCAAGGCTCAGAAGATCAAGGACAAGGCCGAGCAGGAGCAGGGTGAG
CTCAGGCGTCAGAAGAACCGTCAGATGAGGGAGCGTCGCGCCAAGCGCCTCGACGAGAGG
AGGAAGGTCCTCCTCGGCGAGGACAAGAAGGCCGTCAAGAAGGAGGAGACCGGCAAGAAG
GCCGCCGCCCCCGCTGCCGCTGCCGCCCCCAAGGTCAGCAAGAACCAGAAGAAGAAGGAG
GCTGCCAAGGCCGCCGCCGCTGCCGCCCCCGCCAAGGCTGCCGCTCCCGCCAAGGCCGCC
GCCCCCAAGAAGGAGGAGACCAAGGCCGCCGCCCCCAAGGCTGCTGCTCCCGCCAAGGCC
GCCGCCCCCAAGAAGGAGGAGACCAAGGCTGCCGCCCCCAAGGCCGCTGCTGCCAAGCCC
GCCGCCAAGGCTGCCGCTCCCAAGAAGGAGGCTGCCAAGCCCGCCGCCGCCAAGCCCGCT
GCCAAGGGCGGCAAGAAGGGTGGCAAGTAA
>g1185-1547702-1549236
ATGTCACACGGACACGGCGGAAAGGCTGCGGAGGGACCGGCCACCGAATATCTCTACAAG
ATCCTCGTGGTGGGCGACATTGGAACGGGTAAGACCGCCATCATTCGGAGGTGCGTCGAG
AACCAGTTCTCTGAGTCCTACAAGACCACAATTGGCGTCGATTTTGCCTTGAAGACCATC
CAGCGTTCCAATGCCACCATCCACCTCCAGCTGTGGGATATTGCTGGTCAGGAGCGTTAT
GGCAACCTCACCAGGGTGTACTACAAAGAGGCTGTTGGCGCCTTCGTCGTGTTTGATCTC
ACCCGCAACTCGTCGTTCGAAGCTGTCAAGAGATGGAAGGAGGACATTGACAACAAGGTT
AGACTGCCCAACGGCGATCCTCTCCCCGTTGTGCTGTTGGCCAACAAGTGCGATCTTGTC
AAGACGCCGCTTAGCGAGGAGGTGATGAACGATTACTGCAAGGAGAATGGATTCCTCGCG
TGGTTCGCCACCTCCGCCAAGGAGAACATCAACATCGACGAAGCTGTCAATTTCCTCGTC
GAAAACATCATCGTTCATGGCAGCAACGATGCCATCAAGGGACCTGGCCCCGACGCCCTC
AAGGTCGGCGGAGAGCGTGGCGGTGCCCCGGACTCCAACGCCTGCGGCTGCTAG
>g1185-1652152-1653521
ATGTCGTACGATCGTGCCATCACCGTCTTCTCGCCCGATGGACACTTGTTCCAGGTCGAG
TATGCTCTCGAGGCAGTCAGAAAGGGAACCACTGCTGTGGGCGTGAGGGGCAAGTCCATC
CTCGTGCTGGGTGTGGAGAAGAAGTCTGTGCCCAAGCTGCAGGATCCCCGCACGGTGCGC
AAGATCGTGCAGCTAGATGATCACATCTCGCTCGCCTTTGCCGGATTGACGGCCGATGCA
CGCGTCCTCATCAACAAGGCACGACTCGAGTGCCAGAGCTACTCGCTCACCATCGAAGAG
CCCGTGTCCGTCGAGTACGTCGCCAAGTACATCGCTGGCGTTCAGCAGAAATACACACAA
AGCGGTGGCGTGCGACCATTCGGTATCTCCACGCTCGTGATCGGTTTCGACGACGGCAAG
CCACGCCTCTTCCAGACCGATCCCTCTGGCACTCACGCCGAGTGGAAGGCCAACGCTACT
GGTCGTAACTCCAAGACGGTGAGAGAGTTCTTGGAGAAGAACTATCAGGATGGCATGAGC
GATGATGAAGCGATCAAGCTTGCCATCAAGGCCCTGCTCGAGACTGTCGAGGGAACGAGC
AAGAACCTGGACATTGCGGTGATGCGTTCCGATAAGAAGCTGACCTTCTTGCCCGATGAG
GAGGTGGACAAGGTCGTGAAGGTCATCGAGGAAGAGAAGGAAGAGCAGAAGGCCAAGCCC
AAGCAGCAGCAGTGA
>g1185-1665893-1666734
ATGGGTCGTATGCATGGACCTGGTAAGGGTATCTCGGGCTCCGCCCAGCCCTTCAAGCGC
ACTGCCCCCTCGTGGCTGAAGACCACCACCCCCGAGGTGACCGATCTGATCTGCAAGCTC
GCCAAGAAGGGTCTCACCCCCTCGCAGATCGGCGTCATCCTCCGTGACTCGCACGGTATC
GGTAAGGTCAAGTTCGTCACCGGCAGCAAGGTGCTCCGCATCCTGAAGGCCAACGGTCTT
GCCCCCGAGCTCCCCGAGGATCTTTACCACCTCGTCAAGAAGGCCGTGGCCGTGAGGAAG
CACCTCGAGAAGTTCAGGAAGGACAAGGATGCCAAGTTCAGGCTCATTCTCATCGAGTCG
CGTATCCACCGTCTCTCCCGCTACTACCGCCGCACGAAGCAGCTGCCCCCCAACTGGAAG
TACGAGTCTTCCACGGCCTCGGCCCTCGTCGCCTAA
>g1185-1715612-1717587
ATGGAAGCATCAACCCCTACCCCCGAGGTAACCACTGCAGTGGCCGCCGCTACCCTGCCG
GTCGTTGCTGCCCCCTCCGGCCGACACATGTTCTCTTCGCGATGGACCGTGCGGGACATC
TTGGGCCGCGAGGATGGCGGTGTGGGCCTGGTCGATCAGGAGGGCGTCGTTGCGGGCTGG
ATCAGGACCGTCCGATTCGGCAACAAGGGCAAGCTGGCCTTCATCAACCTCAACGATGGC
TCCCTCATCACCGGCCTCCAGGTCGTGTGCGACAGCAACAAGGAGGGCTTCGACCAGCTC
AAGGCCACTGACTGCTCCGCGGGCGCCTCCATCTGGATCTTTGGCAAGGTCGTCAAGAGC
CCCGCCAAGGGCCAGACCGTCGAATTCCTCGCCTCCAAGGTGGCCCTCGTCGGCGGATGC
GACCCCCTGACCTACCCCCTGGCCGGCAAGGGACACTCCGTCGAGTTCCTCCGCGAACAG
GCCCACCTCCGCGCTCGCACCAACACCTTCAGCGCTGTGGCGCGTGTGAGGAACGGTCTG
TCGTTCGCGACGCACCAGTTCTTCCAGTCGAAGGGCTTCCTGAACGTGCACACGCCGCTG
ATCACGTGCTCGGACTGCGAGGGCGCCGGTGAGATGTTCCAGGTGACGACGCTGCTCAAC
AACGTGAAGAAGCCGGAGGAGGTGGCGCGCACGCCGACGGGCGAGATCAACTACGCGGAG
GACTTCTTCGGCAAGCCGTCGTACCTGACCGTGTCGGGCCAGCTCAACGGCGAGTACTAC
GCGTGCGGGCTGGGCAACATCTACACGTTCGGCCCCACGTTCCGCGCGGAGAACTCGAAC
ACGCGGCGCCACCTGGCCGAGTTCTGGATGATCGAGCCGGAGATGGCCTTCTACGATCTG
GTGGACAACATGGACCTGGCCGAGGCCTACGTCAAGCACTGCATCCAGCACGTGCTCACC
ACCTACCCCGACGACATGAAGTTCCTCGACTCGGTCTACAAGGGCGAGGGCGGCAGCCTG
CTGGCCCGCCTGGAGCACGTCCTGGCGACACCCTTCACCCGCATCACCTACACCGAGGCC
ATCGAGGCCCTGCTCAAGAGCGGTGCCAAGTTCGAGGAGAAGGTCGAGTGGGGCATCGAC
ATGAGCTCCGAGCACGAGCGCTACCTGACCGAGAAGATCTACAACACGCCCGTCATCGTC
ACCGACTACCCCAAGGACTTCAAGGCCTTCTACATGCGCCTCAACGACGACGGCAAGACC
GTCGCCGCCATGGACGTGCTCATGCCCACCCTCGGCGAGCTCATCGGCGGGTCCCAGAGG
GAGGAGAGGCTCGACGTGCTCCGCCAGCGCTTCGCCGAGCACAAGCTCCCCGAGGAGCCC
TACAAGGCCTACCTCGACACCCGCCGCTTCGGCTCCGTGCCCCACTCCGGCTTCGGCCTC
GGCTTCGAGCGCCTCGTCCTCTTCGTCTCCGGCGTCGAGAACATCCGCGACGCCATCCCC
TTCCCTCGCTACCCCAAGCACGCCCAGTTCTAA
>g1185-1720182-1721257
ATGTCTGACGATGAGGGACCAGAGACGACGCCCGGCTACAAGATCTCCAAGAAGGTCGAC
ATGGGCACCATCCTCGAAATGGATAACGAAGACGAGTCTCTGAGGAAGTACAAGGAGGCC
CTCCTCGGCAAGGCTGCCCTCTCTGGCGCCGTTGCTCCCTCGGATGATCCTCGCCGCGTG
GTGATCACGCGCATGAAGGTCATCTGTAAAGAACGACCCAACGGCGACATTCTGTACGAC
TTCACGGAGAGGGGATCGGAACAGAAGCTGAAGGACCAGCCCTTCACGCTCAAGGAGAAG
TGCGAGTACAAAATCGAGGTTGCCTTCAGGGTCCAGCACGAAATTGTGGCCGGTCTGAAG
TTCATCAACTTGGTCTTCAGAAAGGGCGTACGAGTGGCCAAGGAGGAGGAGATGTTGGGC
TCGTTCCCCCCGCAGGGTGAGGCCCACGTCGTCGTGTTCCCTCGTCACGGCTGGGAGGAG
GCCCCCAGTGGCATGCTCGCCAGGGGCAACTACAAGGGCAAGCACAAGTTCGTCGACGAC
GACGGCCAGTGCCATCTCGAGTACGAGTACACCTTCGCCATCAAGAAGGATTGGGAGTAA
>g1185-1741072-1742366
ATGTCGCAGATTCAGAAGGTGCACCTCACGTACTTCCCCCTGCGCGGCGTTGTCGAGAAG
CTGCGTCTTGTGCTCGAGGCTGGCGGTATTCCCTACGAGGAGACCAGGCTGAGGCCGAAG
GAGTGGGGCGCCCACAAGAAGTCCGGCGGGTACCTGTTCGAGCAGCTGCCCAAGGCGACC
ATCACCTTCGAGGACGGCTCCACGTTCGACCTCTTCCAGAGCCTCACCATCTTCCGCTAC
CTGGCCCGCAAGGCCGGGCTGGCCGGGGAGGGCGAGCTGAACCAGGCGCGCGTGGAGATG
CTCATCGACGCCTCGGAGGACCTCAGGCAGAAGTTCGCCAAGGTGTGCTACTCGCCCGAC
TTTGAGAAGCTGCGCGGCCCCTACGTGGCCGAGACCATGCCCGTCGAGTTTGGCAAGATC
GAGAACATCCTCAAGAGCAACGGCACCGGCTTCCTCGTCGGCAGCTCGTTGACGGCTGCG
GATCTGTACCTGTTCGACGTGGTGGAGAACCACATCGTGCTGGCCACCGACCAGTTCCTG
GAGCCCTTCCCGCTGCTCAAGAAGCACCACGAGACCGTGGGCTCCCACGCCAAGATCCAC
GCCTACGTGGCCAGCGGCAAGCGCCCCCAGTTCCCCAACGGACCCACCGCCATCTTCGGC
GGCTACAAGGAGGAGGCCACCAACTGA
>g1185-1806178-1808434
ATGGGAGACATTGATGTAGATAGCATTATCGAAAGACTCTTGGAGGTCCGAGGAAGCCGA
CCTGGCAAGCAGGTGCAGCTGGGAGCCCAGGAAATTCGCAGCCTCTGCATGAAGGCGCGA
GAAATCTTCATCTCCCAGCCCATCCTCCTGGAACTCGAGGCCCCCATCAAAATTTGCGGC
GACATTCACGGCCAGTACTATGACCTGCTGCGTCTGTTCGAGTACGGTGGGTTCCCTCCC
GATGCCAACTACCTCTTCCTGGGCGACTACGTCGACAGGGGCAAGCAGAGCCTCGAAACC
ATCTGCCTCCTCCTCGCCTACAAAATCAAATACCCAGAGAACTTCTTCATCCTCAGGGGC
AACCACGAGTGCGCCTCCATCAACAGGATCTACGGTTTCTACGATGAATGCAAACGGCGG
TACAGCGTCAAACTATGGAAAACCTTCACAGAGTGCTTCAACTGCCTCCCCATCGCCGCC
ATCATAGACGAAAAGATATTCTGCATGCACGGAGGGCTCTCCCCCGATTTGAAGACTATG
GAGCAGATCCGTAGGATCGTCCGCCCCACGGACGTGCCAGACCAAGGTCTGCTGTGCGAT
TTGCTGTGGTCCGATCCCGACAAGCAGATTCAGGGCTGGGGGGAGAACGATCGTGGCGTT
TCGTTCACGTTCGGAGCGGACATTGTCACGAGCTTCCTCAAGAGACACGACCTCGACCTC
ATCTGCAGGGCTCACCAAGTGGTGGAAGACGGTTACGAATTTTTCGCGAAGCGCCAGCTC
GTCACCCTCTTCTCGGCGCCCAACTACTGTGGCGAGTTCGATAACGCCGGCGCCATGATG
AGTGTCGATGAGACCCTCATGTGCTCTTTCCAGATTCTCAAACCCGCCGACAACAACAAA
AACTGA
>g1185-1855544-1856934
ATGGCCACTCAGCAGAAGCAGCCCATCAAGCTCTACAGCCTTGAGTACTTCGCGGCGTGC
GGCCTCGGTGGTATCCTGTCGTGCGGTCTCACCCACACGGCTGTGACGCCGATCGATCTC
GTCAAGTGCAACGCTCAGGCCAACCCTGAGCACTTCAAGAACACCGTCCAGGGTTTCCGT
GCCATCTACTCGGGCTCCCTGACCAGCATCGGCTTCGGCTCGGGCGTGTCTGGCCTCCTC
AAGGGCTGGGGTCCCACCCTCTGGGGCTACTCCCTCCAGGGCCTCTTCAAGTTTGGTCTG
TATGAGGTGTTCAAGCACTACTTCGCCGAGGCTGTTGGCCCCGAGAACGCCTTCAAGTAC
CGCGATCTCGTCTACATGGGTGCCTCCGCCTCTGCCGAGTTCTTCGCCGACATTGCCCTC
TGCCCCTTCGAGGCCATCAAGGTTCGCATCCAGACCAGCCCGTCGTTCGCTCGCGGCATC
ATCGACGGCCTGCCCAAGTTCATCAAGGCCGAGGGCTTCGGCAACCTCTACGCCGGTCTC
GGTCCCCTCTGGGCTCGTCAGATCCCCTACACCATCATCAAGTTCGTGGCCTTCGAGAGG
ATCTGCGAGGCCATCTATGCCATGCTCCCCAAGAAGAAGGAGGACATGTCCAAGACTGAG
CAGATGGGCGTCATCTTCGCCGCTGGTTACACTGCTGGTATCATCTGCGGTGCCGTTTCG
CACCCTGCCGACACCATGGTCTCCAAGATCAACAAGATCAAGAGCTCTGGTTCGCTCGGC
GAGAAGATGAAGCTCATCTACAGCGGTACCCCCGAGGCTCCCGGCATCGGCTTCGCCGGC
CTCTGGAAGGGCTTCGGTCCCCGCGTGGTCATGATCGGTACCCTCACTGGTCTCCAGTGG
TTCATCTACGGTGCCTTCAAGGCCTACGTCGGTCTCCCCACCCCCGGCGCCTCCGCTCCC
GCCGGCGACAAGAAGCACTAG
>g1185-1867882-1870710
ATGTACTCCAGGCTGCTTTTCATCGCGCTGGCGCTCGCGCTCTGCGCCGTGCTCGCCTCC
GGTGAGCTGGCCAAGCTCAAGGTCCACAAGAACAACCACCGCGCCCAGCAGGAGCGCGTG
CTCAACCGCCTCGAGGAGGTCCTCAAGACCAACCCCAAGGCCCTGGCCTACCACTACACC
CAGCGCAAGGCCGAGCTCAAGAAGGTCGAGGCCATGAAGAAGGAGGTCTTCGGCGGCGGC
AAGGGCGTCGAGCCCATCTCCAACTTCCTGGACGCCCAGTACTACGGCGAGATCTCCATC
GGAAACCCGCCCCAGTACTTCAACGTCGTGCTCGACACCGGCTCGTCCAACCTGTGGGTC
CCCTCGATCCAGTGCCCCTGGTACGAGATCGCCTGCGATCTGCACCACAAGTACGATCAC
AGCAAGTCGTCGACCTACAAGGCCAACGGCACCAACTTCCAGATCCAGTACGGTTCGGGC
GCCATGTCCGGCTTCCTGTCGGCGGACAACGTCGTGATCGCCGGCCTCACCGCCAAGGGC
CAGCTCTTCGCCGAGGCCGTCGCCGAGCCCGGTCTTGCCTTCGTCGCTGCTCAGTTCGAC
GGTATCCTTGGTCTCGGCTTCGACACCATCTCCGTTGACGGCGTGCCGCCTGTGTGGTAC
ACGCTCTTGGCCCAGAGCCAGGTGGCCGAGCCCGTGTTCGCCTTCTGGCTCAACCGCGAC
CCGTCGGGCATCTCCGGCGGCGAGCTCGTGCTGGGTGGCGTCGACGAGAGCCACTACACC
GGCGACTTCACCTACACCCCGATCACCAAGGAGGGCTACTGGCAGTTCCTGGCCCACGAC
TTCCTCATCAACGGCAAGTCCATGGGCTTCTGCCCGGCTGGCGGCTGCAAGGCCATCGCC
GACACCGGCACTTCGCTCCTTGCTGGTCCGTCGAAGATCGTGGCCCAGATCAACAAGATG
ATCAACGCCACCGGCATCCTCGAGTCCGAGTGCGACATGCTGGTGAACCAGTACGCCGGC
CAGATCATCCAGTACATCCTCCAGGGCCTCCAGCCCGACCAGGTCTGCTCTGCCGTCAAC
CTGTGCCCCGGTGGCTCGTGCCAGCTCTGCAAGGTGCTGGTCTCCACCATCGACGCCATC
CTCGGCACCGATCCCTCGCAGCAGGAGATCGTCGCCCTCCTTAAGTACATCTGCACCTAC
CTGCCGTCGCCCCAGGGCGAGGCCACCGTGGACTGCAAGACCCTGCCCTCGCTCCCCACC
TTCGACGTGGTCATCCCCACCGCCAACGGTCCCAAGACCTTCACCCTCAAGCCCGAGGAC
TACATCCTCAAGCAAAGCATGGGTCCCGAGGAAACGTGCATCAGCGGCTTCATCGGCCTC
GACATCCCGGCCCCCTATGGTCCCCTCTGGATCATGGGCGACGTGTTCCTGGGTCCCTAC
TACACCAAGTTCGACTTTGGCAACAAGCAGCTCGGCTTTGCTGTCGCCAAGTAA
>g1191-34206-36170
ATGGGTAAGCTCACGATCAAATCCCCCAGCGGCAAGGTGCTCAAGGAACTCGAGGTCTCC
GAGTCGTCCACGGTGGCTCAGCTCAAGGCGGCCTACGCCAAGGCCTTCCCCAAGTACTAT
CCCGACCGGCAGAGGTTCTACCTGCAGGATGGAGAGACGAGGGTCAGCCTGGAGGATGAG
AAGAAGCTTTCGACCTACAACCTGCTTTGCAGCAAGAGGGATGACGTCGTTTACTTCCGC
GATCTTGGTCCTCAGATTGCCTGGAAGACGGTGTTCTTGGTTGAGTACGCTGGTCCGCTG
CTCATCTACCTGTTCTTCTACGCCCGCCCTCCGTTCATCTACCCGGCCTCAGACGCGCCG
CACACGTGGGTGCAGAACCTCGCTCTCCTCTGCTGGGCGGGTCACTACCTGAAGCGCGAG
CTCGAGACCATCTTCGTGCACCGCTTCAGCCACGGCACCATGCCGATCATGAACATCTTC
AAGAACTCGGGCTACTACTGGGGCTTTGGCGCTCTCTGCGGCTACTTCGTGAACCACCCC
TACTTCACCAACCCCGAGCCCACTCAGGTCTACACTGCCCTCGCGTTCTTCGTTCTTTTC
GAGCTTGGCAACCTCATCTCGCACATCCAGCTGCGCAACTTGCGTCCGGAGGGTACTACC
GTCCGTAGGATCCCCCGCGGCTTCCTCTTCGAGTTGGTGTCGTGCCCCAACTACACTTGC
GAGATCCTCGCCTGGGTGTGCTTCTCGCTCATGACCCAGAGCGTTGCTGCTTTCCTGTTC
ACGCTGGTCGGTGGCGGTCAGATGCTGGTCTGGGCTCAGCAGAAGCACAGGCGCTACAAG
AAGGAGTTCCCGGGCTACCCCAAGAACCGCAGGATTCTGTTCCCCTTCCTCTACTAA
>g1200-8476-9536
ATGACGTCGCACTCCCTCTCCAACTTCAACAAGGCCCCCATCCAGAAGTTTGGCACCCAG
GCTACAGCGCCCCCGAAGGTGAAGATCTTCAGGTGCGGCGAAAAGCATGACAAGGGGCAG
GACTTTCTTGTGAAGCACTTCCGCACTCTCGACCAGTTCCACAAGCACGTGGCTGAAAAA
CTCAGCCTGCTGCCCGCGGTGCGCGCACTCTACACGGTGGAGGGAAGGAAGATAGGCAGC
CTGGAGGAGATCGAAGACAAGCAGGTCCTTGTCGCCGTCAAGACAGGCACCACCTTCACC
AAGGACAGGCTGCCGCTGGCCATCCAGCCCCCAGCGCCCGTCGAGTGA
>g1229-35010-35758
ATGGAAAAGGCCAAGGACTTTATCGCCAAGAACACCGTTATGATCTTCTCCAAGTCGTAC
TGCCCGTACTGCACGAAGGTCAAGCAGCTATTCCAGGGCCTCGGCGTCAACTTCACTGCC
GTGGAGCTTGACCAAATCGCCGATGGCAGCGAGATTCAGGCCGCGTTGAAGCAGATCACC
GGCGGCACCACCGTCCCGCGCGTGTTCATCGACAGTGAGCACATTGGTGGCAACGACGAC
ACCCAGAACCTGCACAAGAAGGGTGGCCTCGTGCCCAAGCTCACCGCTGCTGGTGTGACC
GTGAAGCAGTAA
>g1229-161946-163008
ATGGCGATGCAAGCGATCAAGTGCGTGGTGGTCGGCGACGGTGCCGTCGGTAAGACCTGT
CTTCTGATCTCGTACACCACCAACGCCTTCCCCGGCGAGTACATCCCTACGGTCTTCGAT
AATTACTCGGCCAACGTCATGGTCGATAACAAGGCCATCAACTTGGGCCTCTGGGATACG
GCCGGCCAGGAGGACTACGATCGTCTCCGCCCGCTGTCCTACCCGCAGACCGACGTCTTC
CTCATCTGCTTCTCCGTCGTGTCGCCTCCCTCGTACGAGAATGCCCGCAACAAGTGGAAT
CCCGAGATCATGCACCACTGCCCGACCACCCCCAAGCTGCTCGTCGGTACCAAGACCGAT
TTGAGGAACGACGCCGACACCATCGCGCGCCTCGCCGACAAGAAGATGCAGCCCATCCAG
CAGGACCAAGGCGACAAGCTCGCCAAGGAGGTCGGTGCCGTCAAGTACCTCGAGTGCTCC
GCCCTCACCCAGCAGGGCCTCAAGAACGTCTTCGACGAGGCCATCCGCGTCGTCCTCAAC
CCGCCCTCGCCCGCCAAGAAGGAGAAGAAGGGCAAGTGCTCGCTCTTCTAA
>g1271-78097-79898
ATGAAGGCGAACACGGCACTGCGGCCCTACGTGGCCCCGAAGTGGGCGCAAGAGGCGGGC
CTCAAGGTGGTGCCCACCAAGTTCGTGTCGCTGACGTCCTCCACCGGCCCCACCCGCCTC
CACCGCTGGCACCTGCCCTCCGTCGATGGTGTGGAGGTGTGGATCAAGCGGGACGACGAG
ACCGGACTGGTGACGTCCGGCAACAAGATTCGCAAGCTCCAGTTTCTCCTGGCCGATGCC
ATCGACAAGGAGCACGACTGCGTTGTCACCATCGGTGGGTGGCAATCGAATCACTGCCGC
GCGACGGCGGCCGCAGCGCGCGAGCTGGGAATCGAGTGCCACCTCATCCTAAGGACCGAC
TACACTCCCGAGAACATCCCCATCACTGGTAATGTGGCGTTGGACATGATGATGAACGCC
CAACTGCACCTGGTGTCGGTGGCCGAGTACAACCGCGTTGGCTCGCCTCAGCTGCTGAGC
GACCTGGGCAAGCGGCTGGAGCAGGAGGGGAAGAAGCCCTACCTCATCCCCGTCGGCGGC
TCCAATGAACTTGGCTCTTGGGGCTACATGCAAGCCACGGAGGAATTGTGTCACCAGATG
AAGGACCTCAACATCGAGTTTGATGATATTATCACAACCATCGGCAGCGGAGGTACCACC
GGCGGATTGGCGCTCGGCGTGGCACTGAGCGGGCTCTCCGCGCGCACGAAGCTCCACGCG
TTCTGTGCCTGCGACGACGACGAATATTTCTACCGGGAGATCGACCAACTGATCACCAAC
CTGGGCCTCGGCGACAGGTTCAAGGCTCGAGACCTGGTCAGTATCAACGACAAGTATGTG
GGCTTGGGCTACTCAATCAGCCAAAAGGAGGAGCTCGAGCTGATCATCAAGGTGGCGCAG
CACACGGGCGTGGTCGTGGATCCGACCTACACCGGCAAGGCCCTGTACGGACTCATTCAC
GAGATCAAGGCTGACCCAGAGAAGTGGAAGGGCAAGAAGGTGCTGTTCCTTCACACGGGC
GGACTGTACGACATCTACTCGCGACTCGATCAGCTCTCCCCGCTCTTTGCCAAGAGCCAG
ATACACACCTTCGCCAGCAGCCAGTAA
>g1271-112912-114048
ATGTCGGAACCCAGGATCGCCTTTCTCGGTGCGGGCCAGATGGCCACTGCCCTCGCGCGC
GGCTTCATCGATGCCAACGTGACGCGACCAGAGAACATCATCGCCAGCGATGCGTACGAG
CCCCAGCTGCCTCGATTTCAGAAGGACACTGGCGGCAGCACGAAGACCACGAAATCCAAT
CTCGAGGCCGTTGAGAATTCGGATGTGGTGATCCTCTCGGTCAAGCCGCAGGTCATGTCC
ACCCTGCTCAAGGAGATCAAGGACGCGGTGGCCTCCTCGCCCCATCTCATCATCTCGATC
GCCGCCGGCATCACGATCGACACCATCACCAAGCAGCTGGGCAGCGACACCCGCGTGGTG
CGCGTCATGCCCAACACTCCCTGCCTCGTCGGCAAGACCGCGGCCGCCTTCTCGCTGGGC
GGCAAGGCCACGCCGGAGGACGGCAAGCTGGTGGCGCACCTGCTCTCGTCGGTGGGCCTC
GGGACCGAGGTGCCCGAGCGGCTGCTCGACGCCGTCACCGGCCTCAGCGGTAGCGGACCG
GCCTACGTCTACCAGTTCATCGAGGCGCTCAGCGACGGCGGCGTTGCCGCTGGCCTGCCG
CGCGACGTGGCCGCCAAGCTGGCGGCGCAGACGGTGCTGGGCGCTGCGCAGATGGTGGTG
CAGACCGGCGAGCACCCGGGCGTGCTCAAGGACAAGGTGGCGAGCCCCGGCGGGACCACC
ATCGCCGGCATCCACGCCCTGGAGAACGGCAGACTGCGCGCGACGGTGATGAACGCCGTG
CTGGCCGCCACCGAACGATCCAAGGAGCTGGCCAAGTCTGAGTGA
>g1271-378261-381467
ATGGATCAGGGATTCGACATGACGGCCAGCATGCACGCGGACGTGCTTCGCATGTGCAAG
GGCGATAAGGAGAAGGCCAGACAGATGGTCTCGGGTCTGCAGGGCCAGATGGAGGAGGAG
AAGCGCAGGTCCCAGCAGCGGAAGATTGATGAGCTCTCCCCCAATTTCCCCAGCATCGAT
CGCCATGCCCTCGTAAAGATTCTCAGCGACAACGACTGGGATGTGGAGAAGGCCATTCTG
CCCTGCTTCGAGTTCCAGGAGAAGGAGAGGGAGGCCCAACGACAAAAGGAGCGCGAAGAG
CAGAACAAGAAGAGGGAAGAGGAACGTCGTGCCAGGCAGGCCGAGGCGCGCCAGCAAGCG
AAGACCTTCCTGGTGCAGCTGTTCTCGAACGTGCCCGAGGACAAGATCCAGGCCATGCTC
GAGGAGAACGAGGGTGACGTCGACCTCACCACCGACCAACTCATCAGCATGAAGGCCGAG
GAAGAGGAGAAGCAGGAGCAGTTGCAGAAGAGGCGCAAGGAGGAGGAAGAGCAACTCAAG
CGACAGGCCGAACTCGAGAAGCAGCTCAAGATCGACACCCTTGTGCAAAGGTTCGGGGAC
TACTGCAACCCGAACGAGATCGTGAACATCCTCTCCAAGCACAACTGGGACGTCAAGGTC
GCAAGCAATGACGTGTTGCGACTTGTCGAGGAGCGTAAGCTCGAGCAGCTCAAGCGCGTC
CACACGACTCTGGACGAAGTGGTCATCAGGGAGGCGCTCTACAAGAACGACTGGAACCTG
ATCGACACGATGAAGCAGCTGCGCCTCATGGCGGCCGAGCAAAAGGCCCAGCAGGCGCAG
AAGGAGGAGGAGGAGCGCCGCAGGAAGGCGCAGGAGGAACTGGAGAAGGCCAAACAAACC
GCCGAGGAGGAGGCCAAGGCTCGCGCCGAGGCCAAGGCACGCGAGGAAGCCAAAGCCGCC
GCCGCGGAGGAGAAGAAGAAGGCTGCCGACAAAGCGGCTAAGGAGGCCGCAGACAACATG
TTCCTCGAGCGCTCGGTCATCATCGGCAAGGAACTGGACGAAATCATCCAGGCTCAGCGC
AAGATTGCCGAGCACGAGGCCGACCCGCTGACGCTCATCAAGCAGCAGCTCGAGGAGAAG
CTCAAGTTCGGCCCCGAGAACCTGCCCGGCATTCCCGGCATGGTCCCGCCCACTCGCAAG
GTGATCGACGAGCTCCTCGGCAAGGAGAGCCCCGTTCCCGTCACGCCCGAGAAGCCCGAG
CAGGAAGACCTGCCCGAGAAGGTCAAGGTCCTCCCCACCGGCATGGAGTTCACCGAAGTG
GACACGACCGCCGTAGATTCGAAGACGACCCTCAAGGCCTCGCCCGAGTCGCTCGATATC
AGGCACCCCATCACCGTCGAGTGGGAGATGGAGACTGCGCCCTCGAGCTCGGATTGGATT
GGTCTCTACAAGGTGGGCAGCGATAACGGCAGCTACGCCATCTACAACTGGATCAGCCCG
GTGGCCCAGAAGGGCGCCATGACCTTCACGCCCACCGAGTTTGGCGAGTACGAGCTCCGC
TACTTCGCCTCGAGCTCCTCGTTCTTCTCGCGCCAGTACTCTGTCAAGGCTGTCTCCAAC
CCTATCCGAGTCGGACCTCAGCTCAAGCTGGTCCCCTCGTGGGACGCCGCGACTAACATG
CTGTCGGTCAAGTTCGAGCAGAAGTCGGGCAACCAGTACCTCAACGCCTGGGTGGGCCTC
TACGCCAAGACCGAGAAGGACAACAGCCAGTACCGCGCCTTTGACTGGCTCTCGAACGCG
GTCGACCACACGCTTAAGTTCGCCTGCCCCAAGGCCGGCGAGTGGGAGTTCCGCTTCTTC
CCGCAGCGGTCCTACGTCGACGTCGCGCGCGAGAGCGTAACCGTCGGCGGCACCGATCGC
GTCGAGCTGTCGCTCGTGGATGGTCAGATGATCGTCAAGACCGAGCTCACCACCGTCGAT
CCCGCCTACGACAACGTGTGGGTTGGCATCTACAAGACTGATGAGACCAACAACAGGCAG
TACAGGAAGTACAAGTATGTGAGCCAGGCCACGGGCACGATCACGTTCCGGGCGTGTACG
ACGCCGTCGACCTACGAGGCGCGCATCTTTGCCAACAAGGGCCTCGAGGTCGTGAGCAGG
AGCAACACCATCGTCGTCCCGCCCAAGCAGCAGTAA
>g1271-419498-420448
ATGAACCCCAAGTGTGGCGTTTGCAACAAGACCGTGTACCCCGTGGAGAAGATCAGCCCG
GGTAACGGCAAGAACTACCACAAGCTGTGCTTCAAGTGCTCGGTGTGCAAGATCACGCTC
AACCTGAAGAACTTCAAGTCGCACGAGGGTACCCTCTACTGCCCGGTCCACTACGCGCCC
GCGCAGGTCGAGGTGAGGTCGTTCGAATCCGAGAGGAAGGCCGACCAGGGCGAGTACGCC
AGCAACCCCTCCTCCACCCAGGCCGCGGCCGGTGGTGCCTGGGGCCAATCGACTCCCGAT
TCGGGCGAGTACGGCGGCGGCGGCGGCGCTGCTGCTGGCGGCTACGAGCAGGGTGGATAT
GACCAGGGCGGCTACGAGCAGCAGGGCGGCTACGATCAGGGTGGCTACCAGCAGGAGTAC
TACGAGTAA
>g1271-482491-484867
ATGACGGAGTACGGAGTCCAGACTACCAACCTCTTCGATCTCCTCAGCGAGGAGGGTCCC
TCCAAGGCCGCCGCCGCTCAGAAGAAGGCCGCCGCTGATGCTGCCGCCGCTGCTGCTGCC
CCCGCCGCCGCTGCCGCTGCCGCCCAGAAGAAGGCCCAGCAGGCCGCTGGCAAGCCCGCC
GCCGCTGGCAAGAAGCCCGCTCAGGGTCAGGCCAAGCCCGCCGCCGCCGGTCAGGCCGCT
GGCAAGGGTCCCGCCCGCGAGGGCAGGCCCGCCCGTGATGGTCAGGTCAGCCGCCCCGCC
CGTGATGGCCAGGCTCCCAGGCGTGATGGCGCCCCCGGTGAGGGCAGGCGTTTCGATCGT
CCCGCCGGTGAGCGCGGTCCCCGCGTGAACAACGGCGAGCGTCCCCCGCGTCGCGAGGGT
GCCCGACGAGAGGGTGGCGGCGCCGTCGCCACCGTCGACGCCGAGGGCCGCCCCGTCCGC
GAGCCGCGCCAGCGCAACGACCGCAGGGACCGCCCCGTCAAGGACCGCGGTGATGCCAAC
GCCCCCAACTACGAGGGCAGGCGCCCCGCCCGTGGCCGCGTGTTTGACCGACACTCTGGC
ACCGGCCGCGACCCGACCGAGAACAAGAAGGGCGGCGCCGGCAAGTCCAACTGGGGCGCT
GCCGGCAAGGACGAGCTCGCCGCCGTGATCGAGGCCGCCGCCGAGCCCGCCGCCGCCGGT
GAGGAGGCCGAGGGCGCCGTCGCTGCCGAGGGTGCCGCCGCTGAGGGCGCCGCCGCCGCC
GCCGAGGGTGCCGAGACCAAGGGCAAGGACAAGGCCGTCGAGCCCGCCGCCCCGGTCGAG
GAGGACCCCGACGACAAGCTGGTCTCCTTCGAGGAGTACCAGAAGCAGCAGAAGGCCGAG
GCCGCCGCTGCCAAGAAGAAGTTCAAGCAGCCCAAGGCCCGCTCGGCCGGCGAGGGCGTC
AAGCTCGACCCCAAGTGGGCCAACGCCGTTCCGCTCAAGAAGGAGGACGACGCCCAGCCC
ATCTCCATCACCATCGCCAAGAAGGAGGCCAAGAAGGAGGCCAAGGACGCCCAGCCCCAG
GTCCAGCTCGCCAAGAAGGCCGCGCCCAAGGAGAAGACCATCTCCGCCGCCGACATCCTC
CGCTTCGACACCAGCCGCCCACGCGGCGAGGGTCGCGAGGGTGGTCGCGGTGGCCGCGGC
GGCTTCAACCGCGAGGGTCGCGAGGGTGGTCGTGGCGGCTTCGGCGGCCGTGGCCGTCGC
GACGGCGGTGAGCCCACCGGCGAGCGCAGGCCCCGCCGGGAAGGCCAGGGTTCGCCGGCT
CAGGCCGCGCCCCAGGCTGCTGCGCCCAAGAGGGAGGCCAAGGCCGCTCCGGCCCCGGCC
ATCACCGATGCGGCCCAGTTCCCCGCCCTCGGCCAGTAG
>g1271-691301-694119
ATGTTGTCGAAAATACAGCCAGATTTCGGCTGTCTTTTTGTTGTGTGTGTTTTGACGCAA
GCCGTTCCTCCTTCTTCACCTCGCTTCACCACATCGCACAGCATCATGTCTTCCGACGAC
AATGTTGAGACGTTCGCCTTCCAGGCTGAAATCAACCAGCTGCTCAATCTCATCATCAAC
ACGTTCTACTCGAACAAGGAGATCTTCCTGCGCGAGCTCATCTCTAACGCTTCCGATGCT
CTGGACAAGATCCGCTACATCGGCCTCACCGACAAGGCGGCCCTCGAGACCAACCCTGAG
CTCTACGTTCACCTTGTGCCCGACAGGGAGAACAAGTGCATCCACATCATCGACAGCGGC
ATCGGCATGACCAAGGCCGACCTGGTGAACAACCTCGGCACCATCGCCAAATCCGGCACC
AAGGCCTTCATGGAGGCTCTTCAGGCTGGCGCTGACGTGTCCATGATTGGTCAGTTCGGC
GTGGGTTTCTACTCGGCCTACCTGGTCGGCGATCGCGTCGTGGTGACCACCAAGCACAAC
GATGACGAGCAGTACGTGTGGGAATCGGCTGCCGGTGGCTCGTTCACCATCCGCAAGGAC
ACCGAGGGCGAGCCGCTCAAGCGCGGCACCAAGATCACGGTCTACATGAAGGAGGATCAA
ATGGAGTACCTCGAGGAGAGGCGCATCAAGGATCTGGTGAAGAAGCACTCGCAGTTCATC
CAGTACCCCATCTCGCTGTGGGTTGACAAGACCAAGGAGACCGAGGTCGAGGAGGAGGAG
GAGGCGACGGAGGAGAAGAAGGAGGGCGAGGAGGAGAAGCCCAAGATCGAGGAGGTGACC
GAGGAGGGCGAGGAGAAGAAGGAGGGCGAGAAGAAGACCAAGAAGGTCAAGGAGACCTAC
CAGGAGTGGGAGCTGCTCAACAAGACCAAGCCCATCTGGACGCGCAACCCGCAGGACGTC
ACCGACGACGAGTACCGCACCTTCTACAAGAACCTCACCAACGACTGGGAGGACTACCTT
GCCGTCAAGCACTTCTCCGTCGAGGGCCAGCTCGAGTTCAAGTCCATCATCTTCGTCCCC
AAGCGCGCGCCCTTCGATCTGTTCGAGACCCGCAAGAAGCTCAACAACATCAAGTTGTAC
GTGCGCCGCGTGTTCATCATGGACAACTGCGAGGAGCTCATCCCCGAGTGGCTCAACTTC
GTGCGCGGCATCGTCGATTCCGAGGATCTGCCGCTCAACATCTCGCGCGAAATGCTCCAG
CAGAACAAGATCCTCCGGGTCATCCGCAAGAACCTCGTCAAGAAGTGCATCGAGCTTTTC
AACGAGATCGCCGAGAAGAAGGAGGACTTCGACAAGTTCTACGAGTCCTTCGGCAAGAAC
ATCAAGTACGGCATCCACGAGGACTCGGCCAACCGCACCAAGCTCGCCGAGCTGCTCCGC
TTCCACTCGACCAAGACCGGCGACGACACCACCTCGCTCAAGGACTACGTCACCCGCATG
AAGGAGGGCCAGAAGGAGATCTTCTTCATCACCGGCGAGTCCAAGAAGGCCGTCGAGAGC
TCGCCCTTCGTTGAGGGCCTTAAGAAGAAGGGCTACGAGGTGCTCTTCCTCACCGAGCCC
ATCGACGAGTACATGGTCCAGCAGCTCAAGGAGTACGACGGCAAGAAGCTCTTCAACATC
TCCAAGGAGGGCCTCAAGCTCGACGAGACCGAAGAGGAGAAGAAGAAGGCCGAGGACCTC
AAGAAGGCCAACGAGAACCTCTGCAAGGTCGTCAAGGACGTCCTCGGCGACAAGGTCGAG
AAGGTCATCCTCTCGAACCGCCTCGTGGACTCCCCGTGCGTCCTCGTCACCGGCGAGTTC
GGCTGGAGCGCCTACATGGAGAAGATCATGCGCGCCCAGGCCCTCAGGGACAACAGCATG
CACACCTACATGGCCTCGAAGAAGACCCTCGAGCTCAACCCCGAGCACCCGATCGTGTCG
GAACTGAGGAACAAGGCCGACGCCGACAAGAACGACAAGACCGTCAAGGACCTCGTGTGG
CTGCTCTACGAGACCTCACTGCTCAGCTCCGGCTTCTCGCTCGAGGAGCCCGCCACGTTT
GCCTCGCGCATCCACAGGATGATCAAGCTCGGTCTCTCCATCGACGAGGACGCCGGCGCC
GACAAGGGCGATGACGACCTTCCGCCCCTCGAGGCCGACGACCAGGCCGGCGAGGAGGAG
GGCAGCAGGATGGAGGAGGTCGACTAA
>g1271-729355-731133
ATGGAGCACGAGCAGATGACTGGTGATGTGGAGGAGGAGCACAGGCTGCAGGTTTCGCCG
GAGGAGCTCAAAGTGATCCGCGAGACTCTCCTCGACCGTTCTCTCACCATGGCTCAGAGG
TATCGCTCGGTCTTTACCCTCCGTAACATCGGCGGTGATGAGGCCATCTCCATCCTGGCT
GAATCGTTCAACGATCCTTCGGCCCTGCTTAAGCACGAGGTGGCCTACTGCCTGGGTCAG
ATGCAGAACGTCACCGCTCTGCCTCACCTCGAGCGACTTCTGCGCAACGCGGAGGAGAAC
TCCATGGTCCGCCACGAGGCCGGAGAGGCGCTGGGCGCCATCGGTCTGGAGGAATCGCTG
CCGCTCCTCGAGCAGTACAGCCGGGACTCCGTGCCCGAGGTGGCCGAGACCTGCAGCCTG
GCCATCGACACCATCAAGTACAAGCTCAAGCACAAGGGCAACAAGGCTCCGTTCGAGTCG
GCGCACATGTCGATCGATCCCGCTCCTCCCTCGGCCAAGCGATCTGTTGAGGAGCTCAAG
CAGCGACTCATGAACACGTCGTTCTCGATGTTCAAGCGGTACCGCGCCATGTTTGCCCTT
CGCGAACTCGGCACGGCCGAGGCTGCTATGGCTCTGGCGGACGCCTTCGTCGACAGCAGC
GCCTTGCTGCGCCACGAGATCGGCTACGTGCTGGGACAGATGGCCCACGAGGCCGCTGCT
CCCGCGCTCACCAAGGTCCTGCAGAACCTGGACGAGCACCCGATGGTGCGACACGAAGCT
GCGGAAGCCCTCGGCGCGATCGCCACGCCCGAGGCCATGGATCTGCTGGGCCAGTTCCTG
ACCGACAAGGAGCCCGCCGTCAAGGAGAGCTGCGTCGTCGCGTTGGACATGAGCGAGTAC
GTGAACTCGGACAGCTTCGAGTATGCCGACACCCTGCAGCTGGCCAAGGCCCAGCTCGCC
GCCGCCCAGTGA
>g1271-828281-829321
ATGTTGAAGCTATCGTCCAAGCGCTCGCATGACATGATCATCGACGCGCCTCCGCCCCCC
TGCTCGCCGCATCGCATCCAATTCCTCGGCTCGCCCCCTTCCCCTCCCTCGCCCTTCAAG
CGGGTGCGAACCTCGCCTGCGGTAGAGCCGCAGTCTCGGAAGCATTCGCGGGATGAGAGC
CCGTTCGCTCCCGCCTCGCCATTCAATCCAAATGTGGCCGAAAGATTTGTGCCCAGAAAG
AAGCAGCGCGCTGAGGAAGGAGAGAAGCTTTACACCTTAGAGGAGGTGAAGCGGATCGTG
GCGCAAGTGGTGGCAGAACGAGAGAATGCTCTGCGGGAGGAGTACACGCAAACGCTACAA
CAGAAGCTTGAAGATCAATTCAATCTCTTCTCCAATTTTGCCCAAGACAACATCGCGAGA
CAGCTGAGGGAGAATGACACCTGCAGCTACATCGGCTAA
>g1271-997171-999447
ATGTCGCACATTAAGAAGGAGAAGAAGAAGTCCAAGGAGACCCTCGGAGAGACCCAGGCC
GGAGGCGATTTCTCCATTTCGCCCGCCGCTAAAGGTGCCGTCCTTGATACCTCCAAATGG
CCACTCCTATTGAAGAACTACGACAAGCTCCTTGTCCGCACCGGACACTACACCCCCATC
CCCACCGGTCACTCGCCGCTTAAGCGCCCGCTCGATGACTACGTCCGTTATGGTGTGATC
AACCTCGACAAGCCCTCCAACCCCTCCTCGCACGAGGTTGTGGCCTGGATCAAGAGGATC
CTCCGCGTCGAGAAGACCGGTCACAGCGGCACCCTTGACCCCAAGGTCACCGGTTGCTTG
CTTGTGTGCATCCAGAGGGCTACTCGCCTGGTCAAGTCCCAGCAGGGTGCCGGTAAGGAG
TACGTCGCCGTCGTCCGCCTGCACAGCGCCATCGACGAGGAGGCCAAGCTGGCCAAGGCC
ATCGAGACTTTGACCGGTGCGCTGTTCCAGCGTCCCCCGCTGATCGCCGCCGTGAAGAGG
AGGCTGCGCGTGCGTACCATCTACCAGAGCAAGCTCGTCGAGTTCGACAAGGAGAGGAAC
CTCGGTATCTTCTGGGTCGACTGCGAGGCCGGAACCTACATCCGTACCCTCTGCGTCCAC
TTGGGTCTTCTCCTCGGCGTCGGCGGTCACATGCAGGAGCTCAGGCGCGTCAGGTCCGGT
CTTCTGACTGAGAACGCCAAGGACTGCTTGGTGACTTGCCATGACATCTTGGACGCCCAG
TACGTCTACGACACCACCAAGGACGAGTCCTACCTTCGTCGCATCATCCAGCCCCTCGAG
GTGCTCCTTACCAAGCACAAGCGCGTGGTCGTCAAGGACTCTGCCGTCAACGCCATCTGC
TACGGTGCCAAGTTCATGATCCCTGGTCTGCTCCGCTATGAGGAGGGCATTGAGGTCGGC
GAGGAGATCGTGATGATGACCACCAAGGGTGAGGCCATCGCGCTCGGCATTGCGCAGATG
TCCACCGCCGACATGGCCTCGTGCGACCACGGCGTCGTCGCCAAGATCAAGAGGGTGATC
CTCGAGAGGGACACCTACCCCCGCAGGTGGGGCAAGGGACCCAGGGCTCAGCTGAAGAAG
CAGCTGATCAAGGACGGCAAGCTCGACAAGTTCGGCCGCCCCAACGACAACACGCCCAGC
GAGTGGAAGAACACCTACGTTGACTACAACGTGCCCAAGGGTGAGGCCCTGCCCGCGCCG
GTCAAGTCGACGCCCACCACCCCGATGAAGATCGACGTCACCATCAAGGAGGAGCCCAAG
GCCGACAAGGAGGAGAAGAAGGAGAGCAAGAAGAGGAAGAAGGAGGAGTCGTCGGACGAT
TCCGACTCGGACTCGGATTCCGATTCCTCTTCGTCCGAGGAGGAAAAGAAGAAGAAGAAG
AAGAAGGCCAAGAAGGCCAAGAAGGACGAGACGCCCTCGAAGAAGGACAAGAAGAAGAAG
AAGAAGTCCGCCTAA
>g1271-1002551-1003802
ATGAGCGGCTCCAATAGTGTGGTGTACGTCGGTCGTCTGTCCTCCCGAACGAGGGAGCGC
GATCTTGAAGATGCTTTTAGCAAGTACGGTCGCATCATCCGTCTGGACATGAAGGCCGGC
TACGCTTTCATCGAATACAACGATTCTCGCGATGCCGATGATGCTGTTCGCGGAATGGAC
GGCAACGATCTCGATGGTGCCCGCATTTCCGTCGAACCCTCGCATCGCGGTGAGGGTCGC
TGCTTCTCCTGCGGCAAGGAAGGCCACTGGGCTAGGGACTGCCGTGAGGGTCCTCGCGGT
GGTGGCAGGATGGACCCCAGGGACAGGCGCGGCGCAGGTCCTGCCCGCGCCTGCTACAGC
TGCGGCGAGGTCGGCCACTTCGCCCGCGAATGCCGGGGCCGCGGTGGCGATCGCTACCAC
CCCTACAGCAGGAGCAGCAGCCGTCGTGGAAGGAGCCGCAGCAGGAGCCGTTCTCGTTCG
CGCTCCCGATCTCGCTCGCGTTCGCCCCGATCGAGGGACAGGTCTCGCTCCAGGGACAGG
CGCAGGGACAGCCGTTCCCCTTCTCGCTCCAGGTCACCCAGACGTTCGAGGTCTAGGGAC
AGGCGCAGCAGGGACCGTTCCGCCAGCCGTGGCAAGAGCCCTTCGCCCAGGAGGGACAGG
AGCCGTACTCCCGCCAAGGAGGACAATGGCAAGGCCGCTTCTCCGCGACCTGACAACGGA
CCTTCTTCCCCCAAGGCGGAGACCAGGCGTAGCCCTTCGCCTTCGGCCGAGTAA
>g1271-1023131-1023827
ATGAACCGTCTGTGCCTCGCCTCGCGCCCTGCTGCCTCCCTCCTCATCGCCCGCCAGGGC
TCGTCGGCTGCCAACAGGATGGCCATCAGGCGCTTCACCGAGGACATCAACCCCTCGCGC
CTTTTCATCGGAGGTCTCTCGTGGGGCACCGATGAGAACAAGCTCAAGGAGGCCTTCGAG
GGTTTTGGTGAGGTCAGGCACGTGCGAGTTGTGGTCAACAGGGAGACCGGTCGCTCGCGC
GGCTTCGGCTTTGTGACCTTCCAGTCCCCCGAGGACGCCGCGGCCGCCGCTGAGGCCATG
CACGACAAGGAGCTTGATGGCCGCAGGCTCAGCGTCTCCTACGCCAGGAACACCCCGCGC
GAGGGTGGCTTCAGGCAGCGCCCCGTCGAGGGCAAGGAGGAGGAGAGCATGTAA
>g1271-1040527-1041053
ATGTCCATCCACAAGCTCGACAAGACCCACGCTACGGTGAACCTCGAGGAGGCCGCCACC
CGCACCGGCACCATCCGCTCGGTGCCCTACCACGAGACCATCGCCTACCAGCGCTTCTAC
GCCAATGTGTTCACCAAGGCCACCGTCTTCGGCAAGAGGCTGATTCCCCTGACCAAGAAC
TACGCTCCCACTGCTTTCCGCTGGGGTGCCCTCGCAGGTCTCGCTGCGGCCTACGTAATC
GAGCCTTCCTTCCTCACGAAGTACTTCACCAAGTCTGAGTCCAAGTAA
>g1271-1045125-1046333
ATGTCGAACAAAGCCGCCGCTGAAGCCAAAGCCAAGGGTAACGCCTTCTTCCTGAAGAAG
CAGTACCCCGAAGCCATCGAATGGTACACCAAGGCCATTAAGGCCGACCCCAATGACTCC
ACCTTCTACTCCAACCGGTGCGCTGCGTACATGGGCCTTGATAAGTTCAACGAGGCGCTC
GGTGATGCTGAGATGTGCATCAAGCTGCAGCCCGCTTGGGTCAAGGGATGGTACCGCAAG
GGTGCTGCCCTGATGTCTCTTAGCCGCTATGAGGAAGCTGCCATGGCCTTCAGGAAGGGC
GTTGAATACGAGCCTCAGAATGACGATCTGAAGCAGAAGCTCGAGGATGCCGAGAGGCAG
GCCAAGTATGCCCCCAAGAGGTTCCGGGAGGACGGCTCTGCTCTCAACGCCGCCGAGCTC
GCCAAGGAGGAGGGCAATGCTCTCTTCCGTCACGCCAAGTACGAGCAGGCTATTGACAAG
TACACCAGGGCCATCACGCTTGCCTCGACCGAGGAGGAGAAGGCCACCTACTACACTAAC
AGGGCCACCTGCTATGCTCAGCTGCACCATTTCAAGGAGGTGGTGGACGACACTACTGCG
GCGATCAACATCAAGCCCTCTTCCAAGGCTTATCTCCGTCGCGGCCTCGCCCTCGAGAGC
CTGGAGAAGTACAAGCTCGCTCTGGAGGACATGAAGAAGGTGCTGGAGCTGGACCCTAGC
GCGATGGTGGCTTCGCAGGCGATCCACAGGCTCACGCGTGCCGTCAACAACTTGTAA
>g1271-1046431-1047491
ATGTCGTGGGATGATGACGATTTTGAGGTGAAGGTGCCGGCAGACAAGCCTACCATCGTT
AATGCCTGGGATGATGAGGAGGAGGACACCGCCGGTCCTGCTACTGATGATTGGGAGGAT
TTTGGTAAGCCCAAGGAACCGAAGGTCCCCGCTGGTCCGGCCGAGGAGAAGAAGACATTC
ACGCAAGTATCGGGGAAGAAGAAGCGAGGAAAGGCCCTTGCCGAGAAAATCAGGCAAAAG
GAAGAGGAAGAGGAAGAAGCCCCGCGCAAATTGACCTACGAGGAGAAGAAGAGGCTGCAG
GAGCAAGTCGTGGCCTCCGATCTCGAAAATGCGATGGACGCGTTCGCCGTGAAGGACTCG
GCGCTCAAAGATTCGGACATCCTGTTCGGCGATGACGGCGACGAGGGCGAGGCGAAGAAG
GAGATCACACTCGAGACCTTTAAGCCCAGGAGCGAAAGGGAGTTCATCAAGCTCGCCGAG
CTGGTGAGCAAGAAAGTGGAGCCGTTCTCGGCGAGCGCTTTCTACACGACCTTCCTGAAG
GAGATGCTCAGGCAAGCGACCGTCAACGTCGAGGCCGAAGACGTCAAGGACCTCATCGCC
ACGCTCAACGTCGTCGTCAACGACAAGCTCAAGGCCTCCTCGGCCAAGAAGGGCAAGAAG
GGAAAGAAGAAGGCCCCCACGAAGACCACCGCCGCCGTCCACGAGGATATGGTGGACGAC
GAGTACGACATGTTTGCTTGA
>g1271-1087559-1089173
ATGGCGCTGTCGAGTGGTGATGCGAGGAAGCGATTCGAGGCCGAGAACAAGATCGCGACC
GAAGACCCTGACCACATCTACAAGTACGATGAGGCCAAGCACCAGGGCTGGACCAGCCAG
CGTTTGTGGCAGAAAGACCCCAACTACTTCAAGAAGGTCAAGATCTCTGCCGTTGCGTTG
CTCAAGATGGTGATGCACGCGCGGTCGGGCGGCAAGCTCGAAGTGATGGGCTTGATGCAG
GGCAAGATCGACGGCGATACCATGATCGTGATGGATTCGTTCGCCCTGCCCGTCGAGGGC
ACCGAGACGCGAGTCAACGCGCAGGTGGAGGCCTATGAGTACATGGTCAGCTACCTCGAG
CTCATCGGGCAGGCCGGTCGTCTGGAGAACGCCATCGGTTGGTACCACAGCCACCCCGGC
TACGGCTGCTGGCTCTCCGGTATCGACGTCGGCACGCAGATGCTCAACCAGCAGTACCAG
GAGCCTTGGCTCGCCGTCGTCATCGATCCCACCCGCACCATCTCCGCTGGCAAGGTCGAG
CTGGGCGCCTTCAGGACGTACCCCGAGGACTACAAGGCCCCCGATGAGGCCCCGTCCGAG
TACCAGACCATTCCCATCTCCAAGATCGAGGATTTTGGTGTGCACTGTAAACAATACTAC
CCGCTCGAGGTCTCGTACTTCAAGTCGTCGCTCGACTCGCAGCTGCTCGACCTCCTGTGG
AACAAGTACTGGGTCAACACCCTCTCCTCCTCGCCTCTTCTTGCCAACCGTGACTACTTT
GCTGGAGCGATCCACGATCTGTCGGAGAAGCTGGAGCAGGCGGAGACGCAGCTGTCACAC
AGCGGGCGCATGGGCGGCTACCTGGCGCCGGAGAAGAAGAAGGAGGAGTCGCAGCTGGCC
AAGCTCACCAAGGACTCGACCAAGACGACGATCGAGCAGGTGCACGGGCTCATGGCCCAG
GTCATGAAGGACATCCTCTTCAACATCAACAAGTCCGAGACCTCGTCCTCCTCTTCCTCT
TCTCAGTGA
>g1271-1103220-1104373
ATGCGGCGGAGCACTGTGCACGTTTTTCCTCGTCTTGCTCTGCGCCAGGGCCAGCAGACT
TCTGCTCTTTGCCTGTTCAGCAATGGTTCGCGCACCGCCGTGAGCTCTGAGACGAACATG
GCTTCAATTGCCAGAAATTACGCTGCCCCGGCGAAGGGAGGCAAGGGTGCTCCGGCCGCT
GCCAAGCCCGCCGAAGATGAGCCCGCCGAGCCCCTCAAGCTCGAGACTGTGACATTCCCC
GATTTTGGCGAGGACAAGGCCTTCACCGCCGACATCTCCGATTTCCCCGCCCCCAAGGAC
TGGAAGTACGTGTCCGATTGGAAGGCGTTCGAGTTCTCGCCCCGAGTGCGAAAGATCGGC
GAGGCCCTGTTCCAGCTCAACCAGGTCGAGATGTTCGAGTTGGTGAAGCTGCTCCAGCTG
CGGTTCGACGTGCCCGACAGCGCCCTTATGGGCGGCGGCGTGGTCGTTCAGGCTGCCGGT
GCCGGCGCTCCCGCTGCTGCCGCCGAGCCTGCCGCTGAGGAGCCCAAGGAAGAGAAGAAG
GAGAAGATGATCTTCGACGTTCAGCTCACCGCTGTCGCTGAAGCCGACAAGTTCAAGGTG
CTCAAGGAGATCCGCGCTCTCAAGCCTGGCATGAAGCTGCTCGAGTCGAAGGAGATGGTG
GAGAAGCTTCCGTCCATGCTCAAGCAGAACGTTCCCAAGGAGGAGGCCGAGCAGATGGTT
GCCAAGTTCAAGGAGCTTGGCGGCACCGTGGAGCTCAAGTAA
>g1271-1107511-1109401
ATGACCGACTCGTCGAAGGTGATCGTGTGCGACAACGGCACGGGATTCGTGAAGTGCGGC
TTCGCCGGCTCCAACTTCCCGGCCTCCATCTTCCCCTCGATGGTGGGCAGGCCCATCCTG
CGTTCCGAGGAGAAGTTCGACAACGTCGAGATCAAGGACATCATGGTCGGCGACGAGGCC
TCCAAGCTCAGGTCCATGCTCCAGATCACCTACCCGCTCGACAACGGTATCGTCCGCAAC
TGGGAGGATGCTGAGCACGTCTGGAACTACACCTTCTTCGAGAAGCTGAAGGTGGACCCC
AAGGACTGCAAGATCCTGCTCACTGAGCCGCCCATGAACCCCCTCGCCAACCGCGAGAAG
ATGGTCCAGGTCATGTTCGAGAAGTACGGCTTCAAGGCCGCCTACATCGCCATTCAGGCT
GTCTTGACCCTCTACGCCCAGGGTCTGCTTACTGGTGTGGTCGTCGATTCGGGTGACGGT
GTGACCCACATCGTGCCCGTGTACGAGGGCTTCTCGCTGCCCCACTTGACCCGTCGTCTC
AACGTCGCCGGTCGTGACGTCACTCGCTACCTCATCAAGCTTCTCCTCCTCCGCGGCTAT
GTCTTCAACAGGACTGCCGATTTCGAGACCGTCCGTCAGATCAAGGAGAAGTTCTGCTAC
GTCGGTTACGACCTCGAGCTTGAGAAGCGTCTGGCCCTCGAGACTACCACCCTCGTCGAG
AAGTACACCCTGCCCGACGGTCGCGTGATCCGCATCGGTGCCGAGCGATTCGAGGCTCCC
GAGTGCATGTTCAACCCCGCGCTCGTCGATCAGGAGAGCGTCGGCGTCGGCGAGCTCGTC
TTCGACTGCATCAACAAGGCCGATATCGACACCCGTGCCGAGTTCTACAACCACGTCGTG
CTCTCCGGCGGTTCGACGATGTACCCCGGCCTCCCCTCGCGTCTCGAGAAGGAGATCAAG
CGCCTCTACTTCGAGCGCGTGGCCAAGGGCAACAAGGTCTCGATGCAGAAGTTCAAGTGC
CGCATCGAGGACCCGCCGCGCAGGAAGCACATGGTGTTCCTCGGCGGTGCCGTGCTCGCC
GAGATCATGAAGGACAAGACCGCCTTCTGGATGAACAAGTCCGAGTACGAGGAGCAGGGC
CCTCGCGTCCTCCGCAAGTGCTTCTAA
>g1271-1118439-1119803
ATGTCCACGCGAAAGAAGGTTCTGTTGAAGATCATCATCTTGGGCGACAGCGGTGTCGGT
AAGACCAGCTTGATGAACCAGTATGTGAACAAGAAATTCAGCAATCAGTACAAGGCCACG
ATCGGCGCCGATTTCTTGACGAAGGAGGTCATGGTCGACGACAAGCTCGTTACCCTCCAG
ATTTGGGATACTGCTGGTCAGGAGAGATTCCAGAGCTTGGGCGTGGCGTTCTATCGCGGC
GCTGATGCTTGCGTGCTGGTGTTCGACGTCAACGTCGCCAAGACCTTTGAGAATCTTCAC
AGCTGGCGTGAGGAATTCCTCGTGCAGGCCGGTCCTCGCGATCCCGAGACGTTCCCCTTC
ATCGTGCTGGGTAACAAGATCGATCTCGAGAGCTCGCGTGTGGTCTCGCAGAAGCGCGCA
CAGACCTGGTGTCAGTCGAAGGGCAACATCCCTTACTTCGAGACCTCGGCCAAGGAGGCC
ATTAACGTGGAGCAGGCTTTCCAGACGATCGCCAAGAACGCCATGAAGGAAGAGGAGGAT
GTCGATCTTGCCGGCATTACCGACGCCATTCAACTCGACAAGTCTGAGCCCTCTCAGTCG
GGTTGCTCGTGCTAA
>g1271-1120016-1120501
ATGGGCCGCAGAGGAGGAAGTTCGAGATCATCGGCCAGGCCCGCGTCCAAGCCCACTGCC
ACGGCAGCACCCGCGAAGAAGGCTGCTCCCCCCGCCACCCAGCAGCGCGCTCAGCCCCCT
GCCACCCAGCAGCATGCGCAGACCGCCCCCGCGATGGGTGGTGGCGGCCAGAGCTTCCTG
GGCGGCGTCGCCCAGATGGCTGCTGGCTATGCTGTCGGTCACGTCGCCAGCCGCGCCATC
GAGAACGTTATTTTCGGCGGACACAACGCCACCCCCGAGCAGATCCAGAAGGCCGAGGAG
CAGGTGCAGCAGGGACCTTGCGCGCGCCCCTACGACGGATTCCTCAAGTGCCTGAAGAAG
AACGAGGATGACGCCACTGAGTGTGATTGGGCCTACGACATGTTCAAGGAGTGCCAGACC
ACCAACCGGGCGAACCGCGATTTCGGCTATGGAGGAGAGAGCAAGGAGCAGAGCGCTACC
TACTAA
>g1271-1167429-1169629
ATGATGGCCACACCTACAGCATCCCCTTCTTCCCTCGCTGCGTCGGACGGTGCCGCGCAG
AGGGAATTCCACAAGGCGTCGAAGAAGTCGATCACACAGGCGGTCGAAAATGCGGTTAAC
AACCTGATCGAGTCGGTCGATTCCCACAAGAGCGAGGATGGCAAGGTCTACAGTGCTCAG
GACAAGGTCAACTTCGCCAAGGATCTCAAAGATTTCACCGGCGTCTTCAATGCGTTCCTC
AAGCAGCGCGGCCAGGTCATCGATTGGAATCTGATCAAGCCGCCCCCGAAGGGCATGATC
GTTCCCTACGCCGAGCTTCCGACTGTCCCCGACGCAGAGAAGGGCAGCTACCTCGACAAG
CTCGTCGTGCTCAAGCTCAACGGTGGCCTCGGTACCACCATGGGCTGTGTCGGTCCCAAG
TCGGCGATCGAAGTGCACTCCAAGTACACCTTCCTCGATCTCACTGTCCAGCAGATTGAG
TACCTGAACAAGAAGCACAACGCCAACGTGCCGCTCGTGCTCATGAACTCGTTCAACACG
CACAAGATGACGCAGAAGATCTTGCGCAAGTACCAGAACCACAAGCTCCAGATCGAGACC
TTCAACCAGAGCCGCTACCCGAGGATCTTCAGGGAAACCCTTCTGCCCCTGCCCGACGAC
ATCAACGGCACCCTCGAGGATTGGTATCCTCCCGGCCACGGCGACGTCTTCCCCGCTCTC
ATCAACTCTGGCCTCCTCGACAAGTTCCTCGCCGAGGGCAAGGAGTACATCTTCATCTCC
AACGTTGACAACTTGGGCGCCACCGTCGACGTCAACATTTTGAAGCACATGGTGGAGACC
AACAACGAGTTCATCATGGAGGTGACCGACAAGACCAGGGCCGACATCAAGGGTGGTACC
CTCATCGACTACGAGGGCAAGCCCAAGCTGCTCGAGATCGCCCAGGTGCCGGAGAACAAG
GTCGACGAGTTCAAGTCCATCAAGAAGTTCAAGATCTTCAACACCAACAACTTGTGGATC
CGCCTCGACGCCATCAAGCGCCTCATCGACAGCAAGGTCCTCGAGGACATGGACATCATC
CAGAACCAGAAGTCCGTCAACGGTGCCAGCGTCATCCAGCTCGAGCGCGCCGCCGGTGCT
GCGATGCAGTACTTCCAGCAGGCCCAGGGCGTCAACGTGCCGCGCTCGCGCTTCTTGCCC
GTCAAGAGCTGCTCCGATCTCTTCGTCGTGCAGTCCAACCTCTACTCCCTCTCCAACGGC
TCCCTCGTCATGAACCCCGCCAGGCAGTTCACCACCGTGCCCTTCGTCAAGCTCGGCGAG
CACTTCAACAAGGTCGACCAGTACAAGAAGCGCTTCAAGGGCATCCCCGACATGCTGGAA
CTCGATCACTTGACCGTCTCTGGCGACGTGACCTTTGGCGCGGGCGTTGCGCTGGCCGGC
ACTGTCATCATCGTGGCCAACCACGGCTCGCGGATCGACATTCCCGACGGCGCCGTTCTC
GAGAACAAGGTGGTCTCTGGCAACCTCAGGATCCTCGACCACTAA
>g1271-1190020-1192921
ATGGCAAGCAGCTTCAGCTTCGATGATGGACGCAAGTTCGTGGCCCAGCAGTGGGACGAT
TCGATCGTGCCGGCCTTGCAGGACTACATCAGGATCCCCAACCAGTCGCCTCTCTACGAC
CCCCAGTGGGCCACCAACGGCCTGATCGACCAGGCCGTCGATCTTATGGTGCAGTGGGTT
CGCAATCAGCAAGTGCCCGGTCTGATTCTTGAGGTGATCAAGCATGAGGGCCGCACCCCT
CTCATCTTCATCGAAATTGAGGCCACCACCGACAACAAGGAGGACACCATTTTGCTTTAT
GGCCACTTGGACAAGCAGCCGCCCATGCACGGCTGGGAGGAGGGCCTTGGCCCCCACACC
CCCGTCATCCGCGATGGCAAGCTCTATGGCCGAGGTGGAGCTGACGACGGATATGCCATT
TTCGCTGCCATTACCGCCATCCAGGCCCTCAAGAAGCAGGGAGTGCCCCACTCGCGGTGC
GTGGTGGTGATCGAGGCCTGCGAGGAGTCGGGCTCGCGCGATCTGCCCTACTACATTCAG
CTCCTCCTGCCCCGCATCAAGAGCCCCTCCCTCATCATCTGCCTCGACTCTGGCTGCGGC
AACTACGAGCAGCTGTGGCTCACCACCTCGCTGAGGGGTGCCGTCATGGGCAACCTCAAG
GCCGAGATCCTCACCGAGGGAGTGCACTCGGGCTCCGCCAGCGGCGTCGTGGCTTCGTCG
TTCCGCGTCCTGCGCCAGGTCCTGGACAGGCTGGAGGACGTCAAGACGGGCCAGATCCTC
CCGCCCTTCCTCTCCGCCGAGATCCCCGCCTTCCGCATTGAGCAGACCAAGCGGGCGGCC
GAGGTCCTCGGTGACCTCATCTACAAGGAGTTCCCCTGGAAGGCCGGTGCCAAGCCCGTG
GCTGATGATCTCACCGAGCTGTTCTTGAACAAGACCTGGCGCCCTGCCCTCTCGATCACC
GGAGCCGAGGGACTGCCCAGCCTCGAGAGCTCGGGTAACGTGCTTCGCCCGTACACCACG
CTGAAGGTGTCGCTGCGCCTGCCGCCGACGATCAACGCCGACAAGGCCGGTCAGCAGCTC
AAGGAGTTCTTCGAGGAGAACCCTCCCTATGGTGCCTCGGTGAGCTTCGAGGTCGAAAAG
GCCGGCACTGGCTGGGAGTCGCCTAAGCTGGTCGATTGGCTGGCCGAGTCGGTGGACAAG
GCCTCCAAGGCCTATTTCGGCAAGGAGGTCTGCTACATGGGCGAGGGCGGTTCCATCCCG
TTCATGGGCATGCTGGGCGAGCTCTTCCCCAAGGCGCAGTTCATGATTACTGGAGTGCTT
GGCCCCAAGTCAAACGCGCACGGACCCAACGAGTTCCTGCACATCGAGATGGGCAAGGGC
GTCACGTCGTGCGTGGCCTCCCTCCTCGCCGACCAGGCCTCCCACCAGGTGGCCTAA
>g1271-1193024-1193302
ATGACGCTCGGAGGACTCGATCTTGAAGTAATGAGCGCTAAGGCCGCGCGCCTATACGAC
AAGGCCGTGGTGGTTGCCCGAGTCGGCTTCCACTGGGGCTTCATCCCGTTCATCATCTTC
CTCGGCGTCCGATCCATGAGGCGAGAGCAGAGCTGGCTGTCCCTCCTCAACCAAACCACG
CCATCTCCTTCCCCCGCCCCGCACTGA
>g1271-1193404-1196538
ATGTCGTTTTCGCACAGTGTAGCGCAGCGATTCAACAACAAGAAGCGCCTGGTGAAGGTA
TCGTGGGAGAAGACCAGAGATGAGGACGAGGTGCTGTCGAGTAGACCGCAGGCGCAGGCC
AAGCTGCTGGAGCTGTTCGGCAGAGCCGTGCTGAGCGATAAGCTGCTGCGCGAGATGCTG
CCCGAGTACACCTACAAGAAGCTCATCGCTGTGCGCGAGGCCGAGCGCGAGATGGACTCC
GACGTGGCCGAGCAGGTGGCCGCTGCCATGAAGGAGTGGGCCACCAGGCAGGGCGCCACC
CACTACACGCACTGGTTCCAGCCCATGAACGGCTGCACCGCCGAGAAGCACGACTCCTTC
ATCTCCCTCAACAAGAACGGTGGCATCGATCTCAAGTTCGGCGGCTTCAACTTGGTGAAG
GGAGAGCCCGACGCGTCGTCGTTCCCCAGCGGTGGCATTCGCTCTACGTTCGAGGCCCGC
GGCTACACGGCGTGGGACATGAACACGCCGGCCTTCCTGCGCAAGGGCATCAGCAAGGGC
CAGTCGGAGTACACCACCCTCTGCATCCCCACCGCCTTCTCCTCCTACACCGGAGACGCG
CTCGACAGCAAGACCCCACTCATCCGCTCCGAGGAAGTGCTTTCCAAGGCCGCCGTTCGC
TTGCTCCACATTCTCGGTGAGACCGACGTGAAGCGTGTGTACTCGACTCTCGGAGCCGAG
CAGGAGTTCTTCCTGATCGATAGGGGATTCTACCTTCTCCGTCCCGATCTTATCGCCACG
GGCCGTACCCTGATCGGTGCGAGGCCGCCCAAGGGCCAGGAGCTGGAGGAGCACTACTTC
GGCAAGATGCCGCGCAGGGTGCAGGCCTGTCTTGAGGAGGTCGACCAGGAGCTGTGGGAG
CTGGGCGTGCCCTCGATGACCAGGCACAACGAAGTGGCCCCTTCGCAGTATGAGATGGCC
CCCATCTTCGAGAAGTCGACCCTCGCGTGCGACCACAACATGCTTACCATGGAAATTATG
AGGGACGTCGCCAAGAAGCACGGCCTCGTGTGTCTGCTCCACGAGAAGCCGTTCTCCTAC
ATCAACGGATCGGGCAAGCACAACAACTGGTCCATGTCGACCAACACCGGCAAGAACCTG
CTCAACCCGGGCGTCACGCCCGAGCAAAACCTGACCTTCCTCACCTTCCTCACGGCCGTT
CTGCGCGCCGTCCACCTGCACGGCGACTTGCTTCGCGCCGTCGTCACCGTCCCCGGCAAT
GACTACCGTCTGGGCGCCAACGAGGCTCCTCCCGCCATCATTTCCGTCTTCTTGGGCAGC
CACCTTAACGCCGTGTGTGAGGCGATTATTCAGGGCAAGGCGGAGGTGGGTACGCCCCAG
GGCATGAAGAGGAAGGTGAGCACGATCATGGAGCTCCATTCCATCGCCAAGATTCCCCGC
GACACCACCGACAGGAACAGGACCTCGCCCTTCGCCTTCACGGGCAACAAGTTCGAGTTC
AGGGCTGTGGGATCGTCGCAGACTTGCGCCACCCCGGTGACGACCATCAACACGATCATC
GCCGACAGCTTGATCTACCTCGCCGACCTGATCGAGAAGCGACTGAAGGAGGCCGGCTTC
ACGACGGACCGCGCGGTCAACATCTCGACCGCGGAGGAGGACGAGCGCGAGGACATCATC
AACGCCGTCATCTGCGACACCCTCAAGGAGCACTACGACGTCGTCTTCAACGGCAACAAC
TACAGCGAGGAGTGGACCGAGATCGCCAAGGAGCGCGGCCTGCCCAACCTGAGCACGGCG
CCCGAGGCCCTCGCCGTCTACACGGCCAAGAAGAACATCGACATGTTCGCCCGGCTGGGT
GTTCTCTCGGAGACTGAGCAGCACGCGCGAAGCAACATCACCAACGAGTTCTTCCTCAAG
AGCCTCGTCATCGAGGGCCAGTCCATGGTCCAGCTCGCCGAGAACTACGTCCTGCCCGCC
GCCCTCCAGTACCAGAAGAACGTCGCCGACTCCATCATCGTCGCGGCCCAGGTGCTCGAC
AAGGAGGCCGCCGGCCTGCTCGCGCCCCAGAAGCAGCTCGTGGCCAAGGTGACCGGCCTC
ATCGCCGATCTCATGCGCACCACCCAGGCCCTCAAGAGCGTGCTCGACAAGGAGAAGAAC
GAGGATGAAGAGGATCCGCAGCGCATGCTGCACGTCTACCACCCGGCCATCAGCAGCGGC
ATGAGGGACGTGCGAGCCGTGTGCGATGCGCTCGAGGAGACGATCGACGACGCCCTCTGG
CCGCTGCCCAAGTACTCCGAGATGCTCTTCCTCGTGTAA
>g1271-1254117-1254741
ATGGCGGCACGCAAGGGCAGCAGCGGTTTCCTCTCTCGCATCTCTTCCTCCTTCAAGCCC
GTCTCTGGCTACCAGGCCTACGGTCTCAGGCTCGAGGATTTCTACAATGCCGAGAACCCC
GAGATCCAGGAGGTGCTGAGGAGGCTCCCCAACAAGACCAAGGAGGAGAGGGACCTCAGG
ATCCGTCGCGGTCATGAGCTCCACTTGAAGGGCACTACCCTGCCCGAATCATCGTGGACC
ACCCCTGAGGAGGATGGTCAGACCTACATGGAGCCCTACATGAGCGAGGTTGTCCAGGAA
ATCGAGGAGAGGAAGGACTTCCGGGCCGACTTCTTGCTCCCCGTGGAGAAGAGGCACAAG
CGCAAGTAG
>g1277-32596-33571
ATGTCGTCGAAGAAGGCCGCCCCCGCCAAGGCTCCTGCCAAGGCCGCCGCCAAGGCCCCC
GCTAAGGCCGCTCCCGCCAAGCCCGTCAGGGTCCGCAAGGAGAAGAAGCCCACTCAGGAC
CTCACCATCAAGACTCCCCTCGGTATTGCCCGTGGTCTTAAGAGGGGCTTCCCCGTCACT
AAGAGGACCAGGAAGATCCGTACGGCCGACGTCAGGAGGATTGGTAAGCGCACCAAGTTC
GTCCGTGAGGTCATCCGTGAGGTCTCCGGCTATGCCCCCTATGAGAGGCGCATCATTGAG
TTGATGAGGGTCGGTCTTGACAAGCGTGCCCTCAAGCTCGCCAAGAGGAAGATCGGTACC
CACCGCCGTGCGCTCAAGAAGAGGGAGGAGATGGGTAACGTCGTCTCGCTCCAGCGTCTT
GCCGCTTCCAGGGCCAAGGAGGATGCCGAGGAGCAGCAGTAA
>g1295-208799-212120
ATGTCGGTCATCGCAAGATTCCCCAAATCGGACCCCCCGCTGACGGCTCTGGTCACTGCC
GAGTTCGTGCCGCAAGACAAGAAGACCGGCATCAAGTTCACTCCCAAGAATGACAAGGAT
CAGGAGATCACCGAACTTGCCTTCTTCCCGCAAGGAGACGTGCTGAGGGGCGATCACGTG
ATCGCGCGTTACATCTCGAGGCAGGACCCCTCGAAGGGTCTTGCTGGTGAGGATGCCAAG
GCCCAGAGCAACGTGGACCAGTGGCTCGAGCTGGCGCTGGTGCGTCTGTCGAAGAAGGTC
ACCGACGAGGAAGCCTTCAACGCCGCCCTCCGCCTGCTGGACGCCCACCTGACCCTGCGC
ACGTTCTTCGTGGGCTACGGCGTCACCCTGGCCGACATCGGCGTGTGGGGCGCCCTCAAG
CTCCACCCCCTCTGGGCCAAGGCCTCTCAGACCAAGCATGCTAACGAGCACCTCGTCCGG
TGGTTCAAGTACGTGGACTCGCTGCCTCAGTTCGTGGCGGTGTCGACCAAGTACGCCGGC
GGTACTGTCAAGACCGAGGAGGCTGCGGCCGCGGCCGGCGCGGCTGCCGGTGGGCGCAAG
GCGGCCTCCAAGGCCAAGTACCAGGAGCTCGAGGGCGCTGAGGAGGGCAAGGTCGTCACT
CGGTTCCCTCCGGAGCCGTCGGGCTACCTCCACATGGGCCACGTCAAGGCCGCTCTGCTC
AACTACCACTATGCCCACATGTACAACGGCAAGCTCATCCTCCGCTTCGACGACACCAAC
CCCACCAAGGAGAGCAACGAGTTTGTGGACAGCATCACTGCTGATCTGGCCACCCTGGGC
ATCGTGCCGGCGAAGATCACCTACACGTCCGATTACTTTGACCTCATCATCGAGAAGGCC
GAGTACCTCATCAAGAAGGGCCTCGCCTACATCGACACCACCCCCAACGAGGAGCTCAAG
GCCATGAGGAAGGCGATGATCGAGTCCCCCTTCCGGAACCAGCCCATCGAGGAGAGCCTC
CGCCAGTGGCAAGAGATGAAGCTCGGCTCGCCCGAGGGCCAGAAGTGCGTGCTGCGCGCC
AAGATCGACATGCAGTCCAAGGTCGGCTGCATGAGGGACCCCAACATCTTCCGCGTGGTT
CTCATTCCTCACCACAGGACTGGATCCAAGTACAAGGTGTACCCGATCTACGATTTCGCG
TGCCCCATCGTGGACTCGATTGAGGGTGTGACCCACGCCGGTCGGTCCAACGAGTACCAC
GACAGGGATGGCCAGTACAAGTGGTTCCTCGAGAACCTCGACCTGGCGCACCACCCCAAG
ATCAAGGACTTCAGCCGACTCAACTTCCAGTACACTCTGCTCTCCAAGCGTAAGCTGCAG
TACTTCGTCGACAAGGGCATCGTCGAGGGCTGGAACTCTCCTGCCTTCCCCACCCTCCAG
GGTGTGCTGCGTCGCGGTCTGACGGTGGAGGCGCTGCGCGAGTTCATCATCGCCCAGGGC
TCGTCGTCCCGCACCAACCTCCAGGAGATGGAGAAGCTGTGGGCGCTCAACAAGCAGATC
ATCGATCCCGTCATCCCCCGCTACACCGCCATAGAGACCAGCAAGAAAGTGCCGTTCCAC
CTCACCAACGGCCCTGCCACGCCCTACACCAAGTCGGTGCCTCGCCACCGCAAGAACTTG
GAGCTCGGCGAGAAGGTCCTGACCTTCACCAACACCGTCTACCTGGAGCAGGAGGACGCC
AAGATCCTCAAGGACAATGAGGAGTTCACGCTGATGAACTGGGGTAACGCTATCGTGAGG
AAGATCACCAAGTCCGACGACGGCGTGGTGACCGCCATGGAGGGCGAGCTGCACCTGGAG
GGCGATTTCACCACCACCAAGTGGAAGCTCACCTGGCTGCCCGTCATCGACGATCTCATT
CCAGTCGACCTGCGCAAGTACGGCTACCTGATCACCAAGTCCAAGCTGGCCGAGGACGAG
GACTTCAAGCAGTGGGTCAACCCCAACATCATCAGCGTGACGAGCACCCTCGGCGACCCC
AACCTGAGGCTGGCCCAGAAGGGCGACCGGCTGCAGCTGGAGCGCAGGGGCTACTTCATC
GTCGACCAGCCCTACCTGGGCTTCGACGCCGCCCAGCCGCTCGTGCTCATCCAGATCCCC
GATGGCCACAAGGAGGAGATCGCCGATACCTCTTCCGCTGCCGCCGCCGCCGCGCCTGCC
AAGGGCAAGGCCCAGCAGGCCAAGAACAAGAAGGCCGCCGCCCAGTAA
>g1295-321775-323248
ATGGATCAGCTGCTCAATCAATCCGCTTTCAGCTACGACAACGTACAGAGGAATGCCTTC
TTGGAGGCCAAGGGCTACAAGGCGCCCACGACCAAGAAGACTGGCACCACCATTGCTGGC
ATCATGTTCAAGGATGGTATCGTGCTGGGTGCTGATACGCGTGCCACTGAGGGTCCCATC
GTGTGCGACAAGAACTGTGAGAAGATCCACTACATTGCGCCCAACATCTACTGCTGCGGA
GCTGGCACAAGTGCCGACACCGAGAACAGCACTGCCCTTATCAGCAGCCAGCTGAAGCTC
CACCGGTATGCGACCGGCAAGGAGTCGCGAGTGGCCACAACGATGACCCTCCTCAAGCGC
ATGCTGTGGCGCTACCAGGGCCACGTCGGCGCTGCTCTCGTGCTCGGTGGCGTGGACGCG
ACCGGTGCTCACCTCTACACAATCTATCCTCACGGCTCTACCGATAAGCTTCCCTACGCT
ACCATGGGATCCGGTTCGCTGGCCGCCATGGCCGTGTTCGAAGCCGGCTGGAGGCCAGAG
ATGGAGAGGGCCGAGGCCATGGAGCTCGTGCACCAGGCCATCCTGTCCGGTATCTTCAAC
GATCTGGGTTCGGGTTCCAACGTGGACTTGGTGGTGATCACCAAGGGCAAGGTCGACATT
CACCGCACCTACGACCAGGCCAACCCGCGCCCCTATTCGCGTGTCAGGCCCTACGACTTC
CCTCGCGGCTCCACGGAGGTGATCTCGCATAAGGTGGTCCCGCGCATCTCTGAGTTCAAG
GAGGAGGAAGAGGCCGCGGCGCAAGCGGCTGCCGCCGCCAAGGATACCATGGAAGAGTAA
>g1295-400157-403348
ATGCCCCTCCTCCTCAAGTACAGCGTGGCCACCCTCCTCGTTTGCCTTGCCTGCGCCTTC
CTGTGCGTGTCGGCGCAGGGGACCATTCTGGAGGTGTTGCGCAACCAGACCCGATTCAGC
GACCTGGCCAGCTCCGTTGATGCCTCCGCGCTCGTGCGGGCTCGCCTGGGCAATGGGTCT
GTGGACAGCACGGTCTTTGGCCCCGTCGACCACAAAGCCCCCTTCAACTTGACCGAGCCT
CTCCTCACCTATCACCTCGTGGACGGACGCTACCTCGCGGCTAACTTGAGTGACGGGGAG
CTCCTGCCCAGTCTCCTCAGGCTGTCGAGCTTGAGCAACCAGCCCCAGCAGCTCAAGGTC
TCCATTGACGGCAACGGCACCGTGTCCATCAACGGAGTGCGAGTGCAGCAGGCGGACATT
GCGGCCTCCAATGGCGCCATCCACGCCCTGGAGTCGGTGCTGCCCCTGCCCGGCTCTCTC
GCTAAGGCCCTCGACGCGTTCCCCACCCTCAAGTCCCTCGTCGCTGCCGCCAACCTCACC
CTCCCGGCTGCTTCGTCGACCGTGTTTGCGCCCACCGAGGCGGCATTCGCCAGCCTGAAG
CGCCAGAATCCCGCCCTCCTCGGCTACCTCACCTCCGCCGCCAATGGGTCCCAGGCCGAT
CTCGCCACGGTGCTCAAGTACCACGTGCTGGGCGAGGTGCTCTACGCCCAGGACATCGCG
CTGGGCTCGCAGCAGGCCAACACCCTGGCCGATCAGTCGGTGACCATCACCAAGGCGGTG
GTGAACAACTCCGTGGAGGTTACCTTGAACAGCAGCTCGACGTCTGCCCGCGTCGAAGCC
GTCAACACCCTGGCCTCCGATGGAGTGCTGCATTCCATCAACTCCGTTCTTGTCCCCAAC
GGGTTCGTGTTCAGCCTGAGGAAGATCCTCGTCGGACTCAACGACACCACGCTGCTTCGT
CTGCTGGACCAGGCCAACCTCACCAGCTGCTTGACCGAGGACTGCGGGTACACCATTTTT
GCCCCGAGCCAGGAGGCCTGGGAGAAGGCCGACGAGAGCGACTACGGCAGGACCACCGCG
AGGCTGGCCAGCGTCTTGAAGTCCCACATCTACCCGGCTCCGCTCCCGGCTCTCTCGCAG
AACATGACCCTGACCATGCTGAGCAACAAGACCGTGCACATCGTCAACAACACCCTCACC
CTCGAGGGCGATGCCCGCTTGGGCTCGGCCCATCTGCTGGGCTCCGTCACCGCTGGTTCC
AACGGCTACGCCTACGCTATCGACCAAGTCCTGGGCGTCGCTCTGGCCTCTGACGACTCT
CTGAGCAAGGCGACGATTGGTTGGATCATCGTGGGCGGTATGGGCGGCATTCTCTTGCTC
GCTGCCGTGGTCGTGGCCGTCGTGTGGTACGTGAAGCGCAGGCGTGCCGAGTACGAGGTC
ATCAACGGATCCGTGTAG
>g1295-417414-419168
ATGGACGGCAGCGGGTCGCTGTTCGCCACGCTGAGCGGCAACACGCGCGATGTGATCCAC
CGGCTCACGGTCGATCTGCCCAAGAAGCACGGCCGCGGTGGTCAGTCGGCCCTGCGATTC
GCGCGTCTGCGTCTGGAGAAGCGACACAACTATCTGCGCAAGGTGGCCGAGCTCGCGACG
CAGTTCTTCATCACCAACGACCGCCCCAACATCGCCGGTCTCGTGCTGGCCGGTCTGGCC
GATTTCAAGAACGATCTGGCCGCCTCGGACCTGTTCGACCCGCGGCTCAAGGAGATCCTG
CTCCAGATCATCGACGTCTCCTACGGCGGCGACAACGGTTTCAACCAGGCCATCGAGCTC
GCCGCCGAGGGCCTGCAGAACGTCAAGTTCGTGCAGGAGAAGAAGCTGCTCACCAAGTTC
ATGGAGCAGCTGGCCCGCGACACCGGCAAGTACTGCATCGGCGTCAAGGAGACCCTCCAG
GCCCTCGAGCTGGGCGCCGTCGAGACCCTCATCGTCTACGAGAGCCTCGATCACGTGCGC
TACCGCCTCAAGAACCCGCGCGACGGCACCGAGAAGACCATCTACCTCAACAAGGAACAG
ACCAGCGTCGCCTCCAACTTCAAGGACACCGACGGCGGCGACCTCGAGGTCCTCTCGCAG
GAGCCCCTCATCGAGTGGTTCGCCGACCACTACAAGGAGTTTGGCTGCACGCTCGAGTTT
ATCACCAACAAGTCCCAGGAGGGCTCCCAGTTCGTCAAGGGCTTTGGCGGCGTGGGCGGC
ATCCTCCGCTACAAGGTGGACTTTGACATCATCGAAGCCGCCGAGGAGGCCGACGCCGAA
GACGAAGACGACTTCTACCTCTAG
>g1295-487871-490368
ATGTCGAGCCAGCTTACTCACGGGGCCGTGGAGGCCATGCACAAGGGCCAGCAGATTCGA
ACGCCCTTGGTGCAGGTCATTGATGTGAAGAAAATTCTCGGCCCAAATGGGCAAGCCACA
AGCCCCGAACGCTACCGATTGGTGATTTCGGATGGCATTCATTTCCAGCAGGCCATGCTG
GCTACACAGCTGAACGAGCTCGTTAATGATGGTAAGCTGCAGCCCAAGTGCATAGTCCGC
CTCAACGAGTACATTTGCAACACCGTACACGGCCGTCGGATCGTTATCGTGTTGAGCATT
GAGTTGACTGGCCCTCCTCTCGCGCAAACCATTGGCACCCCACAAAACATTGAGGACGTT
GGCGGCGGTGCGGCCGCTCCGGCTCCCGTCGCGGCAGCCCCGGCTCGTCCCGCCCCGCAG
CAGCAGCAGTCGCGCCCCTTCCAGCCGCAGAACACTGGCCGCGGCGGCGGTGGCAGCGGC
AGCAGCTTTGGCGGCCCGTCTCCCTCGCTTGCCGAGGGCACAATGCCGATCAAGGGCCTC
AACCCCTACCAGAACCGGTGGACCATCAAGGCCCGCATCACTCATAAGTCCGACATGCGC
CCCTTCAACAACGCACGCGGCCAGAGCTTCCTCTTCAGCGTCGATCTGCTCGACGCCTTT
GGCGGTGAGATCCGCTGCACGTTCTTCGGCGAGGCTGCCACCCAGTGGAACGATCAGATC
GAAGCCGGTCAGGTGTTTCTGATCGGCCGCGGCAGCGTCAAGTACGCCAACAAGCGGTTC
TCGACGCTCAAGAACGACTATGAGATCTCGCTCGACAAGAACGCGCTCATCCAGCCCACC
GAAGACGACCCGTCGATCCCCTTCTACAAGTTCAACTTTGTCGATATCGCCGATCTGGGT
GGCCATGCCAAGGACGAGACCATCGACATCCTGGGCGCCGTGCTCGAGAGCGGCGCGATC
CAGGACATCCGCACCCAGGCCGGCAACGAGCTGCAGAAGCGCGTCGTCAAGGTGGGCGAT
TCGTCCAACGCGCAGGTCGAGGTCACCCTCTGGGGCGAGCAGGCCGCCCAGTGGAGCGGC
GACCGCGGTCTCGTGGTGCTGTTCAAGGGCGTCAAGATCTCCGACTACAACCAGCGCTCG
CTCACCGTGCTCCGCACCTCCAAGCTCGAGTTCGAGCCGCGCATTCCCGAAGCCGACCGC
GTGCGGGAGTGGTTCGAGACGGCCGGCCAGGGCGAAGTCGCCAGCATGTCCCGCAACGAC
TTCAAGGGCCGCGGCGGTGGCGGCGAGGGCGGCTTCGGCGGCGGCGACAAGCGCCCGTGG
GACGAGACGAGGAAGAGCTTCGCCGCGGTCAAGCAGGTGGCCTTTGGCGGCCAGGAGAGC
GTCTACTTTAGCGTGCGCGCGACGGTGACAGAGATCAAGCACAGCCGGGACCACCCGCCG
TGGTACGAGGCCTGCCCGACCGAGAAGTGCAACAAGAAGGTGACGTCCGTCGGCGGCGCG
TACCACTGCACCAAGTGCGGCGCCACCCACGACCACTACAAGCCGCGATACGTGCTCAGC
CTCAACGCCAACGACCACTCCGGCTCCTCGTGGCTCACCTGCTTCAACGACACCGCTTCG
GATGTCCTGAATGGCACGACTGCGGACGATCTGCTGGAGATGCTGAGTCGGCCGCAGGGC
GAGAGCCAGTACGAGGACGTGTTCCAGAAGGCCCTCTTCAAGTCCTACAACCTGCGCGTG
CGGGCAACGCAGAGCGAGTACGAGGGCGAGAACAAGGTCAAGCTCAGCGTCGTCAAGGTC
ATCCCCATGGACTACGCCAAGGAGTCCAAGTTCCTCATCAACTCCATCGCCAAGTACATC
ACCGCCTAA
>g1295-490433-492218
ATGCAGGCTGACAAGATGGATGCGGCCCCAACTGCGGCGAATGCTGGCCCAACCCCATCG
CCCTTTTCCTACAAGGACTTTGATCTGGAAGGCTACTTGAGCAACTACTCGGGCCACACC
AAGATCTTCCGCGCTATCTTCATCGCCGAGCGCTCGAAGGACCTGGAGCTGGAGGCGACG
CGCATCGCTCTTGAGGAAATCAAGAAGACGCACAACACGGCACTCTACAGAGAGGTCGTG
GACAAGGCCATCGAGAAGCATGGAGGCCTGTTCACGAAAGACCAGGCTTGGATCGACAGC
GTAGAGAAGAAGGCTCAGCAGCAAAACGACAGGCTCGAGCAAGAGCTCAACGGCTACAGA
ACCAACCTCATCAAGGAGAGCATTAGGATGGGACACAACGATTTGGGCGACTTCCATTAC
GACCGAGGCGACCTTCAGTCGGCCCTGAAGTGCTACGTGCGTACGAGGGACTACTGCACG
ACCTCGAAGCACAACATCTCGATGTGCCTCAACGTGATTAAGGTGAGCATTGAGATGGGC
AATTTCGCGCACGTAGTCAACTACGTGAACAAGGCCGAACAAGAGCCCGAGGTGCGAGAC
GCCAGCTGCGACCCCGTCGTGGTGGCCAAGCTCAAGGTGTGCGCTGGCCTCGCCCACCTC
GACACCAGGAAGTACAAGCTTGCCGCGCGCAAGTTCCTCGAGACCACCTTCGACCTGGGC
AACCACTTTAACGAGATCATCTCGCCTCAGGACGTGGCCATCTTCGGTGGTCTTTGCGCG
CTGGCCATGTTTGATCGTGCCGAGCTTAAGAGCAAGGTGCTGGACAACACCGCCTTCAAG
AACTTCCTCGAGCTTGTGCCCCAGGTGAGGGAGCTCATCGCTGACTTCTACAACAGTCGC
TACGCTTCGTGCTTGAACTATCTGCAGCAGCTGAGGCCTGAGCTGGAGCTCGACATTCAC
CTGCACGACCACGTGGAGAGCCTCTACCAGAAGATCCGCAACAAGGCCATCGTCCAGTAC
TTCTCTCCCTTCACCTCGGTCGACCTCAACACGATGGCCCAGGCCTTCAACACCGATGTG
CCAATCCTCGAGAAGGAGCTGGCGGGCCTCATCATGGAGAACTCGATCCAGGCGCGCATC
GACAGCCACAACAAGATTCTCTACGCGAGGACCACCAACGAGCGCTGTAACACCTTCGAA
CGCGCCCTGCGCATGGGCGAGGAGTACCAGCGCAACACCAAGGCCGCGCTCTTCCGCATG
AACCTCATGAAGCACGAGTTCGTCGTGCGCCCTCCCCGCTCCGAGCGTGAGGACCGCGAC
AACAAATGA
>g1297-40667-42668
ATGCTCAGGCGGCTGCAGAGGCTCGGCATCGACAAGACCGACCCCGACCAGCTCACCCCC
GAGGAGGCCGCTCGCTTCTCGCGCCTCGATCTCGACCTTTCCACCCTCACGTGGAACCGC
GTGGTGGACACCAACGACAGGTTCCTGCGTGGCGTCACCGTCGGCACGGGCCCCGAGGAG
AAGGGCAAGACCAGGGAGACTGGCTTCGATTTGACCGTGGCCAGCGAGATCATGGCCATC
CTGGCCCTGACCAACGACGTTGCCGACATGCGGGAGCGCCTGCAGCGCATGGTGGTGGGC
AGCAGCTCCAAGGGCGAGCCCATCTGCGCTGACGACTACGGCGCGGCTGGTGCCCTGGCC
GTGCTCATGAAGGATGCCATCATGCCCACGCTGATGCAGACCCTCGAGGAGACCCCCGTG
TTCGTGCACGCCGGCCCGTTCGCCAACATCGCCCACGGCAACTCGTCCATTCTGGCCGAC
AAGATCGCGCTCAAGCTCGTCGGCGAAGACGGGTTCGTGGTGACCGAGGCCGGCTTCGGT
TCCGATATCGGTGCTGAGAAGTTCATGGACATTAAGTGCCGCTACAGCGGCCTTACTCCC
AACTGCGCTGTGCTCGTTGCCACCGTGAGAGCTCTCAAGATGCACGGTGGTGGCCCTAAC
GTGGTGCCCGGTAACCCGCTTCCCCGTGAGTACGTCGAGGAGCACCTCGACCTCGTGGAG
AAGGGCTGCTGCAACCTGGCCAAGCACATCCAGAACATGAAGGAGTTCGGGGTTTCGGTC
GTGGTCGCCGTCAACAAGTTCGGCACCGACTCGGACTCGGAGATCGACCTCGTGGTCAAG
AAGGCCCTCGAGGCCGGCGCCGACGGAGCCTACCCGTGCACCCACTTCGCCCACGGCGGT
GCGGGTATCGCTGACCTGGCCAAGAGGGTCGTCGAGGTGTGCGAGAAGCCCAACGACTTC
AAGTTCCTCTACCCCCTTGATATCAGCCTCAAGGAGAAGATCGACATCATCGTCAAGAAG
ATGTACGGCGGTGATGGTGCTACCTACAGCGAGCAGGCCGAGAAGAAGCTGGCCACGTAT
GAGAAGCAGGGCTTCGGCAACCTGCCCATCTGCATGTCCAAGACCCATCTCTCCCTCAGC
CAAGATCCCAAGCTCAAGGGTGCGCCCACCGGCTTCACCATCCACATCCGCGACGTGAGA
GCGTCCGTCGGTGCTGGCTTCATCTACCCGCTCGTGGGCACCATGAGCACCATGCCCGGC
CTGCCCACTCGTCCCGTGTTCTACGAGATCGACCTGGACCCCGCGACCGGCAAGATCCTC
GGCCTCTCGTAA
>g1306-148886-150120
ATGAACCAGGAGCAGGTGAGGAAGCAGATCGAGAACATGAAGGCCTTCATCATGAAGGAG
GCCCAGGAGAAGCGGGATGAAATCTTGGCCAAGGCCGATGAGGAATTCTCGATGGAGAAG
GCCAGGCTCCTGCAGGCCGAGAGGATGAAGATCGCTAAGGATTACGAGCGCAAGGAGAAG
CAGCTCGAGACCAACAAGAAGATTGCGTACTCGAACCAGCTGAACCAGGCCCGCCTGAAG
GTGCTTAAGGCTCGCGAGGACATCGTGGTCCACCTCAAGGAGCGCGCTCAGGACCGTCTT
GCCGAGCTCGGCAAGCCCGGCCAGGAGTACGAGACTCTCCTCCAGCAGCTCATCCTCCAG
GCTCTCATCAAGCTCGATGAGACCAAGGTGAGCCTGAGGTGCAGGAAGGACGACGAATCG
TCGGTCAAGTCGGTGCTCTCCGCCGCCGTTGAGGCGTTCAAGCAGAAGTCCCACAAGAAG
GACGTGAAGGTCACCATCGACACCGTCAACTACCTCCCCGCCGGTCCTGGCAAGAGCAAC
TCGCTCGTCTCCTGCTGCGGTGGTGTGGTGCTGAGCGCCCACGACGGCAAGATCGTGTGC
GACAACACCCTCGACCAGCGCCTCGCCCTGGCCTTCGACGCCAACATCCCCAAGATCCGT
TCGCTCGTCTTCTCCTCCTAA
>g1306-162966-163484
ATGGAGGTCGTGGATTACGTTCTCGTTCCCACCAAGAAGTTCGCCAAGGATTCGTTCCAC
TTGGTGAAGCGATGCACCAAGCCTGACAAGAAGGAGTTCATGAAGATCGCGATCGCCACT
GCCATCGGCTTCATGATCATGGGCTTCATCGGCTTCTTCGTCAAGTTGATCCACATCCCC
ATCAACAACATTCTCATCGGAGGCTAA
>g1306-204236-204705
ATGTCGAGGGACCCTGCTGTTGCTGCCGCTTCGTTCGGATTTGTGAACCACCGCTGCGCC
AAGGACGTGAAGGCCTTCCAGGAGCAGAAGGGTTCTTCCGAGGCCGTGAAGGCCTGCTTC
GCCAAGGAGACCAAGACGCTCACCTCCACCTGCGGCAAGGAGGTCCACGCCTTTGCCGAG
TGCCTCGCCGACCCTGAGAGTGGCTCGTGCTCGCGCCCCGAATGGGATCTCCGCGTCTGC
GTGCGGTCCAACTTCGGCTTCGATTCCGCCCTCGAGCAGAAGAAGCAGGCCGCCAGGCTT
AGCAAGTCCACCGTCCCCACCGAGCTTGCCGACTCCGAGAAGCTTGAGACTGCTGCCTCT
CGCTGGAAGTGA
>g1306-229601-232212
ATGATGAACGTAGGTGGTCTGCCGTCGCTCCTCAAGGAAGGAACCAAGCATCTGTCCGGT
GTGGAGGAGGCCTGCCTCCGCAACATCGAGGCCTCGAAGCAGCTCTACCACATTGTGCGC
TCGTCCATGGGTCCCAATGGCATGAACAAGATGGTGATTAACCAGCACGACAAGCTCTTC
GTGACGAACGATGCTGCCACCATCATCCGCGAGCTCGATGTCGTCCATCCTGCTGCCAAG
ATGGTCGTGATGGCCGCTAACATGCAGGAGCAGGAGTGCGGAGACAACACCAATCTTGTG
GTGTCGCTTGCCGGCGAGCTGCTGGTGCAGGCCGAGTCCCTCATCCGCATGGGCCTCCAC
CCTAGTGAAATCATCGCCGGCTACACCAAGGCCGCTCAGCATGCTTCTGAGCTCCTTGAA
GGCCTTGCCTGCCACACGTGTGAGGATCTGCGCAACATCGAGGAGGTGACCCGCTGCCTG
AAGAGCGCTATCGCCTCTAAGCAGTACGGTTATGAGGACATGCTGTCCAAGGTTGTCGCC
AAGGCCTGCATCCAGGTCCTGCCCAAGAACACGAGCAAGTTCAACGTCGATCACGTGCGC
GTGGTCAAGATCCTCGGCGGTGGCGTGCTCGACACCCACGTGATGAAGGGCTTCGTCCTC
GCTCGCGATGCTGTCGGTACCATCAAGCATGTCACCAGCGCGAAGATTGCCGTCTTCGCC
ACGGGCATTGATCTCGCCAAGACCGAGACCAAGGCCAACGTCACCCTCAAGAGCGCTGAC
GAGCTGTTGAACTTTGCCAAGGATGAGGAGAAGCACATGGAGGAGGTGATCAAGCAAATC
TCGGAGACCGGCACGAAGGTGGTGGTTGCGGGTGGCAACGTCGGCGAGCTCGCTCTCCAC
TTCATCGAGCGCTACGGCATGATGGTTCTGAAGGTCGAGTCCAAGTTCCAAATCAGGCGA
CTTTGCAAGGCCACCGGCGCCACTCCGTTGGTCAGACTGGGTGCCCCCATTCCTGAGGAG
CTGGGCTACTGCGACGTGGTCTCCGTGGACGAGATCGGAAGCACCAAGGTGACCATCTTC
AGGCAGGACACCGAAGACTCAGGCATCTCGACCATCATTGTGAGGGCCAGCACGCAGAAC
CTCGTCGACGACATTGAGCGCGCGATCGACGATGGCGTCAATGTCTACAAGGCGATGGTG
AAGGATGGCCGCTTCGTTGCTGGCGCCGGTGCCACTGAGATTGAGCTGGCCAGGAAGATT
CACGCCCAGGGTGAGGCTTCTCCAGGCCTCGACCAGTACGCCATCAAGAAGTTCGCCGAG
AGCCTCGAGGTGGTGCCCCGTACTCTTGCCGAGAATGCCGGACACAACGCTACTGAGATC
ATCTCTCAGCTCTACGCTGCCCACACTGGCGGCAAGACGAACGACGGCGTCAATGTCGAG
ACCGGCGGAACCATCAACGCCGCTGAGGCCGACATTTTGGATCTGCTGGCCAGCAAGGCC
TCTGCCCTCAAGCTCGCTGCCGATGCTGCCACCACCATCCTCCGCATTGACCAGATCATC
ATGGCTCGCGCTGCTGGTGGCCCTAAGCCTCCGGCCATGGGTGCCCGCGACGCCAGCTAA
>g1311-71314-74358
ATGGCCCGAGTCATCTTGTTGCGAGCCGTGCTGATCGTGCTGGCCCTTGCTTGCTATGCG
CACGCTTTCTATCTTCCGGGCGTAGCCCCCATCGAATACAACACGGGCGATCGCGTGTAC
CTCTCGGTCAATCAGCTCACCTCCGTCCACACCCAGCTCCCCATGCGTTACTACACCCTC
CCCTTCTGTCGGCCCGAGACGATCGAGGATGACAGAGAGAACCTGGGCGAACTTCTCCTC
GGTGATCGCATAGAGAACTCCCCCTATCTGCTGGCGGCCAAGCAGTCCGAATCGTGCAAG
GTGCTGTGCCCCGTTACTCTCACCAAGGATGAGGCGAACGCATTCATCGAAGCCATCGAG
CAGGAGTACCGCGTTCACTGGATCGTGGATGGCTTGCCTTCGGCTACCAAGAAGTCGATG
ACCGACGCCAATGGCGAACCCAAGTCCTTGTACGAGGCTGGCCACCCTGTTGGTGAGACG
GTTCTCGCTGCGGGCAAGCCCACGAGCATCTTGAACAATCACGTGGACATCACGATCCTC
TACCACGAGGAGCCCGTGGACTACACCGGCGCTCGTGTGGTCGGCTTTGAAGTGCGCGCT
CACAGCGTGGCTCATAACCTCGACTACCCCAAGGACGGCACTCCTTCCACCTGCCCTCCT
CAGTCTGGCGCCGCCCCCCTGGTCCTGGAGAAGGACAAGGAGGGCCAGAAGGTGCTCTTC
ACCTACTCTGTCAAGTGGGAGCAGTCCGAGCACAAGTGGGCCTCGCGTTGGGACTCGTAC
CTCCTGATGACCGACGACCAGATCCACTGGTTCTCGATCATCAACTCTCTGATGATCGTC
CTCTTCCTCACCGGTATGGTGGCCATGATCATGATGAGGACCCTGCACGCCGACGTCAGG
CGTTACCGTGAGATGGCCGAGAACGCCGAGGAGGCGCAGGAGGAGACTGGCTGGAAGCTT
GTGCACGGCGACGTGTTCCGCGCTCCCTCGCACCCGATGCTGCTGGCCGTCTCGGTGGGC
AATGGCGTCCAGGTGTTCGCCATGACCGTCGTCACCATGATCTTCGCCGTCCTCGGCTTC
CTGTCCCCCGCCAACCGTGGCGCCCTCATGACCGCCATGGTGGTGCTCCTCGTGGTGATG
GGCATCTGCTCCGGCTACTACTCGGCGCGCATCTACAAGATGTTCAAGGGCAAGAACCTG
ACGAGGAACACCCTCGCCACCGCCATGCTCTACCCCTCCATCGTCTTCACCATCTTCTTC
GTCCTCAACACTATCATCATGGGCCAGAAGACCTATGGCGCCGTGCCCTTCCTCACTCTC
CTTGAGGTTCTCGGCTTGTGGCTCTGCATTTCGGTCCCGCTCGCCTTCCTCGGCGCCTAC
TTCGGCTGGAAGAAGCCCGTCGACGAGCCCCCCGTGCGCGTCAACCAGATCCCCCGCCAG
ATTCCCGAGCAGGTGTGGTACATGAAGCCCATCGTGTCCATCCTCATGGGCGGCATCCTC
CCCTTCGGCGCCATCTTCATCGAGCTCTTCTTCATCCTCTCCTCGATCTGGCTGCACAAG
TTCTACTACCTCTTTGGCTTCCTGTTCATCGTGTTCGTCATCCTGATCCTCACCTGCGCC
GAGATCACCATCGTGATGTGCTACTTCCAGCTCTGCTCTGAGGACTACCACTGGTGGTGG
AGGGCTTTCCTCACCTCGGGCGCGTCGGCCCTTTACGTGTTCCTCTACTCGGTCTTCTAT
TTCTTCTCGAGGCTTCAGATTACCAAGTTTGTGTCGGCCATGCTCTACATGGGCTACACT
GCCATCATGGCCCTCGAGTTCTTCCTCCTCACCGGAACGATCGGTTTCTTCGCTTGCTAC
TACTTCGTGAGGCAGATCTATTCGTCGATCAAGGTCGACTAA
>g1311-140145-141738
ATGAGCCGTCAGAAGAAGCAAAAGGAAGAGGAGGCGGTCGTCGAGGCTGTGGAGGAGGAG
GAATGCGGACCCATTCCTATCACCAAGCTCGAGCAACATGGCATCAGTGCTGTTGATGTG
AAGAAGCTTCAGGGTGCTGGCTTCTACACCGTCGAATCGGTGGCTTTTTCGACCAAGAAG
GCCTTGATCGCGGTGAAGGGTGTATCCGACACTAAGGCAGACAAGATCCTCGCTGAGGCC
GCCAAGCTGGTGCCGATGGGCTTCACCACGGCCACTGAATTCCAGAAGCAAAGAGCCGAA
ATCATTCAAGTCACAACCGGATCAAAGGAGCTCGACAAGCTGCTTGAGAATGGCATCGAG
ACCGGCTCGATCACGGAAATCTTCGGCGAGTTCAGGACGGGCAAGACTCAGCTCTGCCAC
CAGCTGTGCGTCACCTGCCAGCTTCCGTTGGATCAGGGCGGCGGTGAGGGCAAGGCTCTG
TACGTCGATACCGAGGGCACCTTCCGTCCGCAGAGGCTTCTTGCCATCGCTGAGCGATAT
GGCCTGAACGGAGACGATGTGCTGGACAATGTAGCCTACGCGCGAGCCTACAACTCGGAC
CACCAGATGCAGCTGCTGGCGCAGGCCTCGGCCATGATGTCCGAGTCGCGGTACGCCATG
CTCATCGTCGACAGTGCCACGGCGCTCTACCGTACGGACTACTCGGGTCGTGGCGAGTTG
TCGGCCCGTCAGATGCACCTGGCGCGCTTCCTCCGCACGCTCCAGCGACTGGCCGACGAG
TTCGGCGTGGCCGTCGTCATCACCAACCAGGTGGTGGCCCAGGTCGACGGTAACGCGGCC
ATGTTCGGCGCCGACCCCAAGAAGCCCATCGGCGGTAACATTATGGCCCACGCCTCGACC
ACTCGCCTCTACCTCAGGAAGGGCCGCGCCGAGACCCGAATCTGCAAGATCTACGACTCG
CCCTGCTTGCCCGAGGCCGAGGCCGTCTTTGCCATCAACGCCGACGGTATCGGTGATCCC
AAGGAGTAA
>g1311-169827-170908
ATGCAGATCTTCGTCAAGACCCTAACCGGCAAGACCATCACCCTCGAGGTCGAGTCCAGC
GACACCATCGAGAACGTGAAGCAGAAGATTCAGGACAAGGAGGGTATTCCCCCTGATCAG
CAGCGTCTCATCTTCGCTGGTAAGCAGCTTGAGGACGGTCGCACCCTCGCCGACTACAAC
ATCCAGAAGGAGTCTACTCTTCACCTCGTCCTCCGTCTTCGTGGCGGTATGCAGATCTTC
GTCAAGACCCTCACGGGTAAGACGATCACCCTCGAGGTCGAGTCGTCGGACACCATCGAA
AACGTGAAGCAGAAGATCCAGGACAAGGAGGGCATCCCTCCCGATCAGCAGCGTCTCATC
TTCGCCGGCAAGCAGCTCGAGGACGGCCGTACTCTGGCCGACTACAACATCCAGAAGGAG
TCCACCCTCCACCTCGTCCTCCGCCTCCGTGGCGGTATGCAGATCTTCGTCAAGACCCTG
ACTGGCAAGACCATCACCCTCGAGGTCGAGTCGTCCGACACGATCGAGAACGTGAAGCAG
AAGATCCAGGACAAGGAGGGTATCCCCCCTGACCAGCAGCGTCTCATCTTCGCTGGCAAG
CAGCTCGAGGACGGCCGCACCCTCGCCGACTACAACATCCAGAAGGAGTCCACCCTCCAC
CTCGTCCTCCGTCTCCGGGGTGGCAACTAA
>g1311-173047-175548
ATGGTGCACGCCGGCAAGGGCGGCGGACGCATGTTCGTCAATGCGGCTCGCATTAACGGC
CGCAAGTCCAACTTCTCGCTTCTCGAGTACATTCAATCGGAGGTGCGGGCCGGAGAGTCG
CCTGTGCCGCCGTGGATGCCGGCCACGCTGATCAGCCCGCCCCCCACCATCCCCAACGTC
CCGCACCCCGGTCCCTTGACCTATCCTGAGGACAAGCTGCGTCAGGTGTGGTTCGATCGC
CATCCCGAGGCGAGGAAGATCAAGCTCAAGACCGTCTACGTGGGCGAGCACAGATATTGG
CAACACCCGGCCACCTCGTTTGTGCACCGACAGAGCAAGCTGATCAAGAAGGGCTACTCT
GAGCAGGATGCCTATGACGTTGTCGAGGCCGAGGACAAGAAGGCCGCGCGCTACAGGGAG
CTGGAGCGGCTGATGGCGATCGAGCAGGCCAAGGAACTTGGCTTTGATGACCTCGAGACG
GCGAGGGACCTCCTTGCCCCTCCCGATCTCACTCAGGCCAGGGAGCGCATGCTGGAGGAG
TTGATCGACGTGCTCAAGGCTAAGGGCGTTCCTAACGTTGTGCCCGAGCTGCTCCCCAAG
GGCATGAAGTGGCGAGACGTACTGGCGTTCGTCCACAAGTACCCCCAGCACGCCCACTTC
TTCAGCCTGGGCTTCATGGCCACTCATGCCATTGAGACCATGAACCCTGAGATTGCTGAT
GCCTACAACTGGATTTACGGCGGAGAAGTGCTTCTCGACGACGACAGTGTGCCGCTTGAA
GAGCAGGAAGAGCTCAATGCCTTCTTGGACGAAGAGGGTTTCGAGACGGTCGAGGACGGC
GACGAACTGGACCAGCAAGACCTTCGAGAGCAGGAGCGGCTGACTTCGGAGGCGGTCGCC
CAGTACGAACCGGTCGTGGCGGGTGCGTCGCTGGTGGAGGAAGGCTTGTTCTTCGACGAA
GACGAGGGCGAAGATGAAGACGAGAGCGAGGCAGCCAGGGAAGAAGAACACGACGAGGAG
CAAAGGATCGCGATGGTCAAGAAGGAAGGCGAGCAGTTCAAGGCGATTCTCAAGAGGATC
GAGAAGATCTACCGCCCCGAGCAGTTCCCCACGACCGCCGACACCCCGTGGGCCGACCTT
GTCAACCTACTCGCCCTTCGCCCCCAGGCTCCCGATGTGAAGCTGGCCCAGGACGCCGAA
CAGAAGCTGAGGAAGGCGAATCAGGTCAAGAGGCCCAGTGGTGGCTCGTCCAGGCCACAG
AGCCAGCAGCCGAACAAGCCCACCAAGGGAAAGGAGGTCGAGCTGCAGTAG
>g1317-147146-147850
ATGGCGACCCCCTTGAAGAAGCCCGTCGAGCAGTATGACGAGTCGCACTACCACCGCATC
CGCATCACCCTGACCAGCCGCAACGTCAAGAACCTCGAGAAGGTCTGCGCTGATCTCATC
AGGGGTGCCAAGAACCAGGACCTCAAGGTCAAGGGTCCCGTCCGTCTCCCCACCAAGCAC
CTCCGCATCACCACCAGGAAGACCCCCTGCGGTGAGGGTACCAAGACTTGGGATCGCTTC
GAGATGAAGATTCACAAGAGGCTCATCGATCTGCACTCGCCCTCTGAGGTCGTGAAGCAG
ATCACCTCCATCAGCATTGAGCCCGACGTGCAGATTGAGGTGACCATGGCCCACACTGCC
TAA
>g1317-148627-149438
ATGGCTTTGGTGCAGCAGGATGATTTCCAGCACATTCTCCGTGTGCTCAACACGAACATC
GACGGTAAGCAGAAGATCGTCTTCGCCCTGACCGCCATCAAGGGTGTTGGTCGCCGTTTC
GCCAACGTTGTGTGCAAGAAGGCCGACGTTGACCTGAACAAGAGGGCCGGCGAGCTGTCC
AACGATGAGGTCGACAAGATCGTCAACATCATCCAGCACCCCCGTCAGTTCAAGATCCCC
GATTGGTTCCTTAACAGGAAGAAGGACATCAAGGACGGCAAGTACACTCAGGCCTACTCC
AACATTCTCGACCAGAAGCTCCGTGAGGACTTCGAGCGTCTCAAGAGGATGAGGTGCCAC
CGTGGTCTGCGTCACTTCTGGGGTCTCCGTGTGCGCGGTCAGCACACCAAGACCACCGGC
CGCAAGGGCCGCACCATGGGTGTGTCCAAGAAGAGGGAGAACAGGTAA
>g1317-154778-156138
ATGGCCACCAAGTCCATCAAGCTGGTCGTCGTTGGCGACGGTGCGGTCGGTAAGACGTGC
CTTCTCATCTCGTATGCCAACAACCGCTTCCCCGAAGAGTATGTGCCCACCGTGTTCGAC
AATTATGTTGTGAATTTGACTGCTGGTGAGGAGACCATCGAGCTTGGTCTTTGGGATACT
GCCGGTCAGGAGGAGTACGATCGTCTGCGCCCCCTCAGCTATGCCAATGCCAACGTGTTC
TTGGTCTGCTTCTCGGTGGTGAACCCCGTATCGTTCGAGAACGTCACCTCCAAGTGGTTC
CCCGAGGTCAACCACTTCTGCCCCAACGTGCCCCTCATCGTCGTTGGCACCAAGCTCGAT
CTCAGGAACGACAACTCCACCCTCGAGAAGCTCAAGGGTCAGGGCCAGCGCCCCGTCACG
CACGAGGAGGGCGAGGAGCTCGCCAGGAAGCTCAAGGCCGTCAAGTTCATCGAGTGCTCT
GCCTTCACCGGTGAGAACCTCAAGACGGTCTTCGACGATGCCGTCAAGTCCGTCCTCTTC
TCCAAGAGGAAGAAGGCTAAGGGTGGCTGCTCTCTGTTCTAA
>g1317-329386-331226
ATGACCACCCTCTCTCCCACTCTCGACTTGAACACCGGCGCCAAGATCCCCGTCGTTGGA
TTGGGCACCTGGCAGGCTGGCAAGGGCGAGGTGGGCGCTGCCGTGAAGGCTGCCATCAAG
GCCGGCTACCGTCACTTCGACTGCGCCGAGATTTACGGCAACGAGGCCGAGATCGGCGAG
GCGTTCAAGAGCGCATTCGATGAGGGCCTCGTCAAGCGTGAGGAGCTCTTCATCACGAGC
AAGGTGTTCAACAACCACCACCAGCCGGAGCGTGCGGTCAAGGCCATCCACAACACCCTC
AAGAACCTCCAGATCCCTTACCTCGACCTCTCCTTGATCCACTGGCCGATCAAGTTCGAG
GAGGAGCAGATCGCGCAGCCGCTCCGCACGCCCGAGGGCAAGCTCAACCCGGCCATCACG
TGGTCGTTCGACTTCAAGGAGACCTGGAAGACCCTGGAGGAGCTCCAGAAGCAGGGCCTG
GCCAAGTCGATCGGCGTGAGCAACTTCACCGTGAAGCAGCTCGAGGAGCTCCTGGCCGAC
GCGCAGGTCGTGCCGGCCGTGAACCAGGTCGAGTTCCACCCGTACCTCTTCCAGGCCGAG
CTCCTCAACTACTGCACCTCCAAGGGCATCGTGCTGACGGCCTACAGCCCGCTCGGCTCG
ACCGTCAGCTCGGAGGGCGTGGTGCCGCTCCTCGAGAACGAGGTCGTGAAGGACATCGCC
GCCGAAGTCGGTCGGTCGGCGGCTCAGGTCGTCCTTCGATGGGGCGTCCAGAAGCACATC
ACCGTGATCCCCAAGAGCTCGAACGAGGAGAGGCTCCGCGCCAACTTCGCCATCTTCGAC
TTTGAGCTCAGCCCCGAGCAGGTCGCCCGCCTGGACAGCCTCCCGCAGCACCGCTTCATC
CGCCTCGTCCCCGGCCACTTCGAGTGA
>g1317-513138-514217
ATGTCCGATTCGACCACCCCCACCACCGAGACCAAGCCCGAGGAGACGAAGACGGAAGAG
ACTCCCGCCGTCGCTGCCGAGGGTCAGACCCCCGCTGCGGAGGAGACCAAGACGGAGGAG
ACTCCCGCCCCCGCCGCTGAGGGCCAGACCCCCGCTGCTGAGGGTGCTGCCGCTGAGGGC
GACCTCTCCGAGGTGAAGGTCGTCACCAACGAGGAGAATGAGGAGACTCTGTTCAAGGTC
CGCGCCAAGCTGTTCCGCTTCGCCAAGGAGACCAGCGAATGGAAGGAGAGGGGCGTGGGT
GATGTCAAGTTCTTGAAGCACAAGGAGAGCGGCAAGATCCGCGTGCTCATGCGCCGAGAG
AAGACCCTCAAGATCTGCGCTAACCACTACATTCTCCCCGCCATTAAGCTCGAGACCAAC
GCTGGCAGCGACAGGTCGTGGGTGTGGACTGCCTACTCTGACGTCTCCGATGAGGAGGCT
GGCGTTAGGGACGACGTCTTGGCGATTAGGTTTGCCAATTCTGAGAACGCCACTAAGTTC
AAGGAGGAGTTCGAGAAGTGCCAGGCCGAGATGGAGAAGCTTTCCAGCGACAAGAAGGAG
TAA
>g1317-520850-522727
ATGAGACCGATTCTGCTCAAGGGACACGAGAGGGCGCTCACGTTCTTGAAGTACAACAGA
GATGGCGATCTGATCTTCAGTGCCGCCAAGGATCCCACCCCGTGCGTGTGGTTCTCCGAC
ACCGGTGAGCGCCTCGGCACCTACAAGGGCCACACCGGTGCTGTGTGGTCGCTTGATGTC
TCATGGGATAGCAAGCTTCTTCTCACTGCTTCCGCCGACACCACGGCCAAGCTTTGGAAT
GTTGAGACCGGCGAGGAGCTCTTCTCCTGGTCTCACCGAGCACCCGTGAGGTGCGTCGCT
TTTGCCAAGGGCGACCGCAAATTCCTTGCGGTGACTGATCCCTTCACCAAGCTGGCCTCG
TCCATCTACATCTACAAGCTCGACCCCGAGAGGCCCGCCAAACAGAACCCGCTGCCCATG
CGGGAGATCGTGGCTCCCACGCCCAGCGCGAAGATCACACAAGCGCTGTGGGGACCCCTC
AACAAATACATCATCTACTCCAGCGAGGACTGCTCGGTGTACATACACGATCCTGAGAGC
GGAGAGCTCCTGCACACAATCACGGACCACCAGGCTCAGGTGAACTCCATCGCCTTCTCG
TGGGACGAGACCTACTTCATCACATCGTCGGATGACCGTACCGCCAGGTTGTACGACAGC
AAGACGTTCAAGCTGCTGAAGACGTTCGAGTCGGCCAAGCCCGTGAACTCGGCTGCGATC
TCGCCCATCCTGGACCACGTCCTGCTGGGTGGTGGTCAGAAGGCGCACGGTGTGACCACC
TCCGCCGTCGAGTCTGGCGGTTTCGAGGCCCGCATCTACGACATGATTTTCGAGGAGGAG
CTGGGCTCCATCAAGGGCCACTTCGGTCCCATCAACACCATCTCCTACGCTCCCGACGGC
AAGAGCTACACGAGCGGAAGTGAGGATGGTTACATGAGGATCTACCACCTGGACAAGTTC
TACTTCACCCTCGGCAGGGACTCTGTGGGCAACTTCCAGGACCAGCTCGACCTCGACGAC
TTCTAA
>g1317-546555-548315
ATGAGCACGGTGTCGAAGCTGAATGGGATCGCGTCGGTGGGCGATCAGAAGACCAAAATC
GCTCAGTACAGAGATCTGCTGAACGAAATCTTCGCGGCGCAGAAGGCTGAGGATTTCAAT
GCCTTCGTCGATCACATGGTGTCTGAGGACACCCCCCTCGTCATCTCGCGACAGATCCTC
CAGAACTTTGCTCAGATCCTGAAGGACCTGCCCACCGAGCTGCACAAGACTGTGGCCACT
TACGCCCTTGCCAAGCTGCAGCCCCGCGTGGTTGCGTTCGAGGAGCAGGTGTCCATCATC
CGCGAGCACCTGGCATCCGTCTACCAAGATGAGGAGGAGTGGGCTGAGGCTGCCGCTTGC
CTGCGTGCCATCCCCCTCGACACTGGCAACAGAGTGCTGGATCCCGAGTACAAGGTCAAC
ATTTACGTCAAAATCGCTCAGCTGTACCTGGAAGACGATGAGGCCGTGCAAGCCGAGACC
TTCCTCAACAGGGCCTCGATTCTCATCCCTGACTGCAAAGAAGCCAACCTGCACATGCGC
TACAAGGTCTGCTTTGCCCGTATCATGGACTACAAGAGGCGCTTCCTCGAGGCGTCGTCG
CGCTACTACGAGCTCTCGCAGATCGTGGGCGAGCGGGAGAGGCTCGACGCGCTCAAGTGC
GCCGTGACGTGCGCCATCCTGGCCAACGCCGGCCCGCAGCGTTCGCGCGTGCTGGCCACC
CTCTACAAGGACGAGCGGTGCTCCAAGATCGACATCTACGACATCCTCGAGAAGATGTAC
CTCGAGCGCGTCCTGAGGAAGCCCGAGGTGCAGAAGTTCGCTGCCGACCTCAAGCCCCAC
CAGATGGCGCTCCTCTCCGACGGCTCGACCGTGCTCGACCGTGCCGTCATCGAGCACAAC
CTCCTCTCGGCCTCCAAGATCTACAACAACATCACCTTCTCCGAGCTTGGCTCTCTCCTC
GAGATCACTCCCGAGAAGGCCGAGCAGGTGGCGGCGCGCATGATGGTGGAGAGCCGACTC
CAGGGAAGCATCGACCAGATCGACAAGCTCATCCAGTTCGAGACTGACGGTGGCTCGCTG
AACCTCTGGGACAAGCACATCGAGGGAGCGTGCCACACCATCAACGTCATCGTGGAGAAC
CTCGGCACCAAGTACCCCCAGTTCATCCAGTCCTAA
>g1317-556358-557497
ATGTCGAGCGAGCGCGAGGAGAATGTCTACATGGCCAAGCTGTCTGAGCAGGCCGAGAGG
TACGACGAGATGGTCGAGGCCATGAAGAAGGTCGCCACCACCGACGTTGAGCTCACCGTC
GAGGAGAGGAACCTGCTCTCCGTGGCCTACAAGAACGTCATCGGTGCCCGCCGCGCTTCG
TGGAGGATTATCTCCTCCATCGAGCAGAAGGAGGAGACCAAGGGCAACGAGGAGCACGTC
AAGATGATCAAGGAGTACCGCAGGAAGGTCGAGAACGAGCTCTCCGGCATCTGCAAGGAC
ATCCTCGCCGTCCTCGACGATCACCTCATCCCCTCTTCCTCCAACGGTGAGTCCAAGGTC
TTCTACTACAAGATGAAGGGTGACTACCACCGCTACATGGCCGAGTACGCCCAGGGCGAC
GGCCGCAAGGAGGCTGCCGATGCTTCGCTCCAGGCCTACAAGTCCGCCTCGGACATCGCC
GTGACGGAGCTCCCTCCCACCCACCCCATCCGCCTCGGTCTTGCCCTCAACTTCTCCGTC
TTCCACTACGAGATCCTGAACTCGCCCGACAAGGCTTGCCAGCTCGCCAAGCAGGCTTTC
GATGATGCCATCGCCGAGCTCGACACCCTCTCGGAGGACTCGTACAAGGATTCCACGCTC
ATCATGCAGCTCCTCAGGGACAACCTTACCCTCTGGACGAGCGACATGCAGGGCGATGGT
GACAACGATGACAAGGAGGAGGAGAAGGCCGGCGACAACGAGGCCAACGAGTAA
>g1317-622615-624389
ATGAAGAGGCGAGTGCTTGTTGGCGTGAAGAGGTGCATTGACTACAACGTCAAGATCAGG
GTCAAGCCCGACAACACCGGGGTGCTCACTCAGAATGTTAAGTTCAGCATGAACCCCTTC
GACGAAATTGCCGTGACCGAGGCAGTCAAGATGAAGAAGGAGGGCGGCTTCGAGGAAATT
GTTGCCGTATCTGTTGGTCCCAAGGAGTGCCAGGAGACCATCAGGCGTGCCCTCGCCATG
GGTGCCGACCGTGGCATTCACGTGGAGACGGACGAGGAGCTGCAGCCCATCGCCGTGGCA
CGGATCTTCAAGAAGCTCGCGGAGAAGGAGCAGCCCCAGGTGGTGGTCCTGGGCAAGCAG
GCCATCGACGACGACGCCAACCAGACCGGCCAGATGCTCGCCGGCATGATGGACTGGTCG
CAGGGCACGCATGCGTACCGCCTCAAGTTTGCGCCCGAGGAGGTCGAGTGCACCCGGGAG
ACCGACACGGGCCTGCAGACACTCAAGCTCAAGCTGCCTTCCGTCATCACCTGCGATCTG
CGCCTCAACGAGCCCATCTTCGCCAAGCTCCCCGAGATCATGAAGGCCAAGTCCAAGCCT
CTCCAAAAGTTCACGCCGCAGGAGCTGGGCGTGGAGACCGCGACGCCCGTGAAGGTGGTG
AAGGTGACCGAGCCGCCCAAGCGCGAGGCCGGCAAGATCGTGGAGTCCGTCCAGGAGCTC
GTGGCCGCCCTCAAGAAGGAGGGTGCCATCCAGTAA
>g1317-629336-630577
ATGGCCCTCAACACGGTTATCTACTATGCTGCCCTCCGCGGTATCGATTACCTGAGCGAG
GCCGTCACCGTCGAGGCTGCCGCCCCCGCCCAGACTGAGACCAAGCCCTCCGCTGCCCCC
GTCAAGGAGGCGCCCGCCGCCAAGAAGGCCGAGGAGGAGGTGGATGAGGACGACCTTTTC
GGAGGCGTCGATGAGGAGGAGCTCGCTGCCGAGAAGAAGAGGCGCGAGGACGAGAAGAAG
AGCAAGAAGAAGGTTGAGGAGATTCAGAGGTCCAACATCATCTTCGACGTCAAGCCTCTC
GGTGACGACACTGACCTGAACGAAATGGAGAAGGTCGTGAGGGCCATCACCCTCGACGGT
CTTACCTGGGGTCCCTCCAAGTTCGTCGACATCGCCTACGGCGTGAAGAAGCTTCAGATC
AGCTGCGTCGTCGTTGACGACAAGGTGTTCACTGAGGATATTGAGGAGGGCATCATGGCG
CACGAGGAACTTGTGCAGTCCGTCGACATTGCCTCCTTCACCAAGGTGTAA
>g1359-61676-62848
ATGCGTTCCTTCCTCGTCTGCGTCGTCCTCGCTCTCGCGATCGCTTGCGCTGCTGCCCAG
CAGACCAGGCCCAAGCTCTCCGAGACTTTCGAGTCCAAGGGCTTCGTGCAGATCAAGAAC
AACGGCACCCTCTTCTTCGGCGAGGGCTGGTACCACGTCTCGCAGCCCGACGGCAAGGCC
CTTGAGGCCTTCGCTTTCGGTGGTGCTGAGCACCTCAACGTCTACGAGCTCCAGAGGTTC
GACAAGGGTAAGGCTTTCGAGGTCATCCACGCTGGTCATGACAAGACCCCCATCTGCCAC
ACCAAGTCCCTGACCGGCAAGATGCCCCAGCTCTGGGATTGGGTTGAGAAGGCCGACTAC
GTCCGCAACTTCACCGCCAACGGCTCCAAGTTCGATCTCTGGGGCTACAAGACCGCCGGT
ATCACCCTCGAGGTCGCCGTGCCCGTCGGCTACCCCAACATCCTCGCTTACTTCGGCCGC
TTCTCCGCCGGAAACGAGTTCTCCTACTACATCGAGCAGTGGAAGACTGATAAGCCCCAC
AGCACCTGGTTCGAGATCCCCAAGGAGTGCCACCTCGGCCCCAAGTAA
>g1359-79036-79757
ATGCCTAAGAGAGGAGCGGGTGCTACCACTGGTGCTAAGTTCAGGATTACCCTCGGTCTG
CCCGTCGGCGCCGTGGTCAACTGCGCCGACAACACCGGTGCCAAGAACCTGTACGTCATC
TCCGTCAAGGGCATCGGTGCCCGCCTCAACAGGCTGCCCGCTGCCGCCTCCGGCGACATG
GTGATGGCCACCGTCAAGAAGGGTAAGCCCGAGCTCAGGAAGAAGGTCACCCCCGCCGTC
ATCATCAGGCAGAGGAAGCCCTTCAGGCGGCAGGATGGTGTGTTTATTTATTTCGAGGAT
AACGCTGGCGTGATCGTGAACCCCAAGGGCGAAATGAAGGGCTCCGCCATCACTGGACCC
GTGGCTAAGGAGTGCGCCGATCTGTGGCCCCGTATTGCCAGCTCTGCCGGCACTGTTGTG
TAA
>g1359-87096-87912
ATGCCTAAGAGAGGAGCGGGTGCTACCACTGGTGCTAAGTTCAGGATTACCCTCGGTCTG
CCCGTCGGCGCCGTGGTCAACTGCGCCGACAACACCGGTGCCAAGAACCTGTACGTCATC
TCCGTCAAGGGCATCGGTGCCCGCCTCAACAGGCTGCCCGCTGCCGCCTCCGGCGACATG
GTGATGGCCACCGTCAAGAAGGGTAAGCCCGAGCTCAGGAAGAAGGTCACCCCCGCCGTC
ATCATCAGGCAGAGGAAGCCCTTCAGGCGGCAGGATGGTGTGTTTATTTATTTCGAGGAT
AACGCTGGCGTGATCGTGAACCCCAAGGGCGAAATGAAGGGCTCCGCCATCACTGGACCC
GTGGCTAAGGAGTGCGCCGATCTGTGGCCCCGTATTGCCAGCTCTGCCGGCACTGTTGTG
TAA
>g1359-122277-123828
ATGGCGTCCCCCGAAAAGAAGACCGTCAAGTGCGTCGTCGTCGGCGACGGTGCTGTCGGT
AAGAGTTGCATGTTGATCAGCTACACACGGAATGAGTTCCCCACGGACTACGTTCCTACT
GTGTTCGACAATTACGAAGCGACTGTGTTGGTGGAGGGCAAGGAGGTCATCTTCTCTTTG
TGGGATACTGCTGGTCAGGAGGCCTACGCCAGGATTAGGACCCTCAGCTACCAGAAGACC
GACATCTTCCTTCTCTGCTTCTCCGTCGCCGCGCGCACCTCGTTCGGCAACGTCACGGAA
ACGTGGGTCCCCGAGTTGAAGCACCACTGCCCCAAGGCGCCCATCATTCTCGTGGGTACC
AAGACCGATTTGAGGAAGGAGCAGGCCGATGTTGTCTCCCAGGAGGAAGGCCAGAGGCTG
GCCAAGCAGATCAAGGCCCTCAGATACATGGAATGCTCGGCCCTGACGAAGGTGGGTCTC
AAGGAGGTGTTCGACGCCGCCATCACCAGCATCGTCTGCAACAACGTGCCCAAGCCCGCG
TCGGCCGGCAAGAAGAGCTGCATCCTCATCTAA
>g1359-124026-124950
ATGAAGACTGAAATCTGCCAGTTCTCTGGGTACAAGATCTACCCTGGTCATGGCCGTCGC
TATGCCCGTACCGACATGAAGACCTTCGTCTTCATCAACGCCAAGGCCGAGAAGGCGTTC
CTCAAGAAGAGCAACCCCCGTAAGGTGCTCTGGACGACCGTGTACAGGCGCATCCACAAG
AAGGGCACCACCACGGAGGTCCAGAAGAAGAAGACCAGGAAGGTCGTGAAGGTGCAGAGG
GACATCGTCGGTGCCACCCTCGACCAGATCAAGCAGAAGCGCGCTCAGAGGCCTGAGATC
CGTGCTGCCGCTAAGGAGGCTGCCCTCAAGGAGATCAAGGAAAGGCAGAAGAAGAAGGAG
GACCTCAAGAAGAAGGCTGCCCCCGCTCCTGCCAAGGGTGCTAAGGGCAAGAAGGTTGCT
GCCCCCAAGGCTACCAAGGCCAAGGCCCCTAAGGCTCCCAAGGGCCAGAAGGGCCGCTAA
>g1359-256957-261528
ATGACCACCTTCTCGATCTCGTCGGTTCTTGAAAAGATGTCCAACAGGGACAAGGACTTC
AGATACATGGCGCTGTCCGATCTTCTTAACGAGCTGAAGAAGGATACCTTCAAGATGGAC
AACGACTCTGAGAAGAAGATTTGCACTGCCATGGTGCAGCTCTTTGACGACCCTTCGGGG
GACGTGCAGGGTATGGCCGTCAAGTGCCTTGAGCCCCTCATCTCCAAGATCAAGGAGGCC
CAGCTGCAGGTCATTATCGACACGCTGAGCGACTATGTGCTGCAAAACAAGAAGAGCGAG
CTTCGAGACATTGCCGGCATCGGTCTCAGGACCGTTGTGGCCCAGACCCCTCCCGAGTCG
CCTACTGCCACCATCGTCATCCAGAGAGTGAACCCCAAGCTCATCCAGGGCGTGTCGCAA
GATATCCCTGAGGTGGTCATGGAGTGCTTGAGCGTCCTGAGCGACGTTCTCAGGCGCTAC
CCCACGCTCGTCCAGGAGCACGAAAAGATCCAGAAGGCCATCGTGCCCCACCTCACCTCT
ACACGCGACGCCTCGCGCAAGAAGGCCATCTCGTGCCTTGGCTACTGGTCCGTCTCGGCC
CCCGACAACCTCTTCTCGGACCTTGTCACCTACCTCCTGAACGAGATCCAGAGCGCCAAG
AAGGCCAACTACATTCGCACCCTCATTTCCGTGATTGGTGCCATCAGCCGTTCTGTGGGT
TACCGCCTGGGCAAGTTCCTCGAGAGCAAGGGCATCATCGGGCTGCTCGTCAAGTACCTG
CACGACGAGCGCTTCCAGTCCGACGACGAGCTCAAGGAGAACACTTTCCAGACGTTCGAG
TCGCTCGTGCTGCGATGCCCGTCCGAGGTGCGGCCCTTCATCGACGAGATCCTGGCCCAG
GCCCTCGAGTTCATCAAGTGGGACCCCAACTACGATGCGGTCTCGGATGAGGAAGAAGAG
GAAGAGGGTGGCGAGGACGACGAGGAGGAAGAGGAGCCCAGCGACGACGAGGACTACAGC
GATGACGACGACATGAGCTGGAAGGTGCGCAGGGCCGCTGCCAAGTGCATCGACGCCGTT
GTCGTCACCAGGCCCGATCTCCTCGAGAAGCTCTACAAGATGGTCGTGCCCGCCATCGTC
GCCCGCTTCAAGGAGAGGGAGGAGAACGTGAAGCTGGACATCTTCGGCGTGTTCATCGAT
GTGCTCAAGCAGACCACCCTCGTCAGCAGGGGCTCCAGGAACACCGAGGAGGGCGTGCTC
GCCCAGCTCAAGTCCAACATCCCCGCGGCGATGGGCAACCTGAACAAGGAGCTCAAGTCC
AAGGCGGTGAAGGCCAAGTCCAGGACCGGCATCTTCCAGCTGCTGAAGGAGCTCGTCCAC
ACCTACCCCGGTGCCCTTAACGAGCACATCGGCGACGCCGTCGTGGGCATCACCACCTCC
CTCTCCGGCAAGGGCACCGACGCCAGCACCAAGATGGAGGGCCTCACGTTCGCCCGCCTT
CTGCTCACCTCGCATGACGCTGCCGTGTTCCACCCGCACATCAAGGCCCTCTCGGCGCCC
GTGCTCAAGGCCGTGGGCGACAACTACTACAAGATCACCGCCGAGGCGCTGCGCGTCACC
TCGCACCTGGTCACCGTGATCAGGCCCCACAACGGCGCCGCCTCGCAGTTCGACCACAAG
CCCTACGCCCAGAAGATCTACGACAGCATCTTTGAGAAGTACAACGCGCAGGACATCGAT
CAGGAGGTCAAGGAGAGCGCCATCACCTGCATGGGTCTCACCGTCGCGCACCTGGGCGAC
GGCCTGTCGGCCGACTCACTCAAGAAGGCGCTCGACATCCTCCTGCAGCGCCTCAGCAAC
GAGATCACCCGCCTCACGTCGGTCAACGCGCTCATCGAGATCGCCAACTCGCCGCTCAAG
GTCGACATCCGCGCGATCCTGCCCGAGGCCCTCACCGAGCTGGCCGCCTTCCTCAGGCAG
GCCAACCGCCAGCTCAAGCAGGCCTCGCTCCGGGCGCTCGCCGTGCTGGTCAAGTCCTAC
GGCGCCGACATCAAGAGCCAGCAGTACGAGGCCGTGCTCAACGAGTCGGTCAACCTCATC
AGCGACGTCGATCTCCACCTCACCCACCTCGCGCTCCGGCTGCTCGAGACCGTCATCTCG
GTCGACAAGGACTCGATCAACGTCGTCCAGGCCAAGCTCTACCCGCACATCCTCGCCCTC
GTGCAGAGCCCCGTGCTGCAGGGCCTCGCGCTCGAGTCGCTCCTCGCCCTCTACAGCGCG
CTCGTCGCGGCCGACCACAAGAAGTTTGGCTTCCAGGAGCTGCTCGACTCGCTCCTGGGC
CTGTCGCTCAAGGCCTCGCAGACCACCAAGCAGAGCCAGGCCAACATCGCCAAGTGCATT
GCCGCCCTGTGCGTCAACGCCACCGCCGACCAGCGCAAGGCCACCGTCGAGCGCTTCATC
GCCGACGTGCGCAAGGCCGGCACCTCGCGCGTGCTCGCGCTCCTCGCGCTGGGCGAGATC
GGCCGCCGCGTCGATCTCTCGGCCCACACCGACATCCAGTCCGTGCTGCTCGACGCCTTT
GACGGTGGCGAAGACGAGAAGTCGGCCGCCTCGTTCGCCCTCGGCAACGTCGCGGTGGGC
AACGTCGAGCGCTTCCTGCCCTTCGTGCTGGCGCAGATCAAGGAGACGCCCAAGAAGCAG
TACCTGCTCATGCACTCGCTCGAGGAGATCATCTCCCACAGCACCGGCGCCGCCGCCGCC
ACTGCCCTGCTCCCGCACCTGACCGAGGTGCTCGCCCTGCTGTTCCAGCACGCCGACAAG
GACGAGGAGGGCATCCGCACGGTGGTGGCCAAGTGCCTGGGCAAGTTCACCCTCATCAGC
CCCGACCAGCTCGTGCCCGCCCTCCAGAAGAAGATCGGCGACGCCTCGCCCCAGACCCGC
GTGTCGGTCATCACCGCCATCAAGTACGCCGTCGACCCGCACCCGCACCCGGTCGACGCC
GCGCTGGCCACGATCATCCCGCAGGTGCTCGCGGGCCTGAGCGACCCCGAGGTCGAGGTC
CGCCGCGCCGCGCTCCTCACCCTCAACTATGCCGCGCACAACAAGGCCAACCTCGTGCGC
CCGGTGCTGAGCGAGCACCTCAACGCCCTCTACGCCGAGACCCTGGTCAAGAAGGAGCTC
ATCAAGGTGATCGACCTCGGCCCGTTCAAGCACAAGGTCGACGAGGGCCTCGAGAACCGC
AAGGCCGCGTTCGAGTGCCTCTACACCCTGCTCGACACGTCGATCGACAAGCTCGACATC
CCGGCCCTCATCAAGCACCTCGTCGAGGGCCTCCAGGACATCCACGACATCCAGCTCCTG
TGCCACCTCATGCTCGTCAGGCTCGCCCAGCACGCCCCCACCGCCCTCCTCACCGGCGTC
GAGCTCCTCATCGAGCCCCTCAGGAAGACCGTCACCAGCAAGGTGAAGGACAACTCGGTG
AAGCAGCAGGTGGACCGCAACGAGGAGCTCATCAGGAGCGCGCTGCGAGCCATCGCCGCC
ATCTCGCGGATCCCCGACATCGAGACCGCTGTCAAGTTCGACGAGTTCGTGAAGCAGACC
GTCAAGACCGGCCCCTACGCCGAGCAGTTCGACAACATCGTCAAGGAGACCGCCCCCAAG
ACCTCCAGCGACTAA
>g1359-285747-286328
ATGCCCAAGCTCGAGATTATCGTTTCCGGCGGCAACTTCAAGCGCATGGACACCAAGGCC
TACAACATCACCAAGAAGGAGGCGTGGGTGAAGCTGCAGGTCGCCGGCCAGGAGGTCACC
ACCACCCGCGTGAACTGCCCCATGCTCGACCCGGTGTTCGATGAGACCTTCGTCCTCGAC
GTCAACGACCCCGCCACCGACAAGGTGACCGTCACTTTCTACCTGGCCGATACCCTCATC
GGTCAGCCCGCCGACTACATCCTCAGCGGCCTCACCAAGAACAAGGGCACCTACAAGGGC
ATGGCCATCGTCGGCGGCAAGCTCGACATGACCTTCCGCGCCCTCGACTTCGGCAAGGAG
GAGGAGGCTCAGGAGGAGGAGGATGATGGCTTCATGGAGTTCCTCTAA
>g1359-331737-333598
ATGTCGTCGACGAAGAAGATGGACGCCTTCCTCACCAAGGATATCTCACAGATGAGCTAT
GAGGAGCTCGAAAAGGCCGCCCGCCACTACGGTCTCGATGAGCAGTCCAAGGTCGACCTC
GTCCAGATTGACGGCCTGGTTGTGCTCAAGATTATCAAGCACTGCCGTGAGAACCTGCCC
GAGCTTGTGACCGGCCAGCTTCTGGGTCTCGATGTAAACTCCACCTTGGAGGTGACCAAC
AGCTTCCCTTTCCCTCAGCGCGAGGAGGAGGCTTCCGTCGACGAGGCCGAGTCGGGTGCC
AAGTACTCGCTCGAAATGATGCGTCATCTCCGTGAGGTGAACGTGGATAACAACACAGTG
GGCTGGTACACGTCCACGTACCTCTCTTCCTTCCTCTCGGAGTCTTTGATCCAGGACCAG
TTCAACTACCAGACCACCATCAGCAAATGCGTGGTCGTGGTCTACGATCCCCTCAAGACC
AACCAGGGCGAGCTGTCCCTCAAGGCCTACCGGCTGTCCGATGCCTTCATCACCCTCTAC
CAGAACGAGGACTTCACCGCTGCCAGCATTGCCCGGGCCGGTCTCTCGTACAACACCATT
TTTGAAGAAATCCCCATCAAGATCAGCAACATCTCCCTCGTCAAGGCCTTCCTCGCCGAG
CTCGAGGACAACGATTTCCTGGATCGGGGCGCCGAGTTCGATCGTCTCTCGGACGTGTCG
AGCGATGCCTTCTTGGAGAAGGGCATGAGGTACCTCATCAAGTCGATTGATGCGCTGGAC
AACGAGCAGAAGAAGTTCCAGAAGTTCCAGTACTACCAGAAGAACCAGAGACAGAGGAGG
GGAGAGGAGGAGGACAGCAGACCGCTGCCGCAGCCGCCGAGCCAGCTGGACAACCTGCTG
ATCACGGGCCGCGTGAACAACTACTGCGATCAGATCAACCAGTTTGTGGGACAGGGCTTC
TCCAAGCTGTGCATGCTGCAGGGCCTGGTGCAGGGACAGGGTAAGGGCAAGGAAGACGAA
ACGACCACCACCAAGACCTCTGCCTAG
>g1359-343816-345818
ATGAAGGCCGCCCTTCTCGCTTTTGCGCTCTTTGGCCTTCTGGCCTTCGCCTCGTCCACT
GTTCACTTCCAGGAGGACTTTGATGACTCGTGGGAGAGCCGATGGGTGTACTCCACCCAC
GATGATGCCTCGGGCAACGCTGGCAAGTTCGCGCACACCGCCGGCAAGTACTTCAACGAC
GCTGAGAAGGACAAGGGCATCCAGACCTCGCAGGATGCCAGGTTCTACAAGCTGTCGGCC
AAGTTCCCGAAGTTCACCAACAAGGACAAGCCCCTCGTGATCCAGTACTCCGTCAAGCAC
GAGCAGTCTCAGGATTGCGGTGGTGCCTACATCAAGGTCGGCCCTGGCCCTCTTGACCAG
GAGAAGTTCGAGGGCGAGACCAAGTACAACGTCATGTTCGGCCCTGACGTGTGCGGCTCG
ACCAAGAGGGTGCACTTCATCCTGAACTACAAGGGCGAGAACCACCTCATCAAGCGTGAG
GTCAGGCCCGAGACTGATATCTACACCCACCTCTACACTGCCGTCCTTTTCCCCAACCAG
ACCTACGAGATCCGCATTGACAACGAGGTCAAGCAGTCTGGCTCGCTCATCGAGGATTGG
GACCTTCTTGCCCCCAAGCAGATCCCCGACCCCGCCCTCAGCAAGCCCGCCGATTGGGTC
GATGAGGAGTACATTGATGACCCCGAGGCCAAGAAGCCCGAGGATTGGGACAACACCCCC
AAGCAGATCGCCGACCCCGAGGCCAAGAAGCCCGAGGACTGGGATGACGAGCTCGACGGT
GAGTGGGAGGCCCCGATGATCGCCAACCCCGACTACCAGGGTGAGTGGCAGGCGCCCCGC
GTCAAGAACCCCGCCTACAAGGGCCCGTGGGTGCACCCGCTCATCGACAACCCCGACTAC
GTGGCTGACGACCAGATCTACGTGTTCGAGAACGAGTATGTCGGCTTTGAGCTCTGGCAG
GTCAAGACCGGTACCATCTTCGATCACATCCTCATCACCGACGACCTCGCCGAGGCCGAG
GCCTTCGCCACCGGCTACTTCGCCGAGCAGCAGAAGGGCGAGAAGGCCGCTTTCGAGAAG
CAGGAGGAGGAGAGGAACAAGGCCGAGGAGGAGGAGAGGAAGAAGCGCGACGCTGAGACC
CAGGAGGCGGACGACGACGCCGAGGACGATGACGACGATGATGACGACGAGGATGACGTT
GCCGATGACCACCACGGTCACGACCACGAGGACCTCTAA
>g1359-350792-353398
ATGCTCAGCCAACACGCCGGAAACCGTACCCTTGCTGACGCCGATCCCGAGGTGCAGGGC
CTCATCCGTCTGGAAAAGAAGCGCCAGCTCAATGGCATCGAGCTCATCGCGTCCGAGAAC
TTCACATCGCGGGCTGTGCTGGAGGCGCTCGGTTCGTGCATGACCAACAAGTACTCGGAG
GGCCTTCCGGGCCGCCGCTACTACGGTGGTAACGAAGTGATCGACCAGGTCGAGAATCTG
TGCATCAAGCGTGCGCTTGAGGCCTTTCACCTGTCGCCCGAGCAGTGGGGCGTGAACGTG
CAGCCCTACTCGGGCAGCCCCGCCAACTTTGCCGCCTACACCGCTCTCTTGAACCCGCAC
GATCGCATCATGGGCCTCGACCTGCCCTCTGGTGGCCATTTGACGCACGGATACCAGACC
GACAAGAGGAAGATCTCCGCCACGTCGATCTATTTCGAGTCCATGCCCTACCAGGTGTCG
TACCAGACCGGCCTGATCGACTACGACAGACTCGAGGAGAACGCCGCTCTCTTCAGACCC
AAGATGATCATTGCGGGAGCCAGCGCCTACCCTCGCGATTGGGACTACAAGCGCCTGCGC
CAGATCGCCAACAAGCACGGAGCCTATCTGCTGTGCGATATGGCGCACATCAGCGGCATC
GTGGCGGCGCAGGAGTGCAACAGCCCGTTCGAGTACTGCGACGTGGTCACCACCACCACC
CACAAGACCCTCCGCGGACCGCGTGCCGGTCTCATCTTCTTCCGCCGCGGCAAGAACGAG
GCCACCGGCGCCGCCTACGACTACGAGGACCGCATCAACAACGCCGTCTTCCCCGCGCTC
CAGGGTGGCCCGCACGAGAACACCATCGCCGCCGTCGCCGTCGCCCTCAAGGAGGCCGCC
GAGCCCGAGTTCAAGACGTACATCCAGCAGGTGAAGAAGAACGCCAAGGTGCTCGCCGAG
ACCCTCGTGTCGAAGGGCTACTCTGTCGTCACCGGAGGCACCGACAACCACCTGGTGCTC
TGGGACGTGAGGCCGCAGGAGATGACCGGCAGCAAGCTCGAGAAGCTGTTCGAGCTCGTG
TCGATCTCTGTGAACAAGAACGCCGTGTACGGTGATGCCAGCGCGCTCTCCCCGGGCGGC
GTTCGTCTTGGTGCGCCCGCCATGACCTCGCGCGGCCTCACCGAGGCTGACTTTGTCCGC
GTCGCCGAGCTGCTCCACAAGGGTGCGCAGATCGCGATTGCGATCCAGAACAAGACGGGC
AAGCTCCTCAAGAACTACCTGCCCGCCCTCGAGACCAGCGAGGAGGTCAAGGCCCTGAAG
GAGGAGGTGGAGGCCTTTGCTTCGACCTTCCCCATGCCCGGCTACGAGAACCACTAG
>g1359-355454-358398
ATGAAGAGGGGAGTGTCTCGAAGCAGCCGTGTTGCGCGTGGCGTGAAGGGCACCAGTGCC
ACTGCGCAGTGCCTCCGTGCTACTGCCGTCGCGCAGCGTTCCGCCTCTACTCTGTCTTCC
ACCTCGAGGCGCAGCGCTTCGCCTCTCTTCTCTGGTCCCGTGGCCCGCTCCACCGCTACC
GTCCAGCAGTTCGGCGTTCGCTCGTATGCCACTCTGCCGGCGCACGAGGTGCTGGGCCTG
CCGGCTCTCTCGCCCACCATGACCCAGGGCAACCTCGCCAAGTGGCTGAAGAAGGAGGGC
GACAAGATCCAGCCCGGCGATCTCATCGCCTCCATCGAGACCGACAAGGCCACCGTCGAT
TGGGAGGCCACCGAGGCCGGCTACCTAGCCAAGATCCTCATCCCCGAGGGCTCCAAGGAC
GTAACCGTCGGCAAGCCTGCCGTGGTGACCGTGGAGGAAGAGGAGGACGTGGCCAAGTTC
AAGGACTTCAGCCCCGAGGGCGGTGATGCTGCCGCGCCTGCCGCCCCCAAGGAGGAGGCC
CCCGCCGCGCCCAAGGCTGCCCCCGCCGCCCCCGAGCAGCCCAAGGCCGCCGCCCCCGCC
GCGCCCAAGGCTGCCCCCGCTCCCCAGGCCGCTCCTGCTGGCGGACGGGTGTTCGCAAGC
CCTCTCGCCCGCAAGGTGGCCCAGGAACAGGGCGTCGATGTCGCCGCCGTTCATGGAACC
GGCTCCAATAACCGCGTTATTCGCGCTGACGTTCTCGATTATGCCGCCAAGGGTCCGGCT
TTCGTGCCCGCCGCCACCTCGGTGCCCACGCCCGCTCCCGGCGGCCTGTTCACCGACATC
CCCAACACACAGATCCGCAAGGTCATTGCCGCTCGTCTTACCGAGTCGAAGCAGACCGTG
CCTCACTACTACCTGAGCATCGAGTGCCGCATGGACAAGCTCCTCAAGGTGCGCCAGGAG
CTGAACGCCAAGGGCGAGGGCGCGTACAAGCTCTCCGTCAACGACTTCATCATCAAGGCC
GCTGCCCTGGCCCTCCAGAAGAAGCCCACCTGCAACAGCGCCTGGTTCGGCGACTACATC
AGGCGGTACCACAACGTGGACATCAACGTGGCCGTGAGCACCGACGAGGGCCTGTTCACG
CCCATCGTGCAGGATGCCGACAAAAAGGGTCTGGCCACCATCGCCAACACGGTCAAGGAC
CTCGCCAACAAGGCCAAGGAGAAGAAGCTCCAGCCCCACGAGTTCCAGGGCGGTACCTTC
ACCATCTCCAACCTCGGCATGTTCGGCGTGAAGCAGTTCGCCGCCGTCATCAACCCGCCT
CAGTCGTGCATCCTCGCCGTGGGCGGTACCGAGAAGAAGGTGGTGCCCAACGAGGACAAG
GAGACCTCGGCCGCGCAGCCCTACGCCACTGCCCACGTGATGACCGTCACGCTCAGCTGC
GACCACCGCGTGGTGGATGGCGCCGTCGGTGCCGAGTGGCTCAAGACCTTCAAGGAGCTC
GTCGAGGACCCTGTCAAGATGCTCCTCTAA
>g1359-404961-407032
ATGCCCGCACGCGCCGGCCGTACGGGTCTCTGTGGGTCACACGCGCTGTGGCTCGCGGGC
TCTTGCGGTTGTGTACGGGCGGGGTGCGACTCCCATTCGGTGGTGAGGCCTCACTCGGGC
GCTGCGCGGGAGGAGGGGCCTTGTGAACTTGTCCGCCCCTTGGCGTGTGCAGGTAAGCAA
TGCTCCCTCCCTTCCAGCGGTTGTCGTGTGTGTCTCCCCTCTCCTCCAGTCCCGAGAAGA
GCCAGCGCCCACCTTTCTGTTTTTGTTGTTTTTTTTAAAAAATCACCATTTTTCGCGCTA
CACCACACTTGGGTACGCAATAAAATAGAAAATCCCGAGAGTAGAGACAAGCAAGTCCGT
TGTTGTTGTTCTCCTTCTCCCTTTCCACCCTCTTTAGCCATGGGAGAATACGCCGATGTA
GACAAATGGATCGAAACGCTCAGGCAATGTAAGCCACTCTCCGAAGCCAATGTCAAAGGG
CTGTGCGACAAGGCGCGAGAAATTTTGCAAGATGAGTCGAATGTGCAGCCTGTGCGCTGC
CCCGTGACCGTGTGCGGTGACGTTCACGGCCAATTCCATGATTTGATGGAGCTGTTCAAG
ATTGGAGGTGAAGTTCCCTCCACCAACTACCTCTTCATGGGCGACTACGTCGACCGTGGT
TACTACTCAGTTGAAACCGTCTCCCTTCTTGTAGCTCTCAAGGTGAGGTACCCCCACCGC
ATCACCATCCTCCGCGGTAACCATGAAAGTCGACAAATCACCCAAGTGTATGGGTTCTAT
GACGAGTGCCTTCGCAAGTACGGGAGCGCCAACGTGTGGAAGTTCTTCACGGACCTCTTC
GACTACCTCCCGCTCACCGCTCTCATTGAGCAACAAATTTTCTGCCTCCACGGCGGCCTT
TCGCCCTCCATCGATACTCTCGACCACGTCCGTCAGCTCGACCGAATCCAAGAGGTGCCC
CACGAAGGCCCCATGTGCGACCTCCTCTGGTCCGACCCCGACGACAGGACCGGCTGGGGT
GTCTCCCCGCGAGGAGCCGGCTACACTTTCGGGCAGGACATTTCCGAGCAGTTCAATCAC
AACAACGGCCTCAACCTCGTCGCCCGCGCTCATCAACTCGTCATGGAAGGCTACAACTGG
TGCCACGAGCAGAACGTCGTGACCATCTTCTCCGCTCCCAACTACTGCTACCGCTGTGGC
AACCAAGCCGCTATCATGGAGCTTGACGAACACATGAAGCACATGTTGTACGTAGCCTCC
CAATTTGCCCGCCCTGGCCACGCGCTGACCCAGACTCCTCCCCAACAACAGCCTCCAGTT
CGACCCAGCGCCCAGGCGGGGAGAGCCCCACGTCACGCGGAGGACACCAGACTACTTCCT
CTGAGTGTGTTACCAGTTTCCCCCCCCCCCAAAGGTGGGCGATGA
>g1359-460757-462431
ATGACCGACGCGCAGAAGAGGCTCAATGTGTTCCCCACCCGAATGACGCTTCAGCTCATG
AAGGGTAAGCTGAAGGGCGCGCAGAGGGGTCACGATCTTCTCAAGAAGAAGGCCGACGCC
CTGGCCATGCGCTTCCGCGTCATTCTCAAGAACATCAAAAAGAACAAAGCGGCCATGGGT
GCCATCATGAGGAAGGCCCACCTGTCGCTCGCCTCTGCCAAGTATGCCGCCGGTGAATTC
AGCACGTCGGTGATCGAGAACGTCACACAGGCCACGTTCAAGGTCAAGCTCGACGAGGAC
AACGTGGCTGGTGTGCACTTGCCCATCTTCAAGAACTACGCCGACATCTCCAACTTGCCG
AAGGAGCTGCACGGTCTCGGCAGGGGTGGTCAGCAGGTGCGGGAGGCCCGCGAGACCTAC
ATCAAGGCCCTCGACGCCCTCGTCGAGCTGGCCTCGCTCCAGACGGCCTTCATGACGTTG
GACGAGGTGATCAAGATCACCAACCGTCGTGTGAACGCCATCGAGTACGTGGTGCTGCCC
CGTATCGACAACACCATCAAGTACATCATGTCGGAGCTCGACGAAGGCGAGAGGGAGGAG
TTCTACAGACTCAAGATGATTCAGAAGAAGAAGGAACAGAGGAAGAAGGCCGCCGCCGCC
GCCAAGGGCGTGGACGTCGAAGACGAGGTGCCCTACCAGCAGCAGATCGAGCAGCCCTCG
GACTTGACCAACGAGGCCAGGGATCCCGACCTTTTGTTCGCCGACTAA
>g1359-547267-548239
ATGCCCAAGTTCCTCAAGCCCGGTAAGGTCGTGATCGTCCTCAACGGCAGGTATGCCGGC
AGGAAGGCTGTCATCGTCGAGAACCGCGACGACGGTACCAAGGCTCGCCCCTATGGTTAC
GCCGTCGTTGCTGGCGTCGACAAGTACCCCAAGAAGATCACCAAGTCGATGGGCAAGAAG
AAGATCGCCAAGAAGACCAAGATCGCCCCCTTCGTGAAGGCCATCAACTACAACCACCTG
ATGCCCACCAGGTACGGTCTCGATGTTGAGCTCAGGAGCATTGTCTGCAAGGAGAACGCT
CTCGAGAAGGGTCAGAACAAGTACAAGACCAAGCAGGCCCTCAAGAAGGTGTTCCAGACC
AGATACAACTCCGGCAAGAACAAGTGGTTCTTCACCAAGCTCAGGTTCTAA
>g1359-558623-559229
ATGGGTAAGGTCCACGGAGGTCTCGCGCGTGCCGGTAAGGTCAAGGGTCAGACCCCTAAG
ATCGAGAAGGTCGAGAAGAAGAAGACCCCCAAGGGTCGCGCCAAGAAGAGGATTCTCTAC
ACCAAGCGTTTCGTCAACGTCGTCGTCGGTCCCGGTGGCCGCAAGAGGGGACCCAACGCC
AACGTGCCCGCTGAGGACAAGAAGAAATAA
>g1367-54308-56288
ATGGATCGGGAGTATCTGTACAACTCCGTGCTGTATGTGCTGTACGAGTCCGCTTCAGGC
TACGCCCTCTTCGAGCGCATCGAATCGGAGGAAATCGCCGATGAGTCGCCGGAGCTGCAG
AGGTCGATCCAGGACTTCGGCCGTTTCTCGAGGATTGTCAAGTTCAAGTCTTTCGTGCCC
TTCGTCTCGGCCGAGAGCGCTCTCGAGAACGTCAACGCCATCTCTGAAGGTCTGGTGCAC
GACGTGTTGAAGAACTTCCTGGAGCTCAACCTGCCGGGCGGGGGCAAGAAGAAGAGCAAG
AAGGTGGAGCTGGGCGTGACGGACGAGAAGCTGGGCGGCGCCATCAACGACTCGCTGGAG
CACGTGGCATGCGTGAAGAGCAAGGCGGTGGCGGAGCTGACGCGCGGGATCCGGCTGCAC
TTTGCCAAGTTCATCAAGGAGTTCAAGGAGGGCGACTACGAGAAGGCGCAGCTGGGTCTG
GGCCACTCGTACTCGCGGAGCAAGGTCAAGTTCAACGTGAACCGGGCGGACAACATGATC
ATCCAGACCATCAACCTGCTCGACACGCTCAACAAGGACCTCAACACGTTCTCCATGCGG
TGCAAGGAGTGGTACTCGTGGCACTTCCCGGAGCTGGTCAAGGTGGTGCCGGACAACTTC
CAGTTCGCGCGCGTGGTCAAGTTCCTGAAGAACAAGGCCGAGGCCGACGAGAGCAAGATC
CCCGGCCTGGTGGACATCACGCAGGACGAGGCCAAGGCCAAGGAGATCATCGACGCTGCC
AAGGCCTCGATGGGCACCGACATCTCCGACCTCGACATGCTCAACATCGAGAAGTTCGCC
GACCGCGTGATCCACCTCTCCACCTACCAGCAGCAGCTCCAGGAGTACCTGAGCAAGAAG
ATGCACGTCATCGCGCCCAACCTCAGCGAGCTCGTCGGCGAGCACGTCGGCGCGCGCCTC
ATCTCGCACGCCGGCAGCCTCACCAACCTGGCCAAGTACCCGGCCTCGACCGTGCAGATC
CTGGGCGCCGAGAAGGCCCTCTTCCGCGCGCTCAAGACCCGCGGCAACACGCCCAAGTAC
GGCCTCATCTTCCACTCGTCCTTCATCGGCCGCGCCGCCGCCAAGAACAAGGGCCGCATC
TCGCGCTACCTGGCCAACAAGTGCTCCATCGCCTCGCGCATCGACGCCTTCTCCGACGTG
CCCACCACCAAGTTCGGCCAGAAGCTCAACGCCCAGGTCGAGGAGCGCCTCAAGTTCTAC
GACACCGGCGCCCTGCCCCGCAAGAACGTCGACGTCATGAAGGAGGCCCTCTCCGAGGCC
CTCACCGAGGCCGACCAGAAGCAGCGAGAGGACGGCATGGACGTGGTCTCCACCACCGCG
GAGAGCACCGACAAGAAGAGGAAGAGGAAGGCCGCGGAGAAGGACGACAGCGACAGCGAC
GAGGAGGAGAGCAAGAAGGACAAGAAGAAGAGCAAGAAGGCCAAGAAGTCCAAAAAGGAG
GAGGAAAAGAAGAAGAGCAAGAAGGACAAGAAGAAGAAGAAGTCCAAGCACTAA
>g1369-80088-81438
ATGGCCCAGAGCGACAGCAAGCTTACCCTCTACACCAACCCTATCTGCCCCTTCGCGCAC
CGCGCCCTCCTCACCGCCACCGAGAAGGGCCTTTCCTTCGAGACCGTCATCATCCCTCTT
GGCGGTGACAAGCCCGACTGGTACTTGAAGGTTAACCCCAGGGGCACCGTGCCCACGCTC
GTCCACGGAGAGCACACGATCCACGAATCTCTGTTGATTACCGAATACCTGGACGACGCC
TTCTTCAACGCCAGCCCTCGCCTCCTCCCCAGCGACCCGTACCAGCGGTACGCGTCGAGG
TTCATCGTGGACCAGTTCGGCTCTCAGGTCATCCCCGCGCTGTACCAGCTCCTCAGGAAC
CAGGATCGGTCCCAAGACGACAAGATCAAGGAGGAGATCACCAAGAAGCTGAAGGCGCTT
CTCGACCTCTACTCCGCGCAGGCTGGCGAAGGCCCCTACTTCCTGGGCCAGCACATCTCT
CTGGCTGATGTGGCCATTCTGCCGTTCATCGGCCGCTTCGCCATCTCGCTGCCGCACTAC
CGCGACTTCGACGTGCTGGCCGTTGACGAGCGCCTCAAGAAGTGGCACGACGCCTTCGTG
AGCCGCCCGTCGTGGCAGCAGACCTTCCCCCCCACCGCCGACATCCTCCAGGGCTACGCC
AAATACGCCAACCCCACCCAGTAG
>g1369-181606-186430
ATGGATAAGCCGGTGATTGTAAAGGAGAGCACCGATTCTGAGGGTGTGGACACTGATCTC
ATGTCTCGCATCATCCCCATCTTGGGTGCCGGCAACTTGAAGAAGCTTATGGGCCTGAAG
GTCCTCATCTCGGGCATCAGTGGCCTCGGTGCCGAGATCGCCAAGAACCTCATTCTCACT
GGTCTGGGTGTTGTCACCATCCACGACACTGAGAAGGTCGACTGGATCGATCTTTCCTCT
CACTTCTACCTCACTGAGGCGGATATTGGAAAGAACCGGGCTGAGGCTTCCAAAGCCAAG
CTGGCCGAACTCAATCCCTACGTCAAAGTGAACCTTTCCACTGAGCCCCTCACGGACGAC
TACCTCAACCAGTTCCAGGTCGTGGTGTGCGTCGACTACACTTCGGAGGAGAAGCTGCTG
CACGTCAGCGAGTACTGCCATGCGCATGACCCCGCCATTGTCTTCATCAAGGCCGATATC
CGCGGTCTCTTCTGCAGCGTCTTCTCTGATTTCGGACCCAAGCACGTCATTTACGATAAG
ACTGGCGAGGAGCCCAGGCAGGCCATCATCACCTCCATCTCCAACAGCAACCCTGCTGTG
ATCACCACACACGAGGAGAAGCCTCACGGTCTGGGCGAGGGCGACTACGTCGAGTTCTGC
GAGGTCAAGGGCATGGTGGAAATTAACAATCCCGACAGGGACAGCGAAGAGGCTGGCGAG
AAGAAGGAGGTCAGCCCCCTCGCCGCCGTCAAGGTCCTCTCGACTAAGGGTCTTTACGGT
CTTGAGGTTGAGCTGGACACCACCAACTTCCAGCCCTACAGCGGCGGTGGCCTGATCAAC
CAGATCAAGGTTGAGGAGCACGTGTCCTTCAAATCCTATCGTGAGTCGCTGGAGCACCCG
GGCGAGTTCATGATCTCCGACTTTGCCAAGTTCGGTCGTGCCGAGCAGCTGCACTTTGGC
TTCCAGGCGCTGCACAAGTTCCAGGCCAAGCACTCGGCTCTGCCCGAGCCCGGCAATGCC
GAGCACGCCGCCGAGGTCGTCCAGATCGCCAAGGACCTCAACGCCAACGCCAATCAGGGC
ACCCACAAGGTCGAGGAGATCGATGAGAACCTCATCTCCAAGCTGGCCCTCACTGCTCGC
GGTAACCTCAACCCGATGGCCGCCTTTGTCGGTGGTATCGTGGCCCAGGAGGTGATCAAG
GTCACGGGCAAGTTCAACCCCGTCACCCAGTGGTTCTACTTCGACTCGCTTGAGTGCCTT
CCCGAGCAGCCCGTCAGCGTGCCCAAGCTCGAAGGCACCCGCTACGACGGCCAGATCGCG
GTCTTTGGCACCGACTTCCAGAAGCAGCTGGGTAACCTCCAGCTGTTCCTGGTCGGTGCT
GGTGCTCTTGGTTGCGAGTTCCTCAAGAACTTTGCGCTGATGGGCATCTCCGCGGGCGAG
GAGGGCCTGCTCTCGCTCACCGACATGGACAACATCGAGAAGAGCAACCTCAGCAGGCAG
TTCCTCTTCAGGGACTCGGACATCGGTAAGATGAAGTCAGCCTGCGCCTCGGCCGCCGCC
AAGAAGATGAACCCCAACCTCCGCATCAAGGCCTCGGAGGTGCCCGTGGGCGAAGACACC
GAAGACACCTGGAATGACACCTTCTGGTCGGGCCAGGACCTCGTGGTCAACGCCCTCGAC
AACATCAAGGCTCGTCTCTACGTTGACTCGCAGTGCGTGCGCTACCTCAAGCCGCTCCTC
GAGTCCGGTACCCTGGGCACCAAGGCCAACTCGCAGGTCATCGTCCCCCGCATGACCGAG
TCCTACGGCTCGTCCCGCGACCCTCCCGATACCGCCATCCCCATGTGCACCCTCAAGAAC
TTCCCTCACCAGATCGAGCACACCATCGAGTGGGGGCGTGACAAGTTCGCGGGCTACTTC
ACCAACGCCGTCGAGGACGCCAACAACTGGGTCTCGGGCAGCGACTTCTTGGACAGGATC
AAGCAGGTCGAGTCCTACGCGGCCAAGAAGGAGAGGCTTCAGTCGTGCCTGCAGCTGCTG
AAGCTGTACAACTACGGCAAGGCCGACTTCCAGACTTGCGTTGAGTGGGCTCGTCTGCAG
TTCGAGGAGCTATTCCACAACACCATCGCCCAGCTGCTCTACAATTTCCCTCTCGATGCC
ACCACCTCCACCGGCGCGCCCTTCTGGTCGGGTCCCAAGCGTCCCCCCACGCCTCTTAAG
TTCGACCCGAACAACGCCACCCACCTGGACTTCATCATTGCCGCTGCCAACCTCCTGGCC
TTCAACTTCCACGTCCCGCAAGTCAGGGATAAGGATCAGGTCAAGGAGATGGTGGGCAAG
GTGCACGTGCCTCCCTTCTCCCCTCAGCAGGGAGTCAAGATCAAGTCCGGCGAAACCGAC
ACCACCGAGGAGGGAGCCGAAGACGACGAGCAGAAGGTCGCCAACCTGATCGCCGAGTTG
GGCCAACTCGACAAGGCCAAGTACCCCGTCGGCGAGAGCGGTCGTTGCTTCGAGCCGGCT
CAGTTCGAGAAGGACGACGACTCCAACTACCACATCTCCTTCATCACCCAGGCGTCGAAC
CTGCGTGCGGCCAACTACAAGATCCAGCCGGCCGACTTCCACAAGACCAAGAAGATCGCT
GGTCGCATCATCCCGGCCATCGCCACTACCACGGCCATGATCACCGGCCTCGTGGGCCTC
GAGCTGTACAAGGTCGTCCAGGGCGCGTCGGTGCCCATTGAGCGCTACCGCAACTCTTTC
GTCAACCTGGCGCTGCCCTCGTTCGTCCAGTCGGAGCCCATGCCCTGCACCAAGAACAAG
TCCGACCCGGCCAAGGGCCTCAAGTACTACCCCGAGGGCTGGACCCTGTGGGACAACTTC
GTCATCGACGAGGGCGACATCACGTTCCAGCAGCTCCTCGATCTCTTCAAGGCCAAGCAC
AACCTTGAGGTGACCAGCGTGAGCTGCGGCACCACGCTCGTGTACAACCCGTACTTCCCC
AACCACAAGAACCGTCTCGGCACCAAGATCTCCGAGTTTGTGCGCACGAGTGTCCCCTCC
TACGACCTCAAGGACACCGACAAGCACATGTACATTGTGGTCCTGACCGAAGACGAGGAG
GGCAATGACGTGGAGATCCCCGATCCCGTCATCCTCAAGTTCAAGTAA
>g1369-260336-261344
ATGTACACGGCTCGCAGAAAGATCCAGAAGGAAAAGAACGCCGAACCCACCGAGTTCGAG
CTTCAGGTCGCCCAGGCCCTGTTCGACCTCGAGGTCAACGCCACTGATCTCAAGGGTGAC
CTCAAGGAGCTGTTCATCAGCGGCGCCAGGCAGGTTGACCTCGGTGCTGGCAAGACCGCC
ATCCTCGTCTTCGTGCCCTTCCGTCTCCTCAAGGGCTTCCACAAGGTCCAGGCCCGTCTT
GTCCGCGAGCTCGAGAAGAAGTTCAGCGGCCAGCACGTCGTCGTGATCGCCAACCGTCGC
ATCCTCCCCGTCATCGGCAAGAAGAACAGGAGGAAGAGGCAAAAGAGGCCCATGAGCCGT
ACCCTCACCGCTGTACATGATGCCATCCTTGAGGACCTCGTCTACCCCACCGAGATCACT
GGCAAGCGCATCCGCATCAGGCTCGATGGCTCCAGGCTCTACAAAGTGTACCTCGACAAG
AAGGACCAGAACAACGTTGAGTACAAGCTCGACACCTTCTCGAGGCTCTACAAGAAGCTC
ACCGGCAAGCAGGTCGCTTTCCTGTTCTAA
>g1369-333938-335190
ATGACCCGCCTGTCGACCATGCACGCTGACATGTCGGGCGACATCACCTCCAGGAGCTCG
TCGTCGGGCACCTCAGGCGGCGCCCGGCTGAAGTTCGAGGTGGCGATCGCGTTCACCGAC
ATCACGATGGTGCTCAGAGCCCAAGGTGGTGCGCGACCGTCGGGTGGAGTACGTCGGCCT
GCGGTGAACTTGGCGGCGCTCATCACTGCGCTCACCCACGGTGACCAGATCGTCATGAGC
CACGCCAAGCTCGGTGTGGCCTTCCTCGACAGCGCTAACAACCGAGTTACCTGCCTGGGC
AAGTTCGACACCCCGGGCGTTCATTCATTCACGACCGCCGTCAACAAAAACCTCTCACAG
ACCACTCCACAACAACTCCCCTTCAAATCAACAATGTCGTGGCAGACGTACGTCGATACC
AACCTCGTGGGCACCGGTGCCGTGACCCAGGCCGCTATCCTCGGCCTCGACGGTAACACC
TGGGCCACCTCGGCCGGCTTCGCCGTGACCCCCGCCCAGGGCACCACCCTCGCCGGTGCC
TTCAACAACGCCGACGCCATCCGCGCTGGTGGTTTCGATCTCGCTGGTGTCCACTACGTG
ACCCTCCGCGCTGATGATCGTTCCATCTACGGTAAGAAGGGCGCCGCCGGTGTCATCACC
GTCAAGACCTCCAAGGCCATCCTCGTGGGCGTCTACAACGAGAAGATCCAGCCCGGTACC
GCCGCCAACGTCGTCGAGAAGCTCGCTGACTACCTCATCGGCCAGGGTTTCTAA
>g1369-335797-338290
ATGAGCTTCAGCGGCAACGAGTCGGTCGCCACCAAGAACCGAAAGGAGAAGCAGGCCGAG
ATTGAGAGGCTTCACGAGAAGACCTTCAAGGCCTGGATCAACGCCCAGCTGCGCGTGCGC
AACATCAAGATCGAGGGCGAGCTCAAGGACGAACTCAAGGATGGTCTGGCCCTCATCAAC
CTGATGGAGGTCCTCAGCGGCGAGAAGTGCCCTGAGAAGTACAACACCAAGATCACCCTC
GACATGCACCGCATTGAGAACACCACCATCGCCATCAACTTCATCACCAAGCACGTCGGC
AAGCTGAGCGTCTCTTCCACCGATGTCCAGCAGGGTAACATGCGTATCATCCTCGGAATG
ATCTGGCGCGTCATTCTCACCTTCAAGGTCGAGCGTCGCGGTGAAGGCGACGCCGACGAC
AAGGAGCTCTCCGCCGCCCAGAGGAACCGACAGGCCAAGAAGAAGCTGCTCGACTGGTGC
GTGGAGAAGACCAAGGGACACAACGGCGTCGACATCAAGGACTTCGGCGAGTCGTGGTAT
GACGGTCTGGGCTTCTGCGCCCTCATTCACGCGTTCGACCCGTCGCTCATCGACTACGAG
TCGCTCAAGGCCGAGAACGCTCAGGCCAACCTCGAGCTGGCCTTCGAGCTCGCCGAGAAG
CACCTCGACATCCCGCGCCTGCTCGACCCCGCTGATATCTGCGCCGACGATGAGATGTCG
CGCCCCGACGAGCAGTGCTTCATGACCTACCTCTCCGAGTTCCCCATCGCGTTCCTCGCG
GGCAAGGAGAAGGCCGACGACCGCGCCGCGATCGAGGCCGAGGCCAAGAGGAAGGCCCAG
GAGGAGGAGGAGCGCAAGAGGCTCGAGGCCGAGCGCCAGCGCGCCGAGGAGGAGCGCCTG
AAGGCCGAGGCCGAGAAGAAGAGGCTCGAGGAGGAGAAGGCTCGCCTCGAGGCCGACAAG
GCCGCCGCCGAGGAGGAGTCCAAGCGAAGGAAGGAGGAGCTCGAGGCCCAGTCGGCCGCG
CTCAGGCAGCTCGAGGAGGAGCGCAAGAGGAAGGACGAGGAGGAGGAAGAGAAGAAGAAG
AAGCGCGAGGAGAAGAAGAAGCGAAGGCAGCAGGAGGCCGACGAGGCCGAGGAGCGTGCC
AAGCGTGAGGCCGAGGAGGCGTCGCGTCTCGCCCGCGAGGAGGCCGAGAAGGAGGCCAAG
GAGCGCCAGGAGGAGCTCGAGAAGCGCCAGGAGGAGGACGACGCCAAGCGCCAGGAGGAG
CTCTCCAAGCTCGAGGCCCAGCACGAGGCCGAGCGTCTCAGGCTCGAGGAGGAGTGCCGC
CGGCTCAAGCAGCAGCTCGAGGCCACCAAGGGCAAGCTCATCGGCAAGCTGAAGGTCACC
GTCAAGGAGGGCCGCGGCGTGCACAAGAAGGACAACTCCGGCAAGGCCGATCCCTACTGC
GTGCTCTTCCTCGAGAGGCAGAAGGAGAAGACCAGGACCATCAAGAAGAACCAGAACCCC
AAGTGGGACGCCGACTTCGAGTTCTACGTCTCCGACCCCGAGGCCGCCCTCGAGGTCACC
ATGTTCGACTGGAACCGCATCTTCTCGGACTCCTTCCTCGGCAAGGTGTCGATCCCCATC
GCCACCCTCAACGACGGCGAGGAGACCACCGCCTGGTACAAGCTCGAGGGCAAGAAGGCC
AAGGACAAGGTCACCGGCGAGCTCTGCCTCACCATCCTCTACAGGAAGGAGGTGTAA
>g1369-497445-500306
ATGGCGGAACAGAATAAGACCTGGGTTGATGTCCAGAAGAAGGCCTTCACCCGCTGGGCC
AACCAGTTCCTCTCGGAGAGGCGCCTGAAGATCGAGAACATCGAGACCGATCTGGCCACT
GGTATCAACCTGTGCAACCTGCTCGAGATCATCTCGTCCAAGTCGCTCGGCAAGTACAAC
CACAAGCCCACCATGCGTTACCACTACCTCGAGAACAACGGCCGCGCCGTCAAGTTCATC
AAGGACGAGGGCCTCCAGCTCGTCGGTATCGGTCCTGAGGATATCGTGGACGGCAAGCTC
AAGCTTATCCTGGGTCTCATCTGGACGCTCATTCTGCGTTACCAGATCAACATGATCGGC
GAGGGTTCGCCCAAGTGGGAGCTCCTCCAGTGGGTGAACAAGCAGATCGCCCCCTACAAC
GTCGACAAGCCCGTCGTCAACTTCACCACCAACTGGTCCGACGGTAAGGTGCTCAGCGCT
CTGGCTGACTCGCTCCAGCCGGGCGTGCTCACCCCGACCGACATGTCGGCCCTGTCCGGC
GACGCCCTGCAGGATCTCGAGAAGGCCATGCAGACCGCGCTCGACAACTACAACATCGCC
AGGCTCATCGACCCCGAGGACATGGTCAACTGCCCCGACGAGCTGTCGATGATGACCTAC
GTCTCGGCCTTCCGCAACTACCTCAGCGAGGAGGAGCAGAGGCGCCGCGAGGAGCTGGCC
CGCAAGAAGAGGACCGCCGACCCCTCGAACTGCTACGCGTACGGCCCCGGCCTCGAGGGT
GCCGACACCTACCAGCCGGCCCACTTCACCATCGTGGCCAAGAACTACTTCGACGAGGAG
CTGCCCACCGGCGGCGACCAGTTCGAGGTGACCATCGCCGGCCCCGACAGCAACGTGCAG
CCCACCGTCACCGACAACGGCGACGGCAAGTACCCGGCCCAGTACACCGTGGCCAAGCCC
GGCGACTACACCATCACCATCAAGCTGCGCGATGAGCCCATCAAGGGTTCGCCCTACCAC
GTGCACGTCGACGGTCCCAACGCCGGCCACTCGGTCGCCTCCGGCCCCGGTGTCGAGGGC
GCCCAGACCAAGAAGCCCGCCAACTTCCGCATCACCTCGTTCAACGACAAGGGCCAGCAG
GTCCAGTCCGGCGGCGACAAGTACCAGGTCCAGGTCCAGGGTCCCGAGGACGTGGCCGAC
CCCAGCCTCACCGACAACAACGACGGTACCTTCGACGGTGCCTACCAGGTGTCCACCCCC
GGCCACTACTTCGTCAACATCACGCTCGACGACGAGCCCATCAAGGGCAGCCCCTACAAG
GTGCTCATCGAGGGCGCCCGCGCCGGCAACTCGTACGCCGAGGGTCCCGGCCTCGAGGGT
GGCCAGGCCACCAAGCCCAGCGTGTTCACCATCCACGCCTACGATCCCGAGGGCCAGAAG
TGCACCGACGGTGGCGACCCCTTCAAGGTCGACATCCAGGGTCCCGCCGACGTGCAGCCC
ACCGTGACCGACAACGGCGACGGCACCTACACCGTTGAGTACACCCCGACCGAGGCCGGC
GACTACACCGTCAACGTGACCCTCCACGACGAGCCCATCAAGGATGTGCCCCGTCAGGTC
CACGTCAAGGCCACCCCCGACGCCTCCAAGACCTGGGCTGAGGGTCCCGCCCTCGAGGGC
CTCGTTGATAACGAGCCCGGCCTCTTCACCATCCACGCCGTGGACAAGGACGGCAACCCG
CGCGTTGACGGCGGCGACAAGTTCGACGTCGACATCAAGGGCCCCAACGGCAACGTCCCC
GCCGACGTCACCGACAACGGCGACGGCACCTATGGTGTCAAGTTCGACCCCGAGAACCCC
GGTGACTACGACATCAACGTCACCTTCGAGGGTGCCCCCATCAAGGATGCCCCCTTCCAC
GTCCACTGCAAGGAGGGTACCGACGCCGACGAGTCGGGCTTCGGCATCTTCTCCTTCACC
ATCCAGTCCCGCGACAAGCGTGGCGAGAACAAGACCTTCGGCGGCGACGCCTTCGACGTT
GCCATCAAGGGTCCCGACGATTCGGACGTCGAGGTCCAGACCACCGACAACAGCGACGGT
ACCTACACCGCCGTCTACGCTCTTACCGGCGACAAGGGCGCCGTCTTCACCATCACCGCC
AAGCTCAACAACCGGAAGGTCGGTACCTACAAGCAGAACCTCGACTAA
>g1369-729197-730236
ATGACTGAGTACAAGCTTGTCGTTGTCGGAGGCGGTGGCGTGGGTAAGTCGGCGCTCACC
ATCCAGCTGATCAACCATCACTTCATGGACGAGTACGATCCTACCATCGAAGATTCCTAC
CGCAAGCAGGTCGAAATCGACCAGGAGACCTGTCTCCTCGACATTCTGGATACCGCCGGC
CAGGAGGAATTCAGCGCTATGAGGGATCAGTACATGCGTACCGGCCAGGGCTTCCTCTGC
GTCTACTCTATCACGAGTCGATCGTCGTTTGAGGAAATCTCTTCCTTCCGCGAGCAAATC
CTCAGGGTAAAGGAGGAGGACAACGTGCCGATGGTTCTTGTCGGTAACAAGTGCGATCTC
GAGGACTCGCGAGTGGTCGCCACCTCCGAGGGTGCCGATCTCGCCAAGTCGTTCGGCTGC
AAGTTCCTCGAGTCGTCGGCCAAGTCGCGCATCAACGTCGAGGAGTCCTTCTTCGAGCTC
GTCCGCGAGATCCGCAAGTCCATCGGCGGCGATTCCGACAGCGGCAAGGGCAAGGGTAAG
GGAAACAAGGGCGGCAAGGGCAAGGGCATGAAGGGCAAGCTCAAGTTCTCTTCCAACCAG
TGCTCCCTCTTCTAA
>g1369-730493-733156
ATGGCTGATAAGCAAAAGGAGGACCATGGCGACATGAGCGAGGAGATCCTCAACATGAAG
AAGAGGCCCAACAGATTGATGGTCGACGAGGCGACCAACGACGACAACTCGGTTGTGGCA
CTTCATCCGAAGAAGCTCGAGGAGCTGCAGCTGTTCAAGGGCGACACAGTGCTGCTCAAG
GGCAAGCGTAGGAAGGACACAATCTGCATCGTTCTCTCGGACGAGACGTGCGAGGAGGGC
AAGATCAGAATGAACAAGGTGGTGCGAAACAACCTGCGCGTGCGGCTGTCCGACATCGTG
TCGGTGCACGCCTGCCCTGACGTCAAGTATGGCAAGCGCATCCACGTGCTCCCGCTGGAG
GACACCATCGAGGGCCTCTCGGGCAACATCTTCGATCTCTTCCTCAAGCCCTACTTCCTC
GAGGCCTACCGACCGGTGCGCAAGGGCGACACCTTCGTCGCGAGGGGTGGCATGAGGGCG
GTAGAGTTCAAGGTCGTAGAGACCGACCCTGCCGAGTACTGCATCGTCGCGCCCGACACC
GTCATCCACTGCGAGGGCGAGCCGATCAAGCGCGAAGATGAGGACAAGCTCGACGAGGTG
GGCTACGACGACATCGGTGGCTGCAGGAAGCAGCTGGCGCTCATCAGAGAACTGGTGGAG
CTGCCCCTGCGTCACCCGGCCCTCTTCAAGAGCATCGGCGTCAAGCCCCCCAAGGGCATC
CTCATGTACGGTCCGCCCGGCAGCGGTAAGACCCTCATCGCTCGCGCCATTGCCAACGAG
ACTGGCGCCTTCTTCTTCGTCATCAACGGTCCCGAGATCATGTCGAAGCTCGCTGGCGAA
TCGGAGAGCAATCTCAGGAAGGCCTTCGAGGAGGCCGAGAAGAACGCCCCCGCCATCATC
TTCATCGACGAGATCGACGCCATCGCGCCCAAGCGTGAGAAGACCAACGGCGAGGTGGAG
CGCAGGATCGTCTCGCAGCTGCTCACCCTCATGGACGGCCTCAAGTCGAGGGCTCACATC
ATGGTCATCGGCGCCACCAACCGACCCAACAGTATCGACCCGGCCCTCAGGCGCTTCGGC
CGCTTCGACAGGGAGATCGACATCGGTATCCCCGACCCCACTGGCCGACTCGAGGTGCTC
GCCATCCACACCAAGAACATGAAGCTCGACGACAACGTCGACCTGGAGCAGGTCGCCAAC
GAGACCCACGGCTTCGTCGGAGCCGATCTTGCCCAGCTCTGCACGGAGGCGGCCCTCACG
TGCATCCGCGAGAAGATGTACCTCATCGATCTCGAGGACGAGACCATCCCGGCCGAGGTG
CTCGACTCGCTCAGCGTCACGATGGACCACTTCAGGTCGGCCCTCGGCGTGTGCAACCCC
TCGGCCCTGCGCGAGACCGTCGTCGAGACGCCCAACACCACGTGGGAGGACATCGGTGGC
CTCGAGAACGTCAAGCGCGAGCTCAAGGAGCTGGTGCAGTACCCCGTCGAGCACCCCGAG
ATGTACCACAAGTTCGGCATGGAGCCTTCCAAGGGCGTCCTCTTCTACGGCCCTCCCGGT
TGCGGTAAGACGCTCCTCGCCAAGGCCATCGCCAACGAGTGTCAGGCCAACTTCATCTCG
ATCAAGGGTCCCGAGCTGCTCACCATGTGGTTCGGTGAGTCCGAGTCGAACGTGCGCGAC
GTCTTCGACAAGGCCCGCCAGGCCGCGCCCTGCGTGCTCTTCTTCGACGAGCTGGACTCC
ATCGCCAAGGCTCGCGGTGGTTCCTCCGGCGACGCCGGTGGTGCCGGCGACCGCGTCATC
AACCAGATCCTCACCGAGATGGATGGTATCGGCAAGAAGAAGAACGTCTTCGTCATCGGC
GCCACCAACAGGCCCGACATCATCGATCCCGCCATCCTGCGTCCGGGTCGTCTCGACCAG
CTGATCTACATCCCGCTGCCCGACCTCGGCTCTCGCATGGCCATCCTCAAGGCCGCTCTC
AGGAAGTCGCCCGTCGCCAAGGACGTCAACCTCGACTTCCTCGCCCAGCACACGCACGGC
TTCTCCGGTGCCGATCTGACCGAGATCTGCCAGCGGGCCGTCAAGCTGGCCATCCGCGAG
TCCATCGACATCGTCTACCAGCGCAGGAGGGAGAAGGAGGAGAACGCCGAGAGCATGGAG
ACCGAGGTCGAGGAGGAGGAGGACCCCGTCCCCGAGATCACGCGAGCCCACTTCGAGGAG
GCCATGAAGTTCGCCCGCCGCTCCGTCAGCGACAACGACATCAGGAAGTACGAGATGTTC
GCCCAGACCCTCGTCCAGTCGCGTGGCATTGGCAACAACTTCAAGTTCCCCGAGCAGAGC
CAGTTCTCCACGCCCGAGAACGACGAGGACCTCTACGGCAACTAA
>g1369-776435-777066
ATGCGAATTATTGCGGCTTACCTGCTTGCAGTTCTTGGCGGGAACTCCTCCCCCTCTGGT
TCTGACATCAAGAAGATCCTCGAGTCTGTCGGCGTTGAGGCTGAGGATGAGAAGATTGAG
CTCCTCGTCGGCGAGCTGAGCGGCAAGGACGTCTTCGAGGTCATCGAGGAGGGCAAGAAG
AAGCTCGGTGCCGTGCCCGTCGGTGCTGCTGCCCCCGCTGCCGCCGCTGCTGCTGCCCCC
GCCGCTGGTGCTGCCCCCGCTGCCAAGGCCGAGGAGAAGAAGAAGGAGGAGGAGGAGGAC
GATGACGTCTTCGGTGATGGTGGTGGTGATGGTGGTATGGGTCTCTTCGGCGGCGATGAG
GAGGACTACTAA
>g1369-777164-778988
ATGGCATTCCTGTTCGGCTCGCCGATTGTGGTGACAATCTTGCTTGATGGTGTGGAAGAG
AGGAAAACCATCGACTTCACCACCGAGAACGGATCAGAAAAATTGCCCCTGTACATTGGG
GACGAGACCGTGAAGGGTGTTGCCAAAGTCACGTGCAAGGAAGGAAAGAAGTATGAGCAC
CAGGGAATCAAGGTCGACTTTATCGGGCAAGTTGAGATGTTCTACGAGCGAGGCAGCAGC
CACGAATTTGTTTCGCTATCGAAGGAGCTCGCCGCTGCGGGAGAGATCACAGGCGTGAAC
GAATTCCCCTTCGAATTCTCCAAGGTGGAGAAGCAGTACGAGTCGTACCACGGCATCAAC
GTGCGCCTCAGGTACTTCGTGCGCGTGACAGTGACGCGGGGCCTGGCGTCGACGGTTACG
ACCGAGAAGGACATTTGGGTGCACAACTACCAGGTGAACCCGGACGTCAACAACCCCATC
AAGATGGAGGTCGGTATCGAGGACTGCCTTCACATCGAGTTCGAGTACAACCGATCCAAG
TACCATCTCACTGATGTCATCATCGGCAAGATCTATTTCCTGCTCGTGCGCATCAAGATC
AAGCACATGGAGATCGCCATCATCAAGCGAGAGTCGACCGGCTCTGGACCTAACCTGTAC
AACGAGAGCGAGACCATCACCAAATACGAGATCATGGACGGTGCCCCTGTCCGAGGCGAG
TCAATCCCCATCCGTCTCTTCCTGACGCCGTTCGAGCTCACCCCCACCTACCGCAACGTC
CACAGCAAGTTTTCCCTCAAGTACTACCTTAACCTCGTTCTCGTGGACGAGGAGGATCGT
CGCTACTTCAAGCAGCAGGAAATCGCCCTGTGGCGGAAGGCCGACAAGACGAGAACGAAG
ACCGGCGAGAGGACGCGTCAGCACAAGAGGTACGAGGGCAAGCGGGACAAGGAAGCGGCC
GAAAGGGAGAAGGCCGGCATCAAGGAGCCCAAGAACGACGACGAAGAGCACGAGTACTAG
>g1369-891692-892646
ATGCTGAGGCAGGCCACCGCTCGCACCGCTCCCCTGTTCGCCGTACGCGGCACCGTCACA
GGCCTTCAGGTCCTCAGCCGCTCGCCGGTGGCGCTGGCGACTCGCACCCTCGCCACCCTC
CCCTCCGATGCCTCGACGGAGGCCAAGCCCAACAGGCCTCTTTCGCCCCACGTCTCCATC
TACAGGTTCCCTCTGCCCGCCTTGACCTCGATCACCAACCGCGCCACTGGTGGTGCCCTC
ACCGCCGGTATCTACACTGCTGGTGCCCTCGCTCTCTTCGGTGCTCACGATCTTCCCGTC
TACATCGACGCCTTCAAGGCTGCCGTGCCCCTCCTTGTCTACCCCACCAAGCTCCTCGTC
TCTTTCCCCTTCGTCTACCACACGCTGGCCGGCATCAGGCACTTGTACTGGGACTACACG
GCCAAGGGACTCACCCTCCCCGAGGTCTACACCTCGAGCTACGCGCTCATGGGCGCCACG
GCTCTGCTCACCCTCGGCCTCACCTTCTACTCGATCTGA
>g1369-918275-921006
ATGGCAGAGGACGTGGAGACGTTTGCCTTCCAGGCTGAGATCAACCAGCTGCTCAACCTC
ATCATCAACACCTTCTACTCGAACAAGGAGATATTCTTGCGCGAGCTCATCTCCAACGCG
TCGGACGCCCTCGACAAGATCCGCTACCTCGGCCTCACCGACAAGCCCGCGCTCGAGACC
AACCCGGAGCTCTACGTGCACCTGGTGCCCGACAAGGCCAACAAGTGCATCCACATCATC
GACTCGGGCATCGGTATGACCAAGGCCGACCTCGTCAACAACCTGGGCACCATCGCCAAG
TCGGGCACCAAGGCCTTCATGGAGGCCCTCCAGGCCGGCGCTGACGTCAGCATGATTGGT
CAGTTCGGCGTGGGCTTCTACTCGGCGTACCTGGTCGGCGATCGGGTGGTGGTGACGACC
AAGCACAACGACGACGAGCAGTACGTGTGGGAGTCGGCGGCCGGCGGCACCTTCACCATC
AAGCGCGACACCGAGGGCGAGCCCCTGAAGCGCGGCACCAAGATCACGGTCTACCTCAAG
GAGGACCAGCTCGAGTACCTCGAGGAGCGTCGGCTCAAGGACCTCGTCAAGAAGCACTCC
CAGTTCATTCAGTACCCGATCTCGCTCTGGGTCGAGAAGACGAAGGAGAAGGAGGTCGAG
GAAGAAGCTGAAGAAGAAGAGGAGAAGAAGAAGGCCGAGGAGGAGACTACGGCGGAGGCG
CCCAAGATCGAAGAGGTGACCGAGGAGGAAGAAGAGAAAAAGGAGAAGGAAAAGAAGAAG
AAAAAGGTCAAGGAGACGTACCACGAGTGGGAGCTGCTCAACAAGACGAAGCCGATCTGG
ACGCGCAACCCGCAGGAGGTGTCCGAGGACGACTACAAGCAGTTCTACAAGAACCTGACC
AACGACTGGGAGGACTACCTCGCGGTGAAGCACTTCTCGGTCGAGGGCCAGCTCGAGTTC
AAGTCGATCGTGTTCGTGCCCAAGCGCCCGCCGTTCGACCTGTTCGAGACCCGCAAGAAG
CGGGCCAACGTCAAGCTCTACGTGCGTCGAGTCTTCATCACCGACGACTGCGAGGAGCTC
GTGCCCGAGTGGCTCAACTTCGTGCGCGGCATCGTCGACTCGGAGGACCTGCCGCTCAAC
ATCTCGCGCGAGATGCTCCAGCAGAACAAGATCCTGCGCGTCATCCGGAAGAACCTCGTC
AAGAAGTGCATCGAGCTCTTCAACGAGATCGCCGAGAAGAAGGAGGACTTTGACAAGTTC
TACGAGGCCTTCGGCAAGAACATCAAGTACGGCATCCACGAGGACTCGACCAACCGGACG
AAGCTGGCGGAGCTGCTGAGGTTCCATTCGACGAAGTCGGGCGCGGAGATGACGTCGTTC
AAGGACTACGTGACGCGGATGAAGGAGAACCAGAAGGAGATCTACTTCATCACGGGCGAG
ACCAAGAAGGCGGTCGAGAGCGCGCCGTTCGTGGAGGGCCTCAAGAGGAAGGGCTACGAG
GTGCTGTTCATGGTGGATCCCATCGACGAGTACATGGTGCAGCAGCTCAAGGAGTACGAC
GGCAAGAAGCTGGTCAACATCACCAAGGAGGGCCTCAAGCTCGACGAGACCGAGGAGGAG
AAGAAGAAGGCCGAGGAGACCAAGAAGGCCAACGAGAACCTGTGCAAGGTCGTCAAGGAC
ATCCTCGGCGACAAGGTCGAGAAGGTGGTGATCTCGAACAGGCTGGTGGACTCGCCGTGC
GTGCTGGTGACGGGCGAGTTCGGGTGGTCGGCCAACATGGAGCGGATCATGCGCGCCCAG
GCCCTGCGCGACAGCTCGATGCAGACCTACATGGTCTCGAAGAAGACGCTGGAGATCAAC
CCCGACCACGCGATCGTGACCGAGCTCAGGAAGAAGGCCGACGCCGACAAGAACGACAAG
ACCGTGAAGGACCTCGTGTGGCTCCTGTTCGACACGGCCCTGCTCGCGTCGGGCTTCTCG
CTCGAGGAGCCGGGCGGCTTCGCCCAGCGCATCCACCGCATGATCAAGCTGGGCCTCAGC
ATCGAGGACACCGAGAGCGACCGCGTCATGGGCGACGACGACCTCCCGCCGCTCGAGAGC
GAGGAAGCCAGCGCTGCTGATGAGGGCTCCAGGATGGAGGAGGTCGACTAA
>g1377-45590-48558
ATGGTGCTCTACAACTTCAAGCAGATCCAGTCCGTGCCTACGGGCCAGGACTTCATCGAT
ATCGCTCTCTCGAAGACGCAGCGGAAGACGCCGACGGTGGTCCACAAGCACTATGCCATC
GCGAGGATTCGCCAGTTCTACATGAAGAAGGTCAAGTTCACCCAGCAGACCTACCACGAC
AGGCTCACCCAGATCCTCGAAGATTTCCCCGTGCTGGATGACATCCACCCGTTCTACGCT
GATTTGATCAACGTCCTGTACGACAGGGACCACTACAAGCTCGCGCTTGGTCAGCTTAAC
ATCGCCCGTCATCTCATCGACAACTTGTCGAAGGACTATGTCCGCCTCCTGAAGTTCGGT
GACTCTCTGTACCGATGCAAGCAGCTCAAGAGGGCCGCCCTCGGAAGAATGTGTACCCTC
ATGAAGAAGCAGGGTCCCTCTCTGGCCTACCTCGAGCAGGTGAGGCAGCATTTGGCCCGT
TTGCCCTCCATCGACCCCAACACCAGGTCAATGATTCTCTGCGGCTATCCCAACGTGGGT
AAGTCGTCCTTCATGAACAAGCTCACTCGCGCCGACGTCGACGTGCAGCCCTACGCCTTC
ACCACCAAGTCGCTCTTCGTGGGTCACACCGACCACGAGTACATGCGCTTCCAGGTCCTG
GATACGCCCGGTATCTTGGATCATCCCCTCGAGGAGAGGAACACCATTGAGATGCTTTCG
ATCACGGCTCTGGCGCACTTGAGGGCGGCCATCATCTTCGTCATCGATATCTCGGAGCAG
TGCAGCTACTCCATCGAGGACCAGGTCAAGCTCTTCAACTCCATCAAGCCCCTCTTCACC
AACAAGCCCCTCGTCGTGGCCCTCAACAAGATCGACGTCATCACCCTCGACAACCTGCGC
GAGGACGCCAAGGCCCTTCTCCGCGATCTCGAGACCCAGAACAACGTCTACCTCATCCCC
ATGTCGACCCTCACCGAGGAGGGCGTCATGAAGGTCAAGGAATTCGCTTGCCAAAAGCTG
CTCGACCAGCGTATCGATCAGAAGCTGGCCACCGTGAAGCAGGACGACGTGCTGCGTCGC
CTCCACGTCGCCACCCCGCTCCCCCGCGACAGCCGGCAGAGGAAGCCCGTCATCCCCAAG
TCAGTCGTGCAGAAGCGCCAGCTCAAGAAGGAGGGCGCCGAGGAGATGGAGCGTGAGGTG
ACCTCCAACGCCATCGACCTCGTACACAAGAACAGGTGGCTCATGGCCGAGGACCCCGAC
TGGGACCCCACCGTGTTCGGCCCCGACCTGAGGAACCAGTTCATGCTGGCCAACGACGAG
TGGAAGTTCGATAACATCCCGGAGATCATGAACGGCATGAACGTGTTCGACTACATCGAT
CCCGACATCCTGGTCAAGCTGCGACAGCTGGAGAAGGAAGAGGAGGAGCGACTGGAGAAC
GAAGCGGGCGAGATGGAGGAGGAGCAGCCCTTCCGCCTGACCGAAGACGAGATCAACCTC
CTCAAGGAGCTGAGGAACAAGGACGCCCTCACCCGCATGGAGCACGCCCTCAGAAAGGGC
AACAAATCCCGACCCACCCTCACCCAGGCCTCCAGAACCGGCAGGCTGCAACTGGGCGCG
TTCGAGGACCATTTGAAGGACATGGGCATAAACCCGAGCGCTGCGGTGGACCGAGCCCGC
AGCATGAGCCGAGGCCGGAAGAGGTCGCGCTCCGAGAGCCCGTCGGTGGAGCGGTCCAAG
TCGCGAGGCAGGAGCAAGACCCCGCAGGAGGAGGGTCTGCGAGACAAGCGACAGAAGCTG
GAGGTCGAGGTCAAGGCCAAGAAGGCGCAGAGGACCATGAACAAGGACGGACGAAAGGGA
GAGGCCGACAGACACATCTACAACCTCAAGCCCAAGCATCTCTACGCCGGCAAGCGCGGC
AACGGCAAGACCGACAGGAGGTGA
>g1377-147786-148825
ATGGAAGCTTCTGGTGGTGCCTCTGTGTCCTCTTCATCTGGTGCCAAGGCCATGCAGGAC
CTTCTTGAGGGACACAAGGAGTGGTCGAGAGCTGTGGTTTATGACCAGGACGGCAAGGTC
CTCGCTTCCACTTTCGATGTGGACCTTAATGACATCGAAGATTTGCTTCCACTTTTCAAC
GACGAAGACAATGCCTTCAGATTTGGCCTTGATCTGGGCGGTGAGCACTACGACGTGCAC
AGATTCTACGACACGCTGGTCTACGGCAGGAAGGTGGACCAGAAGACGGGCGACGGAATC
TGTGTCTGCCGAACCAAGAGTGGGGACAAGGCGATTTTTGTGCTCATCACCTACGCCTTC
CCCACTCTCTCGGCCAAGGCCGTCCCCGATCTCCAGCAGTTCTGCAAGGCCCACGTGGAG
CCCCTGCTCTGA
>g1377-172335-174248
ATGGATCGGGAGTATCTGTACAACTCCGTGCTGTATGTGCTGTACGAGTCCGCTTCAGGC
TACGCCCTCTTCGAGCGCATCGAATCGGAGGAAATCGCCGATGAGTCGCCGGAGCTGCAG
AGGTCGATCCAGGACTTCGGCCGTTTCTCGAGGATTGTCAAGTTCAAGTCTTTCGTGCCC
TTCGTCTCGGCCGAGAGCGCTCTCGAGAACGTCAACGCCATCTCTGAAGGTCTGGTGCAC
GACGTGTTGAAGAACTTCCTGGAGCTCAACCTGCCGGGCGGGGGCAAGAAGAAGAGCAAG
AAGGTGGAGCTGGGCGTGACGGACGAGAAGCTGGGCGGCGCCATCAACGACTCGCTGGAG
CACGTGGCATGCGTGAAGAGCAAGGCGGTGGCGGAGCTGACGCGCGGGATCCGGCTGCAC
TTTGCCAAGTTCATCAAGGAGTTCAAGGAGGGCGACTACGAGAAGGCGCAGCTGGGTCTG
GGCCACTCGTACTCGCGGAGCAAGGTCAAGTTCAACGTGAACCGGGCGGACAACATGATC
ATCCAGACCATCAACCTGCTCGACACGCTCAACAAGGACCTCAACACGTTCTCCATGCGG
TGCAAGGAGTGGTACTCGTGGCACTTCCCGGAGCTGGTCAAGGTGGTGCCGGACAACTTC
CAGTTCGCGCGCGTGGTCAAGTTCCTGAAGAACAAGGCCGAGGCCGACGAGAGCAAGATC
CCCGGCCTGGTGGACATCACGCAGGACGAGGCCAAGGCCAAGGAGATCATCGACGCCGCC
AAGGCCTCGATGGGCACCGACATCTCCGACCTCGACATGCTCAACATCGAGAAGTTCGCC
GACCGCGTGATCCACCTCTCCACCTACCAGCAGCAGCTCCAGGAGTACCTGAGCAAGAAG
ATGCACGTCATCGCGCCCAACCTCAGCGAGCTCGTCGGCGAGCACGTCGGCGCGCGCCTC
ATCTCGCACGCCGGCAGCCTCACCAACCTGGCCAAGTACCCGGCCTCGACCGTGCAGATC
CTGGGCGCCGAGAAGGCCCTCTTCCGCGCGCTCAAGACCCGCGGCAACACGCCCAAGTAC
GGCCTCATCTTCCACTCGTCCTTCATCGGCCGCGCCGCCGCCAAGAACAAGGGCCGCATC
TCGCGCTACCTGGCCAACAAGTGCTCCATCGCCTCGCGCATCGACGCCTTCTCCGACGTG
CCCACCACCAAGTTCGGCCAGAAGCTCAACGCCCAGGTCGAGGAGCGCCTCAAGTTCTAC
GACACCGGCGCCCTGCCCCGCAAGAACGTCGACGTCATGAAGGAGGCCCTCTCCGAGGCC
CTCACCGAGGCCGACCAGAAGCAGCGAGAGGACGGCATGGACGTGGTCTCCACCACCGCG
GAGAGCACCGACAAGAAGAGGAAGAGGAAGGCCGCGGAGAAGGACGACAGCGACAGCGAC
GAGGAGGAGAGCAAGAAGGACAAGAAGAAGAGCAAGAAGGCCAAGAAGTCCAAAAAGGAG
GAGGAAAAGAAGAAGAGCAAGAAGGACAAGAAGAAGAAGAAGTCCAAGCACTAA
>g1386-34326-35310
ATGGGTATTGATCTTGAGCACAAGCACAGGAAGAACAAGAACAGGTCTTCCCCCAAGACC
GAGGATCCCTACCTGCTTCTGCTCGTCAAGCTCTACAGGTTCCTCGCCCGCAGGACTGGC
GCTGACTTCAACCGCGTCGTGTTGAAGAGGCTCTGCATGAGCAGGACCAACCAGCCCCCC
CTGTCCCTCATCAAGCTCGCCCGCTTCACCAAGGGCAAGGAGGACAAGATCGCCGTTGTC
GTCGGCACCGTCACCGATGATGCCCGTCTCCTCAAGTTCCCCAAGCTGACCGTGTGCGCT
CTGAGGTTCACCGAGGGCGCCCGCACCCGCATCCTCAAGGCCGGCGGTGAAATCCTCACC
TTCGATCAGCTCGCCCTCCGTGCCCCCACCGGAGCCAACACCGTCCTCCTTAGGGGTCGC
AAGACCGCCAGGAAGGCCACCAAGCACTTCGGCGCTCCCGGTGTCCCCAACAGCACCACC
AGGCCCAAGGTCGCCAACAGGAGCAGGAAGACCGAGAGGGCTAGGGGTAGGAGGAAGTCT
GTCGGCTTCAAGGTGTAA
>g1386-44106-46660
ATGAGCGACGGTCAGGAGCCGCAGGGAGAGGAGGGGGGAGAGCAGCAGGTGGGATGGACC
AGTGAGATCCACGAAGCCGTGTGGCAGAACAACACCGAGAAGGTGCGACAGCTGATCAAC
AGCCCACAAGGCGCGCCGCTGGACCTGGAGGCCGTGGTGGAAGATTGCACGCCGCTCTAC
TTGGCCTGCCTCAAGGGCCACACCGATGTGGCCGAGATTCTCATCAAGGGAGGCGCCAAG
GTCGACGGCGATTCGCCGCCGGTCTTCGCGGCTGCGGGCAAGGGCAATCTCGATCTCGTG
CGCCTGCTGTTGAAAAATGGCGCCAATCCGTCGGCTTTCTCGTCGCAGCATGCCGGCTGC
TGCGGCCTCCACATCGCCGCCGAGAAGAACTACGTCGATGTCGTTCGTGCGCTGCTCGAG
GCCGGCGTCGAGGTCAACATTCGATCCGAGAAGCAGAAGCTGACGCCCCTCATCTCGGCC
GCCTGCTGCGGTTCGCTCGACGCGGTCAAGGTTCTCATCGAGGCCGGCGCCGATGTCGAT
GCCCAATCGTCGACGGGCAACACGGCCCTCATGCTTGCCATCGATCGCGGCAAAATTGAC
GTCGCCACCACGCTCATCGATTCGGGCGCCAACCTCGAGATCAAGGGCCAGAAGGGCTGG
ACCGCTCTCCACAACGCCGCCAGCGGGGGCGACAAGGGCTACCGCGAGGTGGCCGAGGCG
CTCCTCAAGGCCAATGCCTCGGTCGATGCGCTGTCCGAGACCATGCTCACGCCCCTTCAC
GAGGCTGCCGGCAAGTCGCTCACCGATCTCGTGCGCCTGCTCGTGGACCACGGTGCCAAC
GTCAACGCGCGCGACAAGTTCAACAACACGCCCCTCCGGATGTGCGCCTCCAACGCGCAG
TCGTTCGCCTCCCTCGACAGCTTCAAGCAGACCGTCCAGACCCTGCTGGACGCCGGCGCC
GACATCAATGCCGGTACCACGATTAACACCACCTCGCTCCACTCGGTGGTCAAGTGGGGC
AACCCCGACGCCGTGCGCTTCATGCTCGACCACGGCGCCGACCCCAACGTGAGGACCACC
AAGGGCGAGCTCCCCATTGATTTTGCCAAGGAGCCCGCAATCCGCGCCCTCCTCGAGCCC
GTCACCAAGGCCGCCGCCAAGACCGACAAGCCCACCAACGGCCCCGCCGACGCCAAGGGG
GCCAAGGCCGACAAGAACGTTTCCCGGAGCAAATACAGCCACGACAACTGGCAGCCCGAC
AACACGACTCCCGAGTGCATGCACTGCTCGAAGAAGTTCACTCTCATCGTCAGAAAGCAC
CACTGCCGTAACTGCGGGCAAATATTTTGTGACCAGTGCACGCCACACCGCATGCGTCTG
CCCGACAAGGGCATGAAGGAGCCAGTGCGCGTATGCGTGGTGTGCGCCGACACGAACACA
AACACCAAGGCCAAGTCGAACTAA
>g1390-149736-151000
ATGCCGAAGAAGAGGGAGAAGGAGAAGAATCATTGGCAGGTGCACTTGTGCGAGACCTGC
TACCAGGAGCCTTGCGAGTTCTGCAAGGCCTGCGTGTGCCCGTGCGTCTACGCCGGCTGC
CAGCGCTACGAACTCCTTGACCACAACCTCGACAACTACACCTGCTGTGCCGGCATTTGT
GGCGACTGCTTCGATCCGTGCACCTCGCCTTGCCCCGGCTGCTGCCTGGTCGTGGAGGTC
TCCGTCTGCTGCTGGTTGTCCATTGCCGGAAACCGTTCAATGATCCAGAACACCTATGGC
ATCAGGAACACCAAGTGCGAGGACTGCATGATCTGCTGCGCGTGCGTCTTCTCGTTCGTG
TGGTGCATCATCAACATCTTCTTCAACGTGCCCGATGGGTCGAACATGGTGGTGGACTGC
TTCTACCTGGCCTTATCGGCGTGCTTCCAGTCTCAACAGCACGCCGAGATGGAATACCAG
AGCAAGAAGCACGCACCCCACGAAGTCGAGATGAAGAAGATGTAA
>g1390-176500-178208
ATGGAAGGCGCGATTGATGCTCAGGCCGGTGGCGTAGAGGCCCCCGTGGCTCCCCTCTCC
GATGTGGCGAAGAAGGTCGGCAAGTGCCGGATGTACGAGGCCGAGTACCCCGAGCTGGAC
GATCTCGTGATGGTGCGCGTCAATCGCATCAACGAAATCGGCGCCTTCGTATCGCTGCTC
GAATACAACGACAAGGAGGGCATCATTCTGCTCTCCGAGCTGTCGAGGAGGCGCATGCGC
TCGATCAACAAGCACATCCGAGTGGGCAAGAAGGAGGTGCTGCAAGTGCTGAGGGTGGAC
AAGGAGAAGGGCTACATCGATCTCTCGAAGAAGCTGCTCAAGGAGGAGGACATCGTCGAG
TGCACTCAGCGGTACCAGAAGTCGAAGACCGTACACAGCATCATGGCCCACGTCGCCGAG
CTGCAGACGGGCGACAACATCCTCACTCTCGAGGACCTGTACAAGCGCATCGGGTGGCCT
CTGTACGAGACGTACAACCATGCCTACGACGCATTCAAGAAGGCCGCCGCCGGCGAAGAC
ATCTTCGAGGGCCTCGACGCTCCTGCCGAGATCAAGGACAACATCCTCAAGACGATTCAG
CATCGCCTGGGCAGCCAGCCCGTCAAGATCCAGGCCGACATCCAGGTGACGTGCTTCTCC
TACGAGGGCATCGACGCCATCAAGCCTGCCCTCCGCGCCGGCCAGCAGTGCAGCACCCCC
GATTCGCCCGTCCGCATCCAGCTCGTCACGTCTCCCGAGTACATCCTCCTCACTTCGTCC
ACCGATCACGAGCGCGGCGTTGCCCTCCTCAAGAAGGCCGTCGAGGCCATCCAAACCGAA
ATCAAGAAGCACGGAGGTGACTGCGTGATCAAGACCGAGCCGCGAGTCGTGGCTTAA
>g1390-178306-179670
ATGGACTCCCGCTACTCCTTCTCGCTCACCACCTTCAGCCCTTCTGGCAAGCTAGTGCAG
ATCGAATACGCGCTCAACGCCGTGAACGCTGGTGCCACCGCTCTTGGTATCAAAGCCAAG
GATGGCGTGGTGATGGCCACCGAGAAGAAGCTGCCTCCTCTCATGGATGACACTTCGATC
CAGAAGATTAGCCTCCTCACGGACAACATCGGCGTCGTCTACGCTGGAATGGGACCTGAT
TCGCGTGTGCTCGTGCGCAAGGGACGCAAGATTGCCCAGCAGTACTTCAGGACCTATCAC
GCTCCCATCCCTGTGAATCAGCTCGTGCGAGAGCTCGCTGCTATCATGCAGGAGTTCACT
CAATCTGGTGGTGTGCGACCCTTCGGTGTCTCCCTCCTTATCGCCGGCTATGACGAAGTT
GCTGGTGCTCAGCTGTACCAGGTGGATCCTTCGGGTTCGTATTGGGCGTGGAAGGCCTCG
GCCATCGGCAAGGGCATGGTCAACGCCAGGACCTTCTTGGAGAAGCGCTACAAGGAAGAT
GTGGGTCTCGAAGATGCCGTCCACACGGCCATTCTGACGCTCAAGGAGGGATTCGAGGGT
GAGATGACGGAGAACAACATCGAGCTGAGCGTGGTGCAGGTGATCGAAGGCAAGCCCGTC
TTCAGAGTGCTCACGCCCGCCGAAGTCAAGGACTACCTCGGCGAAGTACAGTAA
>g1390-216047-218303
ATGTCCAAGGAAGTCTGCGTCGGTATCGATCTGGGCACGACCTACTCGTGCGTGGGCGTG
TGGCAGAACGATCGCGTCGAGATCATCGCCAACGATCAGGGTAACCGCGTGACCCCGTCG
TACGTGGCCTTCACCGACAGCGAGCGTCTCATCGGAGATGCCGCCAAGAACCAGGTCGCC
ATGAACCCGCACAACACCGTGTTCGATGCCAAGCGTCTGATCGGCCGTAACTTCTCGGAC
CCCATCGTGCAAGCCGACATGAAGCACTGGCCGTTCAAGGTCGTCCAGAAGCCCGGCGAC
AAGCCCTTCATCTCCGTCGAGTACAAGGGCGAGACCAAGGAGTTCTCCCCCGAGGAGGTC
TCGTCGATGGTCCTCATCAAGATGAAGGAGACCGCTGAGGCCTACCTCGGCCACCCCATC
AACTCGGCCGTCATCACCGTGCCCGCCTACTTCAACGACTCGCAGCGTCAGGCCACCAAG
GATGCCGGTACCATCTCCGGCATGCAGGTCAAGCGTATCATCAACGAGCCCACCGCCGCT
GCCATCGCGTACGGTCTCGACAAGAAGTCGCAGGGCGAGATGAACGTCCTCATCTTCGAT
CTCGGCGGCGGTACCTTCGACGTGTCGCTCCTGACCATCGAGGAGGGCATCTTCGAGGTC
AAGGCCACCGCTGGTGACACTCACCTCGGCGGTGAGGACTTCGACAACCGCATGGTCACC
CACTTCATCCAGGAGTTCAAGCGCAAGCACAGCAAGGACATCTCCGGCAACGCCCGCGCC
GTCCGTCGTCTCCGTACCGCCTGCGAGAGGGCCAAGCGCGCTCTGTCGTCGGCCACCCAG
ACCAACATTGAGATCGACTCGCTCTTCGAGGGTGTCGACTTCTACACCTCCATCACCCGT
GCCCGTTTCGAGGAGCTCTGCGCTGATCTCTTCCGTTCGACCATGGACCCCGTCGAGAAG
GTGCTCCGCGACGCCAAGATGGACAAGCGCACCGTGAGCGAGGTCGTGCTCGTCGGTGGC
TCCACCCGTATCCCCAAGATCCAGCAGCTCGTCACCCAGTTCTTCAACGGCAAGGAGCCC
TGCAAGTCGATCAACCCCGACGAGGCCGTCGCCTACGGTGCCGCCGTCCAGGCCGCCATC
CTCAGCGGTGCCTCGACCGAGGGCTCCGTCCTCGGTGACGTGCTCCTCATCGACGTCACC
CCGCTCACCCTCGGCATTGAGACCGCCGGTGGCGTCATGACCGCGCTCATCCCCCGCAAC
ACCACCATCCCCACCAAGAAGACCCAGGTCTTCTCCACCTACTCGGACAACCAGCCCGGC
GTGCTCATCCAGGTCTACGAGGGCGAGCGTGCCATGACCCGCGACAACAACCTCCTCGGC
AAGTTCGAGCTCTCGGGCATCCCCCCCGCGCCCCGCGGTGTGCCCCAGATCGAGGTCACC
TTCGACATTGACGCCAACGGTATCCTCAACGTCAACGCCGCCGACAAGACCACCGGTCGC
TCCGAGAAGATCACCATCACCAACGACAAGGGCCGTCTCTCCAAGGAGGAGATCGAGCGC
ATGGTCAAGGAGGCCGAGAAGTACAAGAACGACGACGAGAACGCCAAGGAGAAGGTCGAG
GCCAAGAACGCCCTCGAGAACTACGCCTACACCATGCGCAACACCATCCGCGACGACAAG
ATCGCCTCCAAGCTCGACTCGGGCGACAAGTCCAAGATCGAGGAGGCCGTCGACGCCGCC
ATCAAGTGGCTCGACACCAACCAGACCGCCGAGAAGGACGAGTTCGAGCACAAGCGCAAG
GAGCTCGAGGACCTCTGCAACCCCATCTTCACCAAGATGTACCAGGGTGCCGGCGGCGCT
GGCGGCATGCCCGACATGGGCGGCATGGGCGGCGGCTTCCCCGGCGGTGCTGGCGGTTTC
CCCGGCGGCGCGGCTCCCGGCGGTGGTGCCCGTCCCTCTGGCGGTTCGTCGGCCGGCCCC
AAGATCGAGGAGGTCGACTAA
>g1390-397593-398409
ATGGTTGCAGTTGACGGCAGCGAGCACTCGAACAAGGCCTACGCCATCGCGGCCAAGTTG
CTCACCAACAAGGACGAGGTGATCTTCGTGACCGTTGGGCAGAAGGGCAAGGGCGCTGCT
GCGCAGGATCTGCTCGAGACATGGACGAAGAAGGCCGAGGCTGATGGCTTCACTGCCAAG
CCGCTCTTCCTCGAATCGGCCGATCCTCGTGACGCCATTTGCAACGCCGTCACGGAGCAC
GGCATCGACATTCTCGTTGTGGGCACCAGGGGTCTGGGTACCATTAAGAGGATGCTGCTG
GGCTCGGTGAGCAACTACTGCGTGCAGCACGCCTCGTGCGACGTGATCGTGGCCAAGTAA
>g1390-448270-449109
ATGACCAAGTACATGGTTGCAGTTGACGGCAGCGAGCACTCGAACAAGGCCTACGCCATC
GCGGCCAAGTTGCTCACCAACAAGGACGAGGTGATCTTCGTGACCGTTGGGCAGAAGGGC
AAGGGCGCTGCTGCGCAGGATCTGCTCGAGACATGGACGAAGAAGGCCGAGGCTGATGGC
TTCACTGCCAAGCCGCTCTTCCTCGAATCGGCCGATCCTCGTGACGCCATTTGCAACGCC
GTCACGGAGCACGGCATCGACATTCTCGTTGTGGGCACCAGGGGTCTGGGTACCATTAAG
AGGATGCTGCTGGGCTCGGTGAGCAACTACTGCGTGCAGCACGCCTCGTGCGACGTGATC
GTGGCCAAGTAA
>g1390-687387-688358
ATGGCCCATCGCGAATATCAGGTTGTCGGTCGCCGCATCCCCACTGATGCTGACCCCACC
CCCAAGGTGTACCGCATGCGCATCTTCGCCCAGAACGACGTTGTGGCCAAGTCGCGCTTC
TGGTACTTCCTGAGCAAGCTTGCCAAGGTCAAGAAGGCGAACGGCGAGCTCCTCTCCATC
AACGAGCTGACCGAGAAGAAGCCCAGCACCGTCAAGAACTTCGGTATCTTCCTCCGTTAC
CGATCGAGGACTGGCATCCACAACCTGCACAAGGAGTTCAGGGACACCACTCGCGTCGGT
GCCGTCAACCAGATGTACAACGAGATGGCCTCCGTCCACAAGGCCACCTGGAACAACATC
AACATCATTGAGGTCAACCAGCTTAAGGCCAAGCAGACTCTCCGCCCCAACATCAAGCAG
TTCCACGACTCGAAGATCAAGTTCCCTCTGCCCCACCGTGTCCAGCGCGTGCCGATGAAG
AGGTACAGGAGCACCTTCGTCGCCAGGAGGCCGTCTACCTTCTGGTAA
>g1390-714707-716810
ATGAGGGAAATCATCTCTATCCACATTGGTCAGGCTGGCGTTCAAGTCGGTAACTCGTGC
TGGGAGCTTTACTGCCTGGAGCACGGACTCAACCCTGACGGTACTCTTCAAGAGGGCAAG
ACCTCGACGCCGCTCGCCTTCAGCACCTTCTTCAGCGAGTCGGGCAGCGGCAAGTACGTG
CCGAGGGCTGTGTACTTCGATCTTGAGCCTTCGGTGGTCGATGCCGTCAAGCAGGGTCCC
CAGGCTAAGCTCTTCCACCCCGAGAGCCTCATGAGCGGCAAGGAAGATGCGGCCAACAAC
TACGCTCGTGGTCACTACACCACCGGCAAGGAATACGTCGAGACCGTCGTCGACAAGATC
CGCAAGCTCACCGAAGATTGCGAGGGCCTCCAGGGTTTCTTGGTCTTCCACTCGGTCGGC
GGTGGTACCGGATCCGGCTTCGGTTCTCTGCTTCTTGAGCGTCTCTCCGTCGATTACGGC
AAGAAGAGCAAGCTCGATTTCTGCGTGTTCCCCTCGCCCCAAATCTCGACCAGCGTCGTT
GAGCCCTACAACTCCGTCCTCAGCACCCACGCCCTGCTCGAGCACACCGATGTCGCCTTC
ATGCTCGACAATGAGGCCATCTACGACATCTGCCGCGACAAGCTGCGTCTGGAGCGCCCC
ACCTACCAGAACCTCAACAGGCTCATCGCTCAGGTCATCTCCTCGCTGACCGCCTCGCTC
CGATTCGACGGCGCCCTCAACGTCGACATCAACGAGTTCCAGGTCAACTTGGTGCCCTAT
CCTCGCATCCACTTCCTGCTCTGCTCCTACGCTCCGATCGTTAGCCCCGAGCGCGTGAAT
CACGAGAAGCTCTCCGTGCCCGAGCTCACCTCAGCCGTGTTCGAGCAAAGCACCATGCTC
GCCAAGTGCGACCCGAGGCACGGCAAGTACATGGCCGTGTGCATGATGTACCGCGGTGAC
GTCGTCCCCAAGGACGTCTCGGCCTCGATCGCCAAGGTCAAGACTAACAAGACCATTCAG
TTCGTCGATTGGTGCCCCACTGGCTTCAAGTGCGGCATCAACAACGAGGCCCCTACCGCT
GTTCCCGGCGGCGACATGGCCAAGGTCAACAGGGCTTGCTGCATGTTGTCCAACACCACC
GCCATCGCCGAGGTCTTCTCCCGCATCGACCACAAGTTCGACCTCATGTACGCCAAGAGG
GCCTTCGTCCACTGGTATGTCGGCGAGGGTATGGAGGAGGGTGAGTTCTCTGAGGCCAGG
GAGGACCTCGCGGCCCTCGAGAAGGACTATGAGGAAGTCGGCCTCGATTCCGTTGACGAT
TCCTCGGTTGACGCCGACGGTTCGCTCTCCGTCGATGGCTCTGAGATCTAA
>g1393-41406-43944
ATGATGGATACGCAAACTCCCGTACATCCCCCTCGGCCGTTCGAAATCGTGGTGGCTGCG
TCTGTCAATGGCGGAATCGGGCTGGCAGGACAGTTGCCCTGGCAATTGCCTCAGGAGATG
GCCAGGTTCAAGGCCTTGACTCTCAAAACAGCAAACGATGACCACTCGAACGCGGTCATT
ATGGGCCGGCGGACCTACGAGTCGATCCCCGCGAAATTCAGACCTCTCAAGGGCAGGGTC
AACATCGTACTCTCGCGCGACCAGCATCGTAATTGTGTCAGTTTGCCCGACAGCGTGGTG
GTGGCGAGTAGTTTTGACGAAGCACTCCTGGCCATCCAGAGCATGGAGAAGGTCGAAAGG
GTGTTCGTTATTGGTGGGGCTCAAGTCTACGCGGAAGCCATAAAGCATAATGGTTGCCAG
GCGATTTACCTTACACAAATCACGTCTCCCGAGTTCGAGGTAGACACCTTCTTTCCGCAG
ATTGATCCGCACTCTTTCAAGCTCGACGCCAGCTATCCTGAGCAAGGGAAGGCGCATGAA
GAAAAGGGCGTCACGTATGAGTTTTTACGGTATGTACGCAGCAAGTCCGTTGGGCCGCAT
GAAGAAGAGCAATACCTTCGGCTTGTGCACGCGATCATCAAACATGGCAACGACAGAGAC
GACAGGACGGGAGTTGGCACTCTCTCTCGGTTCGGGTGTCAGATGCGGTTTAGCTTGCGG
GACGGCACCTTTCCTCTCCTCACCACTAAGCGCGTGTGGTGGAAGGGGGTGGCCGAGGAG
CTCCTGTGGTTCGTGTCGGGCTGCACGAATGCCAACACTTTGGCAACCAAAAAGGTCAAC
ATCTGGAAGGAGAACGGATCCAAGGACTTCCTCGCCAAGTGCGGACTTGGCCACAGAGAA
GAGGGAGACTTAGGCCCCGTTTATGGCTTCCAGTGGAGGCACTGGGGAGCAAAATACGTG
GACATGCATACCGACTACTCGGGCCAAGGGCATGACCAGTTGGCCGAATGCATCGCACTC
ATCAAACATAATCCCACATCGCGTCGTATCGTTATGTCAGCCTGGAATCCGACAGACCTC
AAGGAAATGGTGTTGCCTCCGTGCCACATGTTTTGCCAGTTCTACGTTGCCAACGGCGAG
CTCTCTTGTCAGATGTATCAGCGCTCTGCTGACATGGGACTAGGCGTGCCATTCAACATT
GCTTCGTACTCCCTGCTGACCTGCTTGGTCGCTCATGTATGTGGCCTGATCCCAGGTGAG
TTTGTACACACAATTGGTGATGCACATGTGTACAAAAGCCACATTGAAGGGTTGAAAGAA
CAACTCACGCGTCCTCCATATCCCTTCCCAAAGCTCCACATCAAAGACAGAGGCCAGAAC
ATTGATGGCTTTGTCTACGAGGATCTGGTCATTGAGGGATACCAACACCACCCCACCATC
ACCCTCCCTTTTGCTGTGTGA
>g1406-111173-112358
ATGCAGCGTGCTCTTCTCTTCTTCGCCATCCTGGCCCTCACCGTGGCCTGCGCCCTCGCC
CAGACCAGGCCCAAGCTCGCCACCACCTTCGAGACCCACGGCATTGTCCAGATCAAGCAC
AACCACTCCGTCGTCTTCGGCGAGGGTAGGTGGATCTTTGATTTGGAGGCCGGCAAGTCG
CTCGACTGGGCTCACTTCGGTGGCTTCGAGCACCTCGGCGTCTACGACCTCGCCAGGTTC
GACCTCAAGAAGAACTATTTCATCTCCTCCGCTGACGCCAGGAAGTGCGAGGAGAAGGAC
CTGACCGACAAGATCCGTTCCCCCTGGGAGTGGCTCGACAAGGCTGAGGTTGTGGGCAAC
CTCACCAGGAACGGCGTCCTCTTCGACCTGTGGCAGTACAAGACCGCTGGCGTCACCCTC
GAGGCTGGCGTGCCCCAGAAGAACCCCACCGAGCTGGTCTACTTCAGCCGCACTTCCGTG
CGCGAGGAGTTCCACTTCTACGTCGAGCAGTGGAGGACCAACAAGCCCCACCAGTCGTGG
TTCGAGGTCCCCCACGAGTGCAAGAAGGCCTTCTAA
>g1406-120156-121446
ATGGAGGGCAAGGACGCCGAAGTAGAGCAGATTGCAGAGGAGACGGAAGCCGAGATTCCC
GAGGAGATCCCTGCCATGAAGGCCAAAGAAGTCCTTTACTGTGGTGTCTGTGGCCTGCCT
GCCGAGTACTGCGAATTCGGACCTGACTTTAATCGATGCAAGCCGTGGCTGATCGAGCAC
TGCCCCGAGATCTACCCTTCGCTGATCAAGAAGGAAGGCGAGGAAGGCGAGGAGAAGGAA
GGAGAAGGGGAAGAGGAAGAGAAGAAGAAGTCCAAGCGTGGCGGCAAGGGAGTGGTGAAG
GCCGACAAGGGACCCCAGAAGGGAGGCAAGGACGCCGAGTTGACCATCACCACGAGCCAG
AAGAAGGGCAAGAAGATCGTCACCACGGTCTCTGGCCTCAAGGGATACGGCGTCAACCTC
AAGGACGCAGCGAGTGTGTTCAAGAAGAAGTTTGCGTCGGGCGCGTCAGTCACGAAGGAG
GGTGACGTGGACATTCAGGGTGATCTGAGCCGCGAAGTCAAACAGCTTATCGTGGAGAAT
GCCGAGTGGAACATCCCGCGAAAGTCGATCAAGATCGTGGAGTCCACCAAGAAGCGACAC
TGA
>g1406-163052-164156
ATGAACCGTCTGTGTGGAAGTCTCGCTAAGGGCGCTCAGAAGTTCCAGGCTTTGGAGCAG
AGCACTACCAGCTGCGCTTGCAACAAGCAGCTCTACGGCTACCTCGGCACCCAGTTCGGC
TGCTGGTCTAACCGCTGGCAGGCATTCCCTGCCGACGCCAAGGCTCTTTGGCGGCGCGTG
ATTACCGGCCAGATCACCGTGGGTGAGGTCGGTCGTGGCGTCCGCGCCAGCACCCCCATT
CTGCTCGCCGCTCTTGCGGGTAACTTCCTCGGCAGGGGTGAGTTCCTTTACTCTCCTATC
GTCGGTGCCTCCTACACCTACCCCGAGTACTAA
>g1406-164242-165361
ATGAGCAAACTGCAAACCGAAACCCTCAGCAAAGCCATCGCTGGAGTACTGGCTGGTCGC
GTCGACAAGGCCGGCAACAAGAAGAAGCGCAGGTTCCTCGAGACCGTCGAGCTTCAGATT
GGTTTGAAGTCGTATGATCCCCAAAAGGACAAGCGTTTCGCTGGTGCCGTCAAGCTGCCC
TACGTCGCCCGCCCCAAGATGAAGATCTGCGTCCTCGGTGACGAGCTTCACTGCGATGAG
GCCAAGCAGGCTGGTCTTGACTTCCAGACCACTGAGGATCTCGGCAAGTTCAAGAAGGAC
AAGAAGATGGTCAAGAAGCTCGCTGGCCAGTACGACGTCTTCCTTGCCTCGCCCGCCGTC
ATCAAGCTGATCCCCCGTCTCCTCGGCCCGGGTCTCAACAAGGCTGGCAAGTTCCCCACC
CTCATCGACCACAAGACCCCGATCACCGACAAGGTTGAGCAGATGAAGTCGACCATCAAG
TTCCAGCTTAAGAAGGTCATCTGCATGAACGTCGCCGTCGGTCACGTCAAGATGAAGCCC
GACGAGCTCGACCTCAACATCCGTCTCGCCGTGAACTTCCTCGTGTCCCTCCTGAAGAAG
AACTGGCAGAACATCAAGTGCCTCTACGTCAAGTCCTCCATGGGCCGTCCCTTCCGCATC
TACTAA
>g1406-181868-185544
ATGAGTAAGTTCTGGGGTGGTGCCGACAGCTCTGACAGTGAAGAAGAGGTTTCCGACAAG
GAGCTGCTCTCGGGAAGCGAAGGCTCGGATGGAAGCGGCGATGAGGGTGAGGGAGAGGGA
GAGAAGGACATCGACTGGGGCATCGATTCGGACTCGGAAGGCGAGGATGCCAAGCGAGTA
GTGAAGAGCGTCCGAGATAAGCGTTATGAAGAGATGCACAAGCTCATTGAGAGCCTCGAC
AAGGACATCAAGGAGGCCAATTGGGTCAACATCAACACTGATTTCCAGAGCCTCAGTGTG
TGGATCAAAAAGGCCAGCAACCTCATTAACAAGGAAGGTGTGCCCGGCCCCTATGTTTCC
ATCCTGATCCAGCTTGAGGACTGTCTCAAGCAGACTCTCGCCGACAAGGAGGCCAAGAAG
GCCATGAGCAAGAACGATGCCAAGGCCTTCAACACCTTCGCTCAGAAGCTTAAGAAGAAC
AACAAGGATTACCAGGCCGAAATTGATCGCTTTAAGCAGAGCCCTGCCACCTTCAACAAG
GCCGCCGTCAAGGCCGCTAAGAAGGACGAAGATGAGGACGAGGACGACGAGGAGGACAAC
GAAGATGACCTCATGGCCCTCGCGAAGCCTGGCAAGAAGGCTGCTGCCATTTCCGACGAG
GATGACGAGGAGGATGACAAGAAGGCTGCCCCCAAGCCCGGACAACGCAAGGCTCCCGCC
CCCGTTCAGGCCAAGATCGTGTGGACCGACGAGCTGATCGCCGAGAAGCTCGACGAAGTC
GTCAAGAAGCGCGGTATGAAGAAGGCCACCCCCAAGGAGCTGATCGACCAGCTCAAGGTG
CTCCTGGCCAACGTCAAGTCGGCTCAGAAGAAGGTGGAGATCTACATGCAAATTATCGCC
ATCATGTTCGACCGTACCAGCGCCAACGTCACGAAGGCCATGAAGCCTGGTGCCTGGGCC
AGAGCCCGCGAATACATCGCCGAGCTGCTGCAGCTTCTGAGCTCCGACAGCTCTCTGATC
CTCACCGAAACCGCCAGCGAGGCTGCTGTCGAGTCCGAGGACGGCAGCTTGCCCGCTGTT
GCCCACATCGCCGTCAACCTGACGTCATTCATCGAGCAACTCGACACCGAGTTCAGCAAG
GGCCTGCACCACGTTGATCCCCACACGCACGAGTACGTCGACCGGCTCCGCCAGGACGGC
CTGTTCCTCGAGCTGGCTGCTCAGGTGCAGGAGTACTATGAGAAGCACGGCGACTTGGCC
CAGGCTGCTCGTATTGCCATGAAGAGGATCGAGCACTCGTACTACAAGCACGACACCATC
ACCGAGAAGCTTGAGGAGGCCCACCTCGAGCGTCAGCAGAAGGAGGCCCAGAAGAAGAAG
GAGGGCCTCGCCGAAGTCGTCGCTGATGACAAGAAGTCCTCCACCGACGTCGAGAAGCTC
TGCGTCCTCGTCTACACCCATGGCGACGAGAGGCTCAAGACCAACGCCCTTCTGTGCCAC
GTCTACCACCACGCCCTGCACGACCGGTTCCACCAGGCCCGAGACCTCATGCTGATGAGC
CACATCCAGGACAACATCCAGCACATGGATGCCCGCATGCAGGTCCTCTTCAACAGAGCG
ATGGTCCAGCTCGGTCTCTGCGCCTTCCGCCGGGGTGCCATCTACGAGGCCCACTCGTGC
CTTTCGGACATCTGCTCCGGTAAGACGAGGGAGTACCTCGCGCAGGGCATGTCCAGCGCC
CGCTTCCAGGAGAAGACCCCCGAGCAGGAGAAGCTCGAGAAGCTCCGCCAGGTGCCCTTC
CACATGCACATCAACCTCGACCTGCTCGACACCGTCCACCTCACCACCGCCATGCTCCTC
GAAATCCCCAACATGGCCGCCCACCCTCACGACATCAGACCTAAGATCATCTCCCGCGCC
CTGCGTCGTTCGCTCGACGGCTATGAGAAGAACTACTTCACCGGCCCGCCGGAGAGCACC
CGCGATTACTGCGTCGTTGCCGCCAGAGCCCTGGCCAAGGGTGACTGGAAGCGGACGGAG
GAGCTGCTTCTCGGTCTGCCGGCGTGGTCTCTGCTCCAGAACTCGGAGGGAGTGAAGACG
ATGCTCAAGCGCAAGATTCAGGAGGAGGGCCTGCGGACGTACATCTTCACCTACTCTCCC
CACTACACCGCCATGTCTCTCGAGGAGCTGTCCAACATTTTTGAACTGCCGCAGCACAAC
GTGCACAGCCTGATCAGCAAGATGATCATCAACAAGGAGATCCAGGCCTCGTGGGACCAG
CCCACCGCCACCCTTGTCATGCACAAGGCCCAGTCCTCCCGCCTGCAGCTCCTCGCCCTT
CAGCTCGCCGATAAGGCTGGCGTGCTGGTGGAGAACAACGAGCGTCTGCTCGAATCGGCC
AAGCCCGGCGGCTACGGCTACAAGTTTGACAACAAGGGCAAGAGCGGCTACGACTCGAGA
CGGGGTGGCGGCGCCAGGCGAGGCGGCTACGGCGGCTACTCGGGCGACCGCCAGGGACGC
GGTGGTGGCCGTGGTGGCTACGGCGGTGACCGCCGCCAGGGTGGTGACCGCCGCCAGGGT
GACCGTCAGGACCACCGTTCGGACGAACGTCGCAACGTCGCCGACGGCTTTGTCAGGCAG
GAGAGGCGCGGCAGGCGAGGCGGTCGCCAGCAGGAGGGCGGCTGGACCAACGCCTAA
>g1406-201874-205879
ATGCGCCTAATGCTGACCGGGGGTGTATTTATGTACCCTCCCATCCCCTACCCAACTTCT
CTTGTTTTTTATTCACCTCCGCACACAACACACAACACAGCCCTCGGCCCCCACCGGTCG
TTGTACTCCGTAGTACCTACGTCGTCGGTTTCTTATCACGAACAGCCGTCGGATAGGAAC
GAGAACACCAAAGACTCCTACACACCGACCACCATGCGGAGCCTTCCCGTCTTCGTGGTC
TTGATGGTGGCCTTGTTCGCCGCCGCCGCCTCCGCCGGCACCTGCAACTTGTCTGGTACC
ATTGCGCAACCCGGCCTTGCGTGCTCTTCGACTTCCTGCTCCATCACGAGCGGCACCAAG
CCCTTCCCGTTGCCCCAGGGCGAGACCTACGACAGCTTCTACAGCTGGATCCTGGGCGTC
ATCGGCACGGACGGAGCTCAAGTGAACACGCAATATGTCAACCCGTCCAACGCCGACCCC
AACATCTACTTCACGGCCGGTCAGACCAACTGCATGGTCAACCTCACCTTCGTCTACGAG
GTGGCCTACTACCGTAACAGCATGGGCTACTTCACCTTCCCCCGCGACTCCAAGCCCACC
TCCGCCGGCAGCGTCACCCTGAACCCCGTGTTCAGCGAGACGACGGTGGACTGCAGCCGT
GGGTCCAACCAGGCGCTGCCCGGCACCTCGTGCCTCGCGCCCGGCTCGACCATCTCCCTC
GGACCCTTCAGCTCCACCCAGGCCGTCGGCTTCTACCTCAGGCAGAACTCCATCTGCGGC
GGCACCACCACCTTCTACTCGGTCGACGCCATCAACAAGGTCACCTCCCGCTGGAAGCCC
ATCCCCGCCGCCCACGGCCGCATGGTCGCCGTCCTCCGGGACCCCAACACGCTGCGCGCG
TACTTGGGCTGGGAAGACGCGCCCGACGGCAGTGACAGCGACTACAACGACAACGTCTTC
TCGGTGACCTCCAACTGCGAGATCGACGTCACCAAGCTCCCGTGCGCCACGGTCACCACC
TGCCGTAACTCCAAGCAGACCTTCGATTCCAACAAGTGCACGTGCTCCTGCCCCAACCCG
ACCACCTGCACGGCGCCCCAGGTCTACAGCACCGACCTCTGCGGCTGCACCTGCCCCAAC
GCCACCCAGACCTGCACCTCGCCGCTCACCTGGAACTCGGCCACCTGCCAGTGCGACTGC
CCCGCGACCAAGCCCCCCGGCGTAACCTGCTCCAACTTGCAGCAGTGGAACAGCGCCATC
GCCACCTGCGGCTGCAAGTGCCCCGACCCGGCCACCTACACCTGCAGCGACAGCCGCTTC
GTGCTGCGCACCTCCGACTGCACGTGCAACTGTCCGTCCACCGGCTCCTGCAGCGGCAAC
CTGCAGTGGAACTCGGCCAACAGCGTCTGCGGCTGCCAGTGCCCCGCCACCCCGCCCACG
CCCTGCTCCGGCAACCTCAAGTGGAACAGCGCCACCAACAAGTGCGCCTGCGAGTGTCCG
GCCACCGCGGCCCAGGCCGGCGTGACGTGTAAGGACAGCGAGGTGTGGGACACGGCCAGC
TGCTCGTGCAAGTGCCCCGCGTCGGCCAGCGCCGCGGGCAAGACCTGCCCCAACGCCAAC
TACCAGTGGAACTACAAGGGCAAGTGCGACTGCGGCTGCCCGGCGACGTCGGCCGAGGCC
GGCATCAACTGCGGCTCGCTCGGCCTCGGCAACACCGTGTGGGACACCACCTCGTGCAAC
TGCGCGTGCCCCCTGACCGGCACCTGCCCGGGCAACAAGGTGTGGAACCCGAGCAGCGAC
CCGGCCAAGTGCGGCTGCAGCTGCCCGGCCTCGGCCCCGGCGGACAAGGAGTGCAAGGGC
AACTTCTACTGGAACACCGACGAGGACGTGTGCGACTGCTACTGCCCGCTCGAGGCCCCG
GCCGACGACCCGTGCACGGGCTACACGACCTGGAACCGCACCGAGTGCGACTGCTACTGC
CCGCTCGAGCCGCCGTTCGAGGGCGGCTGCCCCGGCGTCCAGGTGTGGGACCGCGACCAG
TGCCAGTGTGTGTGCCCCGACGACGACCCCTGCGCCGCCCAGTCCACGGCCTGCAAGCAG
TACTACTGCAGTAGCTCGACCGGCGAGTGCACCCTGGTCTTCGAGGACACCTGCGCTAGC
CAGAAGCTCCAGTTCAACACCACCGGCTGCCTGTCCTGGCAGTGCGACCCCGATCTCGGC
TGCGTGCGCAAGGCCAACGGCTCCTGCTGCGAGGACTACAAGGACTGCCCCACGTGCGCC
AAGTACGACGGCTGCGACTGGATCGGCACCAAGTGCGCCGACAGCGGCCAGGTGCTGACG
TCGCCCATCGACCAGGCCGACCACCCCGATTGCTTCCCGAGCACGGGCCTCTCGGCCGGC
GAGACTGCCGGTATCACGGTCGGTATCGTGGCCGGCGTGACCGTCGGCGTCGGCGGCGCG
GCGGCCCTCTTCGGCGCGGGCTACGTCCTGTACAGGATGCTCAACAAGCCCCCGCCCCCG
GAGCAGCTGCCCACCATCGAGAACCTCGACACCGAGGCCGGCACCGACGACAACCCGCTC
TTCCACAAGAACGAGATCGAGATGACCAACCCGATGTTCTCGGCCGCCGGCGCCGGTGGC
GGCGGCGGCGACGCCGGTGCCATGTTCGCCCAGGGCGGCGGCGCCTCGGCGCTCCCTGCT
GACCTGCACACCCTCTAA
>g1406-299352-300071
ATGGCCCGTACCAAGCAAACTGCCCGTAAGTCGACCGGAGGCAAGGCCCCCCGTAAGATG
GCCTCCAAGTCGGCCAAGAAGAGCGCCCTCGCTGGAGGCGTGAAGAAGCCCCACAGGTTC
AGGCCCGGTACCGTCGCGCTCCGTGAGATCAGGAAGTACCAGAAGTCCACCGAGCTCCTC
CTCAGGAAGCTTCCGTTCCAGCGCCTCGTGAGGGAAATCGCTCAGGATTACAAGACCGAT
CTCAGGTTCCAGAGCGCTGCCGTCGCTGCCCTTCAGGAGGCTGCCGAGGCTTACCTCGTG
GGTCTCTTCGAAGACACTAACCTTTGCGCCATCCACGCCAAGAGGGTGACGATCATGCCC
AAGGATATCCAGCTCGCCCGCAGGATCCGTGGTGAGCGTGCCTAA
>g1406-596250-597294
ATGGCGGACGAGAACAGGGACTTCCCCGAGTACGAGGCTTGGGTCAAGAAGCAATCCGCC
TTTGCTAGGTGGGCTATCAGGACCAGGGCGGCCATTGCCGACCAGGATGAGACCAACAAC
GCTGAGTATCTCCGGCACGTCGCTAAAAACATGATCATCATGAAGGAGGAGAGCTACCTT
TGGAGCAGCTCCGCTCAGCGGCCCGACGACTACTACAAGGGTCGTTCCCCCCTGACTGCT
GCGTACAGGGACCACGGCATGTTCAAGAGCCCCTACGAGATCCGCGCCAAGGAGCTGCGA
GGCGAGCTGATTAGGTGGAACCACTGGCACTCGTGGGACCTCAAGCCGTCGGCGACGACG
AGCTACCCCAAGCCGCCCGCCAAGTACGATCCGCTCAACCCGACCCGCCCCAACATCCGC
AAGCAGCGCCTCGAACGCGAAGCCGCCCACTAG
>g1406-625513-626114
ATGAACCGCATCGCTCGCCCCGCTTTGCTCCGCGCGTCGGCCCGTGCCCCCGCCTTCGCG
CCCGTCGCCTCTTCCCTCAGATTCTCCTCCATTTCCTCATCTGCTGCCCTCTCCACCACC
TTCGCGAGGAGGAGCGATGCCATCACGACCTTCGATTACCAGAGGAGGCACTACGGCGCT
GCTGCCGGTCTGTCGGAGGCCGACGCCAGGGAGCGGGTCATCAAGGTGGTGAAGAATTTC
CACAAGGTCGACCCCAATGCCGTGAACGAGAAGTCGCACTTCATCAACGACCTCGGTCTC
GATTCGCTCGACACTGTCGAGCTCGTGCTTGGTCTCGAGGACGAGTTCTGCATCGAAATC
CCCGAGGAGCAGGCTGACAAGATCCAGACCATCGAGGATGCCGTCAGCTACCTCGTCACC
AACCCCGCTGCCAAATAA
>g1406-686343-687374
ATGTCTACCGCGCGTTTGCCCCCCAGAATTGTCAAGGAAACGCAACGCCTGATCAGCCAG
CCAGCCCCTGGCATCAGCGCTACCCCCCACCAGGATAACCTGCGTTACTTTGATGTTATT
ATCGCTGGCCCCTCTCAGTCGCCCTATGAGGGAGGCATCTTCAAGCTCGAGCTTTTCTTG
ACTGAAGATTACCCCATGAGCCCCCCGAAGGTGCGATTCTTGACGAGGATCTACCATCCC
AACATCGACCGACTCGGCCGGATTTGCCTCGACATTCTGAAGGACAAGTGGAGCCCCGCG
CTTCAGATCCGCACAGTTCTGCTCAGCATTCAGGCGCTGCTCAGTGCTCCCAATCCCGAC
GATCCCCTCGCCAACGACATTGCTCAGCACTGGAAGACCAACGAAAAGGGAGCCATCGAT
ACTGCTCGGGAGTGGACGAGGAGGCACGCAATGTCCGACTAG
>g1406-699622-700664
ATGAAGAGGACTGCGATTTCTGCGGCTGCGACGCTGAGGGCCAGCACTGCGGTGAGCGGC
TCGGCCTCCGCCACCCTTACCCGCATGGCTCCCGTCCTGAGCTCCGGGTCGAGCAAGCGC
GAGGTGCACGAGATTAATGTTCCCGATTGGGGCCACTACAAGCGCGAGGCGCAGGAGGGA
GGCGAGAGTGGAGGCCGAGCCTTCAGCTACATGGTGCTCGGTACCGCTGGTGTGGGCTAT
GCCGCCGCGGCCAAGCACACCGTCATCAAGTTCCTCGATTCGATGAATCCCGCCGCCAAT
GTGCGCGCCATGGCCAACGTCGAGGTTGATGTCTCCAACATCGCCGAGGGCACCATCATG
ACGGTAAAGTGGAGGGGCAAGCCCCTCTTCATCCGTCACCGCACGGCCGAGGAAATCGCC
GATGCCGAGTCGGCGCCCCTCACCGACATGCGCGATCCCTGCCCCGATGCCGCCCGCAGG
AAGGCCGACAAGCCTGAGTGGCTCATCGTGCTCGGCGTGTGCACCCACCTCGGCTGCGTG
CCCCTCGGTGGCCAGGGCGAGTATGGTGGTTGGTTCTGCCCTTGCCACGGTTCTCACTAC
GATACTGCTGGCCGCATCCGTAAGGGTCCCGCCCCTCTGAACTTGGAGGTGCCGCCCTAC
GTGTTCAAGGATGACTCCAAGGTGCTCGTCGGCGTGGACTCTGTGTAA
>g1406-734565-736554
ATGCCGAAGGAGACGCGACTTTATGATCTCCTTGGGGTGCGGCCAGAGGCCACTGAGGCC
GAGATCAAGAAGGGGTACTACAAAATGGCCAAGGAATTCCATCCCGATAGGAATCCGGAA
GCTGGCGACAAGTTCAAGGAGATCAGCTTTGCCTACGAGGTCCTCTCTGACGCAGAGAAG
AGGCAAGTGTACGATCGTCACGGTGAGGAGCGTCTCAAGGAGGGCGGCGGTGGCGGTCCC
GGCTTCGGTTCTGCCGAAGACCTCTTCTCCTCCATCTTTGGAGGCGGCTTCTTCGGTATG
GGCGGCGGCGGCGGCGGACGACGACGCCAGAGGCGCGGCGAGGACCTAGTGCACCCGCTC
GAGGTGACGCTGGAGGATCTCTACAACGGCAAAGCGACCAAGCTCGCCCTCCGCAAGAAC
GTCATCTGCACCGTCTGCGCCGGAAAGGGAGGAAAGAACCCCGACTCGGTGAAGCCGTGC
GACGGGTGCAAGGGCAGCGGCGTGAAGGTGACGCTGCGCCAGCTGGGTCCCGGGATGGTG
CAGCAGCTGCAGAGCGTGTGCACGAAGTGCCGAGGACAGGGTGAGGTGATCAAGGAGAAG
GACCGCTGCAAGAAGTGCCGCGGCAACAAGGTGGTGCAGGAGAAGAAGGTGCTCGAGATC
TTTATCGACAAGGGCATGCGGCACAAGCAGAAGATTGTCTTCTCGGGCGAGGGCGACCAG
GAGCCGGACGTGATTCCGGGAGATGTGATCATCGTGCTGCAGGAAGCCGAGCACCGCACG
TTCAAGCGCAACGACGCCGACCTGCTGGTGGAGCACGAGATCACGCTGTTCGAGGCGCTG
TGCGGCTTCGAGTTTGTGCTCACGCACCTCGACGGCCGCACACTGCTGATCAAGTCCAAC
CCGGGCGAGATCATCAAGCCGGGCGACGTCAAGGAGATCCCCGGCGAGGGCATGCCCATG
TGGAAGCACCCCTTCGACAAGGGCCTCCTCATCATCAAGTTCACCATCAAGTTCCCCGAG
GCCATCTCCCCAGACGCCGCCAAGGTGCTCCAGCGCGTGCTGCCCAACCCCGAGCCCCTC
CCGCAGCTGGAAATGGGCGAGTACGAGGAGGTCATGCTGCAGGACTATGGCACGTCGGAG
CGCGCGAGCGGCAACCACGGCAACGCGAGGCGTGAGGCCTACGACGAGGACGAGGAGCAC
GGCGGCTCCCGCCTTGACTGCACCCAGCAGTAG
>g1406-738572-739089
ATGTCGCTCCAGGAGAACTTCGACAAGGCCGCCGCCGAGGTGAAGGCTTTCAGCAAGAGG
CCCGACGATTCCGTCCTCCTTCAGTTGTACGGTCTCTACAAGCAGGCCACCGCTGGCGAC
AACACCACTGAGGCTCCCTGGGCCATCCAGTTCGAGGCCAAGGCCAAGTGGGACGCCTGG
AACGCCAACAAGGGCACCTCGCAGGACGATGCCAAGCAGAAGTACATCGACCTTGTCAAC
CAGCTCAAGACCGAGTTTGCCTAA
>g1406-788292-789280
ATGTCCGAGACTGAGCTTAAGCCTGAGGAGAAGGTCACCGTGACCGAGGGTGAGGAGAGC
GACGATGAGATCCCCGAGCTCGAGAGCGCCCCTGAGGCCGCCCCCGCCGCTGGCGCTGAG
GCTGAGGAGGGTGCTGGCAAGCACTCCCGCAGCGAGAAGAAGTCGCGCAAGGCCATGGCC
AAGCTCGGCCTCAAGCCCGTCACTGGCGTCGTCCGCATCACCGTCCGCAAGTCCAAGGCC
GTGATGTTCGTGATCACTCAGCCCGACGTGTACAAGAGCCCCGCTTCGGACACCTATGTG
TTCTTCGGTGAGGCCAAGATGGAGGACTGGGGCAGCCAGGCTCTTGCCGACACTGCCAAG
CAGTTCGAGAAGGCCGTCGGTGGCCAGCAGGCCGAGGGTGCCGCTGAGCAGAAGGAGAGC
AAGGCCGCCGCTGAGCCCGAGGGTGAGGTCGACGCTGGCAACCTCGAGGAGAAGGACATT
GAGCTTGTCATGTCGCAGGCCTCCGTCAGCAGGGCCAAGGCCGTGACCGCCCTCAAGAAG
ACCAACGGTGACATCGTGAACGCCATCATGGAGCTCACCATGTAA
>g1406-797858-798584
ATGTCCTGGCAGACGTACGTCGACACCAACCTCGTGGGCACTGGCGCCGTGACTCAGGCC
GCCATCCTCGGCCTCGACGGTAACACCTGGGCCACCTCTGCTGGCTTCGCCGTGACCCCC
GCTCAGGGCCAGACCCTCGCCAGCGCCTTCAACAACGCCGACCCCATCCGCGCGTCTGGC
TTCGATCTCGCTGGTGTCCACTACGTGACCCTCCGCGCTGACGACCGTTCCATCTACGGT
AAGAAGGGCTCGGCTGGTGTCATCACCGTCAAGACCTCCAAGTCCATCCTCGTGGGCGTC
TACAACGAGAAGATCCAGCCCGGTACCGCCGCCAATGTGGTGGAGAAGCTCGCTGACTAC
CTCATCGGTCAGGGCTTTTAA
>g1449-20456-24212
ATGGCTCACGTTGCGTCGAAGAGGGTTTCGCTCGATGAGATCTGGCCCGACCTCGAGGAT
GGCATTACGTCGCTCGTCACCAACCTCAATGCAGGCTTCCCCAGGAAGAGATGGATGGAG
CTCTACTCAAACGTGTACAACTACTGTACCACTTCACGACCCGCGCCTGGCCGAAAGCCT
GGTGGCATCTCTGGTGCCAATTTTGTGGGCGAGGAGCTGTACACTCGGCTGAGCGAATTC
CTGAAGAATCACATGAAGAAGCTGTGCAAGCAGGCTGAGGGCAAGATGGACGACGATCTG
CTCATGTACTACAGCAAGGAATGGGAACGATTCACCACTGCCATGAAGTACATCAACAAC
ATCTTCGACTACCTGAACCGTCACTGGATTAAGAGGGAGGCCGACGATGGCAAGAAGGAG
GTCTACGATATTTACACGCTTTCGCTGGTCATCTGGCGCGACCACATGTTCCACGCGCAG
AAGGGCAGGCTCATCAACTCCCTGCTCGGCCTCATCGAAAAGGAGAGGAACGGTGAGCAG
GTCAACACGCACCTGCTGGGCACTGTCATCCAGGGCTACGTCAAGCTCGGCTTGAACAAG
GAGAAGCCGAAGGAGAACACCCTTGAGATCTACCGCCAGTTCTTCGAGGACGACTTCATC
ACCGCCACGGAGGTCTACTACACGGCCGAGTCGACCCACTTCATCGCCACCAACAGCGTG
GCCGAATACATGAAGAAGGTGGAGACCCGCCTCGACGAGGAGACGAGGAGGGTGCAGCAG
TACCTCAACCCCACCACGGAGACCGAGCTCGTCCAGAAGTGCGACCGCGTGCTCATCGAG
AAGCACCTCGACACCATTCGCTCCGACTTCCAGAACATGCTCTCCAACGACAAGATCGAA
GACCTGACGCGCATGTACAAGCTGCTTTCGCGCATCAATCGAGCGCTCGACCCGCTGAAG
ACCACGTTCGAGAAGCACGTGAGCCAGGTGGGCAAGCAGGCCATCCAGCAGGTGATGAAG
ACCGCCATCAAGGACCCGCAGCAGTACGTGGAGACCATCCTCAAGGTCTACAAGCAGTAC
AACGCCCTCGTCATCGGTTCGTTCCGCAACGACCCGGGCTTCGTCGCCGCGCTCGACAAG
GCCTGCCGCAGCTTCATCAACGAGAACGCCGTCTGCAAGCTCGCCAAGTCGGCCTCCAAG
TCGCCCGAGCTGCTCGCCAGGTTCGTCGACTCGCTGCTCAAGAAGAGCGCCAAGAACCCC
GAGGAGCAGGAGATGGAGCAGCTCTTGACCGACGTCATGACGGTGTTCAAGTACATTGAA
GACAAGGACGTGTTCCAGACGTTCTACTCCAAGAACTTGGCGAAGCGTCTCATCCACGGC
ACGTCGGCCTCTGAGGACCTTGAAGGCACCATGATCGGTAAACTGAAGTCCGCCTGCGGT
TACGAGTACACCTCGAAGCTGCAGCGTATGTTCACGGACATGTCGACGAGCAGGGACCTT
CTCGAGCGGTTCAAGAACCACCTCGAGGACTCCAGCCAGACCTCCGCGGTCGGCCTGGAC
TTCAACGTGCTGGTGCTGGCGACCGGTTCTTGGCCGCTGCAGCCGCCCTCGACCAACTTC
ACGATCCCCAAGGATCTGGTGGCGTGCGAGCAGCTCTTCGTCAAGTTCTACCAGCAGCAG
CACTCTGGCAGGAAGCTCAACTGGCTGCACCAGCTGTCCAAGGGCGAGCTGAGGGCGCGC
TACGGTAAGAAGTACTCGTTCGTCACCTCGACCTACCAGATGGGCATCCTCCTCCAGTTC
AACTCCAACGAGTCCATGACCACCGACGATCTCCAGATCGCCACACAGCTCACCCAGAGC
ACCCTCGTCAACACCCTCGCGACCCTGGTGAAGACGAAGGTGGTGAAAATGGACCCGGCC
CCTCCGCCGGAGGAGGAAGTGCCCAAGATCGAAAAGGACGCCGTCTTCACGATCAACGAG
AACTTCAGGAGCAAGAAGCAGAAGGTGAACATCAACATCCCCATGAGGAGAGAAAAGGAG
GAGGAGAACGAGGAAACGCACAAGGCCGTCGAGGAGGACCGCAAGCTGCAGATTCAGGCC
GCCATCGTGCGTATCATGAAGATGCGCAAGCGCTCGCAGCACGGCGCGTTGATGTCGGAG
GTGATCGCGCAGCTGCAGACGAGATTCAAGCCTCGCGTGGCCGTCATCAAGAAGTGCATC
GACATCCTCATCGAAAAGGAGTACCTCGAACGCGTCGAAGGCCAGAAGGATATGTACAGC
TACGTCGCGTAA
>g1449-118768-120377
ATGGCGACCAGCACTCTTGCCCCCTACCTGAACTGCATCAGGCAGACCCTCACTGCGGCC
ATGTGTCTTGAGAACTTCGCTTCTCAGGTGGTCGAGAGGCACAACAAGCCCGAAGTCGAG
GCTAGGGCCAACAAGGAGCTGCTGCTCAACCCTGTAGTTATTAGCCGCAACAAGAACGAG
AAGGTCCTCATCGAGGGCTCGATCAACTCCATCAGAATTTCCATCTGCATCAAGCAGGCC
GACGAGCTCGAGACCATCCTCTGCAAGAAGTTCATGCGCTTCTTGGCCCAGCGCGCCGAG
AACTTCGTCGTCCTCAGGAGGAAGCCCATCAAGGGCTACGACATCTCCTTCCTGATCACC
AACTTCCACACCGAGTCCATGTTCAAGCACAAGATCGTGGACTTCATCATCCAGTTCATG
CAGGACATCGACAAGGAGATCAGCGAGATGAAGCTCGGCGTGAACGCTCGCGCCCGTATC
GTCGCCCAGTCCTACCTCAAGGAATTCATGTAA
>g1449-177097-181210
ATGCAGCAGCCACCAGCACGTCGCTATTCCATGGACCGGTTCGCGAGCGAGCTCGACTTC
ACCGACACGAGGCCGGTGCTGTTCGACGTCGGGTGGGAGGTGGCCAAGAAGGTGGGCGGC
ATCTACACCGTGCTCAAGTCCAAGTCGCCCATCACGGTCGAGGAGTTCGGCGGCCGCTAC
GCGCTGGTCGGCCCCTACAACCAGGCCACCGCCGCCACCGAGATGGAGGAGATGGAGCCC
GGCCCGCTCTTCGGCCCCGTCATCGCCCGCCTCCGCGAGCGCTACGGCATCATCGTTCAC
TTTGGCAAGTGGCTCGTCGACGGTTATCCCAAGGTGCTCCTGATCGACGTGAACAGCTCG
ATGCATGAGCTGGACAAGTGGAGGGGCGAGCTCATGGGCGGCTGGGACGTGCCCGGCGAC
TTCGAGTGCAACGACGGCATCGTGTTCGGCTACCAGACGGCGCTGTTGATCAGGGAGTTC
ACCCAGATCTACCGCGACATCCGTCTCATCGCCCACTTCCACGAGTGGCAGGCCGCCGTG
GGCCTCATCGTCGCCCGCATTTGGAACGTCAAGTTCGCCAGCATCTTCACCACCCACGCC
ACCCTCCTCGGCAGGTTCTTGGCCTCTGGCGGCGTGGACCTGTACAATCAGATCGCGCAG
ATCAACGTCGACTCCGAAGCCTCCCGCCGCGGCATCTACCACCGCCACTGGATCGAACAG
CGCGCCGCCCAGGAATGCCACGTATTCACGACGGTGTCGGAGATCACGGCCTACGAGGCC
GAGCGGATCCTGCGGCGGCGCGCGGACGTGATCGTGCCCAACGGTCTCCAGGTGAAGAAG
TTTACGGCGCTCCACGAGTTCCAGAACCTGCACGCCAAGTACAAGGAGGTGATCCACGAG
TTCGTGCTGGGCCACTTCTACGGCCACTACAACTTCGACCTCGACAACACGGTCTACTTC
TTCACCTCGGGCCGCCACGAGTACTTCAACAAGGGCGTCGACATGTTCATCGACGCGCTG
GCCGGCCTCAACTACCTCCTCAAGCAGCGAGGGTCCAACATCACCGTCGTCGCCTTCATC
ATCATGCCCGCCCCGACCAACAACTTCAACGTCGAGTCCCTCAAAGGCCAGTCGTTGATC
AGGGATCTGAAGCGTACGACGACGCACGTGGTGGAGAAGCTGTCCGACCGCATTTTCGAG
GCCACGGCCCGCGGCCAGATGCCCGACGTCAACGATCTCCTCAAGGAGGAGGAAATCGTG
ATGTTGAAGAGGCGCATCTACACGGTGAAGCAGCGTAACATCTACCCGCCCATCGTCACC
CACAACATGATCAACGAGGGCAAGGACGAAATCCTGAACCACCTGAGGCGCGTCAAGCTG
TGGAACGACCCCGCCGACCGAGTCAAGGTGATCTACCACCCGGAGTTCCTGTCGTCGACT
TCGCCGCTCATTCCGCTGGACTACCCCGACTTCGTGCGCGCCTGCCACCTCGGCATCTTC
CCCTCCTACTACGAGCCGTGGGGTTACACGCCCGCTGAGTGCACGGTGCTCGGTGTGCCC
TCGATCACCACCAACTTGTCCGGTTTCGGTAACTACGTGCATACCAACATCGAGGACCAC
GACTCCAAGGGTCTGTTCATCGTCGACAGGCGCTTCAAGGCGCCCAATGAGACGGTCGAC
CAGATCATCGACATTCTCTGGAAGTTCTGCCAGCTCGACCGCAGGCACAGGATTGAGCTG
CGCAACCGCTGCGAGCAGATGTCGCAGATGCTGGACTGGAAGAACCTGGGCAAGGCCTAC
CAGGAGGCCCGCCACCTCGCCCTCGAGAGGGTCTACGGCCCCGTCGACCAGCCCGACATC
GGCGCCCTCAAAATCAGCTCGTAA
>g1449-211717-214488
ATGGACGGAAAAGACGGCAGGAAGAAGGACAAGAAGCATAAGAAGAAGGACAAGAAGGAC
AAGGAGATTGAGGAAGCCATGGATCAAGGAGGCGAGGAGCAGCAGCAGATCGAGGGCACC
GAGACCGAGGTCTTGGAGCCTGAAGCCAAGAACATTCTGCTCCATCTGCTGTCGCAGGTG
CGAATTGGCATGGATCTCTCGCGCGTCACGCTCCCCACCTTCATCTTGGAGCCCAAGTCG
TTCCTGGAGAAGCTCACCGACTTCTTCACTCACCCTGACGTCATGCTCAGCTCGGTTGAG
TGCCAGAGCCCCGTTGAGCGGCTGGTCGCCATCACTCGTTGGTATCTCTCTGGCTTCTAC
ATTCGCCCCAAGGGAGTCAAGAAGCCCTACAACCCTATCCTGGGCGAGATCTTCCGTGCC
CAGTGGGATCACGGGACCTCCAAGTCGTTCTACGTCTCGGAGCAGGTGTCGCACCACCCC
CCAGTGAGCGCCTTCTACGCCTCCAACAGGGCCGTGGGGTTGGCCATCAATGGCTACATC
AATTTCCGCTCCAAGTTCATGGGTAACTCCTCCGCCGCCATCATGGACGGCGAGGCCGTC
TTGTACTTCCTCCGTATCCCCGGCGAGTCCTACACCATCACCTTCCCCACTGCCTACGCT
CGAGGTATCCTGTGGGGCACACTGCTGATGGAGATGGGCGGCACCGTGTACATCACCTGC
GAGAAGACCGGACTCCAGTCCGAGATCGAGTTCAAGACCAAGCCCTACTTTGGTGGCGGC
TACAACTACGTGGCGGGCAAGATCAAGGAGACCTCCGGCAAGAAGAAGACTCTGTACACA
ATCTCTGGCAAGTGGGATGAGGAAATTTTCATCAAGCCGGCCGACACCAAGAAGGACGAG
CTTCTCTGGGATCCCAAGAACGCGCCACCCAAGGTCACCATGGACATCAGGCCGCTCGAC
AAGCAGGACGAGTGCGAGTCGCGAAGGCTGTGGCAACACGTTTCCGCCGCGCTGAGGAAG
AACGACCAGGAGACCGCCACCACCGAGAAGTGCAAAATCGAAGACGCGCAGAGGGAGGCC
GTGCGCAGGAGGGAAGAAGAGAAGGAGACATACGAGGTCAAGTACTTCGAGAAGGACGAG
AATGGTGGATGGGTATACAAATGGAAGAATCTCGAGCCATTCCCCCTGGACGAGTTGGAC
CAGTGGGAGGAGTACGAGGAGGACTGCGTCATCGGCGCCAGGAAGAAGTGA
>g1449-304348-305698
ATGTCCAAGGGCGGCGGTAAGTTTCAGAGGAAGAAGGAGTACTTCACCAAGCTCTCCAAC
CTCCTGGTCGATTACAAGAAGGTGCTCATCGTCGCGGCCAACAATGTCGGCTCCAACCAG
CTTCAGAGGGTGAGGCAGGAGCTCCGCGGCAAGGCCGTCCTCCTCATGGGCAAGAACACC
ATGATCAGGAAGTGCATCAGGGAGAACCTGACCAAGAACCCCGACCTCGAGGCCCTGCTT
CCCTACGTTAAGGGCAACGTCGGTTTCGTCTTCACCAACGGCGATCTGTCGGACATGAGG
ACCAGGATCGGTGCCGTCAAGGTCAAGGCTGCCGCCAAGTCTGGTGCCATCTCTCCGTGC
GACGTCATCGTGCCTGCCGGCCCCACCGGCCAGGATCCTGCCAAGACCTCGTTCTTCCAG
GCGCTCACCATCTCCACCCGTATCTCCAAGGGTGTGATTGAGATCGTCAACGACGTGCAC
CTCGTGAAGGAGGGCGCCAAGGTGACTGCCTCGCAGGCTGCTCTGCTGCAGATGTTGAAC
ATCCAGCCCTTCGAGTATGCCCTGGCCGTCAAGACTGTCTACGATGATGGTTCCGTCTAC
CCCGTGTCTCTCCTCGACATTACCGACGACGACATCATCAACAGGTTCCGCAAGGGTGTC
GCCAACGTGGCCGCTATCTCGCTCCGCATCGGCTACCCCACCGTGGCTTCCGTCCCCTAC
GCCCTCACCAGCAGCTTCCGCAACCTGCTCTCCGTGTCGCTCGCCACCAGCTACACTTTC
CCCCAGGCCGAGAAGCTCAAGGACCTCCTCGCCAACCCCGAGGCCTTTGCCGCTGCCCTC
GCCGCCGCTGCTCCCGCCGGCGGTGCTGCCCCCGCCACTGCGACTTCGGCTGCTGCGCCT
GCCGCCGCCGCCGCCAAGGTCGAGGAGGAGGAGGAAGCCGAGGACATGGACGCTGGTGGC
CTCTTCGGTGGCGGTGACGATGACTACTAG
>g1450-322366-327048
ATGTCGAGTTCTTCAGACTCGGACGAGCCTCCGAAACACGAGGAGGCCTCCCTCAGTAGC
GACAACGAAACGAAACAGGCGAAGAGCAGCACCGACAAGAAGAAGAAGTCGAAAGGCAAA
CACAGCAAGAACAAGGACAAGAAGAAGGGAAAGTCTTCATCGGATGAAGAGTCGTCCGAT
CACGACGACGATAATCCGCAAGACAAAACGGCAGCTAGGAGCGCTAAATTCACGACCAGG
GTTGACGAGGTTCTTGCGGGCATGGGCGCTGGCAACACCCGCGACGGCCTTACTGACGAA
GAAGCTGCGAAACGACTACTCGAGTTCGGCCCGAACGCGCTGCCAACCAAGAAGGAGAGT
CTCATCCTCAAGTTTTTGTACTTCTTCTGGAATCCTCTTTCCTGGGCGATGGAGTTTGCG
GCCCTCCTTTCTTTCGTGCTGGTCGACTACGTGGACGGCATTCTGATCACCGCGCTCCTC
CTCCTCAACGCCTGCATCGGTTTCTATGAGGACTACTCGTCCGGCAATGCCGTGGCCGCG
CTGCAGAGCGCGCTCGCCCCGACTTGCAAGTGCCTGCGTAACGGCGAGGTCGTGGCCGGC
ACGGCGTCCGTCGGCCTCGTGCCCGGCGACGTGGTGCTGCTGCGTCTGGGTGACGTGGTC
CCGGCCGATTGCTTCATCCTCGACGACGGCGACTCCCTCAAGATCGATCAGTCCTCCCTC
ACCGGCGAGTCTATCCCCGTGGATCGCTTCCCCGGTGACGAGATCTATTCGGGTTCTATT
GTCAAACAGGGTGAGATGAAGGCCATCGTGCACGCGACGGGCCTGAGCACGTTCTTCGGT
AAGGCCGCTGACCTGGTGAACCGCTCGGAGAAGAAGTCGCACATCCACCTGGTCCTGAAG
TCGATCGCCTACTTCTGCATCATCTTCATCATGGTCGGCGTGGTGGCCGAGCTCATCACC
CAGTTCGCCATCCGCGACAAGCCCTGTACCGGTGTGTCCGACGGCGACTGCGCGCCGCTG
AACAACATCCTGGTGCTCGTGGTCGGCGGTCTGCCGATTGCCATGCCCACCGTGCTGTCC
GTCACCATGGCCCTGGGCGCCTCGGCGCTGGCGAAGAAGAAGGCCATCGTGTCGCGTCTC
ACCGTCGTGGAGGAGATCGCCGGCATGGAGATCCTGTGTTCCGACAAGACCGGCACCCTC
ACCAAGAACGAGCTCTCCGTCAAGGACCCCGTGGCCTACGTGGGCGACCTCGCCGACGTC
ATCTTCGACGCGGCCCTCGCCGCCAAGCCCGAGAACGGCGACGCGATCGACATGGCGATG
GTGGGCTACCTGACCGACGAGCAGAGGGAGCAGCGCAAGAAGTTCAACGTGCTCCACTTC
CACCCGTTCGACCCGGTCGGCAAGAAGACCGTGGCCAAGCTGCAGAGCCCCGACGGCGAG
ATCTTCCACGCCACCAAGGGCGCGCCGCAGGTGATCCTCAACCTGTCGGAGAACAAGAAG
AAGATCAAGGACCGCGTCATGGCCGACATCGAGACCCTCGGCAAGGCCGGCTACCGAACC
CTCGGCGTGGCCATCTCCGACGAGCACGGCAAGAAGTGGACCATGACCGGCCTCATCCCC
ATGTTCGATCCGCCGCGCGACGACACGGCCGACATGATCGCTAAGACCGAGGGCCTGGGC
GTCGGCGTCAAGATGATCACCGGTGACCACCTGACCATCGCCAAGGAGACGGCCAAGCTG
CTGGGCATGGGCAGCAACATCTTCCCCGCCGCCTACATGAAGGACGAGGCCAAGGCCCGC
AACGAGACCGGCATGAGCATCTACGACATCGTCTGCGAGGCCGACGGCTTCGCCGAGGTG
TTCCCCGAGGACAAGTACACGATCGTCGAGTACCTGCAGCGCGGCAGCCGCATCGTCGGC
ATGACCGGCGACGGTGTCAACGACGCGCCCGCCCTCAAGAAGGCCAACATCGGTATCGCC
GTGTCGGGAGCCACCGACGCCGCGCGCGGTGCCTCGGACATCGTGCTCGCCGAAGAAGGT
CTGTCCGTCATCGTCGACGCCATCCTCGGTTCCCGCAAGATTTTCCAGCGCATGAAGAAC
TACTGCATGTACTCGATCTCAGTGTGCGTGCGTATCGTGTTGACGTTCGGTATCCTTACC
CTGGCCTACGATTGGTACTTCCCGACCATCGGCTGCGTGCTCCTCGCCATCTTCAACGAC
GGCTCCATGTTGACCATCTCCAAGGACAAAGTCAAGCCTTCAAAGGAGCCCGAGCACTGG
AACCTGCTGGAGATCTTCGGCACGGCCATCGTCCTGGGCACCTACCTCACCATCTCGACC
ATCGTGCTCTTCCATCTCGCCGTCTACACCGACAGCTTCGAGCGTTGGTTCGGCCTGCCC
CACTTGACCGCCGCCGAAGCCCGCGGCCTCATCTACCTCCAGGTGTCCGTCTCCGGTCTC
TCGACCGTCTTCGTGACGCGTACGCACGGCCTGTCGTGGCTCTTCTGGAGAGAGCGACCC
GGTCTCGCTCCCGTGATTGCCTTCATCATCGCCCAGACTGCCGCCACTATCCTGTGCGCC
TACGGTTTGAACGGTTTCCCCGACGACAAGGAGACTGACTTCGAGGGCGCTGGCTGGTGG
TATGTGCTGGTCGGCTGGATCTGGTGCATCATCTGGTTCCCCGTGATGGACATCCTCAAG
ATCGTCGTCCGCTCCGTCATGAAGGGCGAGATCTTCCTGTTCAAGCACAAGCTCTCGCTC
CACTTCCAGCTGGTCCACGGCCACCCTTACCACGGCCAAGCTGCGGGCAACGCCCACCCG
GACGAGTGGGAGGAGCACGTCGTTACGGCGGGCGACTTGGCCCACATTCCCCAGCAGCTC
AAGTCCAAGATGATGGCCAAGTTCAAGCACAGCGAGGACGGCAGCGACGACGAGAGCGCC
AAGAACGGCGCCGCCAGCGGATCGAGCTCCGCGGTGCCGTCCAAGCGCAGCTCCAGCAAC
AACCTCACCAGGAACAGCGCCTCAGGCCGGAAAGCCAGCGGCCTGAGCAAGAGCCTCAAG
CCCGAGGAGAAGAAGCTCAAGAAGCACAGCGACGAGAGCGACGCCACGAGCGACGACTAA
>g1450-363687-366568
ATGCTGTACCCCCAGGCGGTCAGGAGATGGCTCGCATCTTCTTCTTCGAGCATCGAGGCC
TCGACATCCAACAAGAAGCTGATTGAGTGGCTCAAGACCCAGCAGAAGCTGCTCCAGCCC
AGCGCCGTGCACATCTGCGACGGATCGGTCGAAGAGGACGAACGCTTGAAGAGCGAGCTC
GTCAAGAGCGGCACGCTCATCAAGCTCAACGAGGCCAAGCGGCCCAACTCGTACCTCGCT
CGCTCGGACCCTGGTGATGTTGCGCGCGTAGAGGGCCGCACGTACATCTGCTCGACCAAC
AAGGCCGACGCAGGCCCGACCAACAACTGGGTGGATCCGAAGGAGATGCGGGCCACGCTC
AACGGCAAATTCGCTGGCGCGATGAAGGGCCGCACGATGTACGTGGTCCCCTTCAGCATG
GGTCCCGTGGGCTCGCCCCTTTCCAAGATCGGCGTGGAGATCACCGATTCCCCCTACGTC
GTGGCCAACATGCGCATCATGACGCGCATGGGCAAGCAGGTGTTTGATGCGCTGGGCAGC
GACGGCGACTTCGTGCCGTGCGTGCACTCTGTGGGCGCGCCGCTGCAGCCGGGCCAGAAG
GACGTGCCGTGGCCGTGCAACCCCGACAAGTACATCGTCCACTACCCCGAGACCCGCGAG
ATCTGGTCCTACGGCTCCGGCTACGGCGGCAACGCCCTCCTCGGCAAGAAGTGCTTCGCG
CTCCGCATCGCCTCCGTCCAGGCCCGTGATGAAGGCTGGCTGGCCGAGCACATGCTCATT
CTGGGCATCACCAACCCCGAGGGCAAGAAGAAGTACATCGCCGCCGCGTTCCCCTCGGCT
TGCGGCAAGACCAACCTCGCCATGATGAACCCCACCCTCCCCGGATGGAAGATCGAGACC
GTCGGTGACGACATCGCCTGGATGCGGTTCGGCAAGGATGGTCGTCTCTACGCGATCAAC
CCGGAGAACGGATTCTTTGGCGTGGCGCCCGGCACGTCGATGTCGTCGAACCCCAACGCC
ATGGCCACGACGGTCAAGAACGCGCTCTTCACCAACTGCGCCCTCACGGACGACAACGAC
GTGTGGTGGGAGTCGATGACCGACAAGCCGCCCGCGCACCTGATCTCGTGGAAGGGCAAG
GACTGGACGCCCGAGTCCAAGGAGCCCGCGGCCCACCCCAACGCCCGCTTCACCACGCCC
GCCGACCAGTGCCCTGTGCTCGACTCCAAGTGGGAGGACCCGCAGGGCGTGCCCATCTCG
GCCATCCTCTTCGGCGGCCGCCGCCGCACGGTGGTGCCGCTCGTGTACCAGGCCTTTGAC
TGGGAGCACGGCACCTACATTGGCAGCTCGATCGGCTCCGAGGTCACCGCCGCGGCCGAG
GGCAAGCGCGGCGAGATGCGACACGACCCGTTCGCCATGCTGCCCTTCTGCGGCTACAAC
ATGGCCGACTACATGCAGCACTGGCTCAACGTCGGCTCCCGCTCCGCGGCCGACAAGCTC
CCCAAGATCTTCTTCGTCAACTGGTTCCGAAGGGGAGCTGATGGCTCGTTCCTGTGGCCC
GGCTACGGTGAGAACTCGCGCGTGCTGAAGTGGATCTTCGACCAGACGGACAACAAGTCC
GGTAATGCCAAGGAGACCCCGATCGGATACATCCCGAAGGAGGGCGCCCTGGACACGTCG
GGCCTCGACGTCTCGCCGGAGGCCATCAAGCAGCTCTTCGAGGTCAACCCGTCCGACTGG
AAGGAGGAGGTCCAGGAGATGAGGCGATACACCGAGCAGTTCGGCGATCGCCTGCCCGCC
GGCCTCAAGAAGCAGATGGACGCCCTCGAGAAGCGCCTCGCCTAA
>g1450-375617-377598
ATGGCAGCCACGGCGGACATCGGTTTGGTGGGTTTGGCGGTCATGGGCGAGAACCTCGTG
CTCAACATGCTCAACCACGGCTTCACTGTGGCCGTGTACAACCGCACCACGTCCAAGGTG
GACAACTTTGTGAGCGGGCGTGCGGCGGGCAAGAGCGTGGTCGGGTGCCACAGCCTGGAG
GACCTGGTGAAGAGCATCAAGCGGCCGCGCAAGGTGATGCTGATGGTGATGGCGGGCGAG
GTGGTGGACATCTTCATCAACGCGCTGGCGCCGCTGCTCGAGGAGGGCGACATCATCATC
GACGGCGGCAACTCGCACTTCCCGGACTCGAACCGGCGCACGCGGGACCTGGCGGCGCGT
GGGCTGCACTTTGTGGGCGCGGGCGTGTCGGGCGGCGAGGAGGGGGCGCTGCTGGGGCCG
TCGATCATGCCGGGCGGGGCGTGCGAGGCGTGGGAGGCGGTGAAGCCCATCTTCCAGGCC
ATCGCGGCGCGCGTGGAGGACGGTTCGCCGTGCTGCGACTGGGTGGGCCAGGGCGGCGCG
GGCCACTTTGTGAAGATGGTGCACAACGGCATCGAGTACGGCGACATGCAGCTCATCTGC
GAGGTCTACTTTGTCATGAAGCACCTGCTGGGCATGGGCAACGACGAGATGCACGCCGCC
TTCGCCGAGTGGAACCGCGGGGAGCTCAACAGCTACCTGATCGAGATCACGGCCGAGATC
TTGGCCAAGCGGGACGACGAGCAGCCCGACGCGCACGTGGTCGACCTCATCCTCGACACG
GCGGGCCAGAAGGGCACCGGCAAGTGGACCGTCGAGGCCGGCCTCGACTTTGGCGCGCCC
GTCACGCTCATCGGCGAGGCCGTCTTCGCGCGCACCCTCTCCTCGCTCAAGGCCGACCGC
ACCGCCGCCGCCGCGGTCCTGGCCGGCCCCGCCGGGCTGCCGCCCTTTGCCGGCGACCGC
GCGACCGTGCTGGGCCACCTCAAGGACGCCCTCTACGCGTCGAAGCTGGTCTCCTACGCG
CAGGGCTTCATGCTCATGCGCGCCGCCGACGCCAAGTTCGACTGGAAGCTTAACTACGGC
GGCATCGCCCTCATGTGGCGCGGCGGCTGCATCATCCGCTCCACCTTCCTGGGCAAGATC
AAGGAGGCCTTCGACCGCGACCCCGCCCTCACCAACCTCCTGCTCGACCCCTTCTTCCGC
GACCAGATCGACAAGGCCCAGGCCGGCTGGCGCAAGATCACCGCCCTCGCCGTCGAGCAC
GGCGTGCCCGCCCCGGCCATCGCCTCGGCCATCTCCTTCTACGACGCCTACCGCACCGCG
CGCCTCCCCGCCAACCTCCTCCAGGCCCAGCGCGACTACTTTGGCGCCCACACCTACGAG
CGCGTCGACCGCGACCGCGGCCTCTTCTTCCACACCAACTGGACCGGCCGCGGCGGCGCC
ACCCACTCCACCACCTACAACGTGTAA
>g1450-434387-437046
ATGGAAAACAAGACGTCCAACGCCTCCACCTGGGGATTCGGAACGCGGGCCATCTTGGCC
GGGCAGGAGCCCGACCCCACCACGGGAGCGGTGGTGGTGCCCATCTCCCTGGCCACCACC
TTCCTTCAGGACTCGCCCGGCGTTCACAAGGGGTTCGAGTACTCGCGCACGGACAACCCC
ACCCGCCGCGCCTTTGAGGCCAACGTGGCGGCCTGCGAAAATGCCCGATACGGCATCGGC
TTTGCGTCGGGCAGCGCGGCCACCATGACGATCCTCCACATGCTCAAGGCCGGCGACCAC
GTGGCTGCCATGGATGACTGCTACGGAGGCACCTACCGCTACTTCACCAAGATCGCCACG
CCCATGGGCCTCAACTTCTCCTTCGCTGACTTCAGCCAGGAGGGCGAGGTGGAGAAGATC
CTCACCGACAAGACCAAGCTGATTTGGATGGAGTCGCCGACCAACCCGACCCTCAAGCTC
ACCGACATCCGCAAGGTGGCGGAGGTGGCCAAGAAGAGGAACATCCTGCTGGTCGTGGAC
AACACCTTCATGTCGCCCTACTTCCAGAACCCCCTCGACCTCGGCGCCGACATCGTCGTC
CACTCGGTGACCAAGTACCTGAATGGTCACTCGGACGTGGTGATGGGCATGGTGCTCACC
AACTCGGACGAGATCAAGACGCGCCTCCGCTTCCTCCAGAACGGCATCGGCGCCGTGCCC
AGCCCCTTCGACTGCTTCCTGGCCATGCGCGGCATGAAGACCCTCCACCTCCGCATGCGC
GAGCACGCCAAGAACGCCCTCCACATCGCACAGTTCCTCGAGTCCCACCCCAAGGTGGAG
AAGGTGATCTACCCCGGACTGCCCTCGCACCCGCAGCACGAGCTGGCCAAGGCGCAGATG
AAGGGCTTCGGCGGCATGGTCACCTTCTACGTCAAGGGCGAGTTGGAGCAGGCGAAGCAG
TTTTTGGAGAACCTCCACATCTTCCACCTGGCCGAGAGCCTCGGCTCCGTGGAGAGTCTG
GTCGATTTCCCGCCCATCATGACGCACGCGGCCGTGCCGAAGGAGGAGCGGGAGAAGCTG
GGCATCTCCGACACTCTGGTCCGGTTGTCGGTGGGTGTCGAGGACCTGGACGACCTCGTT
CGTGACCTCGAGAACGCCCTCGCCCACGTCCAGCTCTAG
>g1450-442897-443511
ATGAGTTCGGCCCAGTTGTTTGCGTACGGCAGCCCCGTCAGCACTTCGGCGTACCCGACG
CTCGCCGTCGTGCTCACCAGCGTGGGCCTGCTCTTCCTCACTTGGTTCTTCGTGTACGAG
CTGACCACAACGAACGAAACGGGCGACAAGAAGAGGAACCTGTTCAAGGAGCTGTTCATC
GCCTCGTTCGCCTCCCTGTTCCTGGGCTTCGGCACCCTCTTCCTGCTCCTGTGGAGCGGC
GTCTACGTGTAG
>g1450-455238-456109
ATGGCCGACATCACCGAGGAGCAGAGAATCGAGATCAAGGCTGCCTTCGATCTCTTCGAC
ACCGATGGCAACGGCAGCATCAGCGCTACCGAGCTCGCCTCCATCCTGAAGAAGATGGGC
ACCGAGGCTTCTGAGTCCGAGCTCAAGGACATGATCCACGAAATCGACGTGGATGGAGAC
GGTGAGATCCAATTCGAGGAGTTCCTTCTCTTGTTCTCGCGTCACAAGAAGAACCAGCTG
CCCGAGGATGAGGAGCTGAGGCAGGCCTTCAAGGTCTTCGACGCTGATGGCAATGGCACC
ATCTCCAAAGTCGAGCTCAAGCGCGTCATGGATATGCTGGGCGAGAAGCTCAACGACGCC
CAAATCGACGAAATGATGAAGGAAGCTGACACCAACGGCGACGGCGAGATCGACTTTGGC
GAGTTCAAGAAGATGATGGCCAGCAAGGGCCTCTAA
>g1450-460477-461402
ATGTCGTGGAGGGCGAGGCTTTCGACTAACATGCAGGAGCTCCGCTTCATCATGTGCACT
ACCTCACCCAACAGTGGAGGCGTGCGGTCTTTCCTGGACAGCAACTATGCTGAGTTGAAG
AAGCTCAACCCGCGGCTCCCCATTCTTATGCGAACTGGCGAACAAGCCCCGCCCAAGGTC
TACGCTCGCTACGACTGGGGCGAGGAGCGGGGGTTCGACCTGCACAACGCGACGGAAAAG
GAGGTCGAGGACCGAGTGCGTGAGCTCACCCTGATGGGCGAGAAGATGGAGAAGAGCTTC
GAGTCGATTCCCAAGGACAGGGATCTCATCGACGAGTCCGACGTCAGGAAGTTCGACACC
TATTAA
>g1525-75307-76608
ATGTCGTACGCCCCCGCTAAGCCCGTCTCCGTCCCCGTCGTGGTCGTCCCCACCGTCGTG
AGGCCTGCCGCCCCGCTCTACTCGAACCTCGGCAAGGGCGCCAAGGATCTGCTCAGCAAG
GGCTTCCCCTCCACCTACAAGGTCGAGGTGACCACCAGCGCTGAGAACGGCGTCCAGTTC
GTCTCCTCCGCCGAGAAGAAGCAGGCCAACAAGACCGATGTCGTCGTGGGCACCTTCCAG
CCCAAGTACAAGCTCGCCTCGCGCGGCCTTGAGCTCACCGGCACCTTCGATACCGACAAC
CAGATCAAGGCCGAGCTCGCCCTCGATAACCTCTTCGTGCCCGGCGTGAAGGGTACCTTC
AAGGCCCAGACCGGCGCCAGCCACGATCTCGAGGCTGCCTTCGAGTACAAGCACGAGGTC
GGTACCTTCACCTCGACCTTCGTCCACAACCCCACCACCGCCAAGACCCTCCTCTCGGCC
ACCGCCACCGTCTCGCGCCAGTCGGTCACCGCTGGTGTGGAGAGCAAGTACAGCCTCGCC
CCCGCCCAGCCCGGCACCCTCACCACCGTCACTGGTGCCCTCAACTACAAGGCCGCTACC
CACGATCTTACCGCCTTCGTTAAGAGCGATGCCGGCAGCGTGACCGATGGCGGCGATGCC
CCCAGGCTCTACTCGCTCGGTGCTCACCTGCACTACACCCCCTCGAAGGAGAGCAGCTTC
GCCTCCTCGCTCGACTACGACCTCCAGAAGAACACCATCAAGGTGACCATCGGTGGCTCG
CAGAAGCTCGATGCCCGCACTGAGACCAAGGCCAAGTTCTCCTCTGATGGTCGTCTCGGC
CTCGGTGTCGCCCAGCAGCTCACCCCCGCCGTCAAGGCCACCCTCGGCACTGAGCTCAAC
ACCTTCGACCTTGCTGGTTCCACCCCCAAGTTCGGCGTCCACTTCGAGGTCAGGGCTTAA
>g171-97507-98518
ATGACGAGCCCCAGCGGAACCCCTGCCATCAGCATCAACCGTGTGCCAGACAACTTCAAT
TTTGCTGATCTCTCGACCACCCCTGGCGGCACCATCTACGCCACCACCCCTGGAGGCACG
CGCATTGTGTATGACAGAAGCGCCCTCCTCTTTCTGAGGAACTCTCCTCTGGCAAAGACC
CCGCCCAACAACATCGCCTTCATCCCTGGCGTCACTGGTCCTGAGGTCAGCCCCAAGGAC
AAGACGAAGCAGGAGAAGGTGAAACAGCCTAAGGAGAACAAGGACGACGCCGACGAAATG
TTCAAGATGGACTAA
>g171-190975-191890
ATGAAGACCACACTCGCCTTCCTGGCTTCGATCGTCGTGCTGCTGTGCGCCTTTAACGTC
CTTAAGACCGAGGCCGCCATCGATGGCGCCACCGCCACCTTCACTAACCAGACCTTCAGC
GAGAACGGCACCAACCTCATCGGCACCTTCGAATTCACCAAGGACTTTGCCGCCGATTCC
CTCCAGTGCAACTTCACCTTCCCCGGCGACAAGACCTTCTTCAAGGTCATGGACCGCTAC
CTCATCAGCTACAACCAGCAGCGCCCCAACTTCACCCTCAAGAGCGACATCAGCTCCAAG
ACGCTCAGCACGGGTTTGGTGCACATTTACAACAATGCCACCATCACCAACGGCACGTCG
ATCGTCTACTACCTCTACCCGTACGACCAGCAGAAGAACTTCGGCAGCAAGTTCGACCTC
GGCGTCAGCTGCCAGCTCGATGGTGATGTGCTCGTCGAGCAGACCCTCGCCAGCGTGGTG
ATCAACAACCCCAAGGGCGGCAGCTCGAGCGCGGCCACCATCACCAGCTTCTTCCTGCCC
CTCCTCACCACCTTCCTCCTCTACTAA
>g171-333410-334087
ATGGTGGAACAGTTGAGCGAGGAGCAGATTGCTGAGTTCAAGGAGGCCTTCTCCCTCTTC
GACCGCGATGGCGACGGCAAGATCACGTCCAAGGAGCTGGGCACCGTCATGAGGTCCCTC
GGCGCCAACCCTACAGAGGCTGAGCTGAAGGAGATGATCAAGGAGATCGATACCGACAAC
AACGGCACCATCGACTTCCCCGAGTTCCTCACCATGATGGCCAGGAAGCTGCAGGATTCG
GAGGGTGAGGAGGAGATCAGGGAGGCCTTCAAGGTCTTCGACAAGGACGGCACGGGTTTC
ATCTCGGCCGCCGAGCTCAGGCACGTCATGACCAACCTCGGCGAGAAGCTCACCGATGAG
GAGGTCGACGAGATGATCCGTGAGGCCGACGTCGATGGCGACGGTCAGGTCAACTACGAC
GAGTTCGTCAAGATGATGCTCGCCAAGTAG
>g171-373267-373977
ATGGGTAAGGGTACGGCATCTTTCGGTAAGAGGCACACCAAGTCGCACACTCTGTGCCGT
CGCTGTGGCAGGAGGTCCTACCACATCCAGAAGGCCCGCTGCGCTTCGTGCGGTTACCCC
GCCGCCAGGCTCAGGAAGTACAACTGGTCGAACAAGGCCCTCAGGAGGAGGACGACTGGT
TCTGGCCGCATGCGCCACCTTAAGGACATGCCCAGGAGGTTCAAGAATGGTTTCCGTGAG
GGTACTCAGGCCAAGAAGGTCAAGTCCGCCCCCGCTGCCCAGTAA
>g171-399845-400287
ATGAACTTCCTCCGCAGGACCCCTCTTTCTGGCGTGCGCTCGTTCGCTCTCCGCGGCCAC
GGCCCCAGCCACGGACCCGATGGCTTCCAGCTCGGCCAGGCTGTCGGCACCACGCTCCCC
TTCTCGACGGCCCAGACCGCCAAGCCCTTCAGGGTGGGTCTCAGCACTTGGGGCTACTTC
GTCGCCGGCATCGGTCTCCCCACCTGGGCCGTGTGGTACTCCGCCAACAAGGAGGCCAAG
TAA
>g171-482667-483411
ATGCCCTCGCGCTGGGTAAAATCCCGGAACTATAAATCGGGCCGAAAGACAAAATGCCTT
GTTGTCGACCTCTTTTGTTTCCTTCTTCCTTTCTCTCTACCGTTCATCGTCATCCCTGAG
GTGTACTTTGATCAACAAGTTCGCCATGGTATCCGAGCCACAAGTCCTTCAAGATCAAGC
AAAAGCTGGCCAAGAAGCAGAGGCAGAACAGGCCCATCCCCTACTGGATCCGCCTGCGTA
CCGACAACACCATCAGGTACAACTCCAAGAGGAGGCACTGGAGGAGGACGAAGCTTGGCT
GGTAATCCAAGCAAGCTATTTCTGGTTTCGCTCTTTGCTCTTGTTACAGAAATAATTATC
CCCAGATCCTTCTGGAACTTCTCCTCACAATCTCTACCGTTACCTCTTTTCATCCCTTGT
CACAATCGTGTGCCCCCTCACTCGCATGTAAGGTGGTCGTTATTGGTGGTCGTTGGGATT
TACTTTTTACGATCTGTATGTGGAGTGAAGAACGTCAGCAGCAAAAGATGA
>g171-561704-562628
ATGGCACAGAGGAGACAAGAGCTGGAGAAGGAATTGGTTGGGCTTGCGCAGAAGGGTGCC
TTCGCCCTCATCGCACAGAATCTTGAGGAATACGAGTACTTCTGTGCTGAGAATCCCAAA
GATGCACTGCCTCTCTACGGTGCCCAGCTGCTGTCGTACCTGATCGAAAATGAGATTCAC
TACGCTCGCTTCCTGTGGCGGAGGATCCCCAAGAAGATCAAGGAAACCGATCCCGAATTG
AACGCCATCTGGACGATTGGCCAGAACGTGTGGAACAAGAAGTACACCGACATCTACCAG
AGCGCACAGGCCTACAACTGGAGCCCAGGCACCGTGCTTTTCGTGCAGGCCTTCGTGGAG
AAGTTCAGGGAGCGCACCTTCAAGCTCATCTCGAGCGCCTACTCCAACATCTCGACCGGC
GATCTTGCCGTGCTTCTCGGCCTCAGCGAGCAGGACGCCATTACGCTGGCGGTGCAGCAC
GGCTGGACGCACGATGCTGCTTCGGGATCGGTGGCTCCCAGGCCCATCGTCCCCGAGACG
AGGCAGGGCGCTTCGCTCGACCAGCTGCAGCAGCTCACCGACTACGTGTGCTTCCTCGAG
GAGAACTAA
>g171-610434-611798
ATGTTGGAGTACAAGATTGTCGTTCTGGGAAGCGGTGGCGTTGGTAAGTCGGCCTTGACC
GTTCAGTTCGTCCAGGGCATCTTCGTCGAGAAGTACGATCCCACCATCGAAGATTCCTAC
CGTAAGCAGGTGGAAGTCGATGGACAGCAGTGCATGCTCGAGATCCTCGATACTGCAGGA
ACTGAGCAGTTCACCGCTATGAGGGATTTGTACATGAAGAACGGCCAGGGCTTCGTGTTG
GTGTACTCCATCATCGCCCCCTCGACCTTCAACGATTTGCCCGACCTCAGGGAACAGATT
CTGCGCGTGAAGGACATGGACGACGTACCGATGGTGCTCGTCGGCAACAAGTGCGATCTG
ACGGACCAGCGCGCCATCTCGACCGAGCAGGGCGACTCGCTGGCCAAGAAGTTCAACTGC
GTCTTCCTCGAATCGTCGGCCAAGACGAAGATCAACGTCGAGCAGATCTTCTTCGACCTC
ATCCGGCAGATCAACCGCAAGACCGGTTCTGGCGGCAAGGACGCCGGCGGCGGTGGCGGC
AAGAAGAAGGGCTGCCTCCTCTTCTAA
>g171-633784-635368
ATGTCGCAGCAGCAGCAGACCGCCATCCCCGCCCCCGTGGTTGCGCACCACGCCCCCGTC
TTCGATCTGATGTACTACGTCAAGGCTGCTGTCGCCGGCGGTCTCTGCTGCTCCGTCACC
CACGGCGCCGTCTGCCCCATTGACGTCGTCAAGACCCGCATGCAGCTGGACCCCCAGAAG
TACAACAAGGGCATGATCTCGGCCTTCCGCCAGGTCGTCTCGACCGAGGGCGCCGGCGCC
TTGGCTACCGGTCTGGGTGCCACTGCGGCTGGATACTTTGTCCAGGGTTGGTTCAAGTTC
GGCGGTGTGGAGTTCTTCAAGGTGAACATCGCTCACGCCGTTGGCGAGCGCGCGGCCTGG
GAGAACCGCACCGGCATCTACCTGGCCTCGTCGGCCATGGCCGAGTTCATCGCTGATCTC
TTCCTCTGCCCGCTGGAGGCCATCCGCATCCGCTCCGTGTCGGACTCGACCTTCCCCAAG
GGTCTCGGCGCTGGCGCTGCGCGCATGTTCTCGACCGATGGTCTCCTCGGCTTCTACGCC
GGTCTGGGCCCCATCCTCTTCAAGCAGATCCCCTACACGATGGCCAAGTTCGCCGTCCAG
GGCAAGGCTGCTGAGCTCATCTACAAGAGCGCCAACACCTCGCCCGACAAGGCGACCAAG
GGCACCAACCTGTCCATCTCGCTCCTCTCCGGTGTGATTGCTGGCGTCGTCGCCGCTATC
GTCTCCCACCCGGCCGACACGCTCCTGTCCAAGATCAACAAGAAGGGCGCCGGCGGTTCT
GGCAGCACGACCTCGCGTCTCTTCACCATCGCGCGCGAGATGGGCTTCGCCAAGCTCTGC
CTCACTGGTCTCCCCGCTCGTTGCATCATGATCGGCACGCTCACCGCCGGTCAGTTCGGT
ATCTTCGACTCCGTGATGGCTGCCATTGGTGCCCAGAAGTTCCACTTCCACAACCCCGAC
GAGAAGCACTAA
>g173-5624-7267
ATGAAGAAGCACCAGCTCAGGAAGAAGGTGTTGAAGGGGGTGCTGGCTCAGGCCGCGGAC
AAGGCGGACGACGGCGTCAACCCCAAGTGGCGCGTGCGTCTCATGACCGAGGCCGACGTT
GATGAGGTACTGCGCATCCAGCGTCTCTGCTACGAGCCCGAGTACCGCGAGCTGCCCGAG
TCCTACACGGGCCGCATCCGCCTCTACCCGCAGGGCAACGTCATCGTCGAGGTGCCCGTC
TCGGCCGCCACCGGCGACAACGACGACTCGAGCGACTGCGACTACGCCGGCGACAGCAGC
GACGACTCCAGCGACAGCGAGAACGACAATGACAACGACGTCAGCAACGACTGCGATCAC
GACGACACTGACGACGACGACAGCGACAACGATCAGGCCGTGCCGATGATCAAGAACCAG
GTGACGAGGTACTCGCCGTCGGGTTCGCCCAGGTGCGAGGAGGACGTCCACTCGGTCAAG
AAGCGTCGCGTGGTGGGCAAGCCCGCCGCCGTCACCACCACCAAGTGGCGTATGGCCGGC
TACATCCAGGCCCAGCCGTACCTGCGCGAGGGCATCAACGACGTCAACGATCTCACCGAC
CTCGAGCGTTGGCTGGCCGACCACCCGACCATGGAGCTCGACCCGCAGCGCGACGTCATC
TACGTGCACGAGATCGCGATGGACCCGGCCTTTAGGGGCCAGGGCCTCACGACGCCGCTC
ACCGACTATGTGCAACAGCTGACCGCCGACGAGGGCTTCCCGATGATCACCCTCGTGAGC
CTCGGCCCGGCCCTCGGCTTCTGGAAGCGCAACGGCTTCATCATGTGCCGCGAGCTCGAC
TATGGCGGCCACATCTGCTACTACATGGAGAAGCCCCTCGCTCCCCAGCCGCTCGCCGAC
AGGTGA
>g173-12638-15278
ATGTTGAACGCCCCCCTTCCCCACTCGGCCAAGGCCCAGTACAAGACCATGGCCCCGGGT
CAGAGCATCAAGCGCTCGCTCAAGCACTCCCTGCAGCAGAAGGAGGAGGTGCCCTTCAAC
CCTGTGAACGTGCGTTCGCAGATCATCTGCACCGTCGGTCCGGCCACCAACAACGTGGAG
ATCCTCTCCGAGCTGCTCAAGAACGGCATGAGCGTCGCCAGGCTCAACTTCTCGCACGGC
AGCTACGAGTACCACGCCAGCGTCATCCAGAACGTGCGCGCCGCGTCCAAGGCCACTGGC
CACACTTGTGCCATCATGCTCGACACCAAGGGACCCGAGATCAGGACCGGCAAGTACAGG
GACGGCAGGAAGGAGGTCAAGTTTAACGTGGGTGACTCCTACACGTGGGTCCCTGAGGAG
GGCTTCCTCGGTGACGACAAGTTTGGTGCCCTCTCGTGGCTCAACATCGCCAAGCACGTT
TCGCCCGGTGACCGTATCCTCGTCGGCGACGGTCTGCTCGCCTTCGTGGTGCTCCAGGTG
TTGGACAACGGCTGGATCGAATCTACGGCTGAGAACTCTGGTACCATGGGTGAGAACAAG
AACGTCAACTTGCCCGGCGTGATCGTCGATTTGCCCGCCGTGACCGAGAAGGACATCAAG
GACATCGAGTTCGGCGTCCAGCAGGAGGTGGACTTCATCGCCGCTTCGTTCATCCGTAAG
GCCGAGGACGTGAGGGACATCAGGGCTCTCCCCGGCATCAAGGAGGCCAAGATCCTCATT
ATCTCCAAGATCGAGAGCCAGGAGGGTCTTGACAACTTTGATGAGATCGTCGAGGAGTCG
GACGGTGTCATGGTTGCCCGCGGTGATTTGGGTGTCCAGATCCCCATCAAGAAGGTCGCC
ACCGCTCAGAAGATGATGATCACCAAGTGCAACTCCGTTGGCAAGCCCGTCATCACCGCT
ACGCAGATGCTCGAGTCGATGATCCAGAACCCGCGTCCCACTCGTGCCGAGGCCACCGAC
GTTGCCAACGCCATCTTCGACGGTTCCGATTGCGTCATGTTGTCGGGCGAGACTGCTGCA
GGCAAGTACCCCGTGGAGGCTGTTGAGATGATGGCCCAGATCTGCTATCAGGCCGAGTCC
GACATCGACTATAGGGCTCTCTACAGGAAGATTCGTGAGTTGGTCATCGCTCCCCCGATC
TCCGTTCCCGACACCATTGCCTCGTCCTCGGTCAAGTCGTCGTGGGATATCGCCGCCTCC
GCCATCATCTGTCTCACTGAGACTGGTAACACCGCCAGGCTCGTGTCCAAGTACAGGCCG
TCGTGTCCCATCCTCTGCGTCACTCCCAACGCTTATGTGAGCCGCCAGATTCAGATCTCG
CGAGGCTGTATCCCTTACGTCGTGGAGTCGATGAAGGGTACGGATAAGGTCATCGAGAGT
GCCATCCGGCATGCCAAGGATGAGCTCAAGATCGTCAAGGCTGGTGACTTCGTGGTCATC
ACGTCGGGCTTCCTCGAGGGCACGTCGGGTGCCACCAACATGTTCCAGGTCCGTCAGGTC
CCCGAGTAA
>g185-3386-4880
ATGGAGCGGCCGGGCGATGACGAGGCGTCGGGATACGTGATCAAGGCGCTCATGGGCAAT
GCCAACCCCAAGCTGGGCAAGGACATCTCGGAGGCCCTCGGCGTGCGATTGTGCGACTGC
GAGGTGGCCAAGTTTGCCGATGGCGAGATCAACATGCAGATCAAGGAAAACATTCGTGGT
GCCGACGTCTTCGTCATCCAGGGCACCTCGCCTCCCGTCAATGACAACCTCATGGAGTTG
CTCCTGCTCCTCCAGACCATCAAGCTCTCCTCGGCCAAGCGAATTACCGCCGTCATCCCC
TACTACGGCTACGCTCGCCAGGACCGCAAGACTCGTCCCCGCGTCCCCATCTCGGCCAGT
GCCGTGGCACAGCTCATCGAGACCATGGGAGCGGATCGAGTGGTGACGGTGGACCTGCAC
TGCGGGCAGATCCAGGGCTTCTTCCACCACACGCCGGTGGACAACCTGTTCGCCGAGGGC
GAGTTCATCGATACGCTGCGGCTCAAGTACCCGGAGCCCGCCGACGTGGTCATCGTGTCG
CCCGATGCCGGCGGCGTGCTCCGGGCGCGGCGAGTGGCCGACAAGCTGCACGCCGAGAAC
GTGGCCACCATCCTCAAGCGTCGCGCGCAGGCCAACGTGATCGAGGCCATGCAGATCGTG
GGCTCCGTCGACGGCAAGAAGTGCGTCATCATCGACGACATCATCGACACCGCCGGCACC
CTGTGCAAGGCCGCCGAGCTGCTCAAGGAGAACGGCGCCACGGACGTCATCGCCTGCGCC
ACCCACGGCGTCTTCTCCGGGGGCGCCGCGATGGACCGCATCAACGCGTCGTGCCTCACC
GCCGTCTACGTCACCGACTCCATCCCGCAGGAGCACAACAAGGCACGCTGCGACAAGCTC
CACGTCCTCTCCATCGCGCCCCTCCTCGCCCGCGCCATCCGCCGCATCCACGAGGAAAAG
TCCCTCTCCGTCCTCTTCGGCAACACCCTCTGA
>g185-13127-14308
ATGCCCAAGGAGACCAAGCAGAGACGCGGAAAGCAGGTGGCGGCTGCCCCCTACGACAAG
AACGCCAAGGCCCCCGTCAAGGGTGGCAAGAAGGCTGTGCCCGCCCAGCCCGCCAAGAAG
GCCAACAAGGGTCCTTCCCCCGCTCTGTTCGAGAAGAGGCCCAAGAACTTCGGCATCGGT
CAGGACATCCAGCCCAAGCGTGATCTCACCCGCTTCGTCAGATGGCCCAAGTACATCCGC
ATGCAGAGGCAAAAGCGTGTGCTTCTCCACCGCTTGAAGGTCCCGCCCACGATCAACCAG
TTCACCAGGACCCTTGACAAGAACCTTGCCAAGAACCTCTTTGCCTTCGTGGACAAGTAC
AGGCCCGAGACCAAGGCCGAGAAGGCTGACCGTCTTAAGAAGAGGGCTGCCGAGATCGCC
GCTCTCAAGAAGGGTGAGCAGCCCCCCGCCGTTGCCACGCCCTACTTCGTGAAGTACGGC
CTGAACCACGTCACGTCGCTCGTTGAGAGCAAGAAGGCTAAGCTCGTCGTGATCGCTCAC
GATGTTGACCCCATCGAGCTCGTCGTGTGGCTGCCCTCGCTTTGCAAGAAGGTCGGCGTG
CCCTACTGCATTGTGAAGAGCAAGTCCAGGCTCGGCCAGGTGGTCCACAAGAAGACCTCC
GCCGTGCTCGCCATCACCAACGTGAGGAAGGAGGATCAGCCTGCCCTCGCCACCCTGACG
AAGGCCATCCAGGAGAACTACAACGACAGGTATGACGACCTCAGGAGGCAGTGGGGCGGT
CTTCAGCTCGGCCGCAAGTCCGTGCACAAGCAGAAGGCCAAGGCCAAGGCTGCTGCTGCC
AACCAGTAA
>g185-14416-15859
ATGAGAAGATTTTTTGGCGGTTCGGCTAAGCCCCAGCAGCCCACCCCCACTCTTGCCGAA
GCCAGTGCCTCGGTTGATAAGCGATGCGAGGCGCTCGATGAAAGGATAAAGAAGCTCGAC
GCCGAGTTGATTCGGTACAGACAGCAGATGCAGCGAACGCGCCCTGGTCCTGCACAAAAT
GGCATCAAACAGCGTGCGCTCAAGGTCATGAAGCAAAAGAAAATGTACGAGCAGCAGCGG
GACCAGCTCATGGGCCAGTCTTTCAACATGGAGCAAGCCCAAATGATGACTCAGACCCTC
CAGGACACCGTCACCATTGTGCAAACGATGAAGGACGCCAAGGTGGCCATGCAGAAGCAA
TTCAAGGACGTCAAAATCGGCGACATCGAGAATCTCTGGGACGACATGGAGGACCTCTAC
GAGACCTCAAACGAGGTGCAGGACATCCTCTCCCGCTCTTACGGAGTGCCCGAAGAGTTC
GACGAGGCCGACCTCGAAGAAGAGCTGGCGGCGTTGGGAGAGGAGGACTGGGGCCAGGAG
GAGGCTTCTCCGTCCTACCTGCAGGCCGTGACGTCGCCCATGGGCGGCGAGTCCCCCCTG
CCCGCACCACCCGTGGGGCAGGGGCCGGGCGCCTCCGATCCCTACGGGCTCCCCTCCGTA
CCGCAGGGCGAGCTCGCCAACTGGTAG
>g185-18353-20458
ATGGAGCAGCCCCCATCATCCCAAGGCCAGCCCGCCAAGGGGGGCAACTACTCGGACCTG
CAGAAATGGAAGGAGAACGTACTGGAAGGCCGACCGCGCATCGATAAGTCGCTCCTCACC
GAAGACGTGACAACAACGAAGGGTAACGAATTTGAAGATTACTACCTCAAGCGAGAGCTC
CTTATGGGCATTTACGAGAAGGGATTTGAGAACCCCTCGCCCATCCAAGAAGAAGCCATC
CCCATCGCTTTGGCAGGACGAGACATCCTGGCACGCGCGAAGAATGGAACCGGCAAGACG
GCGTCCTTTCTCATCCCCGCACTCGAGCGCATCAACACTGAACAAAACATCATCCAAGCC
CTCATCCTCGTGCCCACGAGAGAGTTGGCCCTGCAGACTTCGCAGGTTTGCAAGGAACTC
GGCAAGCACATGAACGTGAAGGTCATGGTCACCACTGGCGGTACCAGCCTCAAGGAGGAC
ATCATGAGGCTCCACAGCGTGGTCCACATCCTGGTCGCCACTCCCGGCCGAGTGCTCGAT
CTCGCCAAGAAGAAGGTCGCCGACCTCAGCAAGTGCACGTTCATGGTCATGGACGAGGCC
GACAAGCTTCTGTCGCCTGAGTTCCAGCCTCTCGTGGAGCAAATCATCGAATTCACGCCG
GACGAGAGGCAAATCCTCCTCTTCTCGGCCACTTTCCCCATCACCGTCAAGGATTTCAAG
GAACGATTCCTGCGGAAGCCTTACGAGATCAATCTCATGGAAGAACTCACCCTCAAGGGT
GTCACCCAGTACTACGCCTTCGTCGAGGAGAGGCAGAAAGTGCACTGCCTGAACACTCTC
TTCTCCAAGCTTCAAATCAACCAGTCGATCATCTTCTGCAACTCTGTCAACCGAGTGGAG
CTGCTGGCGAAGAAGATCACGCAGCTGGGCTACTCGTGCTTCTTCATCCACGCCAAGATG
CTGCAGTCCGACCGTAATCGCGTCTTCCACGAGTTCAGGAAGGGTGTGTGTAGAAATCTG
GTGTGCACAGATCTGTTCACGCGAGGAATTGACATCCAGGCCGTGAACGTAGTCATCAAT
TTTGACTTCCCCAAGAACTCGGAGACCTACCTTCACCGCATCGGCCGCTCGGGCAGATTC
GGACACCTGGGCCTGGCGATCAACCTCGTGACCTACGAGGACAGGTTTAACTTATTCAAG
ATAGAGCAGGAGCTGGGTACTGAGATCAAACCCATCCCTCCCGTGATCGACAAGAGACTG
TACGTGGCCTACTAG
>g185-111308-112608
ATGGAACGTGTTTCGGTGCGTCTCGTGGCGACGCTCGCGGTGCTGGCCCTGATGATCCTC
GCCGCGTCGGGCCAAAGCGACTGCAAGGGCAAGGTGGGCAAGTACGAATACGACCTCACC
CCGCTGGCACAGAAGCTTGGCGCTGTGGACCTCCAGACGCAGGATGCCGGCAACCCGCCC
CAGACCTACTACTACCGCGTGTGCGGCGTGGTGAGCAACAACTTCTGCCAGACGGTGGAC
GACATGACGCCCGCCGTGTGCCAGAAGGACACGCGCATCCCCGCCGAGTTCCACGATTGC
GGCAACCAGAAGACGGCTCGCTTCCAGAAGCTCCCGAGCGGATCCGATTCTGATGGTTTC
ACCCTCTCCTACACGGGCGGCCAGGATGGTCGTGCATCCATGATTTACTTCAAGTGCGAC
AAGTCCAAGGATCCTGGCGTGTTCAGCTTCGTCAAGGAGGACCCCGTCAAGACGTATGAT
TTGCAGTACCTCAGCAAGTGGGCTTGCCCCACCAACGGTGGCGGCGGCGGCGGCGGCGGT
GGCGGTGGCGGCGGCGGTGGTGGCGATGACGACGACGGCGGCATTTCCGGCGGCTGGATC
TTCATCATCATTTTGTCGAGCTTGCTGGTCCTGTACCTCGTCGGTGGTGTGGCCTTCAAC
AAGTTCTACAGGCACCACGAGGGCAAGGAGATCATCCCCAACGTCGAGTTCTGGATGGCT
CTCCCCGGTCTCGTCAAGGACGGCCACCTCTTCGTCTGGCGCAAGGCTCGCAGCCTCACT
GGCCGCGGCAGCTACGAGGAGATGTAA
>g185-220327-220978
ATGCTGATCAAAGTGAAGAACTTGACGGGTAACATCATCGAAATCGACGTCGAGAAGACG
GACACTGTTCAGAAGCTCAAGGAGTACGTCGAGGAGAAGGCTGGTATCCCTCCCAAGCAG
CAGAGGCTTATTTTCGGTGGCAAAGCCATGGCTGACGAGAAGACGGTGGAGAGCTACAAG
ATCCAGGCCGGCGCTGTGCTCCACTTGGTGCTTGCGCTCCGTGGCGGCAACTAG
>g185-339279-340918
ATGGCCGATCAATCTTCGTACAAAAAGGGGGATAAGATCCTTTACATTACGCCGGCCCCG
GCCGTGCCACCGGAAGTTTCACAGCTGCAGGAAATCGTCGGCAGCGATGGCGCTGTCAAG
GTCATCTCTCGCCCCCAAATCACCGACGATAAAATTGGCGAGCCTTCGTCGACGTACGAC
ATGGTGATTTCGATCGGCGCCTCGGCTGCTGGCCACCAGACCGACTTCCTGTTCGAGCTG
GCACGCGTGCTGAAGCCCTCCGGTTCGCTCGTCCTGCGCGAGCCTCTGCTGCTCGGCTCC
AAGGAGGGCATCGTGACGGCCCTGCGCACGGAGAAGGAGGTGGAGAGCGCGCTGCTCATC
TCGGGCTTCACGTCGACTACCTTCCAGAACACCCAGGGCGCCTCGTCTGTGGCCTCCTTC
CCCGCGCTCAATGGCTCCGCCGACGTGCAGACCCAAATCGCCACCTACGAGATTCGCTCG
GTCAAGCCCAACTGGGAGTTCGGAGCCTCGGCCGCACTCTCGCTTCCCAAGTCCTCGAAG
CCCGTCTCCGCTGCCCCCGTCTGGTCCCTCTCCGCAGATGACACGATCGATGCGAGCATC
CCCGTGGCCAAGCCCGTTTCTGTCGGTTCGACCTGGAAGCTGGACGCCGACGACGGCGAT
CTGATCGAAGACGACGCCCTCCTCGAGAAGGAGGACTTGGCCAAGCCGGACAAGGTGTTT
GATTGCGGCACGAGCGCGAGCGGCAAGAAGAAGGCGTGCAAGGACTGCTCGTGCGGCCTT
GCCGAGGAGCTCGAGGCCGGGCAGGAGGTCAAGAAGAAGACCCCCGAACAGGCTTCGTCC
TGTGGCAGCTGCTATTTGGGCGACGCGTTCCGATGCGCGAGCTGCCCCTACCTCGGTCTG
CCGGCCTTCAAGCCTGGCGAGAAGATCTCCCTCGCCAACATGGAGTAG
>g185-360966-362837
ATGAAACCGGGATCGGATATTTTGTCGACACTTTGTCTGCTGCAAAAGGCCGACGTGCAG
ATTCCGGATCTTTGTCTCGCTCTCAAACCGGCCAGCAACACCATCAGCTCCTCTTCATCA
GAATCCAACATGTCCCTCAAGAACGACATCGTCTCCGCCCTCGAGAAGGTCACCGCTAAG
GATGCCACTGAGACCCACGCCTGGGTCAAGCCCTACGAGGCTCCCGCCGCTGATGTGCAC
CAGTTCCTCTTCTTCCTGAAGCCTGAGGCCACCGCCAGCTACGATGGCGTTAAGGTGGAG
GCTGTGGTCGAGCTCGCCCTGAAGACCCTCGCCGATTTCGGCGTTGAGGTCGGCTCCGTC
CGCGTGCTGTCCGGTGACTACCTCGACAAGCACAACCTGATGGGCCAGCACTACGGTGTC
ATCTCGGCCATCTCCAGGGAGGGCGTCTCCGTCATCTCCGAGCAGGCCAAGAAGAACCTC
GACGAGAAGTTCAAGGCTGACATTGAGGCCGGCGCTTCCGTCCTCGGTGGCCACCAGTTC
CTTGCCAAGGAGCCCGGTTTCAACGCCTTCTCGCTCAGCGTGCTGAACGACAACCTCGGC
ACCACCAGGCTCGCTGGCGGTACCTATGCCATGAAGATCAAGGTCCTCGGCAAGCCCGTC
ATTATCCTCAACCCCTTCCACGCCTACCAGCTCGTTCCCTACACCACCAAGGGTCACTCG
ATCATCCTGTTCGAGGCCCGCTCCACCAAGTCGTGGGAGGATCTCCGCCAGAAGCTCACC
GGCACCACCGACCCCAAGGATGCCGCTGAGGGTTCCATCCGCAACCTGTTCCTTAAGCAC
AAGGCCGACCTTGGCCTCGGTGACGTCGACAAGGGCACCAACGGCGTGCACGCCTCGGCT
GGTCCCCTCGAGGGCATGGTCGAGCTCCAGCGCTTCTTCTCCGACCACGAGGCCGGCTCC
ACCCTCGGCTACGATCACACCGCCTTCGGCAAGCTCCTCATCTCCAAGGGCCTCAGCCTC
GAGCAGGTCCAGAAGCTCGCCTCCAACCCCGACCTCGACCAGGAGGGCAAGAAGGTGTCT
GCTTTCGACGCCACCGAGGAGAAGGACGCCGCCGAATCTGCGGACGTTCTCGCCAACGCC
AAGTTCGCTGCCGCCCTCTAA
>g185-371587-375028
ATGACAACTCGCTCGGCTCTGCTCCTTCTTCTTGTTGTTGTAGGGCTCGCCCTCTTCATC
TCCTCCGGCGATGCGTCGGTGGACTTTGGGTACCCCAACAAGTGCTTCTTCACAGTGGGC
AAATCGGCCTCGGGCGCCTGGTGGTTCGTCGACCCGAGCGGCAACCCCTTCCTCTCGCTC
GGCGTCACCTCCGTGAGCTGGTGGGGCGACACCTCCACCACGGACGCAACACCGTATTAC
GATGCCGTCAGTGCCAAGTTCAACAACAACCAGTCGGCCTGGGCCGACTCGGTCGTGAGC
CGCATGGCCTCGTGGAAGCTCAACACCCTCGGGGCGTGGGCCGACACCGTGGTCATCACC
AAGGGCATGCCCTACACCCGCATCTTGGGCTTCGCCCAGGGCTTCGGCTCGTGGCTCGAG
GGCACCTTTCCCGACGTGTTCGACCCCGCCTGGGAGGCCGCCACCTACGACAAGGCCGCC
CAGCTCTGCACCCCGCTCCGCAACGACCCCAACCTCATCGGCTACTTCCTCGACAACGAG
GTGATGTGGGGTCCGATGCTGCAGGGGTGGGCCGACTGGCGCTATCCGGGCACCATTCTC
GACCAGATGCTGACCGTGTTCAACAAGACGGCGGCCGGCAATGTCAAGGCCGTCGCCTTC
CTCAAGAGCAAGTACCCCACCATTCAGGCCCTCAACACCGCCTGGAACACGACCTACGCC
AGTTTCGACCAGATCTCCACGCGTCCGCTGAAGTCGACCGCGGCGCACACCAACGACTCG
GCGGCCTTCACCTACCTCGCCGCCGTCCAGTTCTTCAGCGTCTCCCGCAACGCGATCAAA
AAGTACGATCCGAACCACCTGCTGCTGGGCGTCAAGTTCGCCGGCGCGCCGAGCGCTCCG
GTGCTGCAGGCCTGTGGGGAGTACAACGACGTGATCTCGCTCGACGTCTACCCGACCGCA
TCCGAGCCGGTGCCCAGCACCAGCTTCCTGAACCAGGTCTACGCCTACGGCAAGAAGCCG
CTGCTCATGGCCGAGTTCGCTTTCCGCGGCAATGACTCTGGTTTGCCCAACACGAAGGGT
GCGGGCCTTGTGGTGCCCACGCAGGCTGACAGGGCTCTGGGCTACAAGAACTACACCACG
ACGCTGGCCAAGCTGCCCTACGTCATCGGATTCCACTGGTTCGAGTGGTTCGACGAGCCG
GCCGAAGGTCGCAAGCTCGACGGCGAGAACAGCAACTATGGCCTAGTCAAGATCAACGAC
GACACGTACACTGTATTGACGAACCAGATGAAGACCACCAACCCGCAGCTCAACGGCCTG
CACGGCAGCTCCACCGGCCTGACCTCGGCCAAGTGTGTCAGTGCGCTGACGTGCTTGAAC
AAGTGTTCGGGCCAGGGCTGCTGCAACACCGACACCGGCGTATGCAAGTGCAACACGGGC
TTCAGCTCTGCTGACTGCTCTGTCGATAGCTCGAAACTGCAGGTGCCTTCTTTTAGCACA
GTCGACACCAACGTGTGGTCTATCAACAACGGAGGCGGTTGGCTCAGCCAGACCTTTAGC
ACGTCCCAGACGTCGACGTCGTCCAACCAGCTCAAGCTGGCGATCTCGCCTTGCACAGGT
TCGTGCACCGGCTTCAATTTCTCCGCCGGCGGAATCACCACCAAGTCCTCGCTTTACGGC
TACGGCATCTACACGGCCGAGATCAAGGCCCCTGTCTCTCCCGGCTTCACCGCCCAGTTG
GAGATCAACAGCAGGTCCACGCCCAGGGATAGCGTGACGCTCCACGTCAACGGCGGCCGT
TCGGTGGCCTCGCTCGACTACTTCCACAACGACCAGTACAAGTACCTCAAGTGGATCAAC
CCGCTGCCCTTCGCCAATGCGTCCACCCAGTACCACACCTACAGTATCCACTACATGCCC
ACCTACTTCGCCTTCTACGTCGATGGTATCCTCCTGGGCCAGTACAACAGCACGACCGCC
ACCGCCGGCCTGCCGAGCCGCTCCATGGCCGTGTGGCTCTACGTCACAGCCGACAACACG
TCGCCCCAGACCACCGACGCCATGTGGGTCAAGTCGGTGTCCTACAAGCTGGTGGGTGGA
TCCACCGCTACTTGTCCGGCATCGGCCAACGGCGGTGACCAGAGCCCGGCCGACCGGCCC
GTCTCTTCCATCGGTGCCTATCTGTCGTCCCTCTTCTCCTCCTTCGTCAAGTGGTTTATC
TGA
>g185-395816-398240
ATGAGGAGGATCAGCAGAGTTGTGGCGTCGTCCGCTCGCGGTACGGCCCTGAACAAGTCT
AGCCGCACTGCCGCCGTCTTCTCCCGCGGCTATGCTGCCCCCGAGACGCCCCAGACCGAT
TTCAAAGTGACCGATGCGGGCATTCGCGTCTATGCTCATGGCGTTGTGCACGACATGAAC
TTCCTGGGCGATCTTCCCGCCGATGCGCAGGAGGTCCTCTCCGGCAAGGAGTGGGTGCCC
CAGTCTGTTTCCGACTTCACCGCCAGGTGGGAGAAGGACGCCCCCGAGCTCGCTAATCAG
GCCCGCCAGCTTGAGTCTCTCATTACCAAGTTCGAGAAGGACCCCGTCCAGTTCGGCAAG
TCTGAGGCTTATCTGCGGGACATTGCCATTCACACTACGCTCGAGCGTGCTGCCGCTCGC
GACGTGAGCCCTGTTGAAATCGCCCTCACCCACTTCTGGACCGCCTACTCCGAGGTCCCC
TCTGGCGGCAAGCTGCGCGAGTCCGCCCCCACCCAGCTCGAGCGCGTCAAGGCCTTCCTC
CAGAGCAAGAAGGATCCCGAGCTCAGCGCCCAGAACTCGCGGGCGAGGAAGACTGCTGCC
ATCGTCAAGTTCGTCCAGGCCTCGAGCGCCGAGGACAAGGCTGCCGCTGCCGCTCTGCTC
CTCGGTTCGGAGAAGGGCCAGGCCCTCAAGGCCGCCCTCAAGGGCGTTAACGACGCCGCC
AAGAAGGAGCAGATCGTCAAGCAGCACCTTCAGTGGGATGAGGCCACCCAGGCCGCTGCC
GAGCTCACTCAGAGGCACCCCGAGTTCACCGAGCTCAAGGAGGTCCTCGCTCACTTCCCT
CCCCTCGTTCATCACATCGGCGAGCCTTCGGCCGTTCAGTACAGGTTCAAGCACGCCACC
GACAAGGCCTACAAGAAGTGGCAGCAGAACTACTCGCTCACCAGCTCCGATGAGGTCACC
GAGAAGGACCCCCTGCTCAAGAACCTCAGGGAGCTCCTCTACTCGGGCAAGCCCGCCAAC
ACCGTCGCCGACGCCAAGAAGGCCCAGGCCCTCCAGCGCAGCTTCCCGGGTCTGCTCTTC
TCGCGCGACGTCGAGTCCTTCTCCAACTTTGTGAGGAAGGACGAGGAGGTCAAGAGCCAG
CTGAGCACCATCTACGAGGACAGCCTGAAGGAGTACCCCAACGACCCGGCCCTCGCCGTC
CCCAAGGACCTCGACCAGAGGGTCAAGCTGTGGAGCAAGCACACCCAGGAGCTCGCCGAC
GTGCAGCAGCAGTGGCTCGACGCCAAGATCGCCTTCATCGACGCCAAGATCCAGGAGGGA
ATGGTCCAGATGGACAAGTTCGTGAACGGCACCGTGGATGACATCCTCGCGGACCACCCC
GAGTGGGAGAAGGAGATTGAGGACGACATCGCCAACCACCGCTGGGACCCCGAGATGGAG
AAGAAGGAGTACGACAACGCCCTCCACCACTACTACGAGCACCACACCGTCCACGCCTAA
>g185-447548-448469
ATGTCTACCAACATTTCTGAGTCGGGCTCTATCGATCGCGTCCCCTTCAAGCAGGAGAAC
TCCCTTGAGAAGAGGACTCGTCTTGCTGCGAAGATCAGGGCCGCCCACCCTGACCGAGTT
CCTGTGATCGTTGAGAAGCACGCCAGGAGCAAGCTCATTCCCGACATTTCCAAGAGGAAG
TTCTTGGCTCCTGGCGATATTGCCGTCACACAGCTGATCTCTGAGATTCGGAAGCACATC
GAACTCAGGCCCGACGAAGCCATCTTCCTCTTCGTGAACGGCCATTCGTTGCCCCAGTCT
GGCGCCCTCCTCTCGCAGGTCTACGAGAAGCACAAGGACGAAGATGGATTCCTGTACATC
GTCTACTCGGCCGACAGTATGATGGGCGCGTCGTGCTAA
>g185-488188-489949
ATGTCTCGTCGAGGTAAGAACAATGTGAAGCGCGCCATCGTTAGCGATGCGACTTCCATC
TTCTCTGGGACGACTGCGCCGGCCAAGCCGACTCCCATCAACAACTTCAATCCTCTCTTC
CACTACCTCCAGACGATGTTTCCCAACTACGACAACGCTGTGCTCAACTCTGTTCTCGAG
CAAGAGGGCAACTCGATCGAGAGGGCCATCGAGGTGCTGCTGACCTTCGCCATACCTGAG
GAGCCCAAGACCTCCAAGGATGCCGCGGCTGGCGACGAGATCTCCTTCATCGATGACTTC
AGCGATGACGATGCCGAGGAGACACCCTCGAACGTGTTCGCCGTGCCCGTGATGCCGCCC
GTCTCGTCCCACAACAGCGGCCGCGACCACGTCTCCCTCTACGAGTATCGCAAGAAGAGG
AAGGCCACCAAGTCCCAGCGCGCGAGGAGGAGGCAGCGCGCCCACCAGGTCCAAGCCGCC
GCCGTTGCTGCCGCCGCGGCCGCGCAGAACAACAAGAAGAAGGTCAAGTGGGTGCCGCTT
CAAGAGTTCATCGCCGAGACCGCCAACCCGTTCGACGCCCTCGCGCGCGACGAGGAGCTG
CTCACTGCTGCAGCTGAAAGCTTGGTCCTGGGCAGTGAGAAGGAGACCGAAGAGAAGCTC
CCCGCCACCGTCGCCGATCCTGAGGAGGATGAGACCCGGTGCCTCCTCAGCGCGGAGGAG
ATCAAGGAGGGCTGGGACGAGTTCGAGTCGGCTCAGGTGCTCGACGACGAACCGCAGGCG
CCTGTGGCCGTCGACTGCAGCTTCGACGACTTCGATGACTTCAGCGATTTCAGCAGCAGC
CCCAACGCCGACGAGTGGCTCCACTACACCCCTCCTACCCCGCAGCCGCAGGAAGAGGAG
GAGACCACCTCGAGCTCTGCTTCGTCGTCGTGTTCTTCGTCGGAGGATGAGGACTTCGTG
GTGATCCAGGAGTCCCTGCCGAGCCTCCAGCTGGAGCCCCTCTCCACGCAGCCCATCACT
GTCGCCCCGGTGGTGGTTACTCCGGCTGCTGCCGCCGCTGTCGTTAGCGAGGCGGAGGAG
AAGAGCGAGCAGGTCGTGGTGGTGAAGCTCTACTACTCGTCGAGCGACATCCACAGGCTC
GGCCTGGCGAGGAGCACCTTCACCTTTGCCAAGCTGTTCGACTACGCCGGCGCCTACCTC
CAGAGCAAGTGCCAGCAGCTGCTGGCCAAGTCGGCCCTTCCGCGCGCCTTTGCGCTGACC
TACGTCGACGACGAGGGCGACGTCATCCACCTCTGCTCCGAGGAGGAGCTGGCCGAGGCG
CTCAGGCTCCACGAGCAGGTCTTCTGGACCCCCGGCCAGCAGCCCGTCCTCCGCGTCCAC
ATCAAGTCGCTCGAGATGGCCGCCAACGATGGTCACATCGTCGTCCGCGCCTAA
>g185-491198-492019
ATGCGCAGTTCTTCTCTTGTCGTTGCCCTTGTGGTGGCCGTTGTGCTCCTGGCCGAGGCC
TCGCTGGCTTCGCGGGTCATCCTCCGCAACAACTGCGGCGGCGACATTCAGGCCGTCTGG
ACCGGCAATGGCCAGGCTCCTCGGGTCGTGTGCAACCTTGGTCGCGGCCAGGGCTGCCAG
GTGAGCGAGAGCGGTGGCGGCAACTTCAAGTCCGGCTGGGGCGGTAGGACGCTGGCCGAG
TTCGACTTCAACAACGGCGGCAAGGACTGGTACGACATCTCGGTGATCGTGGGCTACGAC
GTGGCCATGTCCATCCAGGCCCCCTTCGGCGGCTACTCCCCCTCGTGCTACAACGGCGGC
TGCCCTGGTACGGTTCATCACTCATTCATTGCCTCTTGGTAG
>g185-542178-544089
ATGAAGTCCTTCATAGGTGGATTTCTGCTGTTCGCCTTCTTGGGCCTCTTCATCTCCGTT
CATTCGGCCGAGGTTTACCGAGGATGGGAACTGAAGCAGGTGGATCGCCAGATCTCGCTC
CAGACCCAGAACGTGCGCCATCAGATCCGCATCGTGGCCGTGAATGCGGGCGACAAAGCC
GCCTCCACCTTCCTCCTCGCCATCCCCTCTGAATCGGCAAGCCACTTGGCCCTTGTGAAT
GCCGTTCACGACGGCAAGCTTGCCGATGTTGCGCCCACTACTGGCGAGGCCCGCGATGGA
GTGACCTACTTCAGCGTTGCGCTCAACCAGGCCGTCTCCCCCAAGGAGCAGGCTGTCATC
GACCTGATCTACGTCTTCACCCACGTCCAGCAGCCCTACCCGACCCACGTCAGCCAGAAC
GATCCCCAATTGGTCGTGTACGCCGACAATCACTACTTCCTCTCGCCCTACACCGTCCGC
AGCCAGAAGACCGTCGTCAAGCTGCCCAGCGCCTCCCTGGAGAGCTACTCGCAGGACGCT
CGTCCGGCCGTCCACAAGGGCGACACCATCACCTATGGCCCCTATGAGGGCATCCAGGCC
TACCAGCAGAGCCCCCTCAAGATCCATTTCGAGAACAACACGCCTTTCTTGACCATCACC
AAATTGGTGCGAGAAGTGGAGGTTTCTCACTGGGGCAACGTCGCCATCACCGAACACATC
AATATGCAGCACGACGGCGCCAAGCTCCAGGGCTCGTTCTCGCGCTTCGACTACCAGCGC
ACCCAGAGGACTTCGGGCGCGCCCATCCGCTCCGTCCTCAATCTCATTCCCCTCAGCGCC
TCCGAGATCTACTACAGGGACGAAATCGGCAACATCTCCACCTCTGACATCACCGTCGCC
AAGGACAAGCTCGCCGTCGAGCTCACTCCCCGCTTCCCGCTCTTCGGTGGCTGGAAGACT
GAGTTCATCTTCGGCTACGACCTTCCGCTCTCCGACTTCGTCTTCACCGACGCGTCTGAC
TCGTCCAAGCTCGTGCTCAACACCACCTTCGGCAAGGAGATCGCCCTCGACGCTGTCATC
GATGAGCTCACCATTCGTGTCATCCTCCCCGAGGGTTCCAAGAATGCTCGCTTCGAGCTG
CCTTTCGCTGTTGATTCGGAGGAGGCCACTCTTCACCACACCTACCTCGATACCTCTGGC
AGGCCCGTGCTTGTTCTCCACAAGAAGAACGTTGTGGCCGAGCACAACCAGCACTTCTTG
GTCACCTACAACTTCAGCAAGACCAGCATGCTCCTCGAGCCGCTGATCCTCGTCATCTCC
TACTTCGTCGTGTTCCTCTTCGCCATGCTCTACGTCCGTTTCGACCTCTCGATCAGCAAG
GAAAAGGTCGTCGTCTCCAAGGAAGCCAAGCAGGCCAAGATGTGA
>g185-561577-563441
ATGCAGCAGGACGACGACATCCAGGAGGAGGAGCTTAACGCCAGCACCCCCGAGGTGTAC
GAGAAGTACCGCACGTGCGGCGAGATCACCAACCGGGTGCTCGCCAAGGTTGTGCAGGCT
GCCGTCCCTGGCGCCAAGATCATCGATCTCTGCGTGTTGGGCGACAAGTCCATTGAGGAG
GGCACCGCTGCCGTCTACAACAAGTCCAAATTCGAGCGTGGTGTGGCCTTCCCCACCTGC
GTCTCCGTCAACAACTGTGCCGGCCACTTCTCGCCCCTCACCGGCGATAACACCGTCCTT
CAGGAGGGCGATCTCGTGAAAGTTGATCTCGGAGCCCACATCGATGGCTTTATCACACAG
GGTGCCCACAGCTTTGTCTGCACGGCCAACAAGGATCAGCCCGCCACTGGCCGTCACGCC
GATGTGATCTGCGCTGCCTACTTTGCTGCTCAGGCCGCCCTCCGACTGTTCAAGGCTGGT
AAGACGAACCAAGAGATTACCGCCACCATCCAGAAGGTAGCCGACGAGTTCAAGGTCCAG
CCGCTCGAGGGTGTGCTCTCGCACCAGACCAAGCGTTTTGTCGTCGATGGCAACAACGTG
GTTATCAACAAGGAGACCATCGACCAGAAGGTTGAGGACGTCACCTTCGAGGATTGGGAC
GCCTACACCTTTGACATTGTGATGTCCACCGGCGAGGGCAAGGCCAGGGAGACCGAGGCC
AGGACCACCATCTTCAAGAGGGCTGTCGACCAGCGGTACACGCTCAAGCTCAAGGCTTCG
CGCGCCGTCTTCACCGAGATCAACACCCGCTTCCCCACCATGCCCTTCACCCTCAGGGCT
CTCAAGGACGAGAAGACCGCCAGGCTCGGCATCACGGAGTGCCTCAAGCACGACCTCGTT
GAGCCCTACCCCGTTCTCCACGAGAAGCCCGGCGAGCTTGTTGCTCAGATCAAGTTTACC
GCCATCATCACCCCGTCGGGCACCGTGCGCATGACCACCCACCCCGAGCCCCTCGTGCGG
TCGGACTTCAAGATCACCGACGCTGGTGTGAAGAGCATCCTCGCCTCGGGCGTGAAGAAG
GGCGCCGCCGCCAAGAAGAAGAAGAAGAGGAGCAAGAAGGCCGCTGCCGGTGGTGTCCCC
GCCGCCGCTGCCGCCGTCGAGGCCGGAGCCGGTGGTGCCCCCGCTGCCGCCGCCGAGAAG
AAGGAGGAGAGCGCTTAA
>g185-569957-572411
ATGTCTCTCAAGGGCATTTCTGTCTCGTCTCTCACAAATCCGGACAAGGACGGCTACCTG
ACCAAGCAGGGCGGCAACATCAAGACATGGAAGAAGAGATGGTGCGTCCTCAAGGACGGC
TCGATCTTCTACTTCAAAACTCCCAAGGATCAATCGCCCAAGGGAACGATCGATCTCGAG
AAGAGCTCCAAGCTCTCGGAGACGACCCTCACTAAGAAGAAGTACGCCTTCCAGATTCAG
ACGGCACAGAGGACGTTCGTGATTACGGCCGAATCGGAGAGCAACAAACGAGAATGGATG
GCGGCCATCAATGCCAGCGTGGAGAACCTGCAGAAGCAGAATCGCGAGGGCGGTTCTGCC
CCTACGCCGACCGCCTCTGCCGAGAATGGCGAGAAGGCCAATGGAGAGGTGAAGATCAAG
GATCCATCGGACGAAAAGACTGAGACGAGGAAGGAGGGAGAGACGAAGGACACGAGCGAA
TCGACCTCGACCACCCCCTCGCCCGCAAAGAAGGATGAGAAGCCCAAGGCTCTCAAGCCT
GCCAACGCCTCGGCGCGCACCCAGCTGTCCCTGGCCAAGGGCTGCATCCCCTTCCTGCAG
GAAGACGAGAGCAAGGTGCTGGAGTTCTGGCAGATCTGGTCCGAGAGCATCCCCACGGGC
GATGACATCCCCAAGGACATGGCGATCGAATTCCACGTCTTCACGTCGGCCAGCATGCAG
AAGCTCACCTGGAGGACTGCCGGCCCCCAGAACATCTTCATCCAGCGAATGGTCGATTTC
TTCTGGAACGTCGGTGCGCCGGAGAGCGAGATCGACCGACTCAACGACGTGGGCGCCCTC
ATCAACCCGGTCAAGATAGGATCGTGGATCGACATGTCGGGCAAGGGCGGCATGGACGGC
GGCTGGTACTTCCCCGTGGAGATTCCCCTCAGCCTCGCGCTCGAGGCCGCCGACGCCGGC
GAGCCCTCCGTCAAGGTCAAGGACTGGGGCACGCGACACAACATCGACAAGGTCTACAGC
GTGGGCCGCGACATGGGTGCCGCTCCTCCTCGCCAGACCGAAATTCGAGTCAAGCTCCCT
GGTGCGACGTTCCAGGATCAGCTGAGGCAGGCGCTTGACGCCTTCGAGAGCTTCGGCTTC
CCCAACATGCCGGAGGACGCGCTCGACGTGCTCAAGAACTCGAGCGTCACCCCGCTGTGC
CTCTCGATCGTCACCTCGTCGGAGGGCTTCGTGCGCCTTGGCGTGCTGCAGCCCAACCCG
ACCACCGACAACGTGCTCACGCTGTGCGACATCTCTGGCGCCAACAAGGACGACCTCGCC
GCATTCGAGGGCGCGCTCGGGTCCGACGGCCCGGCCTACGCCGAGTACCAGTACCTGATG
AAGGGCTTCGGGTACAGCGTGTACAAGGAAGGGTTCGACATCATCTTCCACTATTTTGTT
GGCGAGGAGCGCGGCGCGGACGAGTAA
>g185-581788-585009
ATGGAGGACCAGAAGAAGTACCTGAACGAGCTGAGATTTTTGACTCCTGAAGAAATAGCG
GCCTTGGCCGAGAAGATCATCGCCGATTCCACCAAGGTCCAAGATGCAGTGGCAGCATTG
CAAGATGGAGAGCACACCTTTGCAAACACGGTGAAGGCACTCGCCAATGACGAGCTTATT
AGCGATACTCTTGCAGCCAACTGCTACTTCCCGTCCTACGTCTCCACAGACAAGGCCACC
AGGGATGCGAGTACTGAAGCCAACAAGAAGATCGAGGCGTTCGGCATTGAGTCGAACATG
CGCGAGGACGTATATCAGTCGCTCCTCAAATACAAGGCCAAGGGCGAACAGCTCGGCCCT
GTGGACCAGCGGCTTCTTGACAAGCAGCTGGAGACCTTCGAGAGAAACGGCCTGGGTCTT
CCTAAAGAGGAGCGCGAGAAGCTCAAGGCCCTCAAGAAGAGGATGTCGGAGCTGTGCATC
CAGTTCCAGCAGAACATTAACGAAGACAAGACTTCGCGCGAATTCACCAAGGAAGAGCTG
GCTGGTATGCCCGATGACTTCATCGAGAGCCTGGGCAAGTCTGAAGATGGCCAAAAATAT
GTGGTCACGCTCAAGTATCCCGAGGTCTTCCCCCTGCTGCAAAAGGCTATCAATGAAGAG
ACCCGCAAGACCATGGAGTTTGCCGACTCAACCCAGTGCATGAAGGAAAACGCGCCTATT
CTCGAGGAGGTCATCAAGCTTCGGCACCAGGCCGCTCAGCTGCTTGGCTTCCCCACCCAT
GCTGACTACATCCTCAAGATCAGAATGGCCAAATCCGTTCAAAACGTGCTCGAGTTCGAG
AACGGGCTTAAGGACAAGCTCATTCCCTTCGGACTCAAGGAGAAGGAGAAGCTTCTCAAG
CTCAAGGAAGAGGAGAAGAAGGAGTTGGGTCACTCGTTCGACGGTGAGATCAATGCTTGG
GACTTCAGGTATTACCATCGCCTGCTGCTCGAGAAGGAGTACGAAGTCAACGATGACGAG
ATCAAGGAGTACTTCCCTATTGAAGTCGTGACCAAGGCCCTTCTGGAGATCTACCAGGAG
ATCCTCGGCTTCAGGTTTGAGGAGGTGGAGAAGCCCTACGTGTGGCACGAAGACGTGCAA
CTCTTCTCCGTGTGTGATAAGGACTCGAACGATTTTATTGGCCATTTTTATCTTGATCTC
TACCCGAGGGACGGCAAGTACACCCATGCTGCGGCCTTCCCGCTTCAGCCGACCGGTATC
AAGTCTGATGGCTCTCGCCAACACCCAGCTGCCGCCATGGTGGCCAACTTCTCGAAGCCG
GGTAAGGACAGGCCCTCGCTCCTCAAGCACTCGGAAGTGGTCACCTACTTCCACGAGTTT
GGTCATATCATGCACAACATTTGCTCCACTGTCACCTACAGCCGCTTTGCTGGCACTTCC
GTTGAGAGGGACTTTGTGGAGGCGCCTTCGCAGATGTTGGAGAATTGGTGCTGGGAGAAG
GACATCCTGTACAGGCTCTCTGGTCACTACAAGGACAACTCCAAGCACCTGCCCGACAAC
CTGCTCTCGAAGATGGTAGCCGCCAAGAACGTCAACACCGGCCTCCTCAACCTCCGCCAG
ATCTTCTTTGGCCTGTACGATCAAACCATCCACTCGCAGCCGGAGACCGACACCGCTGCC
CTGTGGCACAAGCTCAAGACCGAAGTGTCCCTCGTGGGCAGCACCCCTGGCACCAATCCC
TCGGCCAGCTTCGGCCATCTGGCGGGCGGCTACGATGCTCAGTACTATGGCTACCTCTAC
TCGGAGGTCTTCTCGGCCGACATGTTCAGCCGATTCAAGAAGGAGGGCATCCTCAGCCCC
AAGGTGGGCAAGGAGTACCGGGAAGTCATCCTGTCGCGCGGCGGATCGGTGGACAGCATC
ACCTCCCTCGTGGAGTTCCTCGGCCGGGAGCCCACGCAGGAGGCCTTCCTCGAGCACCTG
GGCCTCACCGAACCTACCAGCGCCTAG
>g185-587757-588901
ATGGGTTTCTTCACCAAGCAGAGGTATGACTACGCCGGAATGCTTGCCGTGAGGCTCCAG
AAATTCATCATCTTCCTCTTCGTGCTGAGCCTCGTCTGGTTCGGCGTGCACGTCCACGCC
ATGGTCAAGCACGGCCAATTCGTCCCTGCCCCCTTCATCGCGGCTAACATTAGCTGGCTC
ATCATGTATGCGGGTTTCATTGGCGCCTACAGGCGCAACACCGCTCTCTTGATGTTCTAC
TTCATCGTCTCGCTCCTGGGCGCCCTCGCTCTCATTGCCGGCTTCGTTGTCGCTTCGGTT
GGCATCAGCCTGGCTGTCCTCCACGACTGCCAGGAGACTGCCGGCTGCGACGCCCGCGCT
GAGGCCCCCAAGGACGTCACCGTCGTCACTGCCATCGTCTTCGCCTTCACCATCGTGCCT
CTCCTCCTCAAGGTTGTGGGCGCCGTTCTCGCTCTCGTTACCAGGCGCGAGATCCTCATT
GCCCGTGCTGAGGCTGCCGCCAAGCAATCCATGATGGAGGAGGGCATTACCATCCCTGCC
GACGAGATTGAGGTCGAGTCCCCCAAGGCCGCCAAGGTCGTCGACTTGCCCGAGGTGGCC
CAGCAGCAGATCCAGTACATGCCCATGCCCGCCTTCTACCCTCCCTCCGGCCAGAGCTAA
>g185-615397-617261
ATGTCGTTCCCAATCTATGCGGTGGTGGGCATCCCGGTGATCTTCCTGCTGGGCCTGCTG
GGTGCGCTCACCCCGCCTACCATCGCCTTCTTCTTCCCCAAGTACGGCGTGACCCAGAAG
TATTACTTCACCTTCTTCAACGGCCTCGCTGCCGGACTCATCCTCGCCGTCGGGTTCATC
CACTCGATACCGGACTCGTTCGAGTCGTTCGGTGGGGTGCTGACGGACGAAGATAGCCAG
GTGGAGTCGTACGCGTGGCCGGCGTTCATCGCCATGATGGGCGTCATCATCTGTTTCACG
ATCGAGGAGGTGGTCGACACGCTGAGCGCGCTCTTCGGCGTGTCCAACCTCCACTCCCAC
GGTGGCGCGCACGGCCACGGCCACGGCGCCGCGCCGCACGCCGGGCACGAGGACCACGCC
TGCGGCCACTCGCTGGAGGACCACGAGCAGAACGACGGCGAGTGCGACGCGCAGCCGCTC
GAGGAGATGTGCGACCACCACCACGGCCACAGCGTGTCGACCGATGACGAGGAGGACAGC
AAGAACCTGGAGGGCCTCGGCAAGAAGAAGGTCAAGAAGGGCGACGCGCCCAAGGAGTCG
GACGACGAGCTCGACGTCGACGGCGACCTCGTCAAGGCCGCCATTGACCGCAACGCCCAC
ACCAAGCGCGTCGTCAAGATGTTCGTCCTCTTCTTCGGCCTCCTGTTCCACAACGTGTTC
GTGGGCCTGGCGCTGGGCACGGCGGACAACGACCACGCGCTGTTCATCGCGATCGTATTC
CACCAGTTCTTCGAGGGTCTGGGTCTGGGCTCGCGCGTGGCGACGGCCAACCTGAAGCGA
TTCATCTCCATCCTCATCATCGACATCATCTTCGCCGCCTCGGCGCCCGTCGGTATCGGC
ATCGGCATCGGCGTCAAGTCCGCGCTTGAGGACGACGACTACGCCTACAGTATCGTCGAC
GGCACTTTCCAGGCCCTGTCGGGCGGCATCCTGATCTACGTGGCGTTGGTCCACATGCTC
CGAGGGTATGCCGAGGTCGATATCAAGGGCATGGCGCTGCACTGGCACAAGCTGAGCTCG
TACCTGGGTCTGCTCTTGGGCGCTGCCGCCATGGCGGTGATCGGTATCTGGGCCTAA
>g218-5197-6688
ATGAGCAGATCCGCTGGCTACGATCGTCACATCACCATCTTTTCGCCTGAGGGGCGTCTG
TACCAAGTCGAGTATGCCTTCAAGGCCATCAAGACTGAGAACCTCACATCGCTGGGCATC
AGGGGCGCAGACAGCTGCTGCGTGATTACACAGAAGAAAGTGCCCGAGAAGCTCGTGGAC
CCCACCTCTGTGACACACCTGTTCAACATCACCCCTACCATTGGCTGCGTCATGACTGGC
ATGATCGCCGACGCTCGATCGCAGGTGCAGCGTGCCAGAAAGGAGGCTGCTGATTTCGCC
CACAAGTTCGGCTATGACATCCCAGTGTCCTACCTGGCCAAGAGGATGGCCGATATTGCT
CAGATCTACACCCAGCACGCCTACATGCGCCCGCTCGGTGTCTCGATGATCCTGATCAGC
ATCGACGAGGAGGAGGGTCCTCAACTGTACAAGTGCGACCCGGCCGGATCGTACGCCGGG
TTCAAGGCCACCGGTGCCGGCCACAAGGAGCAGGAGGCCCGCAACTTCCTCGAGAAGAAG
TTCAAGACCGACCCCAAGCTCGACGCTGACAAGACGATCAAGATGGCGATCAACGCGCTG
CAGACGGTGCTGGGCGAGGATTTCAAGGCATCGGATATCGAGGTGGGCGTCGTCACGACC
GACAGCCCTCGGTTCAGGAGGCTCTCCGACGACGAGGTCGACCGCCACCTCACCGAGATT
GCTGAGCGCGATTAA
>g218-21116-22240
ATGCAGGGTCAGGTTCCTACTTTCAAGCTCATCCTTGTCGGCGATGGTGGCGTGGGTAAG
ACTACCTTCGTGAAGCGCCATCTTACTGGTGAGTTCGAGAAGAAGTATGTCGCCACGCTT
GGCGTTGAGGTCCACCCTCTCTCCTTCCACACCAACTTCGGCCCCATCTGCTTCAACGTG
TGGGATACCGCCGGCCAGGAGAAGTTCGGAGGTCTTCGCGATGGTTACTACATCCAGGGC
CAGTGCGCCATCATCATGTTCGACGTTACGTCGCGCATCACCTACAAGAACGTCCCCAAC
TGGCACAGGGATCTTGTGCGAGTCTGCGAGAACATCCCCATCGTTCTTGTCGGAAACAAG
GTCGACGTCAAGGACAGGAAGGTCAAGGCTAAGGCCATCACTTTCCACCGCAAGAAGAAC
CTGCAGTACTACGACATCTCGGCCAAGTCGAACTACAACTTCGAGAAGCCTTTCCTCTTC
CTCGCCCGCAAGCTCACCAACAAGGCCGATCTTGCCCTCGTGGAGTCGCCCGCCCTTGCG
CCCCCTGAGGTTGCCCTCGACATGAACCAGATCAAGCAGTACGAGCAGGAGCTGTCTCTG
GCTGCCCAGACCCCTCTCCCCGATGAGGATGAGGACCTGTAA
>g218-23738-24225
ATGTTTTTCCACACAGCAAGAATCACAAAATCATTTGATTTAGGGGGACCGGGGTGGTCA
ACCTGGTTCACTCCAGTCTACCCCCCCTCCCTCATTGCACACACGAGGGAGGTTCTGCAC
AACACAAATTCGCCGGTCGAGCCCGTGGCAGGGGAGGGCTCTCGATTCGGACAAGCAACC
AGGGGCTACAGAGCCGGTAGCCCTCCAACCACGAACGAGGAAGAACTGTCGACAGTCAAG
GCGCAGACAGACACCCCCCGGCAGAGTTTCGGGGTCGAGCTCGTTCTCCGCAATCTCACT
CCCGCAGCTCCTCCGACGTGGCTGGCATTCCTGGGGAACTGGCAGCAATTTGGGCCAGCC
TAA
>g218-56086-57340
ATGCTGAAGAGATTCTCCTACGTGCTGGGCAATACTGTGCGAGAGACCGCCTATGCGCTC
GACCGCGTTGGTTGCCGCCTCCAGGGCAACTACGCCTTCACGGAGGAGCTGAGCAGGCAC
AGGAGGGTCATGGGCCTGTACGATAAGCAGCCTGCCATTTCACAGGACGTTTTCATCGCC
CCCAATGCCAGTGTCATCGGTTCCGTCTCGTTGGGCGAGGGCGCCAACGTTTGGTATGGC
AGCGTTCTTCGCGGCGATGTTAATGACATCTCCGTGGGCAAGAAGAGCTCCATCGGCAAC
CGGTCGGTTGTTCACGCCTCGGGGGGTCTCACAACCCTGGCCCCCACCAAGATTGGCGAC
AACGTCGTCGTTGGTGACGGCGTCGTGCTCCACGGCTGCACGCTGGAGGACGAGTGCCGG
GTCGACGACGGGGCCGTGCTGAACGACAACGTGGTCGTGGAGAAGCACGCCATCGTGGGC
CCCGGTGCCGTCGTCACCTCGGGCAAGAGGGTGCCTTCCGGCCAGGTGTGGGCGGGCAAC
CCGGCCAAGTACGTGCGGGACGTCTCCGAGGAGGAGAAGGAGTTCGCCGGCTGGGCTGAG
AAGCGGTACACGCAGGCCAAGGCCCACCTGGCCCAGACCATCAAGCTCGCCGAGGAGAAG
GAGGTCGACCTGCTCACCGAGGACATCCTGCGCGAGATGCGCCCCGGCACTCGCTTTGCC
GACTAA
>g218-57377-58738
ATGTTGGTGCTCGTAATTGGTGATCTGCACATCCCGTACCGTGCTCACGGTCTGCCCAAG
AAGTTCAAGAAGCTTTTGGTTCCGGGCAAAATCCAACACATCTTGTGCACCGGCAACCTG
TGCACAAAGGAGGTCTTCGAGTACTTCAAGACCCTCGCCAACGACGTACACATCACCCGC
GGTGATTTCGATGAGAACACCAAGTACCCCGAGAACAAGGTGCTCACGCTGGGCGAGTTC
AAGGTGGGTCTGTGCCACGGCCACCAGGCGGTGCCGTGGGGCGACAGGGAGAGCCTGGTC
ATCCTCCAGCGACAGCTCGACGTGGACATCCTCATCACCGGCCACAGCCACAAGTTCGAG
GCCTTCGAGTACGAGAGCAAGTTCTTCATCAACCCGGGCTCCGCTACGGGCGCCTACTCG
GGCCTCAACGTGGAAGCGACGCCGTCGTTTGTGCTGATGGACGTGCAGGGCGCCCATGTC
GTGACCTACGTCTACCAGCTCATCAACGACGAAGTCAAGGTGGAGAAGATCGAGTTCAGG
AAGAGCTAG
>g218-68723-70868
ATGTCTCGCGTGGTTCGTCAGTCCAAGTACAGGCACGTCTTCGGTACCCCCCAGAAGCCT
GAGAACTGCTACACCGACATCAGGCTCTCGACCAACCAGTGGGATTCCAACTACGTGACG
GCCAACACCAAGTTCTTCGCCGTGTGCTGGGAGGCCGCTGGTGGCGGTTCCTTCGCCGTC
GTCCCCTGGGTCCAGAAGGGCAAGCTGAAGGCCGACTACCCCCTCGTCTCCGGCCACAAG
GGTCCCGTCCTCGACGTCGACGCCAACCCCTTCAACGACTACCTCTTCGCCTCGGCCTCT
GAGGACGGCACTGCCAAGATCTGGAAGGTCCCCGAGGATGGCCTGACCGAGACCATGCGC
GACCCCGTGCAGAACCTCTCCGGCCACAAGAGGAAGGTCGGTAACGTCCGCTGGCACCCG
ACCGCCAACAACGTGCTCGCCACGTCGTCGACCGATTACACCGTCAAGGTGTGGGACGTC
GAGAAGGGCGCCGCCAAGTGCCACGTTGATGGTCACGCCGACATCATCCAGTCCATCGAT
TGGAACTACGAGGGTTCGCTCATCGCCACCGCTTGCAAGGACAAGAAGATCAGGATCATC
GATCCCCGTACCGGCCAGGTTGTGTCGGAAGCCGCCGCTCACACCGGTGTGAAGGGCAGC
CGCGCCATGTTCCTCGGCCGCACCGAGAAGGTCTTCACCGTCGGTTTCTCGCGTACCTCG
GACCGTCAGTACGCCATCTGGGACCCCTCCAACATGGGCACCGCCCTCGCCCAGGAGAAC
ATCGATACCGGCTCCGGTCTCCTCATGCCCTTCTTCGACCCTGACTCCAGCATCATCTTC
CTTGCCGGCAAGGGTGACGGTAACATCCGTTACTACGAGCTGACGGACAACGGCTCGAAG
ATCTACTTCCTGTCGCAGTACCAGTCGAACGTGCCCGCTCGCGGCATGGCCTACTACCCC
AAGTACGGCGTCGACGTGGGCTCGTGCGAGATCTCGCGTCTCATCAAGGCCACCACCACT
GGTATCGAGCCGATCTCCTTCAACGTGCCCCGTAAGGAGGGTCTCGACGTCTTCCAGGAC
GACATCTACCCGCCCACCGCCGCCCCCGAGCCCACCACCACCGCCGAGGAGTGGTTCGGC
GGCAAGACCGTGCCCCCCAAGCAGATCTCGCTCGAGGGTGGCTTCGTCGCCAGGGAGAGG
CCCGCCGACTTCAACCCGACCGAGGTCAAGTCCGTCGAGGACGAGGGCCCCGCCTCCGAG
AAGGAGCTCAGGGCCGAGTGGAGGAAGCAGAAGGACCGCATTGCCTTCCTCGAGAGCGAG
CTGGCCAAGAAGGACGCCCTCCTCAAGAACCAGGGCAACTAA
>g218-115547-116688
ATGTCTCCGCGCGGTGCTGGAATATTTCCCCTTTTGGAAGGCCCGGCCCGCCTGACGTCA
GGCCTGCGCGCGAGCAAGGCCCTTTGTTGTTTTCATCTCTCGCCTCGAGCAGACACTCAC
CCCCAGCCTCCGACGATGTCCACCCACGCCGTTATTGGTAAGCCCGCCCCCGACTTTGAC
GAGGAGGCCGTCCTCGGTCAGGACTTCACCCGCGTCAAGCTCTCCGACTTCAATGGCAAG
TACTTGGTGCTTTTCTTCTACCCGCTCGACTTCACCTTCGTGTGCCCCACTGAGATCCTT
GCCTTCAGCGACCGCGCGGACGAATTCCGCAAGATCAACACCGAAGTGGTCGGCGTGTCC
GTCGATTCCAAGTACTCGCATCTGGCCTGGATCAACACCCCGCGCAAGCAGGGTGGCCTC
GGTGGCGCCCTTAAGATTCCTCTCGTGGCCGATCTTACCAAGAGCATTTCGCGCAAGTAC
AACGTTCTGATGGAAGAGGCCGGACACACCTACCGTGGTCTCTTCATTATCAATCCTAAG
GGCGTGCTCGTTCAAGCCACCCTGAACGACGCTCCTGTCGGTCGCAGCGTCGACGAGACT
CTTCGCCTCGTGCAGGCCTTCCAGTACGTTGACGAGCATGGCGAGGTCTGCCCTGTCAAC
TGGACGCCTGGCTCGGCCACCATGAAGGCCGACCCCAAGGAGTCGCTCGCCTACTTCGAG
AAGGTCAACCAGTAG
>g218-238264-239590
ATGACGACCGAACGCGAGGCCAACATCTACGAAGCCAAGCTCGCCGAGCAGGCCGAGCGC
TACGACGAGATGGTGGCGGCCATCAAGAAGGTCGCTGCGTCGCTGAAGACCGGTGAGGGT
CTGTCCGTCGAGGAGCGCAACATCTTCAGCGTGGCCTACAAGAACGTCATCGGCTCGCGC
AGGGCTACGTGGCGCATCGTCTCCTCCATCCTCAAGAAGGAGGAGGACCGACCGGAGAAG
ATCGAGATGAGGATCAAGCACGCTCGCGCGCTGGCCAACAAGGTCGAGGGCGAGATGAAC
TCCATCTGTAACGACTGCCTCAAGGTCATCGACGACCACCTCCTCCCCTCGGCCGCCTCG
GATGCCGAGAGCAAGACCTTCTACTACAAGATGAAGGGCGACTACCACCGGTACATGGCC
GAGTACTCGTCCGGCGAGGGTCGCCAGAAGGCCGCCGAGGCCTCCCTCCAGTCCTACCAG
TCCGCCGCCGAAGTCGCCAAGGAGCTGCCGTCGACGCACCCGATTCGTCTGGGCCTCGCG
CTCAACTTCTCCGTGTTCTACTACGAGATCCTGTCGTCGCCCGAGAAGGCGTGCCAGATC
GCCAAGCAGGCCTTCGACGAGGCCATCAACCACCTCGACGGCATCGGCGAGGAGGAGTAC
AAGGACGCGACGCTCATCATGCAGCTCATCAGGGACAACCTCACGCTCTGGACTTCTGAT
ATGCAGGAGGGAGACGAAGACGACGACGACGAGGGTCAGGAGAAGAAGGAGGAGCAGGAC
TAG
>g227-92186-93690
ATGGCCACGTACAAGGTTGTGTTGATTCGCCACGGCGAGAGCACCTGGAACAAGGAGAAC
CGCTTCACGGGCTGGACTGACGTCGATCTCTCGGACAAGGGCATCGAGGAGGCTCTTACC
GCTGCCAAGGCTCTGAAGGACGGTGGCTACACCTTCGATGTTGCCTACACCAGCGTGCTC
AAGCGTGCCATCAGGACTCTCTGGCTCGTCCTCAACGAGCTCGATCTTAACTGGATTCCC
GAGCACAAGAGCTGGCGCCTGAACGAAAGGCACTACGGTGCTCTCCAGGGTCTGGACAAG
GCCGAGACCGCTGCCAAGCACGGCGAGGACCAGGTCAAGATCTGGAGGAGGGCCTACGCC
ATCCCTCCCCCCGCTCTCGATGAGACCGACGAGCGCTTCCCCGGCAAGGACAGGAGGTAC
GCCGATCTGGACGCCGGCCTGCTGCCCAAGACCGAGTCGCTGGCCGACACCGTCGAGCGC
TTCCTGCCCTACTGGCACGACACCATTGCCCCCGCCATCAAGTCCGGCAAGCGTGTCATC
GTCGCCGCTCACGGCAACAGCTTGAGGGCGCTCGTGAAGTACCTGGACAATGTGCCTGAG
TCTGAGATCACCGAGCTCAACATCCCCACCGCCATCCCGCTCGTCTATGAGCTCGACGCC
GACCTCAAGCCCATCAAGCACTACTACCTCGCCGATGAGGAAACTGTGCGCAACGCCATC
GCTGGTGTGGCTGCCCAGGGCAAGGCCAAGTAA
>g253-178716-180130
ATGGGAGACGAGGTTCAGGCTCTGGTTATCGACAACGGTTCGGGCATGTGCAAGGCTGGC
TTCGCCGGTGATGACGCCCCCCGCGCTGTGTTCCCCTCGATCGTCGGTCGTCCCCGCCAC
ACTGGCGTCATGGTCGGCATGGGCCAGAAGGACTCGTACGTCGGTGACGAGGCCCAGTCC
AAGCGTGGTATCCTCACCCTCAAGTACCCCATCGAGCACGGCATCGTCACCAACTGGGAC
GACATGGAGAAGATCTGGCACCACACCTTCTACAACGAGCTCCGCGTGGCCCCCGAGGAG
CACCCCGTGCTGCTCACTGAGGCCCCCCTCAACCCCAAGGCCAACCGCGAGAAGATGACC
CAGATCATGTTCGAGACTTTCAACACCCCCGCCATGTACGTCGCCATCCAGGCCGTGCTC
TCGCTCTACGCCTCGGGCCGTACCACCGGTATCGTGCTCGACTCCGGCGATGGTGTCACC
CACACCGTGCCCATCTACGAGGGTTATGCCCTGCCCCACGCCATCCTGCGTCTCGATCTC
GCCGGTCGCGATCTCACTGACTACCTCATGAAGATCCTCACCGAGCGCGGCTACTCGTTC
ACCACCACCGCCGAGCGCGAGATCGTGCGTGACATCAAGGAGAAGCTGTGCTACGTCGCC
CTCGACTTCGAGCAGGAGATGCACACCGCCGCCTCGTCGTCGGCCCTCGAGAAGTCGTAC
GAGCTTCCCGACGGTCAGGTCATCACCATCGGTAACGAGCGCTTCAGGTGCCCCCGAGGC
CCTGTTCCAGCCCTCGTTCCTCGGCATGGAGTCGGCCGGTATCCACGAGACCACCTACAA
CTCGATCATGAAGTGCGACGTCGACATCAGGAAGGACCTCTACGGTAA
>g253-184876-188554
ATGAGGAGCGTCGCACTGTTCCTCTTCGCCTATGCGGCGCTTTGCTTCATCTACACAGCC
TCGGCGAAGGCTCCGCTTACGATCGTGAGCCCCACCGAGGCGCTTGACCAGACCCAGTAC
CAAATTGGTGCGGGCATCTATGACATCACCGGTCCTGCGGCTCAAATCGGCATGATGGGC
TACGCCATGGCCAACCAAAGCACTGCTGGCGTGCACTTCAGGCTGAGGGCTCGCGCTTTC
ATCATCGCCGACGGCACCAACAGGGTCGTTTTCGTGAGCACGGACTCTTGCATGATCTTC
ACCTCGGTGAAGAGGGAAGTCGTTCTTGCCCTGCAGAGGAAGTACGGCAAACTGTACAGC
CACGACAATGTGATGCTCAGCGGCACCCACACCCACTCTGGTCCCGGAGGCTACGCCGAG
TATAGCATCTACCTCATGACCACGCTGGGCTTCAACAAGGACAACTGGCACACGATCATC
GATGGCATCGTGAATGCGATCTCCGCAGCTCATGATAGCGTCAAGCCTGGCAAGATCATG
GTCAACCAAGGTGAGCTCCTGGACTCCAATCTCAGTCGCTCGCCCTTCTCGTACCTCAAC
AACCCGGCCGAAGAGAGGGCCAAGTACCAGTACAACGTCGACAAGAACATGACCCTGCTC
AGGTTCGAGGACATGAACGGTAATGAGATCGGCATGGTCAACTGGTTCGCCGTGCACGGT
GTCTCCATGACCAACCAGAACAAGCTCATCAGCGGCGACAACAAGGGCTACGCTTCCTAC
GCCTTTGAGAAATACAAGAACGGACCCAAGTCGCTGCCCGGCATGGGACCCTTCATCGCG
GCCTTCGGTCAGAGCAATGAGGGAGACGTGACTCCCAACACCAGGGGCGCCTTCTGCAAC
AACGGCATGCCGTGTGACTTCGCTCACTCCACCTGCGGCGGCTATTCGGAGAACTGCCAC
GGCTACGGCCCGGGCAAGGACCAGTTCGACAACACCAAGATCATCGGTACCAACCAGTTC
AAGAAGGCTCTTGAGCTGTACGAGTCCGCCAAGGAGCCCATCAGCGGCCCGATCCAGTTT
GCCCACACTTTCGTGAACATGCACAACGTCATCGTCCAGCCCGCTTTCACTGGCCTCTCT
GCGCCCGTTTCGACTTGCGTGGGTTCTCTCGGTGACGGCTTTGCTGGCGGCACCACCGAC
GGCCCCGGCGACTTCAACTTCAAGCAGGGAACCAACAGCACGAGCACCAACGCCTGGTGG
AACTTCATTGCCCACTTCCTTTCCGAGCCCACTGACCAGGACATCAAGTGCCAGCACCCC
AAGCCCATTCTGTTCAACTCGGGCGGTATCGACCTGCCTGCTCCCTGGACTCCGCACATC
CTCCCTCTGCAAGTGTTCCGCGTTGGCCAGCTGGTGATCATCGGCGTGCCCGGCGAGTTC
ACCACCATGTCTGGTCGCCGTCTGCGCGACACCGTCCAGAAGGTGATGTCGAAGCACGGC
ATGACCAACGGCATCCCCGTGATCGCCGGCCTGGCCAACGCCTACTCGCACTATATCGCC
ACCCAGGAGGAATACCAGGTGCAGCGTTATGAAGGTGCCTCGACCCTCTACGGTCCCTGG
ACTCTGGCTGCCTACCAGCAGCTGTACACCTCCCTCACCGATGCCATGCTCTCTGGCACC
CCTGTCCCTCCGGGTCCCACTCCGCCCAATCTCTACGACCGGACCTTCACTCTCCAACTG
CCCGTCATCGAGGACTCGGCTCCCGGCGCGTTCGGCCAGGTCGACAAGGACGTCTTGCCG
TCTTACTCGCTCAACTCCACCGTCACCGTGTCCTTCTGGGGCGCCAACCCGCGTAACGAC
TTCAGGACCCAGGACACTTTCCTGACCGTGGAGCAGCTTCAGACCGACGGCAGCTGGAAG
GTGATGCTTGTCGACGGCGACTGGGACACCCGCATGTACTGGGAGGAGGTCTGGCTCATC
CAGTCCAGGATCACCATCACCTGGCAGATCGCCGCCGACACTGCTCCTGGCAAGTACCGC
ATCAGGACCTTCGGCAAGTCCAAGGACCTCCTGGGCTACTTTACCCCCTACACGGGTACC
TCCAGCACCTTCACGGTCGCGTAA
>g253-217629-219378
ATGGCCAAGTCAGTGGTGCGCGTGCATTTCATGGACTCGAACTCGAAGGCGTTCGCCATC
GACGCGGGAGCATCGGCGGACGCCATGCTCAAGCTCGTCATCGAACGTCTCGAACTGAAA
GAGTCGGCCACATTCGCTCTCTTCGAGAAGAAGGACGACTGGGAACGCTGCCTCGAACCC
GACGAGAAGCCCGCCGAGCTGATGAAGGCCTGGACCGGCGACCCCAAGGACGAGAGCTCG
CCACGGTTCTTGTTCAAGAAGAAAACCTTCATTCGCGACGACGACCGCGAGCTCAGCGAT
CTCGTGGCTCGCCATTTGGTCTACATCCAGGCCCTCGCCTCCGTGGTCAACGCCGACTAC
CCGACCTCCCCCGAGCAGGCCGTTCGACTTGCCGGCCTCCAGGTGCAGGTGGTCTACGGT
GACCACAACGCCGCCACCCACGTCCCCGGTTTTCTCACGACCAATCTCAAGGACTACATC
CCCAAGACCCTGTTCGCGACGAAGAAGCCTGCCGAATGGGAGTCCATCATCTTCGCCGAG
CACGCGAAGCACAAGGGCAAATCGGCGGACGAGGCGAAGCTGGGCTACCTGTCGCTGGTG
AAAAAGTCGCAGTTCTACGGCACCACCTTCTACCCGCCGTGCAAGAGCGTCAACAACGGA
CGAAAGATCCCCAACAAGGTCATCATCGGCGTCAACGCCGACGGAATCATGCTGCTCAAG
CCCAAGGACAAGGAACTCATCTCTACTCACCCCTTCACGGAGATTTGCAGCTGGGCCTCC
TCCTCGACGACCTTCGCCTTCGAGTTCGGCGTGCAGTCCGAAGCGACCAAGTACACCTTC
GAGACCAAGCAGGGCGCCATCATCGCCTCCACCATCCAGACCTACATCGATATCCTCGTC
GAAATGCTCACCAACGGCAACGAGGAGGACGAGTCCACCGCCACCGGCACCTCGCACGGC
TCCGAGGGCGACGATTGA
>g253-305426-308180
ATGGAGAAGATACTCGCCGAGATACGGTCTCACGAGAAGTCTGCGCTCTTTGCCGGCACG
AGCGCACACAATCCTGACCTCCAGATGTCTCTCGACGCCGTCCAGCACAAGATGGACAAG
TGCCTCTACAAGAAGCCGAGGGATTTCGTACATGACATGAACACGGTCTTCACCGTTGAT
GCTGATAGCCCTCTCGCTTCCGCGGCTTCTAGCTTCAGAATACTCTTCCAGTGCCTGCTG
GACAAGTACGACATGGTCGGCAGCCGCCAGAGCTCATCCAAGAAACACAGGCGTGGCAGC
AGCAAGCGTGCCAAACCCGAAGTGTTCGACTTCGTTGCTGAGTTTGAGGAGGAACTGAGC
TCTGGCTTCCTGTTTGAGGGCGAGCAGTTCCTCGACGGCGGCAGCTCCTCCGACGAGCAC
TTCTTCGGCAAGGACGACGACGACGACTCGTCGGCGGAGGACTTTGTGCCCCTCAGCTTC
TCGTTCGAGCAAGAGTCCGTCCTCGACGGTCGCTCGCCTGCCCTCTACTCCGAGCTACTC
GGCGAGTACGGCTGGACCGAGTGGGAGGACCCCGTCTCCGACGACAACCCGACCTCGCCC
AAGACCGACTCTGCCGGCGAGTCATCCTCTCCCAGCAGCCCGCTCTCCACCACCCCCAGC
AGCAGCACCGCCACCAGTCCCGTCAGCGGCGGCGGCGGCGGAGGAGGTTCCAGCCTCCAG
TCGAAGCGTGGCAAGCGCCAGCGGTCGGCCTCCTTCCGTCTGTCTTCCACGCGCTGGAGC
AAGGAGGAGCTGCTCTTCAAGATCACCAACGAGCTCCCGAGCGACAAGCTGGAGGGCATC
ATCATGATCGTCAATCCTTCCCTCGAATTCGGTGACACTGAGGACGAGGATCTGGAGTTT
GATATCAACGCCTTGGACGAAGCTACTCTCTACCGACTCGAGGATTATGTCCACGCGTGC
TTGATGGACACCAAGGACGACCAAGGCAACGAAAGCATGATGGACCACCACCACCACGTC
GGACGAGGAACCGAGATAATCACGATCACGCAGACGATAGAGGAGACGGTGACGACTTCG
CCCCGGAAGAGGACGCGAACGAAGGGCCAAGGCACCACGCGCAACTCTCGAAAGACCAAG
CCGACAACGACAAACGCCCAAGCCCAGACCCTCCCGAGTCCCGTCGCCGCGAAGAAGAGC
ACCACACGCGCCCAGAGGCGGAAAAACGCGTCGCCGACCAAGACGAAGAGCCCCGCCGCC
GCCACCACGTCGAAGAAGGCCGCCACGAAGACGACAACGACGCAAGTCATCGAAACGACG
ATCTGCCACTTCGTGGAGCAGGACGAGGACTTCAACTACATCCCGCACAGCCGAGGGGCC
AGACTTAACACGTTGGTGCCGGAGGCGTTCGAAGTGTTCCGAACGGAGCAGGTCATCAAG
GTGCAGAAGTCCATGTCGGACAGCGAGATGGAAGAGGAGGTGGACATTATCTAG
>g254-17093-20188
ATGCTGCGTTCCTCCAACAAGGTCGCCAGTACCCGAGGCATGCAGCGGGCATGCTTCGCC
TCTCGCACCACCATCTCTCATGGCAGCAAACAACTCCAAGTGCCCTCAGGTGGACGCCTC
TACTCGTCCACCACTTCCACGGCTGGCTCTGCCAGCCGCCAGTCCGCTTTCAGGTGGCCC
GTGGTGGCTGCCATCACCGCGGTGGGCGTTGGCGGTGGCCTCTATCTCCTCCAGGGACAC
GTGTTCGCTGCGGAGAGCCAACCCAAGCCCAAGCTCGTCATCCTCGGCTCCGGCTGGGGC
GCGCTGAGCGTGGTGCGAGAGCTGGACACGAGCAAGTACGACGTCACGATCGTGTCTCCG
CGCAACTATTTTCTCTTCACCCCGCTTCTTCCCTCGGTCACCGTGGGCACGCTCGAACCC
AAAGCCATCATCGAGCCCATCCGAAAGTACTGCCGCCGCAGCCACGCTGATGTTGACTAC
TTTGAAGCTGTGGCGACCGATGTGGATCCCACCAACAAAACGGTTTCTTGCCACGTGAGC
ACTCCCGGTCTCGATGACTCGGCGCGCGATTTCACCCTGCCGTACGACAAGCTGGTGGTC
GCTGTTGGTGCTATCAACAACACATTCGGCACGCCCGGTGTGGAGGAAAACTGTCTCTTC
CTCAAGGAGATTGATGATGCGATGGCCATCCGCAACAAGATGCTCGACTGCCTCGAGCTC
GCCTCTCTGCCCACCACATCAGAGGAGGAGAAGAAGAGACTTCTTCACTTCGTTGTGGTC
GGAGGTGGTCCCACTGGCGTGGAGGCTGCGGCCGAGCTCAGGGACTTTGTGCAGAGCAAC
GTGCACAAGTGGTTCCCCAAGCTGGAGCCTCACGTGTCGATCACCCTGGTCGAGCTGATG
GACCACATCCTCAGCACTTACGATGCCAAGATTTCCACTTACACCACGTCTCACTTCAAG
AATACCAACATTGACATCCGAACGAAGTCGCGCGTGGTCGCGGTCAAGCCCGGTGATGTG
ATCATTCAGAGGACCGACACCAAGGAGACCCAGCACATCCCGTACGGTCTGTGCATCTGG
TCGACCGGTATCGGCACCTCGCCGCTGATCAACAAGATCCGGGAGAAGCTGCCGCAGGAC
ATCCAGACCAACCGACGAGCCCTGCTGACCGACCAGTTCCTTCGAGTCAAGGGTGCTGAT
GGTATCTACGCTCTGGGCGACTGCGCCACGATCGCCCAAGAGGCGATGCTCGGCAAGCTT
AACGACCTCTTCAAGGAGGCCGACTTGAACAAGGACAATCAACTGCAGATCGAGGAGTTC
AGGTCGCTCATCGACAAGTACAAGAAGACGTACCCGCAGATCGATGTCTACGGCAAGAAG
GCCGAAGAGATCTTCCAGGAGGCCGATGTCGACAAGACCGGCGCCCTGTCGCTGGAGCAG
TTCGAGAACCTCGTCAAGAAGATCGACAGCAAGATCAAGCAGCTTCCTGCCACCGCTCAG
GTCGCCAGCCAGGAGGGCCAGTACTTGGGCAAGCTGCTGAACAGAGTCGCCAACAAGAGC
GTCGAGTTGGACACGGGTTTCCACTACAAGCACCTCGGATCGTTCTGTTTCATTGGCTCA
GAGCACGCTGTTGCCGAGTTTGCAGAGGGGCTGGTGCTGGAGGGCTTTGGTGCGTGGTGG
CTGTGGCGCTCGGTCTACCTCAGCAAGCAGTACAGCCTCCGAAACAAGCTCTACGTGGGC
GTCAACTGGCTCAAGACCTGGATCTTCGGCCGCGACATCACCAGGGCCTAA
>g254-128897-129908
ATGCCTAAGGAAACTAAGAAGGCCCCCGCCGCCACTAAGGCCGCCCCCGCCAAGGCCGCT
CCTGCCAAGAAGGCCGCCGCCCCCAAGAAGGACGTCGCCAAGGCCGCCCCTAAGAAGGAC
AAGGTCAACCGCGCCGCCAAGAGGGCCGCTGCCCCCGCCGACAAGAAGGCCGACAGCGCC
GTCGCCAAGAAGACCACCGGCCAGGCTGCCCAGGCTAGGTCCAAGGCCCTCAAGCTCCAG
AAGACCATCAAGAAGGGCGCCACCAAGCGTGTCCGCAAGGTCAGGACGAACGTGCACTTC
TTCCGCCCGAAGACTCTCCGCCTTCCCCGCAACCCCAAGTACCCCAGGAGGTCGGTTCCC
CGCACGAACAAGCTCGACCAGTTCCGCGTCCTCAGGCACCCCTTGACCACCGAGAGCGCC
ATGAAGAAGATTGAGGACAACAACACTCTCGTCTTCATCGTCGACCTCAAGGCCAACAAG
CGTCAGATCAAGGACGCCGTGAAGAAGATGTACGACATCACCGCCGAGAAGGTCAACACC
CTCGTGAGGCCCGACGGCAAGAAGAAGGCTTACGTCCGCCTCACCGGTGACTTTGACGCC
CTTGACATTGCTAACAGGATCGGTATCATCTAA
>g254-156436-158126
ATGTCGTGCTGTGGTGGATCGCCTGCTGCCCCTCCCCCGAGCCAAGCTCTTCCCTACAAG
TACCTCTTCAAATACATCATCGTCGGCGACACGGCTGTTGGCAAGTCGTGCCTTCTGCTC
CAGTTCACCGACAAGCGCTTCCAGCCTGTCCATGATTTGACGATTGGTGTCGAGTTCGGC
TCGAGGACTCTTACCATTGAGGACAACCAAGTCAAGCTCCAGATCTGGGATACGGCTGGT
CAAGAGAAGTTCAGATCGATTACCCGTTCCTATTACCGCGGCGCTGCTGGCGCTCTGCTC
GTCTACGATATTACCAGGCGTGAGACCTTCGAGCACCTTACGTCGTGGCTCGAGGACTGC
AGGAAGTACAGCAACAGCAACATTGTCATCATGCTGATCGGCAACAAGTGCGACTTGGAG
AGCAAGAGGCAGGTGAGCAAGGAGGAGGGCGCTGCCTTCGCCAAGGAGCACAACCTGCTC
TTCCTCGAGACCTCCGCCAAGACCGCTGAGAACGTCGAGCAGGCTTTCATCAACACGGCC
AGGACGATCTATGAGACCACGCGGGAGGGTGACATCGACTGGGAGCACTCGACCCCCAAG
TAA
>g254-159745-160888
ATGTCCGTTAGGGAGACCTTCGAACACACCTTCAACCATTCCTGGGAGGATTGCGCCATC
GCCAGCTGGAAGAAGTGGCCGAATCCTCGTCGCCCCGACGTACTGTGCGTAGACATCATC
AACAAGGAATTCGATGAAGCGACGGGCGTGCTCAAGGCCACGCGATTGATGATGCTCAAG
AGCTGGGTCCCCTCGTGGATGCCTCTTGCCGGGAACAACGTTTGCTTCTTCCTCGAGGAA
TCGATCACGGATCCGAAGAACAAGAGACTGATACTGAAGGGCAAGAATCTCACCTTCCAA
AATCTCGCTGAGATGGAGGAGACCTGCATCTACACCGAAGACGAGAACGGCACCTTCTTC
GAACAGGAGGGCGCCGTCACCGCCTACACCTTCGGTCTTGCCAGACGCATGGAAAAGTTT
TGCCTCGACAGATTCCGCAATGCAGCCATCCAGGGACGAGACATAATGGAGCAGACCATC
CGACGGATTAAGGAGGAAGGCTTCCCCATGGGCATCACGATGAACCTCGAGAAGCCGCCC
ATGCTGACATCCCAGATGCGCACTTGA
>g254-211539-213768
ATGTCTGACGCCGCGATTACCCAGCTCCTGAGCAGGCTCGAAGCCGTTGCCGCTCGCCTC
GAGTCGGTCGAGAAGCAGCTCCAGTCGGGCGGTGGTGCCGGTGGTGCCGCTTCGTCGGCC
CCGGCTGCCTCTGGTGAGGTGACCAAGGCCGTGTCCGAATTCGACAACCTGGTGTCCACT
TACATCGACGAGTACGTGAACGTCTCGGCCAAGATCGACCCCCTGGTCAAGCAGCAGGCC
GACCTTGTCAAGCAGGCCGTCCTGGCCCAGAGGGATCTCCTCTACAAGGCCTCGCAGAGC
AAGAAGCCCGACGCCACCACCTTCGGCAACCTGCTGAAGCCCACCTCTGACCTCATGGGC
AAGATCGTCGCCATCAGGGATGCCAACCGTGCCAGCAAGGTCTTCGGTCATCTCTCCACC
ATCAGCGAGGGTATCACTGCTCTCGGTTGGGTTGCCAGCCCCCCTACCCCTGGTCCCTTC
GTTGATGAGGCCCGCGCCTCGTCTGAGTTCTACTCGAACAAGCTGCTCATGCAGTACCGC
AAGGACGAGTCGGCCGAGGGTAAGACCCAGGTCGCGTGGGTCACTGCCTGGAACACCTTC
CTCAAGGAGCTCCGCGCTTTCATCAAGACCTACCACACTACCGACCTCACCTGGAACCCT
CAGGGTGGTGATGTGTCCAAGGTGGCTTCCGCTGCTGCGCCCCCCGCCAGTTCGGGTGGT
GCCCCCCCTCCCCCCGCTGCCGGCCCCCCTCCTCCCCCCGTCGTCGACCTCTCCGCCGCC
TCCAGCGGTGGTGGTGCCCACGCTGCTCTCTTCGCTGAGATCAACGCCGTCAAGGAGCGT
CAGGCTGGCGGCAAGACTGCTGGTCTTAAGCACGTGACCAAGGACATGAAGACCAAGAAC
ATGGAGAAGACCGCCGCCCCCGTTCCCGCCGCTGCCCCCGTCCGCAAGCCCGCCGCTGCC
GCTGCGCCCAAGCTTGGCCCGCCCAAGTTCGAGCTCGATGGCCAGAAGTGGAAGATTGAG
AACCAAGTCGGCAACAAGAACATCGTCATCGAGAACCCGGAGGTCAGGCAGACTGTCTAC
ATCTACAAGTGCCACGACTCTGTCATCCAGATCAAGGGCAAGGTCAACTCGATCATGCTT
GACTCGTGCAAGAAGACCGGTCTCGTCTTCGAGAACGCCATCTCCGTGGCCGAGGTCGTG
AACTGCGCCAGCGTCCAGGTGCAGGTGACCGGCAAGGTCCCCTCCGTCGCCATCGACAAG
ACCTCTGGTTGCCAGGTCTTCCTGTCTGCCGAGGGCTTCGACACTGAGATCGTCACCTCC
AAGTCCGACGAGATGAACGTCGTGCTGCCCGGCCTCAACCCCAACGACGACATCATCGAG
ATCCCCATCCCCGAGCAGTTCCGCACGCTCATCAAGGACCGCAAGCTCGTCACTGAGGCT
GTCGCCCACTCGGGATAA
>g254-213866-215830
ATGTCGTCCATCCGCTCGCGCGCCCCCTCGATGCTGCTCCCCACCAAGCTCGGTACCTCC
GGTCGTCTGGGTGCCGCTGCCCGCCGCCTCTCTTCCGGCCAGCCCCAGGCTGCTCGCGAT
GTTGAGAAGCGCCTTCGTCTGCCCGAGTACCTTCTCAATGTCCCCCCGACGCAGGTCACT
GCCCTCCCCAACAAGTTCCGCGTTGCTTCCGAGCACAAGCACGGCGAGACCGCCACCGTG
GGCGTGTGGATCGATGCCGGCAGCGTGTGGGAGACTGCCGAGAACAATGGTGTGGCCCAT
TTCCTCGAGCATCTGGCCTTCAAGGGCACCAAGAACCGCACCAAGGAGCAGATCGAGGTC
GAGATCGAGAACATGGGCGGCCAGCTCAACGCCTACACGTCTCGCGAGCAGACTGTGTAC
CACGCTCACGTGTTCAAGAAGGACGTGCCCAAGGCTGTCGAGATTATCTCCGACATCATC
CAGAACTCCAACCTCAAGGAGGACGATGTTGAGCGCGAGCGTGGCGTCATCCTGAGGGAG
ATGGAGGAGGTGGAGAGCCAGACCGAGGAGGTCATTTTCGATCACCTGCATTCCATCGCC
TTCCAGAACACCTCGCTGGGCTACACCATTCTCGGCCCGGAGAAGAACATCAAGAAGATC
AAGAGGGAGGACCTCGTCTCCTACGTCGGCAAGCACTACACCGCCCCCCGCATGGTCCTT
TCCGCCGCCGGTGCCGTCGACCACGACGAGCTCGTCAAGCTGGCCGAGAAGCACTTCAGC
GGCCTCTCGTCGGAAACCAACGTCGACTACTCCAACCGCGAGAAGCTCTTTGACTTCACC
GGTTCGATGGTGCAGGTGAGGGACACCAGCATCCCGCTCGTCCACACCACCGTGGCCGCC
AAGAGCGTCGGCTGGTCCGACCCGGACTACTTCACCTTCCTCGTGCTCCAGCAGCTCGTC
GGCAGCTGGGACCGTTCGCTCGGCGGCGCCAAGAACCTGAGCTCCAACCTGGCCGAGACC
TTCGCCACCGAGGAGCTGGCCCACAGCCTCATGTCCTTCAACACCTGCTACCACGAGACC
GGCCTCTTCGGCGCCTACTTCGTGGGCGAGATGGAGCGCACCAGCGACGCCATCTTCGAG
GTGCTGCGCGAGTGGGTGCGCATCGGCTCCGGCGTGTCCGAGGTCGAGGTCGAGCGGGCC
AAGAACAAGCTCAAGTCCACCTACCTCATGCAGCTCGACGGCACCCAGGCCGTGGCCGAG
GACATCGGTCGGCAGCTGCTCACGCTCGGCCGCCGCATGCCCGCCGCCGAGGCCTTCATG
AGGATCGACGCCATCGACGCCAAGAAGGTGCGCGAGGTCGCCTACACCTACCTCAACGAC
GTCGACGTCGCCGTTGCCGCCGTCGGCTCAGTCGACTCTGGCCTCTTCCCCGACTACAAC
GTTCTCCGAGGCTGGACCTACTGGAACAGGCTCTAA
>g254-222986-224468
ATGGATATGAACACGATCTACACGCAGCAGCCCGATGACAACGAGAAGAAGATGGAGCAG
GACTTCTCTGAGGAGGTGAAGGTCAAATTGCCTGAGCTGCGCGAGCAGGCCAAGACGCAG
CTGGCCGAGGGCGTCGATCGACTGCTCGCCCTCGAGAAGCAGACTCGCCAGGGTGGCGAT
GAGCCCTCCACCACCAAGGTCGTTCGCTGCATCGTCGAGGTGTGCTTGGAGGCGCGCGAT
TGGCCCCGATTGAAGGAGGTTCTCGTCGTGATCTCGAAGCGCCGCCAGCAATTCAGGAAC
ACAATTCAGACGTCGGTGCAGGTGACGATGGACGCCTTCCCCTCGCTCCCCGACAAGGCC
ACGAAGCTCGATCTCATCGATACGCTGCGCGCGATCACCGAGGGCAAGATCTTCGTCGAA
GTCGAGCGTGCGCGCCTTACGCGTATGCTGGCGCAGATGAAGGAGGAGGAGGGCAACGTG
AACGAGGCTGCCGAGCTGCTCCAGGAGGTGCAGGTGGAGAGCTTCGGCACGATGGACGCG
CGGGAGAAGCTCGACTTCATCCTTGAGCAGATCCGACTCTGCCTGGCCAAGGGCGACTTC
GTGCGCGCGCAGATCATCTCACGCAAGGTCACCAACAAGGCCCTCTCGAAGCCTGAGTTC
CAGGAGATCAAGGTGAGCTACCACCTGCTCATGGTCAAGTTCCACACGCACCAGAAGGAC
TACCTCAACATCGCCCGCTCGCACTGGGCCATCTACGACACGCCCGTCGTGCTCGCCGAC
AAGGCCCGCTGGCAGCCGGCCCTCACCCTCGCCGCCGTCAACGCCGCGCTGGCGCCCTAC
GGCAACGAACAGTACGACCTCCTCAACCGCATCTTCATCGACAAGCGCCTCGGCGAGCTC
CCGCAGTACAAGAAGCTCCTCAAGTACTTCACCACCACCGAGCTCATCCGCTGGCCCACG
CTCCTCGGCGAGTACAAGGGCGCCCTCACCCAGCTCCCCTCGTTCGCCGAGAACTCTGAG
ACGCTGCTCAAGGACCTCCAGGCCCGCGTCGTGGAGCACAACATCCGCGTGATCGCCCAG
TACTACGAGCGCATCGCCACGCCCCGCTTCGCCCAGCTGCTCGACCTGCCCGAGTCCGAG
CTGGAGCGGTTCATCTCCGACATGGTCTCCAACGGCGTCGTCTTTGCCAAGATCGATCGG
CCGCGCGCCGTCGTCTCCTTCATCAAGCGCAAGGAGCCCAGCGACGTCCTCAACGAGTAC
TCGCACAACATCTCCGACCTCCTCAACCTCCTCGAGAAGACGTGTCACCTCATCCACCGC
GAGAACATGGTGCACGGTCTCTAA
>g254-236816-239220
ATGAGCAAGCAGAGGATCTGCTACTTCTACGATGGGGATGTGGGCAACTACTACTACGGA
AACGGACACCCGATGAAGCCGCACCGCATAAGGATGACCCACAACCTGCTCCTCAACTAT
GGCCTCTACAAGCAGATGAAGATCTACAGGCCCAGGCACGCCACAAAGCAGGAGATGGCG
CAATTCCACGCCGAAGACTACGTCAAATTCCTAAGATTGATCACTCCCGACAACATGAAT
GAGTACACCAAGCAACTGCAAAGATTCAACGTGGGCGAAGATTGTCCTGTCTTTGATGGA
ATGTATCAATTCTGCCAGATCTCTTCTGGTGGTTCAATCGGTGGCGCTGTGAAGCTCAAC
CACGGCGAGTCCGATATCGCCATCAACTGGGCTGGCGGCCTTCACCACGCCAAGAAGTCG
GAGGCCTCTGGCTTCTGCTACATCAACGACATCGTGCTGGCCATCCTCGAACTGCTCAAG
TACCACGCGAGAGTCCTGTACATTGACATTGACATTCATCACGGCGACGGTGTCGAAGAG
GCCTTCTACACCACCGACCGCGTGATGACCGTCTCCTTCCACAAGTACGGAGAGTACTTC
CCTGGCACAGGCGACATCAGGGATATTGGAGCACAGAAGGGCAAGTACTATTCGGTCAAC
TTCCCGCTGAGGGATGGCATCGACGACGAGAGCTACGAGAACATCTTCAAGCCCATCATC
CAGAAGGTGATGGACTGGTACCGGCCGGGAGCGGTCGTGCTCCAGTGCGGCGCGGACTCA
CTATCGGGCGATCGTCTGGGCTGCTTCAACCTCTCGCTCAAGGGCCACGGCGCGTGCGTC
GAATTTGTACAGAGCTTTGGGCTGCCCCTCCTAGTCCTTGGTGGCGGCGGTTACACCATC
CGTAACGTCGCTCGTTGCTGGACCTACGAGACTGCCCTCCTCTTGAAGAAGGACATCAAT
GACGAGCTGCCCTTCAACGACTACTTGGAGTACTACGGCCCGGACTTCAGACTTCACCTG
TCGCCCTCGAACATGGAGAACCTCAACGATCCCAAGTACCTCGAGAAGACCAAGATCAAG
CTGATCGAAAACCTCAGGAGTGTTCAAGGCGCGCCGGGAGTGTCAATGAACGAGATCCCG
CCTGACACTTACCTCGGCTCGGACGACGAGGACGAAGAGGACCCCGATGCCCGCATCAGC
GAGCGTCACAGGGACCGTCGCGTTGCGCATGACGCCGAGCTTTCCGACTCGGACGAGGAA
GACGCAGGGCGACGCTTCGACGACGTTGCCTTTGACGACGACGATGAGGATGAAGAGGAG
GAGGACTGA
>g254-290431-292598
ATGACGACCAGCGGCGATGACCTGTCCACCCTGCGCACGATGAGGGATGTGTTCGACTAC
TGCCAGCGGAAGGGCCTGTACTCGGTGGCCCAGGGCATGATCGAGCTGCCGCCCCCGCGC
GCCCTGCGCGAGATTGTCGCGGCCGACGTGCTCAAGGACGAGGCCCCGTCCTCGGACATC
CACCAGTACCGCTCGCGTATGGGCGAACGCGACTACCTCAACGCCCTCCGCACGCTCCTC
AAGGACCACTACGCGACCGATGTGCCCGAGGGTTCCATCTTGGCCACCTCCGGAGTGACG
GGCGCGATCGTGGCGGCGCTGATGGTGCTGCGGAAGCAGGGCAAGTCGCGCATGGCGGTG
ATCGAGCCGTACTACACCTACCACTCCCGCCAGGTCGAGGAGGTCTTCCAGAAGCTGCCC
GAGGGCATCCCGTCCCACCTCGATTGGTCTCCCGACTTTGACGCGATCGAGAAGGCCCTG
AAGGGCGGCGTGGAGGGCATCATCATCGCCAACCCCAACAACCCCACCGGCCGCGTGTGG
GCCAAGGAGGAGCTGCAGAAGCTTGTGGCGCTGACCAAGCAGTACGGCGCTTCGCTCATC
TTGGATGAGATCTACTGCGACATGGTCTTCGCCGGCAACAAGCACTACTCTCCCATCCAG
GACTCGCTCGAGGAGCACGTGTTTGTATGCCGCGGCTACTCCAAGACGCTCGGCGCGCAG
AGCTGGCGGTTGGGCTATGCCGTCTCCCACCCGGAGACCATCAAGTCCCTCATGACCCAC
CACGATCCCATCTACATCAGTGTGTCGTGGCAGCAGCACAGCTTGGCCCAGTACTTGAAC
AAGCAGTACAGCGATTACGTTAAGCACATCAACGAAATCAATGCCCTTCTCCAGACCAAC
TACGAGATTCTCGCCCCGGCCTTCAAGGACGCGCTAGGCTGGGAGCCGATTGCTCCTCAG
GGCAGCATGTACGGCATGTTCAAGCACACCGAAGCCAGCGACATCGAGGCCCTTCAGAAG
GGCCTGCGTGCCGGCGTGGGTGTGGCTCCGGGTTCGATGTTCTACGTGGATAACCGGGCC
AACTCCGGCTACATCCGCATCCATGTGGGCATCTCCACTGAGAAGGCCAAGAAGATTGCT
GAGACCCTTCGTGCCAACAAGCAGTAA
>g254-319025-319938
ATGTCCGGCATTGCTGTCTCTGACGATTGCGTCCAGAAGTTCAACGAGCTCAAGCTCGGC
CACCAGCACCGTTATGTCACCTTCAAGATGAACGCCTCCAACACCGAGGTGGTCGTCGAG
CACGTCGGTGGCCCCAACGCCACCTACGAGGATTTCAAGTCGCAGCTCCCCGAGAGGGAC
TGCCGCTATGCCATCTTCGACTACGAGTTCCAGGTTGACGGCGGTCAGAGGAACAAGATC
ACCTTCATCCTCTGGGCTCCCGACTCTGCCCCCATCAAGTCCAAGATGATGTACACCTCC
ACCAAGGACTCCATCAAGAAGAAGCTCGTCGGCATTCAGGTTGAGGTCCAGGCCACCGAC
GCCGCTGAGATCTCCGAGGATGCCGTCTCCGAGAGGGCCAAGAAGGACGTCAAATAG
>g254-323982-326008
ATGGCGCGCGTCCACTACGTTCTCGCGGTCCTCGCCGCTCTGGCCGTCCTCTTCATCGCC
GCTGAGGCTGGCACCATGACTGCCGAGCAGCAGTTCCGTCAGTTTGCCGCCCAGTACGGC
AAGAGCTACGCCTCGGAGGAGTTCGGCGAGAGGCTCAGGATCTTCAGGGATAACCTGGAT
CGCATCGATGCCCTCAACTCGGCCAACACCGGCGCCCGCTACGGCGTCAACAAGTTCGCC
GATCTCACCCCCAAGGAGTTCAAGGCTACGTACCTCAAGGGTGCGCGCAGCGCCGGTCAG
AAGAAGGCCGCCGCTACCGCCAAGCTCGACATGACCGGTCCCCTCCCCTCCCAGTTCGAC
TGGCGCGACAAGGGCGCCGTCACCCCCACCAAGGACCAGGGCCAGTGCGGTTCCTGCTGG
GCGTTCAGCGTGACTGAGGCGATTGAGAGCCAGTGGTTCCTCTCCGGCCGCAAGCTGGTC
AGCCTCGCGCCCCAGCAGATCGTCGACTGCGACCAGGGCAACGGCGACTACGGCTGTGAC
GGTGGCGACCCTCCCACGGCCTACGAGTACGTGATCAAGGCCGGCGGTCTCGACACCGAG
GAGTCCTACCCCTACACTGCCGAGGACGGTCAGTGTGCCTTCAAGCCCTCTGCCGTGGGT
GCCAAGATCTCCAACTGGACCTACATCACCACCACCAAGAACGAGACCGAGATGCAGTAC
GGTCTTGCCAGCAGGGGACCCCTCTCCATCTGCGTTGATGCCTCCTCGTGGCAGTACTAC
ATCGGTGGCGTCATCACCAGCCTGTGCGAAGACAGCCTCGACCACTGCGTCATGATCACT
GGCTACTCGGTCCAGGAGGGCTGGGACTTCATGAAGTACGACGTCTGGAACATCCGTAAC
TCGTGGGGCGAGGATTGGGGCTATGGTGGCTACCTCTACGTCCAGCGCGGCTCCAACCTC
TGCGGTGTCGGCGATGAGGTGACCATTCCCCTCGTGTAA
>g254-343986-345220
ATGGACCTCAGTCACCTGATTCGCTCACAAGTGGTGCTGTCCGCTGCCTTCTTCCCTCCA
CCTCCCGCACCCTCGCCCCAGGCTGAGAACCGTAGGAGGCTGACAGGGGCCAGCCCGTCG
CTGGTCGACCTCGCCGCCAAGGTGGTGGCCGTCAACTTTGAACTGTACCAGGACTGCTGG
GTCCCTAAACCGTTGCATGTGCACCTCCAAAAGTTCATGGAGCCGAAAGCCCAGCGCCAG
ATGCTCGATGCTGTGAAGAAGTACCACCCCAACGGGAACGTCAAGTCCAACAAGGAGTTC
GACGACGAGGGCCAACTGCACGGCAAGATGGAGGAGTGGTATGCCAACGGCCACAAATGT
CGTGAGGAGTGCTACGAGCACGGGGTGCTGAACGGAGAGGCGCGCTGGTGGCACGAAGCG
GGCGGGTTGTGGACCCAGCAGCACTTCCTCAACGGGCACAGGCACGGCAAGTGCGTGTGG
TACTACCCAAGCGGGAAGCCGCACAGGATTGTGAACTACAAGAACGGGCTCAACTGA
>g254-519206-519872
ATGAAGCTCGTCCGCTTCTTGATGAAATTGTCCAACGAAACGGTGACCATTGAGCTGAAG
AATGGCACGATCGTCCACGGCACCATCACCGGCGTCGACATCAGCATGAACACCCATCTC
AAGGCCGTGAAATTGACCCTCAAGGGCAAGAACCCCATTAACCTCGACACGCTCAGCATC
AGGGGCAACAACATCAGATACTACCTTCTTCCCGACACCTTGAACCTCGACACCCTCCTG
GTCGACGATACGCCGCGGCCCAAGCCCACCAAGAAGGTCGGCGAGGCGGGTGCGGCTGGA
CGAGGGCGCGGCAGGGGTGCCCCGAGGGGACGTGGCGGCGGCAGGGGAGGGCCGCGCGGT
GCCGGTGGCCCCTCGAGAGGACGCGGCAGGCGCTAA
>g254-582947-585428
ATGGCGGACGCAACCACCCTCGCAGCAATGTTGCAAAAGGCAAACGATGCCACGCCCGAG
GAGGCCAAGAAGATCTATCAGAAGATCATCGCAGAGGGACAAGGTCAGGCACAGGGAGAA
CAAGTGAACCGTGTGAAGGAGCAGGCCATCTACAAGCTGGGTGAGGTCTATGCCAAGCTG
GGAGAGGCCGACAGCTTGCGCTCGCTGCTCACGCAGATTCGGCCCTTCTTCGATGCCATT
GCCAAAGCTAAGACCGCCAAGATTGTGCGCACGTTGATCGATCTTGTGGCGCGCATCCCC
AACACGATCAACCTTCAGATCGAGCTCTGCCAGGAATCCATCGAGTGGGCCAAGGGCGAG
CAGCGCACCTTCCTCCGCCAACGCATCGAAGCCCGCCTCGCCTCCCTATATTTGGAGACC
GACGGCTACACGCAAGCGCTGCGCTTGCTGACGACGCTGCTGCGCGAGGTGAAGCGATTC
GACGACAAGCCTCTGCTGGTGGAGATCCAGCTGACGGAGAGCAAGGTGCAGCACAAGCTG
CGCAACCTGTCCAAGTCGCGGGCCTCGCTCACGGCCGCCCGCACGGCGGCCAACGCCATC
TACTGCCCGCCGCTGCTTCAGGCCGAGATCGACACTATGTCCGGCACGCTGCACGCCGAG
GAGAAGGACTACAAGACCGCCTACTCGTACTTTTACGAGGCCTTCGAAGGCTACAACTCG
ATCACAGCCGAGAGCGCGTTCGTGGCCCGAGGCGCCATCTCGGCGCTCAAGTACATGCTC
CTGTGCAAGATCATGCTCAACAGCGTGTCCGACGTCAACCAGATCATCTCCGGCAAGATG
GGCCTCAAGTACGCCGGCACCGAGATGGAGGCTATGCGCGCCGTCGCCAACGCCCACAAG
CAGCGCTCCCTTCAGGCCTTCTCCGAGGCCAAGACCACATACGCCAACGAGCTCACCAAG
GACCCAATCATTGACACACACCTGTCGGAGCTTGAGGACACACTGCTCGAGCAGAACTTG
TGCCGCATCATCGAGCCCTTCTCGTGCGTCGAAATCGCCCACGTGGCCAAGATCATCGAC
CTCCCCATCCACAGCGTGGAGAAGAAGCTGTCTCAGATGATTTTGGATAAGAAGCTGCTC
GGTGTGCTCGACCAGGGCGCCGGCACGCTCCTCATCTTCGACGAGCCGACCGCTGACCGC
ACGTATCCCGCGGCGTTGGAGACGGTGCAGGGTATGAACAAGGTTGTGGACAGCCTCTTC
GAGAAGTGGGCCAAGGTGGGCGCCGACTTTGTATAA
>g254-645516-647398
ATGGCGGACCACATTTCGCTCAAGGTAAAGCTCGATGAGGACATCAGGCGGTTCAGCGTT
CCCAAGGGGATCACCTTTGCCGAGCTGCACAGCCTCGTTTCCAAGCACTTCGTGCTCGCA
CCCGAAAACGTGCAGCTCAAATACATCGACGACGAGGAAGAGCTTATCACCCTCGGTTCG
GACCTTGAGCTTCAAGAAGCAAAGCGCCTGCAGCCCACGGTGCTGCGCCTCAACGTTTAC
GACGCCAAGTCGAAGCGGCATACTTCCGATCAGCCCACCGCCCAGTACGAGGCTGTCTTC
GTCCGAGATGTGACCGTGCCCGACGGCACCAACGTAACCGCGAAGGTCAAGTTCCAGAAG
ACGTGGTCTGTCAAGAACACGAGCTCCGCACCCTGGCCCAAGGGCGTGGTCTTGAAGATG
GTCGACCCCAAGGACGACCAGCTGGTCGTGCTCGGCGTAGGCGCCATCAACCGTGTGGTC
GGCCCCGGCGAGGAAACCGAGATCGGCGTGCACCTGCAGGCGCCCTCGCGCCCGGGGCGC
TGCGTCCAGTACTGGCGCCTTTTCACCGAGGATGGCGTGGCCTTTGGCAGCCGTCTGTGG
GTGGACATCACCGTGACCCAGGACAACGCGGCCGCTATGGCTGAGGCCGAGAGGGCTAAG
GCTATGGCCGAGGCCGAGGAAGAGGAACGCCGCCGCTATGAGGAGGCAAGGAGACTTGCC
GAGGAGGAGGAATTTAGGAGGAGGCAAGAGGAAGAAGACGCGAAGAGGAAGCAGCAGCAG
GAGGAAGAGGAGAGACAGCGCCGCGAGGAAGAGGAGAGGTTGGCGCAGCTCGAGAGGCAA
CTCGCCAAGGAGGAGGCGGAGCTCAGGCAAAAGCTCGAGCAGGAGGAGTGCGAGAAGAGG
ATGAAGCAGGAGGCGGAGGCCGAGGCGCTGAGGATGAAGCAAGAGATGGAAAAGGAGATG
CTGAGGCAGAAGGAGGCCGCGAGGCTGCAAGAAGAGGCCGAGGAGAAGCAGAGGCGTCTG
TACAGCAAGTACCCCGGTCAGCTCAACGCCTTGGCCGAGATGGGGTTCACCAACGTGGAG
CACAACGTGAAGCTGCTCGACAAGCACAAGGGCAGCCTCGACCTGGCCCTCGAAGCTCTT
CTCTCTGGTCAGTGA
>g281-44897-46248
ATGAAGGCCATCGCGTCGCTCCTCACCATCGCCTTCCTGCTGTGCGGCTTCGCCGCCATC
ACCCCCGCCTCGGCCCAGGAGACCTGCCCTCTGTGCCAGTTCGCCGTCCAGTACATCGAT
GGCTACCTGCAGCAGAACTACACCCAGGCTCAGATCATCAAGCAGCTCGAGGTTGTGTGC
GCTCTGTCGCCCGAGCCCTTCCGTGACCAGTGCGACTCGTTCGTCGAGTACTACGTCCCC
GTCCTGATCAACTACATCATCAAGTACGAGGATCCCCAGAACGCGTGCCAGCAGCTTGGT
CTCTGCACCTCATTCGCCGCTGAGCCCGAGATCGTTGTTGCCGATCTTGAGAAGTTCCCC
GAGGTTGTCGAGGTCGTTGAGGCCAGCCCCGAGGATTGCCAGATCTGCAAGATGCTCGTC
GGTTTCATCGAGTCCTACGTGCAGGCCAACCAGACCATCACCCAGATCGAGTCTCTCCTC
GGTCGCGTGTGCCGTCTGACCCCCTTCGCCTCGCAGTGCGTCGTGTTCGTGGACACCTAC
ACCCCCCTCATCGTCCAGTACATCCAGGCCAACGAGGATCCCCAGACCGTGTGCCAGCAG
ATTGGCGTCTGCTCTTCGCAGGCCGTCCGTCAGGTCGCCGCCGTCAAGTTCAACTGA
>g281-48947-49998
ATGCCTAAGCACAACAATCTGATCCCCAACCAGCACTTCCACAAGGCGTGGCAAAACCAC
GTCAAGACGTGGTTCAACCAGCCCGCCAAGAAGAAGAGCAGGAGGGTTGCCCGCGCCGCC
AAGGCTGCCGCCATCTTCCCCAGGCCCGTCCAGGGTCCGCTCAGGCCCGTGGTCCACTGC
CCCACTATCAAGTACAACACCAAGCTCAGGCTCGGTCGTGGCTTCACGCTTGAGGAGCTC
AGGGGTGCTGGCATCACCAAGAGGTTCGCCCAGACCATCGGTATCTCGGTCGACCACAGG
AGGAAGAACAAGTCCGAGGGTCCCCTGGCCGACAACATCAACCGCCTGAAGCAGTACAAG
GCTCAGCTCGTCCTCTGGCCCAAGAGGAAGGAGTCCAAGAAGCACCCCAGGAAGGCCGAC
GAGGCCGCCCCCGAGGACAGGGAGAAGGCCACCCAGCTCACCGGCGCCGTCCTTCCCGTC
AAGCAGAAGGAGGTCCGCGTCAAGGCTCAGGTTGTGGAGCCCATGGGCAAGGGATCTGCT
TTCGTCACCCTCCGCCGTGCTCGCGCCGACGCCAAGTACATCGGCATCAGGAAGAAGAAG
GCCCTCGCCAGGGAGGAGAAGGCCAAGCTGAAGAAGAAGGACTAA
>g284-255306-257683
ATGAGCACCAACATGTGGGCCAGAGATCGTCAGGAGTCACGCAAGAAGGAGTTCAAGGCC
GGTGTCTCGGCCGAGGATTCCAGGCGCAAGCGCGAAGATAACGTGGTCCGTATCAGGAAG
AACAAGAGGGAGGAGAGCCTCGCCAAGAAGAGGAACCTCGGCCAGGCCTCTGCTCCCCGC
AGCAAGCACCACGATGCCTCGATCGCCCAGAAGCTCGAGAATCTGCCCAAGATGGTCGCC
GGCGTGATGTCCGAGGACCCCCAGCAGCAGCTCGAGTGCACCACCCAGTTCAGGAAGCTC
CTCTCGATTGAGCGCAACCCCCCCATCGAGGAGGTCATCGCCACCGGAGTCGTTCCCCGC
TTCGTCCAGTTCCTCACGCACGACCACAACCCGCAGCTCCAGTTCGAAGCCGCCTGGGCT
CTTACCAACATCGCCTCGGGTTCCTCGGAGCAGACCAGGATCGTCATCGAGAAGGGCGCC
GTCCCCATCTTTGTCCGTCTCCTCTCGTCCAACAACGACGATGTCCGTGAGCAGGCTGTG
TGGGCTCTCGGTAACATTGCTGGCGATTCTCCCCAATGCCGCGACTACGTCCTTCAGCAC
GGCGCCATGGGTCCCCTCCTGCAGAACCTGATGGAGTCGTCCAAGCTCTCCATGCTCAGG
AACGCCACCTGGACCCTCTCCAACTTCTGCCGCGGCAAGCCCCAGCCGCCCTTCGAGCTC
GTCAGCCCCGCTCTCCCCACCCTCGCTCGCCTCATCTACTCGACCGACGAGGAGGTCCTC
ACCGATGCCTGCTGGGCTCTGTCGTACCTGTCCGATGGTAGCAACGACAAGATCCAGGCT
GTCATCGAGGCTGGTGTCTGCCGTCGCATGGTGGAGCTCCTCATGCACACCTCGTACAGC
GTCAAGACCCCTGCCCTCAGGACCGTCGGCAACATCGTCACGGGTGATGACCTCCAGACC
CAGATCGTCATCAACGTCTCGGCCCTTCCCTGCCTTCTGTCGCTCCTGAACAGCCCCAAG
AAGGGAATCAAGAAGGAGGCTTGCTGGACCATCTCCAACATCACCGCCGGCAACAAGGCC
CAGATCCAGGTCGTCGTCGACGCCAACATCATCCCGCCCCTCATCAATCTCTTGGCCAAC
GCCGAATTCGACATCAAGAAGGAGGCCGCCTGGGCCATCTCCAACGCCACCAGTGGTGGA
TCCCCCGAGCAGATCCGCTACCTCGTTGCCCAGGGCTGCATCAAGCCCCTCTGCGACCTG
CTCACCGCCAGCGACGCCCGAGTCATCACGGTGGCCCTCGAGGGTCTCGAGAACATCCTC
AAGGTTGGCGAGTCTGATGCCAAGGCCTCGGGTGCCCGCAACCAGTACGCTGACTATATC
GAGGAGGCTGAGGGTCTGGACAAGATCGAGCAGCTGCAGGTTCACCAGAACATCGACATT
TACGAGAAGGCCGTCAAGATCCTCGAGAACTACTTCGCCGCCGAGGAGGATGACCAGAAC
CTCGCACCCAACGTCGACCCCAACCAGACTGCCTTCACCTTCGGCGCGCCCAGCGGTCTC
CCCAAGGAGGGCTTCAACTTCTAA
>g284-413416-415650
ATGGCGACCGAGAACGAAACGGCTTTGATTGACCTCACGCCGGCGCAGCTGGAATCGTGG
AAGCGCGACGGCTTCCTCTACATCAAGGCCCAGGACTTCTGGACGCCCCAGCAAATCGAA
AATCTCAAGAAGTGGACCGCTGATGTGGAGACGTGGTCCGAGACGCCCGGCAAGTGGATG
ATGTACTTCTCCAAGTCGGTGCGCCAGGACGACGAGGGCCGTCGGCTGCTCCACCGGCTC
GAGAACTTCTTCGACTTCCACGCGGGCTTCAACGAGGCCTTCAACGGCGAGCGCTTCCTC
CACCTCGTCACCCAGCTCTTCAACGGCGACCGAGCCATCCTCCACAAGGAGAAGATCAAC
TTCAAGTACCCCGGCGGCGAGGGCTTCGAGCCTCACCAGGATCACGCCGCGGGCTGGTGG
ATGTACGGCCAGAGCCTGCACATCTCGATCATGGTCAACATCGATCAGGCCACCAAGGAG
AACGGCTGCCTCGAGGTGGTGGCGGGCGAGCACAACAAGGGCCTCCTCGGTCCCGAGTTC
CAGGCCGTGCCGCAGGAGCTCGTCGACAAGTACACATGGATTCCTCTGGAAACCAATCCG
GGCGATATCGTCTTCTTTGATTCCTACGTTCCTCACCGCTCGGCGCCCAACCTCAGCGAC
AAGCCCCGCCGCGTGCTCTACGTGACCTACTCCAAGGAGAAGGAGGGCGACTACAGGAGC
AAGTACTACGAGGACAAGAGGCAGTCGTTCCCGCCCGACTGCGAGAGGGAGGAGGGCAAG
GACTACTCCTACAAGATCTAA
>g284-445523-446158
ATGAGCAAGAAGCACGATGATCTTCCTGACCAGGGCGGCTCGCTCGACTACCGCGGCAAT
TACGTGCCCTTCTACCCCAACCCCGAGGAGAATCTCAACCTCTGGGAGCGCGTGATGCGG
TACCCGTGGGTGGCCCTCGGCGCCTTCTCGACGGCGGGCATCCTCGGTGCCGGCCTGTGG
ACCCTCAAGACGGGCAACAGCGCCCTCGGCCAGAAGCTCATGCGGGCGCGCGTAGCGGCA
CAATTCACGACCGTAGTGCTCCTCCTGGGCGCGTCGGGCGTGATCACCCTCAACAGCCTC
AACAGCGACAAGCCCAAGCCCAAGGCTGTCGCCGCGCCTATCTCCTCCTCTTCATCTTCG
ACATCCGAGACGCACTAA
>g284-677684-678888
ATGGCAACTGACAGCGGCTGGCTCGGCGAATTGAACCCCTACACTTGGGCCGATCTCGGC
ATTGGTCTCACTATTGGCCTCTCCGTTGCCGGTGCCGCCTGGGGTATCTTCATCACTGGC
ACCAGCTTGCTGGGTGCTGCCGTCAAGGCTCCCAGGATCCGATCGAAGAATTTGATCAGT
ATCATTTTCTGCGAGGCCGTTGCCATCTACGGTATCATTATTGCCATCATCTTCCAGGGC
AAGCTTGAGGACTTCGGCGAGACTGCCAAGGACCCCGATTTCCATGCCGCGTTTGCCCTC
TTCTGGGGTGGCATGACTGTCGGTTTGGCCAATCTCTTTTGCGGTGTATGCGTCGGTATC
ACGGGCAGTTCTTGCGCCTTGGCCGACGCTCAAAACGCCTCGCTGTTCGTGAAGATCCTC
ATCGTTGAAATCTTCGGCTCTGCCCTCGGACTGTTTGGCGTCATCATCGGCATCGTCATG
ACGTCCAATGCCAAGTTCGGCTAA
>g284-678893-681196
ATGGCGACGCACAAGGGACACTTCAGGTGGCATAACGAAGAGGAGGTCTACGGCAAGGCC
CAGTGGACCCGCGTCTACTTCGTGCTCAAAATCGAGGAGCGTGCCCTCTACATCTACAAG
CAGAAGAAGGACCAGTCCAAGGAGTGGAAGAAGATCGACGTCTCCAAGGGTTCGTGCGAG
AACGGCCGCCACAAGCGCCAGAAGCACGTCTTCGTGTTCACCGCCAACGACCAGAGGAAG
TACGTCTTCCGCTCCGAGCACAAGAACAAGAAGAAGCAGTGGGTCCGCTACCTCAACGAC
GCCGCCAAGGATGCCAAGAAGGTCGAGACCCCCAAGGCGGCGGCCGTGGAGAAGAAGGAG
GAGCCCAAGCCCGAGCCGGTGAAGAAGGCCGACGACGAGAGCTCGTCGTCCGACGATGAG
TCGTCGTCTTCCGATGACGAGAGCTCGTCGTCCGAGGGCGAAGATGAGGACGCTGCCGCC
GCTGCCAAGAAGAAGGCCGACGAAGAGGCCGAGGCCGCCGCTGCCGCCAAGAAGAAGGCC
GATGACGAGGCCGCCGCCGCCGCTGCTGCCAAGAAGGCCGAAGAGGAAGCCGCCGCTGCC
GCTGCTGCCAAGAAGGCCGAGGAGGAGGCCGCCGCCGCCGCCGCCGCCAAGAAGGAAGCC
GAGAAGGAGTCGGACTCGTCGTCGTCGGATGAGTCGTCGTCTTCTGATGATTCGTCGTCG
TCCGAGTCTGAGTCCGAGGCGGAGGAGAAGCCCAAGGATGCGCCCGCGCCCGCTGCCGAG
CCCGAGCCCGAGGTGGGCAAGAACGTGACCGTGGAGAACACCTACCCGCGGCTGATTCAC
CTGCAGGGCAAGAAGAAGACCGACATCACCTTCACCCACGTGGCCACCACCGGCGCGAGC
GTCAACGACACCGACGTCTTCATCCTCGACAACAACAAGACCGTCTACACCTGGCTCGGC
GCCAGCCCCAACATGTGGCAGAAGCAGGCCGCCGCGGCTCTCACCCGCGCCATGGACGAC
GAGCGTGGCTCGAGCGTCAGCAACCTGGTCATCTCCAACGCGGGCACCTCCGACGCGGCC
AAGTTCTGGGAGCTGCTGGGCGGCAAGCCCGAGACGCTGTCTAAGTCGGCGCCCGAGGCG
CAGGCGCCCGTGCTGTTCCGGCTGTCCGACGAGTCAGGCAAGATGGAGTTCAACGAGGTG
GGCCGGGGCAACCCGCTCAAGCGCTCCCTGCTCCAGGCCGACGACGTGTTCATCGCCGAC
CTGGGCTTCCACATCTTCGTGTGGATCGGCAAGAAGGCCTCGCGCGACGAGCGCGACAAG
GCCGTCGGCTACGCCGTGGTCTACAGGCACAAGTACAACAGGCCCGAGCACATCCACATC
GAGAGGATCATGGACGGAGGCGAGAATGAGCTCCTCTACTCCTTCTTCCAGTAA
>g284-733975-734736
ATGGCCGCCACTCTTGCTACTCTCCCCAAGGAGGAGATCAACGATCTCGCCCTCACCTAT
GCCGCCCTCCTTCTCCACGATGGTGGCGTCAACGTGACTGGTGACAAGCTCGCCCAGGTC
GTGAAGGCTGCCAACGTCGATGTGCCCCCCTTCTACGTGAAGCTCTACGCCCGCGTTCTG
GCCAACCGCAACCTCGACGATCTCCTGTTCCAGACCGTCGCCGTCGCTCCCTCGGCTGCC
CCCGCCGCTGGTGCTGCCGCCCCCGCTGAGGCCGCTGCCGCTGGTGGCGACAAGCCCGCT
GGCAAGCCCGCCAAGGTTGAGGAAGAGGAGGAGGCTGAGGACATGGATGCCGGTGGTCTC
TTCGGCGGTGGTGATGACGATTACTAA
>g284-746724-749166
ATGCAGCGCTCTCTCGCCGTCGCGCTTTTCCTCGCCCTCTCTCTGATTTCCGCGCTCGCC
CTCCCCGCGGATGTGGTCAATGGCCACTACCCCGGCAGGAGCGGTGATTTCGTGCTCAGG
CAGAAGGGTTACGCTGCCGGTCAGGCCTACTTGACCCTCCTCTCTAAGAACGTCGAGCTC
GCTCAGGCTTCGCTCCCCGAGACCTCCGTGGCCGATCTCATCGCTCACCTCATGGGCGTT
GCTCCCCTGAACCCCGCCGCCAACAGGGAGAACTTCCCCCGCGTCAACTTCCTCGAGTTG
CCCCGCGCCAACGTTCTCTTCTTCGTCCAGTCCAGCAGCGCTTCCGGCGATGTCGCCCTC
ACCTCTGAGGCGTACCCTTCGGATTCCATCGCCAGGCTCACCACCATTCTCACCGGCTCC
AACCCCTCCGAGCACGGCATCATCCAGCGCGCTTGGTATGACCAGGTCCAGCGTGAGATC
GTCTCCGCCTACAGCAGCGAGTACGCTGGCGCCCGGGCCGCTTCTCTTCAGGACGTCGTG
ACCGCCGCCTTCGCTGGTAACTCGCTCGTCGTGTCTGCCGCCACCTGCCGCATGGTCGCC
TCCGCCCTGGCCGCCAAGTCCGCCGTGCTCCCCGCCGCCGCCAACAACCACGCTCTCTAC
TGGAGCTCGGAGTCGCAGAAGTTCGCCAGCGTGTACGCCGATGTCGCCGACAAGCTCGCC
TTCTCGTTCGCCGACGTTGAGGCCTTCGCCGCCAGCCTCGGTTCCTACGATGCCGCCACC
AAGCGTCTCTCCGTTGAGGGCGTCGAGTTCGACCTCAGCACCAAGGATGTCGGTTTCTTC
GCGGAGCTTAGCTTCGTGCACTCGCTCGTTGCTCAGCTGAAGACCGATGAGCGTCTGAAG
GCCCTCGTCGCCGACAAGGCCTCCGATTTCTTCACCTTCGCCATCAGCTTCCGCGGCCTC
GAGCACAAGTATGGCGCCGAGTCTGTTCAGGTCCGCGCTGCTCACTCCATCCTCACCACC
ACTATCTCCTTCGCCACCGAGGAGATCAACGCGCTCTACAACGGTCGCGCCATCGCCGAG
ATCGTGTCTGCCACCCCCTCGTCGCACATCACCGAGGAGCTCGTCGAGCAGGTTCAGACG
GCCGCCGCTGCCGACTCCGTCTTCTTCAACAAGAAGGTCTACCCCCAGGTCTACCTTGTG
AACTGGCTCTCCAACGGCGAGACCCAGAGCGAGGCCGTCTGCTCGCACCTCTCCTCCTCG
CTCACCAACTTCGAGGTTCACTGCGCCGAGGGCACCATGTCCGCTCTTGCCGTGGCCAGG
CTCAGCGAGGGTGATGAGGTGATGCGCATGCTTGTCGGAAACGGCACCAGCGGTGGCGCC
ACCAGCGACGACATCGCCACTTTCCAGATCGTCATCTGGACGTCGGTCATGCTCATCTTC
GCCGCCCTTGCCGCCAGCGTGGTGATGGCTTCTGTGCACGACACCACCGACTCGGTCATC
TACAACTCCCCCGCCCTGCACGCCAAGTCCTCCTAA
>g284-824385-824910
ATGGCCCCTCTTGATCCCTACAATGGCCAAGTCCAAGAACCACACGGCTCACAACCAGGG
TCACAAGAACCACAGGAACGGTATCAAGCGCGCCCCCAACCAGAGGCACGTCTCGACCAA
GGGCATGGACCCCAAGTTCCTCCGCAACCAGAGGTTCGCCAAGAAGCACAACAAGAAGGC
CGTGAAGGTGCTTCAGAAGCAGCAGAAGGCCGCCGCTGCCGTTGCCGCCGCCGCCCCCGC
TGCCACCAAGCAGTAAATGGATCGTTCTCAGTAAACGCTGTGCATTCAACAACTTTGTCC
TCTTGTTTGTGGCGACGAAAGAATTGGGAGAGATGGTTTTTATGTGGTGGATGA
>g297-76566-78284
ATGGGTAAGGAGAAGACTCACATCAACCTGGTCGTGATCGGTCACGTCGATGCCGGTAAG
TCCACCACTACTGGCCACTTGATCTACAAGTGCGGTGGTATCGACAAGCGAACCATCGAG
AAGTTCGAGGCTGAGGCCAAGGAGATGGGCAAGGGCTCGTTCAAGTACGCCTGGGTGCTC
GACAAGCTGAAGGCCGAGCGTGAGCGTGGTATCACCATCGATATCGCTCTCTGGAAGTTC
GAGACTGCCAAGTACTACTTCACCATCATCGATGCCCCCGGTCACCGTGATTTCATCAAG
AACATGATCACTGGTACCTCGCAGGCTGATGTGGCTATTCTCGTCATCGCCTCCGGTGAG
GGTGAGTTCGAGGCCGGTATCTCGAAGAACGGTCAGACCCGTGAGCACGCCCTGCTCGCC
TTCACCCTCGGTGTCAAGCAGATGATCGTGGTCTGCAACAAGATGGACAACGTCAACTGG
GCCGAGAACCGTTACAACGAGATCCAGCGTGAGGTGTCGGGCTACCTGAAGAAGGTCGGC
TACAACCCCAAGAACATCCCGTTCGTCCCGATCTCGGGCTTCCACGGTGACAACATGGTG
GACCGCACCGACAAGATGCCGTGGTACAAGGGCCCCACGCTCCTCGAGGCCCTCGACGAC
ATCAAGCCCCCCAAGCGCCCCATGGACAAGCCCCTCCGCGTGCCCCTCCAGGACGTCTAC
AAGATCGGCGGTATCGGTACCGTGCCCGTCGGCCGTGTCGAGACTGGCGTGCTCAAGCCC
GGCATGGTGGTGACCTTCGCCCCCGTGAACGTGACCACTGAGGTCAAGTCCGTCGAGATG
CACCACGAGGCCCTCCCCGAGGCCGTGCCCGGTGACAACGTCGGCTTCAACGTCAAGAAC
GTCTCCATCAAGGACATCCGTCGCGGTAACGTCGCCGGTGACTCCAAGAAGGACCCGCCC
CAGGAGACTGAGGACTTCACCGCCCAGGTCATCATCCTCAACCACCCTGGTCAGATCCAC
GCTGGCTATGCCCCCGTGCTCGATTGCCACACCGCCCACATTGCCTGCAAGTTCAAGGAG
CTCCTCGAGAAGGTCGACCGTCGTTCGGGTAAGAAGATGGAGGACAACCCCAAGGCCGTC
AAGTCTGGCGACGCCGCCATGGTGCTCCTCATCCCCTCGAAGCCCCTCTGCGTCGAGACC
TTCACCGACTACCCGCCCCTCGGCCGCTTCGCCGTCCGTGACATGAGGCAGACCGTCGCC
GTCGGCGTCATCAAGGCCGTCACCCGCAAGGACCCCAAGGCCGGTAAGGTTACCGCCTCG
GCCAAGAAGGCCGGCAAGAAGTAA
>g297-276196-278530
ATGCAGACCCGTGCCCTCATCGCCGTGCTGGCGGTGATCTTTGCGCTCCACTGCGCGCTT
GCCACCAGGACCATGGAGGCCCCGGCCTCCACCCTGTGCGCCTTCCGCGGTGCCTGCACC
AGGGGCGACTGGGTCCGCCTGCAGAAGTCGGCCGCCGACGACATCCACCCCATCATCATC
GCCGTCCGCATGAGGGCCGACGCTGAGGCCGCCTGCGACGCCCTGCTCATGGAGATCTCG
ACGCCCGGCTCGCCCAAGGCCGGCCTCAGGTACACCCACGAGCAGGTGGGCCAGCTCCTG
TCCAACTTCGACAACACCCGCACCGTCGAGCAGTGGCTGCGCGCCCACGATCTCGAGTAC
GAGACGGCGTCCAACGGTGACTTCATCCGCGTGTCGGCTCCCGTGGCCCGCCTCGAGCGT
CTCCTCGGTGCCCAGTTCCACGATTTCTCGTCCAAGGCGCTCGGTCTCACCGTCCGCCGT
ACCGCTTCCATCTCCATCCCCGAGAATGTGGCGTCTGCGATCGACTTCATTGGCAACACC
ATCCGTTTCCCCAACATCAAGGGTAACTTCGCCATGATGACCCCTGCTGGCACCCACACT
GCCCGCCAGAGCGGCAACGTCTCGCCCCAGACCATCTGGAAGTTCTACGGAGTGACCAAC
ACCACCGTCGACCGTCCCGTCACCCAGTCGCTCTTCGAGTCGCTCGGCCAGAGCTTCTCG
CCCAGCGATCTGTCCACCTTCCAGCGGCAGTTCGGTCTCCCCCAGCAGGCCGTGAGCAAG
GTGATCGGCCCGAACGACCCCAGCCAGTGCCAGTTCAACCCCAACAACTGCGCCGAGGCC
AACCTCGACGTGCAGTACATCATGGCCATCGCCCAGCGCGCCGCCACCACCTACTGGTCG
ATCCCCCAGTCGGCCCAGGACATCTTCCTCGACTGGATCGTGGCCCTCGCCGGCACCCCC
AACCCGCCCTCCGTCCACTCGATCTCCTACGGCTCGATCGCCACTGAGGACCCGCAGGCC
GACATGGCCCGCTTCAACGTCGAGGCCTGCAAGCTCGGCCTCAAGGGCGTGTCGATCATG
GTCTCGTCCGGCGACGACGGCGTGGCCAACTTCGAGGCCCGCAACGACCCCTCGTACTGC
GGCTTCAACCCGTCGTTCCCCGCCACGTGCCCCTACGTGACCGCCGTCGGTGCCACCATG
GGCCCCGAGTTCGGCCAGCCCGAGGTGGCCTGCACGTCCAACAACGGCGGCCTCATCACC
ACCGGCGGCGGCTTCTCGATCTACTTCAAGCGCCCGGCCTACCAGGACGCCGCGGTGAAG
CAGTACCTCGCCAACGCGCCCAACCTGCCCCCGACCTCCATGTTCAACGCCAACGGCCGC
GGCTACCCCGACGTCGCCATCATGGGCCACAACTACCAGGTCGTCATCGGCGGCAGCACC
TACATCCTCTCCGGCACGTCGGCCTCGTCGCCCGTGTTCGCTGGCTTCGTCACCCTCATC
AACGCCTACCGCGCCTCCATCGGCAAGGGTCCCGTCGGCTTCCTCAACCCCACCCTCTAC
CAGCTCCACGCCCAGAACTCGCCCGTCTTCAACGACATCACCTCCGGCGAGAACAACTGC
TGCGCCGGCAACCCGGGCCAGATCACCTGCTGCCAGTACGGCTTCAACGCCACCGCCGGC
TGGGATCCCCTCACCGGCCTCGGCTCCGTCAAGTACCCCCAGCTGGTCCAGGCCCTCGCC
CGGCTCTAA
>g297-280278-283691
ATGTCGTCGTCGGCGCCGCTCACCAGGGAGCAGGTCAAGAACCTGAAGCCCAACCAGGTC
CACGAGACCCTCGGCCGCAAGCTCCTCGTTGATGGCTTCGACGTGGTCTACGATCTGGAC
AAGTCGAAGGATCTCTACCTGCACGATGCGCGCACCGGCAGGGACTTCCTCGACTTCTTC
TCGTTCTTCGCGTCGTGGCCCATCTCGCACAACCACCCGAAGATGTCCGATCCCGACTTC
AAGCAGCGCCTGCTGAAGGCAGCCCTCCACAACCCCGCCAACTCAGACATCTACACGGTC
GAGATGGCGCAGTTCGTGGCCACGTTTGAGCGTGTGGCGATGCCACCTCAGTTCAAGCAT
CTGTTCTTCATCCAGGGCGGCAGCTTGGCCGTCGAGAACGCCATGAAGGCGGCCATGGAC
TGGAAGGTCCGCCTCAACTTCCAGAAGGGCCTCAAGCAGGAGAAGGGCAAGAAGGTGCTC
CACTTCAAGGAGGCCTTCCACGGCCGCGGCGGCTACACCGTCTCCGTGACCAACACCGCC
GACCCCAACAAGTACAAGTACTTCGCCCTCTTCGACCAGTGGCCCCGCATCACCAACCCC
AAGATCAAGTTCCCTCTGGCGGGCGAGAACCTGGTGCAGGCGATCAAGGACGAGGAGAAG
GCCATCGCGGAGATCGTGCACCACCTGGAGACGGACGGAGACGACGTGTGCTGCATCCTG
CTCGAGCCGATCCAGGGCGAGGGCGGCGACAACCACTTCCGCCCCGAGTTCTGGCAGCAG
CTGCGCCAGATTGCCAACAAGTACGACGTGCTCCTGATTGCTGACGAGGTCCAGAGCGGT
ATGGGTCTGACGGGCAAGTTCTGGGCGCACCAGTACTACGACGTCGTTCCCGACATCATT
GTCTTCGGCAAGAAGTCCCAGGTTTGCGGCTTCATGGCGACCGACCGCATGGGCATCGTC
AAGGAGAACGTGTTCGTCGTGCCCTCGCGCATCAACTCCACCTGGGGAGGCAACCTCGTC
GACATGGTGCGCTCGCGCCACTTCCTCGAGATCATCGAGGAGGACAACCTCGTCCAGAAC
TCGGCCCAGACCGGCGCCTACCTGCTGCAGCTCCTCCACGGCCTCCAGCGCGAGTTCCCC
CACCTCATCAACAACGTCCGCGGCAAGGGTCTGCTGTGCTCGTTCGACACTGTTACGCCC
GAGGTGACCAACAAGCTGAAGGCCTTCGCCTACGAGAAGAACATGCTCATCATCTCGTGC
GGCACCCAGACCGTCCGCTTCAGGCCCGTGCTCGACAGCAAGAAGGAGCACTTCGACATC
GCCGCCCGCATCCTGCGCGAGGCCCTGGTCCACATCGACCGCGAGAACAAGAAGGCCGCC
CTCTAA
>g297-345066-346440
ATGGCGGACACCTACCGTAGCGCGGCCACCGAGCTTTTCCACAACACCGAGGTGCGTCAG
CTGTTCCCCGAGGAACACGGGCGCATTTTCTCGGTCACCACCGACACCCCCATCGCCCAG
GTCTTCAAGACGCTGGTGGAGAACAAGATCTTGTCGGTGCCCGTGCTCAACACGAAGACG
CACAAGTGGGTGGGCTTCTTGGACATGGTCGACCTCGTGCACCACGCCCTCAGCGTGCTC
AGCATCCCCAAGAGCGAGCTCGACACCGCCGACTTTGACCAGCTCCTCGCCTCCTCCGAC
AAGTTCGCCAACGAGCCCTGCGGTTCCATCGTCGACCTGTCGAAGCGCAACCCGTACAAC
CCGGTGGACCAGCGTGCGCCGGTGACGAGCGCGATCGACATGATGGTCAACTGGCGCGTG
CACCGCGTGCCGGTGGTTGACACCTCGGGCGAGCTGATCACCATCGTGACTCAGTCGCAC
GTGGCGCGCCTCATCTACAAGTGGATCGCGCGATTCGAGCAGCTGGCCGACTCGACCGTC
GAGCAGCTCAAGCTGGGCTTCGCCGACGTGATCACCGTGCGCGAGGACGAGGAGGCCATC
GCCGCCTTCCGCCTCATTCACGAGCGCGGCGTGAGCGCCGTGGGCGTGGTCAACGCCGAG
GGCAGGCTGGTGGGCAATGTGAGCGCGTCGGACCTTAAGGCCATCGGCTACGACGGCAAG
CTGCTCGCCCGGCTCTTCGTGCCCGTGAGCGAGTTCGTCAAGGAGACGCGGCCGGAGGGC
GTCGGCGCCATCACCGTCGCGCCGCGCACCAGCTTCGGCCAAGTGCTGGACAAGCTGGTC
TCCAACCACATCCACCGCGTCTACGTCGTCGACGAGAGCCAGGGCGGCAAGCCCATCGGC
GTCATCTCCCTCCTCGAAATTCTCAAGGCCTTCCACCAGTAG
>g297-364757-366588
ATGAACACGACAGCGACAGCCACGTTTCCTCCCCACCTCTACTTTGCCCTGCTGAGTGCG
GTGCGTGAGTTCCGGGTGCCCGGGTGCTTCGTCGACATGGTGCAGGACACGGGGGTGGCC
ACCTTCACCGGCCGCCGACCTCAGGTCGTCTCTGCCGGCAAAGCCCAGTTCGAGCAGGTC
GTCAGACGGCTCCAGCGGGAAGACCTGGCCAAAGCCCAAGCCCACGCCGAGGAGGGTACG
ACCCCAGGACCGGTCTACGAGCCTGTTCGTGGTGCGGTCTCGCTGCCCACCAGCAGCCAT
TCGCCCAAGGTGGTGGCGTCGTACAGCTGGAACATGGCCGATCCCGATGAACCCTACGCC
ATCCCTGGCCATCTGAGCACCTTTTCCAAACCGGAGACTCCACTGAGCCTGGCCGCCGAT
GACGGCGGCGCTCACGACCCCTTATCGTGGGAGACGGCCCGAGGCATGGCCACCGCCATG
GAGCCCATCGTGGTGGCCTGTTGCGCTCTCGACCCGACGTTCTCGCTGTCGGCGTGCCAG
GTGGTGACGGACCGCAACAACCTGCGCAAGATCTTCAGGTGGGCCTGTGGGGACAGGAGC
CGATGGAGGATCGACCTGGAGAAGGTGGCGCCCGACACCATTGCCATGCTGCGCTTCGAC
CTCTCGGAGGAGGGCCAATCCGAGCTGCTGCAGCGCAAGCGCAGCGGGTACCGATTCGAG
GACCTGTGCTGCGCCGGTGATGATGGTCGTGCCAGCGCCAGCTCGGACTCCTACCGCGCG
GTACTGGAATTCGAAACGGCCGGCATCCGATTCCTCATGCGATGCGAGATCGACGCTGCC
ATGGCCCCGCAGGAGTCTGACGAGCTCCTCGAGTTGCAGCTGGCCCAGCTGTCGCTCGCG
GCTCGGCCCCTCAAGTTGGCAGGCAGTGAGCTGGTCTTTCAGCGCTCGGCCTGGCCTAAG
GCGCCCGAGTTCGTCGAGCTGACCTCCATGATTGACCAGCGCTTCGAACACCCCAGGGAT
TTGAAGGAGATGAAGGACAAGTGGTTCCAGGCCTACCTCGCCGGTGTGCACACGACGGTG
TTCGGCCTGAAGCCGGCCAGGGACAAGATGGTGGTGGAGTCCACTGTTGAGCTGAGCCCC
TCGGAGCTGTTGCAGTGGTTCAAGATGGACGTGTCAAGGGAGCTCACGGAGACGATGAAC
GTGGTGGGCTTCGTAAGGGAGTGCCTGGCCGACGAGCCCGTGGGCGCCGTGCATTCGCTC
TACTTCGAACCCACCGACCCCCAGCCGGGAGTTCGGTTGCGCCAACGGCGGGTCACTGTC
GATCGGGCCTTCCCGAGCTACGTCTACGAACAGCAATCCTACGCCGTCATCGACGCGGCC
ACCCACCAGGCTTGA
>g297-446656-448437
ATGGTGCTCGACATTAATCTGTTCAGGAAGGAAAAGGGAGGCAACCCCGAGCTGATCAAG
GAATCGCAGAGGAGGAGGTTCAAGCCGGTAGAGATCGTCGATGAAATCATCGCGCTCGAT
GAGGAATGGAGGAAGCTGCAGTTCCAAGTGGACGAGGTGAAGAAGGCACAGAGGAACCTG
TCGAAGGAGATCGGCGCCCTCTACAAGGCCAAGAAGACCGAGGAGGCCGACGCGATGAAG
CCCAAGATGGCCGACCTCAAGAAGGCGGAGGAGGACACCGAGAACGCCTGCAAGGTCATC
AAGGAGCAGCTCGACAAGAAGCTCAACCTCATCGGCAATCTCGTCCACGAGAGCGTGCCC
GTGAGCAAGGATGAGACCCACAACGCCGTCGTGACGGAATGGGGCGAGTTCAAGTTCAAC
GAGGCGGCCAGGCGCCACCATCACGAGCTGCTGTGGATGATCGACGGCTATGAGCCCGAC
AGGGGCGTGAAGGTGGCCGGCCACCGCGCCTACTTCCTCAAGGGCGTCGGCGTGCGACTC
AATCAGGCCCTCATCGCCTACGGCACCGACTTCCTGATGAAGAAGGGCTACACTGCGCTC
GCGACCCCTTTCTTCATGAACAAGGAGGTCATGGCCCGCACTGCCCAGCTCGAGGAATTC
GACGAGGCCCTCTACACCGTCATCGGCGAGAAGGGCGATGAGAAGTACCTCATCGCCACC
TCCGAGCAGCCCATCTCGGCCTTCCACGCCGAGGAGTGGCTCGAGCCCAAGGATCTGCCC
ATCCGGTATGCCGGCACGTCGTCGTGCTTCAGGAAGGAGGCTGGCGCTCACGGCAAGGAC
ACCTGGGGCATCTTCCGCGTCCATCAATTCGAGAAGATCGAGCAATTCTGCCTCACCGAG
CCGGAGAAGTCGTGGGAGGAGCACGAGAACATGGTAAAGACCGCCGAGGCCTTCTACCAG
TCGCTCGAGCTTCCCTATCACGTCGTGGCCATCGTTTCGGGCGAGCTTAACAACGCTGCC
GCTAAGAAGCTTGATCTCGAGGCCTGGTTCCCCGCCTTCGGCGAGTTCCGCGAGCTTGTC
TCGGCGTCCAACTGTACCGACTACCAGTCGAGGAGCCTGGAGGTCAGGTGTGGCAGCCGG
AAGCAGGGAGGTGAGAAGAAGTACGTGCACATGCTGAACGCCACGCTGTGCGCCACCACC
CGTACGATTTGCTGCATCCTGGAGAACTATCAGACTCCCGAGGGCGTGGCGGTGCCCAAG
GTGCTGCAGCCCTACCTCGGCGGCCTCGAGTTCATTCCCTTCGTCAAGGAGGCTCCCAAG
AAGCACGAGGAGGCTGTCAAGGGCAAGGAGAAGGCTGAGAAGAAGGCCGCGGGCCAGTAG
>g307-8066-9835
ATGAGAAGCAACCGCGCCTCCCTCCTCCGCACTTGCGCTACCACTTCCGCTCCTTCTTTC
GCGACCAGGCGTCTGTATGCTGCTGCCTCCAAGCCGCGCGTCCTGATCAACAAGGACACC
AAGGTCATTTGCCAGGGCTTCACCGGCAAGCAGGGTACCTTCCACAGCAAGCAGGCCATC
GAATATGGCACCAAGATGGTTGGCGGCGTGTCCCCGGGCAAGGGTGGCAGCAAGCACCTC
GACCTGCCTGTCTTCAACTCAGTCAAGGAGGCCAAGGAAAACACCGGAGCCGATGCGACT
GTGATTTACGTGCCCCCTCCCTTCGCTGCCGCGGCCATCATCGAGGCGATTGACGCCGAG
ATTCCGCTTGCCGTCTGTATCACTGAGGGTATTCCCCAGCAGGACATGGTGCGCGTGAAG
AAGCGTCTCATGGCGCCTGGCTGCAAGACCCGCCTCATCGGCCCCAACTGCCCCGGTATC
ATTAAGCCCGGCGAGTGCAAGATCGGTATCATGCCTGGTCACATCCACACCCCTGGCAAG
ATTGGTATCGTTTCCAGGTCTGGTACCCTGACTTACGAGGCTGTCGGACAGACGACCGCT
GTCGGTCTTGGTCAGTCCACCTGTGTCGGTATTGGTGGCGACCCCTTCAACGGCACCAAC
TTCGTCGACGTGCTCAAGCTGTTCGCCGAGGACCCCCAGACCGAGGGCATCATCCTCATC
GGTGAGATCGGAGGCTCCGACGAGGAGGCCGCTGCTCAGTGGATCAAGGACAACGCACTC
GTCTCCAAGAAGCCCGTCGTCTCCTTCATTGCCGGCCTTACCGCCCCTCCCGGCCGCAGG
ATGGGTCACGCTGGTGCTATCATCTCGGGCGGCAAGGGAACCGCCACCTCCAAGATCCAA
GCCCTCCAGGATGCTGGCGTCACCATCTCGGAATCGCCCGCCAAGCTCGGTTCGACGATG
CTCGCGGCCATGCGGGCTGCCGGCAAGGCCTAA
>g307-66407-66908
ATGGCCGCCGCAGTACCCGTTAATCCCAAGCCCTTCCTCACGGGACTCGTCAACAAAACA
GTAGCGGTCAAGCTCAAGTGGGGCATGGAATACCGAGGGCTTCTCGTCTCCGTGGATTCT
TACATGAATCTTCAACTGGCCAACACCGAGGAGTGGGTCGATGGCGCCTTCGCGGGAAAC
CTGGGCGAAGTGCTGATTCGATGCAACAACGTACTCTACGTCCGTGGCGTTCCCGAGGAC
GAAGACGCCGGCAAGGAGCGCGAAGACTGA
>g307-97486-100850
ATGTCTGCCACCAGGAAGAACATCTATGCCTCCGTGCCCTTCACCAACAGGGGCCAGTCG
GTGCACATCGCCGCCGACCCCAAGGGCGAGAACTTCGTCTACACGTGCGGTAATTCCGTC
ATCATCCGCTCCTTCAAGAACCCCCTGGAGGCCCACATGTACAACCAGCACTCGTGCCAG
ACCACCGTTGCTCGCTGGTCGCCCTCTGGCTACTACATCTGCTCTGGAGATGTGCACGGT
AACATCCGCGTGTGGGACACCACCAACATGAACGAGAACATCCTCAAGTTCGAGGGCAGG
CCGATCTCGGGACCCGTGAGGGACCTGGCCTGGAGCGAGGACAGCAAGCGCATCATCGCC
GTCGGCGAGGGCCGCGAGAAGTTCGGAACGGTCTTCATGTGGGATTCCGGCTCTTCCGTC
GGTGAGATCACGGGTCAGTCCAAGACCATCCTCACCTGCGACTTCAAGCCCACGCGTCCC
TACCGCATCATCACCGGCGGCGAGGACTTCACGGCCTGCTGGTTCGAGGGTCCCCCCTTC
AAGTTCAAGAAGAGCTTGACGGAGCACTCGCGCTTCGTGACGTGCACCCGCTTCTCGCCC
AACGGCGAAAAGGTGGCCACCGTGGGCCTGGACAAGAAGGGCATCATCTGGGACGGCAAG
ACCGGAGACAAGCTCGTGGAGCTGACCGGCCACACCCTGGGCGTGTACTCCGTCTCGTGG
AGCCCCGACTCGCAGCAGCTCATCACCGCCTCGGCCGACAAGACCGTCAGGCTGTGGGAT
GCCGCCTCCGGCGAGTGCCTCACCACGTTCGATCTGTCGTTCGGTAGCAACAGCGTGGAC
TACCAGCAGCTCGGCTGCCTGTGGCACCAGGACTACCTCGTGAGCGTGGGCCTCAACGGC
AACATCTACTACCTCGACCGCGCCCACCCCAACGAGCCCCGCCTCGTCATCAAGGGCCAC
AACAAGTTTGTGACCGCCCTCGCGGCTGACCCGAGCCGTGGCCACGTGTACACGGGCTCG
TACGACAGCCTCATCATCAGGTGGGACATCGCCACGGGCGCCAGCGAGACCCTCACCGGC
AACGGCCACTCCAACCAGATCCAGAGGCTCAAGGTGGCCGGCGAGACCCTCGTCTCCGCC
GCCATGGACGACAGTGTGCGCTTCAACTTCACCAACAGCGGCGCCTACGGTGACGCCGTC
TCCACCGACAGCTCGCCCCAGGACATCGCCGTCAGCCGCGATGCCTCCCTCACCGTGGCC
GTCACCCTCAAGAGCGTGGTGGTGCTGCGCAACGGCCGCACGGCGTCGACCCTGAACGTG
AGCTTCCAGCCGACGTCGGTGGCCATCTCGCCCGACTCGCGCACTGTCGCGGTGGGCGGC
AAGGACAACAAGGTGCACCTGTACGCCCTCAACGGCGACAACCTGAGCGAGACCCGCGTG
TTGGACAAGCACAGGGGCGCCCTCACCGCCGTCGAGTTCTCGCCCAGCGGCGAGTTCCTG
GCCACCGCCGACAGCGAGAAGCGCGACATCATCGTGTGGAACACGAGCGACTACTCGGTC
AAGGTCGAGGGCTGGTGCTTCCACACCGCCGCCGTGCGCTCCATCGCCTGGACCAGCGAC
TCGCTCAAGCTGGCCTCGGGCTCGCTGGACCAGGACGTCTACGTGTGGAACGTCAACCAC
CCGTCCAAGCGCATCCACATCTCCCGCGCCCACCAGGGCGGCGTCAACGCCGTCGCCTGG
CTCGACGACCTCACCCTCGCCACCGCCGGCCAGGACATGTCCTGGAAGACCTGGACCGTC
ACCCACCTCTAA
>g311-26894-28828
ATGTCTGCCCAGAAGGAGGTGTTGCACATCCGCGCCGAGGTCAAGCCGCAGGAGGAGCGC
ACGGCCATCACGCCCGAGAACGCCAAGAAGCTGATCGACTCGGGCCGCTACGCGCTCCAC
GTCGAGCGCAGCCCCGAGCGCATCTACAAGGATGACGAATATCAGCAGGCCGGGTGCACC
CTTGTTGAAACGGGCAGCTGGCCCAAGGCCGACCCCTCGGCCTTCATCGTGGGCCTCAAG
GAGCTCCCCGAGGAAGACACGCCGCTGACGCACAGGCACGTGTTCTTCGGGCACTGCTTC
AAGCAGCAGAACGGCTGGAAGGAGCTCCTCCACCGCTTCACCTCGGGCAAGGGCACCCTC
CTCGACCTCGAGTTCCTGGTCAACGAGCAGGGCCGACGTGTGGCCGCCTTCGGTCGTGCC
GCCGGTCTCGCTGGCTGCGCCGTCGGTCTGATGGTGTGGGCGCAGCAGCAGCTGCACGGC
TTCGACGTGCCGCTGTCGCCCATCAAGTCCTACCCGAGCGTAGCGGCGCTGGCCCGCGAG
GTGGCCCAGTCGCTCGCCCAGGTCAAGCAGCTCACGGGCAAGAGCCCCAAGGTCATGGTC
ATGGGCGCCAAGGGTCGGTGCGGCAGCGGAGCGCTGTACTTCCTTAAGGAGGCGGGCGTG
GAGGGCGTGACGGAGTGGGACATGGCCGAGACGAGCCGCGGCGGCCCGTTCGCGGAGATC
GCCGACCACCACATCTTCGTGAACTGCATCTACCTCATGGGCGCGATCCCGCCCTTCGTC
ACCAAGGAGCAGCTGGGCGCCATCCAGGACCGCCCGCTGACCGTCGTCGTCGACGTCTCG
TGCGACTACACCAACCCGGCCAACCCGCTCCCCGTCTACAACGAGGCCACCACCTTCCTC
TCCCCGACCGTCCGCGTGCCCCTCGCCTCCGGTCCGCTTGATGTGGTGTCGATCGACCAC
CTGCCGTCGATGATTCCGAGGGAGAGCTCGAGCGACTTCTCCACCGACCTCCTTCCTTCT
CTCCTCGAGCTTGCCGACTTTGCCGCCGGCCAGCCTGCTCCAGTCTGGGACCGCGCCCTC
GCCCTCTTCAACCAGAAGGTTGCCGAGGCTGCCGCCCAATAA
>g311-38746-39690
ATGGCTCAGTTCGATTTCAGCACCCCCCAGGCCATCGGCAAGTTCAACGGCTTCCTGTCC
GACAAGTCGTACGCGAAGGGCTACCTGCCCACCAGCGATGACGCTAAGCTGTTCGCCGAG
GTGACCCAGCACAACCCGTCGGGTCCGGAGAAGAAGTTCGCCCACGTCTACCGTTGGTAC
CAGCACATCAAGACCTTCAGCGATGAGGAGCGCTCCAGCTGGCCCGCTGAGGTCGTGCTC
GCCGAGCAGCAGACCGCTGCCGCCCCCGCCCCCGCTGCCGCCAAGGCCGAGGAGAAGAAG
GAGGAGAAGAAGGAGGAGGATGTCGATTTCGACGATGATGATCTCTTCGGCGGTGTGAGC
GAGGAGGAGCTCGCTGCTGAGAAGCAGAAGCGCGAGGCCGACAAGAAGAAGCACAAGAAG
GCCGAGGAGATCCAGAGGTCCAACATCATTCTCGATGTCAAGCCCTGGGAAGACACCACC
GACCTCAACAAGCTCGAGGAGCTCGTCAGGGGCATCACCATGGAGGGCCTCACCTGGGGT
CCCTCGAAGCAGGTCGAGGTCGCCTACGGTATCAAGAAGCTCCAGATCTCCTGCGTCGTC
GTCGATGACCTCGTCTACACTGAGGACCTCGAGGAGCAGATCCAGGCCTTCGACGAGTAC
GTGCAGTCCATCGACATTGCCGCCTTCACCAAGGTGTAA
>g311-44726-48631
ATGGATCTTAGCCAGATTTTGTTGAACGCCCAGAGCCCTGATCGCGAGGTGCGCGCTGGG
GCTGAGAAGCAGCTTGCTGCGGCTGAGCAGCATAACCTGCCCTCCTTCTTGCACGCTCTG
TGCCTGGAGCTCGCCTCCGCCGACAAGAACCCCCACTCTCGCCGCCTTGCTGGTCTCATC
TTGAAGAATGCCCTCGACGCCAAGGATGAAACCCGAAAGCAGCAGCGCATTCAGCAATGG
CTTGCTTTGGATGCTGCCGCCAAAGCACAGATCAAGGCCGGGGTTGTCAAAACTCTTGCT
GATTCGGTCAAGGAAGCCCGGCACACCGCGGCCCAGGTCCTGGCCAAAATCGCCGTCATC
GAGCTTCCTCGTGACCAATGGCCCGACCTGATCGAGAGCCTCATGAATCACATGATGCTG
CAGGACAACAACCTCAAGGAGAGCACTTTGGAAGCGCTTGGCTACATCTGCGAAGAAATC
GAACCTCAAGTGATTCAGGAGAAGTCCAACCAGATCTTGACCGCCGTCGTTCAGGGCATG
CGCAAGGAAGAGCCGAGCGCTGATGTCCGAGTAGCTGGCACCACTGCGCTGCTTAATGCC
CTTGAATTCGTCAAGGCCAATTTCGAGAAGGAGGCGGAGAGGAACTACATCTTGACCGTT
GTTTGCGAGGCCACCCAAGCCCCCGTTGCTCCTATCCGAGTTGCTGCCTTCGAATGCCTC
GTCAAGATCGCTGCTCTGTACTATGACAAAATCGGGACCTGGATGCAGAACGTCTTCAAC
ATTACGCTGGAGGCGATGAAGAAGGACGAAGAGCTGGTGGCGCAGCAAGCTGTAGAGTTC
TGGTCGACCATCTGCGACGTTGAAGTTGACATTTTGATGGAAATGGATGAGTATGTTGCT
GCCAAGGAGCAACCCCCGAGGGCCTGCCTGAACTACATCAAGGGAGCCATGAAGTTCCTC
ATCCCCGTTCTCATGGAGTGCCTCACAAAGCAGGAGGGCGAAGAGCAGGAGGAGGACGCG
TGGAACGTCGCTACTGCGGCGGGTACCTGCCTGGCCTTGATTGCCTCGACCGTGCTCGAT
GAGGTGGTTCCCCACGTGATGCCGTTCGTGCGCGACAACATCAGCAACACCAACTGGCAC
TTCCGCGAGGCTGCGCTCCTGGCTTTCGGTTCCATTCTCGAGGGTCCCACCGGCTACATC
ATCACCGAGCTTGTCACGCAAGCCATCCCCATCCTCCTTCAGCACACGAAGGACTCAGTC
ACTCTCGTCAAGGATTCCACCGTCTGGACTATCGGTCGCATCTGCCAGTTCCATGCTCAG
ACCATCGCTACCAAGCTGCCCGAAGTGGTTCAGGTCTTGATTGAAGCCCTCGCCGATGAG
CCTCGCATTGCCGCCAAGGCTTGCTGGGCCATCCACAACCTTGCCAGCGCTTACGAGGTT
GAGGACAAGCCTACGTCTCCGCTTTCGCCCTACTTTCAGGCCCTCGCCACTGCCCTGTTC
CACGCCAGCAGCAGGGATGACGCTGATGAGAGCTTCTTGGCCACCGCTTCTTATGAGGCC
CTCAACGTGCTGATTCAGAACTCGACCAAGGATTCGCTTCCGCTCATTGCGCAGCTGCTG
CCTCCGCTCCTCGAGCGTCTCGAGAAGACCTTTGCCGCTCAGATCGTGTCCAGCGACGAC
AAGGAAGCCGTTGTCGAGCTTCAGGGTCACCTCTGCGGCTCTCTTCAGGCCTGCACCCAG
AAGCTGGAGGGTGAGGTCAAACCGTACGCCGACCGTATGATGACCCTCTACCTCAGGCTG
TTTGAGTTGCAGAGCGCCACCGTCCAAGAGGAAGTGCTGATGGCTGTTGGTGCCCTCGCG
AATGCCGTGGAAGCTGACTTCGCCCGCTACATGCCCGCCTTCGGCAAGTGGCTCGAGCTC
GCGCTCCGCAACTGGGAGGAGCACGCCGTGTGCGCCATCGCAGTCGGCGTGGTTGGCGAC
ATCTGCCGCGCTCTCGGCGACAAGGTCACCCCTTACTGCGATGTTCTCGTCGGCCTTCTG
CTCGAGAACCTCAAGAACCCGCACATCAACCGCAACGTCAAGCCGCCCATCCTCTCGTGC
TTTGGTGACATTGCCCTTGCCATTGGCGGCCGATTCGAGCCCTACTTGCCCCACGTGATG
GGCATGCTCCAGCAGGCCTCCACTACTCCCATCCCCGAGACCGCCGACTACGACTTTGTC
GACTACGTGCTTCAGCTCCGAGAGGACATCTTCGAGGCCTACACGTCGATCATCCAGGGT
CTGCGCACCGACAACAAGTCGGATCTCTTCCTCCGGCATGTGGAACACGTCGTGGGCTTC
GTCTCCTTCGTCTGGAACGACCCTACCAAGTCCGACGAGGTCATCCGCGGTGCCGTGGGC
GTCCTCGGAGATCTCGCGCACGCTCTCGGCCCGAAGGTGAGGGACGTCCTCAAGCACGAG
ATCGTGCAGGGCATCATCAACGAGTGCGCCTCCTCGTCGAACCAGCAGACGAGGGAGGTG
GCCAAGTGGGCCAAGCAGGTAATCGCCAAGCTCTAA
>g311-205253-207421
ATGTTGGCCCGCCGCTCCTCCTCTGTCGCTGCCACGTCTGCCGTCAGGCTCACGTCTTCG
ACTGTCACTGCTTCCGGCCTTGTCCGACCGGCTGCCTCCGCCACTGCCGTCATCTCCAGC
CGCAATGCCTCCACCAAGAGCAAGGCCCAGCAGCTGCGCGACATGCTCACGTCGAACCAG
CTCGAGTACATCATGGAGGCCCACAACGGCCTGAGCGCCAAGATCGTCGAGGAGGCCGGT
TTCAAGGGCATCTGGGGCTCTGGCCTGTCCATCTCCGCCCAGCTCGGCGTCAGGGACAGT
AACGAAGCGTCCTACACTCAGGTTCTTGAGGTCCTCGAATTCATGAGCGACAACACCTCG
ATCCCCATCTTGCTTGATGGCGACACTGGCTACGGCAACTTCAACAACGCCAGGCGTCTC
ATCAGGAAGCTGGAGCAGAGGGGCGTCGCCGGTGTGTGCATTGAGGACAAGCTCTTCCCC
AAGACCAACTCGCTGCTCGAGGGCGTGAGCCAGCCCCTCGCCGACATCGACGAGTTCTGC
GGCAAGATCCAGGCCTGCAAGGACGCCCAGAAGGATGACGCCTTCTCTGTGGTGGCCCGC
TGCGAGTCGTTCATCGCCGGCTGGGGTCTCGACCACATGATGCAGAGGGCCGAGGCCTAC
TCCAAGGCCGGCGCCGACGCCATCCTGTGCCACTCCAAGCGCTCGGACTCGTCCGAGATC
CAGGCCTTCATGAAGGCCTGGAAGGAGCGCGGCAACAAGACCCCGGTCGTGATCGTGCCC
ACCAAGTACTACACCACGCCCTCCAAGGACTTCCAGGACTGGGGCGTGTCCCTCGTCATC
TGGGCCAATCACAACCTCAGGGCGAGCGTGGCGGCCATGCAGCAGACGTGCAAGCAGATC
TACGCCGATCAGAGCCTCATCAACGTTGAGGGCAAGATCGCGCCCGTCAACGAGGTCTTC
CGCCTCCAGAACAACGCCGAGCTCAAGGAGGCCGAGAAGAAGTATCTGCCCAAGAAGGAC
AAGAAGAACTGA
>g311-330656-333070
ATGAACCGCACTACTGCACTCAGGAATCCTATTTTCAACACTTGCTCGGCTTCCCGCCGG
GCGGCTTCGGTCACTTCGTCTGTCGCTAGATCGCAGCAGCTCCCATCGGTGATCGCTACA
AGGAGGGACTTCTCGAAGATCACCGCCAAGAAGCACGTCTCGAATGTGACGAACTTCAAG
ATCATCGAGTCCACGCTCCGCGAGGGTGAGCAGTTCGCCAACGCGTTCTTCACCACCGAC
AAGAAGCTCGAGATCGCCACGCTGCTCGATGACTTCGGCGTTGAGTACATTGAGCTCACG
AGCCCCGCCGCCTCGGCACAGAGCCTCCACGACTGCCAGAAGGTTGCCGCCCTGCCCCTC
AGGAAGTCCAAGACCCTGACGCACATCCGATGCACGATGGAAGACGCCAAGCTGGCCATC
GAGTCCGGAGTTGATGGCATCGATCTCGTGTTCGGCACGAGCAGTGTCCTCCGCGAGTTC
TCGCACGGTAAGGACATCGAGTACATCATCGACCAGGCCACCAAGGTCATCAACTACGTC
AAGGACGCCGGCAAGGAGGTCCGTTTCTCCAGCGAGGACAGCTTCCGCTCGGACATCGTC
GATCTGCTCAAGATCTACAGCTCTGTGAACAAGCTCAAGGTCGACCGTGTGGGTATTGCC
GACACAGTCGGTGTGGCCTCGCCGCTCCAGGTCATGGAGATGGTCAAGACCATTCGCGGC
GTCGTCGACTGCGACATCGAGTTCCACGCACACAACGACACCGGCTGCGCCATCGCCAAC
TCCTTTGTGGCCCTCGAGCACGGCGTGACCCACATCGACACCTCGATCCTCGGCATCGGC
GAGCGCAACGGCATCACCCCGCTCGGCGGCTTCGTGGCGCGCATGTACTCTGTCGACCGC
GAGTACGTCAAGAACAAGTACAACCTCAAGCTGCTCCGCGAGCTCGAGAACCTCGTCGCC
GACTGCGTCTCCGTCCAGGTGCCCTTCAACAACTACATCACCGGCTACACCGCCTTCACG
CACAAGGCCGGCATCCATGCCAAGGCCATCCTCAACAACCCCGAGACCTATGAGATCCTG
AGCCCCGAGGACTTTGGCATGCAGCGATACGTGCACATTGCCCATCGCCTCACCGGCTGG
AACGCTGTCAAGTCCCGAGCCCAGCAGCTCAACCTCGAGTTGACGGATGATGAGGTGAAG
GAGGTGACGAGGCGCATCAAGCAGCTGGCCGATGTGAAGAACCAGTCGATGGAGGACGTG
GATGCCGTGCTCAGAAGCTTCCACAGCGAGGTGACCAAGAACGAGATGTCGCACCACGTC
GGCATCGCCCTCGCCAACGGCCACACGCCCAACTTCTTTGCCAACCCCTAA
>g32-16416-18250
ATGGGCGAGGACGAGGTCGCGGTCACACAGGGAAGCGCAGATGGTGTACATGGAGCTGGC
ACCTTGGTGGAGGACGTGCACACGGCCAATGGTCCCTACACAGAGCCGCGGCAGAGCTCG
AGAGTTCGGCGCAGACACGTTTCACCCGAGCGAAGGCATGCTCACCCCCTGGGTCTTGAA
GTGGTGCTGGTTGAGCAGGGAGGCGTGGAGGTCTCACTGTGGCTGTGGTTGTGGGCAGTG
GAGTTGGGAATGCAGTTTTCTCGTGTGTTGACCGGATTTGCGGTGGTGGGCAGTGTGCTT
GAGGCCGAGTGCTTGCCAGAGTGCGGTCAGTGCGTGGCCCATGCCCACCAAGATTTCGTC
GAAGCACAGATAGCCATGCCCGGCCCTTGTGTTGAAGTCGTGGGCGAATGA
>g327-31897-32748
ATGGCGAAGGCTGGCATCAAGATCCCCCGCAACTTCAAGCTTCTTGAGGAGCTGGAGAAG
GGCGAAAAGGGCGTCGGTGACGGTGCTGTGAGCTATGGTCTCGCTGATCCCGATGACAGC
ATGCTCTCCAGCTGGGCTGGCACCATCATCGGCCCGCCTAACTCTGTTCACGAGGGCCGC
ATCTACACCGTTCACATCAACTGCGGCAACGAGTATCCCCAGAGGCCCCCTCTTGTGAAA
TTCGTGTCTCGCGTAAACATGGCCTGCGTCAACCAGTCCAACGGCACGGTTGATCCTCGT
GGCTTCGCCATTCTGGCGCAGTGGAAGGAGGAGTACAGCATCGAGACGATACTTGTGGGC
CTGCGCAACGAGATGACTTCTGCGCAGAACAGGAAGCTCTCGCAGCCTCCTGAGGGCACC
AACTTTTAG
>g327-32846-34973
ATGCGCAGGGGTCTTTCTACCGTTTCCAGGAGCGCCACCGTGTTCGGCAAGCAGGCGTGC
CTCGCCTCCTCCACCCTCAGGGTTGACCGCAGGGCCTACGCTACCTCGCCTGAGGATGCC
GCCCTTGAGGCTCAGATCGAGAAGGTCCTCGACCTCGTCAAGAAGCAGGGCAACCTCGGC
TCTGAGAAGCTCACCGTGAGGAAGCTCACTGAGCTCAGGCAGACCGTGCCCGAGATTGAT
CGCGCTCTCAGCAGCGTTGGCGACTCGGCCGTCAAGTACATGGCCGAGCTCGAGAAGCCC
GGTGCTGGCCCCCTCTCCGCCGTGCCCCCCGTGCGCGTCACCGTCACTGGTGCCGCCGGC
GCCATCGGCTACGCCATGCTCTTCCGCATTGCCAGCGGTGAGATGCTGGGCAAGCACCAG
CCCGTCATCCTGCAGCTCCTCGAGCTTGAGCCCGCCATGAAGGCCCTTGAGGGTGTCATC
ATGGAGCTCAAGGACTGCGCTTTCCCCCTGCTCCACGGCGTCACCGCCTCGAGCGACGTC
AACAAGGCCTTCGAGGGTGCTGATTTCGCCATGCTCGTTGGTGCCAAGCCCCGCACCAAG
GGTATGGAGCGCGGCGACCTCCTCAAGGAGAACGCCAACATCTTCTCTGTCCAGGGCAAG
GCTCTCAACAAGGTGGCCAACCGCGACACTCTCCGCGTTTGCGTTGTCGGCAACCCTGCC
AACACCAACGCCTTGATTGCCTCGGCCAACGCCCCCAACATCCACCCCCGCAGATTCACC
GCCATGACCAGGCTGGACCACAACCGCGGCATTGCGCAGCTCGCTGATAAGCTGAAGTGC
AAGGTCACCGACATTGAGCGCTTCGCCATCTGGGGTAACCACTCGGCCACCCAGTACCCC
GACATCTCCCACACTCAGATCAACGGCAAGTGGGCGAAGGACCTCGTCGACGAGAAGTGG
GTCAAGGACACCTTCATCCCCGACGTGCAGCAGCGTGGCGCTGCCATCATCGCCGCCCGC
GGTTCCTCGTCCGCCGCCTCGGCCGCCAACGCCGCCATTGAGCACATGCGCGATTGGGTC
AAGGGCACCAACGGCCAGTGGACCTCCATGGGCGTCTGGACCGGCAACGGCAAGGTCGGC
GACTACGGCACCAGCCCCGACATCTACTACTCGTTCCCCGTGGTCTGCGCCGACGGCGAG
TACACCATCGTCCAGAACGTCCCCGTCGACAAGTTCTCCGCCGAGAGGATGCAGAAGACC
AACGACGAGCTTGTCTCGGAGAAGAACGGCGTCGGCGAGTTGGCCAAGAGGACGACTGAC
CCCGCTGCCCTCCTCAAGGAGCAGTACAAGGGCGGCAAGCAGTAA
>g327-85077-85998
ATGGCCGCCCCCGCGAAGACCTACACGCTCGAGGAGCTGAAGAAGCACAACACCACGGAG
GATATCTGGATTGCCATCCAAGGCCGAGTCTATAACATCACTCCCTTCCTCGAGGAGCAC
CCTGGTGGTGATGGCGTCCTCGTTGACAATGCCGGCCTTGACTGCACCGGCGAGTTCGAG
GCTGTGGGCCACTCTGACGAGGCTAGGGCCACCCTTGAGCAGTTCTACATTGGCGACCTC
GTCACGGCCGATGGTGCCCCCGTCAAGTCCGAGAAGGCTCCTGTGCAGCCTAAGGTGTCG
CTGGCCGACACCAAGGCACCCAAGCCCAAGGTGGCTGCCCCCGCCCCCGCCGTGTGGAAG
CAGGCTGCCGTGCCTCTTCTCATCATCGCCGTCGGCTTCGTCCTCCGCTACTACTTCAGC
TCCTAA
>g327-124156-125730
ATGTCGTCGTCCGAAGATGAGAGGAAGAAGAGGTCTGACTCCGAGTCGGAGGAGGAGAGC
GAGGAGGTGGAATCCGAAGAGGAGGAGACGAGCGAGGAGGAGTCGGAGGAGGAGACCAGC
GAGGAGGAGTCCACGTCGAGCGACAAGACCTCGTCTTCTGCTTCTGCCAAGGCCACCCCG
CGATCGGCCACCACAACGCCGCGAGCCGAGGCCAAGAAGGAGCCCGAGATCTACAACCCG
CCCAAGACCAAGGCCAAGGAGGACAACGGCGACAAGAAGAACGGTAAGGCCGACAAGAAG
AAGAAGAGCAAGAAGAGCAAGAAGAACGATGAGCAGTCGGTTGTGGTGATCGACGGCCGA
ACGGTCCTCAAGAAGACCTACGAGCTGGCCAAGGACAAGGTCATCGAATTCACCGTCTCC
ATCGAGAACCGGAACTACAAGCCCGAGGAGAAGGTCGAGCTCAAGTTCGAGATCAAGAAC
CAGACCTCGAAGACCATGAAGTCAATCAAGTGCGTCGTCGAGACCAAGGGCGCCAAGCCC
GAGGGAGACAGCAAGGACAAGAAGAAGAAGAAGCAAAAGAAGCCCAAGAAGGGCGAGGCC
GAGCGGGGCGTCCCCACCGGTACCGAGGATGAGTTCTACTGCGGTGCCCGGTTCCCTCTC
GACAAGTACACCGACTTCAAGGGCACCGACAGCTACAGGCTCCCCAAGAAGATCGACAAG
TCCAGCGGCACTGTCAAGCACGAGCTGCATCTGTCGTTCCCCATCCGGTCGCGTCCCATC
AACAGCTGGAAGAACATCGACGCCTGGCTGCCCATCGAAATCGGGAACTAA
>g360-287806-289940
ATGGCCGATTACTCGCACTACGAATCCCTCGCCAAGAACTGGATCGGCAGCATCACCAAG
CAGCCCGTCAACGACTTCGAAGCCGATCTCAAGAGCGGCGTCGTCCTCTGCAACCTTGTC
AATGGCATTCGCCCCAACACCATCCTCAAGATCAACACCTACAACGCCGCCTACCGTCAA
ATGGAAAACATCGACAACTTCCTGAAGGTTGTGGAGAAGCTGGGCGTGCCCGCCGGCGAC
AAGTTCCAGACTGAGGATCTCTTCTACGGCAACAACATCGCCAAGGTGGTCCTCACCGTC
CTCTCGTTCGCCAACGCCATCCAGGGCCAGTTCAGCGCCCCCGCCGTTGACACCTCCGGA
CTGGACAACCTGAGGCAGATCGCTTCGTCGGCTCAGAAGGAGGGTCGCCAGAGGGAGAAG
ACCAGCGGTCTCTCGCTCCTCGAGGAGGGCGCCAAGAAGGCGCAGGTGCAGGCCTCGGCG
GCGTCGAGGACTGCCGACAAGCTCATCCAGAGCCGCGAGAAGTCGGTCGACACCTCGGGT
TCGCTGGGCCTGATCGAGAGCGGCCAGAAGGAGAAGCAGGCCATGATCTCTGATGCCAAG
CGCTCGGGCACCGACAACATCATCATGAGCAGGGAGAAGGCTCAGGCCACCGCCGAGCTG
GGCTTCATCGATTCGCAGCACAAGGGCCACCAGGCCATGATCTCCGACGCCAAGCGCTCT
GGCACCGACAACATCATCATGAGCAGGGAGAAGGCTCAGGCCACCGCCGAGCTGGGCTTC
ATCGACTCGCAGCACAAGGGCCACCAGGCCATGATCTCCGATGCCAAGCGCGCCGCCACC
GACTCGATCATCAAGAGCACTGAGAAGGCCCAGGCCAGCGCCGAGCTCGGCCTGATCGAG
AGCGGCCAGGTCCAGAAGCAGCAGATGATCTCCGACGCCAAGCGCTCGGGCACCGACAAC
ATCATCATGAGCAGGGAGAAGGCTCAGGCCACCGCCGACCTCGGCTTCATCGATTCGCAG
CACAAGGAGCACCAGCAGATGATCTCCGACGCCAAGCGCTCGGGCACCGACAACATCATC
AAGAGCAGGGAGCAGGCCCAGGCCACCGCCGAGCTGGGCTTCATCGACTCGCAGCACAAG
GAGCACCAGCAGATGATCTCCGATGCCAAGAGGCCCGGCACCGACAACATCATCAAGAGC
AGGGAGCAGGCTCAGGCCACCGCCGAGCTGGGTTTCATCGACTCGCAGCACAAGGATCAC
CAGGCCAACATCTCCGACGCCAAGAAGACCCAGCTCGACCAGATCGTCAGGCAGCCCGTC
GGCGGCGGCGGCGACGACTCTGTCAACCTCTAA
>g360-326525-327048
ATGTCTCACGTCGGACGCGGTAAGGGCGGCAAGGGTCTCGGCAAGGGTGGCAAGGGCCTC
GGCGGCGCCAAGCGTCACAGGAAGGTGCTCCGCGACAACATCCAGGGTATCACCAAGCCC
GCCATCCGTAGGCTCGCTCGCCGCGGTGGTGTCAAGCGTATCTCGGGCATGATCTACGAA
GAGACCCGCGGTGTGCTCAAGGTCTTCCTCGAGAACGTCATTCGCGACGCCGTGACCTAC
ACCGAGCACGCCCGCAGGAAGACCGTCACTGCCATGGACGTCGTCTACGCGCTCAAGAGG
CAGGGCCGCACCCTCTACGGCTTCGGCGGCTAA
>g360-349386-350210
ATGTCTGAGAAGTCTGGATTCATTTGGGCCGTGAGGACTGGTGATGTGAAGGGCGTCCAG
GATGGCCTTTCTAAGGGCGAGAACGTGAACCAGGTCGATGAGACCGTGAACAGGCGCACG
CCTCTTCACCACGCCGCTGACTTTGGTCACGCCGAGGTCCTCCAGATGCTCATCGCCAAG
GGCGCTGACGTGAACGCTCAGGATGCTTTCGGCATTACGCCCCTCCTGGCCGCCGTCTAC
GAGGGTCACACCGAGGCTGTGCAGGTGCTGGTCAAGGCCAAGGCTGACGTGAACGCCAAG
GGACCTGATGGCATGAGCGCTCTCGAGGCTGCCGAGAAGGATGAGGTCAAGGCCATTTTG
AAGTCTGCTGGTGCCAAGTAA
>g360-409586-410810
ATGCAGTCCTCCAAGCCTCTTCGCTTCGTTTTCCTGGGTCCTCCCGGCAGCGGTAAGGGC
ACGCAGGCCGATCTCCTGAAGAAGCAGCACACCGTGTGCCATCTCTCCACTGGCGATATG
CTCCGCGCTGCCGTGGCCGAGGGCAACGAAATCGGCAGGCAGGCCAAGGAGGTCATGGCT
GCCGGCAAGCTCGTGTCGGACGAGATCATGGTCAATCTGATCAAGGATAACCTCACCAAG
CCTGAGTGCAAGGATGGCTTCATCCTCGATGGCTTCCCTCGCACCGTCGTCCAGGCCGAG
AAGCTGGATGAGATGCTGGAGAAGAGCAACTCCAAGCTCACTGCCGCTCTCGAGTTCGAG
ATCGACGACGAGATCGTGCTCGACCGTCTCGGTGGCCGGTACGTGCACCCCGCCTCCGGC
CGATCGTACCACGTCAAGTACGCGCCCCCCAAGGTCGCCGGCAAGGACGACATCACTGGC
GAGCCCTTGATCCAGCGCAGCGACGACAACCCCGAGACGATCAAGAGGCGTCTGGCCAGC
TTCCACAAGGACACCGTGCCCGTGGCGGCCTACTACAAGAAGCAGGGTATCCTCGAGAAG
CTCGACGCCAAGAGGAAGCCCCAGGAGGTCCACGCCAGGGTGCAGAAGATCATCGAGACG
CGTCTTTGA
>g360-448580-450093
ATGCCGCCGTCGGCCTCATCTTCGTCCCTCAAATCTGGCGCCGCGGTGCCCCTCGACCTG
CGTAAGTGGGTGGGCAAGCGGGTGCTCCTCATTTCGGCGCACCCCGATGACATCGAAGCT
GCCGTGGGCGGCACCATCGCTCTCCTCACCGCCCAGGGTACCGAAGTCTTCTACACCATC
GCCACCAATGGCGACAAGGGCTGCGCCAACCCTCTCTGCGCTGGCTGGTCATCGGAGCAG
ATCGCGGCCACGAGGAGGCAGGAGGCCTTCAATGCCGCTCGCGTCCTGGGCGTGCCGGAG
AAGAACGTGCTGCTGCTGGACTACGAGGACAGCCAGCTCACTTCGTATCCCTTCGCTCAG
GTCAAGGCATCGATGATTGCGGCCATCAGGCGGTTCCAGCCGCAGATCGTTATGAGCTGG
TGGCCGTACCCGCGCTTCGAAATGAAGCCCTCTCAGGGCTGGGCCGATCTGGGCTACCAC
CCCGACCACCAGGCTGCGGGCCGATTCGCTTTGGAGACTCACTTCGAGGCCGGGCTGAGC
CTTCTGATGCCTGAGATTGGCCCGTCGCCAGCCCAGGTCGAGGAGTACTACATGTGGGAC
TTCATCACTCCCTCGCACTACATCGATATCTCCTCCGTTCTCGACGTCAAGATCAAGTCG
TGGCTGGAGCACAAGACGCAGTACCCCAACAGCACTGCCGTGAGCACCATGCTTACTGGC
CTCGGCCAAAGGGTGGCGGCCAACACCGGCGCCACCAACGTGCGCTTCGCCGAGGGCCTC
CAGATCTTCAGCTGA
>g376-109411-111198
ATGTCCACCGCTGGTAACGTCATCAAGTGCCGCGCTGCCGTCGCCTGGGAGGCTGGCAAG
GATCTGAGCATCGAGGAGATCGAGGTGGCTCCTCCCAAGGCTGGTGAGGTCCGCGTCAAG
ATCGTCGCGTCGGGTGTGTGCCACACTGATGCCTACACGCTGAGCGGCAAGGACTCTGAA
GGCATCTTCCCCGTAATCTTCGGCCACGAGGGCGGCGGTATCGTTGAGAGCGTGGGCGAG
GGCGTCACCTCGGTTCAGCCTGGTGACCACGTCATCCCCCTCTATATCCCCGAGTGCCGC
GAGTGCAAGTTCTGCACGTCGGGCAAGACCAACCTCTGCTCCAAGATTCGCGTCACCCAG
GGTAAGGGCTTCATGCCCGACGGTACCTCGCGCTTCACCTGCAAGGGCCAGAGCATCTAC
CACTACATGGGCACCTCCACCTTCAGCGAGTACACCGTCATGCCCGAGATCGCCGTCGCC
AAGATCCCCAAGGAGGCTCCCCTCGACAAGGTCTGCCTGCTCGGCTGCGGTATCACCACT
GGTTATGGTGCTGCCCTGAACACTGCCAAGGTCGAGGCTGGCTCGACTGTGGCCGTTTTC
GGTCTGGGTGGCGTTGGCGTCTCCGTCCTTCAGGGCGCCAAGGCTGCTGGTGCCAAGAGG
ATCATCGGTGTTGACATCAACGAGAGCAAGTACGATTTTGCCTACAAGATGGGCGCTACC
GAGTGCATCAACCCCAAGAACTACGACAAGCCCATCCAGGATGTCATCGTCGAGCTCACT
GATGGAGGCGTCGACTACAGCTTCGAGGCCATCGGTAACCCCATGACCATGAGGGCCGCG
CTTGAGTGCTGCCACAAGGGCTGGGGTGTGAGCACCATCATCGGTGTTGCCGAGGCCGGC
AAGACCATCGAGACCAGGCCCTTCCAGCTGGTCACCGGTCGCGTGTGGAAGGGTACCGCC
TTCGGCGGTGTCAAGGGCCGCACTGAGCTGCCCGTCATCGTTGACAGGTACCTGAAGGGT
GAGCTCAAGGTGGACGAGTACGTCACCTTCACCTACCCCCTGGACGAGATCAACGAGGCC
TTCCACGTCATGCACGAGGGCAAGAGCATCCGCTCCGTCATTCTCTTTTGA
>g376-239436-240820
ATGGATCAATACGATCATTTGTTCAAGATTTTGCTCGTGGGCGACAGTGGTGTGGGCAAG
TCTTCTCTGCTTCTCAGATTTACGGACGACATGTTTCAGGAGACCTTCATCAGCACCATC
GGTGTGGATTTCAAGATCAGGAACGTCACCATCAATGACAAGGTTGTGAAGCTTCAAATT
TGGGACACTGCTGGCCAGGAGCGGTTCCGTACCATCACGAGCAGCTACTACCGAGGGGCG
CACGGCATCATCGTGGTGTACGACATCACCGATCAGGCCTCGTTCAACAACGCCAAGATG
TGGCTCAACGAGATCGAGCGCTACGCGTGCGGCAACGTCACCAAGCTGCTGGTGGGCAAC
AAGGCCGACATGAACAACAAGCGAGTGATCGAGGCCACCACGGCCAAGGCCTTCGCCACC
CAGCAGGGCATGCTCTTCAGCGAGGCCTCGGCCAAGGCCGGCCAGGGCGTGGAAGACGCC
TTCATGCACCTCGTCAAGGAGATCTTCGACAGACGCGTCGCCGTCAGCGAGCAGCAGCAG
AAGAAGCCCGAGATCGTGCTTGGCGCTGGCGAGGACATCGGCAAGAAGAAGGGCGGATGC
TGCGCCTAA
>g376-390574-393298
ATGATTAATCGATTCTTCCTGCTCTTGAGCCTGGCGCTTCTCGCCCTGGCCATCACCGCC
TCGGCCACCCCCTCGTGCAAGAGCGAAGAGCGCCTGGTGCACGAGATTTCGCGGCCCGGC
CCCCACGGCGGCGAGAAGCAGCTCAACCGCGCCCTTGCCATCGCCCGCCTCGTTGCCACC
ATCCACGAGCGTCTCGAGAGCGCCGGCGTGTGCTACTTTGCCACCGGTCGCACGCTCGAG
GGCGTTTGGCGCCATGCCGCTTCGATGCCCCACCACGCCCACTCTGTCGACCTCGCCATC
GAGTCCGTCAATTTCGACACCGCTCGCCAGGTGCTGCTCGCGGAGCTGGAGACCGGCAGC
AACTACCTGGTGCAGGGCGATGAGGACGTGAAGCACCTGAAGGTGTCGATCGTCATCGAC
GGCGAGGTGTCGGCCTTCGTCAACCTGTTCGTCTACTCGCGGTCCGACGATGGCGAGCGT
CTCGTGCACCCGTGGTTGTCCTACCAGCCCGCCGTCGCCACCGTCTTCCCCACCAAGCTG
GCTTCGTTCCACGAGGGAACCATCCGCGTGCCGGCCGACGCCAAGGCGTTCCTTGTGGGC
CAGTACGGCAGCGAGCTCGATGCGCCCAACAAGAAGGCCTTCTCGTGGACCCACATGTCC
TACGTCACGCCTCACGCCGGCATGGCCACTGAGTACCCTCTGGTCACCGTGATCATGCCC
ACCTACGAGCGCCCGCAGTTCCTGGGAAAGGCCATCGAGCTCGTCCAGAGGCAGGACTAC
CCCAACATCGAGATCGTGATCGTCGACGATGGTCGCGTGTCGCAGGCCGAGAACCCCGCC
ATGATCGCCGCGCTGTCGGCCGCCAACGTGCGCTACATCCACCTCACCGAGCGCCGCTCC
ATCGGCGTCAAGCGCAACATCGCTGTCGAGAACGCGCGCGGCGAGATCGTGGTGCACTGG
GACGACGACGACTACTTCCGCGAGCACCGTATCTCGGCTCAGGTGGCGCCCATCATCCGC
GGCGAGGTGGACATGACCGTGCTCGAGCACCACTACTACTACATCCTCCCGACCCAGTCC
TTCTACATCGTCAAGCGCGCCTCCACGTGGGGTCCCCACTTCGGCACCTTCGTCTACCGC
AAGAGCCTGTTCGACAGCGGTCTCAGGTACCCCGACAACTCGGTGGCCGAGGACTACGCC
TTCGCCGAGTTCGCCCTCAACAAGGGTGCCACTGTGTACGTGATGAACAACGAGGACGGC
AAGCACGTGTACGTGCGCCACCACAACACGTGGGAGTTCGACTTCGCCGACTTCGACGTC
CAGGTCGAGAAGGTCGACCGCCCGGCCTTCGTGAGCGACCTCGACTGGGAGCACTTCTCC
ACCGTCGAGTCGGAGCCCATCTCCATCAAGCCCCCCAACCACTTCGCCTCGGACCTTATC
CAGTGGAACCGCCCTGAGCTGATGCCCGTCCGGTCGGACGCTCTTGCCTACCCTGCCTAC
CCTAACTACCCTAACTACCCGAACTACCCCCAATACAAGGTTCCCGAGTCTGATTACTCG
ATGGTGGCCAAGCTCGGCATCGGCATCGGTGTGGGCGGCGGCGTGGCCCTGGTGGTGGTG
GGCGGCCTCACGACCTACTTCATCCTCCAGCACAGGAAGAAGCACGTCGGCTACGCGCCC
ATTAACAACGACGACTACGCCTAA
>g376-450681-452588
ATGCACCACCACGTCCACGTCAGCGTCACCACCGAGACGAAGGTGCATCTGAAGTCGCAC
CACGGCAAGTTCCTCTGCGCCGAGCAGTCCGGCAAGGTCGTCGCCGACCGCGCGGACTGC
CGGGAGTGGGAGACGTGGACCATGCGCACCACCAACGGCAAGACCACCTTCCAGTCCCAC
CACGGCAAGTACCTCTGCGCCGAGCCGTCGGGCAAGCTGGTTGCCGATCGCTCCAGCCCC
GCGGAGTGGGAGCACTTCCACGTCATCCAACAGGGCTCCCACGCCGCGCTGCGTTCGCAC
CACGGCAAGTACGTGTGCGCCGAGAAGAACCACAAGGTGGTGGCCGACCGCTCGGCCCTC
GGGCCGTGGGAGCAGTGGCAGGTCATCCCCGTCGGGTCGCACTGCCTCCCGGCGGGTCAC
CACCACCCGTCGCCCCCGCAGGTGCACTGCCAGTCTCCGCAGCCGCAGTACCACCACCAC
CAGCAGCAGCAGCCCGTCGAGACGAAGGTGCAACTGAAGTCGCACCACGCCCGCTTCCTC
TGTGCTGAGCCCTCTGGCAAGGCCGTCGCCGACCGGAGGGAAGCCAAGGAGTGGGAGACG
TGGACCATGGTCACCGCCAACGGCAAGGCCACCTTCAAGTCGGCTCACGGCAAGTACCTC
TGCGCCGAGCCGTCGGGTAAGCTCGTAGCCGATCGCTCCAGCCCCGCCGAGTGGGAGCAC
TTCCAGATCGAGCACCACGGCTCGCAGGTGGCGCTGAGGACGCACCACGGCAAGTACGTG
TGCGCCGAGCAGAAGGACTACAAGGCCGTCGCCGACCGCTCAAAGGTTGATATTTGGGAG
AAGTGGCAGATCATCACCGTAGCCACTGTGACCCACCACCCGGTGCACTACGCGCCGCCG
CCTGCGTTTGGTGGCTACCCTCCGGCGCCCTCCAGCGGCTATCCTCCGCAGTCGGGTGGT
TATCCTCCCCAGCAGGGGTACCCGCAGCAGGGCTACCCTCCCCCGTCAGGTGGCTATCCC
CCACAGCAGGGCTACCCCACTGGCCTGAACCCGCCTGGCCAGTATCCCCCTGGCCACTAC
TAA
>g376-507238-509142
ATGGCGGACACGATGGAAGTTGAGAAGGGAGGCGACAAGCCCAAGACCCTGGAGGAGGCG
CTGCAGGAAGAGGAAATTGACCCTGAGATCCTCAACAGCTCGGTCGATGACATCACTGCG
CGCACGAGGGCACTCGATCGAGAAATCCAGATCTTGAGGCACGAACAAAACCGGCTTCAC
CACGAGCAGGCCGGCCTCAAGGAGAAGATCAAGGAGAACAACGAGAAGATTAAGCTCAAC
AAGCAGCTCCCCTATCTCGTCGCCAATGTCGTCGAGCTGATTGATGCCGAGCCCGAGGAA
GAGGAGGAGGGCAGCACCATGGAACTGGACGCTCATCCCGCCACTGGCAAATGCGCAGTG
ATCAAGACGTCCGACCGAAAGACCATCTTCCTCCCCGTGGTGGGTCTGGTGGACCCTGCC
GAGCTGAGGCCCGCCGACCTCATCGGCGTAAACAAGGACAGCTACCTCATCCTCGACACT
CTTCCGCCCGAATACGATGCCCGTGTGAAGGCCATGGAAGTCGACGAGCGGCCCACCGAG
GACTACTCAGACGTCGGTGGTCTCCACAAGCAGATTGAGGAGCTCATCGAAGCCGTCGTG
CTTCCCATCACTCACAAGGATCGTTTCCTTTCTATCGGCATCCAGCCGCCCAAAGGCGTG
CTGTTGTACGGCCCGCCCGGCACGGGTAAGACGCTGATGGCCCGTGCTTGCGCGGCGCAG
ACCAAGTCTACCTTCCTCAAGCTGGCCGGCCCCCAGCTGGTGCAGATGTTCATCGGTGAC
GGTGCCAAGCTGGTGCGCGACGCCTTTGCGCTGGCCAAGGAGAAGGCGCCCGCTATCATC
TTCATCGACGAGCTGGACGCCATCGGCACCAAGCGTTTCGACAGCGACAAGTCCGGTGAC
AGAGAGGTGCAGCGCACGATGCTGGAGCTCCTCAACCAGCTCGACGGATTCAGCAGCGAC
CTCAGCGTGAAGGTCATCGCCGCCACCAACCGAATCGACATTCTCGACCCCGCCCTGCTG
CGTTCGGGACGCCTGGACAGGAAGATCGAGTTCCCTCTGCCCAACGAGCAGGCCCGCGCC
TCCATCCTCCGCATCCACTCGCGTAAGATGAACGTCAGCAAGGATGTCAACTTCGAGGAG
CTGGGCCAGAGCACGGAGGACTTCAACGGCGCTCAGCTCAAGGCCGTGTGCGTGGAGGCG
GGTATGCTTGCGCTGAGGAGAGACGCCGAGGACCTCAGGCACGAGGACTTCATGGAGGGC
ATCGCCGCCGTTCAGGCAAAGAAGAAGAAGAGTCTGCAGTACTACGCCTAA
>g376-574166-576815
ATGGCCGAGCCTTTTGACGTCAACGAGCTTGCTGATTACTCTGATGGAGAGCTGGAGCAA
GAGGAACAGGAGACGTTGGAGGCGGGAGCCAAGTCCGACGAGGCCAAGACGTCGGCGGAT
ACCCACAGCACGGTTCACACTGCTTCGTTCAAGGATTTCCTCCTGAAGCCCGAACTCTTG
CGCGCCATCACCGACTGCGGTTTCGAGCACCCCTCGCAGGTGCAGTTCGAGGTCATCCCG
CAGGCGATCCTCGGCACGGACGTCATCTGCCAGGCCAACTCGGGCATGGGTAAGACCGCC
GTGTTCGTGCTCTCGGTCCTGCAGCAGATCTCGGCCGAAGACGGCACCAGCTGCCTCATT
CTGGCGCATACGCGCGAGCTGGCGTACCAGATCTCGCACGAGTTCGGCCGTTTCACCAAG
TACATGCCGAACATCAAGGCGTCCGTGTTCTTCGGTGGTCTGCCCATCGTTCAGGACAGG
GCTACGCTCAAGAAGGATCCCCCGCACATTGTGATCGGTACTCCTGGTCGTATCCTCGCT
CTGGCCAACGAGAAGGCTCTCGATCTCAAGAAGATCAAGTTCTTCGTCCTCGATGAGTGC
GACTCCCTCCTCGAGCCCATCGACATGCGCGCTGATGTTCAGAAGATCTTCCGTCTGACT
CCCCACAACAAGCAAGTCATGATGTTCTCGGCCACGCTCAACGACGAGATCCGCGCCGTC
TGCAAGAAGTTCATGCACAACCCGCTCGAAATCTACATCAGCGCCGGCAGCAAGTTGACC
CTCCACGGCCTCAACCAGTACTACGTCCAGCTCGAGGAGCGAGAGAAGACCCGCAAGCTC
GTCGACCTTCTGGACACTCTCGAGTTCAACCAGTGCGTCGTCTTCGTGAGCTCTGTCAAG
CGGGCCGCCGAGCTGAACAAGATCCTCGTCGAGCAGAACTTCCCCTCGATCGCCATCTAC
CGTGGCATGCAGCAGAAGGAGAGAATTGAGAAGTTCGCCCAGTTCAAGAGTCTGCGTGCC
CGTATCGTGGTCGCCACCAACCTTCTGGGTCGCGGTATCGACGTGGAGCGCATCAACGTC
GTGATCAACTACGACATGCCCGGTGAGGCCGACACCTACCTCCACAGGGTGGGCCGTGCT
GGTCGTTTCGGTACCAAGGGTCTGGCGATCTCGTTCGTGTCCACCAAGGAGGACGGCGAG
GTGCTCAACAAGGTGCAGAGCCTCTTCGTTGTGAACATCCCCGACCTCAAGCCCGGCGTG
GAGATCCCCAAGTCCTCCTACATGCAAGCCTAA
>g396-67898-68658
ATGAACCCCGAGCTTCAGTCCGCCATCGGCCAGGGTGCCGCCCTCAAGCACGCCGAGACC
GTCGACAAGTCGGCTCCTCAGATTGAGAACGTCACCGTCAAGAAGGTCGACCGCTCCTCC
TTCCTCGAGGAGGTGGCCAAGCCCCACGAGCTGAAGCACGCCGAGACCGTCGACAAGTCG
GGTCCCGCCATTCCTGAGGACGTGCACGTCAAGAAGGTTGACCGTGGTGCCTTCCTCTCT
GAGATCGAGAAGGCTGCCAAGCAGTAA
>g396-174006-175868
ATGAAGGTCGTCGGACTCGCGGTGGCGTTGGTGGCGGTGCTGGTGGTGATGAGCTGCCTG
GCCCCCAACTCCGGTGCTGAGGCCCAGAGCACCAGCTGGGACTTCCTCCTGCTCGTCCAG
CAGTGGGGTCCGGGCGTGTGCGCCACGTCGCGCGGCAAGCAGTGCGTCATCCCGTCCTAC
GTGCGCTACTGGACCCTCCACGGCATGTGGCCCAACAACTTCGACGGCTCGTACCCCGCG
AACTGTCCCGATTCGGAGTCGTTCAACATGCAGAGGCTCGAGCCCATCCGCAAGAGCCTG
ACCGCCTACTGGCCCACCCTCTACCCCAGCAACACCCTCGAGAGCTTCTGGGAGCACGAG
TTTGAGAAGCACGGCACCTGCGCTGCTTCGGACCCCACCCTCGCGACCGAGCTGGCCTAT
TTCAACGCCACCCTGACGGCCCGCGCCACCTTCGATATTTCCGTCGCCTTCTCCAAGGCC
GGCATCCAGCCCTCGTCCAACAAGGCCTACTCGATCGACACGATCAGCAAGGCGATCCAG
TCCGCCTACGGTGGTGTGCCCCTCGTGCAGTGCTCGCGCGAGAGCTCCCGCGCCCGTGGC
CCCGAGGCCCTGACCTCGATCGGCTTCTGCATCTCGTCGAGCCTCACCATCATCGACTGC
CCCACCAACATCATCCACAAGGAGGGCTGCCACAACTACGACGATGGCGTCTACTTCCTG
CCCTTCTAA
>g397-67935-68414
ATGGAAGGAGAAGATAGGAAGTCCAATATTGTGAAGGCCACGCCCGGCGAAGAGGGTGAG
GACGCTGTGATTTCAGTCAAATTGGCTCCCCAGGACGGCTCAGGAGTTGTGGAAATCCGA
GCAAAGGGAAAGACGAAGATCAAGCGACTGGTGGATGCTTGGGCGAAGCAGAAGAGCATC
AATCCGCAATCTGTGCGGCTTCTTGGACCGGAGGGACAGCGTCTCAATCTTGAAAGCACT
CTGAACGAAGCCAACATCAATGACGGTGACCAGATCGACGTCATGCTTCTGCAGACAGGA
GGCGCCAGAGAGTGA
>g397-92648-92940
ATGGTGACTCTGCTGCCAAGCGTAAGGAGAGGGATGCTGCCGCGATGCGCGAGAAGCAGA
AGGCTGCTGAAGAGAAGAGGCAGCAAGACGCCGGTGGCAAGTAGCACAGATATTCGCAAT
GTAAATCTTTTTTTCCACTTTTACGTCTGTGTCTCTTCCTTCTTCTCCACCACCGCTTGT
AGCTGCGCGAGCTGGTGTGGCGTCTGCTGCTCCTTCTGCTGTTGGCTTTCGTCGTAG
>g397-93901-94628
ATGGCCAACGTCGAGAACCCCGAGATTCAGCAGGCGTACGAGGACGTTCGCACTGACTCC
ACCGGTTACAACTGGTTCATCCTCGGCTACGCGAACAACACGACCATCAAGGTTGATGGC
AAGGGCAAGGGTGGCGTTGCTGAGGGTGTCTCGCACTTCAAGGAGAACGAGGTGCAGTAC
GGCTTCTTCAAGGTGTCCTTCGTGGCCAACGATGAGACCAAGAGGACCAAGTTCGTCCTG
GTCTCGTGGTCGGGCGAGAAGGCCTCCGTCCTCAGGAGGGGTAAGGTGAGCGTCCACAAG
GCCAGCATCAAGTCCATCATCAAGGACTACGCCGTCGAGGTCTCCACCTCCTCCATCGAG
GACCTCACTGAGGAGAACTTCGTCGCCAAGATCAAGGCTGTCAACTACTAG
>g397-100249-102018
ATGTCTTCCAAAGTGTTCGTTGGCAATTTGTCCTTCAAGACCGAGACCGAAGCGCTCCGT
GAGCACTTCAGCGCCGCCGGTACCGTAACGGAGGCCAACATCATCACCCGCGGCCCGCGC
TCGCTCGGCTATGGTTTCGTCGATTTCAAGACCCCCGCCGATGCCGAGAACGCCGTCAAG
CTTTTCAACCAGAAGGAGATCGATGGTCGCGTCGTCAACGTCGAGGTTGCTCACCCCCGC
CAGGAGGAGAGCGCCGCCAATGGTGGTGAGAAGGCCGCCGGCGCCGCCGCCGACGGCACC
CGCCCGCCCCGTGCCCGCCGCGGTCGTGGTGGCCGTCCCCAGACCGCCGAGGGCGAGGGT
GCCCCCCAGCCCCGCGAGGGTGGTCGCCGCTTCCGCCCCCGCCAGACCGATGGCGCCGAG
GGTGCCGAAGGCGAGGCCGTCGAGGGTGGCCGCAGGCGCGGCTACTCCGGTGCCGCCCCC
GGTGCTCCCCGCGGTACCGGCTTCGCCCCCCGCGGCCGTGGCGGTGCCCGCCGTGGTGCC
CCCAGGACCAACCCCGACGGCACCCCCGTCGCCGCTGGTGCCCCCGGCAGCCCCAAGCCC
GCCGGCGAGCGTGCCCCCAGGCCCTTCAGGGAGCGCAAGCCCCGAGAGCCCCAGGCCCCC
AGGCCCCAGCGCCCCAAGGAGCAGTCTGAGACCACCCTGTTCGTGGCCAACCTGCCGTTC
GCCGTGACTGATGCCGAGCTGGCTGAGATCTTCGCCGGCCTCAAGGTGACCAAGGCCCAC
GTTGTGGTCAAGCGCAACGGCAAGTCCAAGGGCTTCGGTTTCGTCGAGTTCGCCAACCAG
GAGGACCAGCTCGCTGCCCTCAAGGCCAAGGATGGTGAGGTCTACCAGGAGAGGCCCCTC
AACGTCAAGGTCGCCCTCGTCCAGGCCGTCGCCGATGCCGCCGCTGCCGCCGCCGAGGCT
GAGAAGCCCGCCGAGGGTGCCGAGGCCGCTGCCGCCCCCGCCACCACCACCACCGAGACT
AAGGAGGAGAAGGCTGCTGAGCCTAAGGCCGCCGAGACCTCTGCCTAA
>g397-103027-103558
ATGTCGGCTCGCAATTTCGGTCTTACGGAGGGTGAGATTCAAGAGCTCAAGAAGCGCCCC
TACCCGCGCTTCGCCCCTGCCCCCATGCAGCGCCTCATTTCGCAGGCTAAGGTCATCTGG
CCCTTCGCCGTCGGATGGGCTGTGACCCTTTTCCTGTACAGCAGGATCCCCATTAGCGAC
GATGACCGGGCCAAGTCTCACTACCAGTACCAGCTCGACGTTCTGGATGGCAAGATCAAG
CCCGAGGACCACCCCATCCACGGCCACCACGGTCACTAA
>g397-103656-104387
ATGTCGCAGAAGGTCGGCGTTACCGTCAAGGACGTTCCCGCTCAGGAGTTCGTGCAGGCT
TATGCCCAGCACTTGAAGCGCAGTGGCAAGATCCGCGTCCCCAAGTGGACCGATCTCGTC
AAGACCGCCACTTACAAGGAGCTGTCCCCTTATGATCCCGACTGGTACTACATCCGTGCG
GCCTCGATCGCCCGTAAGGTCTACCTTCGTGGTGGTCTCGGCGTCGGCAAGCTGAGGCAG
CTGTACGGCGGCAGGCAGAACAACGGCACCCGCCCCAGCCACCACTGCAGGGCCTCCGGC
GCTGTTCCCCGTTCCATCCTCCAGCAGCTCGAGAAGATCAAGGTCCTCGAGAAGGACACC
AAGGGTGGCAGGAAGATCACTTCCAACGGCCAGAGGGACCTCGACAGAATCGCCGGCACC
ATCAAGGTGGTGAAGACTATTTAA
>g397-123643-124298
ATGAAGGGTCGCGGAGTGCATAAGAGCGGCGTGAGTGCGCAGTTCACATCACACTCTCTC
ACCCTCGCACACAACCAAGCGTCGCGCGTACCCTACCACGTTTCTTACGCCATGACCACC
ATCGCCTACTCCAGCCCGGCCTCTGTCTTTGCCACCACCGGGGACGACAGCGCCCCGACC
CTCATGGACAGCCTGCTTGGCGGGCTCGAGGCTGGCTGGTTCGAGCTCATGGACGCCCTC
ACCTACGGCTTCGAAGACAAGGCCTACACGCCAACGGACCCCGCCGGCACCTTCTCCTCG
TGGCTCATGGATGAGAACTAG
>g397-177776-178607
ATGGTCAAGCAGGTCACCAGCAAGGACGAATTCAACACTGAGCTCGCCAACGCCGGCAGC
AAGCTGGTCGTGGTGGACTTCTTTGCCACGTGGTGCGGCCCGTGCAAGCGCATCGCGCCC
GCCATCGAGAAGATGAGCCAGGAGAACACCAACGTGGTGTTCCTCAAGGTCGACGTGGAT
GAGGTCGGCGATCTCGCCGCTGAGCTTTCCGTGAGCGCCATGCCCACCTTCCTCTTCTTC
AAGAACGGCAGCAAGGTGCACGAGGTGGTCGGCGCCAGCGAGGCCAAGATCGCTGAGGGC
ATCACCAAGCACCAGTAA
>g397-205796-207280
ATGTTGAAGAAGGGCATCTTCGCGGATAAGAGTAAGACGCTCAAGGAGCGCAAGCATTTC
AAGAAGGGAACGAAGAGATACGAATTGCACAAGAAGGCCAAGGAGACGCTGGGAAGTGGC
GACATCCGCACGGCAGTAGCCCTGCCCGAGGGCGAAGATCAGAACGAATGGCTCGCGACC
AACACCGTTGATTTCTTCAACCAGGTCAATCTGCTCTACGGCAGCATTACCCTCTTCTGC
ACGGAAAAGACCTGTGCGGTCATGAGCGCCTCGCCCAAGTACGAGTACCTCTGGGCCGAT
GAGATCAACAAGAAGCCCGTCAAGGTTTCTGCTCCTGAGTACGTGGACAAGCTCATGGAG
TGGATCCAATCCAAGCTGGACAACGAGCAGCTCTTCCCCCCAACGACGGATGTCCCTTTC
CCCAGGAACTTCGTCCTTGAGATCAAGCAAATCTTCAAGCGCCTGTTCCGTGTCTACGCC
CACATCTACTACTCCCACTTTGAGAAGATTGTCGACCTCGGCGAGGAGGCTCACCTCAAC
ACCTGCTTCAAGCACTACTACTACTTCGTCACCGAGTTCGACCTTGTGCCGAGAAAGGAA
ATGGCTCCTCTGGAAGACCTCATCCAAAACTTGACCGGCAAGTAG
>g397-224837-226378
ATGTCGAAGAAGAAGATCCTATTGAAGATTATCGTGCTCGGCGAGTCAGGGGTGGGTAAG
ACTTCGCTGCTGCTGCGGTACGTGGAGAGGAAATTCACCATGAACACCAAGTCCACGATT
GGTGCCAACTTCCTCACCAAGGAGGTAGAAGTTGATGACAAGGTGGCCACCTGCCAGATT
TGGGACACAGCCGGACAGGAACGCTTCCAAGGCTTGGGCACAGCGTTTTACCGAGGTTCG
GATGGTGTCATCTTCGTGTTTGACGTTACACAGAGAAGGACGTTCGAGGAACTCGAGCAC
TGGAAGGAGGCGTTTTTGATTCAAGTGGGGCAGGAGGGCAACAAGGACTTCCCCATGATC
ATTATTGCCAACAAGATCGATCTCAAGGAGGAGAGGGTCGTGAGCAGGAAGGAGCTGCAG
GAATGGTGCGCACAGTACGGTCTCAAGTTTTTCGAGGCCAGCGCCAAGGAGGATGAGAAC
GTAGAGAAGGCCTTCGAAGAAATCACGAGGCTCGTTATCTCGAAGATGAAGCCCGAGGAC
ATCATGTACGACACTGTAGATTTATCTGTCGGCGAGAAGAAGGAGGACAAGGGCTGCGAC
TGCTAG
>g397-418028-419548
ATGGCGCAGAAGGCGGCGAGTTCGCACAATGAATACCGAATTGTAGTTGTAGGAGCTGGT
GGTGTGGGCAAGTCTGCCCTCACCGTCATGTTCATCCAGGGCACTTTCCTCACCAAGTAT
GATCCGACCATCGAGGACAGCTACATGAAGCAAGTCGAAGTTGACGGCGTTGCCTGCACC
CTCGACATCATGGACACTGCCGGTCAGGAGGAGTTCGGCGCGCTCAGAGATCAGTACATG
AAGACGGGCCAGGGCTTCCTCATCGTGTACAGCATCACGACACTCACCAGCTTCGAGGCG
GTGACCAAGTTCAGGAATCAAATTTTGCGCGTCCAGGAGGACAGGCTCGACATCCCCATC
ATCCTCGTCGGCAACAAGAAGGATTTGGAGGAGGACCGCGAGGTGCCCACCGAGGACGGC
CAGGCGCTGTCGGAGAAGTTCAACTGCGACTTCCTCGAGGCCTCGGCCAAGACCAACACC
AACGTCAACGAAGCCTTCTTCCGCCTCGTTCACAGGATCAACAAGTGGCGCGAGAAGCAC
CCCCAGCAGGCCCCAAAACCCAAGCCGAAGAAGAAGAAGGGATGCTCTCTCTTCTAA
>g397-536627-538481
ATGCAGCACCGCTTCACGAATGCCCCCTCTTATGGATTGGACTTCTCCAACTCGCTCGAC
CCGTGGAAGGACATGAAGCAGCTGTGGACCGAGGCTGCGGCTGACAAGATCGCCCCCGTC
ACCGGCCCAAGCACGAGGACCCTGGACCCCATTACCACTGGTACCTCGGTGCTTGCCATC
CAGTACAAGGACGGCGTGATGCTTACTGCCGATACTCTTGTGTCGTACGGTTCGCTTGCT
CGATTCCGATCGTGCTCGCGCATCAAGAAGGTGGGCGAGTACACCATTCTGGGTGCCTCG
GGCGAGTACTCTGACTTCCAGTCCACCTCGGTCATGCTTGACGAGCTCATTGATCGGGAT
CACGCCTATGAGGACGGCGCCAAGCTGCACCCGCACGAGATCTTCTCTTACCTGGGTCGC
GTCATGTACGGCCGACGCAACAAGTTCGACCCTCTCTGGAATCAGTACATCCTTGCCGGC
TTCCGCGATGGCAAGAGCTTCCTCGGTCAGGTGGATCTCTACGGCACGGCTTTCCAGGAC
AGCACCCTCGCCACGGGCTACGGTGCGCACATCGCCAGGCCATTGATGCGCAAGGCCTAC
AGGCCCGACCTCTCCGAGGAGGAGGCGCGAAAGGTGCTGGAGGATTGCATGCGCGTGATG
TTTTACCGCGACGCCAGGACCATCAACAAGATCCAGCTGGCCAAGGTGACGGCAGAGGGT
ATTGTCGTGTCGGAGCCCTACGCGCTGGCGACCGAGTGGACGTACAACGAAATTGCCCTG
GGCTACCCGCGACCCGAGTAA
>g40-21764-24998
ATGACGTTGGTGGCGTACCTGAGCCCGAGTGCGCAGGCGGTTGGTGCCATGCCGCCTCGC
GCAAGGATTCCCCGCTACTCGAGCCCGATGAGCTTCCTGGGCCGCGTGCCCTACTCGCTG
GCCCTGCTGGCCACCGCCGTCGGACTGGTCCTCCTCCTGGGCGGGGCCGCCGCCAACAGA
GCGGTCCACGCGCGGTTCCTCGCCCATCGGGAAGACGCGGCTTCGGTCAATTTCGCTCTC
AACTTCACCCAGAAGGTGGATCATTTCGACAGCCTCAACCATGACACCTTCCACCAGCGC
TACTGGGAAAACCAGCGCTGGTGGAAGGTACCCACCCAGGCCAAGGCCCGCCCCCTGGCC
TTCCTGCGTATCAACTCATTCCATTGGGACGGGGATTGGAACACCTCGACCATTGCCACC
TGGGCCAAGGAACTCGGAGCGATCGTGTTCGACCTGGAGCCCAGGTACGCGGGCGAGAGC
TACCCCGTGCCCGACGTATCGACCCGCGACCTCCGCTACTACTCCATCGAACAAAATGTG
GCCGATGTGGTGAACTTCATCCAGGACAAGTCCACGCTCCTTCCCAACAACACGCGTTGG
ATCGTGATGGGCGACTTGTTTGACGGCGCGTTCGCGACCTGGGTGCGCGCCAAGCCCCGC
GTGGCGTTCGGCGTGGTGGCCACCTCGCCCCACCTGCTCGCCGTGGAGGGCTACACGGGC
TACGACCTCGCCTACCAGGCCTCCATCGGTAGGACCTGCTCGCGCATCGTTCACGACCTT
CTGGGCACGGTCTCGTTCACGCTCAACTCGTCGGCCGAGGCAAGAGCGGCCTACCAGGTG
CAGTGCGGCGTCGACGAGGCCGAACTGCTGCCCAACGCCGAGTTCATGTATGTGGTGTCG
ACCATCCTCATGGGCGCCATGGGCTACGACTCGATCAACATGTGCAAAACATTCTCGGAC
GTGCGGAACAGCGACAACGCCACCAGGCTTGAGGCACTCGCCAACTACATCGGCAACTAT
AACGACCAGAACTATATGATGTTCAAGCAGTGGGACTTTGCCCTCGACACGGGCGGCAAC
ATGACCGACTACGGCGCCGGCTACAGGCCCACCTACTACCTCAAGTGCACCCAGCTCGGC
CAGTTCGAGGTGTCATCAGGCTCGCCCTACAGTCTGGTGCCGGCCGAGGTCAACGTCGAT
TGGTACCTCTCCGTGTGCCAGAAGCTCTTCAACAATCTCACCACTGCCCGACCCAACACC
GACGACGTCAACACCAACTTTGGTGGTGGAGTGCCGACGGGTTGTAACATGGCGTTCGTT
CAATCGGCCAACGACCCCTACTCCAGTCTCGGTCCCAACGCCGCGGCCCTGGCCGAGGAC
TACGCCACGACGGGCAACAAGATCATCGAGATTGACTGCGAGTCGACGTCTATCGGCCTC
TCGCTGTTCTCCACGCCTACATCTGCCGACGTTGCTTGTCTCGTCACAGCGCGTAGCCAA
GTCCTCAAGACGCTCAAGGTCTGGCAAGCCCAAGAGGACACTTGTCCGATGTCGACGACG
GTGGTGGATGGTGGGAGCTCGGGGCACGATGACGGCGACATGGCGCTGGTGGGCGTCTTC
TCGGCCGCGGGCGGAGTCATCCTGGGCCTCGTCGTGGCCGTGGTGCTGTTCCTCTACTTC
GGTCGTCGCGTCATCCAGAGGTGGAAGACCTCCTTCTTCAGCAAGATCAACTGA
>g40-54465-55988
ATGGCCACGGCGGCACTCAATCTCGACTTCCTCGACTTGCCCAGCGAGCGTGAGGACAAC
CAGCTTCAAAATGAGGTGCTGGACGTAGACAGATTCACTCTGCCTGCTGTCAGCAACCCC
GCCTCCTTCCTTACCGATACTTTCCAAGAGACCCAAGCCGAGCCCATCAAAATGCTGCAC
GGAACCACTACCCTCTCCTTTGTCTTCCAGGGTGGCATCGTTGTCGCTGTCGACTCTCGT
GCCACGCAAGGAACCTACATTGCCTCGCAGTCAGTGCAGAAGGTGATCAGGATCAACCCC
TTCCTTCTGGGCACCATGGCCGGTGGTGCCGCCGATTGCTCCTACTGGGAGCGCGAGCTC
GGCAGGAGGACGCGACTCTACCAGCTGCGCAACAAGGAGCGCATGACCGTCGCCGCCGCC
TCCAAGGTCCTCTCCAACATCCTCTACTACTACCGCAACCAGGGCCTCTCCGTCGGCTCC
ATGATCTGCGGCTGGGACAAGCGGGGTCCCAACATCTTCTACGTGGACAGCGACGGCACA
CGCCTGAAGGGCGACAAGTTCTCGGTGGGCTCGGGCGGCACCTACGCCTATGGTGTGATG
GACGCCGGCTACCGCTTCGACCTCACCGTCGAGGAGGCCTGCGAGCTCGGCAGGCGCGCC
ATCTTCCACGCTGCCCATCGCGATGCCTACTCGGGCGGTACCATCAACGTGTATCACATT
TCGGAGCAGGGCTGGAAGCACATCTCGGCGCAGGACAGCAACGACCTCTACTACGAGCAG
TACGGCCTGGCGGCCAGGGAGAAGGCCGAAAAGGAGATGAACGAGTGA
>g40-56116-57430
ATGGCTGACACCGCTTCGGCCGAACAGAGAGGTGGGAGGGGTACTGGTGAGCGTGGCTTC
GGCGGCAGGGGCCGTGGTCGCGGTGAAGGTGGTCGTGGACGTGGAGGTGATGGCGGCGGT
CGTGGCCGCGGCCGCGGTGGTCCCCGTCGCGAGAAGGGTGAGGAGAAGGAGCACTGGGTG
CCCGCCACCAAGCTCGGCCGTCTCGTCAAGTCCGGCAAGGTGACCAAGCTCGAGCAGATC
TACCTCTACTCTCTCCCCGTCAAGGAGTACCAAATCATCGATCAGTTCGTCTCTGGCCTC
AAGGACGAGGTGCTCAAGATCATGCCCGTCCAGAAGCAGACCCGTGCCGGTCAGAGGACT
CGCTTCAAGGCTTTCGTCGTCGTTGGTGACTTCAACGGCCACGTCGGTCTCGGTGTCAAG
TGCTCGAAGGAAGTGGCCACCGCCATCCGTGCCGCCATCATCCTCGCGAAGCTGGCCGTC
GTGCCCGTGAGGCGTGGTTACTGGGGTAACTTCATCGGCAAGCCCCACACCGTCCCCGTC
AAGGTGACCGGCAAGTGCGGCTCGATCCGCGTGCGCCTCATCCCCGCCCCCAAGGGTACC
GGCCTCGTCGCCTCGCAGGTCCCCAAGAAGATGCTGCAGTTCGCCGGTATCGAGGACGTG
TTCACGTCGTCTTCGGGCCACACTGCCACCCTCGGCAACTTCGCCAAGGCCACCTTCTTC
GCCATCACCAAGACCTACCAGTTCCTCACGCCCGACTTGTGGAAGACTGGCAAGTTCCAG
AAGGACCTCTACCACGCCAACACCGACTTCCTCGCCTCGTACGGTGAGAAGCACGCCGCT
GAGGAGAAGCAGGCTCGTCACGAGAGGGACTAG
>g40-67477-71598
ATGAACGGAGTTGACAACGCACCTACCACTACTACCAATGCCCCCGCCCAGTTTTCGGAC
CTCGAGGAGGAGAACCGCAAGCTGAAGCAGCAGGTCGAAAGGCTCAAGCTCATGCTCCGC
CGCCAGCTCAACTACGACGGCGACGATCGCGACTTCGAGCACATCTTCGACCGTGCGCTC
AGCTGGGAGGGCGACTACAACGCTTCGGACGAGGAGTACAACAAGACCTACGACTCGGCC
GAGGAGACCGATCCCTACTCCGCCGCGGATGCGGAGGAGAAGCGCCGCCGGAATGACACT
GAGGGTGACTCCGGCGACGAGGCGAGGGACGAGAACAACTCCGAAGAGGGCAACGCTTCG
GAGGAGGACAAGATCGCGATCCAGGCGAGACGCCCCAGACGTGTCAGCGGCCTGCGCATC
ACGCCAGTGTTCGAGAACGACCACTGCGTCGAGATTGTGGCGCATGTCGTGACCGATATC
GATGACCCTTCGCTTACGGAAGAAGGGCCTATCGACCGGGAGTCGGGCAAGGACATTGAT
CAAGGATATTCGGGCGATCAGGAGGAGGGAGACTCGGGCGACAATGACGACCAGACCGAG
GTCGTGATTTCGGCCGACGGCAAGAAGACCAAGCGTCCCAGGCTCGGTCTCAAGAAGAGG
CAAAAGCTGAAGGAGAAGAAAGCCAAGTCCAGCGGCAAGCTTCCGCGCAAGAAGCTGATG
CCCCGCAAGGAGAGCTCCTCCTCTGGCTTCGTGCTGAGCGAGAAGCCTATCCCCCAGGGC
GAGAAGCAGGCGCGCAAGAGACCCAACCACAAGAGCAAGAGCAAGCGCGAGAAGGCCGCT
CTTAGGCCGCGCAGGCGCAGGAGGAACCCCTTGGTCCGCGCCGCGCTCGAGCCCTCAGAC
CTCCTGGCTCCTGTCGTCCCCGACGACTTCTACGAGACCAAGGCTGAGGCTGCCTCTGCG
AAGGACCTCAAGCTGAAGGTCTACGAGGAGCTTTCGAAGAGCTCGTCGATCGAGCTCATC
AGCACCGTGAGCACCGTCGAGGAGCTGCTCGCCAAGGAGCTCGACAAGGGCCAGTATTCG
GCGTTCTACCTCGTCAACCTGGGAGCGGTCGTCGAGAAGTTTCTTCAGTGGAAGAAGTAC
ATGCCGCGCGTGCGTCCCATGTACGCCGTCAAGTCGAACCCCGACATCAACATCATCCGC
GCGCTGCACTACCTCGGTACCGGATTCGACTGCGCCTCGCAGGCGGAGCTTGAGGAGGTC
AAGAGCATCGGCGCCAAGGCCGAGAACATCATCTACGCCAACCCGTGCAAGGGCAAGGAG
CACATTCTCTACGCCAAGCAGCACGGCTTTGACACGATGACCTTCGACAACGCCGCCGAG
CTCGACAAAATCATCGCTCTCCACCCGGCCGCGAAGCTTGTCATCCGCATCCTCCCCGAC
GATCGCTACTCGCTCATGCCCTTCGGCAGCAAGTTCGGCGCCTCCTTCGACGAGAGCTGC
AAGCTGATCCAGCGCTGCAAGGAGCTCGGTGCGAACCTCATCGGCGTCAGCTTCCACGTC
GGCAGTGGCTGCTACTCGAGCCAGGCCTGGCACGACGCTATTCGCCTTGCCCGGCGCGTC
TTCGACGCGGCCCAGGAGGCCGGCTACAAGATGACCCTGCTCGACATCGGCGGAGGTTAT
CCCGGTGTTGATGACGACGGCATGACCTTCGAGGAGACGATCGACGGCGTGGCCCAGATC
CTGGACGACCTGTTCCCCGCCGACGTCACCGTCATCGCTGAGCCCGGTCGCTACTTCTGC
ACGGCTGCCTACACTCTGGCCGTCACCATCATCTCCCGCAGGGACCGCTTTGTGTGCCGC
AACAGGTACGCCTTTGCCTCCCGTTCTTTCCTTTCTCTCTTTAGTCACGGACGTCTCCTT
AGGATGACTACATATAAACCCAGCTCGATGATATCTTATGATTATACTTGTAATTCTGCT
TCTCTGAATCGATTCGTCTGTTTTCATTGTCACACTGTTCTCTTGCTGTCCTCTTCTCCT
GGTCGTGTCTCACCTCTCTTCGCCCTCTCCGCCACCACATCAGGCCGCAGAACAAGCTCT
CGTTCGTGGGTCACGAGGAGGAAGAGGAGAAGCAAGAGCAGGAGACCGCAGCCGACGACA
CCACCGAGCAGGAGGAGATGCCCGCTCGCGAGGTGCTCTACTACCTCTCGGACGGTCTCT
ACGGCTCGTTCAACAACATCGTGTTCGACCACGCCAAGCCCCTGCCGCTCTCGATGA
>g40-116600-118758
ATGTCGGGTTCTGGCAAGGAAGTGTTGGTTCTTGGTGCCGGTATGGTGGTGCGCCCGCTC
GTGCCCTACCTCACCCAGCACGGCTACCGCGTCGTCGTCGCCTCCAGGACGCTGGCCAAG
GCGCAGCACATCGTCGAGGGCATCTCGGGCGCCAAGGCCGTCGAGTGCGACGTCGACACT
GATGAGGGCAAAGCCATCCTCGAGACCCTCCTGCCCTCTGCCGATGCCGTCGTGTCGCTG
CTGCCCTACCTCCTGCACCCGTTCCTCGCCAAGAGGGCTCTCGCCCACAACAAGCACTTC
TTCACCACCTCCTACGTCAGCCCGGCGATGAGGGAGCTCGACGAAGAGGCCAAGGCCAAG
AACCTCGTCTTCATCAACGAGTGTGGTGTCGACCCTGGCACTGACCACATGAGCGCCATG
CAGATCATCGACGACGTCAAGTCCAAGGGTGGCAAGATCCTCTCTTTCACCTCCTACTGC
GGTGGCCTCCCGGCGCCCGACAGCAACAACAACCCTCTCGGCTACAAGTTCAGCTGGTCC
GCCCGCGGCGTGCTGCTCGCCTCCACCAACAACGCCATCTTCCTTCAGGACGGCGAGAAG
AAGGAGATTCAGGGCAAGGACCTGTTTGACAGCTTCCACCTGGACTACATCCCCGAGCTC
AGCTCCGAGTTCGAGACCTACCCCAACCGTAACTCGCTCCAGTACATCGACGTCTATGGC
ATCACCACCACCCAGACCATGATCCGTGGCACCTACAGGAACAAGGGTTGGTGCCCCACC
GTGAAGAAGCTCGGCGCCGATCTGGGCTTCCTCGACCTGACCGAGCGCAACTTCCAGGGC
GTGACCTACGCGCAGGCTCTGCGTGAGATGATCAACAGCCAGGCGGCCGACAAGGAGGCC
CTGAAGAGCGACGTGCGCGCGTTCCTCAAGCTCGACGCCTCCAAGGAGTTCGTGATTTCC
ACCGCCGAATGGCTCGGTCTGTTCGAGGAGGAGCCCATCCCCGCCAAGATCAAGACCAGG
CTCGACGCTCTGTGCCACAAGATGGAGACCAAGATGCAGTACAGCGCCGGCGAGCGGGAC
ATGCTGCTGATGAAGCACACCTTCATCGCCGAGTACCCCGAGGGCAAGAAGGAGAAGATC
ACCTGCACGCTCATCGACTATGGCCTCCCCAACGGCGACTCGTCCATGGCCAGGACCGTC
TCCCTCCCCGTCGCCATCTCCATCCGCCTCGTGCTCGAGGGTAAGTTCACCACGCCCGGC
CTCCAGATCCCCATCATCAAGGAGCTCTACGAGCCCATCCTCCAGGAGCTCGAGGCCCTC
GAGCCCTCCATCAAGTTCGTTCACCACAGGGAAGCCCTGTAA
>g40-197330-199538
ATGAGAGAAATCGTGCACGTTCAGGTGGGTCAGTGCGGTAATCAGATCGGCGCCAAATTC
TGGGAGACCATCTGCGCCGAGCACGGCATTCTCCCCAACGGCCAGTGGGATGAGGACATC
GCTACCAACCCCGGCAAGGACACCATTCAGCTCGACAAGATCAACGTCTACTACACTGAG
GCCAATGGAGGCAAGTACGTGCCGCGAGCGGTGCTGGTCGACTTGGAGCCGGGCGTGATG
GATCAGATCAAGGGCAACAAGCTGGGCAAGATCTTCCGTCCCGACAACATGGTGCACGGC
CAGTCTGGCGCCGGCAACAACTGGGCCAAGGGTCACTACACAGAGGGCGCCGAGCTCGTG
GAGGAGGTCATGGACGTCGTGCGCAGGGAGGTGGAGAACTGCGACCTCATGCAGGGCTTC
CAGCTCTGCCACTCGCTCGGTGGCGGTACCGGCTCCGGTCTCGGTACCCTTGTCATGTCG
AAGCTGCGCGAGGAGTACCCCGACCGCATGATCTGCACCTTCAGCGTGGTCCCCTCGCCC
AAGGTCTCCGAGGTCGTCGTCGAGCCCTACAACGCCACTCTGTCCGTGCACCAGCTCATC
GAGAATGCCGATGAGGTGATGTGCATCGACAACGAGGCCCTCTACGACATCTGCTTCCGC
ACGCTCAAGCTGAACAACCCCAACTACTCGGAGCTGAACGGCCTCGTGTCGCACGTCATG
AGCGGCATCACCTGCTCCCTGCGTTTCCCGGGTCAGCTCAACGCCGATCTCCGCAAGCTC
GCCGTGAACTTGATCCCGTTCCCGCGTCTGCACTTCTTCCTCGTCGGCTTTGCCCCCCTC
ACCTCGCTCTCCAGCAAGGACTACCTCAACCTCTCGGTGCTCGAGCTGACGCAGCAGATG
TTCAACCCGCTCAACATGATGGCGGCGTGCGACCCGCGCAACGGCCGGTACCTCACTGCC
TCGGCCATCTTCCGCGGCAAGGACCTCCGCACCAAGGAAGTCGAAGATGAGCTGCTGAAG
GTGCAGGGCAAGCACAGCTCGTCGTTCGTCGAGTGGATCCCCAACAACATCAAGTCGTCC
GTGTGCTCGGTGCCCAACGCCGGCAGGGACATCTCGGCCACCTTCCTCGGTAACTCGACG
TGCATCCAGCAGCTGTTCAAGCGCGTCGCGGAGCAGTCGAGCGTCATGTTCAAGCGCAAG
GCCTTCTGGCACTGGTACACCGAGGAGGGCATGGACGACATGGAGTACACCGAGGCCGAG
TCCAACTTGCTCGATCTCGTCCAGGAGTACCAGCAGTACGAGACCGCCGGCGTGGACGAC
GAGGAGGACCTTGCCAGCCACGAGGGCGAGTCTGTGGAGGTGGAGAGCGAGCTCAACCTC
GACGAGTAA
>g40-202171-203668
ATGGGAGACAAGCAGCAGCCGACTTTCGATTTCAGCAAGCTCAACTTTGATCTCTCCGGC
GGCGGCGCTGGCGGATTCAACCTCCAGGGCGCGCTTCAGGGCCTGCAGAGCCAGCTCGGC
GCGCTGGGCATGATTGGCGCCGACTCCGGCTACTTCCAGACCCTGCCTAAGGCCGTGCAG
CGCCGAGTCCGCGCTCTCCGCAACCTCGACCGCGAGTACGAGAAGATTGAGGAGGAGTTC
GAGAAGGAGCTGAAGGCCCTCGAGCTCAAGTACCACAAGGAGCAGTTCACCCCTCTCTAC
CAGAAGCGAGCGGCCATCATCAACGGTAAGGTTGAGCCCACCGACGAGGAGGCCAAGGAG
GAGTCCGATGATGAGGAGGAAGATGCCGAGCCCAAGATCCAGGAGATCAAGGAGGAGAAG
AAGGATGAGGAAGTAAAGGAGGAGACCGAGGAGGAGAAGAACGTCGTCGGTGTGCCCGAG
TTCTGGCTCACCGCGCTCAAGCACCACGAGATGCTCGACTCGGCCATCTCCGAGAAGGAC
GAGGAGGCCCTCAAGTACCTCACCGACATCACGCAGGACCCCGTCGAGGAGGAGGCCGGC
AGCTTCACCCTCAAGTTCCACTTCAGGGAGAACCCCTTCTTCACCAACGAGGTCATCAGC
AAGACCTACCACCTCGACGGTGACGGCGAGGAGGGTGACGAGGTTGTCTGCGAGAGCGTC
GACAGCACTCAGATCAACTGGAAGGAGGGCCAGGACCTGACCCAGGGCGTCAAGAAGAGC
TTCGGCCCCGGTGGCTCGTTCTTCAACTTCTTCGCCCCGCCCGAGGTCAAGCAGGGCAAG
CAGCCCTCGCCTCAGATCGTGAGCATGATGGAGCTCGACTTCGAGATGGCCGTGTCGCTC
AAGGAGGAGATCATCAAGCACGCCGTGCACTGGTTCACCGGTGACGCCAGCGTCGATGGC
TTCGGTGGCGATGATGGTGAGGAGGACGATGAGGATGGCGAGCGACTTCATTTGCTTCTT
GAGCTTGTCGATCTCGCCCTGTTGCTGCCTCTGATGCTCTGA
>g406-152697-153628
ATGTCGGTCAAGCTTGAGAGCTCTGATGAACAGGTGTTCGAGGTCCCCAGGGAGATCGCC
GAGATGAGCGTCACCGTCAAGCACATGCTTGATGACGTTGATGCTGACAGCGACGCCCCC
ATTCCCCTCCCCAACGTCACTGGCAAGATCCTCGCCAAGGTTATCGAGTGGGCCAAGTAC
CACCACGCCAACCCTGACGCGCCTTCGGATGAGAAGAAGGACGAGAAGAGGACCGACGAC
ATCATCCCGTGGGACAAGGAGTTCTGCGAGGTCGACCAGCCCACCCTCTTCGAGCTCATC
CTCGCCGCTAACTACCTCGACATCAAGCCCCTCCTCGATCTTACCTGCAAGACCGTGGCC
AACATGATCAAGGGCAAGTCGCCCGAGGACATCAGGAAGACCTTCAACATCAAGAACGAC
TTCACCCCCGAGGAGGAGGAGCAGATCCGCAAGGAGAACGAGTGGTGCATGGACTTGTAA
>g406-157076-162485
ATGTCTGCCAAGGCCATCAGAGAGTACCATGGTAAGAACATCCTGGCGCGCCACCTCAAG
GAGCGATCCAAGGGCCAGTTCCTTCTGGACAACAAGCGCGTCCATGTGTCGCCGCAATCG
CCGGCCGCTTCGCCCATCAAGGCCGTCCTCGAGCAGCACCCGTGGCTGGTGACCGACAAG
CTCGTCGTCAAGCCGGATCAGCTGATCAAGAGGCGCGGAAAGTCGGGGCTCATCAAGCTC
AACGCCACGTGGGAGGAGTGCGAGCAGTGGCTCCAGGAGAGGCGCAGCAAGGAGGTCGAT
GTGGAAGGTGTCAAGGGAGTGCTCGAGTACTTTGTGGTGGAGCCGTTCGTGCCCCACGAG
GCTTCGGATGAGTACTACCTGGCCATCCGTTCGGGTCGCGATGGCGACGAGATTCTCTTC
CATCACGAAGGTGGCGTTGACGTCGGTGATGTCGATTCCAAGGCCCTCAAAGTGATGGTT
GAGGTCGACGAGAGCGTGAGCGTTGACACTGTGCGCAGCAAGCTGCTCACGAGCGAGACC
ATTCCCGCCGAGCGCAGGGAGCTGTTGGCCGAGTTCATCGCCCTTCTGCACGCCATCTAC
GTTGATCTCCACTTCACTTACCTCGAGATCAACCCGCTCGTCGTGGTCGGCAACAACGTT
CACGTGCTTGACTTGGCCGCCAGGCTCGACCAGACTGCCTACTTCGAGTGCCACAAGGGC
TGGGGCGAGATCGAGTTCCCGCCGCCCTTCGGCAGGCCTCTGCTCGAGGAGGAGCGCTAC
ATCCAGGAGCTCGACTCCAAGACCGGCGCGTCCCTCAAGCTGACCGTGCTCAACCCCAAC
GGTCGCATCTGGACCATGGTCGCCGGCGGTGGCGCCTCCGTCGTCTACGCCGACACCCTG
TCCGATCTGGGCATGGCCAATGAGGTGTGCAACTACGGTGAGTACTCGGGCGACCCCACT
GAGTCGCTCACCTTCGAGTACGCCAAGACGATCCTCTCCCTCATGACCAAGGGCGAGCCC
ATCCAGGGCGGCAAGGTGCTCATCATTGGCGGTGGTATTGCCAACTTCACCGACGTGGCG
GAGACATTCAAGGGTATCGTCAAGGCCATCAGGCACTTCCAGGAGCGCCTCAGGATGCAC
AAGGTCAAGATCTACGTGCGCAGGGGTGGTCCCAACTACCAGGAGGGCCTCCGCTCCATG
CGGGAGAGCGTCGAGGGCATGCAAATTCCCATCGTCGTGTCCGGCCCCGAGACCCACATG
ACCGCCATTTGCCAGCTGGCCATCGATGACCTGAAGAGCGCCATTGAGGGCGGCGAGGCC
TCCAGCATGACCCGCAACAAGAGCTGGGGCAACCTCGAGGCGGCTGAAAGCCCCGAGCTG
CAGTTCAGCCCCCAGGCTCCTCCCGCCGACGGCCCCAAGCAGCCCGTCACGGTCTCGATC
GCCGACGGTCTCCCCACCGACGAGAGCCGCACTCTTGCCCAGTCGCAGGGTGCCTACGCC
CAGCACAAGCCCGTCCACACCCTCTTCAGCAGCGAGACCCGTGCTGTCGTGTACGGCATG
CACCCCGTCGCCGTGCAGTCCATGCTCGACTTCGACCACCTGTGCGGCCGCAAGCAGCCC
TCGGTGTCGGCCATCGTCTACCCGTTCGCCGGTGCGCACTACCGCAAGTTCTTCTGGGGC
TCCCAGGAGATCATGATCTCCGTCATGCCCTCGCTCGCCGAGATCTTCGCCAAGAACCCC
TCCAGCAAGCTCGACACGCTGGTGAACTTTGCCTCGTGCCGCTCGGTCTATGAGTCAACC
AAGGAGGCCCTGGCTATCCCCAACATCAAGACCATCGCCATCATTGCCGAGGGCGTGCCG
GAGAGGCGCACGCTCCAGCTCATCAAGCTCGCCGAGACCAGGCCCGAGGCGGCCGGCGGC
CGCGTGACCATCATTGGTCCGGCCACCGTCGGCGGAATCAAGCCCGGCTGCTTCCGTATC
GGTAACACTGGTGGTCGTCTGGACAACGTGCTGGCCAGCAAGCTCTACCGACCCGGCAGC
GTCGCCTACGTGTCCCGATCCGGTGGTCTGTCGAACGAGCTCAACAACATCGTCTCGCGA
AACACCAACGGCGTCTACGAGGGTATTGCCATCGGCGGCGATCGCTACCCTGGCACCACC
TTCATGGACCACCTCCTCCGATTCCAGGCTGATCCGGGCGTGAAGATGATGGTCCTGCTG
GGTGAGGTCGGCGGCACTGAGGAGTACAAGGTGTGCGAGGCCCTCAAGAACGGCACCATC
ACCAAGCCCATCGTGGCCTGGTGCCTGGGCACGTGCGCCAAGATGTTCCCCACCGAGGTC
CAGTTCGGTCACGCCGGCGCCTGCGCCCACTCCGACCTGGAGACCGCCGACGCCAAGATC
CGCGCGCTCACCGAGGCCGGCGCCCACGTCCCCTCGTCCTTCAATGAGATCGCCACCCTC
ATCAACAAGGTCTACACCGATCTCGTCCAGAAGGGCGTCATCGTCGTCGGCAAGGAGCCC
CCTCCGCCCAAGATCCCCGTGGACTTCGCGTGGGCGAGGAAGCTGGGTCTTATCCGAAAG
CCCGCTTCGTTCATCTCCACCATCTCCGACGAGAGAGGCGAGGAGCTCATGTACGCCGGC
ATGCCCATTTCCCAGGTCTTCGAGGACGAGATCGGCATCGGCGGCGTCATCTCGCTGCTG
TGGTTCAGGAAGAGGCTGCCCCAGTACGCCTGCAAGTTCATCGAGATGGTGCTCATGATG
ACCGCCGACCACGGTCCCGCCGTCGCCGGTGCTCACAACACCATCGTGACTGCTCGTGCC
GGGCGCGATCTGGTGTCTTCGCTCTGCTCGGGTCTCCTCACCGTCGGTCCCCGTTTCGGT
GGTGCCGTGGACGGTGCGGCCAAGACCTTCTCGTGGGGTTACGACACTGGCCTCAGCCCC
TTCGAGTTCGTCGAGGAGATGCGCAAGAGGAAGGAGCTGATCGCTGGTATCGGCCACAAG
GTCAAGTCCATCCACAACCCTGACAAGAGAGTGACGATCCTCAAGGAGTTCGCCCTCAAG
CACTTCCCCGCCCACCCGGTGCTGGACTACGCCCTCGAGGTTGAGGTGCTCACCACCAAG
AAGAAGCCCAACCTCATCTTCAACGTCGACGGCTGCATCGGCTGCTGCTTCGTCGATCTG
CTCAGGCACTGCGGCGCCTTCGCTCCCGAAGAGGCTGAGGACTACCTGAAGAACGGATTC
TTGAACGGTCTGTTCGTGCTGGGTAGGAGCATCGGTTTCATTGGCCACTTCCTCGACCAG
ACCAGGCTCCAGGAGGGACTCTACAGGCACCCGACGGACGACATCTTCTACATGACCACG
TCGGGTCCCGACCAGTCGGTTGTTCCCCTCTAA
>g406-168617-169558
ATGGGCGCCTACAAGTATGTCCAGGAACTCTGGAGGAAGAAGCAGTCCGACGTGATGCGC
TTCCTCGCCAGGCTCAGGTGCTGGGAGTACAGGCAGTACCCCATGGTTCTGCGCGTCAGC
AGGCCCAGCCGCCCCGACAAGGCCCGCCGTCTCGGCTACAAGGCCAAGCAGGGCTTCGTC
ATCTACAGAGTGCGCGTCCGCCGTGGTGGCCGCAAGAGGAAGATCAGGAAGGGTCGCGTC
CACGGTAAGCCCGCCACCCAGGGTGTCAACGCCCTCAAGTTCCAGAGGTCCCTCCGTTCC
GTCGCTGAGGAGCGTGCTGGCCGTCGCTGCGGCAACCTCCGCGTCCTGAACTCTTACTGG
GTTGCCCAGGATGCCACCTTCAAGTTCTTCGAGATCATCCTCGTGGATCCCTTCCACAAG
GCCGTCCGTCGCGACCCCAAGATGAACTGGATCGCCGCCCCCACCCACAAGCACCGCGAG
ATGAGGGGTCTGACCTCCGCTGGCAAGAAGTACAGGGGTCTCCGTGGCAAGGGTCACAAC
TACACCAAGTCCAGGCCCTCGAGGCGCGCCGTCTGGAAGAGGAACAACAAGGTCTCGCTC
AGGCGTTACCGTTAA
>g406-172337-174388
ATGGACGAGGAGTATGATTGCATCGTTCTTGGCACTGGCCTCAAGGAGTGCGTGCTCAGC
GGCATGCTGTCGGTTGGTGGCCTCAAGGTTTTGCACATGGACCGCAATGGTTACTACGGC
GGCGACTGCGCGTCGCTCAATCTCACCCAGCTCTACGAGAAGTTCAAGGGCAACGCTGCT
CCCCCTGCCACTCTGGGTCAGTCCCGCGATTACAACGTCGATCTCATCCCCAAGTTCATC
ATGGCCAGCGGTATCCTCGTGAAGATGCTGATCCACACCGACGTCACTCGCTACCTCGAG
TTCAAGTCGGTCGATGGCTCGTACGTCGTGGTGCAGGGTAAGGTCCACAAGGTGCCCGCC
ACCGACGTGGAGGCCCTCAAGAGCCCCCTCATGGGCATGTTCGAGAAGCGTCGCTGCAAG
AAGTTCTTCATGTACGTCCAGGAATACGATGAGAACAACAGCAAGACCCACCAGGGTTAT
GACCTTCGCCGCATGACGATGAAGGAGCTCTTCGAGGCCTATGGTCTCGGAGAGGACACC
ATCGACTTCGTCGGTCACTCGCTGGCGCTCCACGTCAACGACGAGTACCTCTCGCAGCCC
GCCCTCCCCACTGTCGAGAGGATTCGTCTCTACGTCGAGTCGCTCGCCAGGTATACCAAG
TCTCCCTACATCTACCCGCTCTACGGTCTCGGTGAGCTTCCTCAGGCCTTCGCCAGGCTC
AGCGCCATTCACGGCGGTACCTACATGCTTAACAAGCCCGTCGAGGAGGTCATCATGGAG
GGCGGCAAGGTTGTCGGAATCAAGTCTGAGGGTGAGGTGGCCAGGTGCAAGTTCGTCATC
GGTGACCCCTCGTACTTCCCCGGCAAGGTCCGCCAGGTGGGTCACGTTGTGCGCACGATC
TGCTTCCTCGACCACCCCATCCCCAACACCAACAACTCGGAGAGCTGCCAGATCATTCTG
CCCCAGAAGCAGATTGGCCGCAAGTCGGACATCTACATCTCGGTTGTCTCCTCCTCCCAC
AACGTGTGCGCCAAGGGCAAGTTCGTCGCCATTGCGTCCACGACCGTGGAGACCAACAAC
CCCGAGGCTGAGCTGAAGGCCGCCTTCGACCTCATGGGTCCCATCATCGAGAAGTTCACC
TCGGTGTCGCCCATGCTGGTGCCCACCGATGATGGTACCTCGGACAAGATCTTCGTGTCC
ACGTCGTACGATGCCACGTCCCACTTTGAAACCACATGTATCGATATCCTCGACCTCTAC
AAGCGCGTGACTGGCAAGGACGCCGATCTCAGCCCGCCGCCCAAGGCCGCCGAGTAA
>g406-176156-178045
ATGAGACCCTCCACCCACCACCAGAGCACGTTCAAGGTCGCCAGCGACAAGGTCAAGTAC
AACGAGGAGCACATTGAGGCCGAGTACGAGTACCAGACCACCGTCGTGGAGGTCAAGGAT
GGTGAGGCCATCGTCAAGCCCGTCGCCAAGTCCTACACCTTCCGCACCCAGCGCGATGTG
GGCCGCGTCGGTACCATGATCGTCGGCCTCGGCGGCAACAACGGTACCACCGTCGTGGCC
GGAATCATCGCCAATCGCGAGGGCATCACCTGGCGCACCAACAAGGGCGTCCAGACCCCC
AACTACTGGGGCTCCGTGACCCAGGCCTCGACCCTCCGCCTCGGCTCCGATGCCGCCGGC
GATGATGTCTACATCCCCTTCCACTCGGTCCTGCCGATGGTGGACCCCAACGAGCTGGTG
GTCTCGGGCTGGGACATTTCGGGCCAGAATCTGGCGGAGGCGATGGAGCGCGCGCAGGTG
CTGCCATACGATCTCCAGCGCCAGCTGGTGCCGCTGATGAAGGACATGAAGCCGCTGCCG
TCCATCTACGAGCCGGACTTCATCGCCGCCAACCAGGCCGACCGCGCCGACAACCTCATC
GAGGGCACCAAGGCCGACCAGCTGGCCAAGATCAGGAAGGACATTCGCGAGTTCAAGGAG
GCCAACAAGCTCGACCGCGTCATCGTGCTGTGGTCGGCCAACACCGAGCGCTTCTGCGAC
GTCGCCACGGGCCTCAACGACACGGCCGACAACCTCATGGCCTCGATCAACAACAACGAG
AAGGAGGTGTCGCCGTCGACCATCTTCGCCGTGGCCTCGATCCTCGAGGGCGCTCCCTAC
ATCAACGGTTCGCCCCAGAACACCTTCGTGCCCGGCGTCGTCGAGCTCGCCCAGCGCCAC
AACGTCTCCATCGCCGGTGACGATTTCAAGACCGGTCAGACCAAGATGAAGTCCGTCATG
GTCGACTGGCTCATCGGTGCCGGTATCAAGCCCGTGTCGATCGTCTCCTACAACCATCTC
GGTAACAACGATGGCAAGAACCTGTCGGCCCCCTCGCAGTTCCGCTCCAAGGAGATCTCC
AAGTCCAACGTCGTCGATGACATGGTCGCCTCCAACCAGATCCTCTACGAGGCCGACGAG
CACCCCGACCACGTCGTCGTGATCAAGTACGTGCCCTACGTCGGCGACTCCAAGCGCGCC
CTCGACGAGTACACCTCGCGCATCTTCCTCGGCGGCCACAACACCATCGCCATGCACAAC
ACCTGCGAGGATTCGCTCCTGGCCGCCCCGCTCATCATCGATCTCATCATCCTCATGGAG
CTCATGGAGCGTATCGCCGTCAAGGAGACCTCCAACGAGAAGGACGAGTTCGAGAAGTTC
CACCCCGTCCAGTCCATCCTCTCCTACCTCCTCAAGGCCCCCGTCGTGCCCGCCGGTACC
CCCGTCGTCAACGCCCTCATGAAGCAGCGCGCCGCCATGGAGAACCTCTTCCGCGCCTGC
GTCGGCCTGCCCCCCGAGAACAACATGCTGCTCGAGTGCAAGCTCCGTGAGCGTCCTACT
TTCTAA
>g406-185101-187658
ATGCTCCGCCGCGTTGCTGCCACTCGTCGTGCCCCGGTCTCTGCTGCCTCCTTCCTGAGG
AGCTCATGGAACGCCCCTGCCGCTGCCAGGCTCTACTCTGCCAAGAGCGGTGATCACATC
ATCGGTATCGATCTCGGTACCACCAACTCGTGCGTGGCCATCATGGAGGGCTCTACGCCT
CGCGTGATCGAGAACTCGGAGGGTGAGCGCACCACTCCCTCGGTGGTGGCCTTCGTCAAG
GACGACCACGGCACCAACCGTCTGGTCGGCGCCACTGCCAAGCGTCAGGCCGTGACCAAC
CCCACCAACACCTTCTTCGCCGTCAAGCGTCTCATCGGCCGCGATTTCAACGACCCGATG
ACCCAGAAGGATCTCAAGATGGTGCCCTACAAGATTGTCCGTCACTCGAACGGCGATGCC
TGGCTCGAGGACAGCTGGGGCAAGAAGTACTCGCCCAGCGAGATCAGCGCCTTCACCCTC
ACCAAGATGAAGGAGACCGCGGAGGGCTACCTCGGCACCCAGGTCAAGAAGGCCGTCATC
ACCGTGCCCGCCTACTTCAACGATTCTCAGCGTCAGGCCACCAAGGACGCCGGCAAGATC
GCCGGTCTCCAGGTTGAGCGTATCATCAACGAGCCCACTGCCGCCGCCCTCGCCTACGGT
CTTACCAACAAGGGTGGCGAGACCGTCGCCGTCTACGATCTCGGTGGTGGTACCTTCGAT
ATCTCCATTCTCGAAATCTCCAAGGAGGGTGTCTTCGAGGTGAAGGCCACCAACGGTGAT
ACTTTCCTCGGCGGTGAGGATTTCGATAACACTCTCATGCAGCACCTCGTGGGCGAGTTC
AAGAAGGCCGAGGGCATCGATCTCTCCAAGGACAAGCTCGCTCTCCAGAGGCTGAAGGAG
GCCGCCGAGAAGGCCAAGTGCGAGCTCTCTTCCACCGTGTCGACCGAGATCAACTTGCCC
TTCATCACTGCCACCGCTGAGGGTCCCAAGCACCTCCACATCAAGCTCACCCGCGCCCAG
TTCGAGTCGCTCGTCGATCCCCTCGTCCAGCGCACCATCGATCCCTGCAAGTCCTGCCTC
AAGGATGCCGGCCTCGACAAGTCCGACATCAACAACGTCCTGCTCGTCGGCGGCATGACC
CGTATGCCCAAGGTCCAGGAGGTCGTCAAGCAGTTCTACGGCAAGCAGCCCAGCAAGGGT
GTCAACCCCGATGAGGCCGTCGCCGTCGGTGCCGCCATCCAGGCTGGCGTGCTCAAGGGT
GACGTCAAGCAGCTCCTGCTGCTCGACGTGACGCCTCTCTCGCTCGGCATTGAGACTCTC
GGTGGCGTGTGCACCAAGCTCATCACCAGGAACACCACCATCCCCACCAAGAAGTCCCAG
GTCTTCTCCACCGCCGCCGACGGCCAGACCGAGGTCGAGATCAAGGTCCTCCAGGGAGAG
CGCCACATGGCCAACGACAACAAGACCCTTGGCTCCTTCATCCTCTCCGGCATCCCGCCC
GCCCCCAAGGGCGTGCCCCAGGTCGAGGTCACCTTCGACATCGACGCCAACGGCATCGTC
AACGTCTCCGCCCGCGACAAGGCCACCGGCAAGGAGCAGGCCATCCGCATCCAGTCCTCC
GGCGGTCTCTCTGAGTCGGAGATCGACCGCATGGTCAAGGACGCCGAGACCCACGAGGAG
GAGGACCGCAAGCGCAAGGACCAGACCGAGGCCCGCAACCACGCCGAGTCCGTCATCTAC
GACATCGAGAAGAACCTCAACGAGTTCAAGGAGCACGTCGACCAGACCGAGGCCGAGCGT
CTGCGCGAGCAGATCACCGAGCTCAGGAAGACCTTGGAGTCGGCCGACGACCACCAGGCC
ATCAAGTCCGGCGCCGATTCGCTCCAGCGCGAGTCCCTCAAGGCCTTCGAGTCGGCCTAC
AAGCAGAAGGCCTCGTCCAACGACAGCGGCAGCAGCAGCAGCACCGAGAACAAGGAGGAC
GACACCCCCGACGCCGACATCAAGAAGTAG
>g414-25246-26688
ATGTATCTCTTCGATTGGTTCTGGGGTGTTCTCTCTTTCCTGGGTCTCTACCACAAAAGC
GCAAAGATCCTTTTCCTGGGCCTCGACAATGCAGGAAAGACTACCCTTCTTCACATGCTC
AAGGATGACCGCCTTGCCGTCCACTACCCGACCTTCCACCCCACGATGGAGGAATTGACT
CTGGGCAGCATCAGATTCAGGACCTACGATCTCGGTGGCCACACCACTGCCCGCAAGGTG
TGGAAGGATTACTACGCCGACGTGGATGCCATTGTTTTCCTTGTTGACAGCGTGGACCGC
GACCGTTTCCCTGAGTCCAAGAGGGAGCTCGACGGCCTGCTCTCTGCCGACGATTTGAAG
ACCATCCCCTTCCTTGTGCTTGGCAACAAGATCGACATCCCCAAGGCCGCCTCTGAGGCC
GAGCTCCGTCAGGCCCTTGGTCTCCACCAGACCACTGGCAAGAACAAGACCTCCCTGGGA
GACAACATCCGCCCCATTGAGATCTTCATGTGCAGCGTGGTCAAGCGCAGCGGTTATGGC
GAAGGCTTCCGTTGGTTGTCGAACTACCTGTAA
>g414-40155-41168
ATGTCCGACTACGACCACCTCTTCAAAATCCTCATGGTGGGGGACAGCGGTGTGGGCAAA
TCGTCCCTCCTGCTGAGGTTCACCGATGATACCTTCACGGACAACTTCATCAGCACCATC
GGTGTCGACTTCAAAATTAGGACCGTTAACCTTGATGGCAAGGTTATCAAAATGCAGATC
TGGGATACTGCGGGACAGGAGCGGTTCAGGACGATCACGAGCAGCTACTACCGAGGCGCG
CACGGCGTCATCCTCGTATACGACGTCACCGATCAGGTGAGCTTCAACAATGCGCGCCAG
TGGCTGACTGAGATCGAGCGGTACGCCTGCGGCAACGTCGTAAAGCTGCTCGTGGGCAAC
AAGAGCGATCTCGTGTCCAAGCGCGTCGTCTCCACCGCCACCGGCAAGGAGTTCGCCGAC
CAGTTCCATCTGCCCTTCATTGAGGCCTCGGCCAAGGACGGCAGCAACGTCAAGCAGGCC
TTCATGACCCTCGTCAAGGAGGTCTACGAAAAGGTCGTTGGCGATTCTGCTTCATCGGGT
GGCGGCTCTCTGGGCCAGTCAGACAACAACAAGGTCAACGTCGCTGCCGCCAAGCCGGAG
AAGAAGAAGGGCGGCCTCAAGTGCATCCTCTAA
>g414-146359-148708
ATGCCGAAGCGCGGAAACAAAGGACACAGGAAGGTCACCAAGAAGGGAGATTCCGCTCCC
GAGGCCACCGTCCCCCTCACCGAGGAGGAGCAGAAGGAGCTCGAGGCTGCCGCCCGTACT
GCCACGGGTGTGCTCGCGTCGCTCCCTCTGGCGACTGATATCAAGATCGAGCAGTTCTCG
CTCAATCTCGCAGGTAACGAGCTCCTCGTCGATGCGAAGCTCGAGCTCAACATGGGCCGT
CGTTACGGTCTCATCGGTCTGAATGGAACGGGCAAGAGCACGTTCCTCAAGTGCCTCGCC
GCGCGCGAGGTTCCCATCCCTAAGCACATCGACATTCTGTTGGTCGATCGCGAGCAGCGC
GCCAGCGACATGACCGCCCTCGAATGCGTCATTGAGGATCTCGAAGCCACCAGGGAGAGG
CTAGAGACAGAAGCCGAAGAACTCTGCATGTCGGATGACGGCGCCGAGAGCGACACTCTC
ACCCAGATCTACGAACGTCTCGAGGCTCTCGACTTGGACGTTGCGACGGCTGAGGCCAGC
AAGCTGCTCTTCGGTCTCGGCTTCACGTCGGAGATGCAGAGGAAGAAGGCGCGCGAGTTC
AGCGGTGGTTGGCGTATGCGTATCGCCCTCGCCAAGGCCCTCTTCGTCAAGCCCACCATG
CTTCTCCTCGATGAGCCCACCAACCATCTCGACCTCGAGGCCTGCGTGTGGCTCGAGGAG
TACCTCAAGACCTACCCTACCATCCTCGTGCTCGTCAGCCACAGTCAAGATTTCTTGAAC
GGCGTGTGCACCAACATCATGCTGCTGAAGGACCAGGAGCTGACGTACTACGGTGGCAAC
TACGACACGTACGTGCGATCGCGCCAGGAGAAGGAGACCAACCAAATGAAGAAGTACGAG
TGGGAGCAGGCCCAGGTGGCGCACATCAAGGACTACATCGCCCGATTCGGTCACGGTTCG
GCCAAGCTCGCCGCGCAGGCCAAGTCGCGCCAGAAGGTGCTCGACAAGATGCAGGACGCC
GGCCTCACCGAGCGCGTGACGACGGACAAGGTGCTCCGGCTGGAGTTCACCGACGTGGGC
ACCCTGCCCCCGCCCGTGCTGCAGTTCGTCGAGGTGAGCTTCGGCTACAACCTGCCGGCC
GGCGCCAAGAAGGGCAACTTCCTCTACCGCCGCCTCGACTTTGGCGTGGACCTCGACTCG
CGCGTGGCCCTCGTGGGTCCCAACGGTGCGGGCAAGTCGACGCTGCTCAACCTCATGGAG
GGCAGCCTCAACCCGACCGACGGCATGGTCAAGCGACACCTCAAGCTCCGAATCGGCAAG
TACAAGCAGCACCTCATGGACCAGCTCGACGGCAACCTCACCCCGCTCGAGTACCTCATG
AAGTGCTTCCCTGAGAACAAGGAGGTCGAGAAGATGCGCGCCGCCATGGGCAAGTTCGGC
CTCACCGGCAAGACCCAGATCACCCCCATGCGGGTCCTCTCCGACGGCCTCAAGAGCCGC
GTCGTGTTCGCCTGGCTGGCCTGGCAGGAGCCCCACCTCCTGCTCCTCGACGAGCCCACC
AACCACTTGGACATCGAGACCATCGACTCTCTCGCCGAGGCCATCAACAACTGGGACGGC
GGCATGGTCCTCGTCTCCCACGACTTCCGGCTCATCGAGCAGGTGGCCAAGGAGATCTGG
ATCTGCGAGAACCAGACCGTGTCCCCGTGGAAGGGCTCCATCCGAGCCTACAAGAAGCAC
CTCAAGGCCAAGATGGACGAGGAGGCGGCACGCAACAAGTAG
>g414-166386-167284
ATGCAGATCTTCGTCAAGACCCTGACCGGCAAGACCATCACCCTCGAGGTCGAGTCGAGC
GACACCATCGAGAACGTGAAGCAGAAGATTCAGGACAAGGAGGGTATTCCCCCTGATCAG
CAGCGTCTCATCTTCGCTGGCAAGCAGCTCGAGGACGGCCGCACGCTCGCCGACTACAAC
ATCCAGAAGGAGTCGACTCTCCACCTCGTTCTTCGTCTGCGCGGCGGCATGCAGATCTTC
GTCAAGACCCTCACCGGCAAGACGATTACTCTCGAGGTCGAGTCCAGCGATACCATCGAG
AACGTGAAGCAGAAGATCCAGGACAAGGAGGGTATTCCTCCCGATCAGCAGCGTCTCATC
TTCGCTGGCAAGCAGCTCGAGGATGGCCGCACGCTCGCCGACTACAACATCCAGAAGGAG
TCGACTCTCCACCTCGTCCTCCGCCTCCGTGGTGGTATGCAGATCTTCGTCAAGACTCTG
ACCGGCAAGACCATCACCCTCGAGGTCGAGAGCTCGGACACGATCGAAAACGTGAAGCAG
AAGATCCAGGACAAGGAGGGCATCCCTCCCGATCAGCAGCGTCTCATCTTCGCCGGCAAG
CAGCTCGAGGACGGGCGTACTCTGGCCGACTACAACATCCAGAAGGAGTCGACTCTCCAC
CTCGTCCTCCGTCTGCGCGGTGGCAACTAA
>g414-241703-242847
ATGAACGCTCAGAAGCTCGCTCAGCTCCAGCAGGGAGTCCGGATCGGTGGTAAGGGTACC
CCCAGGAGGAAGCACAAGGCCCCCAGGAAGAAGAACGCTACCACTGATGACAAGAAGCTG
CAGAGCCAGCTCCAGAAGCTCGGCTGCCAGCCCATGCAGGGTATTGAGGAGGTCAACCTG
TACAAGGATGACGGTACCGTGATTCACTTCAACAACCCCAAGTTCCACGTTGGAAGCGGA
GCTACCATGTACGTCGTGAGCGGCCGTGCCGAGAACAAGACCATCCAGGACATCATTCCT
TCCCTCCTCCAGAACTCCGCTCTCGGCCAGGCTGCCGCTGCCGCTGCTGCCGCCAAGAAG
GGTGGTGATGATGACGTGCCCGAACTCGTTGAGAGCTTCGAGTCCTCGTAA
>g414-411909-412468
ATGTCCGGACACAGGCACCTCTCGATCAAGGTCAAGGAGGCCAAGGGCATCCCTGCGGCC
GACTCGAACGGCAAGAGCGACCCCTACGTGGTGCTGACGATCGGCGGCCAGAAGAAGAAG
ACGAAGATCATCCACAAGACCCTGGAGCCCAAGTGGTACGAGGAGTTCCGCTTCGACATC
GACGACTCGCAGCACGTGCTGCGGTTCGAGGTGTTCGACCACGACAAGTTCAGCAAGGAC
GACTCGCTGGGCCACTACGAGCTCAACCTCAAGACGGCCCAGATTCCCATCGGCCAGTGG
ACCCCCTTCACCCGCAACCTCATCCACCCCAAGCAGTCGGGCGAGATTCAGTTCGAGATC
AACATCGCCTAA
>g414-433732-435218
ATGGATAAGCAAGACTGGGGTGATATCGAGGAGGCGGAGACCGACATGACGGGAACCAAG
GTCTTCGAGACGCAGCCCGATGAGCACGGCGTGCGGCTGAAGACCGTGATCGAGTACTCG
ACCAATTCCGAGGGCAAGACCGTCAAGACGACCAAGAACTACAAGCTCTACAAGAAGGTG
ACTCGCATTAACAAGCGGGTCCAGGAGAGGAAGAAGTTGAAGAAGTTCGGCGACTGCCGC
CCGGGCGAACAGGGTATCACACTCCTCTCCAACGATAAGATCAACATCGAGACCCCCGGC
AAGAAGAAGAAGGACAAGGAAGAGGAAGCGCTCAAGGCGTCCGAGATGGCCGAGGCCGGC
ATCAAGGAGACTTGGAAGACCCGCGCTCAGCGTCTTGGCGCTGACAGCTGGGACACTATC
ACCGCCAAGGCTGCCACTCCCGGCGGGTCCGAAGCTGCGGCTGATACGCCGAGCAACGTG
TACGTACCGCGCTCGCTGCGGGGTGGTCCCGGCGGCGCTCCTGGCGCCCAAAGCAGAGAC
GACTCCACAACGGTGCGAGTGACAAACTTGTCGGAGGACACCCGCGAGGACGACCTCAGG
GAGCTCTGCAGGAGGTTCGGCCCCATCCAGCGCGTCTTCCTGGCCAAGGACAGGCACAGC
GGTCTCTCCCGAGGCTTCGCCTTCGTCACCTTCGTTTACAGGGAGGATGGTGCCAAGGCC
ATCGAGGCTCTCAACGGTTTTGGTTATGATCACCTGGTGCTCGCCGTCGAGTGGGCCAAG
CCTTCTGGTCCCGGTTTCTAA
>g414-512928-514286
ATGGCCGAAGAAGTTCCCCAGGAGGTGTTGGCGAAGGCCGACAGCATGGATGACAACAAG
GAGTTCGCGGCGCTCTTCGACTTTCTCCACGAGCAGGCCAAGGCCTACCCGGACAACTTC
GAAATCATGTGGAGAATCGCCCGCGTCAACTTCGACATGTCGTCCGAGAAGCCTGCGCAG
AAGAAGGAGTACATCCTCACCAGCTTCGCCATCACGGAGAAGCTCCTTGTCTTGCAGCCT
GACAACTATCTCTCTCACAAGTGGTATGCCATCGTTTTGAGCTCCCTGTCCGACTTCCGC
AGCACTAAGGAGAAGATCCAGGAGTCCGTCCTCATCAAGGAGCACTGCCTCAAGGCCATT
GAGCTGCACGGCAAGGGTGGCGATCAGACGCTGAACCATTTGCTGGGTCGCTGGTGCTTC
GCCGTTGCCAGCATCTCGTGGATGGAGAGGAAGCTCGCCTCGGCCATCTTTGCTTCCCCG
CCCGAGTCCTCGTACGACGAGGCCCTCAAGTACTTCCTCGGCGCCGAAGGCATCAAGGAG
AACGAAGAGTTCCTCGAGAACCTGCTCTGGACCGGCCACACCTACCACAAGCTCAACAAC
GTGGCCAAGGCCAAGGAGTACTACGCCAGGGCCGCCGCGGTGCAACCCAAGAGCACGTCC
GACATCGCCCTCGTCAATGAGGCCCAGCAGAAGCACGCCCGCCTCTAG
>g414-590528-592322
ATGGCCAAGGTAGGAGAGACCGACCCGAGGTGGATTGTGGCCGAGCGGGCTGATGGCACG
AACGTCAACAACTGGCACTGGACCGAAAAGAACTGCATGCCGTGGGCGAAGGAGCGCATG
CCAGAGCTCTTCGAGGGTGCCAAGATCATTGAAAATGGAGAGGACCTGATTAAGATTACC
AAGGTTGACACGATGAACGGCGAATGCCACATTAACACTCGCAAGGGCAAGATTTTCCAC
TTCTTCGAGTTCGAGATCAAGCTCAAGTGGAGCGGCACCATCAAGGGCGACAAGGTGGAG
GGCAACTTTGACATGCCTGAGATTTCGTTCGAGAACGACATGGACGAGCACGAGATCAGG
GTGAACGTCACCAAGGCGGCCGATAAGCACACCATCAGGCAGCTGGTGGCCAACCAGGGC
ATCCCCAAGGTCAGGAAGCTGCTGGAGACCCTCATCGAGGAGTTCAGGGGCATGCGTGGG
TACCTGCAGGAGGTGAAGGAGGTCAAGCCTTTGACCCAGACCACCGCCGACTCGCCCAAG
CCCGCCCCCGCTCCCAGCCACGCTGCCACTACTTCCACCCCCTCGTCGTCGACCACCACG
GCCTCGTCCTCTTCGGCGAGTGGTGCCAAGACCACCAAGGTGGAGTTTAACGACAAGTTT
GGCGCGCGGCCGAGCGACATCTACGAGGCCCTGATGGACCCGCGGCGAGTCGAGGCCTAC
ACGCAGAGCAGCGCCCAGATGGAGACCAAGGAGGGCGGCAAGTTCGCCCTCTTTGGCGGC
AACGTCACCGGCGAGTTTGTCCAGCTCGTCCCCAACGAGAGGATCGTCCAGAAGTGGAGA
ACCAGCACTTGGCCCGAGGGCCACTATTCGACGGTGACGATCGAGCTGAGCGAGGGCAGG
AAGGGCTGTGACATGAAACTGACGCAGGTGGGCGTCCCCGAAGCTGAAGCAGAGCGCACT
CAGCAGGCCTGGCGGGAGAACATCTTCGAACGCCTCAAGAGGATGTTCGGCTACGGCATG
GGATATTCCCCGTTCTAA
>g414-638194-639630
ATGAGCAGCATCGGCACTGGGTACGATCTTTCGTCCACTACATTCTCTCCTGATGGAAGG
GTGTTCCAAGTGGAGTATGCCGCCAAGGCCGTCGACAACAGTGGCACCGCCCTTGGCTTG
CGCGTGAAGGATGGTGTGGTGCTGGCAGTGGAGAAGCTGCTGGTTTCCAAGATGCTCGTC
CCAAACACCAACAGACGCATCCACACTGTCGACCGACACTGCGGCCTGGCCATGTCCGGT
CTGGTGGCTGACGGCAGGCAGCTGGTCAGCAGAGGGCGCGCCGAGGCGACAAGCTACAGG
GAGTTCTACGGCACCGACATCTCGGGCAAGGTGCTCAACGAGCGACTCTCCAACTTTGTG
CAGCTCTACTCCCTCTACGGCTCTGTGAGGCCGTTCGGTACCTCGGTCATCCTCGGCTGC
GTCGACAAGAACGGACCTCAGCTCTACATGATCGAGCCCTCGGGCATCTCGTGGGGATAC
TTTGGCGTGGCCATCGGCAAGGGTGCCAGAGCGGCCAAGACTGAGATCGAGAAGCTCAAG
CTCTCCGAAATGACTGCCCGCGAGGCCATCAAGGAGGCCGCCAAGATTATCTATTCGGTG
CACGACGATGCCAAGGACAAGGCCTTCGAGCTGGAGCTCAGCTGGGTGTGCGAGGAGACG
GGCAACCTGCACAAGTTTGTGCCAAAGGACCTGCTCGAGGAGGCCGAGAAGTACGCCAAG
CAGGCGCTCGAGGAAGAGGAGGACATGTCCGAGGAGGAGGATTAA
>g414-654698-656112
ATGCAGAATCTGCAGCAGATGCTTCGCCAAATGCAGGGGGGTATGGGCCAGGGCATGCCG
CCTCCTGATTCCCCCGTTGTGGACACTGCCGAGCAGGTCTACATTTCCTCGCTCGCTTTG
CTCAAGATGCTGAAGCACGGTCGCGCTGGCGTACCGATGGAGGTCATGGGCCTGATGCTG
GGCGAGTTCGTTGATGATTACACTGTTCGCTGTAAAGATGTCTTCGCCATGCCTCAGAGT
GGAACGGGTGTCAGTGTAGAAGCCGTCGATCCCGTGTTCCAGACCAAGATGTTGGACATG
CTGAAACAAACCGGCAGACCCGAGATGGTGGTGGGCTGGTACCACTCTCACCCCGGTTTC
GGCTGCTGGCTTTCAGGCGTCGACGTCAACACACAGCAGAGTTTCGAGGCTATCAACGAG
CGTTGCGTTGCCGTTGTGGTGGACCCGATTCAAAGTGTGAAGGGTAAGGTCGTGATCGAC
GCCTTCCGCTGCATCAACCCGCAGACTCTGCTCATGGGCCAGGAGCCAAGGCAGACCACC
TCCAACATCGGACACCTCAACAAGCCCTCTATTCAGGCCCTTATCCACGGTCTGAACAGG
CACTACTACTCGATCGCGATCAACTACAGGAAGGACGAACTGGAGCAGAAGATGTTGCTC
AACGTGCACAAGCCCAAGTGGACCGACGGCCTGCTCCTGGAGGAGTGGGAGGAGCACAGC
AAGCAGAACGAGGCCATCGTCAAGGAGATGCTCGACCTGGCCCAAGGCCTACAACAAGTC
GATCCAGGACGAGATCAACATCCCTCCAGAGAAGCTGGCCATCCAGAACGTCGGCAAGGT
CGACCCCAAGAAGCACCTCGAGTCCGACGTCGAGAAGCTCATGGCCAAGAACGCCACCCA
GCTCCTCGGCGCCATGCTCGACACCGTCGTCTTCTAAGCCGCTCGCTGGCTCCTCCGTAC
GTGTGCCGCTTCGTGACCTCTCTCCCCCCGCGTTCCTCTCTCTGTCATAAGTAA
>g414-688478-690340
ATGGGAAAGAGCAAGACCAACAAGAAGGTGAAGGCTGAGGAGCCTAAGAAGGTGGAGAAG
GTCGAGAAGACCAAGGGAAAGAAGGCTGCCAAGGCCGAGGAGGAGAAGAAGAAGAAGCAG
CAGAAGAAGAAGGAGGAGAAGCCCGCCCCCATGGAGGTCGAGGATGACAGCTCCAGCTCG
TCGGAGGAGTCTTCCAGCTCCGAGGATGAGGCCCCCGCCAAGAAGGCCGCCCCCGCTAAG
AAGGCCGCTGCCAAGAAGGAGTCCAGCTCCGAGGAGGAGTCGAGCTCGTCTGAGTCCGAG
TCCGAGGAGGAGGTGAAGCCCGCCAAGAAGGCCGCCGCCGCCGCCGCCAAGAAGGAGTCC
AGCTCCGAGGAGGAGTCGAGCTCGTCCGAGGAGGAGTCCGACGAGGAGGAGAAGAAGCCC
GCCGCCGCTGAGAACAAGAGGAAGCGCGACGAGGAGGAGAACGGCGATGACTCGTCCACC
GGCGATGCCGAGGAGAGCAACACCAAGAAGGCCAAGACTGACGAGACCCCGTCGAACGCC
AACTTCTTCATCGGCAACCTGCCGTGGAGCGCCGAGGAGGACACTGTGAAGCAGTTCTTC
GAGAGCCAGGGCGTGAGCGCCGTGTACGCCGTGCGCCTCATCACCGACCGCGACACCGGC
CGCAAGAAGGGCTTCGGCTACATCGAGACCTCCGCCAGCGACGTCGACGCCGTGCTTGCC
CTCAACGGCGCTGACTTCGAGGGCCGTGAGCTCAAGGTCGACAAGGCCAACGAGCGCCCT
GCCAACGCCGATCGCGACACCAAGCCCCGCGACGCCCCGCGACAGAGCGGTGAGGCTGCC
ACCGACGGCAACGTCTTCCTGGGCAACCTCTCGTTCAACTCGACCGAGGACAGCATCTGG
GCCGCCCTGGAGCAGTTCGGTACCGTCAAGGCCGTCCGCATCGTCTACGACCGCGAGACC
CAGAGGCCCCGCGGTTTTGGCTACTGTGAGTTCGAGGACGCCGATACCGCCAACAAGGCC
ATCGCCGCCAGCGGCACCGTTGACGTCGACGGTCGTCAGATCAGGATCGACACTGCCACT
GCCAGGTCTGGCGGCGGCGGAGGCGGAGGCGGCGGCGGCGGCGGTTTCCGTGGTGGTCGT
GGCGGCGGTGGTTTCCGTGGCGGCCGTGGTGGCGGTCGCGGTGGTGACCGTGGCGGCCGA
GGAGGTCGCGGTGGTCGTGGCGGTTTCAGGGGCTCGTCCACGCCCTCGTTCGCCGGCAAG
AAGACCAGCTTCGACTAA
>g414-705362-707492
ATGACCCTCCTCGAACTGGATACGTGGACTTCGCCGGTCAATTGGGCGTATCTTTTGACG
TCGGCCTTAGTGGTGTTCTTCCTCGCGGACCACCTGAGCACGCTGAGGAAGCGCAAGAGC
CTCCCGGGTCCGTTGTTCGCTTGGCCCTTCTTCGGCGCCATCTTCGAGATGGTGTGGGAT
CCGACCAACTTCTGGGATCGTCAGGAGACCTTCGGCCCGCTCTCGTGGAATGCGCTCTTC
GGCAAATTCATGGTCTTCTCGCGCGACACCGACACTTCGATCAAGATCTTCAAGCACAAC
AGCCCCGAGGAGCTCAAGATCGTGCTCCATCCCAACGCTGTGCGTCTTCTCGGCGAGGAC
AACATCGCCTTCATGCAGGGCCCTGGCCACAAGGAGCTGCGCAAGCGTCTCCTGCCCCTC
TTCACCAAGAAGGCTCTCGGCGTCTACCTCAACATTCAGGAGAAGGCCATCCGCGAGCAC
CTCGCTAACTGGGTCAAGCTCCCTCAGCCTCAGAAGATGCGCGACCTGTGCCGCGATCTC
AACGTCGATACCTCGCAGTCGGTGTTCATCGGCCCCTACCTGTCGGGCGACGAGAGGAAG
GAGTTCGCCAAGAACTACATGATGATGAACGAGGGCTTCCTGGCCTTCCCGCTGTGCATC
CCCGGCACCGCCCTGTACAGGGCCGTGCAGGCGCGCAAGAAGGTGGTGGCCACGCTGACC
ACCTGCGCCAAGCTCAGCAAGGACAGGATGAGCAAGGGCGTCGAGCCCGAGTGCCTGTTG
GACTTCTGGATGGAGAAGACCGTCGCCGAGATCCAGGAGGCCAAGGCCGCCGGCGCGCCG
GCCCCGCCGCACAGCTCCGACCACGAGGTGGCGTGCACCACGCTCGACTTCCTCTTCGCC
TCGCAGGACGCCTCGACCGCGTCGCTCGTGTGGACTTGCGGCCTCGCCCTCACCGGCAAC
CCCGACGTGCTCGCCAAGGTGCGCGCCGAGCAGCTCAAGCTGCGCCCCAACGACGACCCG
CTCACCACCGACGTGCTCGGCGACATGCCCTACACCAACCAAGTGGTCATGGAGCTCCTC
CGCTACCGCCCGCCCGCCACCCTCGTGCCGCACATCGCCCTCAAGGACTTCCAGCTCACC
GACAACGTCAACGTCCCCGAGGGCTCCCTCGTCGTTCCCTCCGTGTGGGGCGCCTGCCAC
CAGGGCTACACCAACCCCAACACCTTCGACCCCGACCGCTTCTCCCAGGAGCGCGGCGAG
GACCGTAAGTACGGCAAGAACTTCCTCACCTTCGGCAGCGGCCCCCACTACTGCCTCGGC
AAGCAGTACGCCATCAACCACTTGATGTCCTTCCTCTCCATCATGTCCATCAACGTCGAC
ATGAACCGTCACCGCACTCCCGAGAGCGAGACCATCGTCTACGGCCCCACCATCTTCCCC
GCCGACGGTGTCATCGCCGACATGAAGGAGAAGAAGTACTACTAG
>g42-16836-17460
ATGCCTAAGAAAGAGAATAAGGGTGTGAACAAGAACACCGTCCTCGCCCGGGAGTACACC
ATCAACCTGCACAAGCGCACGCATGGCCTTCAGTTCAAGAAGCGCGCTCCCCGTGCCATG
AAGGAGATCAAGGCCTTCGCCCGCAAGGCCATGGGCACCTCCGATGTCCGCCTCGACGTC
AAGCTCAACCAGTTCGTGTGGTCGCAGGGTATCAGGAACGTCCCCTACCGCGTCCGCGTG
CGCCTCTCCCGCAAGAGGAACGAGGATGAGGAGGCCAGCGAGAAGATGTACACGCTCGTC
TCTTATGTGCCCTGCAAGGACTTCAAGGGTACCGTCACCCAGGTCGTGGAGGATGTGTAA
>g42-42086-43890
ATGTCGGACAAAGAAGCGCTGTTGGAATGGCTGGGCAACGTCAAATGGGAAACCTACCTT
GACGATGAAGAAGTGAAGGAGAAGGTCACTGAGTTGAAGGACAAAATCGAAGGCCTTGGT
GAGCAAGTGAACATTGCTGGGGCTACTCGCATCAAGCTTGTAGTCGTCGGTGATGGCGCT
GTCGGTAAGACTTCTCTTCTCATCTCGTACGCCACGAAGAAGTTCCCCACGGACTACGTG
CCGACCGTGTTCGAGAACTACACCGCCCAAATGAAGAGCGACAATGAGAACATCCTTCTC
CATCTTTGGGATACTGCCGGCCAGGAGGACTACGACAGGCTCAGGCCTCTCAGTTACCCT
GGTGCCGACGTGGTTTTGCTGTGCTTCTCCACCGTCAACCGAGCCTCCTATGATGCGATC
AGGGAGAAGTGGTACCCAGAGGTGAACCACTACGTGCCCAACATCCCCCATCTTTTGGTG
GGCACCAAGGTAGATCTTCGAGACAGCGAGACTGCCGACCCACACACCACACAATACGAA
CCCATCACCTCCGAAGAGGGCAAGGAGATGGCGAAGAGCATCAGCGCGGCGAGCTATCTC
GAGGTGTCGGCCAAGACGAGGAAGGGGCTGGACAAGGTGTTCAGCGTCGCGGTGCACTGC
GTGCAGCAGGCCAGGGGCGTCACCCCGGCCGCTGACGGCGCCGCCTCGGGGGCCGACGTC
GCTCCCGTGGTCGTCAAGAAGAAGAACAAGAGCAAGGGCTGTTTCCTCATGTAA
>g42-50626-52122
ATGGCGGATGCGTCTGGATCTGAATCGGGGCAGTTGCTCAGGTACATCGTGCTCGCGAAG
AACACGAAGGGACGAGGTGCTGTGGGTGTCATCCAGCAGGCTCTTCAGGCCCCCAACACC
TTCGTTTTTGGCGAGCTGCTCGACGTAGCAAACATTAAGGCATTGGCCGAGGGCGAGTTC
AAGGTGTGGTATGACACCCTTGCGCTCTTCGCCTACGGCACCTTCTCCGACTACAAGGCC
GCGCCCCAGAACTTTGGTGAGCTCTCGGCTCCTCTGCAGAAGAAGCTCAAGCAGCTCTCC
ATCGTCGCCCTCGCCTCCAGCCAAAAGAGCCTGCCGTACTCGCTGCTGCTGGCGCAGCTC
GACATTGGCAACGTGCGAGAGCTGGAGGACTTGGTGATCGAGTGCATCTACGCGGGCATC
ATCAAGGGCAAGCTCGACCAGAAGGAGCAGCGCTTCCAGGTCGACTGGACGATGGGCAGG
GACATCAGGCCCGGCCAGCTCCAGGAGATGATCAAGATCCTCGACCTCTGGTGTCAGAAG
TCGGAGATGCTGATGGGCGAGATTCAGGAGCGGATCCAGTACGCCAACCTGTCGCACGAG
GAGCACACCAAGCAGACGAAGGAGTTTGAGCAGAGGGTGGAGGAGATCAAGTCCAACCTC
AAGGTCGCCATGGAGGGCGACATGCCCGCCGGCGACTTTGCCGATGTCGACTTTGGCTTC
GACTCGGCCAACAAGAAGGGACGCGGCAAGGTCAAGGGACCCGGCGCTCCGCTGGACCGG
CTCAGGAGGAGGTAA
>g42-72077-72658
ATGGGAAAGGGAATTCACCTGCCGGCGTCTCTTAGGTCGTTCCTCTACCGACAGATCACC
GTCATCACCGTCGGATCGGTGGTGGTCGGAACCGCCTTCGGCGGCTGGCAGAGCTACACG
ACGCGTGTGGCCGATGAGAACATGCGTCAGATCCGTTTCAAGCAGGCCGAGAAGGAGGCG
CGCATCTCGCACACTCTTCTCACCAAGGGCGGACTCAAGAAGGCCCTCGAGGAGCAGGGT
TTCGACATGGGCTTCTACGAGGTCGATGAGGCCGATGAGGAGGAGATCCAGTAA
>g422-12926-13547
ATGTCGAAGCGCCTGATCACCAGAGCCACCCTCAACGGCCGTCTTGCCACCGCGTGGGCT
CCCAGCAGGAGCTACGCTGAGGCCGCCGTGCCCAAGCTCGGCAAGGACATTCAGCCCAAG
AACTACCGCAGGCCTGACGGCACCTACGAGCCCTGCGATGAGATCCTCTTCCTCTCCGTT
ATCGGCTACGGCGAAGATCTTCGCGCCGTGACCAACGCCAAGGAGGGCATCACCAACACT
CTCGTGCTTTCGCGCTACAACAAGACTGCGTCGCAGTAA
>g422-158974-161348
ATGCGGCCGGCGCAGGGTGACCCTCACCTCCGGCCACCACTCGCCTCCCTCTTCTTCTTT
GCTTCTCGCCACTTCGAACCCACCCCTCCCCCCTTTCTTCTCCTCCTTCAGCTCATCATG
GCCGCCACCTCTACGTCGAGGCCGGGCCTTATCGGATCCATCTTCGCCCTCGTCATGTCG
ATCTTCTCGTCCATCTTCAACTGGATCGTGGCCCGTTGCTACACGTCGCAAATCGTGTTC
AACTGCGCGTGGGAGGACCCCAGGCTCGACCGCGAGGCCCTCAAGCTCACCGAGGAGGAC
CAGGTGGTCATCATCACCACGGCCGGCTGCAACGTCCTGTCGCTCGCGATTGAGGGTCCT
AAGCACGTCTACAGCATTGATCGCAACCATTGCCAGAACGCCCTCCTCGAGCTCAAGCTC
GCCGCCATCCGCGAGTACGACTACGCCACCTTCTGGAAGCTCTTCGGCACCGGTCGTCTG
CCCAACTTCTCCAAGGTCCACTACCCGCGCCTGAGGCAGCACCTCTCGACCGCTGGTCGC
GCCTTCTGGGACTCGCATGCCCACTACTTCAACGGCACTGGCCTTCGTCCCTCCTACTTC
TGGCACGGCTGCAGCGGCATCCTCGCGTGGGTGATGCTGGGCTACTTCCGTCTCATCGGC
GTCTACCGTCCGCTCCTGGAGCTCATGGAGGCCAAGACCATCGACGAGCAGAAGCACATC
TACAAGACCAAGATCGAGAAGAGGATGTGGAACCCCGTCATCATGTGGTTCCTCGAGCGC
AGCTTCACCCTCGCCCTCCTCAACGGCGTGCCCGAGGCCCAGCGCGAGCTCCTCGAGAAG
GAGGGAGGCTTCAAGTCGATCGGCCTCTTCATCAAGGACTCGATGGAGACCATCATGACC
AAGCTGCCCATCCACGACAACTACTTCTACCGCGTCTACCTCACCGGTGAGTACACCAAG
GACTGCTGCCCCGACTACCTCACCGAGGAGGGCTTCAAGACCCTGAAGAACGGAGCTGTC
GACAAGATCTCGATCCACACCACCACCATCACCGAGTTCCTGAGGGAGCACAAGAAGAAG
GACATCAGCCGCTTCGTGCTCCTCGACCACATGGACTGGATGGCCTCGGCTCCCAAGGCC
CTGAGCGAGGAGTGGTCCGAGATGGTCGCCCACTCGACCGACAACTGCAGGTTCCTGTGG
CGAAGTGCTAGCAAGGAGGCCACCTTCGTCGGCGACACCAGCGTGACCCTGCCCAACGGT
CACGAGACTGTCACTGTCAAGGACGTCCTCAAGTACGACTACCAGACCGCCAACAGGCTC
CACGAGTTGGATCGCGTCCACACCTACGCCTCGTTCTTCATCGCCGACCTCAGCCTCTGA
>g422-210135-211206
ATGCAAATCTTCGTGAAGACCCTGACTGGCAAGACCATCACCCTCGAGGTTGAGTCCAGC
GACACCATCGAGAACGTGAAGCAGAAGATCCAGGACAAGGAGGGTATTCCCCCCGACCAG
CAGCGTCTCATCTTCGCCGGCAAGCAGCTCGAGGACGGTCGCACGCTCGCCGACTACAAC
ATCCAGAAGGAGTCGACTCTCCACCTCGTGCTCCGCCTCCGTGGTGGTATGCAGATCTTC
GTCAAGACTCTGACCGGTAAGACGATCACCCTCGAGGTCGAGAGCTCCGACACCATCGAG
AACGTGAAGCAGAAGATCCAGGACAAGGAGGGTATTCCCCCCGACCAGCAGCGTCTCATC
TTCGCCGGCAAGCAGCTCGAGGACGGCCGTACCCTCGCCGACTACAACATCCAGAAGGAA
TCGACTCTCCACCTCGTCCTCCGTCTTCGCGGAGGCGGCAAGAAGACCAAGTGCTGCTTC
GGCGAGTGCTCCAAGCGCGTCGTTCTCATCACGGGCGATTGCCGCTTCTGCCAGTCGCAG
TTCTGTGGCTCGCACCGCCTCCCCGAGGATCACGCCTGCCAGAGCATGGACTCGTGCCGA
CAGGCGGCCTTTGATCGCAACAAGAACAAGCTCATGAACGAGAAGTGCGTAAGCCAGAAG
GTCGGTGGAGTGTAA
>g422-218714-220268
ATGTTTCTTACCAGGACTGAGTACGATCGTGGCGTGAACACCTTCTCCCCTGAGGGTCGT
CTCTTCCAGGTCGAATATGCCATTGAAGCCATCAAGCTGGGCTCCACTGCGATTGGCATC
CAGACTTCTGAGGGTGTGATCCTGGCTGTGGAGAAGCGCCTCACGTCGCCCCTTCTCGAG
CCCAGCAGCGTGGAGAAGATTATGGAAATCGACGCTCACATCGGTGCCGCCATGAGCGGT
CTCACTGGAGATGCCAAGACCCTTATCGACTACGCCCGAGTTGAAGCACAGAACCACACC
TTCACGTTCGATGAGCCCATGTCGGTCGAGTCGTGCACGCAAGCCATCTGCGACCTCGCC
CTCCGCTTCGGCGAGGACCGCAAGTCTGAGAAGGCCATGAGCCGACCGTTTGGTGTGGCC
CTGCTGGTGGCGGGCGTGGACAGCGAGAACGGTCCGGCCCTCTTCCACACTGACCCGTCG
GGCACCTTCGTCCAGTACGAGGCCAAGGCCATCGGCGCCGGCTCCGAGGGCGCCCAGTCC
ACGCTCCAGGACAAGTACTCCAAGTCGATCACGTTGAAGGAGGCGGAGAAGCTGGCGCTG
CACACGCTGAAGCAGGTGATGGAGGAGAAGATCAGCGCCGTCAACGTCGAGATCGCGTCG
GTCGAGGCGGGCAAGACGTTCCGCATCTACTCGGCCCAGGAGCTCGAGGCCATCCTCGCC
CAAATCGACGACGGCTCCTCGTCCTCGTCTTCCTCTTCGTAA
>g422-360514-361158
ATGTCCTGGAGCGCACTCAACCGCAAGGCCCCCGGCGTCTTCAACGAAGGCACTAAGCCC
GTGTGGCTCACCCTGTTCCCGCTCATCGCCGGTGGTTGCTTGTGGGCTGGTTACGTCAGC
ACCAGGACCCTCTTCGGCCACAACGAGATTGTGCTCTCGTCCAAGGGTCAGGAGCCCTAC
CTCGAGAGGGACTCGCCCAAGCTGCTCGGCCGCGACAGGAACGTCTCGACGCACGCCAAG
TACAACGCCATGATGGACGGCAAGCAGTAA
>g422-365974-367525
ATGTCTCTTCCTACGATTGAAGAGCTGGAGGACCTGACCTCGCAGTGCCTGGCGAGGACC
TTCTCGGACCAGCAGGTGCAGGCCATCATCAGCCACTCGCGCCAGCTGGCATCGTTCGGG
GTCACCGACTACGTCGATCGCGAGGACCTGCGCAAGATCTTCAAGGACGAGAAGGACAAG
AAAGACAAGAAGAAAGACAAGAAGAAGGACAAGAAGGATAAAAAGAAGAAGAAGGGCAAG
AAGGACAAGAAGAAGGACAAGAAAAAGAAGAAGAGCAAGAAGGACAAGAAGAAGAGCAAG
AAGGACAAGAAGAAGGACAAGAAGCATAAGAAGAAGGACAAGAAGGACAAGAAGAAGTAC
AAGGAGGGCTACAAGGAGCAGCCCTGGAGGCCTGCCGAGTGGAGCCTCAGCAAGGAGGCC
AGCGCCGAAGGCAAGGAGTACGACTTCGACGAGGACGAGACCCGCTACGTGCGCACCAGG
CTCCAGGAAGTCAGAGAGAAACTGCTGCACAGGCGCGAGGCCAGCGTCAGCAACTTCGCC
CCGTGCATGCTGCTCTACACCGAAGGCAGCGAGCCCGACGTCAAGGAGACCAAGCTTGAC
GCCCAGTGCACCCTGCTGAGGAAGGCCAGCACCCACGCCCTCAAGAGCGAGCAGTGCTGG
TCGCAGTACCTGGCCAAGATGGACGAGTGCCTCCACCACCGCACCGGCGAGTACTGCCTC
CGCGCCTTCCGCCCTCTCGAGAGGCAGTGCGCTCAGTCTGGCTGGAGGAGCGTCAACGAC
CTGAGGAAGCTCACCAGGCTGCGCTCCAAGGAGGAGGTCGACCTGCAGTAG
>g422-375474-377816
ATGGCTGCCAACTCTCCCTACCAGTCTGCTTCGCTCTACGTGGGTGATCTCAACCCCACC
GTTACGGAGGCTCTCCTCTTCGAGATCTTCAAGGCCGTCGGACCCGTGGCCTCCATCCGC
GTGTGCCGCGATGCGGTGACGAGGCGCTCGCTGGGCTACGCGTACGTCAACTTCCACAAC
GTGGTCGATGCCGAGCGCGCCCTCGACACGCTCAACTACACCCTGATCAAGGGCCGCCCG
TGCCGCATCATGTGGTCGCACCGCGATCCGTCCATCCGCAAGTCGGGCCAGGGCAACATC
TTCATCAAGAACCTCGACAAGAGCATCGACAACAAGGCGCTCTACGATACGTTCTCGGCC
TTTGGCAACATCCTGTCGTGCAAGGTGGTGACCGACGGCAAGGGCAACAGCAAGGGCTAC
GGCTTCGTGCACTACGAGACCAGCGAGGCGGCCGACTCTGCCATCGCCAAGGTCAACGGC
AAGATGCTGAACGGCAAGATCGTCTACGTCGGCCGATTCATCGCGCGCAAGGAGCGCACC
CCCGGCAGCGACCCCGAGAAGTTCACCAACATTTACATCAAGAACCTCGGCGAGGCCTAC
ACCGAGGAGGACCTCAAGCGCGACTTTGGCGCCTTCGGTACGGTGCAGAGCGCAGTCCTG
ATGAAGGACCCGCGCGACATCGGCCGCCAGTTCGCCTTTGTCAATTTCGAGGATCACGAG
GCCGCCCACCGCGCCACCGAGGAGCTCAACGGCCGCAAGCTCGGCGACAAGGAGGTCTAC
GTCGGCCGCGCCCAGAAGAAGTCCGAGCGCGAGTCCTTCCTCCGCAAGCTCCGCGAGGAG
CGTGCCCAGAAGTACCAGGGCATCAACCTCTACATCAAGAACCTCGATGACACCGTCAAC
GACGAGGAGCTCCACAAGCTCTTCTCGGCGCTCCCCTTCGGCCAGATCACCTCCTGCAAG
GTCATGTCCGACGACAAGGGCAACTCCCGCGGCTTCGGCTTCGTCTGCTACACCAACCCC
GAGGACGCCTCCAAGGCCGTCTCGGAGATGAACGGCAAGATGGTCGCCAACAAGCCCATC
TACGTCGCCCTCGCCGAGCGCAAGGACGTACGCTCCGCCAAGCTCGCCGCCCAGCACGCC
GCCCGTGCCGCCGCCCGCGTCGCCGGCAACGCCGGCCTCGCCGGTGCCCCCATGGCCTAC
CCGGGCTTCTACGCGCCCCAGGGCGCCCCCCAGGCCCAGCGCGCCGCCCCGGCCGGCTTC
GTCGGCTACCCGCCCCAGGGCGTGCCCGTCCGCCGCGGACCCTGGACCGGCGCCCCGCAG
GCCGCCGGCCCCCACGCCCAGGGCCGCCAGTACCAGCCCCTCCCCGGCTACGTCATGCCC
GTCCAGGGCGCCCGCCAGCCCCACCGCCAGCCCCGCCCCGCCGCCGGTGGCGCGCCCGCC
GCTGCTGCCGGTGCCGCCCCCGCCCAGGGTGCCGTTCCCGCTGTCGCCGGTGGTCAGCCC
AACGGTGCCGCCGCCGCCGGTGGTCGCAACTTCAAGTACACCCAGAACGCGCGCAACCAG
CAGGCCGCCCAGGCTGCCGCCCCGGCCGCCGCTGCGGCTGCCCCCGCCGGTGCGGCCAAG
CCGAGCGAGATCGCGGCTCTCACGTCGCCGTCGCTGGCTTCGGCCTCGCCCGAGGAGCGC
AAGAACATCATCGGTGAGACGCTCTACCCGCTCGTGGAGGCCCTCACGTCGGCCACCCAG
GGCCCCAAGATCACCGGCATGCTGCTCGAGTCGATGGACGTCACCGAGCTCCTCCACCTG
CTGCAGTCCCCCGACGCCCTCCGCGAGAAGGTCGACGAGGCCGTCGGCGTGCTCAAGGCC
CACGAGGGCGAGGAGAACGGAGATGCCCCCAACGAGAGCGCTTGA
>g422-377924-379281
ATGAAGCTTTCTCTCCTCCTCGTTGCCGTTCTCTGCGTTTTGAGCGTGCTCGCTCAGGCT
GAGGCGGACAAGAAGGGACCCAAGATTACCAACAAGGTCTACTTCGACATCGAAATCGAT
GGCAAGCTTGAGGGTCGTGTGGTGTTCGGTCTGTATGGCGGCACCGTGCCCAAGACCGTC
GAGAACTTCCGTGCGCTGTGCACTGGCGAGAAGGGCGTCGGCAGCAAGGGCAAGCCGCTC
CACTACAAGGGCTCTTCCTTCCACAGGGTTATCCCCAACTTCATGATCCAAGGCGGCGAC
TTCACCCACGGCACTGGTGTGGGCGGTGAGTCCATCTACGGCGACAGGTTCGCTGATGAG
AACTTCAAGCTGAAGCACACCACCCCCGGTCTCCTCAGCATGGCCAACGCCGGCAAGGAC
ACCAACGGCTCGCAGTTCTTCATCACCACCGTGGCCACCCCCTGGTTGGACGGTAAGCAC
GTCGTGTTCGGCAAGGTGCTCGAGGGCTACGACATCATCAAGAAGCTCGAGTCGCTCGGC
AGCCAGTCCGGCAAGCCCAGCGCCACCCTTACCATCGCCGACTCTGGCGAGCTCCCCCTC
TCCGAGTAG
>g422-496887-497476
ATGCCCAAGAAGTTCACCATCAAGACTCACCACGGCAAGTACATCTGCAGCGAGCCCTCG
CACACCATTGTGGGCAACCGCGACTCGGCCGATGCCTGGGAGCACTGGGAGGTCATCAAG
CTCGGTGGCGGCAAGGTCTACCTGAAGTCGGCCCACGGCAAGTACCTCTCGGCCGAGACC
AACGGCACCGTCGTGGCCAACCGCGACACCCCCAAGGAGTGGGAGACCTTCCACGTCCAC
AAGCTGGGCAACAAGAAGGTGCAGTTCAAGACCCACCACGGCAAGTACCTGTGCGTCGAG
CCCAGCGGCAAGGTCATCGCCGACCGCGCCGCCCCCAAGGAGTGGGAGACCTTCGAGTTC
CGCAAGGTCGGCCACTGCTGCTAA
>g422-581324-583206
ATGGGAGACGAGGTTCAGGCTCTGGTTATCGACAACGGTTCGGGCATGTGCAAGGCTGGC
TTTGCCGGTGATGACGCCCCGCGCGCTGTGTTCCCCTCGATCGTCGGTCGCCCGCGCCAC
ACCGGCGTGATGGTCGGCATGGGCCAGAAGGACTCGTACGTCGGTGACGAGGCCCAGTCC
AAGCGTGGTATCCTCACCCTCAAGTACCCCATCGAGCACGGCATCGTCACCAACTGGGAC
GACATGGAGAAGATCTGGCACCACACCTTCTACAACGAGCTCCGCGTGGCCCCCGAGGAG
CACCCCGTGCTTCTGACTGAGGCCCCGCTCAACCCCAAGGCCAACCGCGAGAAGATGACC
CAGATTATGTTCGAAACGTTCAACNNNNNNNNNNNNNNNNNNNNNNNNNNNNNNNNNNNN
NNNNNNNNNNNNNNGGATGGCCGCGATTGGGAAAGGAAAGCGTTCGCGGCCCGCCGATTA
CAGACCAGGCTCGAGAGAGTGGGCCAGTCCATAAAGGACGAGAAGACACTGCTACGGTTC
CGGAATCCGTTCGGCATCGCCCAGTACATCAACCACCCTCCGGGAGGCAAACAGCCGAAT
GTGATGTGCGTTGGCTACGAATTCCCCAACACTTTTCCTGACGAGCTCAAGCCCTACATT
CCTCACGAGCACGTCAAGGAACCCAACATCTTCTTCAACACGATGACCGATTGCTACATC
CGGTCGATCATGTGTATCGCCACCCGGCACATCACCGATGAGGAGTTGTTCCTCAACTAC
CGGTACAACCCGATCAACCCGTATCCGGACTGGTATGTGCAACCCGACGTGGCGGAGGCC
GAGCGGCGATGGAAGAAGCCCAAGGCCTTGGCCAACCCCTTCTGGTAG
>g422-624234-625070
ATGCAGATCTTCGTGAAGACCCTGACCGGCAAGACCATCACCCTCGAGGTCGAGTCGAGC
GACACGATCGAGAACGTCAAGCAGAAGATTCAGGACAAGGAGGGTATTCCTCCCGATCAG
CAGCGTCTCATCTTCGCCGGCAAGCAGCTCGAGGACGGCCGCACGCTCGCCGACTACAAC
ATCCAGAAGGAGTCGACTCTCCACCTCGTGCTCCGCCTCCGTGGTGGTATGCAGATCTTC
GTCAAGACTCTGACCGGCAAGACCATCACCCTCGAGGTCGAGAGCTCGGACACCATCGAA
AACGTGAAGCAGAAGATCCAGGACAAGGAGGGTATCCCCCCTGACCAGCAGCGTCTCATC
TTCGCTGGCAAGCAGCTCGAGGACGGCCGCACGCTCGCCGACTACAACATCCAGAAGGAG
TCGACTCTCCACCTCGTTCTTCGTCTCCGTGGTGGTATGCAGATCTTCGTCAAGACGCTG
ACCGGCAAGACTATCACCCTCGAGGTAGAGTCCAGCGACACGATCGAGAACGTCAAGCAG
AAGATTCAGGACAAAGAGGGTATTCCTCCCGACCAGCAGCGCCTCATCTTCGCCGGAAAG
CAGCTCGAGGACGGCCGTACCCTGGCTGACTACAACATCCAGAAGGAGTCCACGCTCCAC
CTCGTCCTCCGTCTCCGTGGTGGCAACTAA
>g422-625093-626146
ATGTCGCTCAACGTGACGGAGGAAATCACTATCGATGCGCCAGCTGACGCTGTCTGGAAG
GTGGTTGGCGATTTCTTTGGTCTTAGCCAGTGGCTCCCCACCACCACCTGCGAGGAGACC
GATGCTGTCGATGGCAAGAAGACCCGTCTTATCACCATGACGGTTACTGGCGCCAAGCTT
GTCGAGGCGCTTGACGAAGTCAATGAGGAGGCCAAGAGCATCAAGTTCCACGTTGTCGAG
AGCCCCTTCCCCATCACCAACTGCAACACTGAGATGAAGGTGCACGATTTGGGAGATGGC
AAGAGCAAGTTCACCTGGGCCAGCGACTGCGACCCCAAGCCCGGCACTGGCGACAGCGTT
AAGCCGCTGCTCGCTCAGCTCTACAAGGGTGGCAATGGCGCTCTCAAGGCGCACATGGAA
GAAGCCTCCTCGTAA
>g422-692726-694076
ATGACTGCCGTCTTGAATTCGTTGGGTCTGGAGACGCTCGAGGAGCTGTACTACCATGAC
GATCCCCACGCCGCGAAGGAGGAGAAGAAGAAGCTGAAGAAGAAGGACATCGTGGATGCC
ATCAGCGACAAGATCGACACGTACGCCATCGAGGTCTTCATCGCGTCGCTGCCGCTCGCG
CAGCTCAAGACGGTCGCCGAGAAGGCGAAGGTGCCGTTCAAGGAGGAGGACAACAAGAAC
AGCCGATCGGTGCTGTCCAGGCGACTCACCGAGCAGGTCGTGTCTGCGGGCTTCGATGAA
TTCCTCGAGACCGTCGATGCCGATACGCTCAAGGCCGTCGCGGAGGACTTTGACGTGAAG
GCCGACAAGAAGGCGATCGCTGAGGCCGCTCGCACCTTCGCCGCCGAGAGGTACTTTGGC
AACTTCGATGTCGAGTCGCTGCGCGCGCTGGCCGAGGAGCTCAAGCTCAAGCAGGCCGCC
AAGACCTCGAGCAAGAGGAAGCTCGTCGAGGCCATCGTCACGCAGGAGGATGCCGAGCCC
GTCGAGGCCCCCAAGAAGAAGAAGCAGAAGACCGATGTGGGCAAGAAGAAGGCCCTCGAG
AAGGGAATCACCTACGACGAGATCTTCCAGCACTACTACGTCGAGGAGCTCAGGGACTTC
GTCAAGTCCAAGGGCATCAAGGTCTCGGGCAAGAAGCCCGTCCTCATCAAGCGCATCCTC
GCCTACCTCGCCGGCGAGACCGAGGGCATCATGGCCGGCGACAAGACCGAGAAGGCCAAG
AAGAAGAAGAAGGCCACCACCAAGAAGGCTGCTGCCGCCAAGAAGGGCGCCGCCTCCACG
AGCGCCAAGGGCAAGGGCAAGAAGGAGGAGAACGGCAACGGCGACGAGTAA
>g430-7946-10549
ATGGCCGCCCCTCCCATGCAGGCTCCCATTCTTGTGCTCACCGATAACACGAAGCGCGAG
AGCGGTCGTAAGGCGCAGAGGAACAACATTGCCGCTGCCAAGGCCGTCTGCAGCGTTGTG
CGCACAGCTCTTGGCCCGCGCGCCATGCTCAAGATGATTCTCGACTCCATGGGCGGCATC
GTTCTCACCAACGACGGCAATGCCATCCTCCGCGAGCTCGAGGTCAGCCACCCGGCCGGC
AAGAGCATGATCGAGCTCAGCAAGACCCAGGATGAGGAGGTCGGCGATGGCACCACCTCC
GTCATCATTCTCGCCGGCGAGATGCTCACCGTGGCAGAGCCCTACCTTGAGAAGAACATG
CACCCTACGGTCATCATCCGTGGCTTCTTCAAGGCCAGGGACGACGCCATCGAGGCCCTC
GACAAGCTCGCCATCAAGGTTGACATTTCCAAGCGTGAAGAGCTTTTGAGCATTATCAGG
AGCTGCCTCTCCACCAAATTCGTCAGCCGCTGGATGGATCTTATGTGCGACCTTGCCCTC
GATTCCGTCTCCGCTGTTGTCCACCAAGATGGCAACCGCAAGGAGATCGACATCAAGCGC
TACGTCAAGATTGAGAAGGTGCCTGGTGGCGAACTGGAGGACAGCTATGTGCTCAAGGGC
GTGATGCTCAACAAGGATGTGCTGCACTCGAAGATGAAGCGCAGGATTGAGAACCCCCGC
ATTCTCCTCCTCGACTGCCCCCTCGAGTACACGAAGGGCGAGAACAACATCATGATGGAT
GTCACCAAGGAGGGCGACTGGACGGCGATCCTGAGGGCCGAGGAGGAGTGGGTCAAGAAG
ACTTGCGACCACATTATCGCCCTCAAGCCCGACCTCGTCATCACGGAGAAGGGTGTCTCC
GATCTTGTGCAGCACTACTTCGTGAAGAACAACATCACTGCGCTCCGCAGGCTCCGCAAG
ACCGACAACAACAGAATCGCCCGAGCTGTCGGCGCCGTCATCGTGAACAGAGTCGAGGAG
GCCAAGGAGTCCGATCTCGGTACCAAGTGCGGACTCTTCGAGGTCAGGAAGATTGGTGAT
GAGTACTTCTCGTTCATCGAGGACTGCAAGGAGCCTAAGGCCTGCACCATTTTGTTGCGC
GGCGCCAGCAAGGACGTCCTCAAGGAGGTTGAGAGGAACCTCCAGGACGCCACCTGCGTG
GCCCGTAACATCCTGCTGGACCCGCGTCTGTGCCCCGGCGGTGGTGCCTCCGAGATGACC
ATCTCGCAGGCCCTCCGGGAGAAGTCGAAGGCCATCGAGGGTGTCGAGCAGTGGCCCTAC
ATTGCCGTCGCCAACGCTCTCGAAGTCATTCCCAGGACCCTGGCGGAGAACTGCGGCGCG
AGCGTGGTGCGCGTGCTCACGGAGCTGAGGGCCAAGCACGCCGAGGGCAAGAACCAGTCG
TGGGGCATTGACGGCGTGAAGGGCACCCTGGCCGACATGATGGGCCTCGGCGTCTTCGAG
CCCTACTCGGTCAAGGCGCAGACCATCAAGACCGCCATCGAGGCCGCCTGTCTTTTGCTT
AGAGTGGATGACGTGGTCTCCGGCATGAAGAAGAAGGGCGGCCAGTAA
>g430-16557-17408
ATGAAGAGGCTCACCATCGCTCGTCGCCCCTTTACTTTTGCTGGGGCCACTCCCCTTTAC
CGCTCGGCGGCTCCTCTGTCGTCCCAGCAGCGCTTCCTCAAAGACATTGCCTCCAATTCG
GCCAATGAAGCGCCCCCGAACACGGATGCCGCTCAGGTCCGCCTCCACATGGCGATCAAG
GGCAATGCCGACGAGGTCTCCGAGGAGAGGCGCAAGAGCATCTCGGCCGTTATGGGCACC
CCCGTCGAGCACCTCAAGCGCCGTGTGCGCATCTACATCCCGGCCCGGTGCACAATGCAG
TCGGGCGTCCATTCCGCCACGTTTTGGAAGATTTCGTATCCCGAGGAAGAGAAGAACCAT
TGGGCCAACCCGCTCATGGGCTGGACCGCGACCAAGGACCCCGTCTCCAATCTCCACGTC
AAATTCGACACCAAGGAAGCCGCCGTCGCCTTCTGCAAGCAGCACGGACTGGACTACGAG
ATCGAGGACAGCACGACCCCGTACGAGAACATCACGCCCAACAAGGACTACGCCGACAAC
TTCAAGTTCAAGGTCGAGCGCAGCCTCGAGCCCGACATCTACTGA
>g430-19193-19854
ATGTCTTTTTGGGGCGGGGGCTCGCGACGTTCTTTCTTCCTTTCTCTCCCACATCGTCGG
CCTTCCCTCTTTCGCTGTCGAAGAAGATCAACCATGGGTCACGCCGCCGTCTGGAACTCT
CACCCCCGCAAGTTCGGTCCTGATGCCCGTAGCTGCCGCCGCTGCGGAAACAACCACGGT
CTCATCCGCAAGTACGGCCTCATGATGTGCAGGAGGTGCTTCAGGGAGCGCGCTGAGGAG
ATCGGTTTCCTCAAGATGAGGTAA
>g430-55226-55970
ATGGCCGACGATGGTGAATTGGACACGGTGAAGCTCATCTCAGCCGACGGCCACGAGTTC
GTTGTCGATCGCAAGGCTGCCATGGTCTCGGGCACCATCAAATCCATGTTGTCTGGCCCT
GGTACGTTCACCGAGCAGGCCATGGGTGAGATCAACTTCCGTGAGATCTCTACGCCCATC
CTTGAGAAGGTTATCCAGTACTTCTACTACAAGCTGCGCTACACCAACAGCACGACCGAG
ATCCCCGAGTTCCCCATCGAGCCTGAAGTTGCCCTGGAGCTTCTCATGGCTGCTAACTTC
TTGGACACTTAA
>g430-66903-68568
ATGACTGAGAAGGAGGTCAAATGCGTTGTCGTCGGTGACGGCGCCGTCGGTAAGACGTGC
ATGCTCATTTCCTACACCGAGAATCACTTTCCTGTCGAGTACGTGCCGACCGTATTCGAT
AATTATGAAGCGCAGATCCTCGTGGAGGGCCAGGAGGTGAAGTTCTCGCTCTGGGATACC
GCTGGCCAGGAAGGCTATGCGCGCATCAGGACGCTCTCGTACCCGAAGACGGACATCTTC
CTCCTCTGCTTCTCCGTCGTCAACCCCTACTCCTACGAAAACGTCAAAGAGACCTGGCTC
CCCGAGCTGAGGCATCACTGCCCGACGACGCCGATCATTCTGGTGGGCACGAAGATCGAT
CTGAGAGAGGATGCCAAGACCCTCGAAGAGCTCCAAAAGAACAAGAAGGACCCCATCACG
CCCGAACAAGGCCAAAAGCTGGCCAACGAGATCAAGGCCATCAAGTACCTGGAGTGCAGC
GCCCTCACCCGGCGGGGTCTCAAGAACGTTTTCGACAACGCCCTCACCGCTGTTGTCTGC
GCCAAGCAAGAGGATTCGAGTGCGGGGTCTTCCAAGCCCAAGAAGAAGTGCAGCCTGTTC
TGA
>g430-130807-133448
ATGAGTCGTCGCATCGAAGCCCTGGAGGAAGAAGCCACCACGACGGGTGCCGCTGGCGGA
AGCCCTCGGTCGACCCAGTCGCAGAAGAAGATCACTGTAAAGCTGGCGAGCAACCTCTTC
CTCAACCTCCCCGAGGGTCTTTCCGAGCTCTCCTACTCGAGGATCGGCAAGACTCGCATG
ATCGCGTGCTTCATGATCGACGGCAGTGACCGCTACACCGCCATGACCATCAATGCCATC
AACAGCTTCCTCAAGTCCACGCCTGAAATGCACGCCGGTGTGTTGGTGCCGCCCTCGTGG
GGCCTCAGCATTTTTGCCACGCGCGTGGTGCCCCAGCTGATCGCTCCCGAGCGAGTCATC
TTCAAGAAGTGGGGCCAGCACTTTGCCCAGTGGAACCCCACCCAGTACAAGCTCGACATT
CTCCAGTTCGCCGACGAGTACGACGTGGTCCTCTGGCTTGACTCGGACACCATTGTGTAT
GACGATCTGCGCCCGCTGTTGTTCGCCTTCTACAAGTCGCCGGCCAAGTACATGTTCGTG
CGCGACCATGTGTGCTTCCTCCCGGAGTTCCTCAACAACTACCCCTTCAAGGACAACAAG
GGCGGCGCCTTCGTGCCTCAGGCGTGCTTCATGGGATTCAAGGCCGAGCACATGAAGCGC
CTTTTCAACGTCTGGGAGGACACCTGGAGGCAGTGGATCGAGCCGGAGCCGTTCAAGAAC
TTCGCCGACCCGCTCCCGTCCTTCCCTGGCAGCAAGTTTTGCATTGAGCAGTACGCTCTC
GGACACGCCCTGGTTCTCGCCGAAATCGGCGAGGAGGACATCCTGGAGATCGCGCGCTCC
CACATCGTCCTCAACACACGCAACCCCAACCAAACGGGCCAGGCGTCCACCAACGGCGCC
CACCATGCCGGCTATGCTGGGCACGGTACGGGGAGCGGCGCTACCGGGTACGGCACGACG
GGCGGAGAGGCCCAGCGCCAGGCGGGTGCCAGCGTGCTGTCCGGCTCGACGGCCTACGCG
GGCCTGCCCTCGTCCCAGTTCCTGGCCACGTCGGCCTTCCTCGCCTTTGCCTCGGCCAGC
TCGTCCTACGGCTCGTCCTACCTCGGCTCCTACCCCTCGTCCTACCTCACCTCCTACCCC
TCGTCCTACTGGTCCTCCTACTCCTCCTATGGCGTCGCCACCGGCGGCACCATCACCTAC
TTCGGCTCGACCACCACCGACCTCCACCAGCGCAACGCCGCGGGCCAGGCGGGCGCCGCT
GGTTTCGCTCCGGGCACGGAGGGCGGCCAGGAGGGCAGGCATGCCTACCCCGACCTCCCC
GAGCACATCGCCATGGACAACCTGGGCGCGTGTGTGGTGCACTTCTACAACAGCTTCTAC
GACGCTGCCCACTACTGGTGGACCGAGAACGAGCAGTCCATCGTCAAGGCCCTCAAGCCC
CAATAA
>g433-73037-74438
ATGACCGCGAGCAAGTCGTTCATCGTCGTCGTGGCGATGCTGTGCCTGATGGCCACGGTC
GCCCTGGCCCACCGCCCGAGCCCCAGCCCCAGCCCGAGCCCCAAGCCCACCGTGTTCGTC
TTCACCGACGTCAACACGGACGACTTCATGGCGCTCAAGCTCATCCTCAAGTACTCCAAC
GTCAAAGTGGGCGGGTTTGTCGCCGGCTGCGCGGGCTTCTGCAACATGGGTCCGGGCATC
CAGAACCTGTTCGGCCTGCTCAAGTTCATGGACCGCGACGACGTGCCCGTGTGGGCCGGC
GAGGCCTACGCCTCGACCGAGATCGATTCGGGCAACTACTCGTGCACCTTCCAGAAGACC
GTGCCCCTCTTCCCCAAGGGCAAGACCTGGGCCGACACCATCCTGGGCCTCAACCAGCGC
TATCCGCGCCTGACCGACCCCCAGCGCAACTACTACCCGGGCTTCCCCCAGGTCTACGAG
CCCCTCCGCGCCGCCATCGCCGCCCTCGACGGCCCCGTCGAGTTCCTCTCCCTGGGCACC
CTCACCGAGATCGACTACCTCTTCCGCCGCTACCCCGACCTCAAGCAGCGCGTGCGCCGC
ATCACCATCATGGGCGGCGCCGTCCACGTCCCCGGCAACCTCTTCTTCCCCCGCGGCAGC
GCACCCAACACTGTGGCCGAGTGCAACATCTACCTCGACCCCCACTCGGCCCGCAACGTG
TTCGTGTCGGGCGTGCCCATCCGGCTCGTGCCGCTCGACGCCACCAACGCCTTCCAGCTG
TCGTGGGAGTTCCTGAACGAGTTCGACGACGTGGCGCGCACCAAGGAGGCCAAGTTCGTG
AAGGACCTGCTCATCCTGATCAAGAACAACTCGGCGGCGACGTCGATGTACTCGCTGTGG
GACCCGCTGACGGCCGCCATCCTGGCCGACCACTCGATCATCTTCGAGGAGGAGACGGTC
AACATGACGGTGGTCACCTCGGGCTCGCAGGCCGGCCGCACCGTGATCGACAACGTCGAG
GGCAAGCCCGTCCAGGTGGTCCTCACCGCCGACCCCGACTTCTACGACATCTTCATCGAC
AAGCTCAACAGGCGACGCCGCTGGTAG
>g433-222853-223830
ATGACTCAGCCCCTCATCAAGAAGCGCATTGTGAAGAAGCGCACCAAGAAGATCGCCCGC
TTCCAGTCCGATCTTTTCCTCCGCGTCAAGACCTCATGGAGGAAGCCCCGCGGTATTGAC
AACAGGGTGAGGAGGAAGTTCAAGGGTTCCCTCATCATGCCCAAGATCGGTTACGGATCG
AACAAGAAGACCAGGCACATGCTGCCCAACGGCTTCCACCGTTTCCGCGTCTACAACGTG
AACGACCTCGAGCTCCTCCTGATGCACAACCGCAAGTACGCCGCCGAGATCGCCCACAAC
GTCTCCTCCAAGAAGAGGAGGGAGATCATCGAGAGGGCCGCCCAGCTGAACGTCAAGGTG
ACCAACGCCAGCGCCAGGCTCAGGACCGAGGAGAGCTCGTAA
>g433-263946-264749
ATGGCGACCTCTGTACCGATTCTGCGTAGGGAAATCAACCCGCTGCGTGAGGGCGCCACT
GCCGTGACGGCCCAATCCGATGTTCCCATCGTGCATCCCCTTCAGGCTCGTGAGCTCAAC
TTCGCCAAGAACGAAGTGCAGTTCAAGAGAGTGGCCTTGCAGAACGTGTACGGTTCGCAT
CTGTCCATGAGGCTCCAGATGGAGGAGAGCATTCTGTCCAATTTCCAGCGACTCCCCACC
CTCAAGAGCGAATTTGTGGGTTTGTCCACCAGCCTGGGACTTGACGAAGAGATTGGCTTC
AGCGACTTCCTCAGGGATCCTTCGGCTCCCGAGTCTGCCATCGATCTGCACGAGGTGATG
GAGCACCGTCTTGGTTTGTAA
>g446-295237-295717
ATGTCGCGCGTGTTCTTCATCCAACTGGTGGCCGTCATCGTGGCCCTCCTCTTCGCCTTC
GCCGTCTTCGCCGACGCTCAGGCCACCCCCACGCCCTCGGCTTCCGCCTCGGCCCTGCCT
TCCCCCGTGGCTTCCGCCTCGCCCTCGCGCTCGCCCAAGGCCTCCGTGGCGCCCTCGTCG
GCCTCGTCGCTCAACGGCTTCCTCCTCGAGGCCCTCTCCTTCCTCTTCTAA
>g448-52807-54488
ATGGAAGCTGCCAGGAAGTATTTGGAGGATTCTGTGGCCGCGCTGACGGGCCAGAGCCCT
GAATTGGCTGTCAAGTATCAGGAGCTGCTCGATCTCTTTGAGAGGAGGCTCTGGCACAAC
CTCACCACCCAGTTGCTGGAGCTGGTGAACCACCCTCACCTCAAGGAATCGCCACAGCTG
ATCCCCCTCTACACCGATTTCATCAAGCACTTTGAATCCAAGATCAATCAGCTCAGCTTC
GTTCACATCATTCTCGCCATCTCCAAGGCCTACCGCGACCCGGTGGAGACTATCAAGTTC
TTGGAGGGCGTGGCGACGAAGGTGTCGGGAGCCGAGGGCAGCCCTGATGCCTACCTGCTG
GTGCGTTCGGTGATCGCCCAGCTCAAGCTGCAGGACCCCAAGAACCGAGAGGAGACCAAG
GAGCTGCTCGAGTCCATCCAGACTGGCCTCGACGGCGTGGCCGGCATCGACACCTCGGTC
TATGCCAACTACTACAAGGCTCAGGCCATCTATCACAAGGCTGCCGGTCTCTCTGCCGAG
TTCTACCGAAACGCTCTCCTCTACCTCTCTTACACCGCGATCGAGTCCCTCTCGGAGCAG
GAAAGGCTCCAGATGGCCTTCGATCTCGGCATCTCTGCCCTGGTTGGCGAGGACATCTAC
AGCTTTGGCGACCTTTTGGCGCACCCTATCGTGGATTCGCTTCTGGGCACGCAAGGCGAG
TGGCTGCACCACCTGCTTCACGCCTTTAACAAGGGTGATATCCACACGTACGAGCAGCTC
GTGGCGAAGTACGAGCAGCAGCTGGCCGGCCAACCCATCCTCGTCCAGCACGTCGACCGA
ATGAAGGAGAAGATCTCCATTCTCTGCCTCATCGAGCTCATCTTCGCGCGCCAGGCCATC
GACAGGAGTGTCCCTCTCTCTGCCATCGCTGAGACCACCAAGGTCGGCCTTGACATGGTC
GAGCTGCTGGTGATGAAGGCGCTCTCGCTCAAGCTGTTGAAGGGCAAGATCGACCAGCTC
AACCAGACCTTCAACGTGACGTGGGTGCAGTCGCGCGTGCTCAGCCTGGACCACATCAAG
AAGATGAGGGACAACCTCCAGGACTGGACGAAGAAGGTCAACCAGACCTCCCTCTACATC
GAACAGGGCACCCCCGAGCCTGTCATCTAA
>g448-84020-85758
ATGGTGACAATCCAATCGGTCAATCAATCCGCGCTCGTGTTTCGGGAAGGACAACAAGAA
ACAACACACATGGAATACGATTATCTCTTCAAGATCCTCCTCATCGGCGACTCGGGCGTT
GGCAAGTCTGCTCTTCTGCTGCGTTTTGCTGATGATGAGTACAGCGAGTCGTACATCTCC
ACCATTGGCGTGGACTTCAAGATCAGGACTATCAACATTGATGATAAGAGCGTGAAGCTT
CAGATTTGGGACACGGCTGGCCAGGAGAAATTCAGGACGATCACCTCTTCCTATTATCGT
GGCGCCCACGGCATTATCATCGCCTACGACATCACCGATGAGGCCTCGTTCTCTCACGTG
CGAGGTTGGTTCCAGGAGATCGAGCGCTATGCGCAAGATAACGTGCGCAAGCTTTTGGTC
GGAACGAAGTGCGATCTTGAGAGCAAGCGTGTGGTGGACAAGGCCAGAGGCCAGCAGCTT
GCGGATGAGCTGAACGTTCCGTTCGTTGAGACGTCATCCAAGAACTCCACAAACGTCGAG
CAGGCCTTCTTGCTCATGGCCAAGGAGATCAAGAACAAGCAGGGTCCTCCTCCTGCTGCC
TCCACCCAGAAGAAGGTCGATCTCAGCGGACCCACGACCCCTGTGGGCGGCGACAACGGC
GGCTGCTGCTAA
>g448-218756-220330
ATGGAATCGACGCAGAAGAAGTACAAGATCCTCGTGTCGAACGACGACGGCATTGATGCG
CCTGGTGTGCTCTCCATCGTGGAAGAGTTGGCCCGCTACCACGACCGCTACGAGGTGCGC
GTAGCCTGCCCGGCCGAGCAGCAATCCGCCCAGTCCCACGCCGTCACCATCTTCAAGCCC
CTCTGGGCCGAGCCCTATGCCTTCCACTCCGACCTCGCCCATGTGCCCGCCTACAAGGTG
TCCGGCACGCCCACAGACTGCGTCAAGGTGGCCCTCATGTCCGACCTGCTGGGCGGCTGG
CAGCCCGACCTCGTCGTCTCTGGGATCAACGCGGGACAGAACGACGGATTGAATGTGATC
TACTCGGGCACCTGCGCTGCGGCGTTGGAGGCGAGCATGTACGACATTCCGTCCATCGCC
CTCTCGCTCGAATACAACTTTGCCATTGGAGGTGTGTGGCGCTACGCGCAGTGTGCGTCC
CTGGCGATTCCCATTATCGACGCCGTATTGGCCGACATCTCGCTGTGGAAGAACATCTGC
TGCAACGTCAACTTTCCCAACGTGCCCGAGGACCAAGTCAAGGGCTACAAGCTCACAAAG
CAGGGCAACAGCAGCTTCAAGGACAAGTACGTGCGGCACCACATGACCGAGGAGGAAAAG
CACCTTCACCCCACCCGCACCGTCTACCGCATGGAGGGCTTCATGGAGCTGTCGGACACG
GACGAGGAGCTGGACACGGTGGCGATGCGACAGGGCTGGGTGGCGGTGACGCCCATCTCG
GCCTACGCTCAGAACGCGCTCGCCCTCGACACCACCGCCAAGATCACCAGCTGGCCCGTC
TTTTCCCTGACTCCCGCATCCCTCTGA
>g448-240796-241600
ATGCAGATCTTCGTGAAGACCCTCACCGGCAAGACCATCACCCTCGAGGTCGAGTCGAGC
GACACGATCGAGAACGTGAAGCAGAAGATTCAGGACAAGGAGGGCATCCCTCCTGATCAG
CAGCGTCTCATCTTCGCCGGCAAGCAGCTCGAGGACGGCCGTACTCTTGCTGACTACAAC
ATCCAGAAGGAGTCCACCCTCCACCTCGTTCTTCGTCTGCGCGGCGGTATGCAGATCTTC
GTCAAGACGCTGACCGGCAAGACTATCACCCTCGAGGTCGAGTCCAGCGACACGATCGAG
AACGTCAAGCAAAAGATTCAGGACAAGGAGGGTATTCCTCCCGACCAGCAGCGCCTTATC
TTCGCCGGCAAGCAGCTCGAGGACGGCCGCACGCTCGCCGACTACAACATCCAGAAGGAG
TCCACCCTCCACCTCGTCCTTCGTCTTCGTGGTGGTATGCAGATCTTCGTCAAGACCCTC
ACGGGTAAGACGATCACCCTCGAGGTCGAGTCGTCCGACACGATCGAGAACGTGAAGCAG
AAGATCCAGGACAAGGAGGGTATCCCTCCTGACCAGCAGCGTCTCATCTTCGCTGGCAAG
CAGCTCGAGGACGGCCGTACTCTGGCCGACTACAACATCCAGAAGGAGTCCACCCTCCAC
CTCGTCCTCCGCCTCCGCGGTGGCAACTAG
>g448-345884-347168
ATGGCGAGTAGCGTGGACCAAGTGTTTGAATTAATCACCAAGAACGTAGCAGCCGATCCG
GGCCTTGTCAAAAGGGTGGGCGGTGTGTACCACTTTGACATCGATGGCAAGGTGTGGACC
GTAGACCTGAAGAATGGCAACGGCAGCGTCAAGTCGGGCAAGGACGGCAAGGCTGATTGC
ACCATTACCATCAAGGGCGACGACTTCCTCAGCCTGGCTTCTGGCAAGCTCAATGGCCAA
ACCGCGTTCATGCAGGGCAAGCTTAAGCTTGGCGGCAACATGGGCTTGGCCATGAAACTT
GGGCAACTCTTCGAAAACAGGAGTGGCGCTGCGCCTCAGCAGCAAGCCAGCAGCGGTGCC
ATCGAGGTCGCGTTCAACGAGATTCAGAAGAACATCTCTGCCGATCTGAGCCTTGTGAAC
AAAATCAATGGTGTCTATCAGTTTGACATCACCCTTGCGTCAGGCGACGTTCAAAAGTGG
GCTGTTGACCTCAAGACTGCTCCTGGCAGCGTGTCCAAGGGTGCGCCGCCCAAGGCTGAT
TGTACCCTCGCCCTCAAGGAGGAAGACTTTGTGAACATGCTCACTGGCAAAGCGGATGGC
CAGAGCCTCTTCATGCAGGGCAAGCTCAAGATGACTGGCAACATGGGACTTGCCATGAAG
CTTGGTCAAGTGGTGGCCAACAAGCAGCCTAAGCAAGCCAAGCTCTAA
>g448-347266-348415
ATGGCGTCACGGGGCAAGGACGAAACGGCCAAGTTGCGCACCAACATTCAAGACCAGCTC
AATCGACTTATCACGCAGCTGCAGGACTTGGAGGAGCTGCGTGAAGAGATGGAGCCCGAT
GAGTATCGCGAGACCAAGGAAGAGACTCTCCAGCAGATGCGCGAGTTTCAGGCCTTCTTG
CGCAAGACTGCCGCTGGTGACCTCTCCCTCGTCAATGATCTCGGTTCCATACAGCTGGCC
ATCCAAGCAGCTGTGAGCGAGGCCTTTCATACGCCCGAGGTCATTGGCCTTTTCGCCAAG
AAGCAGCCCGGTCAGCTCCGTGGCCGGCTAGATGCACTCAAGAGGGACCTGAAACTGGGC
AAGTTGTCATCAGACGCCTACACGCAGCAAGCTCTCGAGATCCTGACCGCCCTGAAGAAG
CTTGGGGAAACGCTTTCAGCTGAGGAGCTCCATTTCTTGTCCGCCAACATGACAGCATCC
CTGTCGGCCTTTGAGCGGGTGGAAGCGGACATTGGGAAAGGCACACAAGAAGCTGTGCTC
TCGGTGGCAGGCAACCAAATCAAAGCGTCCCGCGGTAACTGA
>g448-348417-350528
ATGCAAAGCAAGGATCACTCCATTCCCAAGATCGGGTCCATGGCGGAGGACAAGGTGGCG
GCCGAAGTGGCCGAGTACGTGCGCTTCCACAACGACGACAAGGAGGACCGCACGGCCGAG
CGCAAGGGCAAGTACGCGGCCATGGTCAACGCCTACTACAACCTGGCCACCGACTTTTAT
GAGTGGGGCTGGGGTCAGTCCTTCCACTTTGCCGTGCAGGCCTACACCGAGTCCTTCCAG
ACCTCCATCGTGCGCCACGAGCACTACCTCGCCCTCCGCCTCCAGTTGGAGAAGGGATGG
CGGGTGCTTGACGTGGGTTGCGGAGTGGGTGGCCCTGCCCGCAACATCGCCCACTTCGCC
AAGTGCAACGTGACGGGCCTCAACAACAACCAATACCAGGTGGACCGCGCCACGGCGCTC
TCGACAAGGCAGGGCCTGAGGGACTCCACCAACTTCGTCAAGGGCGACTTCATGAAGCAG
CCCTTCGAGGACAACACCTTCGATGCGGTCTACCAGATCGAGGCGACGGCTCATGCCCCC
GACAAGGTGGGCTGCTACAAAGAAATCTTCCGCGTGCTGAAGCCGGGCCAGCTGTTCGGC
GGATACGAGTGGTGCCTCACCGACAAGTACGACCCCTCCAATCCTCAGCACCGTCAGATC
AAAAAGGGAATTGAGGAGGGAGATGGCCTTCCCGATATCGCCACCTGCAACGAGGTGGTG
GAGGCGCTTAAGGAGGCCGGCTTCGAAATCGTTGAGGCTTTCGACGCAGCACACTTGCAC
CGCCCCGATTTCGATTACCCGTGGTTCTACTACCTCACCCCGGGCTACAATCGTCCCTCT
CGCTTCCAGTTCACGCCCGCGGGCAAGTACCTTACCAAGAAGGTCCTTGGTCTCGCAGAG
AAGCTGCACATCGTGCCCGCCGGCACCAACGGCGTTTCGGGTTTCCTGATGACCGCCGCG
GAGGCCCTGTCGGCAGGAGGAGAGCAGGACCTGTTCACTCCTATGTTCTTCCACCTTGCG
CGCAAGCCCTTGGACAACTGA
>g448-396616-397931
ATGAACACGAAGAGGATTATCGACGAGGAAGCCCTCCAGCTGGCCAAGAAGAAGCCGCGA
ACGCCCGAGCCGCTGCTCGAGGAGAACCCCAACCGATTCGTGCTGTTCCCCATCCGCTAC
CAGCGCATCTGGGAGATGTACAAGAAGGCCGAGGCCTCCTTCTGGACGGCCGAGGAAGTC
GATCTGAGCTCCGACATGAAGCACTGGGAATCGCTCAGCAACGACGAGAGGCACTTCATC
AAGCACGTCCTGGCCTTCTTCGCCGCCAGCGACGGCATCGTGGCCGAGAATCTCGCCGGC
CGCTTCATGAAGGAGGTGCAGGTGCCCGAGGCGCGCTGCTTCTACGGCTTCCAAATCGCC
ATCGAGAACATCCACTCCGAGATGTACTCGCTCCTCATCGACACCTACGTCAAGGACCCC
ATCGAGAAGGACCACCTCTTCCGCGCCATCGAGACCGTGCCCTCGGTCAAGAAGAAGGCC
GATTGGGCCATGAAGTGGATCAACAAGGAGGGTGTCGAGACCTTCGCCGAGCGCCTCATT
GGATTCGCCGCCGTCGAGGGCATCTTCTTCTCCGGCAGCTTCTGCTCCATCTTCTGGCTC
AAGAAGCGCGGCCTCATGCCCGGCCTCGCCTTCAGTAACGAGATGATCAGCCGCGATGAG
GGCCTCCACTGCGACTTCGCCTGCCTCCTCTACACCATGCTCGTAAACAAGCCCTCGACC
GACGTGCCCAAGCAGATCATCCTCGAGGCCGTCGAGATTGAGAAGGAATTCGTGACCGAG
TCGCTGCCCGTGGCCCTCATCGGCATGAACGCCGACCTCATGTGCCAGTACATCGAGTTC
GTGGCCGACCGCCTCCTCGCCGCCCTCGGCTGCGAGAAGCACTACAACCAGACCAACCCG
TTCCCGTGGATGGAGCTTATCTCCCTCGAGGGCAAGACCAACTTCTTCGAACGCCGCGTC
GGCGAGTATGCCAAGGCCGGCGTCTCGTCCAAGCAGGAGAACAGCCGCGTCTTCACCACC
TCGGAGGATTTCTAA
>g448-406766-408323
ATGTCCAAGAGTCCCTTCCAAGGTTTGGTGAAGGCCAATCTGCCCGGCATGGGCGGCAAG
GGCCTGAAGACTGCGATGTCACTCGCGGTTGTTGGTGTGGGCGCCTCAGCAGTCGCCTAC
AACTCCCTCTTCACAGTGGAGGGTGGTCAGAAGGCCATCATCTTCAGCCGCTTTAGCGGT
GTTCTGCCCAAGGTGTACAATGAAGGGCTGCACTTCAGGATGCCCTGGCTTCACATCCCG
CACGTCTTCAACGTGCGTACGCGACCCACGAGCATCCCCTCCCTGACTGGAAGCAAAGAT
TTGCAGATGGTGAACATCACGCTGCGCGTGCTGACGAAGCCCAAGTGGGAGAAGCTGCCT
GAGATTTACAAGAAGCTCGGCACTGATTACGATCAGCGAGTTCTGCCTTCCATCGTGAAT
GAAGTCCTCAAGGGCGTCGTGGCCCGCTTCAACGCTGCGCAGCTTATCACACAAAGAGAG
CTGGTGAGCGGCATGATTCAGGACCGTCTTAGGGAGCGTGCTGCCGACTTCTTCATCGAT
CTCGATGATGTGTCGATCACTCACCTCAGCTTCGGCCGCGAGTATACCGCTGCCATTGAG
GCCAAGCAAGTCGCCCAGCAGGAGGCGGAGCGCGCCAAGTTCATTGTGGAGAAGGCTCTG
CAGGACAAGCGCAGTATCGTCATCAGGGCTGAGGGTGATGCCGAGTCGGCGCGGATGATC
AGTGAGGCCGTTCAGTCGAATCCCTACTACCTCGAGCTCAAGACCATCGAAGCCGCGCGA
GACATTGCTGGCTCCCTAGCCAACTCGCAGAACAAGGTGTACTTGAGCAGCGACATGCTC
ATGTTCAACCTTCTCAGCTCCATCAGCGGATCCAACACCGCTTCCAGCCTCGTGGGTTCC
GTGCTGTCGGCCAAGGATGCCACCATCGCCAGGCAGTAG
>g448-516809-518949
ATGGACGGCAAGCCGAACGTGTTGGTTCTGGGTGCTACTGGCTTCATCGGCCGCAACCTC
GTCAAGTACCTGGTGGAGAATGACCTCGCTGCTGTGACCTCTGTTGATAAGGTCCTCCCC
AGCCTCTCCTACCTGAGCCCCCAGGAGGAGGCTCTCTTCGAGAAGGCCAACTTCATGCAG
AAGAACCTCGTCAGCCCTGTCTCGATTGCCTCCATCTGGAGCGACACCGGCATCAAGTTT
GATTACGTCATCAACTGCGCCGCCGAGACCAAGTACGGCCACAACGACGAGGTCTACAAG
GAGCGCGTGCACCACCTGTCTGTCTCCTGCGCCCAGGAGGCCGCCAAGCAGGGCGTCAAG
AGGTTCATCGAGGTCTCGACTGCCCAGGTCTACGAGCCCAGCAACAAGGCCAAGGACGAG
AGCGGCAAGGTCGACCCCTGGACCAACCTCGCCAAGTGGAAGCTGCAGTGCGAGGAGGAC
CTCAAGAAGATCCCCGGCCTCAGCGTTGTCGTTGTGCGCCCCGCCATCGTGTACGGCCCT
GGCGACAAGACCGGCATTGCTCCTCGCATCCTCTGCGGCGCCATCTACAAGCACCTCGAC
AAGAAGATGAAGTTCCTCTGGAGCGGTGACATGAAGATTAACACCGTGCACGTCCACGAT
GTTGTCAAGGCCCTCTTCCACCTCTGCACTCACGGCGACGATGGTGCCACCTACAACCTT
GCCGACAAGAGCGGCACTGATCAGGAGCTTGTGAACAAATGCCTCGCCAGCATGTTCGGC
ATCAAGACTGATTTCTTCGGCTCGATCAAGTCGAACATGGCCAAGGCCCTCGGCATGGAC
GCCGTCACGTCGCAGGTCAACGACAAGCACGTCGCCCCGTGGAGCAAGATGACCAAGGAG
AAGGGTATCGAGGTCACTCCCCTCTCGCCCTACCTCGACAAGGAGCTCCTCGGCAACAAC
GCCCTGAGCGTCGACGGCTCGGCCATCGAGAAGACCGGCTTCACCTACGACGTCCCCGAG
TTCAGCGAAGCCAAGCTGAGGGAGTGGGTCGACTACTACACCCAGCTCAAGCTCTTCCCC
GAGGGCTACGTCCAGTAA
>g452-21447-22148
ATGCGTCGCATCTCTGCCTCCTCGCGAGTTCTTGGTATGTCGGCCGCCGCTGGCGGGCGT
TTCGCCCCCCGCACCGCCTCGGTGCTGACCACTACTACTCGCCGCTCCTACGCGACCGAT
GACGCGCAAAGGGAGGCGCACGAGAAGGCCGTGAAGGAGGGCAAGCTCATCTTCACGCTT
GCGTGCCCCCACGAGACCCTCATTAATGCTGAGCCCGTCAGGATCGCCACGGTGCCCTCG
GCCACCGGCGACATGGGTATTCTCGCCCACCATGTGCCCACCATCGCCCAGCTCAAGCCC
GGCGTGGTGAAGGTGACCAAGTTCGACGAGTCGGGCGCCACCAGGGACGCCGACTACTTC
ATCAGCGGCGGCTTCGTGACCGTCTACCCCGATTCTCACTGCAACGTCAACGTCGTCGAG
GCCTTCCCGCTCGACCAGCTCGACCCCGAGAAGGCCAAGAAGGGTCTCGAAGATTTTACC
AAGGAGCTTGCTTCGGCCAGCGACGCTGAGGCCAAGACCGTGGCCACGATCGGCGTTGAG
GTGTACAAGGCCATGTGCTCTGCGCTCAAGGTGTAA
>g452-22276-25291
ATGGGGGCTTGGGGCCTCGTCTTCCTTTGCGCTTCTCTGGGCGTCAGTCCGGGTTTTGCT
AGCAGCTGTGCGCCACCACCACACACTCCCGCAGCAGGTATAAAGATGTTGAGAAACTTC
AGCAAGAGGGCTCTCCCCCGCACTGCCCTTTCTCGGGGTCTCGCTACCCGCACCGCTGCC
GGGCTCAGGCAGGGCGCCAACACGACCCTCGCTACCTCGTCGGCTCCTGCCAAGTATGCC
ATGTGGGGCAGCAGGACTCTGGCCACCCAGCCCGAGCCCAAGACCGCCAGCAAGGCCGAG
GCCGAGTCCCAGGCCGTGGATACCGACGGCAAGATCGTGCAGATCATCGGTGCCGTCGTC
GACGTCAAGTTCCCCGAGGATGCCCTCCCGCCCATTCTCAGCGCTCTCGAGATTCAGGAC
CACAACATCAAGCTCATCCTTGAGGTTGCCCAGCACCTTGGTCGCGGTGTGTGCCGTACC
ATTGCCATGGATGCCACTGAGGGTCTCCAGCGTGGCCAGAGGGTCAAGGACCTGAAGGGT
CCCATCACCGTGCCCGTCGGCGCCGCCACTCTTGGCCGCATCATCAACGTGATCGGTGAC
CCCATCGACGAGAAGGGTCCCATCTACTCGAACAAGAGGAACCCCATCCACGCTGCCCCT
CCCACGCTCACTGAGCAGGGTGCCTCCGATCAGATCCTCGTGACCGGTATCAAGGTCGTC
GATCTGCTCGCCCCCTACGCCAAGGGTGGTAAGATCGGTCTTTTCGGCGGTGCTGGTGTC
GGCAAGACTGTGCTTATCATGGAGCTCATTAACAACATCGCCAAGGCCCACGGTGGTTTC
TCCGTGTTCGCTGGTGTCGGTGAGCGTACTCGTGAGGGTAACGATCTGTACCACGAGATG
ATCACGTCCGAGGTGATCAAGCTGAAGGACCCCAAGACCCTGAAGGAGGGTGACCACATC
GGCGAGGGCTCCAAGGCCGCGCTCGTGTACGGTCAGATGAACGAGCCCCCGGGTGCCCGC
GCTCGTGTGGCCCTGACTGGTCTGGCCGTCGCCGAATACTTCCGTGACGAGGAGGGCCAG
GACGTGCTTCTCTTCATCGACAACATTTTCCGCTTCACCCAGGCCGGTTCCGAGGTGTCT
GCCCTTCTGGGTCGTATCCCGTCCGCTGTCGGCTACCAGCCCACCCTGGCCACCGACATG
GGTCAGCTCCAGGAGCGTATCACCACCACCATGAAGGGCTCCATCACCTCCGTGCAGGCC
ATCTACGTGCCCGCTGACGATCTTACGGATCCCGCCCCCGCCACCACCTTCGCCCACTTG
GACGCCACCACTGTGCTCTCCCGTCAGATTGCTGAGCTGGGTATCTACCCCGCCGTGGAC
CCTCTCGATTCGACCTCGCGAATGATGGATGCCGAGATCGTCGGTAAGGAGCACTACGAC
GTCGCCCGTGCCGTGCAGAAGACCCTCCAGGACTACAAGTCGCTGCAGGACATCATTGCC
ATTCTCGGAATGGACGAGCTGTCGGAGGAGGACAAGGCCACCGTCTACCGTGCCCGTAAG
ATCCAGAAGTTCCTGTCGCAGCCCTTCCAGGTCGCCCAGGTCTTCACCGGTTTCGAGGGC
AAGTTCGTCTCGCTCAAGGACACCATCTCGGGCTTCAAGTCGATCCTCGAGGGCAAGTAC
GATCACCTGCCGGAGCCCGCTTTCTACATGGTGGGAGACATCGAGGAGGTGGTCCAGAAG
GCCCAGAAGCTCGCCGCCGATCTCGGTGGTGGCTCCGCCGCCAAGAAGGACGGCAAGGAC
GCCAGCGGCGCCAAGGTGTCGGGCAAGCGTGAGCTGACCCTCGACGCCTTCAACGACCCC
TCGTACCCCGAGCTCAAGGAAGGCGACTTCGAGCTCAACGAGAAGTCCTTCAGGGTGTTC
GCCGAGTACACCAAGAACGTGGCCATCCCCTACGCCCGCGACCACGAGCTCAAGGAGTTC
CCCGAGGATGCCGCCAAGATCAAGGCCAAGTACGACAACATCCTCTCCAAGATCGACGGC
GAGACCGACGAGCTGGTCAAGGAGCTCCACGATGCCTCGCAGAAGCACGACGCCGAGAGG
AAGCGGAAGGAGCAGGAGGCTGCCCTCAAGGCCCAGAAGGCCGCCGCCCCCCAGGCCGCT
GCCCACTAA
>g452-37936-39120
ATGCTGAAGCTCAGGAAGATGCAGCAGGAGAAGGAGGCGGCGGCAGCGGCTGCTGCTGCC
GCCAAGTCAGAAGCGGCGGCGCCTGCCGAAGGGACGACAACGACGGGGGAGACCAGCACC
AGCGGATCAGCGCCCGGGGGCTACGTGCTGAAGAAGACTAATTCCAAGGAGCTCCTTAGC
GTGAGGAAGTCCAAGTCCAAGGAAAATGTCATGTCTCTTCGCACCAACACCAACCGCAAG
AAGGAGAAGACGAAGGCCGTTGAGCTCCGAGTCCACAAGGATGTGGCCGAGATCGACAAC
ATTCCCGGTTGTGAAGTGGACTTCCCTGATCCCAACAACCTGATGGTGTTCTTCGTTAAC
GTGACGCCGAGCGATGGTCTCTACACGGGCGCGACATTCAAGTTCCAAGTCACCATCCCT
CCTTCCTACCCGTACGACCCTCCCAAAGTCGAGTGCCAGACCCTCGTCTACCACCCCAAC
ATCGATTGGGAGGGTCACGTCTGCCTCAACATCCTCCGCGCCGACTGGATGCCTGTCCTC
AACCTCGGCTCGGTCCTCTTCGGCCTCGTTACCCTCTTCCTCGAGCCCAACCCGGACGAT
CCTCTCAACAAGGAGGCGGCGCAGCTCATGATTGACAGACCGAGCGAGTTCGAGAGCAAC
GTCAAGCGCACTCTCCAGGGCGGTTACTTCATGAGCAGGCAGTTCCCCAAGCTCAAGTAA
>g452-43337-44138
ATGTCGGACACAGAGGATGCGGGAAATGAGATGGTGGAGGAGACGAAGGACACCAAGAAG
AAGGTAGTCGGTAAGGGAGCCGCCTCCAAGGGCGCTGGTGGCAAGAAGTTCGAAGTTAAG
AAATGGAACGCTGTCGCTCTGTGGGCCTGGGATATCGTCGTCGATAACTGCGCCATTTGC
CGAAACCACATTATGGATCTTTGCATCGAGTGCCAAGCCAACCAAGCCTCGGCCACGAGC
GAAGAGTGCACAGTCGCTTGGGGTGTTTGCAACCACGCTTTCCACTTCCACTGCATCAGT
CGCTGGCTGAAGACGAGACAAGTGTGCCCCCTCGACAACCGCGACTGGGAGTTCCAGAAA
TATGGTCGCTAA
>g452-76796-77704
ATGGAAACCCCTGAGAAGCCCGAACGCAGTGACGCCGAGTTCCAGCAGTCGCTGGATGCC
AGGCAGAGGGAGGTGAACAACGCGCTCAACACGGGTAACCCTGGTCGCGCTCTCAGTGCC
GCCCTCCAGGACCCTCCTCTTGGGTGCAAGAACCAGCAGATCAGGGATGCCAGCACGCAG
GCTGTGGTGAACGTTCTCACTTCGGTCAAGGATGCTGACATGCAGAAGAACATCGATGCT
CTCTCCCCCACCGATCTGGACACCCTCATGAAGTACATCTACCGCGGTCTTGAGGACGGT
GAGAACTCGACCTCGCTCCTGAAATGGCACGAGGCTGTCACCAAGAAGGGTGGCCTCGGC
TGCATCGTCCGCGCCCTGGCTGAGAGGAAGGCCTTGTAA
>g452-170596-172195
ATGAAGAGCAGCTTGATTGTAGCTGTACTCCTTGGCCTTGTGGTCGTTATGGCGTCGGCT
CACGGCGGCCACCACGGCAAGGGCGCTCATGGTCACCACGGCGGCGCTGCCAAGGAGAGC
TCGCACAGGCTCACCTGCAGCTACAAGAACACCAAGGGTCACCTCTTTGACCTCAGCCGC
CTTTCGTCGAAGGATCTCCTCGTGAAGGACCTGGTGGGCGGTTCTACCTTCCACTTCAAC
CCTTGTGGTTCCGTGCACGCGAGCCAGTGCCCCTCGGGTGCGGCCGTTTGCGAAATCACC
GGCAAGGGCGAGGCCATCTCGCACGGTCTTGCCTGGGACGCCCACTGGGCCGACGGAGTT
GAGTCTGGTGCTTCGCTTGAGGTCATGTACGGCAGCGGAGAGATGTGCTCCGATGGCGTG
CACCGCAAGACGCTCGTCGAGATGCGCTGCCTGCACTCCTCTGACCCCAACACCAGCCCC
AGCTTCCTGATGGGCGTCATCAAGGACGAGTGCGTACTCAAGCTCATCGTCGAGTCGCCT
CTCGCCTGCGACGTGGAGGCTATCTGCTCCGCCGTTGTGAACGAGGCCACCTGTAACGCC
AACAGCGACCTGTGCGACTGGAAGGCCGGCCACTGCGTGCCGACCGATGGCCACCACAGC
ATGTGGAGCTCGCGCCAGCGCGTGGTCACCTTCGCCATCTCGGCCCTCATCGGCTTCACC
GCCGTCGTCTTCCTTTGCTGCTCCGTCACCTGCGCCTGCATCGCCTGCCGCCGCGCCGCT
CAGCGCAGGAAGCTGCGCCGCAGCCTGCCCACCTACAAGAAGGTTAAGAAGGTCAGCGAC
AAGAAGAACAAGAAGGTCGCCGCCGAGGAGCTCAGCCTCATCACCCCCGAGACCCCCTAC
GAGAGTGCCCCCGTTCCCGAGGCCCAGGTCTACCCCTTCCAGCAGTACGTCATGCACCAG
GCCGCCGACGGCTCCTTCGTGCCCTTCCCCGTGCACATGCCCATGCCCGTGCAGCCCCAC
GCCGTCCCGCCCCACGCCTACTCCTACCCCATGGTGCAGATGATGCCCGCCTTCCACCTG
CAGCAGCACCAGCACCAGCCTCAGCAGTAA
>g452-218277-219358
ATGGCGCAACAGAACTACCTTCGTCTGGGCGATACTGCCCCTGATTTCGAGGCTGATTCC
ACTCAGGGTCGCATCAGCTTCCACAAGTGGAAGGAGGGCAAGTGGGCCATCCTCTTCTCG
CACCCTGGTGATTACACGCCCGTGTGCACCACCGAGCTGGGCATGACCGCCAAGCTGCAG
CCCGAGTTTGCCAAGAGGAACACGCTCGTCATCGGCCTCAGCGTGGACAACGTCGATGAC
CACCACGGGTGGGTCAAGGACATTGAGACGACGCAGAACTGTACGGTGAACTACCCCATC
GTGGCCGACCCGGACAGGACGGTGGCCGAGACCTACGGCATGATCCACCCCAACTCGCCC
CACACCATGGCCGGCAAGCTCACCGTCCGCACCGTCTGGATCATCGACCCCAACAACAAG
GTCCGCCTCAACCTCACCTACCCCGCTGCCACCGGCCGTAACTTCAACGAGGTGATGCGC
GTGCTCGATGCGCTGCAGCTGACGGACAACTACAAGGTGGCCACCCCCGTCAACTGGGAG
CAGGGCCAGGACTGCGTGGTGCTTCCCACCATCTCCACCGAGGACGCCAAGAGCCTCTTC
CCCAAGGGCGTCACCGAGGTCACCCCCTACCTCAGGCTCACCCCGCAGCCCAACCTTCCC
TGA
>g452-244227-247258
ATGGAAGGAGCAGCAGTGGTCGCGCGCACGTTGAAGGCACTCGAGGTCACCGATGTGTTT
GGTGTTGTTGGGCGGCCGGTGACCAACATCGCCATGATGGCACAAGCCGAGGGCATTCAC
TTCTGGTCCTTCCGCAATGAGCAAGCCGCCAGCTATGCTGCGGGCGCTGCGGGCTACATG
ACGGGACGTCCCGCTGTGTGTTTGGCCGTGTCGGGTCCGGGCGTGGTGCACGCGCTGGCC
GGCTTGGGCAATGCATGGGCCAACTGTTGGCCGATGGTCCTCATCGGTGGCGCCACCTCG
ACAGACCACAGCGAAATGGGCGGATTCCAGGAAGCACCGCAAATCGAAACCGTGAGGCCC
TACGTCAAGATGGCCTGCAGGGCCGAATCCCTTGCTCGTGTGCCCTTCTACATCGAAAAG
GCTATCCGTGTGGCTATGTATGGCAGGCCCGGGCCGACCTATGTTGAGCTGCCTGCCGAG
GTGATCTACGATTTGATGGACGAGTCCAAGCTGGTGCTGCCGCGTCCCGTCCCGCCTCCT
CCGCTGACCTTCGCCGACCCCAAGTCCGTCCGTGCGGCTCTGGAGCTCCTCAAGACTGCC
AAGAACCCGCTCGTCATTGTGGGAAAGGGCACTGCCTACGCTCGTGCTGAGGCGGAAGCC
ACTCATTTTGTCGAAAAGACCGGTCTTCCGTTCCTTCCCACCCCGATGGGCAAGGGAGTG
CTGTCCGATGAGCACTCGCAGTCCGTTGCGGCCGCACGCAACCTCGCGCTGCAGTCTGCT
GACGTCATCCTCCTCCTCGGCGCCAGGCTGAACTGGATTCTGCACTTTGGCCAGACTCCC
CGCTACAACAAGGACGTCAAGTTCATTCACGTGGAGATCGCGCCCGAAGAGCTGAACAAT
TCTGTTCGCGCCGAGGTTGCCCTTGCTGGCGATATCAAGGCCGTCCTGACTCAGCTCAAC
GATCTCCTCGCCAAGAGCCCCTTCCAGTTCCCGAAGGCGAGCCCTTGGTGGAACGCTCTG
AAGGACAAGGTGGCGAAGAACAAGGCCGTCAACGCCAAGCTGTGTGCTGATGAACGGATG
CCACTGTCCTATTATAGGGCTCTCAAGGGAGTGCACGACACTCTCCCCCACGATGCCATC
ATCGTCTCCGAGGGCGCCAACACGATGGACATCGGTCGTACGATCTTTGACAACAGATTG
CCTCGTCATCGCCTTGACGCCGGCACGTGGGGCACGATGGGCGTTGGCATGGGCTTTGCC
ATCGCCGCCCAGGTCGTCCACCCTGGGAAGAAGGTGGTAGCCATCGAAGGCGACTCTGCC
TTTGGTTTCTCGGGCATGGAGATCGAGACCATGTGCAGGTATGGCCTCAATGTCTGTACG
GTCATCATCAACAACAATGGCATCAGCATGGGCGTCGAGGATCTGTCCGGCTTTGAGAAG
CCCAACGTACCGTTCTTTGTCTACACTCCCCAGGCACGCTATGAGAAGCTGATCGAAGCT
TTCGGCGGAAAGGGCTACTATGTGACCAAACCCGAGGAGATCGAGCCGGCCATGCGGGAC
GCCCTGGCCCAGAAGTGCCCCACCATCGTCAACATCATGATCGACACAGCGGCTACACGC
AAGCCCCAGGAGCACCCCTTCGAGCTGGGCGCCACCCTTGGCGGCAAGGCCAAGCTGTGA
>g452-273746-276154
ATGGCAACCAACACAGATACTGCATCCACTTCGAGCACTTCGCAGCCCAAGGGTCTGACT
GCCGACCTGACGAGTGGGGAAATCAAGGAGCAGATCCTCGAGATCAACAAATCGGTGGTC
AGCAAGGAGCCTCGTCACTCTGCTCGCGCGTTGCGTAAGTTCTTCACCAAGACCAGAAGA
CACATCAACCGCAACGTGCTCACTGCGACCGTCGATGAGGTGCTTGCAAATGAGAATGAA
CTCAAGGCCAAGCTGCACTCTTACGTTTCGAAGCTGCCCGAGCCTATGGAAACGGTGAGT
GAGGAGACCAAGCCCGCTGCTGCGGCGAAGACGGAAGAGCCTGCTGCTGCTGCTCCTCTG
CCCCTCCAGCCCGAGGTGGAGATTTACCTCCGTCTCGTCACCACCATCGCCCTTGTGGAC
AACAAGCTCTTTGACGAGGCTGCCGCTTGCTCCACGGACATGATCAAGAGCCTGCAGACC
TACAATAGGCGCACACTCAACCCTCTGGCCGCCAGGGCCTTCTTCTACTACTCTCTTGCC
CATGAGCACTGCAACCGTCTGGCCGAGATCAGGCCTACTCTCCTGGCCGCCCACAGAACA
AGCAGCCTGCGCCATGACGATGAGACGCACGCCACCATCCTCAACCTGCTTCTGCGCAAC
TACCTGCACTTTGGTCTCTACGACCAGGCCGACAAGCTCCAGGCTAAGTCCGTGATCAAG
GAGGACTCCGTCTCCTCCAACCAGCTCGCCCGCTTCCGTTACTACCAGGGCCGGATCAAG
GGCATGCAGCTGGACTACACAGGCGCCTACACCTACCTGCAGGAGGCGATCCGTAGAGCG
CCCTCGAACTGCGCTACTGGTTTCCGCGTTACGGTCCACAAGGTTGCGGTGATTGTGCAG
CTTCTGATGGGTGAGATTCCCGAGCGGTCGGTGTTCAGGACGAGCGGACTCAAGGTGGCG
CTGAGGCCCTACCTCAAGCTCACCCAGGCCGTGCGCGTGGGCGACCTCTCCGCCTTCCAC
GAAGTCGTGAAGACCTTTGGCGACGTCTTCCGCGCAGACAAGACCTACACTCTCATCCAG
AGACTGCGCCACAATGTGATCAAGACCGGTCTGCGTAAGATCAACGTCTCCTACTCGCGC
ATCCGTCTCGCGGATGTGTGCGACAAGCTGCGTCTGGACAACGTCGAAGACGCCGAGTTC
ATCGTCGCCAAGGCCATCAGAGATCAGGTCATCGACGCCACGCTCGATCACGAGGCCGGC
ACCCTCCGCTCCAAGGAACTCATTGATGTATATTCGACCACGGAGCCGATGGAGGCCTAC
CACGCCCGTATCAGCTTCTGCCTCAAGATGCACAACGAAGCCGTCCGTGCCATGCGCTAC
CCTCCCAACATGCCCAAGACGTCACCGTTGAGCGAGGAAGAGCAAAAGGAGAAGCGAGAA
GCCGAGAGCGAGATCCTGGAGAAGGTGCAGGACGAAGACGATGACGACGAGGAGTTCTAA
>g452-290416-291272
ATGGGTGGAGGTCCTCGTTTCCCGTTCCCCAAGTGGGTCTGGTCGCCTGCCGGCGGTTGG
TGGTGCGAGAACCCCCCGAACGCGCAGCGCAATCTTAGGATCGTCCTGGGCCTTAACTTC
GCCATTGCTGGCGCTGTCTTCTTCATTTCCGCCGCCAACGAGAGACGGCTGCTGAGCCAC
CCCACCATTCCCGTTCCGTCCCAGCGCTGGAGCGCGTGGACTAAGGTGGACGACCCTGAC
TACAAGCGCAAGCTCGCCGCGTACCACAAGAACAAGAAGCCCCTTTGGGAAAGAATCCTC
CCCGACGCCATGATCCAGGACGAGCACGGCCACCACTAG
>g452-314027-315078
ATGTCCAGCCAGACTTTCCTTCCCAACGTTCTTTGGGCTCAGCGCAAGGACAAGCTGTTC
CTGACGATCGACGTCCAAGATGTGAAGGACGCCAAGATCGAGTTGACCGAGACTCATCTG
AAATTCAGCGGCTCGGCTCAGGGTAAGGACTACAACCTCGATCTCGAGTTCTTCAAGGAG
ATCAACCCGGAGGAGAGCAAGTACGCCGTGCTGCCGCGTCACATCGTCTTCAACATCGTC
AAGAAGGAGAGCGGCCCGCACTGGGATCGCCTCACGAAGGTTGGCGGCAAGCAGTGGTGG
CTCAAGGCTGACTGGGGCCGCTGGGTGGAGGAAGATGAGGAGGATGAGGGTGCCGGCGAG
GGCGGCGATTTCGACATGCCCCCCGGCATGATGGGCATGCCCGGCATGGGTGGCATGGGC
GGCATGCCCCCTGGCATGATGGGCATGCCTGGCATGGGCGGCATGGGCGGCATGCCCGGC
ATGGGCGGCATGAACTTCAACGACTTCGGCGGCGACCTCGAGGGTTCTGACGAGGAAGAC
GAAGACGACGACGAGGTGCCCCCGCTCGAGGAAGACACCAAGGAGGGCCAGCAGTGA
>g452-323856-324880
ATGCCGCGCAAGCCTCACGCCCCCAGAAATCATGACCTCGTCAGGGGTATCTCGCGCTAC
TCCCGCAGCGCCATGTTCCGCCGCAGCGGCAAGGCTGCCGTGAAGAAGTCGGGCCACCAA
TGGAAGGATGTTGCCGCCACCAAGAAGGCCCCCAAGAGCGTCGAGAAGACGTTCAACAAG
ACCGAGAAGAGGACCGTGGTCGCCAAGGCCAAGCGCTTCTACCCGGCTGAGGATGTCCCC
AGGCCCATCCCCTCGAGGAAGAGCCACCACAAGCCCGCCAAGCTGAGGTCCTCGATCACC
CCCGGCACCGTCCTGATCGTCCTCTCCGGCCGCTTCAGGGGTAAGCGCGTGATCTTCCTC
AAGCAGCTCCAGCCCTCTGGCCTTCTCCTCGTCACTGGCCCCTTCAAGGTCAACGGTGTG
CCCCTCCGCCGCATCAACCAGGCCTACACCATCGCCACCTCGACCAAGGTCGATATCTCC
GGCCTGGAGGTGCCCGAGAAGTTCAACGATGCCTACTTCAAGAAGGGTAAGGCTGCCAAG
AAGGAGAAGACTGAGGACGAGTTCTTCGCCACTGGTGCCCAGCAGAAGAAGACCATCGAT
GCCCACAGGATCGCCGATCAGAAGGAGTTCGACGCTAAGGTGCTCGAAGTTGTCGGCAAG
GTCCCCAACCTGGCTGCTTACCTCAACGCCAAGTTCTCGCTCACCAAGGGTCAGTTCCCT
CACCTCATCAAGTTCTAA
>g452-399042-401028
ATGGCGAAGTACGACCTCACTCAAACGATTGCGACCTACTTGGATCGCCATCTGGTGTTC
CCCCTGATCGAATTCCTGAAGGAGAACAGGATTTATGGAGAGAAGGAGCTCCTGTCGGCG
CAGCTGGATCTGCTCTCCAACACCAACATGGTCGATTTCGCCATGGATATCCACCGCAAG
CTTCACGAAACCGAGCCTCCGGCCACGTTCGCGACCAAGCGCGCTGCCGTGCTGGCCAGG
CTGAAGGAGCTGAACGCTCTCTGCGCCCCTCTGGTCCGACTCCTGCAGGATCAGACCAAG
GTCGCTGAATTGAAGGAAGAGAAGAACTACAACATCGAGTTCATCCAGGAGAACCTTGGG
GTGCCCGCTGGCTGCTTGGATCATCTCTATGAGAGCGCCAAGTTCCAGTTCGAATGCGGT
AACTACTCGGGTGCTGCCGATTTCCTCTACCACTTCCGCACACTGAGCAACGACGCCGAG
AAGAAGTTCAACGCCCTCTGGGGTAAGTTTGCCGCTGAGATCCTCATGCTCAAGTGGACC
ACCGCCATCGAAGACCTCAAGGCCTTGAAGGAAGCCATCGAAGCCAAGACTTTCAAAACG
CCCCTTGTGCAGCTTCAGCAGCGAACCTGGCTCCTTCACTGGAGCTTGTTTGTATTCTTC
AACCACCCCAACGGTCGCAATGGCATCGTGGACATGTTCCTGCAGGACAAGTATTTGAAC
ACCATCCAGACCACCTGCCCGCACATCCTGCGGTACCTGACGACAGCCGTCATCACGAAC
AAGAGGCGGAGGAACCAGCTCAACGATCTCGTGAGAGTGCTGCAGCAGGAGTCGCACTCC
TACTCGGACCCCATCACCGAGTTCCTCGAGGACCTCTACGCCAATTTTGACTTTGACGCC
GCACAGAAGAAGCTGCGCGAGTGCGAGAACGTCTTGATGAACGACTTCTTCCTGGTCTCG
TGCCGCAACGAGTTCATGGAGAACGCCCGTCTTCTCATCTTCGAGACCTACTGCCGCATT
CATCAGACCATCGACATCAGCACACTGGCGCAGAAGCTTGATATGGACCAGGACGCCGCC
GAGCGGTGGATCGTGAACCTCATCCGCAACGCGCACCTCGATGCCAAGATCGATTCGGCG
GCCAACCATGTGATCATGGGCACGCAGAACCCCAGCATTTACCAGCAGGTTATCGACAAA
ACGAAGAGTCTGTCGTTCCGCACTCACGTCCTCGCCAACAACCTCGACAAATACGTCCAG
AGCGAAGGCGACGACAAGGCTCAGGCCTAA
>g454-157581-159048
ATGTCGTTCCGAGGCGGCCGAGGTGGTGGCGGATTCAGGGGAGGCGATCGCGGCGGTTTC
CGCGGCGGCCGTGGCGGCGATCGCGGCAGCTTCAGGGGAGGCGACCGTGGTGGATTCCGC
GGCGGCAGGGGCGGTCACGACTTCGCACAGGGTCCCCCCGACACCGTCGTTGAGCTCGGC
AAGTTCACGCACGCTTGCGAGGGCGAGATGGTGTACAAGATGGTGGAGACGGACAAGGTC
CCCAAGTTCAACCACCCCGTCTACACGCACGAGAAGGTCGAGGTCGGTAAGGTCGACGAG
ATCTTCGGTTCCACAACTGAGCCCTACTTCACGGTCAAGCCCAACCAGGGTTTCGTGGCC
ACGAGCTTCAAGGTCGGCGACCCCATCTGCATGGGTCCCTTCTCGCTGCTCAACAAGCAG
ATGTTCACCGAAGAGGACAAGCCCAAGGCTCCCAGGACGCCCGTCAAGTCGCGCGGTGGT
CCCGGCGGTGGTAGGGGTGGTGGCTTCAGGGGTGGTCGCGGTGGCGGATTCCGGGGAGGC
GATCGCGGCGGTCGCGGCGGTGGATTCAGGGGCGGCCGAGGTGGTGGCGGTGGTTTCCGG
GGTGGCGACCGTGGCGGTCGCGGCGGGTTCAGCCCGCGTGGCCGTGGCACCTTCACCAGG
GGCGCCTAA
>g454-230422-231438
ATGAACACCTCGTTCGTCTTCCTGACCATCGCGGCCGTCTTCCTCGCCCTGGCCTCGGGC
TTGGCCAGCGCACAAAACTCGGACATCCCGATCCCGTACGATCCCGTCAACCCGCCGGCG
CCGGTCAAGTTCAACCGCGACCGGTTCCAGATTTGGAACGTCAACACGTCGGCCTTCTGC
TTCTTCGACTGCCCCGACGTGCCCCTGTACCCCGCGGCGTGCCCGCTCACCTGCCGCCAG
TCCAACCGGACGAGCGTGCAGCAGTTCATCGTGTCGTCGGCCTACAACAAGATCGTCCAG
AAGGGCTGCTGGATGGAGGCCCCCGTGAGCGAGGTGGGCTTCCTTGGCGGCCTCGTCGGC
GGCTCGCGCTGGTGCTGGCCCAAGAACGCCACGGGCGCCGGTGGCGTGGGCCAGGACGGC
GACGTGCACTGCAAGGGCGCCGTGGGCAACGTGCCGCTGCTGCCCTACGACTGGATCCGG
TTCCACAAGATCGCCGGCGTGCAGGCCAACTTCTTTTACCCGTACGACACCATCCGCATC
TCGTCGACCAAGAGCGGGAGCTACTGCTCGGCGCACCCGACCGAGTCGCGGTTCGACTGC
GAGAACAAGCCCAACGCGTGGCAGGGCGATCGCTTCCAGCTCATCTTCGTCGCCTCCAAC
CTTAACGAGGGCTGCCCCGGCTTCGACTAA
>g457-83916-84553
ATGCAGAACGACGAAGGTGTTGCCGTTGATCTCTACATTCCCCGCAAGTGCTCGGCCACC
AACCGCCTGATCACTGCCAAGGATCACGCCAGCGTGCAGATCAACGTTGCCAAGGTCGAT
TCCGACGGCAAGATCATCCCCGGTGAGTTCGAGACCTTCGCTCTCTGCGGCTACATCAGG
GCTATGGGTGAGGCCGACGGTGCCTTCAACCGCCTTGCCAGGTCGCATGGCCTCGTCAAG
AGGGTGTAA
>g478-59856-61765
ATGTCGATGGAGGGAGACAACAAGCGCGCGACCACCATCATCAACTACGAGTCGCTGGCC
CGCGACCAGGTCAAGAAGGAGGTCGACGGCTACATCAAGCTGTTTACCGCCGAGGATGCC
AGCGTGGAGGAGCGCAAGGCCAAGTACGAGACCCTCGTCAACTCCTTCTACGATCTCGTG
ACCAACTTCTACGAGTACGGCTGGGGTCAGTCGTTCCACTTCGCGCCGCGTCATCGCTTT
GAGTCCTTCGAGGCCTCGATCGCCCGCCACGAGATGTACCTGGCCCACCGCCTTCATCTC
GAGAAGGGCCAGGTGGCGCTCGACGTCGGCTGCGGCGTGGGCGGACCCGCGCGCTGCATC
GCCCGCTTCTCCGAGGCTAACATCGTCGGACTCAACAACAACGATTACCAGATCGGCCGT
GCCAAGCTCCTCACCAAGGAGGCCCGCCTCGAGCACCTCATCAACTACATGAAGGCCGAC
TTCATGCACATCCCCGCTGAGGACAACACCTACGATGCGGTGTACTCGGTGGAGGCCACC
TGCCACGCTCCCGACAAGGTGGGCGTCTACTCGGAGCTCTTCCGAGTCCTCAAGCCCGGC
GGCCTCTACGCCACCTACGAGTGGGTCGTCACTGAGTCGTTCGACGAGAACGACCCCGAC
CACGTCAAGATCAAGAAGGGTATTGAGATCGGCAACGGTCTGCCCGAACTCGAAAAGCCA
TCGCAGATCGCCGACGCCCTCAAGCAGGCCGGCTTCGAGATCATCGACAACGTCGACGTC
GCCAAGAACGCCGACGCCGAGACGCCCTGGTATCTGTCGCTCTCCGGCTCCTTCTCCCTC
ACTGGCTTCAAGCACACCCGCATTGGCCGCAGCGTCACCCACATGGCTGTGTCGACGCTC
GAGTACCTCAAGATCGCCCCCGAGGGCACCACCAGGGTGTCCCAGATGCTCTGCGAGACC
GCCGACGACCTGGTCGAGGGTGGCAAGAAGGACGTCTTCACGCCCATGCACTTCTTCCTC
GCCAGGAAGCCCCTCGCCAAGGACGAGTAA
>g481-21186-24010
ATGGACGAACACAGCGCTTCCGCCAACAGCCCCTCGGAAACCGCCCAGTTGGCGGCGGCC
CCTGCCCCTGCCTCCAAGTTGAAATTCAACGTTAATGCGCCCGCCTTCAAGCCCTCATTC
AATGTGAATGCTCCCGTCTTCAAGCCCAAGGCTGCTGCTCCTGCGACTGCGGCGCCCGAA
GAACCCAAGGCCACCGAAGAGGAGGCAGCCCCGGTCTCCTCGAGCACCACTACTACCACG
GCGACCCCAGAGCCTGCTGCGGCCGCTACGGTGACGACACCCGTCGCAGCAGAGCCCGAG
GAGCCCGCACATGGGGAGGAGGAGGGCGAAGAGGCCTCTGCTGTCCCCGAAACCACGTCC
GATGCCCCCAGAAAGGTAATCGATCCTCGCGACCACCTCAGCATTGTACTCATTGGGCAT
GTCGATGCGGGTAAATCCACCACGGCCGGCAACCTCTTGTTCCTCACGGGGCAAGTGGAC
CAAAGGACGATCGAGAAGTATGAGAAGGAGGCCAAGGAAAAAGCTGGCGGGAGCTGGTAC
CTGGCCTTCATCATGGACACCAACGAAGACGAGCGCGCCAAGGGCAAAACCGTCGAGGTG
GGCCGAGCGCACTTTACCACCGAAACTCGTCGCTTCACAATTCTGGACGCCCCGGGTCAC
AAGAACTACGTGCCCAACATGATCGGCGGAGCAGCGTGCGCGGACGTGGGCATTCTGGTG
ATCTCGGCGCGCATCAACGAGTTCGAGGCAGGGTTCGACAAGGGCGGTCAGACGCGGGAG
CACACGATGTTGGCCAAGACCCTCGGGGTGAGTCGCTTGGTTGTGGTCATCAACAAGATG
GACGACAAGAGCGTGATGTGGTCGAAGGAAAGATACGACAGCATCCTGCAGAAGCTCCAA
CCCTTCCTGAAGCAGGTAGGCTTCGGACCCAAGGACGTGTCGATGCTGCCGATCTCGGGC
ATCACGGGCGCCAACTTGCTGCAGCCGCTGGACGCGAGCGTGTGCGACTGGTACCAGGGT
CCGTCGCTGGTGCAGGTGCTCGACGGGCTGCGGCCACCCAAGCGCGATCCGGCCGCGGCT
CTGCGCATTCCGATCTTGGACCGTATCAAGGACGCCGGCAAGGTGTTCATCGAGGGCAAG
GTCGAGTCGGGAACAGTGCGCGTGGGCCAGAAGGTGGTGGTCATGCCCACCAAGCAGGCC
GGCGTGGTGCTGTCCGTTGCCTCGGACTTTGCGCCCGACCTCGACGGTGCGGCGGCTGGC
GAATACGTGCGCATCGTAGTCAGCGGCGTGTCGGACGACCAGATTCGGGCGGGCTTCGTC
GTTTGTGACGCTGAGCACCCGATCGTGGCCGTGCCCCAGTTCGAGGCGCAGCTTGCGGTG
ATCGATCTGCTGGAGCACAAGCCCATCATCTCCCCCGGCTACACCGCCGTCTTCCATGCC
CACACGGCCGTCGAGGAGTGCACGATCAAGCTCATCCTCGGCACGATCGACAAGAAGACC
GGCGAAGTCTCCAAGCAGAAGCCTAAGTTTGTGAAGAAGGGCGCCTTTGTCTCGGCCCGC
ATTCAGCTCACCCAGCCGGTGTGCCTCGACACCTACAAGGACTTCCCGCAGCTCGGCCGC
TTCACGCTCCGCGACGAGGGCAAGACCATCGCCATCGGCAAGGTCACGCGCCTCCCCGGC
GCCCGTCCCGGCGCCAAGTAA
>g481-46936-48398
ATGCCCAAGTCTCGCAGAAACAAGATGGTCGCCCTGACCAAGACCGAAAAGAAGGTGGGC
CGACGGAAGGAGGAACTGGTGGACAACCTGCGGAAGGGTCTCGAGGAGTATGCGTCGGTG
TACGTGTTCACGTTCGAGAACATGCGCAGCACCCAGTTCAAGAGCGTGCGTGCTCGGTGG
AGCGGCTCGCGCTTCTACCTGGGCAAGAACAAGGTCATGCAAATCGCCCTCGGCCGCTCC
GGCCAGGACGAGGCCGCGCACGACCTGCACAAGCTCAGCCGCCAACTGGTGGGCAACTCG
GGTCTGTTCTTGACCAACAGCCCCGCGGACGAGGTCGCCACGTTCTTCAAGGACTACGCG
GAGAGCGACTACGCGCGAGCAGGCTTCGTGTCGACGCAGACGGTGGAGCTGCCGGCCGGA
CTGCTCCCCAACTTTGCGCACTCGATGGAGCCCCACCTGCGCAAGCTGGGCATGCCCACC
CGCCTCAACAAGGGCGTGATCGAGCTCGACCGCCACTTCACCGTCTGCAAGGAGGGCGCC
ACCCTCACCCCCGAGCAGGCCAAGATCCTCAAACTGCTGGAGATCAAGATGTCCCAGTTC
AGCATGGCGCTCAAGTGCGTGTGGAAGAGGACGAGCGACTTCGCCACAGGAGAGGGGGCC
TTCGAGATGCTTGCCGCCGAGGGCTCCTCGTCCACCGGCCAGGTGGATGGCCCCGACGAC
GACGACGACGAAGATGACGACGACTTGTCCGAGTAG
>g481-52747-54938
ATGAGCGGAGTTCGTTTCCTGTCGCTTGTGCGGCCCTTCATGGGCGTCCTCCCCGAGGTC
GCTCAGGCTGAGAAGAAGATCCCCTTCAGGGAGAAAGTGTTGTGGACGGCCGTGACGCTG
TTCATCTTCTTGGTCTGCTGCCAGATTCCCCTCTACGGCATCGTCTCCTCCGACAGCGCC
GATCCCTTCTACTGGATGCGTGTCATTCTCGCGTCGAATCGCGGTACCCTCATGGAGCTC
GGTATCTCTCCCATCGTGACCTCGGGTCTCGTCATGCAGCTTCTCGCTGGCAGCCACATC
ATCGAAGTCGACCAGGGCCTCAAGGAGGACCGCGCCCTCTTCAACGGTGCCCAGAAGCTG
TTCGGTATGATCATCACTGTCGGCCAGTCGATCGCCTACGTGTGGTCGGGCATGTACGGT
GATCTCGCCTCGCTCGGAGCCGCCAACGCGCTGATGATCATCATCCAGCTGTTCATCTCG
GGTATTATCGTGATCCTGCTCGATGAGCTGCTCCAGAAGGGTTACGGTCTCGGCTCGGGC
ATTTCGCTGTTCATCGCGACCAACATCTGCGAGAACATCGTGTGGAAGGCCCTCAGCCCG
ACCACGATCAATACCGGCCGCGGCACCGAGTTCGAGGGCGCCCTCATTGCGCTGGTGCAC
CTGCTGATCACGAGGACCGACAAGGTCAGGGCGCTCAAGGAGGCCTTCTACAGGCAGAAC
CTGCCCAACGTGACCAACCTGCTGGCCACGGTGCTCGTCTTCATGGTGGTCATCTACTTC
CAGGGCTTCAGGGTCGACCTGCCCGTCAAGTACCAGAGGCAGAGGAGCGGCCCCGGCACC
TACCCCATCAAGCTCTTCTACACCTCCAACATCCCCATCATCCTCCAGACCGCCCTCGTC
TCCAACCTCTACTTCATGTCCCAGCTGCTCTACAGGCGCTACCCCGGCAACGTCTTCGTC
AACCTCCTCGGCCAGTGGCACGAGTCCGAGGGCGCTCAGCACATGCAGTCCGTGCCCGTC
[truncated: 379,437 more chars]
