# Supplementary material for: Diversity and evolution of the emerging Pandoraviridae family
Source: Nat Commun. 2018 Jun 11;9:2285. doi: 10.1038/s41467-018-04698-4 (PMC5995976; doi:10.1038/s41467-018-04698-4)
Supplement: Supplementary file 6 — Supplementary Data 3 [file 41467_2018_4698_MOESM6_ESM.rtf]

Pandoravirus inopinatum genome reannotation

LOCUS       Pandoravirus_inopinatum2243109 bp   DNA    linear       19-SEP-2017
DEFINITION  Pandoravirus inopinatum isolate inopinatum.
ACCESSION
VERSION
KEYWORDS    .
SOURCE      Pandoravirus inopinatum
  ORGANISM  Pandoravirus inopinatum
            Unclassified.
REFERENCE   1  (bases 1 to 2243109)
  AUTHORS   NA,N.
  TITLE     NA
  JOURNAL   Unpublished
REFERENCE   2  (bases 1 to 2243109)
  AUTHORS   NA,N.
  TITLE     Direct Submission
  JOURNAL   Submitted (19-SEP-2017) NA
COMMENT     ##Assembly-Data-START##
            Assembly Method       :: NA v. NA
            Sequencing Technology :: PacBio
            ##Assembly-Data-END##
FEATURES             Location/Qualifiers
     source          1..2243109
                     /organism="Pandoravirus inopinatum"
                     /mol_type="genomic DNA"
                     /isolate="inopinatum"
                     /host="acanthamoeba"
                     /note="[virus wizard]"
     gene            1689..3083
                     /gene="pino_cds_1"
     CDS             1689..3083
                     /gene="pino_cds_1"
                     /codon_start=1
                     /product="Ankyrin repeat"
                     /translation="MGLAPADDDGDQSPRTDALPLPNELLAAVLAHLDPVDSVAASRV
                     QRLWRAFALPRAARFGPAYAAHLAARGHLKVLQWARADGCPCDESASTAAARHGHLHI
                     LQWLCDNGYPLSAVVASAAARGGHLAVLQWLDDEQRLHDMRAPGGATAVDRFFRVIKL
                     MLADRHGYCGDLCDEAAHGGRLETLQWLRANGHPWSVSTCSTAAAYGRLDVLRWLHAN
                     GCPWSESACMRAADKGHLEVLQWLHASGCPWDASACMRAAENGHLDVLQWLHANGCPW
                     DASACTRAAESGHLEVLQWLHANGCPWTKSACAGAAENGRLEILQWLRANGCPWGAST
                     CYLAALGGHLEVLQWARAHGCRWNKRATAYAAKANRLKVLQWLHASGCVCDERTCSRA
                     AKNGHLGIVQWLHANGCPWDEETCYAAAAQGHLDVLQWATANGCLWDPPTCSLAAKRH
                     PAVLAWISATTASS"
     gene            3283..4740
                     /gene="pino_cds_2"
     CDS             3283..4740
                     /gene="pino_cds_2"
                     /codon_start=1
                     /product="Ankyrin repeat"
                     /translation="MEQCRSRPCLPRLPLPNEILAMILAHLDPIDAVAASRVQRLWRA
                     IALPYAAEFDAAYAAQLAARGHLEVLQWAMSDGCPWDSLACTHAADAGHLDVLQWLHD
                     NGCPWDHWACACAADSGRLDILEWLYANGCPWDEQACSAAAEAGHLEVLQWLCANNCP
                     WDGETCAAAARRGHLKILQWAKANGCPWDAKACACAAKGGHLEILQWLRANDCPWDEW
                     TCTRAAGRGHLEIVQWARANGCPWNGSVCERAAKGGHLDVLGWVCANGCPLTEHTFTV
                     AVKAGRLDIVQWLYANDCPWDERHFFFSAAKRGHLDVLKWIHVNLCDWDGSACDGAAR
                     GGHLEILQWLCANGYPWGAETCAQAAEGGHLEVLRWLRDKGCPWDSTAFERAAQGGHL
                     DVLQWLRANGCPWDERACASAARCGQLGALQWLRANGCPWNAETCASAARNYHQKTLW
                     WARNNGCPWTEPIHSRSNEDGCTGPTVHTNADDAP"
     gene            complement(5022..7661)
                     /gene="pino_cds_3"
     CDS             complement(5022..7661)
                     /gene="pino_cds_3"
                     /codon_start=1
                     /product="Sam domain"
                     /translation="MATTPRSFGWMADKRLKSFWQHNRQWPDLDYNIMDRIEAACAAL
                     GRPLPRCGPPKDRWHVGGMDQAMARIRELHAYNNRQALEHPARDDHDACLDHQGDSAS
                     TSPGVLFSRWFNAKGARLHGIDPETTTAKEAWAYMQSRPTSPRAPRWCTSYWDELSRH
                     ATSDALSAVVPRRTPDGSSVSTIDLFRRATGTSTESRGQRLYAEIMSGLERSGTWQFA
                     KKWAESGAPQRVVDLDRVGPFLRDVCTLLGDTVERDRADAYRHGPACARLFGAAVITA
                     APPISDRDAAIEPFVGDASTAGRSNEPVPVAAKQSSTLEEAARCRVCSLESSSHLAVG
                     LAKDVSRQDHSCGAPSPPAESPLQTKEHGVRRFRFLLSEDDPLDETDTDDDQGDCEGG
                     NHDIDLGDQPPAAVHFHERPGTTVGGQKSAGSLPLAGRNPSDAPHVSDWNADEVGDWV
                     ATCDNGCLAGYREAFVENCITGRNLQDIANRGLVDCLGILQQMGVDRVGDALDLWRLI
                     GDLVSTCSRSFPSNPATQSPEVPLSSSSTLRSVAGGTKRRSEAPTRPNAKRPRTAGDE
                     SLPRASATGDTGNAAKDAPPKCARCSASPVPGHTLCEPHRLAQNARQREYDRKRAKSK
                     QQHVGERNKRQKEARPLQKHGRLDGTTDGHSTPCASQTQSTVCGEQPQRGARRHAEPH
                     RTSKEGVDTVDGIMRYYTTCTTPAAVASRNLGGDHDHRHTASVQDELGDMAPTDDQQM
                     RADPPTVGIVLADQATPIPPSLDAHVVDCATTKARDEIESTKQGGAPTKRKWIDQETI
                     DDEGTETEDEELANEQGAPPSPVEVDYPPMTPRDLLALWPERRRAAQDCAAKGGDDGD
                     DDGDTKEDDNAESNWYVQRNVQA"
     gene            8070..12251
                     /gene="pino_cds_4"
     CDS             join(8070..10312,10724..12251)
                     /gene="pino_cds_4"
                     /codon_start=1
                     /product="Ankyrin repeat"
                     /translation="MSLVPKTKGIHKMRHRRVDMTTLPPEVIAAVVSWLGPRDHASAV
                     LASPRFGVLTPEERGRLHFARFAPKVLARMGSLDGLCYWHGVDPWRIDIHCLRAAAGG
                     GHRALVEWILDKSTGGGCPTEALCAAARGGHVDLMRWLIDERQAAILKEVLVDAISSK
                     RIDAVRLILDRAHVIDHREAVESGICLDEDGAECSSQDDDHGDDRDDNDDDDGKDGSG
                     DGDDDGDDGDDVEENARHDSCGRDWGTRPMRAAARTNDADIMQLVYDQCYYGDDPEVL
                     GRPLSAAAESGAAVAVQWILARYADRDASADAFGAALRWTRRDCAVAILSRWPDVARP
                     VDIYDEPFRQLGGTSIPVLDAALAVALGTQPDTWPLSVAGATAEPGQIYDALLVHCLS
                     REFKDDHSSLATTLLGGAPCDAVFRWAVDTARCVPAVYVACDMARKGMTGRLAYCRER
                     GLLTPVHVEVAMTGAAAAGRVDVLTYMWSSLAAGDGDTQAADAVRANVQKMADAAAES
                     GDWTVVEWLQKHVDRRAHCTAAAFAVAANKGHAAFLKRLYAVGADRCRHPCDAPCEPV
                     PGWSPFLFALRTPCGLYNVNDLWRNVARDGHYDVVQVLREHGALDGVYLRSEAALNGH
                     VAICALDVAINGPCKRDLMIQYAVQARDRACLAFALANGSTWEPLGRWSIHPMCTAVK
                     RAAAPSGICWPGTGRRSTTTFRPTGRMKRATLTRKAINPITIFSSFPLCLPCASVFVP
                     VRSREANKRKAERAQMKRIRSPAAANADRMVQDTDNVDDDNGHGKDEPDQTDPKRPRT
                     DQEASGSIIGNGGAPDASDIYDRDIQTLDARIGRLQAGAANAEDIGEIMRLVAGHIES
                     GTQAARRGGGFYPRLRKAYDGLWEALTWEVGAAAAVAEAARAGRISGWPPTFADVQSV
                     YAEMEPATADRHMAQIETEAAVGLTGAGQSLGIALGQAVRRRAAAASLDPFAFSLVAD
                     AHILAQSLACMPTNVYYVLRFYVDSQAWAMLVNLTPHLGHSPLVAAISVPNNAKRVQY
                     RMRVAAPAFALSPALQPLIRHVGSFLASLARSPPWENPVPLDAISSLLLDPLPAGVAQ
                     IDALMLPVPSALALPSAVQYALTLIGPENTDDVDDGALYGAEVDECHLALAIRLFAGQ
                     IDARLMSAAARRRARTSLVQGAAAAYTGPLNIDLDLAPRETLDPAAAYAWQRTCAAPA
                     LPDGRLPGSARLVEAAAVWGVDVDPLVAVRRPELLCHQLAIEASARLRAPAP"
     gene            12695..13720
                     /gene="pino_cds_5"
     CDS             12695..13720
                     /gene="pino_cds_5"
                     /codon_start=1
                     /product="Hypothetical protein"
                     /translation="MYYDLGVAHLACPAGCMCPWPLPAGSESTEIAAIFAYLPSLALE
                     EIFWHHLVGEALRAATAALADGKNEDDDVTDVGVLCAWLAAAYTPLRGSPEFRRVARR
                     LSAAWPGIADAHFGVRGLAWHGVSPAPRPGHGSATVVESGRRVGKTTVTVRLSRVLAR
                     RVKHVVCVVANGGDLDQVFAGLGVPEYEAAHTDDSRGFDAANVGHMWEAIGPLVRAYA
                     REGVLVLVDDLHWDTEQGALLIDEITNLGVHLVVTSQRMTPRRGLCPDRLVWMLRFKT
                     PWALYGAESFSCVTGIDHHRIVAAYDAHMRNDTTRFPRATLVAQRRRDDTWDLQIMPH
                     AALSPDP"
     gene            14280..15269
                     /gene="pino_cds_6"
     CDS             14280..15269
                     /gene="pino_cds_6"
                     /codon_start=1
                     /product="Hypothetical protein"
                     /translation="MSRRCLGVAHLQTRPCPWPLSLDPTRCAVDPVFDALPSLALEEV
                     FWYHLRNEALGDVANKDEDGDDAKDAFALRAWLGSCKPLRGSPEFCRAARRLLATWPQ
                     PARHDSTDIAWRGVLPPSSRGAGTVTYVWGWRASGKTTAALRIARVLTKRVQHVLCAV
                     HHRREVVDKMFGALGITLDRIIHAEAPLHEYSVAEVWEAAGPLVRAHAHEGVLVIATG
                     LSGDDPGAATVFKQARDIGVHLVVTSGRPPVPALVPHMDRVVLCCKCNAKVWSVYAFD
                     DMGIDHCAVNEIYRACIIDEAARGTIVFQRRRDDTWDTQVMPYMIPLAAAPPV"
     gene            15774..16796
                     /gene="pino_cds_7"
     CDS             15774..16796
                     /gene="pino_cds_7"
                     /codon_start=1
                     /product="Hypothetical protein"
                     /translation="MHFDLGVAHLTHPDGHPCPWPLPVDTGHSDAVASFAYLPSLALE
                     EVFWHHLVGEAIGAAAAAMADEKNKDDDVTCTEALCAWLAAAYTPLRGSPEFCRVARR
                     LLAAWPEIADVRLGACDLSWRGVSRASRSGGGSVTYVGGMRWAGKTTIAVRIGRILAR
                     RVKHVVCVVAVDGCIDRMFASLGIAEYEAARTDSGHGYDVFNVDRMWEAVGPLVRAYA
                     REGVLVLVDDLYWNTEQGAPLIDEIANMGVHLVVTSQAGALCKDLCPDRLIWMCTRVG
                     SWASHAIGSFSCMSRTDRDRMVAMHDAHMRDYSARPALVAQRRLDDTWDLQIMPYEPT
                     PVPPPM"
     gene            17065..18435
                     /gene="pino_cds_8"
     CDS             17065..18435
                     /gene="pino_cds_8"
                     /codon_start=1
                     /product="Ankyrin repeat"
                     /translation="MESGRATDSPGLGDLPMEILALIVAHLTADGDLCRARAAHRCFH
                     ASPQGDAEKRLARWRGQKTPEQFCAVGLVEALAILTDRGAILGPTCVWAAVEHGHCDV
                     LAFLVARGVSLHVGPRRRIGAVRAPAGFVSRATRRVAPHTDLIATAASGGHLGVVQFL
                     HAHGVMSTPSPAALDSAAANGHLDVIRWLHATDRALFTATTDAMDKAAAAGHLDVVRF
                     LDEHRDEGCTEFAMTKAAIHGHLDVVAYLHEHRTEACTDTTYMEACLADQPETSYYLL
                     VHNIGADPTEFQAPRLSEQFMGFFLVAIGSTETSSKVFEIDEPTGDGRGDAHRDNDDS
                     SHSARMTWALDALGSRADVMNLAARHGFLGVVEWLHKRGTAGCTTDAMDGAAIAGHLH
                     VVQWLHVHRSEGCTEAALASTHPASCGSCSRIGPPTAAAPSRNDAAPPPPAKKRIKKA
                     GLLADA"
     gene            complement(18851..19348)
                     /gene="pino_cds_9"
     CDS             complement(18851..19348)
                     /gene="pino_cds_9"
                     /codon_start=1
                     /product="Ankyrin repeat"
                     /translation="MDAVRAGHLAIVRYLCEPPAGGGGAPLCAPTAAIYFEAAGAGHW
                     HVVEWAAAAGVPWDMSSVVMALHRLSQRTVAQRAGYVRLVDDGIWHCSPALRELVGSW
                     PCADDTGGAVPVNQPVPQEHAIRRLYSVFSGFLGCDNAPPTPGNIATVDMFFGLVGRA
                     LASGP"
     gene            complement(19433..20861)
                     /gene="pino_cds_10"
     CDS             complement(join(19433..20073,20152..20298,20549..20861))
                     /gene="pino_cds_10"
                     /codon_start=1
                     /product="Ankyrin repeat"
                     /translation="MDDPHHVWHVVGRGSAGTALCRRDRHRNGRAVWRVRCETRRRVP
                     NPRWHGAGPLPLGARWAGDTRDSPHLVETTVRETLVPGPCHVRPSVDLVVAALAMADN
                     AQSTRDSTAEPTIQPPLDCIKPPMSINDALPNEILLEILTRPEIDPTHIVLDCHAANH
                     GFIGLFEWMDAIGRPRTPLACRAAAFGGHVTTLAWLIEHGWPVDMLVYSRAARKGHAH
                     VLTWLLSRPDALAMDGTVLDDAIHGGCATDHLEWLVRVGAPWPTRDARYPMYTAGQSG
                     KIEVIEWVAREGPQPTAWIDAICGVAEADNVGLLDALHAAGHFQDAQGNRKPLDGAFF
                     WASESQSLAAMAWLADHDPGLTLDDDAIRERA"
     gene            complement(21149..21790)
                     /gene="pino_cds_11"
     CDS             complement(21149..21790)
                     /gene="pino_cds_11"
                     /codon_start=1
                     /product="Hypothetical protein"
                     /translation="MTTIARSGSATEHTKRRRWGPRRGAKALAKSVLSRTETAAQQHK
                     KEHEVDALLVRCDGTATTVTVDRRTGRGMAKALGCVGICRWPYAYTLDTVVARDGRTY
                     RYDVWIDAEASPIVELGVIRRPRTSPDNLYASLAAHPLNEATDHVIGGSALLVSVVEE
                     GSGRHLRKADWEHIWAAVSGVTDPCDGDVDEVVVANCHGATALCRRAGRERFS"
     gene            22193..23895
                     /gene="pino_cds_12"
     CDS             join(22193..23405,23501..23895)
                     /gene="pino_cds_12"
                     /codon_start=1
                     /product="Ankyrin repeat"
                     /translation="MPSLGKRPASSQNCTLSTDAVDADGHNGNVGQPMLCACSDGNVR
                     LCDDDQGTDKTEWGRPAKVRRVDSGYSIEDMPPEILVMILNLVSDGASFVACRMASRL
                     FWTYSPVDFAVAHVSPQALIASRVPPDIARAVFARRGDTPCWFMLPLAADTGSIRTVE
                     WVSRLLCDFHAPNSQLCIGSGDNVLDDDDNRQEALQDDDGDDDDDDGTLEHLADFVYH
                     RPLMVRRSALKDDPFDLWGCFRTHVDEAAYIAARAGTVDIIAHLHETFRKPGPECGCS
                     PSVGKVAFHAARHGVLEWLADVGCDGRYRPGCMSVQDAVNKGNPALVEWTVRVAKPWH
                     RFVTWPMVHTLAVRNNVPMMDLVARLGLYPLSSDDMAIAAGAGSLDVVRWAAGEAIEG
                     VVGAHDGPVWPSGASAAQRALAKGHYEIVRLLGEAGRADFGRWDALFQAVSSGNLDML
                     SFVADNGGIYRPNVLAAALEGRHAGIVGYLCDKYTPSYADMIEAIHTANQTYDTETTD
                     LTVDWLVEHVSAAASLQPMLQRSHPKE"
     gene            complement(23948..25552)
                     /gene="pino_cds_13"
     CDS             complement(23948..25552)
                     /gene="pino_cds_13"
                     /codon_start=1
                     /product="Hypothetical protein"
                     /translation="MSETGTKNNDRGEPTEATRLLPAPSSSSSDRRCIDATSHATIRP
                     TTLREWMRDRVWAVSVWMTLAASVYWVATLVPTYAVFTLTDEIADRPGDGAYTTKALM
                     SIARGVAMPLGFASTLSGTVRRTVVGGWRRPVGLVVYSLLFTGVPNLLFAVDDVAVRV
                     GSRFVGSLFFSWAFCAYIDYAQGRRASDVIMPIATACMLLSAPLSRAFSPLVGRHVLG
                     VCAADDSDCPGDVHAIADQRQLYRWMPTVVSLLLLAPMVAGAVALGASPPPNEIDQRT
                     RMRRVTVSAAADRAWLWRHWAPLAGMSASNAVLQSVRVVRDVFAADLLGADAPWWHWI
                     LADVPACIGACLFYAPFGLIGGHRKAFLVVTSLGLVSAALMTTAGLLGVAGAIPPLAF
                     LVLSGIGYFVAVVPFAGGGVVFERLIAASRMPIDSMLVNVACQLPAYAVSLAAVVATP
                     LADDMGAYFCWTTLLGGGVLIVSYLWTLASGWRVLAPDDPLPHETWEDLSLGDRFCDA
                     DPKDDHEYADPGEPRHDAHALRPLTK"
     gene            complement(26029..26736)
                     /gene="pino_cds_14"
     CDS             complement(26029..26736)
                     /gene="pino_cds_14"
                     /codon_start=1
                     /product="Hypothetical protein"
                     /translation="MEDAKRTVKETAETAATSSTQDAGKNSTLENSNGLNGRRGQTAH
                     DNDDNDNDEYINIHIRVPKANATKLLEDMMSEYSEDSCCAGWGTDIDIDIDADARKRL
                     APGGNVARHSREMLNVAIAQIHRHCGGWWSDRGPRVDQQKAWDLAFYPLDEWVALPVP
                     YGMRDRVAYPNAVVEERPSDWDSPVLDESADPFAWCSEWSDGTMIARSTDPFETAPVE
                     STDTMAVPAEPTTLAAP"
     gene            complement(27182..28198)
                     /gene="pino_cds_15"
     CDS             complement(27182..28198)
                     /gene="pino_cds_15"
                     /codon_start=1
                     /product="Lipase/esterase"
                     /translation="MGSSTSRPAVAAAASMPSPVRIGRPPLHPDIVQLIKSVCRMPAL
                     QSVEHGRTLLDAVQREAYHPLRLAPAVITSAVVPVDDDVAEGTVSIRVVRALWADPEG
                     DCLLPALVYVHGACGDFATHERLMRALAYCTGAAVVFVDYARMPDAPWPAAERQVFGV
                     IAWLAACGDRVGVDGTRLALVGDGIGAHIAASAALLAAADSDMAPRIHAQVLVCPILD
                     APQHLSPDEDKICLPWTTQDALARTWSAYAPDATRSPLTAPVSRLRLLPPTLVVTGER
                     DIAAPHGRTYVDALIRAGAPVQAASYADAFHDFWVLDALADTPSAGATTDLVAHFVAD
                     AFSR"
     gene            28829..30526
                     /gene="pino_cds_16"
     CDS             28829..30526
                     /gene="pino_cds_16"
                     /codon_start=1
                     /product="Ankyrin repeat"
                     /translation="MQETACGVGLARLPPELMDHIASFLPRVADIAACLCASPLFRPM
                     RIDSAVVRLFGGNVVDVIERGAPLAIVEVVRGRSPATDALLLPAVRGGRLDVLERFCG
                     SPMRPLFCTPGLLPDAGLVRCYSHDTGLVHWGYAEEKDHRGYPSSGVGALFEAIACGR
                     ADMVDRLLDFGAIASVTPPPPDLGVECMLRAARKGHLDVMQVIHRRIVGGTLPRRASS
                     TAGCGCAPLVGQSAFNSDQPEAAEWLDRCGCDGAYRPVNNSLGLAVEAGHFGIARWLI
                     DKAETHGAKYDVGPFSVFLAAKLGAAAVVALVYDWYPLRHQDSDPDRNLEIGQVLAEA
                     AARGHMDILRWAAGDPCPIPTCARGASPCPSGTLDARHGGPPPPGASTSWSGSSAGPM
                     PHASCPSRPLMRVSRVDTSTLPSRRTAPASPPLTSGARSTPLCDLATSRLSPPSPTPE
                     PNTGARPLRVPLNEVTSRLWPICANGMARPTHSTRSTALTGLPKPPGPSCAGYVTMSR
                     ACARRASTDQGAPLCATVGRERRERPAAVLEDAPHRDAVPAGTNQPCFGWPVHFVRG"
     gene            complement(31108..32094)
                     /gene="pino_cds_17"
     CDS             complement(31108..32094)
                     /gene="pino_cds_17"
                     /codon_start=1
                     /product="Hypothetical protein"
                     /translation="MAEPGRGSTAHVLVGLPVELWAMIAGRCADDRSALIALAGVCHT
                     LQGIVAQLTTGKIERTRALIDAVVDAWERLSTDANRAWAAGIDCGRCAEPHDDDLGGG
                     GTPAPTLPRANVGKCTHLVRRYVVDQLGSDWMLCDDCAAEAIQRLHECGLGQAAPVVV
                     QRVGLEKRHIWRTFHNFVPSHLVDGGHFALPAGLAHCVNEKAAARLAALTTLPPTHYF
                     DDESTRLLPLPSARSWLPVGPAHAEHAHGTQYRGLFVCCDKTHPMWGVVAAACVGARG
                     LVFNDWWIAYPHMGALMADYREQRKAMRRADIISWVTSVAFDQDDAEAEIIR"
     gene            complement(32826..34052)
                     /gene="pino_cds_18"
     CDS             complement(32826..34052)
                     /gene="pino_cds_18"
                     /codon_start=1
                     /product="2-isopropylmalate synthase"
                     /translation="MLPSLPCKRKREHEKETTTGGHNRQNNKRKGQAETGDHATGAQQ
                     PASGDGDSILDTLPDEVIVDVIAALGDADVAALLFWSTTCRRYRELAMDPALWRRLYR
                     ARFGAPLHERFVDEGKDWLWLYRARACVVRGEATGSAVGTCTVAGRHGGVFSGDLLDG
                     KPHGYGLCLGNASSGNNPPASVGAIVARREGHWRNGLMHGYGMHVCADGVTYRGDWVN
                     DMYHGRGVRTHPDGRIVYRGEWEQNRACGYGVCDYGDGRRYEGQWAKGVPEGYGSLTY
                     GNVDDGDGDAGADLGHAIATYRGMFRAGAYNGYGVAVRRCGTRIEGQWSNGFAHGHCI
                     YEDARGWSLHGAFEHGVQEGYAVRVDPDGSVGRGQFHDGVPHGRCALFHPDGTTYEGL
                     FHNGVPQDDGEDMDAM"
     gene            complement(34734..35096)
                     /gene="pino_cds_19"
     CDS             complement(34734..35096)
                     /gene="pino_cds_19"
                     /codon_start=1
                     /product="Ser/thr kinase"
                     /translation="MTRCGTPWLDAPEVIRGARYSEKVDVYSFGIVMWEVVTRRRPFA
                     DRGFADVALAVLDGMRPTIPTACPPDLADLMAACWDADPDARPSMGEVVSRLDAIALA
                     MNHRDTPSLRPDIERGFF"
     gene            complement(35354..36436)
                     /gene="pino_cds_20"
     CDS             complement(35354..36436)
                     /gene="pino_cds_20"
                     /codon_start=1
                     /product="Ser/thr kinase"
                     /translation="MRDATLLCNDLLRNLAAVHNGVEVAIQGGDTAGAGCMCMAFADA
                     AHARAWCAGAQVALVDAPWPRALLDAHHGAAEVFVDADDGGDDDDNDDNDSTVAEDDR
                     QRPSGKDLPGQSMTRRRRLYAGLRVRMAIHMAHPRWHREPGVLPACSGADVQLCCRLA
                     ATAAGGRVLLTAEAAMLDTGARGDDNGNTGRDKASTPGQAYTVDRITYGHVNSSDDDN
                     NGDDQSRNGRESLYELRPHALRARRFEDIAKASWRAADPTSESVSAASPTDGAILQAG
                     GGLLASANACRWIHRLWRDRVARSTWAPARVAWSTAGGGRASTWPSSAFPTSASTNGA
                     SLSSAPRRQRWRSCVIPTSWPLSARA"
     gene            complement(36572..36982)
                     /gene="pino_cds_21"
     CDS             complement(36572..36982)
                     /gene="pino_cds_21"
                     /codon_start=1
                     /product="Hypothetical protein"
                     /translation="MPLASSSWGCSHGRALCRHVAGVDRRRRHPRQYPAAHALGGAVR
                     LCGPYHRVLARRSGYAPGLCRHPASPVKHHQARWLVVFIVIEQHEPSGADRRGLCLCV
                     VIVVVLLIHDGRHAVGLVALSDCGGGARLAAPPP"
     gene            complement(37066..40176)
                     /gene="pino_cds_22"
     CDS             complement(37066..40176)
                     /gene="pino_cds_22"
                     /codon_start=1
                     /product="Ser/thr kinase"
                     /translation="MAIWSGPFTQGVNDPCRQHARLLAMLVMAILVVSAAIDQASAAS
                     RTAIGAGGMGGTGLYEAWSDGYLYRQDLVSVDYDATAGTEAGMERYFAAGRSDFVGLD
                     HGLDDVTLASISDGTWQAARTAPDDDTDGNQAWVAGSGVIQVPVAALPWVFAYRVDAL
                     AAAGHTAPLVLSGPLVARMWLGEITAWDHPDLVALNPILGTASNLPPLVLVCEASAYG
                     STALLGATLDALYAPFAAWRASLPAPTAWYAVAPATTSTSNSTLAVATVIGGTTPLLA
                     NASATDGALVFAAYATVRRIDGAPLTAASMRNPANKTVTPTSDAVGAALDDFAEVVSA
                     SAPERMHEVLPVGGAGTASWPMTAFALAAVATNVAAADCTYVSYVLDFIGWALLNQQA
                     ADAVATDDAAVVAPPRFAKNAIDLMSTVRCNGAAAFTAALIIGSGSPTPVYSLWAYAY
                     DDDPTAAGATVHYTETRGNRAKAQILAGDVDFGPTNNDVDPDVHAAHPDLALVPVGAY
                     PVVFCYNVPGLMKSGAPSLVLSIDVAMRILLAEITRWDHPDLVALNPGMALPAADILF
                     VGKTGSSIYTGTISRAFALHSDAFAAAYGTGSNNVAWPVAATNRSVVSTLDEYVDTLK
                     TTPYAIAYTAHHVVLRQRNLREARFLSADGSTPLEPTRETTLAAVAEVAAASGGIGKI
                     DRLSFVVGASGPRAWPMANMALVLMHTATMPDPVRARQLVRWLYWTQTAITAVQISNV
                     TGVYGVADLPDVWSDVLGMLVNVTVDGAYVNPLRPCFGDGTLCSDAGTCDETAGQCRC
                     RADRTGDRCEFDVASAGSDDGGWSSGETAAVAASVSVAVVALLCIAGVLAVAAIVATR
                     RRGRGREDWEIDPDDIDIAETNVLGAGGYGVVYRTTWRGTEVAVKVISERVVSGAGGS
                     EARRAFADEVRVMCALRQPPPQRGLVHGGVPPSRPRCASSWSLWPSDRSTTSCTMNSC
                     PRCRTRSSSRWPTRRPRGCIFCTRRASSTATSSRSPSCWTPSGMSRYQTLASPVQGRP
                     GAQRRR"
     gene            complement(41054..42160)
                     /gene="pino_cds_23"
     CDS             complement(41054..42160)
                     /gene="pino_cds_23"
                     /codon_start=1
                     /product="Hypothetical protein"
                     /translation="MHCPRNCNPSLLALAVLALVAAASACASEGITAARLPGNPMVRP
                     DMMQPACDGAYDSNFPTIIRAPHWVRPRLGNFYMYFSDHHGMFINMAYADRIGGPWRV
                     HAPGTLTLEQVYVANNETFNLKNLSSSTEVASPEVYVDEANRRIGMYVHARLPYNGYT
                     SLTGIAFSPDGLHFDMRPGFFALPYVRRFTWSGDRHHVYLLDRRGNLLRSPDGYTNVE
                     TGNSVVGDAFTNASMVNGNGYTGLLRHLGVSVVGDTLYVFGTRVGDAPERIVWTSMDL
                     SCLRRNWTACTTAGLAQEGFRPEYDYEGANLPNIPSSKGSANGPVNQLRDPFVFADGD
                     DCYVFYAAAGETSIAAARVDDRFCFARRRPRSPQ"
     gene            complement(42873..43364)
                     /gene="pino_cds_24"
     CDS             complement(42873..43364)
                     /gene="pino_cds_24"
                     /codon_start=1
                     /product="Hypothetical protein"
                     /translation="MENSKCRQRPPRSVRLTDDMGLDEADDWHVLPAGDADVDAGEDV
                     ASGFSNEALFYIRAIPKRCKATGTNARTGEVQQLLTPEGARILCLMVGTKAALDFVAY
                     MDEKEALHEERVALMREMADNARVLSRRRKIPRDVYQSIRKTCQKALDAKRRRDNAQM
                     GLV"
     gene            43969..44523
                     /gene="pino_cds_25"
     CDS             43969..44523
                     /gene="pino_cds_25"
                     /codon_start=1
                     /product="Hypothetical protein"
                     /translation="MDPPPIDVLPDDILYLIVSNHVRDPKDLGACLLAWRRFHMLVPA
                     DLTIRRCRFATLLSLCAAGDLDGLQYAASRPEVFGPVAGFRWDACLYVAVVGDRVDIL
                     DHIKGRIVAMASSEKSTMPDGQEATLLDKLGCIHSVSQNPTAGAPTWPLMPAPWLALA
                     VAAARCGMDGSLTWLCAEGNRPSA"
     gene            44556..45815
                     /gene="pino_cds_26"
     CDS             44556..45815
                     /gene="pino_cds_26"
                     /codon_start=1
                     /product="Hypothetical protein"
                     /translation="MFGKTARQGAWLLWPRLRLDIGVALKSMWAAQNMTVDEMRAVAE
                     RISVASGLDIVGLFQRSIALGGGADDSRVPWAAASAPPDIGRCGEQKASDDAVAREAM
                     ADPSSASDDALEAIWRVALHGGLPGLRAQHGDDAVAAALKRCADYPAMFAKFAFQWLE
                     SMSSDCPSLDSLSINLPWPSARDDIVWLYRHGKSHEASSNATIFASIAPTLAIVMALA
                     GRRDLMAELGDDPQSADRVAPASEPASGDPLTEEESLYLPVAATAYARGDMDIARWAC
                     VRMGPDDPRWAWKAWREGHADVARFLYAHGCARAPLTTGKDDMLPSEEPSKSPLYASL
                     CSRDAEAVALLLDPTAADDSCRGAVDEAISAAIAMATDEALAEGNVRVVLWLHRRYED
                     LVDAVLAKARATRAPSLVPVCVRLEKI"
     gene            46169..46687
                     /gene="pino_cds_27"
     CDS             46169..46687
                     /gene="pino_cds_27"
                     /codon_start=1
                     /product="F-box domain"
                     /translation="MAEASSSHHACEPAIDILPDEVLHHIVAQYVGDPKDLGACLLAW
                     RRFHVLTSTDIVAHRCRFSTLLSLCTVGDLAGLRYAMAHPQVFGPVAGFRWDACLYVA
                     AIGDHIDVLDHLKACITNIAAALPPPPDLNPEPTADLHVNELRAIRSFMQNCTINAPT
                     WRPVRRRGWLSL"
     gene            46722..48008
                     /gene="pino_cds_28"
     CDS             46722..48008
                     /gene="pino_cds_28"
                     /codon_start=1
                     /product="Hypothetical protein"
                     /translation="MAVRRGQPSSACAHAAGRALCPRDGQKVDQTSRRHAALDTHALD
                     LGVFLANKWAARAMDDDAMRAVAERVSAASGFDITTLFQRSAERIHAGEEGPRAALEA
                     VRTQLAPPQETDPAQDELAQEAMADPSGASTDTIEAAWQIVRRGGLTALRERYGDETV
                     AIVLNRVPDYYLRVINMLGGMLTLCTDGPLSDMIAANLSWPSLYDDVMWLHMHAVTAA
                     MHNSAINSIIIPMLRVTMAFVGRHDLMAQLGDDTEDTDRSASQTKLTATSVGRMYPYA
                     AIAALGRGDLITARWACARMGAGDARAAWTAWRDGNADAARFLYAHGCDRAAKIDRPH
                     DACAKSPLYVSLCTRDTEAIRALLDRTAADDSYRDAVNQAIAVAVAQATGEALADGNL
                     RVVMWLHRRYKDIVDAVLADARAARVPMLIPVRADA"
     gene            48489..49259
                     /gene="pino_cds_29"
     CDS             48489..49259
                     /gene="pino_cds_29"
                     /codon_start=1
                     /product="Hypothetical protein"
                     /translation="MQQWTDRGAWPGEARGRKRTADVAMGPSMRDVAAGVCMALRRGE
                     PVDPQAAQFVVSAARAEGAVGSDMDVLVAACSAGLASMALTPMAARSGIAPVGWTGPV
                     AFGPPPSPTPPSPFGRQQQQQYSPTSTAMLGRKRRADEALGTAAAARSARDIVGDICR
                     RAYRGEPVSPDEVETLREIAREEGSLGANLIRDVPGLCRAALVLMGESPATLTALAGG
                     SMRGRRPTMPAAPPSSPVGPSHPPVVRLNLAPRSSRFL"
     gene            49957..50853
                     /gene="pino_cds_30"
     CDS             49957..50853
                     /gene="pino_cds_30"
                     /codon_start=1
                     /product="Hypothetical protein"
                     /translation="MDRRRRGSTVWALVVECMLAEVAALDRSGFGDRTQKRCWRMARA
                     LALLGSVCRVLYDCVGRLNVRFDMLLAVDAEEPTLPNHNTSTHLLWCALARKSTPPRQ
                     DALDTDGNLYHHMYRLVSCEMRSGHLCAPRAVGLLPRSPSALTDDSKDVDAVDRKATN
                     KDCVKDSDQDSDTDDDDKDQHGGDNDSTDSESDQDSDAESDDESQNRPYRSYVEMPDY
                     RNIWELLEWEDGDKDDSNKGRGGEEQDIDEDGDEDEDDEDLHEDEEGILCESGTHHGE
                     WTFNVFNHYRNGLILTGVKVEP"
     gene            complement(51504..53006)
                     /gene="pino_cds_31"
     CDS             complement(51504..53006)
                     /gene="pino_cds_31"
                     /codon_start=1
                     /product="Hypothetical protein"
                     /translation="MDVCGEWTLGAYSDPDGDPTSSSSSPPPPVVHLSDRCYLGSDTI
                     DRGNDQDAGQPGMDQLGHENADNDDGTDDATEFAPAARAHRRWWSQRHGLPVTGASDR
                     DTNVRFMLSAAVPMRLYVRAPSPAAMAAVASLFCRSPSRSSSPVSSPDTGDLGGTASS
                     DVDPMESSVCDFDTDESIVLDVCATAPGVDPNLLHAPSAMINNGDYRLGRSAYVAVQH
                     EALQVHRERGVRLLGEMATATGVAMDLSSVLDLYKRRLAVAHLKSLRQRVVALDALYD
                     HTTRGRCTPPEAVRTAASTTSGVEFAAAWYGQWASTAAPLQTPDDASDEYAQCSDRLR
                     RGGDVTPPRPVAWVPVAGNTGVPLAAGAPLFYVGTMPDPLACPVHRDVHGRLTCPPVG
                     CTHRDLLAGVMHALGDARALDALLTELVAADADTTEFALALRSIVNATLDHARHRWGG
                     KTMTAIRREPVDLFSEFPVDAFVLVRMGADGALLPHVIAVAPRLDIELCL"
     gene            53434..54402
                     /gene="pino_cds_32"
     CDS             join(53434..53698,53891..54402)
                     /gene="pino_cds_32"
                     /codon_start=1
                     /product="Hypothetical protein"
                     /translation="MSTLPQRRTNPFAAVLSRSMSARQPRRASRRSPPPPHNPRLPAA
                     NTDRHTRCHGALAQLRGHAGDDRSQRSLGAFPGHPCASCGHARSRDFSSATITTLCRA
                     GARPIASCRRRSDDLDRACRGSYRDACVGDVRCTVGCRPIANSSIYQGGVGTVAGEPG
                     CIVGGSSRCGSTTRIGGAARGHRCCRLPSCCAKVVAVLTDAAPTSVSAVAPAMTVPTG
                     PSPQHIHVHLSADTRDCKTSLRATHLRRKALGVAPKHRCK"
     gene            complement(55003..57257)
                     /gene="pino_cds_33"
     CDS             complement(join(55003..56377,56446..57257))
                     /gene="pino_cds_33"
                     /codon_start=1
                     /product="F-box domain"
                     /translation="MLDQGRTMLAATATIGPQCAAGPGPRSLSWPRTTPKRPQHETVD
                     GLLDDRHHHHQQQQSHRYNSAKRRRSSDEDSVKEAHPGLVDEAHDNAIDSGDTLPEEI
                     YLLIAGHCDLPDLCRLARASRRWHRVVSDPRLWKAAYARYLPACASPHRCRGVITDAI
                     VDAAFFTSGLAALGVPDTDLIEAPQESAPHVIPMDTDGEADDEAGNRRESAIVQEIHF
                     DAVPAEPPACIPPWAVAVGEALARCCARVARSSGQAYAHASVEARAAVAAAPPVDFDG
                     PHFVCGPSTRSGIAHRSARLFGHLRGIFEWPSRLGTAGTHRAVLFTDGRSYTNTTDEV
                     SMVLLLVDADGRAVWALARAPLTESLAIDGADWCVAGPFAPSSALSLPGAAETSMTMV
                     EEASTEDTSPRAADGAALGEGVQETATTTRSAAILVCRRTDGTLVCAYTSRAPTFLST
                     VPGLLRSIDGPCVMRSRGSDAIYRGLVHHGMRTGHGIARAANGDLVYTGQWDDDLPTG
                     EGTLYASDDRLVFRGVFADGMPDGGAGLLCVPPRQVSGRTCKVWARRWVRPTRLTQWT
                     APCGKGHVRLDDGTRLDCLWDRNGRPPVVVRVHVPAASRLALDGAPCVLDLTVRPPSL
                     YDEALVLLGDGPDSSLASDRQRRRRGAQGVEVPVDERRRRTAKARWTTRIPAPFVAAP
                     LSTWSAEFVPKPGDWMARTLAQPTLCFAVDLAPHRPGRLVVDLLDH"
     gene            57670..58365
                     /gene="pino_cds_34"
     CDS             57670..58365
                     /gene="pino_cds_34"
                     /codon_start=1
                     /product="Hypothetical protein"
                     /translation="MAQRRRQPTSMNTVSITMGPYAGVLPWARRPQVGDGDNGSDSVV
                     VLLGQERYEPGWRDGGRWSDFGGGVEPRVDHDEIAAAAREAYEETMGMLGSHDDIEAG
                     LRDAAAAGRLVEARSPKGAVVFLWEVPFDATLPTQFARVYAYAQEAAAAAAGPSSPRA
                     RYEGGGFAAPKGYYEKINVAWVPVPALIEAIEAALPVAEARAHGRVPPADPGLLRDDF
                     ARTVARFFDPSHR"
     gene            complement(58651..59229)
                     /gene="pino_cds_35"
     CDS             complement(58651..59229)
                     /gene="pino_cds_35"
                     /codon_start=1
                     /product="Hypothetical protein"
                     /translation="MGNTWPSFGQSPGDDEQPTKAAPFGWQGVDPAIERKAMEMGRPV
                     GGGVGLGMIDDLGLSPDDGWVVENRAHPIPTRAADPATQRMLHYMRAIPKRCHVVVVN
                     RRTGQEMPMLTPEGGRLLCLALDTEALRDFMAYMDEKAARRERAALLREMSANALALS
                     RRRQIPSDVYESIRNLCREGLDAKRQRDSAKS"
     gene            59914..61907
                     /gene="pino_cds_36"
     CDS             join(59914..61417,61519..61907)
                     /gene="pino_cds_36"
                     /codon_start=1
                     /product="Hypothetical protein"
                     /translation="MSETVGRRQRRARAESRSGCLRQLIVGELRSYSPAYESMAMLDT
                     VATALIAGATNSGRAYTGAKVLSLTALDHASYPAESGLCTMLCVVLWCGPPVVSWVAT
                     DLDLDAARAPGAHKIAEGALPESLALEFHRAGLLRAPSLCFEPSRPMLPPTRFVLAAI
                     RPALQACVDVAWDGSVSRRCPRTPGACVACDRVWRTALHLVEPPTSLSSSATRTATAS
                     ARPIVRALASVIAQAPTPSCLPRTSFAATSLASPTQALCKSPVVPPAPDAQQQRQGPP
                     LSDQRAKRQRRDPAECVLVATEHPLPTRPTQATAPASVAVVAAAPVVHPRCACATCGA
                     ASRFCVGKIVRVECTAGCRTPFHRTCWRAMAVVPDETRACETPDCWGLWARVTSARRA
                     SDGSESAPYIEWSRPQRRPAASVAAATTKDGAKSTTTRRRMGRAGASAMGPPAPTPPA
                     KGEDQTRTPYKQKAADTDHNDNNTPDGDQEDGNENTYVGRVRRRRPRQQLRACHASQA
                     PADRRLVRNAIAATTTTKPLDGSGRKRSRRSRTAKKEAVQTTAPASDDAVSQGDTSSR
                     GPRDVYGVWAAFFEWDHVPLSLLPEAPATPPSVGRMWTLPPLLRDDVPRASAVWACLC
                     TSVPLA"
     gene            62639..63250
                     /gene="pino_cds_37"
     CDS             62639..63250
                     /gene="pino_cds_37"
                     /codon_start=1
                     /product="Hypothetical protein"
                     /translation="MESTPPPRHAAVAGFLGPALLVVELQGVACANPTAVVGDSLANI
                     VDTASRSLAGAGDGGVHLLAAYAMDRRPCDSKATAGFDDRAGNAPMRPDDICTVLCVG
                     ALRSASTSHWVALDMNRTQESVGPHGMRARRGRKWFAIGAAQATLLGCFTCHASINSH
                     GVIDRKMEAADGCETCARTWRGFRLLFRGRALVSASTRHAWRP"
     gene            63352..64803
                     /gene="pino_cds_38"
     CDS             63352..64803
                     /gene="pino_cds_38"
                     /codon_start=1
                     /product="Hypothetical protein"
                     /translation="MAPTDPCLVRAERALATLGAQIEAALAVCLWADRFGASAALAIM
                     ADADSRAAMTLPEHPPRLRQPADVLESVVAVAVAAGLLVARANLAAATALVATTGSGD
                     GDDATTTPPVSVVPSTLTSSWISSVLSDRGEADMGDWASRASAIFVRVRQAAAVHVAR
                     ARTVVPPPLTVTDDTIAKTGPCNSWARRQKQQRQRQEDAPPVSTCFESASDALCLASV
                     MRCAGADCRAPGRRITSGCVISAHCTADCRATFHRTCWENAGTTLADGTPCPTPDCWG
                     EIARVTSARWRAADRPPRVLWQTLSSSPCASAETARVGRPHSLALDHCLAATHDHPSC
                     ADGGYTECANSHESGDRDHVHWGTRGADNDQDPNREDSAERERHGHANATSPLHGQGT
                     VAAAAPTGGTPYHKNDLSREVPPGRRKRPRNRTGRRQRCRLARQQHDRLLGLTGLADP
                     LAEGPKAEGAVCPRDARMEDDSLWPSFFVPDPA"
     gene            65484..70230
                     /gene="pino_cds_39"
     CDS             join(65484..67497,67753..67769,68083..70230)
                     /gene="pino_cds_39"
                     /codon_start=1
                     /product="Hypothetical protein"
                     /translation="MVADFLPPGIVVVELHGLTYADAVSAPGQFTGIVKDLTSMLDDD
                     YDGAPLLATCVMARRSRDDDDRGGGSRGPTDDDHCVVLCVAALPAGSNSIWTAFAVAA
                     RDTADRRFGVPHPDIRSVTAGMVDATLHGCFTRHSTIDSRGVVARNAHGARDCASCTH
                     TWSGHRALSRKSLRRGPLGQMYSLGFHQSLLDRVTLGDIVSYGAKSIVATGRSGKESQ
                     EDVSRAKRSLDRLLVCVEGVLAACACAERFGLTRALSAVTRPDRGVGRILADSLDERA
                     RTGLALEPITRALVAASVVAHRTKIDAVKIIVEDAGPDLGTAEGMESFVQEWCAPRLA
                     VRTNTDMLAFCERVAAFFMDLHSRPTMGEATHTRAVDATEPRPASLSCTRAPKQRRRR
                     QPQEQRPDAKPATSTDVVACAVPAAPRCGSADCRVSGRRIATGCVVTVACTVGCQVAF
                     HRACWKAAPIVCADHTPCPTPDCWGEIAQVTSVRRRASDDCEPHVLWRAHASRDPPPA
                     TPAPASPRLPMMPKAGAACGDSGHADDGATVHADGPVQDDPATHASGRYVVDAGEHRH
                     DTERASQDDDDAVSEPVVLAARSGTPYHKMTRRARCCRHDASARATAAAKDSVVAWPS
                     NRMIACWCWRAWSIRPSIIPKQVSPANLGARTRTTTPCGRPFSSPIPYHEKKKSSVAT
                     ATMETALARQESFASTHPDAIAASAVAPHLFIVEIQGIGCRTLSVKGLSGLFYRVAGA
                     MVGSASLVSMQTMGRHPWYCGATDNGVPCQCDDTCTVLCVGTMPHGGGGGVWAAVDMA
                     ASAPGKRKTRFSGGHRLMGILCVQPSMAGCFSRHACITEHGVVVRNEAAARDCPTCAH
                     TWNGFQVLLTRAPPRERANAARLAAITTQYLDARPPSTDVGVINVMCMCIQGMRVAAE
                     MVPASDARMTRVERAVAALGTQVDGLFAVCLWARRLDVRRAVRIVVDADMQVGVVLPD
                     RLLPLALTSLDSVVNMAVTAAVIVARANRAAADVICDTPTLDADIVAKSVSATAGMCA
                     GMMNALESCGLNEAAQLAARVFAQARKEAAEFPSSSTKQDAADPATTSLSTTETEGAP
                     KRRVRPVATDGDNDGVSQRQSQRDGRDDEAPTEALRCAGAHCRAPKRRIVSGCAISVQ
                     CTAGCQTTLHRACWEAVGIALVDAAPCPTPDCWGEIARVTSTRLRAVGRPPRVLWEAA
                     AHNGKLPKATTATVNGDSAAQVNLPTHREGPDGSQDNHDNTAGDTNEAQACFARDPCQ
                     DNLITVTHDGNDTDDGDHCCGADTGAPTPPPPTGGMVYQKASASRDTLPIRRKRPRNR
                     AGKRQRRRLIERQQHQALMALPDPAKPLIETLPSKVDATGPPAQTDYADDALWPSFFV
                     ATASDTAQNGPRVM"
     gene            complement(71288..72109)
                     /gene="pino_cds_40"
     CDS             complement(71288..72109)
                     /gene="pino_cds_40"
                     /codon_start=1
                     /product="F-box domain"
                     /translation="MDALPDEIALHIVACLPLASVGSIACVSWRFNRLAMDESVWRGL
                     YDAMCPPCTGPQLDRTCMAHKGRALDHRPWLDVQDSEEKDIHFVAQPYASHPLHPFIT
                     TRPQGRDGPFDVFANAPASTCPHHCPSVIRARSYRWACASQCAPPRPIDSTGTRVGCG
                     VHWYYKPYVYPEGTTSVYRGEWHAQSEVPDGLGMHRVCRPPTRLVCSMNVRETGTWRK
                     GDRAGFVRHWDGAYHPNYIEGRCFNGGTRFKGAIAEGKTLTISVVKYPTEWLNPH"
     gene            72596..73257
                     /gene="pino_cds_41"
     CDS             join(72596..72626,72725..73257)
                     /gene="pino_cds_41"
                     /codon_start=1
                     /product="Hypothetical protein"
                     /translation="MPRWSVAWEPDHKAIIIIRRRPTPTHADEHELDDVPLLPGTFVV
                     ELHGLSRAHALSLADLIPDLVDDAARNLLDGRSAHLLAAYIMACQPRVGGDHNDNGTD
                     PSSPDPACTVLCVAALRGRTLASWIAIYMKPVGEKIKMVTVKCTLSGCFARHARIESD
                     GAINRNEVDADDALWPPFFVDSVGKPL"
     gene            complement(75139..76332)
                     /gene="pino_cds_42"
     CDS             complement(75139..76332)
                     /gene="pino_cds_42"
                     /codon_start=1
                     /product="Moae family domain"
                     /translation="MASILVRPVVLSCLWNHATLAGQRSPACPFFGAVVLWKAIKKRN
                     TWQQVWVFMGKKDEGRGCTVGWPASPNCGAPGKQGRDRPQAFSSLDDMQDDGDFRRLP
                     PGLQSLVAEAARGLPDALQAMASLCETDRRFASVCRTALVHRARVDPSLLAFMDPAGA
                     GDRGRLVSPLDIVHAQQRRDAMRACVRYAIYSGVATKSAPASQSDNVHLLTFGEFTER
                     DRSRHFMPHVLAQTDNPPDHLVINIDNCDGTQVQFREAIPSDDMAAPYDAAAGALVNQ
                     ALTGIIAAALVDTDSASDVPILCACMDLFGAYPEAVNGLEVTRVPGMQEVSLDLAPIV
                     YPDGRAAFIRNYLNPMAQKRARAPLRVPLTRRGAGEPAERNMRDQMGPADLDGEYVPP
                     IFFIH"
     gene            76718..77749
                     /gene="pino_cds_43"
     CDS             76718..77749
                     /gene="pino_cds_43"
                     /codon_start=1
                     /product="Hypothetical protein"
                     /translation="MSTETNKRPAPAAKDHCGRDPARRFAETLAALTASSGGDVYDPY
                     ADRGWMGAVQRCDSGFGALARLRADRAPEVASNAPFTPAAPMAASLGPLKGSARESTD
                     AVFAWIRAAILDDHNLAWDAIDLEEADDEDEDSCFHVIEWLGDCLSLAVSAEERADRA
                     TDRTAALDPTGMCAKACRRVPNALVEAMWALEQLEEGAWRGNVRARPGLAEPHGEAMC
                     LRLATVAAAKRLAPDLDEIAHVFDRPALGNRVSHIGRVYRDAMRHRLDWAYDTYAALA
                     DKVDDMRARGDPAPPGHVPTPRSTAAWFASRSGGLLPAGLDRVIAPRPAKRVCLGSPD
                     ADAREITID"
     gene            complement(78138..80171)
                     /gene="pino_cds_44"
     CDS             complement(78138..80171)
                     /gene="pino_cds_44"
                     /codon_start=1
                     /product="Hypothetical protein"
                     /translation="MPSLVRARASPLPARGAPSVVVCDLTTSDGPLGSPEQYRDVLTT
                     FVTTGAAGRTTALRCAVSDPLGVRPAIAKPRPMPGRAVARRMATTSADDPHRQKQAQQ
                     QYQKRYSVPPALAALYLDGVRAVASWPHVTVSTHGIPSHSSDPCDCGPMCQTSAGVID
                     CDTLDATDVEGSLRLRLASIEETFEYQAARRLITALDRMVAGSVVHAIALHRPLWSRP
                     ACLTCIAASAPPRPSPVRAVQRYAESATSLLDATDVATSLSYFDTATSMATKRTDATP
                     FATLDGVCVYAATRALDGSVMWTARRRSDVHQDLGSTVSIVSPLSTASAAEHGPLSDH
                     ALQSTLALSPMRSPLDGPSVGCARCAVGKRVRGVVPTAPDSGAVVTVDVAEVIGAVAA
                     NLWEGVAGTVISINGGPDHRYRTLWSGVPGERPTTGLLDPAAVPATTWLATETRALVV
                     GAARRRPTSGCDALYTCARIAYATARLVDTTARQRATHTGATSDPRRPRPAAPLVYGP
                     SMDSPLAALAWMRGAASLFGAAVDADSVLSASVLSYRMHVLARDLERTLLTPLEPSRL
                     PTRCAIWPTNSRPRCNPPATTTKPITAPVAVTLMILMSTSKAGSPPSHPDPSVHKNKP
                     KYQRSTNFYQKIGKKGPKNSLANKKIETDTRCATGMHKSTAAPGVLVGCNNFS"
     gene            81678..82076
                     /gene="pino_cds_45"
     CDS             81678..82076
                     /gene="pino_cds_45"
                     /codon_start=1
                     /product="Hypothetical protein"
                     /translation="MEDKHRRLPPLEDGERPGRRKILCEIVDFDSGDKWTDFAIETSY
                     MRKRIADDAGMYRWSDKMYEVREAVACTCPERFRRRQTWEDDAGQVCREKWHKCEDGW
                     DLRGRRFLMEWDGFNWERVRIKKSKRCKLG"
     gene            complement(82096..82371)
                     /gene="pino_cds_46"
     CDS             complement(82096..82371)
                     /gene="pino_cds_46"
                     /codon_start=1
                     /product="F-box domain"
                     /translation="MDLPDEILVRVMKHLGDPRHLRSVALVCKRWCAVSSDREVQRLW
                     WYNHRPRNLGPRPRPRGLHCVGCTLRACLYEYKIDITTPDDGDTRCH"
     gene            82843..83916
                     /gene="pino_cds_47"
     CDS             82843..83916
                     /gene="pino_cds_47"
                     /codon_start=1
                     /product="Hypothetical protein"
                     /translation="MGNMRVYGAILALVAILATIIVIPLVCDRKATNQLVDWTQDALH
                     KHPLDTTNLTSPYMAHYLAETDPQYLVDLYDNDPDAIALLRPTTHEWITARRGRLVRT
                     RIPLIDYARLAAAFGQTDPMLLFLVGATCTARRYVEWRAAEERERRMTLTWKKISTDT
                     PPETDDTEALAVRVAAWYRAAHDTGRPEIPSGGTFVGVLDVDAKVGSAKRGTYMRLRE
                     SHNRTLADGADLRLGPLLRLGEDLTERVVSVSGVDAHWADYTLYADENVGLFKEIEAG
                     LVRKGGAKHVLGLVHRAATASGRTIGVCASQIVTSSPHVPSLDDLWTGPLYLWPSPLL
                     LEPRGRFESAPMADAAALVGLSL"
     gene            complement(84603..85526)
                     /gene="pino_cds_48"
     CDS             complement(84603..85526)
                     /gene="pino_cds_48"
                     /codon_start=1
                     /product="Hypothetical protein"
                     /translation="MALVTHVLGAVAPAYGATAFGAAVPVYGASAAYGVAYGATYAAA
                     APTVFAVGTPSVAACCGVGVSGFGYGFRGGCGCGIHGGCGCSGGGVPYSVCSVCCRTV
                     CICRQRHGGRWCSVCRQTECICGPSICSLCGVYPCACGRGRRHHHHGRHRRHHRDGRW
                     CEICRRERCICKGGVGDPLCTACGWNPCRCAVSSSITSACVPSSISSLSSCFDTTSFD
                     CSLPFDSSSSSSCSSSSSSSSSSCSSSSSSGTECQVVKVKRRTRRKGDHCASSEDDTD
                     EVVKVVRRHVARGSRPHRRRRSSSHHKKHHH"
     gene            complement(85892..86098)
                     /gene="pino_cds_49"
     CDS             complement(85892..86098)
                     /gene="pino_cds_49"
                     /codon_start=1
                     /product="Hypothetical protein"
                     /translation="MPQLCPESPRSPAWIYGVVILGFVVAVVLYGGLMLAQKRVFPSE
                     ARLYGTPVPPHIVRGSSSDLAWDT"
     gene            86672..87658
                     /gene="pino_cds_50"
     CDS             86672..87658
                     /gene="pino_cds_50"
                     /codon_start=1
                     /product="Hypothetical protein"
                     /translation="MHPPHRASDDAIVQQTAQQKKAMDSVLTVQRRRRKAPASDTVPA
                     LVEMLQKRRWHHALCMGPMPVAAAVAMWQSGADGVSGEAPVRVALDAGHPIIALAMLE
                     ATTPFALAGTADDAGSDAAADADRICRRLASHMALALRPRLVWRMIATWEVGGRRFRC
                     KHNKSSAPTDRVPASTTPTTPNLVRAVHPTRDAREMRRAWRFLLRFRLVAFLGALAKA
                     ATEAELARFWETSAPQTRVHILEIVLYHAERTLRLAERPHCQRVWAEAAASGVITASR
                     PTKGRHRKTDKRPAAANAGLLPAMRGAVASLRNVALWAARAQDAAAQQPTSG"
     gene            complement(87849..88856)
                     /gene="pino_cds_51"
     CDS             complement(87849..88856)
                     /gene="pino_cds_51"
                     /codon_start=1
                     /product="Btb/poz domain"
                     /translation="MYTSHVDAVRCLRKMRHRLCDCVIELGPRGADARDDAVETITAH
                     RTVLARWPYFRSLFARADPVRIDVGTGDAKGVCRVVYSVVIPFAASSVRALVDMAYDN
                     ARVDLLDDSKACDPVDVIKCAIYLGVGTRRVHALVANVIETLLAHLPTSVPDGGARTE
                     SADVGAFVLHMLAADLEESTKRRLVARLYYLMSDADRATAAETYRDMLLPPLLYGMET
                     VPPSGLRVCCDRIVAPCPAPRVIGSASAVVMVDFRQAVVNCTDGIVVTLRIDSAAAAF
                     WHCRMRFLHPIEKGDHYTSMTVHTGTPSTWTFAEDLTSDVLGVYRSSLTVCEVDMWPA
                     L"
     gene            complement(89600..90484)
                     /gene="pino_cds_52"
     CDS             complement(89600..90484)
                     /gene="pino_cds_52"
                     /codon_start=1
                     /product="Fascin-like domain"
                     /translation="MIKVTAHAPLAATAAVYLLAAACLLAALGADRADAVPVCTYRVC
                     WCNNKECTVFSSCDSYTQQTGTCGAANSVCNCGANTIVQYANGGCTGTATTYTAGSCY
                     AKTKCYYIDSCAEPSATPTPTPTTTPSPAIATATPSPTMVTASPSPSMVTASPTRSRS
                     PTPSVTTLPAVRLVGMRSSALNYALSVVDQATGQVATNQFSFVESARWTLTRLANGKY
                     TIRSPYGRYLSAQPDGSIVADRLVADLWEQFTLEGTHNLYYDIKTYHGAYMISDTSLS
                     VFAVTYESSLYWEITTIV"
     gene            complement(91025..91306)
                     /gene="pino_cds_53"
     CDS             complement(91025..91306)
                     /gene="pino_cds_53"
                     /codon_start=1
                     /product="Hypothetical protein"
                     /translation="MADDSNSGNGGTDRARPDWRPGPSWASRISWIYCRHQSVFWLAL
                     ALLVLFLAIATWMSVRYRCWQWRVDPVASAVMERRKRRLPTPYSYFATV"
     gene            complement(91662..92169)
                     /gene="pino_cds_54"
     CDS             complement(join(91662..91886,92053..92169))
                     /gene="pino_cds_54"
                     /codon_start=1
                     /product="Hypothetical protein"
                     /translation="MRRAETKATSTCADASSVSSAAPAYKLVCKDYYQLSASWAPKQE
                     ALRLKCEAKDAKRCGERRSFYETRRPSAERQAIIARYLAKRGYRCRYEQQVACDARTC
                     KRLHDGTAVYW"
     gene            92560..93153
                     /gene="pino_cds_55"
     CDS             92560..93153
                     /gene="pino_cds_55"
                     /codon_start=1
                     /product="Hypothetical protein"
                     /translation="MDMDDGRDMRGGRTGVPPPPKRARPMVSGGDDHMSIDACLGPST
                     DDGWQVYTRPEDWMPKRLMPHYDADLDEHHHEVDVTAEHALWRAYVRSGSRIPSADLD
                     VVFDDVVAFAGDFLDDDAYYDMEPLEPGLDRRRWVVAQPPAVQGDRARRSRLATVIGE
                     AMDREDSDLDGSASDGDDNDMGQGSAGSDDAASSCSS"
     gene            93696..95239
                     /gene="pino_cds_56"
     CDS             join(93696..93902,93980..95239)
                     /gene="pino_cds_56"
                     /codon_start=1
                     /product="Hypothetical protein"
                     /translation="MAALVDLAPELIQHITDSIARPRDLVAWSMATGVDVGPAIRRAA
                     VIPDDDMMVIQLLERGAPLDVVRASYVWIEAADRKPVPKRFDPQSATIMEHQWHHIRL
                     ALKVACRQAHDNVALWLLTRLPCAHAIGSAEVVEAGLLEAVCAGHLHVVEAIHARRIA
                     VVGMCGCSTRVGTFALVTDQAPIVAWLHARCVDTPSKHLGRGHREVDSGSLIDAIARG
                     WVRVAQWLLEMESSPCARTVPFQAVMQAAAQGHLPTIALAHDKGLHPCTVEVLVGLVK
                     GKPQSAVDTLRWAVGEPTADVNVPVPVGAVRPIPAWGDPTIAYAALGAASDDAFTWLL
                     NRPDARHLFTVGAVRWALTLHQGYARALCVCTTGIVSFGDCDALATVVRYLGVAQVIE
                     AIEAGAPYTPSAMEAALLRKDPVLLETLCQRYGTEDVPAAVRKMAGTTLDRPVIEWLR
                     DSVPSACIADLRAMLLVGPHYDAVPQEPCPCPVCSRRL"
     gene            complement(95698..96000)
                     /gene="pino_cds_57"
     CDS             complement(95698..96000)
                     /gene="pino_cds_57"
                     /codon_start=1
                     /product="Hypothetical protein"
                     /translation="MDLVTGACRRAAGVLWYALTATLYAPVRWAVGGASVTPIETDWK
                     VMLPIASPDRADDGHEFRLWWDALPETSVHAILDGDNDGDDTKADNVLAGRLHDDD"
     gene            complement(96587..97303)
                     /gene="pino_cds_58"
     CDS             complement(96587..97303)
                     /gene="pino_cds_58"
                     /codon_start=1
                     /product="Hypothetical protein"
                     /translation="MEDVVRHNGHYLVTIHRLGLWLNRQMCADRRAPVTAGTGGAWLP
                     AAALAYAAGRERSLALLSIACNRVHTIAAATTPATTIATTFWGPAGRRVSLGDSDTQS
                     ATSHAERLAVVIAACLAGLEDRRFHVEKGRAADMEAERDADEAFLHGMEPVVDFLTPR
                     SQDRLGAATPVVDIEADDMLQMLRQLGAAKQTGRGAHAATLCLTARFLVAPPGRPLPA
                     DSMRACIANLLVRAASSNQH"
     gene            complement(97333..100232)
                     /gene="pino_cds_59"
     CDS             complement(join(97333..99237,99459..100232))
                     /gene="pino_cds_59"
                     /codon_start=1
                     /product="Dihydrofolate reductase domain"
                     /translation="MESAQTAASDVGDGDTRKGLPAVHIVACTDSNDAITVDGVLPWA
                     FRAADQRDAIDAIVKDAPIVIGARSTSVFGGAPPGARVIILSRSGTLPVGAWARAHVV
                     QSPEAALAMCADEPVLYVLGGASTFAAFVPYAAVVHRIVVAGHTVRPWPQEAAIVHFP
                     WLGRAGGRTTVCGPLVAGRGGCSHQVRSDTLAPVDRAPPPPPPADPLVDDPDLEQAKM
                     LSFYEHAMRSATAAPPPRLSPWSANVCGSATTIADGVRNKKTAARVHGRLLGRCNVAL
                     AAAVIAWLDRRDLLSLWYASPRTRTVLVDVLTTGPTSPCHALVHIDDVRRLAECATAA
                     SVMRICPEDESPWKRAGAVWLWLDVLPRLQGKCASVEQMLAHPSASASGTLVDYVPGT
                     LERVIIWAAATGCGAVVNVCLALGDRLSDACAAYSRSGGFVGRVAGAGPVSEWLAGAM
                     GAEKKGAAADLATTDAWLSAIGLATAAGRLGSDLLLTLACQVASINLAAGRRFADRGQ
                     RVPDPTGSSKRASLARVRLILALVCSLGQARHPTDPPRTADGQDGGDDARAARLLDRV
                     RIDGLVDDVVRTACGAQADLWLGSALGEMCRRLRDIAVHGSSLKGRAAGALLAHVFVV
                     ASPIDHASGWRQRSWTMRAWTTVVPALAVAGAANFWAGLHVQRDPTSPLPVRLAKTKD
                     AVLPVDDTRARVDHVSASLDDSAVPTTAGDRNHRDDNVAATVALADDVDYQETTTSEG
                     GLTLLGLFEHEIDLCVEVAARLPPWDLVSLATASRGALKSVWAAVCRASLDASDNRLA
                     GVTCGASVYIGSGSSVSLMPAPDAGFAYALGALMLTCHRMPRMEMMADDVKALASLTL
                     DADDASERLDALERDPNLPAMLADTVAYAARLAVAP"
     gene            100919..102217
                     /gene="pino_cds_60"
     CDS             join(100919..101828,101955..102217)
                     /gene="pino_cds_60"
                     /codon_start=1
                     /product="Hypothetical protein"
                     /translation="MDRTAHASGHTPDNRPGFDAAGWLAAHRQALARGDKDRARGLRI
                     DVAIDTIDCATRGAYVLRDGTRVDLATEREVRDQVCATALYRPKDVRRAAATATLYNV
                     SATCEVVRGDCLKAAIALKTERGLNPAVLNMASGRRPGGGYKTGAAAQEENIFRRSNY
                     FLSLEDPRRIDRQRQWRYPLHGLCGIYSPSVSVFRGPEDEGYPFLDQPVRLDFVAVPA
                     IVRPQVHVLRDGAQRLGQVDADLMRQKIALMLDIALAHKHDSVVLGAFGCGAFGNPPD
                     HVALLFREVLSTDAYRCHFRHICFAIFDDHNARKAHNPRGNVAPFCDVFATPLDADRS
                     TEADPVIVVDPCDGRDDVDVGPRHNRGARPNQKSTKGKGRRNGRVRPDRSDDGAAG"
     gene            103021..104691
                     /gene="pino_cds_61"
     CDS             103021..104691
                     /gene="pino_cds_61"
                     /codon_start=1
                     /product="Adenylosuccinate synthetase"
                     /translation="MAAATRAATDSLVRITAPCDGAILPVTAVVGTQWGDEGKGKAID
                     YLCAAGDYTHVARCAGGPNAAHTIVVADPAGGAAKRCMVRLVPSGALHPDIVCVIGHG
                     VVVDVGMLVDEMTALGRDHGMRWRQRMHVSDRAHVLLDLHKALDAHGEREAASRGAAV
                     GTTRRGVGPCYADKVARRGVRVGDLAHRDRFVRRVRGLFAYHASAHPAAVVEVLATLG
                     LVSSATATNGINDDDVGTTTLSALTDTVLDAAAEAYAVRYVDGYYDTTLRGLVCDTIN
                     LVAEAICGGGRVLVECSQATMLDIDHGSYPHVTSSSTTAAGAAAGLGVAPRLVGAVGV
                     AKAYVTRVISHPDARAGCPFPTRLPDLHVASDSGIAHLTAQAAAAGTLSSADLPAEVV
                     GVLGVPPGVFWDSADLASARAMSVIGREMDGSGRPRAVGWFDAVIVRRACLVSGFDTL
                     VLNKIDVLSGLAVIKIAVAYRMADGAVVDSLPEDLADVVEPVYRHLTGWHEDVGACRT
                     FDDLPVAARVFVRAIEETIGCRVGWVGVGPARHQMLVVPPTLMTSTTA"
     gene            complement(105002..105903)
                     /gene="pino_cds_62"
     CDS             complement(join(105002..105232,105295..105578,
                     105654..105903))
                     /gene="pino_cds_62"
                     /codon_start=1
                     /product="Hypothetical protein"
                     /translation="MRFKGAVFVVKRPPSRMLTKIGLLALAFVLVVPAVFTYRAHVES
                     APTTIEQFEQTCFDSVWKVVLLFLVFLGLEQGVRVAVRQDVFCEGASRAWGDRAFRRG
                     KRIEPPRKSSQTIDCLCGQGRQQTVIDGVAYAFDHPDEPCEKFYFHIVQCAMCNSIDF
                     ALQQNNSDADTTDSNGNMVRDSKPSESLPLLVPSQRERCPKTRHQEPHEVLVSVKMRG
                     WTRVCYVPFPTKSTFKILCCSACGDHHGKKERRKRF"
     gene            complement(106139..108273)
                     /gene="pino_cds_63"
     CDS             complement(join(106139..106613,106697..108273))
                     /gene="pino_cds_63"
                     /codon_start=1
                     /product="Hypothetical protein"
                     /translation="MGMETKGTSMEKSVETGVSRPSRKRTVAHTVDEFRTILRDLSAK
                     PSKKPRHARHHHVPAYATPCDDTDVFDMLPNEMVREVLMRVDCWRDVVAFQATARRFA
                     SVLAPCDTWTRKYAPTTPYDLTMSARTRDTGEPNLVADDKVALCDDEPLEAFVAVHAR
                     WGMAAKGFDVAGLCRLAAAGRTDALLWLDARVYQMYGDVGIAPASDDYLGASFVEPAV
                     LRCRGRYARAAAAAGQSDTLAAVLAAHVSLAAHGEKIMQSALESNCAATVDVVHAFAR
                     SHDGQYRAHKWCHNGFGPVPPSSPLASSTAPLSVLTHLATRGCPMVPKPTIFAMAMTV
                     SKGLLRVAAWLDGLPEVQARPHELRRCSRRDVDRAAGDGHYAVVQWAHERGIRRCALS
                     TLLSGIRSGHAQGRVDFVRWALAEDPLWPHGIDTTEGQSGGAGDVAAAPAAGPWTGPP
                     STGPRRSRPARVPEWRDGLLAIEAAKAGALDVVQWLYETHPEMVSIEAARAAASACNA
                     DIAIYLHEVGVAPLTAYPQVGRGGCPYDPRVLANVVRHQCAPALRVIVKHYCVDPTNS
                     ANTVPCNGSFSLLSSSPTSTSTSSASSTSSLPPPPAHDGQASPRLPTPADIMRAAVAA
                     DRLDMIRWVRDNVKGARLCVAAEAMRAAGRKPNRIAALGRCRCAQCKAPSAATAKKKA
                     L"
     gene            108685..110592
                     /gene="pino_cds_64"
     CDS             108685..110592
                     /gene="pino_cds_64"
                     /codon_start=1
                     /product="Hypothetical protein"
                     /translation="MAAREPRMMEASAFDMLPVEIVALIVTESLPARWRFCARPVCRL
                     WKSILDAAGDENKNRPRNEYHDLTLRRCIRDVPWKSLKRARAAHGCLHTVACRWRRGV
                     IVLASTVVEWARTRPDLWDRRPAALAAWCMDWQRAPRGDVAKTLVASGREPLVRYAVG
                     PLFLAHGHLYNDPQPSDRGRCGGRSQAQDAAEIISVAAQAGPATTTVVTETLSRINWH
                     EIRDLAWFTTGDCPESFELLLRAWAARVRDNEPLCELWASVAWTGAARIFARLLEIVA
                     TAGDEDSRAPNDGHLDGGGSRARRRRRHPKKAMPLAVRLAATWEQKAKYCLSTAACRQ
                     HDRTPILAIARSRLTDDLTRRVIDKAWRRGCVANIRWCKENLGLPPMTLHLLHGAINQ
                     SVDFFEWLFDPRGGGHVPADDAEITNLFHTLASTNSACALWVAERWPLQSAAAGLTAL
                     TIMVDRVFLTGRHTRSTGCKGRDPYGGLLERLVHVLDHCAPHVPSGDDPVGGCDLWAS
                     LLALGRVKRRWPWDDWHVILCYMWARATGNDDDAIDMLGGHGPCPTPQALWARWCRVG
                     PVSLGDLGLVDADVSIITPSESNESPLIVADRLRHRGTILSRSDVLANSRASALALAA
                     WLRSKGLLADH"
     gene            complement(110753..112905)
                     /gene="pino_cds_65"
     CDS             complement(join(110753..111231,111633..112905))
                     /gene="pino_cds_65"
                     /codon_start=1
                     /product="F-box domain"
                     /translation="MDADNDPADLGILAASPAGPATSLTASPTTTSTPPTKAATAVVP
                     TAEVTRMPLADFRVALPPELLCAILEHMDPLWLPVAARVSTTWYDCAVAVGGAHATMI
                     TTRLVDEAILAGATDVALWCLEKLACPWTERRALLATITDQGLTERMGGTGVRSPLTA
                     IAALVHSRTDTALYASEIAHVDESADPVTSHSLAKRDNNVRGRRHRDDAHNDCSANDG
                     NEDVHAPLAARLHRLERCGYRWDDATLACAAVTANTVDFEALAVAHPVGLALAWVVAS
                     ALGRIDLLDLLGRRWMHAAPAGACLHVAHPAAHDWMARFTPWVHDAEPVRTSGWRSWA
                     DPPEDALAVWIAQRGDAFVLDPHGHLAAALLGVDYDANDDDDDRQIPRCLHIGRMVIS
                     GRPSYERGPYLSESARMARETMLKVDDILRTTLATKTGNTQAKASFDVRANIRIYHEH
                     ARGKTALQEASKKDARRLKEDLYRSQFGADIPADADCKLVWFAIDGGRMPLVVTPTAP
                     TLCEIEQKIIPNAYNWTDTDGGEFPLARAKVSLRAGRSPLKERPSDAAFYASIADGDT
                     LSWRIGRMGCCGCRCAR"
     gene            113601..114299
                     /gene="pino_cds_66"
     CDS             113601..114299
                     /gene="pino_cds_66"
                     /codon_start=1
                     /product="C1q domain"
                     /translation="MSSSTVCHCADGRAPCAAFDPRQTSACLTVRVPGRTGPRGTSGA
                     AGAPGPPGALGIVGPVGPPGPAGPMGVPGPEGPPGPQATPTVVAFRARGLPQVMPLDV
                     TITVIFGIEEYDLENAGAANNYDPVTSTFTAPLDGIYQFDASVSSTHLAGTLNIRVAL
                     VSDSGAPPIERWFTLPSSIDTADNQGASLSGGFALAAGQTVHMEAMATGVSVSPFITG
                     LTQSTFTGHLVAET"
     gene            114427..115188
                     /gene="pino_cds_67"
     CDS             114427..115188
                     /gene="pino_cds_67"
                     /codon_start=1
                     /product="C1q domain"
                     /translation="MDDNPNPRSANPSWHRTMCPAPNSSCAATQCPGRDPHCFCCCRP
                     RCTHVSVMGTRGPAGPPGLVGPPGPPGSPGQSGPPGPPGIAGPAGQQGGLGPEGPPGP
                     PASTVAFRADGVAALTVAAVVTVQVPYENQIYDIQNGVAADNYDPATSTFTAPLTGVY
                     RFAAGVNGIRATGEPPVVVSLQASTGALIQRRFTAFDVTDADDNFGATVAGDFLLAAG
                     DTVVVTINPGAGGSFTVSAATEIGRTFSGSLVAQL"
     gene            complement(115451..116536)
                     /gene="pino_cds_68"
     CDS             complement(115451..116536)
                     /gene="pino_cds_68"
                     /codon_start=1
                     /product="5' -nucleotidase"
                     /translation="MDLDEDVQFARHARDRIDIVLGGHEHHVVVDRAEGRAPVVKCGS
                     DADHLGRIVLRVGDEKSGTRVESIDVLPNVAVGDNDVQATSEERSTRTRVAALLQHLT
                     AQSPKVDIDPSDLVLSDMTALCMFSHATSSTAARSGPCPLADLFCDLLAEADGSDHVL
                     ALIQGGALRGARDYPAGHVFTARDMRVEMPTMSRCAVRLVSGDKLAAAFEHAVAGMSA
                     DMGDRDDVNDNTFAGATVQKGPTHSRALLHVSAPWRVVYDSRRPVGQRVLSITCDGAP
                     LAADTLYRVIMQQFVARGGDGFHMLVGAPTAPSPIEAVFMRRVVADRLRLAADQDPQG
                     LLPVAEFHTPRVSVVSTRQVDDDGGPL"
     gene            complement(116643..117230)
                     /gene="pino_cds_69"
     CDS             complement(116643..117230)
                     /gene="pino_cds_69"
                     /codon_start=1
                     /product="5' -nucleotidase"
                     /translation="MARGERVVILTFNDIYVLEQPAGSTNGGFVGLCAMIARERQAAL
                     DDPDDPAGAVIVCCCGDFMTAAARLSGRGPQDCGRHMVPLLAAAGVTHVVPGNHEFDR
                     GTHGCTLRSAESPFCWLCTNIDSACPENPPPSVAPGPMARLPGVCASRDVAISALDHI
                     TDRQPPLGPGCAPTWSSLAAATASDSSGSARRTHP"
     gene            118222..119397
                     /gene="pino_cds_70"
     CDS             118222..119397
                     /gene="pino_cds_70"
                     /codon_start=1
                     /product="Btb/poz domain"
                     /translation="MRDSSLHHHDAPPDDAPEPPAAAATGDHDQNREPPTLHDDDIVE
                     LDVSGERMSVLRSTLCAGPPTSVLRRMFDPASPAGWAPPMRDGVHFIDHEPRCFRIVL
                     DALRYGTGAVRFVEGYDLACVRGMADYLGLYDLAAECANTLLRNEDRADPDGHTSASY
                     IFEDSLGERDCHEGTGAVGDQVRVPCDWPASRALDTIADVIGMRRRDLVFYAAREVDQ
                     PTTHRIVIGAIVDPAPTTRVFQCPWARHDGHTLFVARRPAGAHIALCKVYEMTRDSLR
                     LRLSRPLVFSLPEPYAPARDVVQAACARTGIALSDVVAAYVEPHDADPRRLPYPPIAG
                     GGGGPGDINQEATRKRFHPGDIVRLIVAGPDAPINDDDAPRRLTAKAAKRWAMTWYE"
     gene            120275..121888
                     /gene="pino_cds_71"
     CDS             120275..121888
                     /gene="pino_cds_71"
                     /codon_start=1
                     /product="F-box domain"
                     /translation="MEDCVDDLAKGQTLAEPPPRPPAKRRSETADTAKRSRSTKKRRT
                     VARTETPTGASDIDTVDLFSLPVEIVLCVLSHCAAVDVARVGLTCRWAAAVVADPVSM
                     AALYRRAVGPLCTNPLCMGRFGDVVDDDYFAAADPAPLNVVRLADCATVECEIDDIDD
                     LDDNDHENDNGNNDDNCDGGGGHDNTNRDSVYGQHDDTGLFGWSGDDQDKDCAPERDL
                     GAADSDNDQSERTDNDKSNNNNNDDDNSDENEGDQGGFDSLCGHRESDIADATAEDCL
                     RENAGGGRLGGTRVHGGWRSSKPSCRVRHMACRPVWCPPSASAGAIRARRAGAAWAPP
                     RSASPSTSLCGVAATCRRRSPPPSDHCARWLWPTRSPTPCRRRFRMAPPLPYVPAVAK
                     SATAHARPRRSDDRVPAMRRRWLGLLARWPSRRAGTCCAYLVPDRRGRAHRGALGAGL
                     GGRRLAQRRSLSPDGWCSVCGARVARRRCACSRRARVSRPRGRPGHGDDLDDRRRRHG
                     RRPGGTGRARDAVVHHHGRRRTRPHHAVR"
     gene            121898..122794
                     /gene="pino_cds_72"
     CDS             121898..122794
                     /gene="pino_cds_72"
                     /codon_start=1
                     /product="Hypothetical protein"
                     /translation="MGDRHGPRRHRRRDVCLWYPAVPARTHATVRVARTVPVAGTDAA
                     VYRGIVDDSGERPNGHGAVYALDVGTDGARETVVYEGGWLDGVPHGWGRLFDPTTPAE
                     AHRDARPLFVGSFCRGAPAESGTLSPVTGCTVEAAGWWAGSDDDNDNDGRQNGDVVSI
                     PAPRGPGVVHLPCGAQLTCDWRSVGSAPVVHAVRHRDAGLGRAVDGGDCALVVEALDA
                     PHDVDIWTDAIEGELLRRVDAVRQMGHSVARPGERASARWVARVLTTSSLRFRVRCGD
                     SPDAVIVVDLASMLLPWPAGAT"
     gene            123441..123650
                     /gene="pino_cds_73"
     CDS             123441..123650
                     /gene="pino_cds_73"
                     /codon_start=1
                     /product="Hypothetical protein"
                     /translation="MLDRLRALKAANAVHVRLTADQLEEFQGYYFRCEIATLIYAPYG
                     DQGEWQAVIDRPSYVRLFDPPHRAE"
     gene            124249..125636
                     /gene="pino_cds_74"
     CDS             join(124249..124661,124730..125636)
                     /gene="pino_cds_74"
                     /codon_start=1
                     /product="Hypothetical protein"
                     /translation="MKRHDTERRDNARRHRATQRRGTASHDNRAVLPLASATNDLRAL
                     CGRLNDGTASADDIFAAMRLSDGARVGAASIIVVGDGNHTIGVEQKCTGLIAAYEALW
                     SSLDDHAIQALVRNGPSGWPPTFVDVERAYIALAPRLGHALSDAARSAAMSCVRQRAC
                     PFAEAWAPVLDWAGAGHLRDCAPHARYAIAIVRRLDGDTIVLLRDVGGDRPAVALLHV
                     ETRDVLPVDDARLLVLAPVPPALVIYGDLLGLFLAAMAHRPVGVDYGTPTERLLTPTP
                     AGLAQIRAALSPRRALRASAAASLGRLFPATVALDAYAPVRSVDLSECQLVLALRLFA
                     GQVEARRAVVPQAATAAPPPSDTAAAVYSGPLHGDMASTDAPDAAAVATWDRVCAASA
                     LPDGTLAESHRLVDVSRLWGHEPDAAEIARPELLCASLALARNLLAQ"
     gene            complement(126006..126878)
                     /gene="pino_cds_75"
     CDS             complement(126006..126878)
                     /gene="pino_cds_75"
                     /codon_start=1
                     /product="Hypothetical protein"
                     /translation="MRTCKPPRAVLVGYSADEALGHLRRDPTLPVRVRWLLDASTAAT
                     LHATLTSDQGAEVTCMTFVDSFHGSDDLPALGWWLRRRDMDGSADAISWCMRVARRVK
                     SHPDNDNNNNNRDESKSDGITLKEDNRDHTDPVAREIDDDGATRHRRGQGRPRVYVHN
                     DIDDDGHLVAMVAPESGARDLSALCLGLYARVSVGRTTYHLAAGPHAAPALCVVVDRV
                     ALGGSGAGSVALQGTAVVRDPAAAQRLINALTAAGVDTSRAPPPSKVAAYLVRRRPAL
                     YARLVQRGALDPSS"
     gene            127769..128722
                     /gene="pino_cds_76"
     CDS             127769..128722
                     /gene="pino_cds_76"
                     /codon_start=1
                     /product="Hypothetical protein"
                     /translation="MAAVMLDRLGIAGTRDPRDTHFQDTVRYVRAGGRLYASIDRAVA
                     ATLSRAHGTSLAASAKAPRTNYGTDDGLDRQERLFGDDADDPVVRSAWWLYQARVAER
                     LCMADLDPDEFIEREDPLDLVVRPCGPIAQARRAAHLYSLAYSAEGLVCLFDRMQRAW
                     ADRAFGSDLVWIARAGGPLACVAARLALASRSTSLVSRRRYYTRQKIHHLSAKLRRNR
                     PCVRQQSGGVDDDNHDDDDDDHNDLQHDRAPGDVTCHDSVMMVPSTPTPATTWPVAHM
                     SLDFMDRLQAVINRRPPVAPHDAPLTPPFADQPDPWTPIDG"
     gene            complement(128777..129077)
                     /gene="pino_cds_77"
     CDS             complement(join(128777..128966,129028..129077))
                     /gene="pino_cds_77"
                     /codon_start=1
                     /product="Hypothetical protein"
                     /translation="MQRHTLASHVTSPDRHKQPTSDRAKARAARILARLKRWPRCVPQ
                     GRRAMPCMPSGKRLWRQTEPPNPSPINLSNGRAMV"
     gene            129563..130141
                     /gene="pino_cds_78"
     CDS             129563..130141
                     /gene="pino_cds_78"
                     /codon_start=1
                     /product="Ankyrin repeat"
                     /translation="MLAARDRRVRGGGGVAAATSASVCAACVRLVLVASLGVLLCTWA
                     YALEPWNNPTARALADDIRHRVTPGGTPPDQRLWAAASRCDADLVRSLLAAGASARFD
                     RGGEYGTPLHAVALASATARGDCIDTAAALLRVGADPYAVAAGRTALDVALAQDRLSA
                     VSGSRAVRGSPLGLFLVSAIDGQRARALSHRA"
     gene            130719..131618
                     /gene="pino_cds_79"
     CDS             130719..131618
                     /gene="pino_cds_79"
                     /codon_start=1
                     /product="Hypothetical protein"
                     /translation="MFWRRARTKARKASDAAPATATTDAHREPTRPSYDDVDYWEAKL
                     NPWLNPQTATERDAQAWPERRGAGAMTRTSASTASTTHRQGVGECIVEPPDAPYAAGF
                     TIHTGLITPAAFDARIPREARLVLTVGETHVPVAFDVREVVGAVEREGLLRSEVATCL
                     MGETDLVRARATARMAREQEIARLLFQPPRVEAVVDPYGAWGLVAAREARSTRHPAPT
                     RGPGLPAVALGVRPAAAMAGVGGVPAGPQPAYLRRPPAFSVYDDVTARDMGYIDMYGG
                     DDDDVQDVDYEGDDDDDDDGYDN"
     gene            complement(131931..134101)
                     /gene="pino_cds_80"
     CDS             complement(join(131931..132485,132629..134101))
                     /gene="pino_cds_80"
                     /codon_start=1
                     /product="Ribonuclease e-like domain"
                     /translation="MQGSTPATTVQPEPINDTDDTGTHDPCNKIHDNDDAFMERTDAT
                     AADGGNGHVPKDPIDVHAGADAGPAVDDYDATGASYAALFAVGGLFSEFVADHDEDFW
                     DNTDMMMMMGTTVTTTATSAVAMDQAPVPVQGLPYASVVDAIDALLATCPRLAECKPA
                     TLALPRAPMIYVIKRHMRDHGGADFGPAAGGPCATLDEACKRIDGLGPSYMLDRGVLA
                     WVAGRIMDLVRPGSSHGIPVTPLSHIGSHIRRHFGHDRWVLEDPEVLDMCIEQPEHAP
                     LRALIAWIVDSARTVAVLRARAATDGTLARQPRGKDYTNGPLKCLAKERDAHVRREAT
                     DSASWATFLREQAEADKARQASADKTRQTKKRRRLARTHPAPAGASTPREQETVSAAP
                     ATSTATTTTGPGEPGRALLTSSLPVVGAAHPMAAFVPPALPPAPLVQAPAPTTLDVTT
                     LDRQILAQIQSFALMSLAAAGPAGITSARPSPLWRRRSLVRAVVGAAATAANGATGAS
                     TDIVPVPTAVPIADSDKHDTAASTADEPTSSMGEPTTQPTTKVAPTSPKVPTSLSYDA
                     PAQAPTCAAESCRPCRDDPMETTGAASDALASVASPDGDDETIDQGLAPLRAAMLASA
                     TQQQQQQQPYAVPMVANMPLADHTYDLDQLLCGDGEDDWDALLACGQLPPA"
     gene            135444..137036
                     /gene="pino_cds_81"
     CDS             135444..137036
                     /gene="pino_cds_81"
                     /codon_start=1
                     /product="oxidoreductase"
                     /translation="MTPQGNNIPFMSRKNGTTATATLLGTAVTILLMLAVSADAASSP
                     FRLATGHRDVCVLGGGAAGMAAAVFAKDRGHSVVVIESADRVGGQCDTIDFAAPAPGM
                     PSWIDIGVQFFANTTAANEVGLGPWTIDSVGIVQRFAGPGSVYPLDFTTDTTPNYAVN
                     LLQGVSYGFQPPAPPTPEFLAAYYRLSVIIASYPWIDRAEVPSPVPAELLVPFSQFIA
                     THDLGPLVPSLFVPQLSGGGLGAFDKLTTLYALLNLSPTISRIFSVPYAGFVVAGGCR
                     GIYDGMRAYLTADDADNVLVNAKTLIAVRPYSPRLPVIVGGTLSTPDNGNGLAGSFTY
                     VCGKLIVAYAQTEQNMKPLALDVAERRLFKEVRQRYYFTGTIDAAGPVVDGGAFNMLN
                     IDPTSPFGTPALPAVTQITRGLPYGPVQFKATSQEPISVAAMRQLVVEQLARMPASLL
                     TNATAVDEFLLHAFQPHFTDAELARPGGAYAALEALQGHRKTYYVGALKNFAVTYQLW
                     QARTTSSPTASRPFQARSLSFF"
     gene            137717..139511
                     /gene="pino_cds_82"
     CDS             join(137717..139389,139502..139511)
                     /gene="pino_cds_82"
                     /codon_start=1
                     /product="Hypothetical protein"
                     /translation="MEVDMEDVDAIAYPQLIDLPLEMRAAIFSHVHEACDMVALFFAC
                     PALSVRPLADEVVDRLGARTVRLAIAAGAPLPTVLYLGTRVGMDDHRWNYAARGLLCD
                     AVCGGHLDVVRWLCLAGEIKDADPFCGVPPQDYARFMGDIAGRNGGTLSESIVEDNFM
                     AATTCDDAIVAWGEWDRNLQRREALWCYDKSSHPMRTEVHVAAALGMAVNADRGDLVR
                     CLLDYWPARSASKAPAQRLPRLFERAVRIGSVSAIEALHRLSCDDGQARCACPVEVGK
                     IAIQHGCIDAIHWLSAAGCRAAPPPTLCSIEMAMEHGHASVVQWACARIRKPLDSAVS
                     PKVLAQAAERGYVEALVEVHRLGAIASTRALALAAARHDQVRVLKWVAGEDPPGPPLD
                     GWAEPRLGHAAFASPAVVAWMLTRPDAHRLLTVGVARHALRLGHHVVPLLLHNAGIAP
                     LDQWNAVATVAACDRPYLPLIAQLIDEGALVDVEAVKIGIASGRVDLLAVLCLAAPFD
                     AIQAAVDDVAGSGQFCHCSAQWLAGNVPGLCLADAYGDSKVVASADLRCRVD"
     gene            complement(140051..141349)
                     /gene="pino_cds_83"
     CDS             complement(140051..141349)
                     /gene="pino_cds_83"
                     /codon_start=1
                     /product="Hypothetical protein"
                     /translation="MFRAIKSAFASSPSPALPAPALVDDCQALGDEDVGLRCLILLSF
                     RQDGQTAALVAVPPAKASGTARRRATAFFVAAPRAGDASGPAVPRALDSGTVDWEIAP
                     SLAPYAHALGWFLRGLARDRTVQKGDRWMGSTAAKYASVHSVYSRTAPVKPGAFPPHI
                     QRALEFTQNGLYNLFWNSDHCEDLRAVWIGEHELVSLLHTALAQRAARRVTPLAGDLA
                     SLAGAACGFKLWHAYCTDALQWACIGARPDEDGTVPEPERLIEAAKALGVEIPTDARL
                     DHGRLCAILAPAAIEQLCTVVYGAKPARLAHKSMSAATRGGILFGEESSGFSNLATRW
                     DYRRWRRLCAEGVSGHLSFSDAEIAVRYVNGRRAGIDQDQVMACIKEAEAEGGHEIAD
                     WEAWAPLMDIAMDIGAQLAPADIARPWRLCAVLAFAMCSR"
     gene            complement(142026..143765)
                     /gene="pino_cds_84"
     CDS             complement(142026..143765)
                     /gene="pino_cds_84"
                     /codon_start=1
                     /product="F-box domain"
                     /translation="MAMMATTTTITADQQPTPLGLVDLPAEIIAHILGCLASLRDLAA
                     CAATCSALVDTTVLEAAASRFAHATGPRLLEAGAPLCVIQRVVQDRALTDVPHEWVVA
                     ATRGGRIDVVEWVCNVATFPPATLPRAKRARRERDDSNPAPGQKRVRPDIGYDSPIVD
                     ALYESLNHGKHDAFAWLLDNWERPVFGLPPGPSARPVESLLVEAVHAPGTPFDIIACI
                     HGDCPSTRFTCSDTVHLAAADADRVDVITWLHSLGCRCNPPSPSDGDANDLFWRALYT
                     GSAKVARWVYDTFGLCKHRHRCATEFQRIFVDMGSRGHASCAALLCNLGFGPLSVEFL
                     IAAARAGHLDVLSQCMDDRPGDDNGAGGHRPIVAVGWPSLAIGHAAAAAGRGDVIRWL
                     AGRADARRNLSVGAARLALESGHFDVVLVLHEAGIAPFDRWNSLCAALTGDIARAGHG
                     LTAPTPTTVALVAAYVGACDPSVFVEALRCGGDQIVAFLCERYGTGHAQAAVDAIAGS
                     PLCENALLWLRDNVAGICVADACAYFHMADVDGYSRDKPSPMCWCPRCFQQAPSPIDS
                     MDDVQNRLLGRGS"
     gene            complement(144166..145791)
                     /gene="pino_cds_85"
     CDS             complement(144166..145791)
                     /gene="pino_cds_85"
                     /codon_start=1
                     /product="Mycoides cluster lipoprotein domain"
                     /translation="MSATATPKRRLAKRTKRASGAAVATTTENLAAHDSFYVLFPQPD
                     STDTPEGQAANVQEPFAFNDGETSMEVDKDAPTPAIATGADDAVSVLPVPSTTMATTS
                     PNMPLAAANNDTEDGEPSSSSEPVDGTDAPAHASSEPAPMLTEEPTEESAVNNTVPDT
                     ALPPAETAASPASSADDAMDQQQEPQPTNNKRPCQDDQPERETFADGGHDDVAKSTET
                     NGDTAEADDESTVTLPLKKRRRMLVPEDDSEGNDNATEAVTTAPDRQRIVAEGDDDND
                     DDEAKPLDAAEDSTAGKPYVTNMDDYPIAMRYLGITRLSTLIGYMLALVVGGHCRWFR
                     MGPAGPSGRPALEISPRFDVAADGRRFVCGVDCRLYGGDGMIERALPHGSTVSGKGTA
                     FDPYVATIKGIVAVTPEIVEPLYDDWAPSLFDQRLARPQDKIVLRFALADRADRDEAC
                     VPLACLLDDGDDTPSALPLAKQRVSVHLDAVWRRLAAHPLMFKTAAKHAEHYRSLVAS
                     AAATATAASSAATSASSPASPLTAVSTPIAASS"
     gene            complement(146572..148278)
                     /gene="pino_cds_86"
     CDS             complement(join(146572..146604,146758..148278))
                     /gene="pino_cds_86"
                     /codon_start=1
                     /product="Ankyrin repeat"
                     /translation="MCTIDNLPDEVLVKVFDRLDCGDLYAGAGATSVRWRDIAADVAT
                     RAEPVCARRARAADPTRIDYVGPYIASLVGHTDADWRLYAHDAQCRWRGHACLRAARD
                     DRITILHVLCRMLGHPWVPGVCVEAASRGHLGLLAYATTHKHPYDRAACETAAAAAKQ
                     HVVLDWLWDSQPPTRDSVDRAAESGDIGLLRLLQRRRCPRSESTMAAAARGGHVDVVR
                     WLMRHRCPWDDTAVLAAATAGSGDVLALLLARRNVRALPEAVEAAVVGGDVACVDLLW
                     ARCVDDDSTDQSLKLSAPGRSSQQRASIVNETSQKGDRGNAVVALYGSRWVALAARHK
                     DTDVLDWLCTRGCAPTPGAMAVAAGMGNMDALLYLRQSGCAWDARTTAAAAAHGRLDM
                     LDHLYRHGCPWDATTCTAAAAGGHVECLQYARRHGCPWDRQVYVTALNHGHTHILQYA
                     HEKGCPHDRTVCRAAAKATSSSDAARFVCHEMCRHRVGRCDLKPKICRRRAVSSPSSE
                     LSARVDPTL"
     gene            149245..149913
                     /gene="pino_cds_87"
     CDS             149245..149913
                     /gene="pino_cds_87"
                     /codon_start=1
                     /product="Ubiquitin-like"
                     /translation="MAAVVYVKLPDGRCTVARTVSFDGEKHVLSLPPDGTTVDLGIND
                     RRWSNADPVGECCVCLNDGDDDRRTLVWYHGCTCTKLRVCQECASAKGPRDGDSNSGN
                     GRDNQYRITRCPVCRHDDPTLGWRWRPYSTIDTIAGPVPEGEWTVQLFCKTLTGKCLA
                     MEVHLSWTVETVKHIICARWGIPLGEQRLIWAGRQLESGRTLLQYGISKECTLHLVKA
                     LRGD"
     gene            complement(150064..151557)
                     /gene="pino_cds_88"
     CDS             complement(150064..151557)
                     /gene="pino_cds_88"
                     /codon_start=1
                     /product="Hypothetical protein"
                     /translation="MDKMTTATDPEDLAQLYPRLDAEGVGWLKAIAAALDGRTPYMWV
                     PTSAGLQWSVLLSDDVPQCAIFITKAYALVAADDRPWCVRMAIDDHDPRRLRFGVFVR
                     RGHDAAPSSSIPPLDGTETLAPTTVGPVAGTPVVTAQTVDDIGRLYPWLSAADLVKAS
                     ALHDALAVVPHAWATHVPDGPFVNAEPFSNRGVDEMAAVVRQRQCEWPPSCRSQPVIL
                     ALDISVGHICGRQQTDLLFAPATEAPLDRCVLGQGDGIHLGRMYPFLGADDLKALEAL
                     DAAIVGVPHRWVVSTHPEPASGSRPIAIDTKVAGEWLGIVCREATTDLGRPCRVVLGV
                     RDATGAVRYWTEPIGDRKTNYVDNIADDKCKHVDGKADPIDSESELTTTTDRCSSNVN
                     NIAMPAGFETLARLPVKGGGEVAWLCALAHGLGSVPIAWTRTRSATDIVLHMSTPAES
                     AAMVVGATYHRGRNANTESARAVLGIDSDAAGGPLFFYRIETDTQTD"
     gene            complement(152033..153880)
                     /gene="pino_cds_89"
     CDS             complement(152033..153880)
                     /gene="pino_cds_89"
                     /codon_start=1
                     /product="Hypothetical protein"
                     /translation="MTCPPTFAAPVPSDATEVEAPSLPADLFMRMYPWMSAGDACLYA
                     AVDRDLADKVGVSLPGIVEGVLRRQWTAEPLVGDNVETVTIDYEPNGPLGRDKWGIRN
                     GPLRHEMWASHIASSIFMDYAVLFDEESHADVWSGHRLVLSVAANNRYEVRFKVEYVG
                     AEDRRVPARPRDTALTTVCPGMGRTARQMALAVDIDAALATVPHNWVGPLDDVFDGAD
                     RSPIDHDTLDLHVRHVIAEAHRRGVRYSPVPRHVTLSIYDDIGRDGGPLFAFIVCHDD
                     DDSGDDDNSVGTGRTRSTQWYATVRPSVDRTFLVATPSSQRRRTQGTPWQAVHKTDNT
                     CEPLFGTGKQAVAAPAERRWVPCGDRAVFGSHRASSAKRDTNANDDTRADRDRDNDTI
                     FDKSERRAVHLCNDPPPLSSTLSTPTQTVADIVALYPDLTADQAARVFAASAQLDGIP
                     YVWVAIEAFGQGRCMATSLRASAAAMLRVKSDYHEAAWYAGRRVVLALGDARDTILYR
                     IVGRAPEAKTQTNDDLAALYPHLSPIELMGVGAINRALKDVPHVWVGGAGLYPHRVSL
                     GPGGPPVTATIEAVRRLYDHAAASRLVLALGRTPAGALTLNYGAVLPRR"
     gene            154651..156974
                     /gene="pino_cds_90"
     CDS             join(154651..155573,155675..156974)
                     /gene="pino_cds_90"
                     /codon_start=1
                     /product="Hypothetical protein"
                     /translation="MDVRLRKGTAVRGGCEEGNGWATLDGILSVGCGELGMLVVRHLD
                     PDSYMALASTSRSMRDLLASDGMRRGCARLMQKGRVLDVWVDLAGYPELHPDDDDGVA
                     PPHVVYVRCRREISRHVRYLVHACLDVQRRLETPEPRPLPRVTVEPSHLLHPRDRLAH
                     RNCNRAPPRSLGTRDEKAASATAHKCYATLAHAVWAMRRCLVRTLAGAIVTNNVPVAH
                     LCIDTAVRLTGSTLHALGFTPAADRFNPDWLDHYMHNHGKNRRAGLPASPLGNPFGRL
                     LDGVDVDLPTHYEPWLAAASEVDERSWRPFDMAYAKDAAIMDAWMHRIGRDRCDEIDE
                     HISAYDFSLDVWCPVKWMAAIVSARTTVIGAADPLFDALSQGLSGVVRSAQVDPIDYH
                     SDSIYRFYRHNVFRWPATPVMQHSWRVQCQAAVIAAQSAHGKATGFDASPLHTRAARA
                     SASCSGTTAADNNKHSVLHLFADYRACLLPVPVETWPVADASAALDAIQSWCHSASTA
                     LLAGTLHPHQRLDLLRAAVSLVLHIGQATTDSPRIEILDYVCRPVLFGALVGAVRRAA
                     IRCPASMDVLMTLALVDLRQARALLWTLYDTAAHGYRCKRIVDLMNEPEPTDEPPADA
                     ANADPDQQYDEARARIVYWSRLMASGFLTESGATDADAKFALVEADAHRAAALTRIRP
                     LPNVVVAALASQPVDPPKGRAAKKIGHQRQPVGTVARPRAKRLRTNSKHGSPAAARQR
                     "
     gene            complement(158120..158425)
                     /gene="pino_cds_91"
     CDS             complement(158120..158425)
                     /gene="pino_cds_91"
                     /codon_start=1
                     /product="Hypothetical protein"
                     /translation="MSTNRAAIVESTDTQSAPAGSTESPESAGSRTKEITTGAAHAQE
                     QPARTDGPRDCRRPVDPVWRALFEALRNSPASRDAVAIPADADDEPLTEALYRMRGR"
     gene            complement(158865..161519)
                     /gene="pino_cds_92"
     CDS             complement(join(158865..161443,161516..161519))
                     /gene="pino_cds_92"
                     /codon_start=1
                     /product="Hypothetical protein"
                     /translation="MACRLGALALSLCRHPTPLFLCLWTIPSRPPKEKQEIDRKKRGQ
                     KGLFFFPDSDGVWKKLATTVAAEIVVFGREAVGRLEAPAGARTRPLAALSRHAPASRS
                     PRHHAHPSARGPGRGRAMRRRRQHRRVHAGVARPGRRLCPRGRPASRLAGAATAPNGR
                     AGRRPRSGMGAPARPRSGSTALLLSSACFHAAAAAGRLATLQWLYADQRRHGTLVCCS
                     TTSVLCKAVEGNRADVVEWMLDAMGPLCSVSHAVAKAVLHGATDALAVVCRHAGGDDC
                     VHECPRKDATGVAMLEYLDRRGLLDPCDIASLLQASNPVHLRVPRESAIASIEWLCRG
                     AFGDRCDWSPPDVRRALSAALCGSETRDAEWRRLVEAAIPRVHADDLWTDLLFEAAAR
                     GAVDVVERAWPLATADACAVGDAAAHGGAADVIDWLVANGHATLWDSGLSWTREAANG
                     LRWGLVHRLYVESAASAPRADDHDLYGTATAIYGIAIEACDVDALVRLRAVCADVLGS
                     DREQCAFDNVVSDPHIGGQFHNGDVEIYLCTHALDKIALPRRLAPDLAFASDEVFVTL
                     LGAVPQVTFCRSLIKSLCNRGREHIAVMLLTARPDVARTIRCWPGAPERVCALLRPDW
                     DQAHRLRHRALSGASGVGAAVAEAVASGRSVCDRLLGAFVTKSLGDDIIDVEAGALIA
                     ACSPKRLCDAGIDALCNGRLATGRRLLEEAAARGCLPEASLLSTAFRRLEQTSGWARD
                     TTADQASTYEIVAYISTLRSDSLRDVCTAMECIGHGDVARGVAILGDAPPESLNPNKK
                     VASALRFGLAAGHLDGFAHVTARGDAWAAALSRAVPGLADTMARAASDVGRRGRGCAV
                     GRPV"
     gene            161920..162171
                     /gene="pino_cds_93"
     CDS             161920..162171
                     /gene="pino_cds_93"
                     /codon_start=1
                     /product="Hypothetical protein"
                     /translation="MDRYLYLHAARGTVGCRAFFCAVLERATDDHRPGRDLTRRRSAR
                     WRRQGLAWSHLPDETWDPIVDGTRRHGRAFLPRPRLGNG"
     gene            complement(162450..164649)
                     /gene="pino_cds_94"
     CDS             complement(join(162450..163228,163341..164649))
                     /gene="pino_cds_94"
                     /codon_start=1
                     /product="Morn repeat"
                     /translation="MDQRRHLGALPDELLAMVAAGLDAVSLGKAACACRTLARICTDP
                     RPWRALCAASGILDAPLYAPIVRGEVAVTPDPDDRWRWLCVLAIAGDDRRFAWHGGTL
                     GGSRERAPGLPAVRACSVHGWFDAAQRVQGFGVEAANHLSTPAEYARWYAGTWEDGLW
                     HGHGVRNTHGTIMTCQWRRGMAHGPGKSVSVSGQCSYVGAWKGGARHGRGVTTCRISH
                     IQCAGGWVAGRNQGWCVFSKLDDQTPSSGLCFECDFTWGTTVDWAVVGDPRVRSVGLW
                     CRNDCDTGKRTAIEYADRSVFETPCRAPSVSYATMPTGRGVLTLADGTRLACDTWDRG
                     SPTGTVTRTRPGSPLACAEWWSEWNLGGCDLVCCVDKTPGPVSRDTAILSKRTTYETL
                     CICLDSPCTVDATDPAASPPWSGSRSPTNIIYYPEPRARPTILSGLKEPLEHPLSQRV
                     NTDRPDWTMCRLTHTSVPIAECVITLGGHLMWNRAAKAWLRTLGRCMGVDETPSHPAE
                     IGDCRVLAFDPAWTAAGLAPTEVAYVAKRSFGAVRNVYAPVERRQYDDLCWYDIGAAW
                     HAMVTAVLVADCRARAATPYDHFERAVAIAGGRPGFHAAALTGVDLVGVTFAEATSFC
                     ASVFTRCRFFGCTFERCLYIGTEFVDCTFVDCTVGGVPDAVLGGPHGRVTGDVSVVAR
                     ATARYGASFATLS"
     gene            165285..166415
                     /gene="pino_cds_95"
     CDS             165285..166415
                     /gene="pino_cds_95"
                     /codon_start=1
                     /product="Phospho-2-dehydro-3-deoxyheptonate aldolase"
                     /translation="MDKTIHADAPCATEPVPSPMQLAEAVPLGAEGVLRVADARRQVR
                     RVLDGSDPRLLVVVGPCSAHDPDAVVEYGRRLRALVRAEVDDALCVVMRAYVEKPRTT
                     VGWKGLASDPDLDGSCRLADGLDTSRRLLRDLVVDVDQPVAVEFLSPLVAPYLADLVT
                     WGAVGARTTESQVHREMASALPLPIGFKNSTDGSVATALDAVRTAAQPHTFLWVDGQG
                     RIAAHHGVGNPSGHIVLRGSPGRPNFDASSVASAVAAAAARRMDPLPAIVIDCSHDNS
                     GKDHRRQADVVRDVAGQIRAGCAHIKGVMIESFLTEGRQNLPMVDTATGDDTAVGRDN
                     VLASLQPGVSVTDACLGWDDTVVLLCELASAVRDGRRPLLRV"
     gene            complement(166626..167942)
                     /gene="pino_cds_96"
     CDS             complement(166626..167942)
                     /gene="pino_cds_96"
                     /codon_start=1
                     /product="Ankyrin repeat"
                     /translation="MSSHKSDQRRRSRRYRRKKCAAVAKQDGCFIGHLVPCCAPAVAP
                     RNPPPPPQQGIGFGDLPDELVAAILARLSCLDLCRDAARVCTQWRAIVYDASAIGRSL
                     CTSAGAREAFLQGPLLAERVGGFGGPLLKENLAPARRGRKRPLVVLARMLAADSGHVD
                     CMARLNAHPWYDGACLVPAAVHGHLDILEYAHVNRCPWHYGVCTAAEAYGRIDCLRYA
                     HGTGCYWTGYCNGAAASGHTNILRYAKEKGLGKGMFACRFAAAGGHLDTLRYACENGW
                     PTCSGTPSYAAQNGHLDVLKYVNESGGEWDSGTTWSAASGGHIDCLDYLLKNGCPGFD
                     TACDWAAEEGQLRTIEWLYTRGFMWSEESCAAAARGGHLHVLEWLRHHGCQWDRWTAR
                     YAACWGHFDCLVYAVENGCPLGATGDLLSVAHDGGHQACIDYIKTI"
     gene            complement(168346..168756)
                     /gene="pino_cds_97"
     CDS             complement(join(168346..168445,168557..168756))
                     /gene="pino_cds_97"
                     /codon_start=1
                     /product="Hypothetical protein"
                     /translation="MGQANTTIKGYSAVSQDALFATYGVTEGDYQAKASAAIDRIRAM
                     PDGYASPEDRAAAINAIRTGTCGDDTELLTRVRAALDRWQRDCAATGRALKSKVV"
     gene            169539..170069
                     /gene="pino_cds_98"
     CDS             169539..170069
                     /gene="pino_cds_98"
                     /codon_start=1
                     /product="Hypothetical protein"
                     /translation="MDAWVHEGGWPNDDDATYRCSDSTQLAALHEWDDCIYNDILSCP
                     TDDDNVYDSIGPCIDHNNAVGGAGGNGNDNGVSGLGECPVAPKHTPYAYGYTFIEGFI
                     PADSFDALVRPERRLVVTVGGVPVALDAAEVERAGNVGRPLMTPLGSCSLGPLDVERA
                     HEADEALYEREASAAF"
     gene            complement(170960..171730)
                     /gene="pino_cds_99"
     CDS             complement(170960..171730)
                     /gene="pino_cds_99"
                     /codon_start=1
                     /product="Orotate phosphoribosyltransferase"
                     /translation="MGDMSTTQSPIASETLPTAGQDNKVVLRWGSADATSRRGTAETL
                     ADHLVRTRAIQLRPRDFFVFPDFTTPAYCDLRLALGDADARRAITDALATAVCGRFLA
                     GTDTDVDAVPITIVGVATGGIAYATGVADRLGLPLAYVRAAPKDHGKGKRVEGGLAAG
                     GRCVIIDDVLGTGAAALNAVQALKDHGAHVLGVCTIFSYDFDTLLGNVAAAGVPYVRL
                     VDFATTIDRAQAAGTIDPAGGDIVRAWYTPKSTWRSSA"
     gene            complement(172495..173109)
                     /gene="pino_cds_100"
     CDS             complement(172495..173109)
                     /gene="pino_cds_100"
                     /codon_start=1
                     /product="Guanine deaminase"
                     /translation="MNTSTHRGDALDVVARAIVANAVRTATDAVRASQPGLFTAAIMG
                     PDGQVVAHGRNRVFDACDPTAHAEIEAIRAACRTRGSIALDDCVLCSNAEPCPMCLSA
                     AYWAGIRLVYYACPKETVAKAVGFDDARLYVDLALPADQRTLIKTVHVDCADAADAFY
                     AWRDRETLASTMASTVSPSARVVSLSSTASASAVSPPLDVSTIE"
     gene            complement(173486..174136)
                     /gene="pino_cds_101"
     CDS             complement(173486..174136)
                     /gene="pino_cds_101"
                     /codon_start=1
                     /product="Hypothetical protein"
                     /translation="MTYGGDCEMESLDGSDDNFQVPSTRPVTPRRQRRRCRCTRAWCF
                     WNVCCPAGVAAVFALSLLVAAIVFVPWFMVGIRPDILLEESMEPTTCLITNHTVIDTK
                     PVDGNTRLLYMPGLGVVVSLDMHVAVATARAERSDSWMSAEVMGDYFARHPINATSPC
                     YTDGERVAMRPGVDGIGKALGGCISITITAFFTALLMFGVAASVLLTCVHLAMLVP"
     gene            174478..175680
                     /gene="pino_cds_102"
     CDS             174478..175680
                     /gene="pino_cds_102"
                     /codon_start=1
                     /product="F-box domain"
                     /translation="MASADIFFGGLPAEILSDVAALLPVVDIVRLAMTCRVMHALTTC
                     QAMWHRLFVRDFAHLYNKGLAVQSWPHSDHPDDPWHVMAIELWRGTDVVSVMPPRCRP
                     LEGLPPPFAHAFAAGKDWRWLYRAHLTTSSEPPDESFSGPRSQRLDPSTLGVADWASG
                     SSVGYTAEIIFGGHNGDEVVSWTEFTPAQAPDDCYWSVECTATSVTHRGATDASGIIR
                     SLSFRVLVCATGWPSTRARLAPLPACPQTARATMVGAVTATSRQSHHIIPMGAPLSVQ
                     CATAEHTASVGSHMPTATLSAPITLTACWQTRLNLCVLWRVRGQNGQGAFSPDVHGAR
                     CRSMSREARHAPLFRSTTATTLVSFGAMSPRASSGGARARGASSWTWSAPGLEHRPAR
                     RRKHRVIK"
     gene            complement(176048..177343)
                     /gene="pino_cds_103"
     CDS             complement(176048..177343)
                     /gene="pino_cds_103"
                     /codon_start=1
                     /product="Ankyrin repeat"
                     /translation="MDPPAGATITAPPAPADNVVRATLLDLPCEILTLIVHFIGDDGD
                     FCAARLAHPCFDVEDRATVHRTRRLPRWRRTDPHLACRRNNVEAVGVLHEAGVRFDLG
                     HVKLAAMSGAFDVVLVLCAWVCAEDWLPAEIMEDAASAGRLDVVVGLHATGRAHSSPA
                     AMDAAAANGHLEVVQFLHENRTEGCTTHAMDDAALFGHTDVVLFLDLYRREGCTVAAL
                     NNAALNGHLDIVHLLDVRGAPCDTDAMDSAAAHGHLDIVAYLHDHRTEGCTTDAMDDA
                     ASRGHSDVVVYLDTHRTEGCTTAAMDGAANAGRLDIVTYLHRRRREGCTVWAIDGAAG
                     EGHLDVVQFLHEEVKIACTTDAMNLAAAGGFLDVVIYLHTTVGATCTSRAVDRAAAEG
                     HEQVVRYLVECVGAPFTAHGVRNARTNGHRGIAAFLSQQ"
     gene            complement(177830..179616)
                     /gene="pino_cds_104"
     CDS             complement(join(177830..178637,178724..179616))
                     /gene="pino_cds_104"
                     /codon_start=1
                     /product="Hypothetical protein"
                     /translation="MTSHLLRGRARILSRWSSDEHMGHDDQTSDVGDPNNHGDHGYDD
                     HDDKDDENVPFFSDDDTMARGPWGVRAWCARTRNKAGNHLGIMAAWLSMAAAAYWMAA
                     LVPGLAVFALTVEIDDRPGGGPYTTKALLSIARAAAMPIGFAAALYGPLRRAAVDGWR
                     RPVSLFTLPLVYSIVPNAAFAMGAGTTAGVTVQLAARFSGTAVISLAFCAYLAYAQGR
                     RASDVAMPVATLAILLAPAASRPLSTGIADLLAGVGDGSDSDGHLWMPLAVSALCVVP
                     TLVAALALALTPPPNQADMHARTVNGAWFRRHWAVIAGLAVSNAALQGLGAVRDVFTA
                     DLVGPDAPWWHSVVADAPTCVAACLCYVPLLWVKDNRRAFVTIGVVGVVAAVVLVVTG
                     AASLMDWLSPLAFLVVGGVGHFFALVPFSGGGIVFERLMGASHMPVDPLLVNVACQVP
                     AYVAGLGVLLLAPAATHPAAFFDWTAVLCGGLLVASCAWTLVAAFCVLPSQSTSPLVP
                     VPTTPLDGDPTLSTDASVPWTMGSESAPVTEMSSADGGAFLIYVGDRPRRSYADVTVL
                     "
     gene            complement(180901..183264)
                     /gene="pino_cds_105"
     CDS             complement(180901..183264)
                     /gene="pino_cds_105"
                     /codon_start=1
                     /product="Hypothetical protein"
                     /translation="MDPSWWLFGLLWSVLVSCATYALLVVHMGIDDHRGDHHYRGNDV
                     NMADDPHVEHAHSATCSDLSPPPPPPPLPPLTLTDLPPEMHVHILSFVDPRGVIAYVG
                     AARALRVLGHRQALALRPLAAIGECLCGCDEIDWGPRADHLDSAVAMGCSLECYPLHF
                     RRLLAYGGLDDARVLYRRLEAFMLPAFIHLGERESRWALASWLLCHALCNEMASARGG
                     EAAAALAHEVAGTCVFHEYIEYIEDPICIGEAIERGHRACAKACLDWRGTKADAIWDL
                     RMASVLYNAWRARSNPLALDRLAARCQAVCELNPHVFADAEAVRSQIIGWNNTSRRGT
                     IKADAFAVIARHVPGLRAIGLVHSHVRLWDHHEERALVEALVCGRWGDADGLCARASD
                     AMARGGPQAKEKIRRAIMEIAELHLHRHVVYVADASVGTNTLAILTRHLPHDWGESLR
                     TILFGECVRRRACSPCSAVLHVEHLAYLNRPSFKDRNVCAALWPSPRKHAQSIRWSRA
                     RALALLRHESVRWPVRAALTAAAYGDMEVLDVLAPERAATEEPGLSGSPSWTADVVAL
                     LVAAGYIEGAQAMASRYGVDIERVNVAAKVIGLDQAHPYKPCSGLYSADLPRCPLPLS
                     VLLHVPRVGAKSTFIEAIRRGIGTPMHPVDAAHLAATFVGALDGADASHVLKPTTAVG
                     AIDWLCGQTYMRFDAAYVTACASIGATGVVHYLVVRRGVACDIGAVSRALCNRGGHSY
                     SGSGSNDTENGAEVRVPSEAALPPSWIDFMEPALRAAVGAQRAPVGD"
     gene            complement(183552..183740)
                     /gene="pino_cds_106"
     CDS             complement(183552..183740)
                     /gene="pino_cds_106"
                     /codon_start=1
                     /product="Hypothetical protein"
                     /translation="MPCDVPEPDLAAALERMRAATSRQSVIVRAMSTLQNMLYNVVIH
                     AFLDAYQRASSWRRFFSP"
     gene            complement(184418..185383)
                     /gene="pino_cds_107"
     CDS             complement(184418..185383)
                     /gene="pino_cds_107"
                     /codon_start=1
                     /product="Hypothetical protein"
                     /translation="MFHPSIAHLNTAKPSPQWGQADPTGTRNYQWFIEEKIDGSQLSF
                     QRQGDTVEFRNRSKVVPVDVALDRVCYANAAQAILRLATQLDPAYTYHGEAVCKRRHN
                     VVAYLGTPLKFWICYGIYDGERHLDRPAMEAECARLGLACVQVLYANADPAVLDPAPK
                     VLDIVKQIEAGQIESCLGGTVIEGVVVKHNAAWHARPKAYKPVQFKHVTAAFKECHGE
                     RRPPLPNHDAESLLAYLAHIGSNFAVPAVYQKALQHIREDPTKRESISLPVVQREVER
                     DIRKEHGQDIAEALLEAFLPVIMRHATAKVPQWAADQGDLLPKDE"
     gene            186003..186302
                     /gene="pino_cds_108"
     CDS             186003..186302
                     /gene="pino_cds_108"
                     /codon_start=1
                     /product="Adenosylhomocysteinase"
                     /translation="MCDGRGESEKGHGRKIYLIAGGNTCNVATGRGHSADIIDITFGL
                     KLRSVLYLAERAWPAEGDNAAAPLENKLQNLPAKLDASVARIALSSRGIETDFCD"
     gene            complement(186779..187417)
                     /gene="pino_cds_109"
     CDS             complement(186779..187417)
                     /gene="pino_cds_109"
                     /codon_start=1
                     /product="Hypothetical protein"
                     /translation="MKPMRSFFLDVMRVPQAPTVALFVGLLMMTALVTAADPTPTPTL
                     SLDRACEMVLDGATCQRRCECEWCPPGPDHGCHTINLAGACGGAPGQRAPHDTCYDDP
                     ASGVEGLVVGAVFVGLALVAVGLWWGCICVRSLRRRFLRLGTCASAVDHDIGAPKGDP
                     SVNHALGEPLDCASRCDGPAMVPPRRPASRPIDMPRPRAPVPSSVYAPQQYQ"
     gene            complement(187605..188108)
                     /gene="pino_cds_110"
     CDS             complement(187605..188108)
                     /gene="pino_cds_110"
                     /codon_start=1
                     /product="Hypothetical protein"
                     /translation="MIGEAWRRRVVVLLVLLGAAAAAAILCAAGILLHRHATVDMVHD
                     DDAVEAPPAAGSGCPSLVHAHRCKARCGCEWCPPGPGFGCHEAASGRRPCGGHNGHRR
                     AFWTCETHLATWLAVGGAGLVGAVFVAAAASLWACWPSMCRPRSTSVDHTTGLLMPLD
                     ASASVNA"
     gene            188598..190029
                     /gene="pino_cds_111"
     CDS             join(188598..188732,188962..190029)
                     /gene="pino_cds_111"
                     /codon_start=1
                     /product="Hypothetical protein"
                     /translation="MTVLAGLPIEVWALIVEQCDADDMPALTKHAPRCASMRGSARTA
                     YEWVGKTTVTVMMTAKTRPTTMTTTAARIEMMTATATTKCRPLGGAPMGHHGGTADPT
                     RRGCATHARRALAEIHYANGADEERDDRTADCHRFHTICEIDLDRRHVWTSHRWDFDG
                     ADFVCADRVDGDDFVVPAPVAHLLDPCLFAQLARDNAARSPSAYGYDGVLARIGSIRG
                     WMPLRRRTHAPACPSTSAEQDVLMICCDATNPMWGALAVVRFHGPRLILGWHCGDCSL
                     GALIARWREHPLRLQDARFAMDWVEWAIDMYVADMRRIRQKEAQMTIDDHGGTLERML
                     AEDHASSGKPWVQLLDAEEHSLFFDDHGTVDDSLDHGGGLYETARQRQRQRQRRRHHK
                     GRRGVRAH"
     gene            complement(190315..191157)
                     /gene="pino_cds_112"
     CDS             complement(190315..191157)
                     /gene="pino_cds_112"
                     /codon_start=1
                     /product="Hypothetical protein"
                     /translation="MNRTDGVSVPQLYLKETSNLTANAPPTPPGNKCSIKGNRRRPTP
                     KTNIPYMQVNAPRHAQLPPPLAPRTEGPPGAPFCVGAFRHFLRNLPPTAIVDLADEPP
                     IVVMDSARLRASYVMFTRGSTDAARLLERLGIAGPFDHLPAMPVRASAPPIHDAQTPA
                     VGARLCHPKRARVTSQCASRPLPTIYDLCCETILDQARAAYRAEGPAAAVNAVADVSS
                     VLTSGAPVDVALYACGATLVTRDVALGMLALGESRSDIDAAYACVAWTVLPHGSVGVY
                     ERRS"
     gene            complement(192167..192835)
                     /gene="pino_cds_113"
     CDS             complement(192167..192835)
                     /gene="pino_cds_113"
                     /codon_start=1
                     /product="Hypothetical protein"
                     /translation="MNTPTTILLCLALCLAATAASAHPRSGGGSGRPCRPGQGSADAP
                     YDVSCPRYYYTRAGLERAEPFKGTNLGWFNCTGSASLHDAVTGQHIASFDLPAFNLQT
                     LYDADCGCWMTTNSLTSYVVREYGTGMPGHSQQGSCFEYEFDPVDGGDQPLKTVARAT
                     NVGLFHRDVAYMHAETGALAGQQTIVLNARGDEMVLLRLYDPVTRVPTFVQNVVCSKF
                     AAQP"
     gene            complement(193730..194865)
                     /gene="pino_cds_114"
     CDS             complement(join(193730..194309,194381..194865))
                     /gene="pino_cds_114"
                     /codon_start=1
                     /product="Morn repeat"
                     /translation="MDQSDIGPCTREMYRPGLDTLASELVVEILARLPCANDIGRCAL
                     LSKRFADLIAHDARQYAWRCAAEHAARLVRLGGPWLAFAASTQGWPWVRRALDPWTPF
                     ARGGIGFLRKSKRTYMGEYHADKRHGHLIKIVSAGDWAFYGPVDANGVSDESWFAANG
                     DTVGGRSYYGNATVTVAINCTCASLLPGGETGLGAPAAHDDAGGPLQEPTSSSPVTSI
                     VHAAHGTAFTRAFGCVMRTTYLRERPVTHYTFLFCNGDLLDYDLKTQGGDTFASVRMV
                     LSPHCPDPRFRSVEIKADIWRHHAVENPGGIPSTLLYPDPIQSPDMFRIYHEYFKTGF
                     LLVRECQRAAVAAILAREAL"
     gene            complement(195173..197037)
                     /gene="pino_cds_115"
     CDS             complement(join(195173..196690,196825..197037))
                     /gene="pino_cds_115"
                     /codon_start=1
                     /product="Hypothetical protein"
                     /translation="MEEPATTASVLLAADALPTGLVADTPADGRAASYASRLDAACRA
                     WNMNGDGNGEIASDPLLTRLDEQAFFDWPPGIEDIVGVYTSLVGTPEAHFAAQEIIGQ
                     EATRSQGLARLVRNIGALYRRRTENRMAAYARRTTMNAQGSLTLRAIMGDWPRAVDPR
                     CDPDDDTLYLVLAERGSRGATVHLVAVTNGGAEAHVLGSMAPDRDFVGDLDALPPAAR
                     PYAAFVPWLLDGAGLPDDEIDVGVGDAVLAVRAIMALLPSWADLPPAVLDAVRPWSPG
                     RIMRLGGRSKTWAAWIDACSLALLLSQMAGADAGATAIQLYRPDMRPPTDPFAPVTQV
                     ETLMRLAHEAAVGATEARFDPTGLPMELAEPLAFDMWRRTCALPTAERGPDGRLVGAD
                     RLLDVAALWGVKPTPAERARPDLLCASLAPTAVARGARVLRGRDTLPLPDRVAPLFGP
                     VILDEVERGAWSRACDGLVDPDSVGVADNPRRAVFGAYRDIVGGDTDEEEEELVRRWA
                     PVRDRALDVVQQAHAYARTRPDLVGPSLTDKARLALLALRHEVPIEAGDLVTFDAACA
                     ALAPLAVLWP"
     gene            complement(197700..199016)
                     /gene="pino_cds_116"
     CDS             complement(197700..199016)
                     /gene="pino_cds_116"
                     /codon_start=1
                     /product="Vv a32-like virion packaging atpase"
                     /translation="MNIASPSSSPAASPPPAVRIPRDMIALFAACANPNLVDLNADPK
                     PVPTDAPLPCETPRPGAEVAPAAGSAGAQESPSTADVSAPSAPIDPLVRTILTQAKAL
                     DDAAKSLLALAGSRTSAAEFERARVELGWPAVPIVETPRPLSSSPAASSPRTGGDGDS
                     LVLHKFNHAAHKHGRINFVIGKRGTGKSVLLKDLLCSNGNQWDVVVGMSPTPESQAML
                     REMFPASCIHDEYDAAAVARIVSTAHALRSAGFHPRILLVLDDCMFDTCILKSKEMRD
                     VHMNSRHLGIEVYNVVSYVMDIPKAIRSQIDYVFALREPQRAYRENLYKNFFGIFPTY
                     KEFSTVFNACTENFGCMVIDNTARTNAIEDTVFWYRASPNPSTMLLGSRAQWLLHHMF
                     YKAPVHVLSDDDIIPALASRYTDRSRALEAALGDPIPVLTRLRLVD"
     gene            199783..200439
                     /gene="pino_cds_117"
     CDS             199783..200439
                     /gene="pino_cds_117"
                     /codon_start=1
                     /product="Hypothetical protein"
                     /translation="MSADAYEVYGHTLMGGVNTAPPTSDQSIHHMYSKILNGGQPADL
                     VFLPVTTSIGTSTIIYEYITQFNHTVNFWLVPYPPTNRPVSVAMVLALGFDDNNKMTY
                     ERFYLDSASILVQIGILKDGYGIYGNDLPQPYPSASACKRHLPVIGDQLTSVLLDGPY
                     NTDVVFNGFYVNEAVGINSTAASATTTASNTGRRQAKERRQYDSFSALVSAHLADLTH
                     "
     gene            200861..201175
                     /gene="pino_cds_118"
     CDS             200861..201175
                     /gene="pino_cds_118"
                     /codon_start=1
                     /product="Hypothetical protein"
                     /translation="MRSLDSVRPRPWSAGWMWMGALATVTGVLGAGTWLALVMAGTAV
                     AARAGPVECRPCTEIASTPAGLVLSGAVIGFVTCALVMPLRSARHDVQTEGIVRVATA
                     AA"
     gene            complement(201471..202712)
                     /gene="pino_cds_119"
     CDS             complement(201471..202712)
                     /gene="pino_cds_119"
                     /codon_start=1
                     /product="Hypothetical protein"
                     /translation="MEQLALPHPVIEHDARATLTSTCPQSIESCAPRKRRALTRSEPT
                     RRRARQRTNNTITTPPSASVYEDQNKTAVAAAAAVVAIKPTVHADRGLAATCPLPMRS
                     RPPRRIHPMARIVQRAVQYVCVDGRQLAVVDVAIALVVQATQGKPGSCDTLLAQSWSL
                     QDSALAVHGMCARPGSVVVLTATVAPPASAPPPFTATAPPRSSERTPLAYTTLGLFWL
                     FGVLRSHGDPRFHDAMGAVLRSGVLDDIGARLELAPVTARASFSCTRAVANIAVVLVD
                     DRCGVPIDASAGNLDNGTNKDNDADLRTTTPDSDNNNNDNDKSDKRNVSVCVLSVAAA
                     AMRNELCGADPWAIMGQGDRRRVVGSDSREACALLAATGACLARRGVAARLLVDAAPG
                     PRVDDARFRHHVEAAFGFVAP"
     gene            complement(205479..206844)
                     /gene="pino_cds_120"
     CDS             complement(join(205479..205658,205843..206844))
                     /gene="pino_cds_120"
                     /codon_start=1
                     /product="Hypothetical protein"
                     /translation="MNGNNAASVGTPAIGNNNNNYYSNNTNTGRPYGSVGASYGAPAY
                     GAPTTAATAYGAPAYAGTTTVLPRYGGPGAYYGTHTPALPTATAAAAPYATVYGGNGT
                     LVGGPVVSPTRPNGNGNGGGPVVSPTTPPTTPTGPVAPPTVPPTTGPVAPPSNLPRRR
                     QRPTWVSLINGMGAAVEARWQSGGLSGTFRVPQSHAHVFMAPAPIDTLRLYALTSDGP
                     PNDPIYDGPRQPAPGIPEPGPALNLLATGCGVFETMLDFYPGSVSGSNGSTITVPPAQ
                     NGAPNGRPPVMPPPPSQRTQNGANGGQPPIMPPPPPAQNGTNNNGPRMMPAQNGNTNA
                     GTPGRPVVGSFGTWRATDIAAAWARTCHGGHCGGGGHPAEVDAVAPYVASVIPPYARG
                     R"
     gene            complement(207460..208671)
                     /gene="pino_cds_121"
     CDS             complement(207460..208671)
                     /gene="pino_cds_121"
                     /codon_start=1
                     /product="F-box domain"
                     /translation="MAVSGSHGIATAAAHDAFQRDDQEAVSVLVLDALPVDVLLEIIS
                     WCRATELRALAETCTVLRDVLGADAVWRTAYMRDWPPCAPTAACLARVDDRALWSQDT
                     TMAASAALHLVKRAPDPRCRHHPPSLVHAHGWRWACMSNLRAPLYRCCTSGRVPCAWG
                     HDAVGDRQQRPMAVVYRSAPVARPSDTRPPFSSQDRQPPPIEAGVAMTMAPAGASWHW
                     GTWERCVGDGPGVCVVHGGNAPGLVFAAHLMAGWPRGPGRLWLPGGAMVEATWVTPLP
                     ASAGSCPVPTGDGRLVTATGDSIWCTWRGLDVPRVSRVVLADGRCLCASTAWRSAIVV
                     MPWLLASTSSLPWFARRGSHLVFWPDERGTPLADTDDGRLLVDCVRSGRLFDPRMAAP
                     HVPSWVLGGTP"
     gene            210639..211348
                     /gene="pino_cds_122"
     CDS             join(210639..210777,210870..211348)
                     /gene="pino_cds_122"
                     /codon_start=1
                     /product="Hypothetical protein"
                     /translation="MLHWPSSQRAPSIEAAPLPPPPSRTPSVFDRWLADTGANPHDLC
                     LPGPKAAGTSLFSFAPATGAAAGGSGDHCDYDHCGRDDHCRSNMSEHDGDKDDDDNDK
                     QEYEQYDPTLYMIDQCDWRKYIETRLGNGITHYNVYNCTAGVPPTPADAEYFAALLRE
                     VVASHPELGAIESLGGCPETFVVLGAMNLDAPPDGAHDAEAQQLE"
     gene            211825..212058
                     /gene="pino_cds_123"
     CDS             211825..212058
                     /gene="pino_cds_123"
                     /codon_start=1
                     /product="Hypothetical protein"
                     /translation="MSPAARPMRHRALGLGAWTLAVVAPATNMWPYAWAAACAVAAAA
                     VVGSLWWCATRRLAPRLRRRRRHLDGPCCCAVQ"
     gene            212530..213069
                     /gene="pino_cds_124"
     CDS             212530..213069
                     /gene="pino_cds_124"
                     /codon_start=1
                     /product="Hypothetical protein"
                     /translation="MANVADEGRALSPAATVAEPAAAPRLRVAVVGGRDFGAYNVLED
                     CLDTLADEHGGIAAIVSGGARGADSLAAEYARHRGIALVEFKPDYDACRTPQERRRAP
                     LLRNADIIAGADMVVAFWDGRSRGTADSIARARRAGLPRRVYDYAGHVRPPTPSSTPT
                     ARTRTITPWTLAAARPPTA"
     gene            213684..214670
                     /gene="pino_cds_125"
     CDS             213684..214670
                     /gene="pino_cds_125"
                     /codon_start=1
                     /product="Hypothetical protein"
                     /translation="MATDTHAVPRPTQPTPLVFPRHLPSLVVEAVFDAYLQTASPPAC
                     VAMACRRRWLAQHTPLAGAPEFCRAARRFARKWLQMVPPTRIVNGVPLVTVWQGCDLC
                     APRLEGAGTLTLVGSRDCGGKNAAACDIVTGMQGQLDRLYVARLSGRTRGSACCNILP
                     DYGTSVHTATLLHDTAADAVDAAVADHKRDPFCKHETAAVLIEACDHYVDSGNADLAD
                     DRRRDVARFADSVAAAKRCGIHVVATVSGASASIGGLCLLRGAVDRFVLVVPDAGRRH
                     DVAHLFAPLTHYPTSTLLALMAVADRYDALVFEHGEAPTETVSVWVRRQKCR"
     gene            215382..217217
                     /gene="pino_cds_126"
     CDS             215382..217217
                     /gene="pino_cds_126"
                     /codon_start=1
                     /product="F-box domain"
                     /translation="MDTDLDVLPDEIVDAILNGASATGHSRLDARWRFAARAVCCRWR
                     AIIDGASRTDMRRIRREVPRAVPRPNTVVHASAVAALARQRTADKLARADADRAVADL
                     WSWANTLRCVCDGEIAAAMLAASTRASIAAALAACMPTNAVDNTGALYCDGTLLRVAA
                     TYCADTQVVATLVDVCGASLTTEAMAACAGTGQCGTVAFMLLRAARSGDRDMLQSMAR
                     TAWLRAAWRDASGRTAALLARMALAPEACVDTLPERAGDFCVLAADDRPASREALAVC
                     EAVKHTADDQTPWWWFTARWGNIRAFTACADRGMTYLPERALMAAAGAGRVSMCAWLT
                     ARDARVLGLADDSGPAADARRRRLALALAVAAAGHGARCAPVLDWLRASLDFVPNTAD
                     AAAILDAALGVTPRAATLFGPLPHAILYALDVWPSAMESAVCAASPSLWHVARDRLTR
                     AVDMGHWNAADALAIVLHRVACVRGDAVGALCDVWASTIAAMVLAAANAPDGLCRLGD
                     LRRVCALALRCADAETVTHPTHDDVIEDQGAWHHVALVDPSWSCAGNHVAWRRCCRPT
                     PVPRALLLGAHDTGAGVAMSPVAGLLVRWLDAVGLVADPEPVTPS"
     gene            complement(217639..220833)
                     /gene="pino_cds_127"
     CDS             complement(join(217639..219023,219104..220473,
                     220802..220833))
                     /gene="pino_cds_127"
                     /codon_start=1
                     /product="Ankyrin repeat"
                     /translation="MAWWHKPGPRMDAAGDADPLSLADMPTEIVALLLSWLPPATVGA
                     CLAASRTFWVLAEGALARQALARTVDGAGGTCPCVALTGKPLVNHAHPRGPRWAARDA
                     DMVSTGARSVQPPLGHARTCLPSALVSAAASGRLDLVHALYARAALLWYGDARCACCV
                     DFRPRSIPSQRQHAHCTTCTAFLDWIFCHDAYDTSLDERPFMAALIGRHPRVALWIAA
                     AGGLPNRVAQDVVDVTCAYARGVTTGADDGATCGTKGDGGDDDVEWDEDSDERLLVTL
                     WCAWPKAAAIAMRRTVRDGDADARESVARLWRHARRGDRWRASGYDCEAMVNAAAMST
                     RPDDALSWVWRHLPEIFLDRPGAHRHMAKAAALAGRIDIAIEAAHAFRVYAGAVLFER
                     TLAMATAGPSVYVRGYADVADNSDGLVDNADGHNVLLDDDNVDGHQVAPCAIVSGAVR
                     SGRADAIDMAMTLWRRCHAGLVHVVAHYPHAWATDRALDAVLHAGRIDLLDRIGVPFR
                     PESARLDRWIESAVRNGHMDTLRWLLTERVEKGQFTGRKVDLAPALVAAADVGRLDMV
                     VLLWPHIPQQKAMRVAEDVTDAAAKSKHAGILAWLVDNVTPPPIHALWTAVAEHADLD
                     LLAILVAHSQYPTSSHASIAVAHGHVACASLLLDLSTAPSDKGASGRGCDRPSGSASS
                     RAPKRHRTSNNAHAPPASARRPTMSPSYVADALARGHMDAVDWAAAGPFRIPWSDVSV
                     GAALNRAHDAFCFDAVVRWATRSSLAHAELSSRLVPDLAVWAAATLASDDPVRIARLL
                     AHCPWQAWDVAPATVARTLCTCPVAMWRTLDARRAIDFDAPCFPDAIAVSGRVDLLVW
                     MTDERRPGERPPPPFGVTHAVTAYNNGHMGATAWILGRLDKVAVADFVARIGDHALYA
                     STRAQILLPLLPRA"
     gene            complement(221081..221386)
                     /gene="pino_cds_128"
     CDS             complement(221081..221386)
                     /gene="pino_cds_128"
                     /codon_start=1
                     /product="Hypothetical protein"
                     /translation="MATPATATTETTCNSTPLRPVPDARLTDQQVEAMRAELWTQWSG
                     HHDVWGIAVGTDDKGRHFIIFDLGYDAQPFPASLPITALGGFEVPLRTCVVGRIVAY"
     gene            221866..222444
                     /gene="pino_cds_129"
     CDS             221866..222444
                     /gene="pino_cds_129"
                     /codon_start=1
                     /product="Hypothetical protein"
                     /translation="MHHHQPATTATATIPMRTNTTATTAPHDARPFLGPAAAGDAPTD
                     MLRIIAKRDLCAATRPLQPVTGGHEPGPIDGARYKRTRQDHGADDEQAQPRQEAKRLR
                     SACAALPKDPHTVTLDAMPPETMRAIIDLIDDRDLHACLLASPCFHVYSRAEMLARRY
                     ALDSGRDIFDSDEPVADIIGLCKRQRRRSRSG"
     gene            222492..223793
                     /gene="pino_cds_130"
     CDS             222492..223793
                     /gene="pino_cds_130"
                     /codon_start=1
                     /product="Ankyrin repeat"
                     /translation="MQAFMPTLRDFTWQASPPSAPGHGRALHRALIAAVDAGHVGPVF
                     EIAGRLGFTDTLVATRLMLHAARTGHLSIVRHLHRGMAHYAVEACKREGGSPTVVSLH
                     AACGRSEWGDNVGHTAWDHGHVDILDWLVENDCPYARRPSPYLLDDAIRRGRVPLARW
                     VASRANSQGISCRRAAVDTAASAGHAEAVRWAHESNLRRCAVSTLEAATVSDRPRCLD
                     ILKWAAGDATRPAAVPEWRDARIALKAAEHGRIEVIQWLAEAHRDCLTPEAARCAARH
                     GHAEIVLFLHKAGVAPLTLCNPLKRTAKSLNAQALSVVADAGAPYDPRALAVAILHKS
                     MPMVTVLCERYTALIDTVEAMRMAGANAACKIARHMVAVLPGACLSHARATVPPHRSA
                     KALGQCPCAACRAAAAAAKETRQPRRSMPTQHPTGACGRTV"
     gene            224489..224995
                     /gene="pino_cds_131"
     CDS             224489..224995
                     /gene="pino_cds_131"
                     /codon_start=1
                     /product="Hypothetical protein"
                     /translation="MASVAGTPVVVAAPAVPVERGLGAAPWIIAGLLLALLVGALVGW
                     LIYRARYEGGGGVVPTPTPTATGFAVQTGVNSIRTNLADEALSQRIADPSVGTYLGSA
                     ATQAACEAGCTSHAGCVQYVYDTNTRPANPLWQAHGCWVRFREPTAAEVVSEPGYITA
                     TRTSAPLA"
     gene            225484..226708
                     /gene="pino_cds_132"
     CDS             join(225484..225829,225948..226708)
                     /gene="pino_cds_132"
                     /codon_start=1
                     /product="Hypothetical protein"
                     /translation="MNTNATTPSAPAPMNRHQRRAQAAIARKVAAEKAAREAKQAQAG
                     HANNGDAVQHDAEQQQDHVAHDEGDANDHANGQNQGHESKHRHQHHHQNNNNNNTKAA
                     RQGARKAAFRAAVPRPAALGIIRDAAGGWWRWAENAPEPVFHADAKFTRVVPRKPVPR
                     GRPRPAAGEGAATTDGDTSANNNNNNNNGTHAAKDERQKAVRAAREAEAQAFKGALKQ
                     ALRALMTDYCQAHFDGAWWYTDLADTLAHRLRVGQGRFDAELAHINVLRNSCRGWWTW
                     VDGTTTGPVFSEDAPKAPRAAKKPAPRRPVAPDARHHHAPQTCPRPAQAASGRRRRAR
                     SSRARPMISAALPRTQCLIFPAFCTVEKKRPAKK"
     gene            227536..227970
                     /gene="pino_cds_133"
     CDS             227536..227970
                     /gene="pino_cds_133"
                     /codon_start=1
                     /product="Hypothetical protein"
                     /translation="MTPRRARDPTRQTSKDPGETGKSTKLDYERQRHQQHPRPLPAFR
                     RLGRRVATMLVTNQLASTSIAVRAVRVRDLPRPASILGPGAQFHTCATPTAWWCGSRS
                     PPSAGRRWTSPTTRCRALPNSARTTWPFDIRVTPQGVRLDNA"
     gene            complement(228551..229924)
                     /gene="pino_cds_134"
     CDS             complement(228551..229924)
                     /gene="pino_cds_134"
                     /codon_start=1
                     /product="Hypothetical protein"
                     /translation="MNVDDASSLHAVQDAICGDRPHASAARIHDEIERARLIAGSLTS
                     GTVRQWLAHIRHASPDQVLAGEYRALWSALASNRNASEAMGNRPTPTSVEVSAEYMAR
                     DTCASAHMAALNELATSDPESAAAQLVEALGASARCGAIAPPTSPDRARTPTHFVLTS
                     QKDGMIRAALFRLPDADGGIARVVLCIDYSLQSAQVDGHMVKSSLRRKSCVGCSYLEF
                     VSLLLHAFAERADLACHSALRPEARAASAWFTRSLTRLSSPHDVPRAVRSYIDKPHES
                     YRWRGAFQGEWMGGVLHGAWVDEARIENLVCLLAGRQCADRASSLFVAPRSLLDRALA
                     AYRGPLRVGVLPSDVLARAAARAWHRVCTETPLPSGRLPHGDTLLDIARAFGVEPTPA
                     QLERPELLCARLAEPAVVEMVRSRYGLDPVRGPVAFTGPHHYARLWASLCDCAPGTRP
                     DAEPCAL"
     gene            complement(230689..230982)
                     /gene="pino_cds_135"
     CDS             complement(230689..230982)
                     /gene="pino_cds_135"
                     /codon_start=1
                     /product="Hypothetical protein"
                     /translation="MTDVDTILTYKRESRNLQGTITITSADTDDKGYSLCVIHGETQP
                     RPGTGVFSAVPSTLLSFAVHAGVEKRATIQQVGPALKAMGLSVTSIMDGLLIF"
     gene            complement(231285..232613)
                     /gene="pino_cds_136"
     CDS             complement(231285..232613)
                     /gene="pino_cds_136"
                     /codon_start=1
                     /product="F-box domain"
                     /translation="MACERRHVVDGSRAINNEDKCVNDESRSTTMTTTTDVFGLLPDE
                     MVRAVLSWLSGADLARVSCASVRLRDLIDDPTLWEGLCRASLAVPDVRPLDDRHADVG
                     PRADLWLDNIVSLDKHPCPPPLPELPPPTLASADPQRKPWRWLYAACHRRVVCPTARP
                     LGGPSWWTRWRPWQVARRPGHVVRGERIGTAALIVIEVGDLDDQGRLAGFGLRATCAR
                     PSDAAPWRWAEWAWGTWHGGRIQGLGRVCAASGAVHTGRFMDGIAHGRGIRTMPAQCR
                     HTINAATASINTSRSNIDVQEHPTPCHAVEVGGCWRAGKAHGRLVQTTSCGDVWVSLW
                     NRGVLAGIESLLLPPRPATGVRPAFGGVRIEGVSWRVEDVASAVRGAAISRGLFGRCV
                     IAVPADAKALDIYLDYVRAGHPCLSADMAAAVLGAGTRIRAALSTRAGSL"
     gene            complement(232986..234491)
                     /gene="pino_cds_137"
     CDS             complement(232986..234491)
                     /gene="pino_cds_137"
                     /codon_start=1
                     /product="Ankyrin repeat"
                     /translation="MQKKKHKGHKEHKGHKEKTSNMASIDTLPAEIIALVLARLPRDR
                     DFCRARMAHRCFRVDSDKTLADRARRWHGEPNPERFCAVGLVEALEALHARGVAMGPG
                     CLVAAASAGHVDVLAFLQRINVPFDVALPPIGTRDIRCPIPTSLRKVTLAAISPSQRT
                     MTAQVNAYVRSSLIDRAAAGGHLAAVKWLSNIPGLKATTVAMDWAAAGGHTDVVAWLH
                     ENRTEGCTKGAMDMAAASGHLSTVAWLHENRTEGCTATAMDAAAAGGHLDIVRFLHER
                     RSEGCTTDAIDCAAIGGHLAVVRFLCRNRTEGFTRAATDEAAAEGHEHVAACLRAQMD
                     AASDRDDAEVFRPRPAAIADDDKGDECARTDKRKLPASIKRAVIKGDIAALEQACERD
                     TVLLGLRPSQGRKLWTWAALGGHIDVARWLDAHSVRGYDDVTTGVALGLGNLDFVRWL
                     WDRAVGAPSATRPSMGLRQMTPTLLRRPTTAWLPPLLLWASTTWREICART"
     gene            234683..235364
                     /gene="pino_cds_138"
     CDS             join(234683..234945,235106..235364)
                     /gene="pino_cds_138"
                     /codon_start=1
                     /product="Hypothetical protein"
                     /translation="MDDDYAQPEDLMPPSAALLDQVRDELGPWRNHSAVHAVIVDGTR
                     ALCASPLRYFPPSPPSATCPRPCGSAPCVVAAWPSTTARRPTTPAPTSLRVLDQRCRS
                     ASASPSTPRQCQSPLMATTANPIAPAPSTGTSGDPTLFTVVVAHANDNDDGGDVKVKP
                     IGTEPVPTAPTDF"
     gene            235640..235999
                     /gene="pino_cds_139"
     CDS             235640..235999
                     /gene="pino_cds_139"
                     /codon_start=1
                     /product="Hypothetical protein"
                     /translation="MDPAKTPRAPTREEQVEDLLRRLSPSCWTSPRDREDAIYALRTH
                     THAVAAFFSILFCYWLYPDILLSPVFFDMRRSLRLSFFFVVGRHGMSDAIMASIREAL
                     ARSDADRPCDLKRKKDD"
     gene            complement(236364..236765)
                     /gene="pino_cds_140"
     CDS             complement(236364..236765)
                     /gene="pino_cds_140"
                     /codon_start=1
                     /product="Hypothetical protein"
                     /translation="MAATRLLGEAIAINGPVQMRAESVERVRSMLALVCALAHRTHPL
                     DGWSTCEGLAHVERQVPPLGDVTPNAWARWCRIRPLDPPPHVIARFARHHGDDTNNAH
                     RLLLLLDLLEPCGDHGAARRNLQALVVVGAK"
     gene            complement(236802..238168)
                     /gene="pino_cds_141"
     CDS             complement(join(236802..237840,237945..238168))
                     /gene="pino_cds_141"
                     /codon_start=1
                     /product="Hypothetical protein"
                     /translation="MGKWGREGRCPQKWLISCSTATRVTGAPFCLDSGDSAPPWCVAH
                     GTLVCARRHHRLPVRSSTPLRRARAYLATSGIEVLCAALLASGRADAVAYVTSHIIPH
                     MSGGFINQQARPWFLEKDSASTMTPCALRTRQCFYPCQIPPEQSGTICMRTGPAVTRA
                     VCRAGRTGGLTVALSLCAPGDWSTYFDCWIDDTARRDHADVLIDLLRRAKCPKGAGLE
                     GTAWQSAVTRAWGAAGEHAAMKSLASLHSFNVSLPHENRTEMRWLWVDRAVLADAVCV
                     LDWWERTCVMPPSPQRLDNWLVRALACGATASADWLAVRLGACDMRALAAQFACCIGD
                     GGAERWADSIAWLERYMPINTVLATVASAFFAHHSVWMHRVVPIVERWPVAGLDALGT
                     LIVRAARAALSMGYWRLIDRLVTAVDRA"
     gene            238347..239081
                     /gene="pino_cds_142"
     CDS             238347..239081
                     /gene="pino_cds_142"
                     /codon_start=1
                     /product="Hypothetical protein"
                     /translation="MPLIFVPRHRVVASPSPHLENKSTTVDFAASTNGTGGVGAKTVG
                     NGTAREHWLRPPKHATTDRPPTEAAAYTWPRSTKAPDQRNRHEHQHRGQPARPSGRRD
                     RQPGERRVLAVHRSLTYTKDGSTASGWWAIEDGHMQGTLRALDGSRFHGTVDARTGFD
                     GTLDMNGAGCSWLFCSHAAFACCDVCHEGGWCVGPPGGAYAQVASMQGRWDRFGTGAG
                     VITLVDGTSIHAAWRDWFSLVDPLSP"
     gene            239561..240361
                     /gene="pino_cds_143"
     CDS             239561..240361
                     /gene="pino_cds_143"
                     /codon_start=1
                     /product="Hypothetical protein"
                     /translation="MWKPVGSHMPRLATVSCGEVLASVATHSVALAVPMAGRTRLLVT
                     ALYSPLEADGKQDNDTNSTNRLDAPGVAMDPMTAHTWWPADVDQWLAVSTAFHSATTR
                     ELGCGDAISRRLVRPHVALAGLYEAAGWGMGRAGGRLMALAAEVRTEGSPPSSCDLVD
                     DNDDSANAVHIVHAGRHAMGVPFAEWRAFCRLAAQRDAALRRAMDVDPTVYGARGSFV
                     VTSMLRTEEALARRWRHHPALPDPDTIAGLRRSFMAIADARRHTAGSL"
     gene            complement(240623..241401)
                     /gene="pino_cds_144"
     CDS             complement(join(240623..241177,241276..241401))
                     /gene="pino_cds_144"
                     /codon_start=1
                     /product="Hypothetical protein"
                     /translation="MARRAAMTVAKALAPCSEMTSVGASIIGTTRDGRLAQVSLTADG
                     PHKQAITCHTWWPRRTSLCVAMAPVCAIVGLDTLSDFNLADLSKPLEGALFAYKAHGR
                     AVTAMATRYGAVPVAMLAETSEDQAIHRLPWMYSAGAACLAHLEATGAATERGRFHKT
                     TLARDAALRAALIDDHDPYSPQGAEIMARVLRRIQDVYVCHGQAAMAECIDDLSRQLF
                     LSPHLRRA"
     gene            242066..243334
                     /gene="pino_cds_145"
     CDS             242066..243334
                     /gene="pino_cds_145"
                     /codon_start=1
                     /product="Hypothetical protein"
                     /translation="MQVTQEPTSAAPAASRASVSQEQRQRQQQAALQFVRLFAQTLAE
                     GTADAIERAEHLCASAPALCGIAWPVLATIFPEAADLFVGPAEPGAPPRALAPRDAIA
                     WARWHEAQGLFAAGQFDLLEQYCVSSSGRRQMCDTPGLGWVEAYVTDFDSAAEPFFGP
                     GGLDDTLAQLLLWRRQQAERRWRALYALGVAYDEWLAEAEDRGGVTDIQAEPSAIAMV
                     DAPENDLQLRLRGLLGTSRVVDVVPLGASAPAPNRVSPIPVMNAPIDRWTPESVANGQ
                     AFASVPGGTDAVIKAVADTIADAELRGLELAVDTALRRQRQVRADASIGAATAAQVEE
                     ADRVVQERMAARDEAVAAAAEPGGSADIGTAFPGTGVYLMNVFVGDALIPALGVIPAA
                     PLALPPPDCPQCIERMAMGLPPIVGRHPPH"
     gene            complement(243371..244235)
                     /gene="pino_cds_146"
     CDS             complement(join(243371..243396,243530..244235))
                     /gene="pino_cds_146"
                     /codon_start=1
                     /product="Hypothetical protein"
                     /translation="MDRSQRGVTPPRAMWPARAGAPVIPIRGVARQRVGPRPTPLGPP
                     MGRAGDVVGRAVGDGVGSPSTPLPIYAEAVGPLKAIGYVEEGRLTAAQIDRVRRAGNA
                     TDGKYVWNNDALARLLALNTHLFGGNIDDFVRMVRGRQPIDDPHLLCLIHAAFLDHRA
                     YAAQPCTASALLLPEPVGAYMDAVLAHGAALSPEDAWVVPLVAAPAQWPRLFALLNNL
                     QRQADDCDGRFMGGPLFQQSQTGLP"
     gene            245070..246056
                     /gene="pino_cds_147"
     CDS             245070..246056
                     /gene="pino_cds_147"
                     /codon_start=1
                     /product="Hypothetical protein"
                     /translation="MENKTEDIKKTSSRPDGADADAVAPSWMAHCPSDDPIASILDAL
                     PATRVLGAFVHAPPGPALVPPLFCLCLRAVVPFAVVDAMLQDRYRVVVTHYDRRTRLE
                     SVGSNDEDSINNDNDGACPDSLRPIPRPLPWPSPWASYCACRPCCYPYEALGGSDVMP
                     CPRGRLVAIPPRTALRIAAWQWASAVTGCVNAPLRWPSAVDVGPCPAPEPQQAAAVDA
                     YARDKGAVVRIRPDGLALIGRWPRSAGTGGDGGNTHGDARPFQHYGTPVRRVTHPWDA
                     RVHVDIYDRTCPIVDEDKDQAYMRRHLPYAVARVDTDSVFLVAIYKTTIMTL"
     gene            complement(246534..247565)
                     /gene="pino_cds_148"
     CDS             complement(246534..247565)
                     /gene="pino_cds_148"
                     /codon_start=1
                     /product="Hypothetical protein"
                     /translation="MNTGGINRDQPAAPDLDALLARYQRAPLDAIERLIITAVSDDDV
                     NEAANAIASLCRITGEDDPHACGALSNTVINAATASDDGGRNDIETALTILCANLLGL
                     DAVCAVQRDPETWTRRYPALAARRTPTPLQTLVDVAFALRRIHAARRCALYALYTSVA
                     ALGSVSHPLPPYSAVSLRDLERWARRWQTGGAGTEPAVPPLPLVGPIGEMGPARERMF
                     PVIADVTEAMPMTHNDIVALAYTEGPASLYGRLAETRGQVATSLANAVSRELASRLAT
                     AVLATESSAAVPRACLDSAINIFLVYPGTRPAVARHFRTDPNDKIDLGVLLRLPSEKA
                     EEDEWGSLQ"
     gene            248437..249651
                     /gene="pino_cds_149"
     CDS             248437..249651
                     /gene="pino_cds_149"
                     /codon_start=1
                     /product="Hypothetical protein"
                     /translation="MEIFAPGLESTLAEPRRVGGGSWGGSGGDARPAVVPGSSMADRS
                     RRDGMHDAPTRDGGGEDGASHTRGNGADDENQVATLIARVDPGRLRQATLLGRCFEVL
                     VPVERLCVAFARQTREAPWSDCSPLAEALRAHMGFADASKALQAAVARVPLGGTDTPA
                     YVACCGCSRPWCSGRPCATGWMRPVDPRACRRLLAWQWAEHGVRGLNPDAAFGGFYER
                     ELVQCVEPDAGHRLASCRRRRDAADDPPIAATASKRHCRRVPSLQAGAGTDANTDNAD
                     TGGKLAHDHNAYGDMIDGTVGLDGTDYDNDGGGDSHPLLQEHIHGPLLLSCGPRCVIT
                     IRDAAVYAGALGASCRRGLDEMVHPSQAALRGRVASGTGAVERWVRVVGDAFIVRVHL
                     PAHDAIPWDAHG"
     gene            complement(250018..251102)
                     /gene="pino_cds_150"
     CDS             complement(join(250018..250806,250911..251102))
                     /gene="pino_cds_150"
                     /codon_start=1
                     /product="Hypothetical protein"
                     /translation="MASSLELLLWHCPPVVDALAARLPLAAMAALGATSSALRDVMHG
                     PGVLGKRLMTEGAFCFRSLKCPMVALPRPVGRCMGARARWRLPLPPDLFAEGVLAGAA
                     CGDARLVARCIGSVTEYMCRADDYAHALDAVTALNDRAWRESDLARAAPIVVHVTTLL
                     LARAHALATGDRIGGSISMAEAMYDPKDDDMGRLYQCSACHIGAGLERIEAEALRRLF
                     GAFCNDLPARSPEACILAIHIVGLFTGHHRDAAPYPNLREGAVAALVRACTAAVPGAR
                     DRDPRWYAMLARVATDLTHLAGASVGLCGGPTAPWFDGMDVCASSACSIP"
     gene            251958..252994
                     /gene="pino_cds_151"
     CDS             join(251958..252614,252695..252994)
                     /gene="pino_cds_151"
                     /codon_start=1
                     /product="Hypothetical protein"
                     /translation="MNVGDIIVPLVDATTGRPRGTIALPCLDADDGTRYFFGPLLVRA
                     SGVKRAQSLYRKIRHMATRRIATDAEMTIVRRRRPDLWEAVMHERHTQVEQEANKDTR
                     RRLRRRRRAAERAIAASQQVDPSPPPVFVTPPRSLVLGKRTRVAPRRIVFRCAAGKND
                     DDDARPLAGDGDAAEIRAETHDLHNDKDDDHDDRDEKEEDDDGDDGDYDADGAGDLVP
                     LATSRIVEFLGSDIAAPDDAISNYNDKNVLTLVSSQRGVKRTWEDILPAVAAWAAARD
                     AVLVTWTGDGDKDARLGTRPYQRRRHRRKQERPMRALQPLVS"
     gene            complement(253582..255075)
                     /gene="pino_cds_152"
     CDS             complement(253582..255075)
                     /gene="pino_cds_152"
                     /codon_start=1
                     /product="Hypothetical protein"
                     /translation="MRRSNQPTLTRPAAARPPARTATAGPPQALTGWDSVRQWAADNG
                     LDSAEAVRQWINEMAPRGLYSGIDPRAAALLQTLQQFSIASSPGQPAHEIQGLPSAYG
                     KFLEALSSPRVGSTTSQARGRYYPPGIDGPEWIRPLVDPLAEGGRLWPPSPVTALVRM
                     NEVAHRVFGYDPVDSDTMIFTMATNNWTRGAPDVADRVAEVLTFFGDGINNVLWNLGG
                     VRADPTAPKLASDADIAEARQRWPAVSPYGDVQAPYIFVIGTGAPGHLYGQTPITGPT
                     HVALITQGPLIIGRLIVSDATGDVIYTSGVFLPRDPSVYMPAGGLWLDKTSGGNPQLL
                     VDLMLPFIRAVRDGRPNAVSMSSTGITRGHRLSYDHEGVTGPYVVRAFEPAEVLAALS
                     VGRRAAAAEAIDEATASASAGGLASLAARAYRGNIATANVPDEVRQLIAAQAMARACG
                     PGATPQDRARVADAARVLGVPDAVRRQDLATICDASADAVRRLYGRP"
     gene            255650..256015
                     /gene="pino_cds_153"
     CDS             255650..256015
                     /gene="pino_cds_153"
                     /codon_start=1
                     /product="Hypothetical protein"
                     /translation="MNDKREQPTAPAALWDGSVMCDDALDTLFHVLASADVASAAAAR
                     LVCRRWGEIGARALVAVRRQMAASAAAPSGRCGHFYTAQAEITCALALDSHPRLMRAL
                     DADRAGVDDPIHLFRLNHL"
     gene            256049..256525
                     /gene="pino_cds_154"
     CDS             256049..256525
                     /gene="pino_cds_154"
                     /codon_start=1
                     /product="Hypothetical protein"
                     /translation="MEKRVHPRAAFDSHVPCDIGVESHAGVPPAELAVFYGAVGCLDL
                     LIERGARIDPTRMDGLLSRFLASCPWVDARVCVGSACRDPFGICVAHGAAGLVRCIDP
                     IPIVQRLLATLPPYPAGTQRIPATRSTRLYRWPRTLRSRMHPWCPCSCHRVTRSGH"
     gene            256555..256818
                     /gene="pino_cds_155"
     CDS             256555..256818
                     /gene="pino_cds_155"
                     /codon_start=1
                     /product="Hypothetical protein"
                     /translation="MDTMHARGFGLTNSDGVAFNMTLTMDKVVALSTATVHEIVHAIG
                     CNNDLVGAILDRAPGSGRGVALDALSHVEIVHNFSSAARSSRA"
     gene            complement(257505..259103)
                     /gene="pino_cds_156"
     CDS             complement(join(257505..258589,258992..259103))
                     /gene="pino_cds_156"
                     /codon_start=1
                     /product="Uracil-dna glycosylase"
                     /translation="MTLSLFVDAPSGGPTDLSLVLARTRPPPVFRRTIAPPPRNGQVY
                     RNTQAPGAGQGRRGFGVGDHDNHSRVQKGADRRRIVIAIVVVVVFSRDDEYPLSVHAA
                     WLFDQLPAHWKPLLREACAHYTFGRVAEFLRCEIGHGRVFYPPMHQVFEALRRCRVAT
                     APQSDGGDALSETGPADVAVVILGQDPYIHERQAHGMSFSVQPGTPTPPSLVNVFAEI
                     RNDLAVLTRPIDFAPTTGCLVGWARQGVLLLNTCLTVEAGHPGSHRDRGWEPFTDAVI
                     SLVSKRSPHPVVFMLWGRDAQSKRKLIDQNRHKILEASHPSPKSATNGFFGCRHFSQA
                     NAFLTKNRRPRIDWTDLSLDPPPAAVVAATTTAAAAVSDAAPPIADPPTSPKDTKDEE
                     KDQHHN"
     gene            259200..263613
                     /gene="pino_cds_157"
     CDS             join(259200..259254,259314..262843,262915..263613)
                     /gene="pino_cds_157"
                     /codon_start=1
                     /product="Ribonucleotide-diphosphate reductase large
                     chain"
                     /translation="MEYSQQQHQQQPHRQSDLLLDRLDRERARLADGQTGGAGPPGPA
                     ARSRPRSRHCRAPRHRGSCAGMTPEQGDTLLAETAVALSSSHLDYEKLAARVAVTALH
                     ASTPPRFSDAVALLAANVERRTGRPVPLVSDELVAFVAEHKEVLDAAICHGRDFGYSY
                     FGLATLRRSYLTKVRDIVVERPQYMLMRVALGHYGFADVDNALEHVLATYEAMSQQLF
                     TSATPTLFNAGTPRPQNSSCFLLDMKADSIEGIYDTLKQCALISKAAGGIGFAAHKVR
                     AAGAYVAGTNGQSNGLVPMLRVFNDTARYVDQCFSGDTLVLTAERGPVPIGLLYREAA
                     IDIATGPASDGSDHAPETSIVPTTGVRVLSDDGRWCPLRSVVRHAPSTRPTYLIGAGS
                     TFVPHWATPPVDALRFGHGVRVTPQHQVQVLCDLLDDGRPVVIANDPIDANDPIDAQD
                     TANAEDSGGDHTATNAEEGPAKPTTTFDAATKMNKAAIAKAKREANEDGESATGKNVN
                     ENDDEDDDDGNNSRDEETGADTAQKEAKRRSADVLGLRLVSLCERINRGHVAPRMVDA
                     EQLVPGAVLCVPIPPEPTESPWVRTKPSDWRMAGILHAAIVHGAVQIGARVDTRTAIF
                     INTYLAIADPDCASASRPKNGICRWRIAHPGFTMARSLAAREVLASAPTLAIEAFVRG
                     VCEGLDGSGASPFLVDTLRWLSLRLTRRAASFSPNADAIAAGKAAILSCRTTPSPLST
                     APTTDGTPRTAVLPWSIVHGDIVHVPIESIDTIVHDTHDTNDVGSADPNNVESDGNVD
                     NTHIANVTKPVGTSGAEGDDGALYDLEVDDPNHTYSVLGFGACHNGGGKRKGAFACYL
                     EPWHADLINWLDLKKNHGKGGGEPGARDLFYGLWTCDLFMRRMVTGRDWSLFCPSEAP
                     GLHECYGAAFDALYERYEREDRARTVVPARQIWSAIVAAHIETGTPYMVYKDAVNLTS
                     NQQNLGTTTEANLCTEIVQFSSPTEASVCNLSSVALPKFVIPDPRGEDRSGPLDSRYD
                     DAGVALGMVFDHGALHEVVQAMARNLNRVIDINHYPVPEARHSNLRHRPMGVGVQGLA
                     DVFALMDLPWESAGARRLNRAIFETVYHAAATASAQLAAQEGPYPSYYEGTYIDGQGN
                     EVRGSPASRGLLHPDLWAAAVNDQRRWAGCDHEEGDPPEKGIPLRPSCAHGLRNSLLV
                     ALMPTATTSQILGNTEAFEVITSNIYTRRVLSGDFTVVNRHLVKRMVARGLWTPDFRD
                     RLIAAQGSVQGFDDEEVPPDLKALFKTVWEVPNRVTIDMAADRAPYVDQSQSLNLYLA
                     DPTAESITSMHAYAWRRGLKTGMYYLRTRPAANAIQFTVDQAMASAAAAVVATDAVAG
                     DATLAKDVSTESPKVDIARETPRSLRKAAAAITQTDGDACYPGCESCSG"
     gene            complement(264454..264867)
                     /gene="pino_cds_158"
     CDS             complement(264454..264867)
                     /gene="pino_cds_158"
                     /codon_start=1
                     /product="Hypothetical protein"
                     /translation="MSKPRLSRAAISDDDYAAVLGKVFVHTTSYAADAYQVVGRTKCY
                     VRAIHVPLVSAHVALYGDGAHKIDWAKVVQPAPGSNSKKGNLYSLVRAGDDDSDGDGG
                     DVSDQPTYWLTKVGDDFHAFPIETGSDHVFTTVGY"
     gene            complement(265177..266073)
                     /gene="pino_cds_159"
     CDS             complement(265177..266073)
                     /gene="pino_cds_159"
                     /codon_start=1
                     /product="Hypothetical protein"
                     /translation="MSHTETAPTGQVVQALTAADPSVGHLMRAHGLDTLDQVFRFVPH
                     EACTAALSTALAAGDAEILGEIVAAFGRHSLDKAIDDLSHDPSAVQRICEASRRRDDT
                     PDNHQCYYEGDTLISSLAIKEHVESLAIVIRHSADWRSSIKRSIRDLALDRRHLRPNL
                     HALRILLDACANEPKIDPETRTRFLADALAYCADGFAYDIIDTLAPMCTPHVLHETVL
                     RCAGDGVSFYAFDNIWPIAEGAVCVHRVAKDLGGGRVRVHMRRCIRDLGKGRPCKSVD
                     CIYGPSHTSDRPEPPCQDAPGH"
     gene            complement(267049..267900)
                     /gene="pino_cds_160"
     CDS             complement(267049..267900)
                     /gene="pino_cds_160"
                     /codon_start=1
                     /product="F-box domain"
                     /translation="MDALPGEIALAILEHLPPSALASVARVSWRYNRLAMDDALWRRH
                     YEARCPPCTDPHIGWTCMAHKGRGLDHRPWLDVSCVDEDNHFLARPYASHPLCPFIGT
                     MPAARDSAFHTFAHPPVYECPHHCPSVIEARSYRWAFASQCAPPRPIDSTDVRVGSGP
                     WCVGHDLVHHEGFAILSRATTAYRGEWHAHDDKPHGVGMFAMRQSETTYTGAKDVRAV
                     AMWRTGHYGGFVRHWERTHVMDPHASSHPNYREGRCRSDGWTVQFSGAIAESKTGTIG
                     TTVSDRL"
     gene            268370..269509
                     /gene="pino_cds_161"
     CDS             268370..269509
                     /gene="pino_cds_161"
                     /codon_start=1
                     /product="Hypothetical protein"
                     /translation="MASVPLPKTGALGDRVVAPNKAPPTVPEPRLLLRKRLDERARAR
                     ADRETASQREHDTDMIERRVAAAWPVDGPRRVKVNDAQAVRMESRAALVDLLATLAWS
                     MPTHLVVVLDDVDVIFIAGGELYFAYRLARVLPAARKIVAAQDLARLGSLCAFVEYVL
                     YLPCAMGRHRIFLSDGVTEVECRVRSILLRFILTVLDAVRAYLAAPKAPSAKGMTQAS
                     GSPVCMPCPVPGGTSWPRVFAAHLLGSGARTVTLSGITYEKEAVDVWVRRAAYAVALL
                     PAAAADRLADDYCMWGGPIAVGASFVRDPVIDWAVDLYLCVRIEGACLWAIERALTEA
                     IACCPPAYLMHGAFRAMLDCVGLVEASPRIRHHVIAPTVLTNMVA"
     gene            270176..271294
                     /gene="pino_cds_162"
     CDS             270176..271294
                     /gene="pino_cds_162"
                     /codon_start=1
                     /product="Hypothetical protein"
                     /translation="MLKRRLDIVAVSADDLQCTRKKARPDDPGFNLGPDTLPEPTRDL
                     DDQDSDAFQRRRNLIARAAASEDRDSLRLLLDGARDKDIKSAIYRVIDDSIAVEYVCE
                     TLAEAGRHLVLATKAIYRAASRLHDDTAFVLVTRVGGSRRRASITGALTRLVEDDEPE
                     PIACLFGACARAGSRVSQERRHMWARDALCVAVKNGRPASAEALVHECALDDMVHLIE
                     RFVAADDGDSVAGLLDACDGIVPRPTWTKWATSALRRAAMADKVGVIESLLDMCEPDA
                     VREALLCCADERHRSETFEALWEHADACAHDLVEQLPRGVGRDFLYDLIDEGGGCEGY
                     CEVLVDGVDEDQSNTDTDDDDDPNGDAFTSILSRHRRR"
     gene            272609..273611
                     /gene="pino_cds_163"
     CDS             join(272609..273468,273578..273611)
                     /gene="pino_cds_163"
                     /codon_start=1
                     /product="Hypothetical protein"
                     /translation="MFKRLLDECQRRRDIDTETPAAKRARHDADDAGTGLGNRSEWGI
                     NFSETHVRLLATTALAKYDKTLARVIPGASDKQIKQAIYRVISKPAAVARICDAANGR
                     IDRPDEVLQEAASQCVHGTVAILVDRVCSEFEITCVLARLIKQGDREGVLAIVDACES
                     SPEISPVVYKRIVEASLGKAAKRGTINIINDLAPRCDGQALRRVLIHCASRNYGIEGF
                     EAVWEHADLCAHDLGDDIEPGAARDFLHDAIHKGDGCRGTCPASRPDNAPASPRRRTG
                     DRTPAISRRSLCLRKLGVRVF"
     gene            274394..275383
                     /gene="pino_cds_164"
     CDS             join(274394..274980,275083..275383)
                     /gene="pino_cds_164"
                     /codon_start=1
                     /product="Hypothetical protein"
                     /translation="MTTLGKRPLEPLFGNVNKHANDDDDDDDIATPSPKRPCYEPPTG
                     PQGIAVGDLWNGVPCDEEDLPPCDAVVKAAMKADASVDDLVAALVEPTTYQVELAMIQ
                     LFGQGFAFGLLCEVIVGPSASLHQVAVDALLHRAASDNHPGAVGTLITHLAKEDDVER
                     ALATAVEEDDADAVKMIIKTYESDANTPDDSCRRVNAQPTTTTPRTVFAALWRHADLC
                     AHAYGKSLLPCPALDYLKEFVASGEPCADGCMSNVSDDSDSETETDDDGDDDRDDEND
                     DSNNGGGGEADRPDYHRPL"
     gene            complement(275862..276853)
                     /gene="pino_cds_165"
     CDS             complement(join(275862..275904,276012..276853))
                     /gene="pino_cds_165"
                     /codon_start=1
                     /product="Hypothetical protein"
                     /translation="MATVTPLLSDSTAPVASTHATPTAGTVATPAPVRAAESDPSPVS
                     PASVAKSMTITDLCKFLRALPDPKAPIHGASEIKTMPITFALVDGTVTQAEAWGDSFN
                     IADFKPKYIDHNLAISFNGDSVPADVLLAAMEPCLAKHAHVRVCVNRRRAVVGKPPAV
                     AIPVDCLPFKKDPTTMADLAMTREALRVAESLAAMGTYDGRVIAATGSTLMPRHWEET
                     RFFVDGTLLQSLSECLPNLHNHDMLTLFVAVGPTHDEAAEKGHGRLIIRMPIPGLTYA
                     MLVDACVSCAYASVPALL"
     gene            complement(277265..278128)
                     /gene="pino_cds_166"
     CDS             complement(277265..278128)
                     /gene="pino_cds_166"
                     /codon_start=1
                     /product="Hypothetical protein"
                     /translation="MLPSNSIHALPVPQPFGAPQGRREPGTRCAVGYHYDVHRRCVPD
                     ASTCRDNGDGTTTCCTTTVNVPPENGRGDAVQRAHALPVAPTVGASWGVLGATAPNHP
                     QCARPGWVYDMRMADCVPPGSVRCYGEPASGQQCCAPTAGNRFRCCDQTLGGVPRCSD
                     LPMGGGGGGDDPGTGQDGGGGSGDGGDPDPKAPGGYGARPMFGSPAVFGATAPSAVGA
                     GPGFNCRPTYSTASGSLTLTCEPQRGSRGEMPGYPQPDSVAFCNQPTYMGGHPLPPVR
                     TALSIQSLTIN"
     gene            complement(278749..279684)
                     /gene="pino_cds_167"
     CDS             complement(278749..279684)
                     /gene="pino_cds_167"
                     /codon_start=1
                     /product="Hypothetical protein"
                     /translation="MQCSLSLCPSFLHKTPAPKRPRETDGGPRRDRATLKRRRTAATA
                     PIIVSSNRPGHASLTRGRVPARDATPQGHPLVDRQPSASIAQACDHVRRVSILLDYLG
                     TLVADGQCEAFGVDINGVRPCYLTVRRGLPCGSGLVWTLQSSWSDVCNVTNTVDTATG
                     ALNAAAGGRTVRSAPYPSGKLSAIVDTRLPAGISSDTGSVSVICRRWAEMRDTAGAAY
                     GGSRLWVGMNSFNRGHSAASMRALGVLVTGTDHQWVHPGPLAIVEVPLDQAWDNWNDS
                     AIRLAAEYLQSLSFALAAQLSACAPLPTARCLLAS"
     gene            280628..282739
                     /gene="pino_cds_168"
     CDS             280628..282739
                     /gene="pino_cds_168"
                     /codon_start=1
                     /product="Hypothetical protein"
                     /translation="MSKEQETATLGCPINDLPVEILAFILNGHVRSPRRASEGADMFT
                     LYGDARDHWVDRPLLDGRWRFAARAVCRAWRVLIENPSLADAAALNAYQPFASYSPLR
                     LQKCPKWATGRIVCLSAAADIVAAHSAQWAQDPEAVFDWCHHNAGATRKQAVATLVAS
                     GVPWAVDAIVNHHWPLISFPVQGDHLSVGYDPTAAAMLGPAAQCSRRAGTDLQYAVAC
                     GDEGCMTVIGNGDNECAQTARNRPCGWGVDLSAVAVHPAVAEDFIFYAEWRDDCGDVA
                     GFLWILLAIALRRGSHTTFCAIKHGFACHMSEYDAVMHAVAGNQPDLVTTLLGDCTEV
                     DDKLWDGAAGASDPACLGRLLDLLAPPAPEPGDKAAEWVVRAIRCDRPGAFALCDARG
                     IAFDAAEVANMAARCGSVGVLAHMVARGQRLFRVSPTTAVPLDLGALAWEAMTDADGY
                     SSDTRTIAWLCDVAGYAPRRGTDDLRRLIEHAYQDDNDGGGPERIVYVAERWPDAFAA
                     LPKLILCGAFGHCVGQARKPYEATARLARVLDTHTNPIDRDVVVQDLDLWDVVVSNLT
                     VEFMRTFRAGSLIRTLCLLARGERPYAEDTGAARHPPEPCGCTRGPSWRHRGVSPARS
                     VCAKDPADHGGPCDDATLAMLAPLGRWCVARPIHIGTIFPGWVAPTHEPTHPYTMTAI
                     SDLGRSHLVAWLDVHGFLLAE"
     gene            complement(283101..283910)
                     /gene="pino_cds_169"
     CDS             complement(283101..283910)
                     /gene="pino_cds_169"
                     /codon_start=1
                     /product="Alpha/beta hydrolase"
                     /translation="MDLWEEMITPIEAPCNPFDGASYCTSYETLASLRPVCMAGTYED
                     QYGRTDMERLENDRIVLVHAPRSLLEATKTKVPLVLFFHGLGSHPWKTALYGTGWRRL
                     AHQHGFIVAFAVGTDCGAQHDRRCGFRIRDPTLDMAYARNIVDHVCRTRNVDESRIYC
                     VGHSNGAIFSSILAERLGGDVFAAMVNVMGGFGKEAAEVAPRRADKPVPLLFVTGTKD
                     DYKQGCECAHRFFSANGYPSSIKVLDGVAHVYPAGDEEQKMWSFLESHARP"
     gene            284310..285266
                     /gene="pino_cds_170"
     CDS             284310..285266
                     /gene="pino_cds_170"
                     /codon_start=1
                     /product="Hypothetical protein"
                     /translation="MDAQQPTRKAVALEGSAETAPPTSAKNLLAEALACMAWDGIVDT
                     SRLASIEFAHDEAAARRVVALAARLIDSRMRAGIMIFPSDGITIGGVDMVEDWNVSRF
                     VAVRHNGAWRVEALGATVTMTDERLALYCALCARGARFSMTWQVCTDDMKRAGHDSAS
                     PLEAGWCLDWRSMWMSRVVRALAAASGAWTGDEVMRTQSLARVLLAVLEARNAKGPLA
                     GLDGSNNAQPTTPSDAVTSTRIAEAALVATGMPRGIEFSSTTDSTIIMDMTLDAVHAV
                     EMLHMIINSAQRIYETESLYTQVILPAEYDALQRNTPAGAFK"
     gene            complement(285533..286326)
                     /gene="pino_cds_171"
     CDS             complement(join(285533..285826,286066..286326))
                     /gene="pino_cds_171"
                     /codon_start=1
                     /product="Morn repeat"
                     /translation="MDALPCEIMLAILEWLERPSDLAAIGMTSRQWHEVASDDRLWRA
                     LLRRRFPKWADDVERFARIDPGLRPDTAKDTLRMLVTCAACGRRGWAMRLYIVAMAEQ
                     VAMGKFGSQEEFDAAWQKREAAAAKRREAALCRRRERQAIEAAVLADIAARKAAEQGD
                     VCKRGRKRKRPDRRHQSDDSQRCE"
     gene            286654..287612
                     /gene="pino_cds_172"
     CDS             join(286654..287343,287487..287612)
                     /gene="pino_cds_172"
                     /codon_start=1
                     /product="Hypothetical protein"
                     /translation="MDSQQLTKEAIAPEGGSETAPQTPTDGDLLAEAMACVAWDGTVD
                     TDRLASIEFAHDEATVRRTVALALKSFESPAHARAGFSKGITVGGIAIHDRWASNSIT
                     VVRNRGAWSIATESHIRTPTMTDEAMVMYCALVARHMHFSMTWTKAVDDDVRGNNALS
                     DFGYPLEWKGMWLSRVIDALAGASGPWTGVEVMCTQSLARALLAVLRARIDQGPLAPV
                     DWHDGARPAPPAASWLCRQCRAAIYETESLYKTVVLPAEYDALRRNTPAGTFQ"
     gene            complement(287787..288518)
                     /gene="pino_cds_173"
     CDS             complement(287787..288518)
                     /gene="pino_cds_173"
                     /codon_start=1
                     /product="Hypothetical protein"
                     /translation="MEARRGRSGPTAADVAEFKRIFARPEGDPKDIGARIRQETPIDD
                     AHLLKKERTRKTVSIARRPPQGKTYPPSLYHTETLWAVQVGDFFYGLVLFPSADMVVP
                     GSSVAQLTMITHGVEPVFVVGHTYLEYHEVFDAISKAAVAFGVKDGPHVLYWHAVDFL
                     KTAAEAVCAADGGCAWDDIQLDEATVRAGLFAARAYAYDAQNFSPSDATSACARTRSA
                     AYIRGHWPPVIDPERDAIMPLCLLS"
     gene            complement(288874..289962)
                     /gene="pino_cds_174"
     CDS             complement(288874..289962)
                     /gene="pino_cds_174"
                     /codon_start=1
                     /product="Hypothetical protein"
                     /translation="MEATDAHGAGKGHVSEETRPTAATETITSTQREAALAEAALDCV
                     AWDETVDVGRLLLLEHEHDVGLLRHIVARAGARLDEYEEHRSFEFINDARVMDLDIFF
                     DDESVGRESVWVHRYDGAVWSVGTDFDRRLTDAQVTSWLALCARHCLFALDYDVPARE
                     NADSEHPDQDDDTCGEEDHETTWLAAVVRGLAGRSGAWTGAEVMRTRNLCLVLLAILD
                     ARAKGGPLAVVDMPDGNKVDQGAMPSLADRREGDDTLLASMGDALAQVEHVRQSGACV
                     LCQPRRLGHAQPRWSHSPGALNGLRRRRPALCHRVLLPWRNCARRIRGHGRQWTPRCH
                     FFPLKMICFLFFFRLSRSGRRRRAPNKK"
     gene            complement(290238..291152)
                     /gene="pino_cds_175"
     CDS             complement(290238..291152)
                     /gene="pino_cds_175"
                     /codon_start=1
                     /product="Hypothetical protein"
                     /translation="MNAVAHTPDGGAPDTAGAPPTQKQGGPEEALVGAALACVRWDES
                     VDLDRLMALEFEHNFGLLASWLAQLSPIARRLVQKLECLTRRPSGESVDLTPHDDGTW
                     SLDNGERMTDAQLAGLLAVCARHTKFRLTLSATDTTHFAQTWLACVVDGLARQQRPWT
                     GIEIMRTRNLCVALAALVDARTECGPLGHVDVPKSGPFDQGAVPPACADVGDDVAICS
                     AAMREAMARVHVLLQTRLAFRGRSVGLWDPVNTVYTFDFTRRLLAAVQSAVERLYATE
                     SVYIKMIAPAEYKAMVAEAPLGATFFRP"
     gene            291608..292432
                     /gene="pino_cds_176"
     CDS             291608..292432
                     /gene="pino_cds_176"
                     /codon_start=1
                     /product="Hypothetical protein"
                     /translation="MRGAVSKSQMASAAWRAEGAKVTSTAALVTGPTRDGHPVRLALA
                     VVYAHRGRDARRIIIGEYARDFYPLLGVDAEATTSDPATADDHAEHTWWPGQVHLCAT
                     LSSVCREAGLHALGDFDANDLEKQDAAKNVADAVAGHVRAWIAEWGALSVVAMTRAWP
                     VIGADGNAFAASLRWSALAPTMSSFADATMSVAGLPDAGDAQALRDFYDLTGFRDAAL
                     KKAVAAGTEPYSYDGVRDMARRLRVFEEYALCHERPTLVSGVRAMRRSLYASPCRL"
     gene            complement(292645..294614)
                     /gene="pino_cds_177"
     CDS             complement(join(292645..293784,293904..294614))
                     /gene="pino_cds_177"
                     /codon_start=1
                     /product="Hypothetical protein"
                     /translation="MEATGPQEASRDLTVGDAAAKMQKDIALVGAALACVRDDRSVDL
                     ARLLLVEYEHNLGLLQGVVAKAGRRLDKHKQDPSYECGYDTGLCQMDLSFLSKTKSAG
                     TRATTVTIARHDEALWSVRPDSRRMTDVEMAAWMAICARHCAFSFLYDAFVKECERQQ
                     PRRLGYVDYEEMPCFKHMWLASVARGLAGRMDAWTNANVTRTRSLCDALVAILDARIE
                     GGPLAIIDTPAGDSLDQATAEYSRPNLEDAADHTRALLCTVYRTTDRLDATGRHLPTP
                     VGPDEVTCRDGTSAGPAKTTTPPTEGTSDTTEDTRLADAALACVAWDETIDVGRLLLL
                     EHEHDLNLLGRAIARAGARLDEYVPSRSSEFVTHTRAIDLDLFSSWGSVRTDSKAILA
                     NRHDGALWSIQDDDRRMTDAQMASWMALCARHCSFMLEYNVQAAAEKDGRRPDCCNGV
                     EFEVYERMWLASVVRGLAGRSGAWTGIEVMRTRNLCLALLAITAAREKAGPLAVVDLP
                     RGHRLDQAVVPMPLVPREADNALLASMGEALAQAEHVRQLGFAFFAGYTAYRHLNTID
                     AADHTRQVLSAVYASVERLYATEAYYVGLIAPAEYGAVVANMPSGATMLC"
     gene            complement(294843..295832)
                     /gene="pino_cds_178"
     CDS             complement(294843..295832)
                     /gene="pino_cds_178"
                     /codon_start=1
                     /product="Hypothetical protein"
                     /translation="MDATTNPGNKDMPDAAAGTAVGAQSASDALVNAALACVRWDESV
                     DLDRLMALEFEHNLGLLAPRLPQTRPGKRPDHWKLYCLFKRAHSTLSADQVSLLRSHG
                     YADPPTADEPVKLTHRNGVWSIDDAEHMTDAQLAGLLAISARQPSFWFWCSFSERVCF
                     AHTWLACVVDGLAQREGAWTGIEIMRTRNLCIALVALIDAREKDGPLGRVDAPKSGPL
                     DQGAVPTAWADDADGDLAVSMREALVRAHRLVQAGFAFCPRDTTQPANSVDAVDHTRR
                     LLGSVQRGVERLYATEPYFLKMIAPSEYEAMVAEAPLAATFFHRDSEHCPPSF"
     gene            complement(296032..297012)
                     /gene="pino_cds_179"
     CDS             complement(296032..297012)
                     /gene="pino_cds_179"
                     /codon_start=1
                     /product="Hypothetical protein"
                     /translation="MEQEQTQDVVTPNTQPAQDLPTTGQQETDKEALVDAALACVRWD
                     ESVDLDRLMVLEFKHNLGLLTSGLSLLPSAGRPGSCKLPCLVNRQKDATLGAYYSILR
                     NHGYTGPESGGEPADLGRHDDGTWSINRGERMNDAQLAGLLAVCARHATFLFIAGPSG
                     KVGFAHMWLARVVEGLAQRADPWTGIEIMRTRNLCIALAALMDARREDGPLGRVDVPK
                     CDPLDQGAVPSARRDADDNDRDLAVSMHEALTRAHQLVQGGLIFSRHTMTHESDNDIG
                     AADHTRQLLTALQRGVERLYATEPYFLKMIAPSEYESMVNNTPLAATFYR"
     gene            297451..298539
                     /gene="pino_cds_180"
     CDS             297451..298539
                     /gene="pino_cds_180"
                     /codon_start=1
                     /product="Hypothetical protein"
                     /translation="MTHTHYRSTNRKKHPAETAAAAARDRAAARGTATVVRDRVVGSD
                     AAAPETAAATPPLPVNPIAWVFVASTEADPKPKPKRCGRPRKADNEAPSSKSNAWYYK
                     NRDKASADAKRRYALVKDDPAYIERRKQRVNRRYRENAGGLRDKIHARNRTPAHRYSL
                     YRTQAKRAGRAFTITREQFDSMFFAAACSYCGTARVDPQTLGIDRFDNTVGYEPENCR
                     ACCAACNYMKSSMTMDNFVKKCRTVRDVSVNGPPTLTAAQVNALPIMRWDKYMCNTFG
                     KYKYTATKRGYEFALGKCDFARLVDEPCHYCWTARGGIDRVDNAVGYLLPNCVPCCGT
                     CNAMKNASTKEDFIMQCTRVANFYCSTQ"
     gene            complement(299735..299962)
                     /gene="pino_cds_181"
     CDS             complement(299735..299962)
                     /gene="pino_cds_181"
                     /codon_start=1
                     /product="SCF ubiquitine ligase"
                     /translation="MSAIIKLESSDEQVFEVAREVAEMSVTVKHMLDDVDADSENPIP
                     LPNVTGRSSAKVIELGQLLRRREIFRLGQPS"
     gene            301351..301872
                     /gene="pino_cds_182"
     CDS             301351..301872
                     /gene="pino_cds_182"
                     /codon_start=1
                     /product="Hypothetical protein"
                     /translation="MGHVSTESETTDAGTDSSSTSWTDSSPSEHRRRSRRLRQTRARM
                     PRPDHQLEGCDVCRVVCVVLGLLLGAAILVCIVHFDEPSGSAVTPLHTCEHIVPASAL
                     ESRWCMCKGIDDMAAWGDALAAGDTRLCQQYRLTHTAHAPRASISWEKIAGALWVPLA
                     VVALVTFCVFPRK"
     gene            302838..303650
                     /gene="pino_cds_183"
     CDS             302838..303650
                     /gene="pino_cds_183"
                     /codon_start=1
                     /product="Hypothetical protein"
                     /translation="MDIDRILGLPRTNDVVTNETTSTVDDTIGTDNDATGTNNDINMG
                     TSDNTVTDAVINDTTGTSATVKTAVAAGQGDFYAAKEADCKPAADNAVKGNTGESRTP
                     PDRASSAVRSSPVPRAAVPKRPWIDAACLGREISSVFIALVVSIVVVSIMRPHAVVTE
                     TTPSATASAAYYYYVPKQPASTPTPVPSPEPCWCLFSNNADEGLSKGQLVGTDCVCHV
                     AVESAQPSLDGDDGSRAPADRVTLADRVVLALAVPLLVLLLIPMAILTGCPM"
     gene            304707..306435
                     /gene="pino_cds_184"
     CDS             join(304707..304932,305012..306435)
                     /gene="pino_cds_184"
                     /codon_start=1
                     /product="Carboxylesterase"
                     /translation="MVMTIAATATAAPIAQTQEGAVAGVDTRDAYEYRGVPFAAPPLG
                     QLRWRPPVDAEPWAPDVYAATTTPPVCPQLPIMVFLTGGAFTLNGAATQVFNGSAFAN
                     ATGTIIVVPNYRLGILGLMGHPDFMADSGTFGNYLLMDQVHALKWVKRNIGGFGGDAD
                     RVTLFGESAGSISIGIQLALPHSAGLFRNAIMESGAPVVLDPPSVYSTAAAQVQAALG
                     CPTGAPWEDTVACMRAANVSTIMAVQASIPYFVLPRPAVDGVFILDQPLSLIRRGLHQ
                     RNVAVIAGFQTNESNIYVGAIAAYRGFNSTEADFEARIASRFGADSVDDIAALYDVSG
                     APGPFSLPSPFSGMGKADTDFGYACPARNYMRLLAEGGSRRLWFYRFNRAAPFAPAEL
                     GAYHTGELAYLFGHPCLVPVMNAQPPLEPFGPSCLGDEAPLWDATAHPADYALSRTMM
                     DVWAAFAATGNPNGRGVFARAARRPWLPYAAALGYPNAVFDANDATGKAHLWTENRVN
                     EERCDYWVGRMAPYCGDDVCNEAPASCPFDCLWGHHHHPHH"
     gene            307237..309204
                     /gene="pino_cds_185"
     CDS             307237..309204
                     /gene="pino_cds_185"
                     /codon_start=1
                     /product="Hypothetical protein"
                     /translation="MEAQCAGLFGDDIATPPPPEACASPGLGDMPPEIHAKIVACIDR
                     PSHLLALRCTSPLFTQADPVTAAIAWGAQRMHRLLPAGAPRDIVAAALAARGRPLCKS
                     AIVHAVRGGRLDVVRIVFENIAVRRAHFVPPRFLSPSFFFYSHPHRRADPAYAKREQA
                     VWPDIFEEGTQTHWTLIKEITEKTCIEARMQTCILTHARGSTNQQATTACEQEDTDDG
                     AAPPNLPASTMQERNKVNVAGMMAAVFEGHLDIARYIYRESWHVRDNITSSALAKHAA
                     ASGRLDALVFAHDIYARKHAQGCGCGTDVGHSAWLAPAPDLVRWMRATGCDGVCHFEV
                     VHVAHAVRSGHLAMLRYMAADGWRCIADINQDFVVSGAIADVAEGGQWDTIVTTIDLG
                     LCVSLTPIIVGAASGDRVDIMARALDSGTMCTASLPRPDRAAARAAVVAAATSDNVDA
                     LKWLLEWFGADLADTPLMWMALLATAMDVVRFLETLLRDPFPWADALPFVLRSSFAPA
                     ARYLIEDKQVPVTPIAIAAAAGRDRHDSMLDFLCTVCPPDRLQAAVDMMGAVEDDCVS
                     TVRGIKRRVPELCTANIVAMQTHTALTSHRQDNDYKPIKPCGCTRCTAALLPLSEPSQ
                     PHLMGRAVDADSEVREPALKKSRTDLRVLQR"
     gene            310306..312183
                     /gene="pino_cds_186"
     CDS             310306..312183
                     /gene="pino_cds_186"
                     /codon_start=1
                     /product="Ser/thr kinase"
                     /translation="MPILRGSPATLCVIAVLATIVLLGARTDAATPTSVTLIGDGGPT
                     AGKLIRALAADYQYTRDDIGLVYIDATPADAIDDYAKGQADFVALHTFTGVGARRAGM
                     RFAPMAATALVAAHNAPPCPGHTGPLVVTCDLLGLLWSGGVTSWSDARVAALNPTCST
                     ADPVASNVTLVMVGPEGDDVEDAFMRALADCSPDFAAALAIAGHNTSRLSPANTVRIA
                     DNRMAWLETAPAGALTFAAAWETTATVPAATLLNATGHALAPDTASVSAALLSVSAAF
                     NDGLMTDPASTAAGAIVGIRAAGVPGAWPLCAMAWVGVDLNGTGRGSVQAFGSADCSY
                     TQELLRLLVWAQVNSAVGTNSAAGVGYVPVPFAWVYTAVNALSDVRCGGLRALSESYM
                     VAMGEPPAQIYQRMSYSYTAGLYRFKFFSGRTVDGIQAMLANQVDLSSITALPTRAQM
                     DLVPDVVLLPMQIGAIGPIYSVPELVGHAPLYFDWSVLTGIYLGEIRTWDHPRIAALN
                     PELAPYLPAGREITIVYQVLPSSVAAHYTHALTLMNATFAAKVGYRWDIQFPVMFDEP
                     NRTVGIVGMAVPPAVQAHPYSFTFWPTHALQGVVASSPAAWSTRRATACCPTRPRWHR
                     P"
     gene            312420..314982
                     /gene="pino_cds_187"
     CDS             join(312420..314239,314298..314982)
                     /gene="pino_cds_187"
                     /codon_start=1
                     /product="Ser/thr kinase"
                     /translation="MPILRGSPATLCVIAVLATIVLLGARTDAATPTSVTLIGDGGPT
                     AGKLIRALAADYQYTRDDIGLVYIDATPADAIDDYAKGQADFVALHTFTGVGARRAGM
                     RFAPMAATALVAAHNAPPCPGHTGPLVVTCDLLGLLWSGGVTSWSDARVAALNPTCST
                     ADPVASNVTLVMVGPEGDDVEDAFMRALADCSPDFAAALAIAGHNTSRLSPANTVRIA
                     DNRMAWLETAPAGALTFAAAWETTATVPAATLLNATGHALAPDTASVSAALLSVSAAF
                     NDGLMTDPASTAAGAIVGIRAAGVPGAWPLCAMAWVGVDLNGTGRGSVQAFGSADCSY
                     TQELLRLLVWAQVNSAVGTNSAAGVGYVPVPFAWVYTAVNALSDVRCGGLRALSESYM
                     VAMGEPPAQIYQRMSYSYTAGLYRFKFFSGRTVDGIQAMLANQVDLSSITALPTRAQM
                     DLVPDVVLLPMQIGAIGPIYSVPELVGHAPLYFDWSVLTGIYLGEIRTWDHPRIAALN
                     PELAPYLPAGREITIVYQVLPSSVAAHYTHALTLMNATFAAKVGYRWDIQFPVMFDEP
                     NRTVGIVGMAVPPAVQAHPYSFTFWPTHALQGVVASSPAACRLCARHGRRRHCPTRRR
                     PGGRAGRALVARRHVQLFDVAHAHFVDWIYWAQSTSEARALAEQNYVVMCSASEGMMA
                     RVLAIVVNITVDGVPVSSLYGCVAPNDGRVCSDHGTCIDSVCVCTPPWTGVHCATDGT
                     LSASTDSTSVIIPAVVAPVVAGLGLLIIVAALVIAMVMWRARRRLHDDDWEIDPTELD
                     MGDQLGAGGYGTVHKAKWKGTVVRRRAARPRRRPPG"
     gene            complement(315071..315407)
                     /gene="pino_cds_188"
     CDS             complement(join(315071..315218,315331..315407))
                     /gene="pino_cds_188"
                     /codon_start=1
                     /product="Hypothetical protein"
                     /translation="MEYMALGSLHDLLANELIPDCRRRLRCLTLASPRGAGDHGSRTS
                     TAIEGTVQWMAPRSLPAITPLLAQRQRRGP"
     gene            315507..316913
                     /gene="pino_cds_189"
     CDS             315507..316913
                     /gene="pino_cds_189"
                     /codon_start=1
                     /product="Ser/thr kinase"
                     /translation="MPPTFVDFERLVVDCWNRDPMMRPAFLEAMTRLSTIIDGDTSSS
                     AGGRYTGTSSSSSSMASSSSYAGSSGGGGGGAQADTDEPSLLADSVRGFDRSSPAATP
                     ITVHVGHKRAPVADDQGPVTVVFTDVHRADTLWDEIPRAMKDALVEHNLTVRTAVAAH
                     GGYESPFGADRPAGEGTFCLVFARADAALDFCRTAQTALLDAEWPPHLLHHADACEET
                     GGNADDAVVFRGLRVRMAVHTGRVRATMDPLTRRYTYTGPGVDGGGRVGLRRQGVACS
                     CRSTRVAPRMLVGSSPPSPASPSSSTGADIDTGSNSDGAMGALSLVGWRRRPWPCAAV
                     VQQRRPANSTLSRRHFGGAHLGPDADHDRGDDNYSRWRPAFHRRTTTTTTTATSNSIA
                     TRRARRTRVPCFGHVERVAVVLDYEEIVVGEQIAPAPMASSIGASGRASTWPSNALSS
                     SVSTRPAHRLSRRGGRPV"
     gene            316966..317559
                     /gene="pino_cds_190"
     CDS             316966..317559
                     /gene="pino_cds_190"
                     /codon_start=1
                     /product="Ser/thr kinase"
                     /translation="MCASSPSGCAAATCANCWPVDGQCSGACVCRSCATLRSASTICT
                     LRTPTLKIMHRDLKSSNVLVAPDDSSDGAWTAKLADFGFARAKADMATMTRCGTPAVT
                     APRSSAARPIRKRPTSTRWAWSCGGAHAPPALCDANFVRISLDVLEGSGPTCPPTALP
                     AFAQLMQRCWHRKAHKRPSAADVAAGLLAMANTELLV"
     gene            318437..319324
                     /gene="pino_cds_191"
     CDS             318437..319324
                     /gene="pino_cds_191"
                     /codon_start=1
                     /product="Hypothetical protein"
                     /translation="MTDYETLAWSRSALDESVLSADDGDAALRGLLRRYVSGYVLSTP
                     SAADDIDDDGGDNDGDGGADDRDQLFFDQHYSARMAFVHRDASKSRDIDQNNDDDDDD
                     NLADGDEQDTGEHAWTGQCVGAGQRDSVYLAQDTQDNDEDGATLLAMLLARVDDDDEE
                     EEEDGEDVDGAGDDAQRAYADALVGVLGDVDEVHLHSDGSVALRKDRRLPVLLMGGVA
                     APAKIADASLTAAQADDGVTVLLGEVALLADEVGAAERACWPTTPAPSSPPSTTTGCR
                     TRPSTTCTFRPSRATPLPF"
     gene            319799..320155
                     /gene="pino_cds_192"
     CDS             319799..320155
                     /gene="pino_cds_192"
                     /codon_start=1
                     /product="Hypothetical protein"
                     /translation="MIAASEPVSMPFGRRDDIMLISPTMRTHIDQLVLSVQRARDEGV
                     PLDDAFRDSVCRRLGLTDDQLTALLQVALVEQRKQIEKARRTQRRLEAELCAKRAGSR
                     RGPLSGVWDFCLSWCG"
     gene            320638..321132
                     /gene="pino_cds_193"
     CDS             320638..321132
                     /gene="pino_cds_193"
                     /codon_start=1
                     /product="Hypothetical protein"
                     /translation="MQRRWQSRDSSDRHRATELDHGSHVIGPVVMRFESAHNGDAGEV
                     LPTVRVRLDQLALLAQRARDNGVPLDDAFCDDACRRLALTESQFDNLLDMTLADKHRR
                     IEQERCKQRSLEAERCRLDAELRHGPHSCSVWTLPAHCSRGWRQLLPCMGSDQKAGGK
                     LKLS"
     gene            321269..322330
                     /gene="pino_cds_194"
     CDS             321269..322330
                     /gene="pino_cds_194"
                     /codon_start=1
                     /product="Hypothetical protein"
                     /translation="MWPFGKGKTLGGPCRWFVFFQTKKEEGTNAATAQSPASVGPPAA
                     YKNRKGHQTKKVLNRHNIHTTGLTLSHNPPTMTSTFFSNPADVPAEATADAKPTLTLG
                     AFWEAVSAMPKDAPVCRIKLAPAFVVPKQSYLETGPLASLDKIDPDAFEGAAAITVYE
                     DRHTFGPASVVAAQIEPLLGRHSHRRLVSGGDAVTGLLTTTRLATGKPLTADHLCPDS
                     AVTSETIIRATEALARTTKVDDTSIVAAMEPALPDGIHVCLYRVGDRVFDTAAFKTTF
                     PRLTTQERRDIFTRVDRPAGARTGVYGRLSMWSAPADITYEMLVAAFGPTQSALDRLR
                     NIAACAGAVPTARDPACPQ"
     gene            322751..323678
                     /gene="pino_cds_195"
     CDS             join(322751..322824,323000..323221,323480..323678)
                     /gene="pino_cds_195"
                     /codon_start=1
                     /product="Hypothetical protein"
                     /translation="MSQAKGTPLRSATTPVEVVASTVAWDDMTLRYSPDVDAIVIDLT
                     PAEYVTNETVALDAPSDAFLDVDNVGKAIRIEFLDASSVFACHFHDHPDDVDGCGPLE
                     WRARYESDRLTLSFVGDADRRAVAASDMDEGITMHTDDAGRIVAVSIADAIRIVHRRV
                     RPHS"
     gene            324108..325163
                     /gene="pino_cds_196"
     CDS             324108..325163
                     /gene="pino_cds_196"
                     /codon_start=1
                     /product="Ankyrin repeat"
                     /translation="MAEQGADEAEKANGQVHTHPECGIDNLPDELVVDILVRVECVWL
                     RDSAALVCRRWRRIIFAYIPQRCRGHASGLGEGWATLEAAHHGHLGCLDDLERRGLLC
                     WSKSVAYTAAANGHLGVLERARAAKCPWDETACQAAARSGRLNILQWLRRHGCPWDER
                     TTYGAAFKGHIDCLQWALENGCPLTAWTIEGAAAGGHLALLTWLDEQWRKIVAKPIPA
                     LFLYDGSAVVPEPWATTPWTARLTAAAARGGHLACLAYLYERGCPWDVRTMTAAATWG
                     HFDCLVYAHEHGCPWNAGATDGAFDAGHHQCLVYAIEHGCPLSARVASEMVWDDGDQV
                     GDSLATEAYPSRALPDP"
     gene            complement(325430..326995)
                     /gene="pino_cds_197"
     CDS             complement(325430..326995)
                     /gene="pino_cds_197"
                     /codon_start=1
                     /product="Morn repeat"
                     /translation="MAESDCPEREGASKNNAQPASDDAEAAKRWVCMEDASPFDCVPE
                     EIALAITRALGDDPASLFWWALTCKRHYLLAMDATVWRGMCETRFGPPLHQRFLDADK
                     DWRWLYKAQARIADAASSDLQVGAATIDLDWHRRVYWGDLVGGKPDGYGLALAIPRGS
                     TRPPTRNRDDNGADPPSRYEGHWKDGKRHGYGVNVTRGGETYDGYWQEDEYHGHGICR
                     WVDGSVYEGEWKDGERHGCGLHIYASGDSYRGEWEHGRCHGRGVYDSGDGFVYDGLWQ
                     EGKQHGHGSAQYANGNRYEGDWARNDRHGYGSYLRPDGSRYDGQWENSLPHGYGEEAA
                     PQGRAYRGLYRAGKKHGYGVGIGDGLAEYDGQWADDYASGYGMARCPDGRTYHGWWAN
                     GVRCGYGVQRWADGTECAGVFRDRQPCDGNGNTPGRPDGEQVASIECDSDTLHMVVTR
                     PDGFRYEGGWTPSLGSTGHGTCVYPDGSCIVGTWSGKVPLDGEITSHGTGDAMSCETS
                     APCTACKVMAQRP"
     gene            327480..328507
                     /gene="pino_cds_198"
     CDS             join(327480..327639,327780..328507)
                     /gene="pino_cds_198"
                     /codon_start=1
                     /product="Ankyrin repeat"
                     /translation="MESCAEHPPPFDTLPDERMAAVLSCLPCVDLCQRVIRVCWRWNR
                     IANDPTALGLPCRGRREGHLDCLQYAHRHGCPWHPYVCEMAEAHGRVDCLRYAHERGA
                     KWRGYCNDAAGGGHLDVLRYAKEKSLTHDLDVCWQAAGNGHLDVLRYAVTNGWSMCVG
                     TLSSAARHGHLDCLRYLHEVRGNGTIVTPCLGAAAGGHLDCLRYAHEHNYSWVSDVTR
                     MAAKGGHIDCLRYAHEHGCPWHLRTCITAAKRGRLDCLRYACENGCPWGTATCARMEA
                     VGADPACVQYARDNGCPPD"
     gene            328770..328922
                     /gene="pino_cds_199"
     CDS             328770..328922
                     /gene="pino_cds_199"
                     /codon_start=1
                     /product="Hypothetical protein"
                     /translation="MKEGSAVSPRFPMAAKCNCPIVCASASKEAHDVYCHDVVPQEKE
                     GARACG"
     gene            329324..331213
                     /gene="pino_cds_200"
     CDS             329324..331213
                     /gene="pino_cds_200"
                     /codon_start=1
                     /product="Ankyrin repeat"
                     /translation="MTTMAATRRTTINDMPNEVLAHILDHVGCIEATAVWSLVDRRWC
                     ALVSSARATGRWACAGAQCVARAREQRRSRKYAMRMAAAGAGHTHCIEYLGVGASVSA
                     RNLSEAAAANGRLNVLRWLDDIRCAWKSARVVLCAAAGGHHGCLDYALVKGCPASTQA
                     VEAAAAKGHAESMRRLLAPDNDRSDTVDKSDSAVATAAARGGHLDCLIYAHESGWAWS
                     RRTTTAAAKAGRIECLAYAHENGCVWSTRTARAAAKRGHLDCLRYAHEKGCACDSHTV
                     DCAVRGGHAACLDYLLGVAVPGGADAETWWLAAACGRVACLDVLRVHAAGRNRAEYVC
                     EAAIAAGHHDVLLWMIANLNYRAIDKEHRAARLGRLECLRVVAAVGPQQGDASIAAAA
                     SGGHTECIKYLRDKGFPMDARACEAAAGGGHLECLTFLREVLGCAWDERACIAAAAAG
                     RVDCLAYLHRNGCPWAHDTVSAAIKRGRVACLTYALDNECVHDAYAAAVEAIDRGRTS
                     CLDALCRAGAPLDQHLLAKCIRSGQSGLIKVLARHGCPRGALTCHDARDCEDARVLCT
                     LYKLGFPWGPSRRIMGRSEDAVSRLANPCAMPKPRKEGARRRRAGKANRRGSPGGMLT
                     IALVL"
     gene            complement(331902..333477)
                     /gene="pino_cds_201"
     CDS             complement(join(331902..332021,332149..332343,
                     332488..332619,332812..333477))
                     /gene="pino_cds_201"
                     /codon_start=1
                     /product="Replication factor small subunit"
                     /translation="MMMAATVTTTTTVVTPLGRRTTIVSEGSTEPQPRRTYAAVRGDA
                     DFGLPWIEKYRPMVLDDIVGNEHAVAYLKALAADGNMPNLLLAGPPGTGKTTSIACLA
                     RVLLGAAADEAVLELNASDERGVDVVRKIIKHHASKRVDLPHGRHKIIILDEADCLTP
                     AAQQALRSTMEKHTNTTRFALACNTSSMVIEALQSRCAILRFVRLTDAQIAGRLREIV
                     AAEKDVVCTDEGLEAIVLTADGDMRQAINNLDATHTGCGEVTPEHVQRACDQPHPALV
                     RAIVLSCAEGDLSKACASMKQLWDMGYAASDIMGTLIATAKRIDMDEALRLELVKEIG
                     ITHGRVVRGLGTLLQLDGLLARMCRVSRAACSRPPQ"
     gene            334103..334738
                     /gene="pino_cds_202"
     CDS             334103..334738
                     /gene="pino_cds_202"
                     /codon_start=1
                     /product="Dutp pyrophosphatase"
                     /translation="MASTSSPSLPSSAPGEPGATFTDDLSVAATPQVSSLDHVTHDDA
                     SDSVPAVTGERKRPRPESTDDDDADGNPAVLKCKRMHDDAKLPRRGTSGAAGYDLWAV
                     GKTTVPARGRAQVPIGLAIAIPAGYYGRVAPRSSMARDGIDVGAGVLDVDYRGEVGVM
                     LFNHTDADYVVGGGARIAQLLVEKIAHPEPVWVDSLDDTERGTGGFGSTGR"
     gene            335307..337262
                     /gene="pino_cds_203"
     CDS             335307..337262
                     /gene="pino_cds_203"
                     /codon_start=1
                     /product="Hypothetical protein"
                     /translation="MDGQEGQSRRKRRVRSRTRRAATAAKSRLPTEVWTLVLAHHLPP
                     RWRFCARPVCRLWRDILAGAPTGRPVDPALGVAYADLDEERYALQEARGRLVRALVVL
                     VEWPLLPGAPATPEALVDFCLAHAGTTDDRLLGQRWLRPLDIVLASLATGTPALIDYA
                     LGNRLNKEVNNVPHGERDSATRKIYGAVAGAAIKIGGMALVGRVAANVPGFDFVDHCS
                     LKDAIAADCVDAVVALTRDRRERAFCDPQWWKIIGTHDAIHVMCKFIADAIADTNFAP
                     GSRGVPLVLLKRMGRRYDDGPCTVIAAQAAAVHGATRVLEFLAQWGGGATLGTKDTPR
                     ANIETLIEMAAGAGNTKTLAWCLSRYPPQDPCAEAFSMACDAVIEPHYDHRFDIHVPW
                     DPYRAVRTVAWMRDHITNGGDGNGAPRHAILKAGDGKRLLCQMARHPGPYHCDIRCVF
                     GLVALFGPMTAARVAVDKGKSCRALADTLVSIACEELVARGAASIAERLTLALEGLAA
                     DMTHTGGDATALHDAVDPWSALTRLWGHLKPSPNGLAGAMAYVLARVDGRSCAESLLD
                     AGRAARPGVATWTQESYETDLLAPPVASTAAWRRWWRPGPVSTAHAHERDTVHRGFWG
                     HGPGPALGDVGTLDAFLFLRARGLLLD"
     gene            complement(337725..339788)
                     /gene="pino_cds_204"
     CDS             complement(337725..339788)
                     /gene="pino_cds_204"
                     /codon_start=1
                     /product="Hypothetical protein"
                     /translation="MDRRLHSRAESPPSRHGDTDDKDENRGAMEVDDIEGKEGDATDE
                     NENKVRRDDTDRHDGSDSGGDHDDGGLYEPIASGFVSAGLDVEAEAAALDALCYWTRE
                     NIIRPDQEVELARLKAGDVLGDVRLADADPAHGSATDTQLIERYRRMWRRLADSDACV
                     AHMSTVASGWPPTLADVERAYRDLDAAYRRRCHGSNPAGDSEDDTAQTLIAYLAAEAR
                     AGRPYPGDILADAVVRTLERSSSTLVASQRWPHYPDPFGGRSVPRHVHGPACVASGEV
                     DADYFVLAVCRSETAEAVLMSVPEGGRARAIAGASLLHDVSHKRIAASMPVDVTGLCR
                     CVRPYATMLPTFLGAVATVLHRPAYGPPEDGIEEDQDHDDDDDVIADGAWHVSSAMQA
                     MPSLYDAPRSVVQAVDEIDDRPYAWRQAEQLCGLLIEPGQLALALSLAAGARAATMVD
                     PPASSLADMAAAAYDGPLDAPGMCAEAGALVAARIWRRICAGSADDTGRLPGGGRLVE
                     VALALDLAPTDAERAVPELLCGRLIEPIVRATMRARGMGMVGDVQPTDASSHTLGALT
                     VRGMAIAARLAPENDPMTSGAMVLADAIAQRGGCVDLDRDLSNSHRLAEVFTRTLWPS
                     AGGDECDAWERLYAADHTSRPDPADVALLEALASRMGIPVDDHHQVTAGALCGLLALA
                     VHWPS"
     gene            complement(340224..340562)
                     /gene="pino_cds_205"
     CDS             complement(340224..340562)
                     /gene="pino_cds_205"
                     /codon_start=1
                     /product="Hypothetical protein"
                     /translation="MPTPFNEVPPPPTFAARVFGRLRDPADALGLSFVYALFVFCRRL
                     GAVGRQPRRRTDHARCVLATTWLCMGGRRPGGGRAFDGSARRAALCRHLALRLWTALA
                     RVARARASSP"
     gene            complement(341136..342720)
                     /gene="pino_cds_206"
     CDS             complement(join(341136..341850,342041..342720))
                     /gene="pino_cds_206"
                     /codon_start=1
                     /product="Morn repeat"
                     /translation="MGRGRTRERRRRRGSTRRGLSYAIQEPVFHDPRLPSPFDDLPDE
                     IVVAIVAAMGHDMATVARWVLTCRRHHALAMDATVWHHLCKVRFGPPLHRRFLDVGKT
                     WRWLYEAQARVGGGDRAGPQTGAVLVDINDTRWVYWGDLVDGCPHGYGMAFPIPRRRT
                     RCPVRVPDDGVVCQSQGRYEGYWQHGRRHGYGTGVADVAYGGATYDGHWKADKYHGHG
                     VCVWPDVHVRDGDRYEGDWSDGRWHGYGLCTVTKGGQVHRYDGQWHYGTLHGYGEDVG
                     PNATYRGLHRCGKRHGYGVMVFSDGTVYEGQWADDMRMGYGTLRCPTDYLYRGWWTGG
                     LMCGQGVCRWADGSEYAGAFEDGKPCGAGVYVRCDGERRTTTEDAAGAMHALVTRPDG
                     FRYEGGWYPSLGSSGQGTCTYADGSCIVGAWSGVVVLDGEITLHRTVGAPCAVGSPCE
                     ACVVMAERPLPKKI"
     gene            complement(343602..345657)
                     /gene="pino_cds_207"
     CDS             complement(join(343602..344769,344867..345657))
                     /gene="pino_cds_207"
                     /codon_start=1
                     /product="Hypothetical protein"
                     /translation="MEDDGDQHVMQVEIVDDGDDTVDGVECQANGDGDNGNSNVDDDA
                     QNSIDDGSAEDDDCDCDDILFDEAMTHAVLDVEAEEAVLVTLCHWAREGLLTPEQGVE
                     LARLKAGDILDGVRLADISPAHGSHTDGRLVMRYARMWSHLADSRACIAFLSSRVDAW
                     PPTLADVEQAYRDLDDAYRERCRTHDGDDDNDNNEEGEENAGQSLIAYLIDDARAGDP
                     YPGDMLADALDQALQRMATGTMSQRWPQYRDPFAGRSVPRHVHGSGAAGHVRSHGASL
                     LHEAPCDCIAASMPVDAAGLCSSVRPYAFLLPAFLGAIATVSHIAPQPAVDEDASMDR
                     DAVGSGEVITNGTRHVADSLQPLPSLFDMPQSVIAALDEIDNRVYAWRQPDELGGLWI
                     EPGQLALALSLAAGARAAGAVDPPLSSLADRAAAVYDGPLDAPGLCADAAALIAARIW
                     RRVCADVADDMGRLSGAERLVDVALALDLVPTDAERAAPELLCGRLIEPAVRAMVRAR
                     GLGIVADVQTRDASGHPALDPLVIEAMGVAARLAPEDDPTSNGVMVLADVIARQGGRV
                     DVERDLSCSHRLAGALTRTLWPSASIDERAAWTRLYAADPASHPDPPDVVLLDALASR
                     MGIQVGDHHRATAGTLCGLLALAVHWPA"
     gene            complement(346037..346435)
                     /gene="pino_cds_208"
     CDS             complement(346037..346435)
                     /gene="pino_cds_208"
                     /codon_start=1
                     /product="Hypothetical protein"
                     /translation="MQSTPLTTAVIPSRPPMPTPPNGAPPPLPNFAAYVWKRLRDPMD
                     ALGLAFVYGLFLLVGGWVPLVAGHVVVGAALGALSRLPGCAWAARDPTATALSAGLPA
                     ILLFAGLLRCDYGRLHAAWRASAHTRRLSS"
     gene            complement(346847..347853)
                     /gene="pino_cds_209"
     CDS             complement(join(346847..347382,347472..347853))
                     /gene="pino_cds_209"
                     /codon_start=1
                     /product="Hypothetical protein"
                     /translation="MIEHLLVALAVDGDDVRIDDVARFAAVNRRMHVIAHRTLLPMPL
                     AMTVHLPRRLFACGEVRPTLGDALDYTREMARSSNAGYWTGLAAVVEIGRRCVLQAYV
                     RWTWSTKVKRRNSAARPLALNTIATVARRHRQEPHAGRLKLARLYARSAVPVGRPFDP
                     TTADDRDTLALLLANARSEMIGSISGIELCREFGLQPDTVESWLIRARAPHGEWCPYW
                     VPRLDGMRPPPPIAEALARPESLGVLVGWIDSSIQQAMARDCPSAAARAHAFFPRPSD
                     FIDLSRVYLVPIKGPSQPTLGHLGIVVVR"
     gene            complement(348301..349926)
                     /gene="pino_cds_210"
     CDS             complement(348301..349926)
                     /gene="pino_cds_210"
                     /codon_start=1
                     /product="Morn repeat"
                     /translation="MSKKIEAVEGARRGLGNSPDDVDEHVTETPFDRLPDEIVVAIIV
                     ALGDDLPTVVRCAQTCKRHHVLAMDAVVWRPLCESRFGPPLHRRFLDVGKTWRWLCEA
                     QARVTDGDTRGPQTGAMLVVVGFAQWIYWGDVVDGMPEGYGLALPLPQNRTRCPARVP
                     DDSVTCQQQGHYEGYWHDGKRHGYGVEVTGDGRSYAGQWEAGKYHGYGVCTDPGESVY
                     NGAWCAGYRCGHGSIAYSNGDRYEGNWHAHQPHGHGVYTWASGATYEGAWQYDTRHGH
                     GSFIGADGMRYDGDWENDRMHGCGSMVYVDGCHYTGDWSTGKRHGYGLYTCTQGSRYD
                     GQWQDDAPHGYGESVTPHRTYRGLYRRGQKHDYGVMVFSDGAVYEGQWADDMPTGYGT
                     LRRPDNSSYHGWWVGGREDGQGVQRWGDGSEYVGAFRGGRPHGTGVHIRGDGQRTVTT
                     EDATGAIHAIATRPDGFRYEGGWDPPVGSSGQGTCVYADGSCIVGTWSGATALAGEVT
                     LHGPTCTTESSCEACVVMGKKALPKSQHLTPDP"
     gene            complement(350634..351446)
                     /gene="pino_cds_211"
     CDS             complement(350634..351446)
                     /gene="pino_cds_211"
                     /codon_start=1
                     /product="F-box domain"
                     /translation="MAGLLDLPDELLLAILRRAIPQAKCVQWLAAATGTCRHMRHLWD
                     DDALWQPVLACVVGSGPASAACTGSTCKQLVRLVASTTVDMTVTMLRDQTAMARAFDG
                     VRVTLPPDLARVRRSLMPLTHAPTLALLALRAAGVFSWCLGRTHVWLASHSPMSSSPG
                     SHSWLRPIHIAGAQDALYPDDALWWWNRWTQAMGPGATIAVHIVIASAEVTRALAVGR
                     LAPPAFDVVPVTIPVCVRNLAQQLPLDAPFPVVWYAPESCTALGHLTGCVCR"
     gene            complement(351884..352393)
                     /gene="pino_cds_212"
     CDS             complement(351884..352393)
                     /gene="pino_cds_212"
                     /codon_start=1
                     /product="F-box domain"
                     /translation="MTPVVGLLRRFLSVHLDEEPPLVSLLRAMQAAASAIALQGVSSF
                     GSHSTRVKLDDTVAVLGASPWQLERLVDRACGGVAYSAQERANALYLVLAAAQQVQLA
                     RGVAALVSAGLGPDTRMGGRTVRETFFSLVSQEPPEDRGSRLNPTVAVAHATLALLTM
                     ILCDHGLTT"
     gene            complement(352411..353127)
                     /gene="pino_cds_213"
     CDS             complement(352411..353127)
                     /gene="pino_cds_213"
                     /codon_start=1
                     /product="F-box domain"
                     /translation="MTETTKRQRHESSPLDLWATCWWTQAKRRCLVTGAMCNNDDHDS
                     NDRTADDGGDLVYENKATTQPTLLSLPPEMLAAIVSHCAIDALVRLRRTSRALNVVAS
                     RALDCRQSRVMATVSIRLHPWMAGHFFDDNADAVAVLMMTGYLRESMCFSGGDDCVLD
                     FHAIRAGDRPFDCEVHGGGAFLTMSPVALAIWLVRPMSWLSWHAPPCAAVTRANHCWQ
                     RPRKPPIVLVSAAVPTPWVL"
     gene            353570..355318
                     /gene="pino_cds_214"
     CDS             353570..355318
                     /gene="pino_cds_214"
                     /codon_start=1
                     /product="Ankyrin repeat"
                     /translation="MVGGDEVCGQQRPCKKRRIHETATTWDDLPTEVIDMVLTALDDF
                     DLVDARCVCRLWRALANGIYRRPLRLAPTRSEYIMEMARRGDLGAVCRAWPESMYSAT
                     SASSAQRADEPDPSNHNDNVDGDDDHNHNCDRGGDDPDNVDGRDHAHCWDDDLNPYTV
                     LRLAEESGNGALVQWLCGEQPHLVTGTAVRAIIIDNGGQAAVEWLHMRGHTQRRLFDY
                     GTATATLAGMGRLDALDWLHTQGDALWDASACAAAASGGHLDVVQWLHQHNHPWDART
                     CVAAAGSGHTDILWWVIAHGCPHVDRDVVVGLIRNGLLDDLVRAVECGCPLSSLAWEE
                     AAAVCRTDILEWLHIQRCQTSDAALHRAASKGCVDTMRWLRAHGAPWHTHIFRHAAYA
                     GHLDALKWAAANGCPSTMVALYEAVCGGHLHVIEWLCDVLGFSPDDDSLVRAAALSKQ
                     YHVLVWLRDRGCRYVGNALAVMACNGHLHGLCWAVRAGFAFDARQCHDAVLHGTRNHR
                     ATKKWIEAFGISPPVRARCSLLCHHSHRPEPTGRRLLPIVDEALLLLFAMSARAYAKK
                     KRKTARDPVSCVRSNG"
     gene            355916..356407
                     /gene="pino_cds_215"
     CDS             355916..356407
                     /gene="pino_cds_215"
                     /codon_start=1
                     /product="Hypothetical protein"
                     /translation="MNSAAGSLDTPVMTTMTPCWLTIVERVGDDDYLAQVNTGFIIDL
                     DGSDLNHTAERIYSAIVEVEAHRSHYLARRPCVPETIVLVAGGTHMASSTGAISANDL
                     AWLTQVDAARSSAIGRLFCIVHWKYELGLGAAASLFPLFFPLPLARPLYSPIQQPLIQ
                     IKK"
     gene            complement(356630..358036)
                     /gene="pino_cds_216"
     CDS             complement(356630..358036)
                     /gene="pino_cds_216"
                     /codon_start=1
                     /product="Hypothetical protein"
                     /translation="MDQDHMDQDDVDPRRMACLGDLPPELLEIISGFLVSLRDLGAWA
                     MATGLPIPAKWLCAAAQAHGYHERLVEAGAPLAVVVHLLQCGTLCPCTSFVSVAACGG
                     RVDVMEHVWSISGAAKSERDTCVNRLSHMYGYVSKSSCMREAVRAVINTDRRDILCWL
                     LAKGCRDTRSWMCTSGTVEKAIKFGLKKAYADVVCGGHALGRAHDKRICAGTCAWYME
                     KAIVKDRPRILAMLAAANCRAIDTLSNRHLGKAIAKGSLGVAQWLAGAIKGPVIVDNN
                     SMQKAAVRGHVGTLAFAHDSGLAECPPNALLDAAAKGRLDVLAWAAGEGTQPPARPLA
                     PWYGTHLAYAAAAKGRCNILEWMAARSDANRTLGVGVARKALASDMWTCAVMLHDRGL
                     APFGTWDALTTAARHNDIHTVSNIAWCGGRCDTATWTAALCRSQDDIVSFCTGNLAPS
                     ACKRPSTPLPVWTLRANP"
     gene            358506..359633
                     /gene="pino_cds_217"
     CDS             358506..359633
                     /gene="pino_cds_217"
                     /codon_start=1
                     /product="Hypothetical protein"
                     /translation="MSRMRKRPTSTVAGNAQKSQQHAATLVQEAWSTWATTESAMRTR
                     QMRHLWCDCVIKLRMDGDTNTDDSGNSSANDDIDDISKTITAHRVVLARWPYFARLFA
                     LTDPDGSEAYEGENGRTHYRHVYNVTLPFESSSVHTLVDMLYDPRRLPSAIHDGDCDA
                     ADLVDCALFLNADRELLLRIMEIVLDALLPPKVDDEAATAPNAAVGAFLLRALGSGLD
                     DEIKRGLVSRLAYLLSDDDRTTLADVFGSYYDPTRCLLLEPVAPGGIRLCCGLRDQRR
                     KVVFVTPEWGKVTMRVTCAPSPGDPTIGDVAIKIDSDLPRWHITVCIFKPFSDAVIAV
                     SASSSPVDNPPTASDDDSRRPLSPFTLWQIDLLPEEQTDGA"
     gene            360050..360583
                     /gene="pino_cds_218"
     CDS             join(360050..360083,360216..360583)
                     /gene="pino_cds_218"
                     /codon_start=1
                     /product="Hypothetical protein"
                     /translation="MQKTEPAVEPSSNPVVDGEAITRAESISTGQDAVVSQGCTFDSI
                     SGNPCNDPRLGFAALTLTDFSSGLNGQVAFTNETIWAIYEMLPWPNAVPVARDLAAGR
                     LVQSRRSLGRAAGDLASRRNRTRKHHFLNRE"
     gene            complement(361124..361849)
                     /gene="pino_cds_219"
     CDS             complement(361124..361849)
                     /gene="pino_cds_219"
                     /codon_start=1
                     /product="Hypothetical protein"
                     /translation="MSTPTGQQAAVATAVPPAPTSTTVVAAAPPQRSVPWGWIIGGIV
                     ASLIAAMVLTAVILAIVFEGRRRRPNNPASFMPVTPVGPSSRGVAAPVGAPLAAGHYK
                     IRWAPTGLYLGVNEISGNPAVLVAAGTAPTWTYAAATAAGAIGGSLTSSTGSALSTGT
                     ATALVGPVPVYVSRAGTAVATGSWAAARDPSGSATLPGTIYNAALGGCLRPNGDGGIG
                     SPIVLAPSCSATEHGWVFEPATA"
     gene            complement(362368..363834)
                     /gene="pino_cds_220"
     CDS             complement(362368..363834)
                     /gene="pino_cds_220"
                     /codon_start=1
                     /product="Hypothetical protein"
                     /translation="MMMATTPTADDDQAPPWATLPRELWREVLLCCPSDHDLYACLCA
                     ARCFHALTPTDLAARFYADATVEGMCAAGDMVGLEYAMVHRSASAPPIDWAACLYDAA
                     LLDHREIVQWILRQPDYCPVDVGGWEQARTVVAETDVLLLRALAALALLAAPIAASDD
                     PTDRMRIEDAMRRMEAVWVHAPLYAKDYTIERCSRLGGAWANLVMRFVHAANQAPVDR
                     TGANVCVSQSTAYQQCHVIGDYDGAQRAAEEVVASGLFAVDRLIREGRLDEAVRLATD
                     PSTPAKLPFFDALDSIVRIAEASARVGRIDLLDVLGCLDANGMAALDAIAQGSAQKAR
                     TTAFCEATAAGHVHILERLGAPSDLQTLVVLTRPDIVAVAVAGDHVDCVRWLCEHAFP
                     AATAKTWCASRARYVSALSLALLGRRKDMATLILAGVDGEAAARRALDEAVAAGDLRV
                     ARYIRAVCPSLLLCPPGVPRSRASCRPTPLVLIPINHA"
     gene            complement(364543..365826)
                     /gene="pino_cds_221"
     CDS             complement(364543..365826)
                     /gene="pino_cds_221"
                     /codon_start=1
                     /product="Hypothetical protein"
                     /translation="MSDDGGDARPAWDQLPPELWREVLLRCPSDYDLGACLRAAHCFH
                     VLTPGDLMARQYADATVEGMCAAGDLDGLEYTVAHWPAAAPPVDWSVCLCEAGIRGHA
                     HVIAWIVNYAGILHAPSYWEGARTIAIETDDPLLRAMASTMLSLAGQAPPTTPYTLGA
                     LSRFNEMWAQAPAEAKAVTVRRCRDLGQGWLHWSESLCTADEPPPDLERAESLVRLDA
                     EERACRTCGNADGAHRLARRLAEAVHPQMVNDLVRAGRLDAATSLMTNPAAHAGLPYH
                     IIMQVIANVARASAEAGRIDLLDTLGCFDTGTSARLDIVTGGRQAHVWTVVVEAAAGS
                     GHTGIMERIWGTPDAPTVVMPDSNAVAAAVVGGHVECVRWLCERGFPTASAKVWSSLW
                     ANQVSALSLALLARRTDMADIILASADADAAAHQP"
     gene            366433..368328
                     /gene="pino_cds_222"
     CDS             366433..368328
                     /gene="pino_cds_222"
                     /codon_start=1
                     /product="Fad/fmn-containing dehydrogenase"
                     /translation="MKKEKPSAARVPPPPDRPDSAKAARQQSTKRAHKEDEPEGTTKT
                     LAALPLCGRTDGGSDECSFVARVRSQCGPCKDPLTVEAISNTWCDILRDKGVSLECLC
                     ALARHVDVRYPWDDAYNTERLIYNKRVNVFPQAIVYAHGAEDIRRVLAWTLDHGVPFS
                     VRSGGHSPEGYSMTTGVVIDMSRMDSLLVSPDASEVAIGAGALIGPTYLRLQTGDTGA
                     KDTRGHGPGGLIVPMGTCANVGIAGLTQGGGVGFLMRRWGLTCDNLVAAEVVLADGRL
                     VRADADGPNADLFWALRGAGGGNFGIVTRFVYRAHRLRWVILFEVAFAWDDAVTVADA
                     WQRWAPTAPDRLTSQLTFEPAAGRVVVAGEWAPDDNNSGDDDDDDKPFDDTEDCTFAR
                     TNDDSSNANIPSAREREAITRARADLEKTLGTLRHTVGGIARKHGQKRPRPLGEGPRA
                     WVSSVLDAARHFTDNNARPPMSKIKTDFADRLLSRRALRRLVDALAVPPRVDRWCATA
                     PILTAVAFQAMGGAIADVQADATAFFHRRGTLFWIEYSAYWTAPEDAPGLVAWVTDAW
                     QSLRGAVSGYAYVNFPDAALVDWEHAYWGPHLERLSAIKARYDPTGRFCMPQGIPLPP
                     HIMRNDL"
     gene            complement(368629..368970)
                     /gene="pino_cds_223"
     CDS             complement(368629..368970)
                     /gene="pino_cds_223"
                     /codon_start=1
                     /product="Ankyrin repeat"
                     /translation="MNNIVWLPDEIVETILSHLDQVDAVAPRWVSRQWRRCSSCRYRG
                     APQNYLDSLLKNGHIETAKWARAHGCPWTRDARAAAAHAGQVETLDWLFRHGCARDDW
                     VCAWAKSKGIA"
     gene            369967..372704
                     /gene="pino_cds_224"
     CDS             join(369967..371619,371682..372704)
                     /gene="pino_cds_224"
                     /codon_start=1
                     /product="Dna pol iii gamma/tau subunit-like domain"
                     /translation="MEPNRRALPLARRQILGPLARPQAASAARLPPPVVGGPRQPLPV
                     QAPPLGRFPRPQAVPTVRPAPTQSRMPTARAAPQGRFAQASVGPAVRPTPAQPALQRG
                     PIQQAPPLGQFAVSRPTPTTRPVPTRPGRPTISGVAAAGAPQQTRAARQVVPTGRFAP
                     SPSLPRGPVPIQRSWTPALVSPRDDGCVAGHNQDLYALLRAIAEQNDDSIVGILANGR
                     VNVNEWIDPDIFGRADLDIHDRTYVEWPTQGYNQWTDQAGYQGEDERLLSDTDLATTR
                     GNAPDSLLGMAVLTGAPRSVETLIDAGALPRPTREFLLNSALSRLNASYYYTPGGDYV
                     RKPVDALGTAGVLLRRFGRSPRLHPLDMNPLSVARLTLQRSPNVILGNNEDAVRSVDA
                     VLVPLLAAGYSPDERIQAVGPPPVLVTNSRRRFEYTPARNRDYGSPPDYNAARAALAR
                     ITERQAIGSGGGEPIDIAQYTGPDSSLEVLSRLWDPLTARVADLYDSLSPPVQDVPTA
                     PTPAATEEGEERDEAESIVSLIESLPPEVVEMIAASRGLSAGDRRAAFMGPGAPCGDY
                     AGCMATLLAAIARDDPATVRRVLQSRVIGLNDLLDPSADTLYGNPLTVVTTQEPADGR
                     LGFGSIEIALRTNSIGRSYAGRRLLPSSRFRGNLGQRGWNYATPLALAVIAKAPDVVR
                     ELAAAGAQPWPSVESLLERALNRPFDERLFALGVDMTRYESTNALDRLIETHGNEAIQ
                     LDDDEPVPRAEQLGPNATVLIREADTPAVVRAIVESYPRSVRFHHGDANPLTVLRLFA
                     LESLNEPRFSPPNPTAIEAMLTPLIDVLLEAGYSPDERGLPCDVDPRGTTERALVAEW
                     AATQSAQSREGILADIFGRAYVLHDLPPLVSPDSL"
     gene            complement(373399..374829)
                     /gene="pino_cds_225"
     CDS             complement(373399..374829)
                     /gene="pino_cds_225"
                     /codon_start=1
                     /product="Hypothetical protein"
                     /translation="MRERGDTESNAADSKCGLALLPPEMVANVVSFLDGADHASCRLA
                     SRLFCVECAVALAARAYARRPRALVASQKAPLDAIVSVFAQWRREPDLPIVATAASSD
                     RAEVVRWALDSFESQWRRRASVTKESFKGDLIHNPYGSGADSKDSRDHGTDDAKRRVD
                     DACGLVQITTNAIAAGCTSVIPTLVCESWLLARGQKVLQEADMLADAVPTAPLSAVAA
                     AVNAFCRRGWDQPPGAVLSVGVFYAMGAGRADTAAWLHARPEIRRRDGQCMCERVAGE
                     RAFRLRRVDWLTWLDNVGCRGRYWPADDTVPLAVRMGDGALVKWAVASSRAAGVDYSV
                     HEASLAVAMRNGGYEALCALDETGAAPFASWPSLLLAVANLCVDVQAVRHIAARGGPY
                     GVDVLERAVTGGRVDVLEYLLGNDGPATAAEAAGVVHDFDHLLASYDRGWRWETAARG
                     LEWLRNHLTTVGTISHADSDNRESWR"
     gene            complement(375256..377213)
                     /gene="pino_cds_226"
     CDS             complement(join(375256..376750,376903..377213))
                     /gene="pino_cds_226"
                     /codon_start=1
                     /product="Hypothetical protein"
                     /translation="MDPVVRPDNVFNQRALAVQRDIRRAWRPVGRGDYTSWRDERIKA
                     VCAAESAVAQAHADVLILDALDCVDRDGVCDREALARVEFIYSYRAWQSIQADKDKDE
                     CRDPNDDGDNDDDGDDGSDCDGEDANDDNLANGDQQGRTKGERVDVSLKQMAAILARA
                     DRCAWGWAYGGHDAPVPWLWLMMRTMAECRVAWTTASLPRLLGLARALVEVAFQRYRM
                     DAHMLWTGIRPRQTDETTDVADDHSAWPHASHGPVIRVGDRYTEAACDADCIVQGLGL
                     GMTFGSPGDAHVAAMLGLVAFAAALWRIQATASIWQGALQPLGRRRRARSVQPCTDDE
                     ATRAAWHNATASDKLPLFLMDAVDHLVHAPDPMSEMGAIEFRYNLYLVGALLAKPDAS
                     AIEIRVARGSSRAITTLHLVPEDCGDALGLAALAIAHDLFSSITIHPSGRTLTWHDGW
                     LAAASRALAVEPIGGWLSRDHVQRVLCMAQTARTVIRARIAAAERNERARSDRHQGAS
                     IETRLHLISSDAPGSTVGRTPPQGLAPDETVAWTRGVALLDRDGMWSHANDVILADAQ
                     RKCAIATTHLDVLVRTTERLLALQHLIDHLPDTCA"
     gene            complement(377701..378863)
                     /gene="pino_cds_227"
     CDS             complement(join(377701..378186,378279..378863))
                     /gene="pino_cds_227"
                     /codon_start=1
                     /product="Hypothetical protein"
                     /translation="MDEECVETMHGDGSGCEAHDSMCTDAIARQASVNASHSVSAIDG
                     GDDDVDPREQVTINEVRTTGMLPYTREAIVRREDAHDRAVVRNLMPYIVDDCKKNALG
                     ADGTPLPPPEPLGPAGRDVVVPYLFDVASSLVDGAEVRACLAPDGVTWSAYRTDPQRL
                     VQPSHVGMAGNPWCADPDCPETRCKSCTLILAGVGMIARLRYEPNFMVETTTTRTVKL
                     KGSQGVKVTHGEGGMWNGGEDEAWYVMVAQNAVRDWGSGGGPALAAHDCVVGLAEARH
                     ALLRLDFAVRHWRLNARGGMTNPDYFGLKDYAKLRARREGLRGWIGAVEALLRNRTFY
                     MAKVAPYEARLIEAASSGVVSA"
     gene            379234..380736
                     /gene="pino_cds_228"
     CDS             379234..380736
                     /gene="pino_cds_228"
                     /codon_start=1
                     /product="Hypothetical protein"
                     /translation="MQAATTKCTKSNTVGGVDATDVLVGQAPVAQDDNARSRKDASTS
                     SLTGSSPEPVALRSAAEIRDAAAAFVASGERRRTLAVRSHLRRSMGVRASSVLGHRTD
                     RRKPRRSADNQHKGGDHTNADDVCADAAIVPPPGVNYVCLGTGEVLWLRMTLGAPEPL
                     LWSDLCDRTRKAFAEAAGLVLPTTYRDGRPVPSIDAADLDAVEPFVGLRSRWMPLLDK
                     AVCAIGSERSLNSLHEAVKTDVRNWVARNGAIARWAAQEPIVAPQPETTPTWDGSPPV
                     YKHSRFTRGHYAVQNTGRHFVSVDMRAASHAILLVEGLIEAPTWRDLVILAASVPKAA
                     AAVVVDNEDLLDYVVSLKKLRASALAVGSGHGRQMALMARVMAQLFERLVDSGVIVRA
                     DLAHFGRDELVFHVADFADAAAKISAIRVIVQDDRWAPHLAYAAYRMEEVGAEAIGYV
                     LVHDDGHWQLKCVDGAHAADYARLWHRRIAGTPSLGNGHTPPPPPLPHDA"
     gene            complement(380838..381964)
                     /gene="pino_cds_229"
     CDS             complement(join(380838..380855,381218..381964))
                     /gene="pino_cds_229"
                     /codon_start=1
                     /product="Hypothetical protein"
                     /translation="MPCEGDAGMHCRCVYASDDTGRRSRRHPCRAITRECSRMRAPGQ
                     LAFTSVCFASVVAAAAVVAQAWHDRGKDAAPVPSANTYGACDAGRRDIASVLGGDRVC
                     GRVWRGQPQSDPVVCHAAWRIDRWQGVTTTTAHFDASGADSLGAFEACGVACMRVDNG
                     GVALAWTQTYTTLLGQCEPPKNHAAARYAFRGAMTMDADGGATIRGAWSRVETPGADT
                     RPGDFVSGRFVLVLASPEADIELRLSFSPSYCWSYD"
     gene            382818..383279
                     /gene="pino_cds_230"
     CDS             382818..383279
                     /gene="pino_cds_230"
                     /codon_start=1
                     /product="Carbonic anhydrase"
                     /translation="MFESKTAGQTMSSSDVESVRYAVEILTPQPCVIVMLGHTDCGAV
                     KAAVEAVLVGAREAAGMPVEEAGPQRVYPTIVANIAPAARTALAEADADTRHVAMCGD
                     ADAVAAIVDSASVINTRIRAAELRLLLAGPDDRVPVLPAFYNVRTGRVRWL"
     gene            383685..384917
                     /gene="pino_cds_231"
     CDS             383685..384917
                     /gene="pino_cds_231"
                     /codon_start=1
                     /product="Hypothetical protein"
                     /translation="MSSSFALLPAWPPTMRAAGPPRRTVDNLARPRAWITSHSPWVPT
                     TTATVADNNDNNINDRLQTVPTTEWAQWTLYRRRPSSLASVTEYAVGSQERWPRSMRL
                     SLPVNGLDGSGAFLSGHGAHGHCGHLCQDAHGAALDLAPIARPDSVSHALAVAVCLME
                     AYVRFLFAHESSAESRGLEPAAVSRALNTVHPGIDLITHIAGSPWLGTLRNRAEAWYR
                     WITVPSRQGQGDLIGRLFAASPGFYGDGHALADDVAALTDRLACRGVAVGSQGLTSPI
                     PPTIQPLFYIKGAEDRRRLADDALGGLVNTDQLAAWVYRSNAATKAVLASREAHEPWR
                     ALSTTRCVVKSAVLAQRVHCLTLQTSLPYAFLWCPCSATLCSWVRSNRPSSRPSWAPS
                     PTRPSPTYLLTNVPINNP"
     gene            385170..385958
                     /gene="pino_cds_232"
     CDS             385170..385958
                     /gene="pino_cds_232"
                     /codon_start=1
                     /product="Ankyrin repeat"
                     /translation="MSRKGSWCGRPTQALPALPDNVLARILVGGLGDHHRRVAALVCR
                     QWRSVIAAGLGPGADLRIDPRTQGRLAADGSLDVLVWLAERIPRSDAHWHPIVRGAAA
                     GGHMDILDWAASEVARAYLDKALPYAAAAGTGRIDILVALDERGYRDLSGTTACTAAV
                     AGGHIDCAEWLLARGAALDYRAACEHAVAACDTNTLAWLCALARDRTEDGYDWDPVSL
                     MALASHPNVSAWLLQRARAAGATTSHHRKQHRRLHRDRQPVRHA"
     gene            complement(386325..386966)
                     /gene="pino_cds_233"
     CDS             complement(386325..386966)
                     /gene="pino_cds_233"
                     /codon_start=1
                     /product="Ubiquitin-like"
                     /translation="MLGRLGAVAGDMVFLEWDDIRSLVVVAQFDGSKYHLRHPNKDAA
                     DANDDAPWVVDISKPDGHNGWRNAALPVCCVCLDAEANCVLSCRCTAPSVCSACAAMI
                     DQCPLCRHSCLTDDVGRRVRTVYSIESHNPTDPWSYVFLKALDGKSCMVEARDGWSMR
                     TLKGLCAHRLGNPTVVFQRMLFGGRVLGDEGTVASYGLVREATIHTTLQLRGD"
     gene            387601..389105
                     /gene="pino_cds_234"
     CDS             join(387601..388391,388451..389105)
                     /gene="pino_cds_234"
                     /codon_start=1
                     /product="Hypothetical protein"
                     /translation="MDRLVIFFNAAQETVPLPPSGRYTLPEDAVAASAVAYVDGRGGP
                     TPVPFSVEGGAAPARVTVVKDGVNFAGDLISLDPHSVTLATGRGVTQTVEAYESVSVR
                     QRDRPVVAVAPDTSLLRDGAQVAFMREGLSWRPSYLIYLDNGPRYGDRDGGDDVANEN
                     TSIRQIVGVADIANRRDEAVAADDVWLAAARVPLPPPQVPPPRPRAAVMMAAQDVSEE
                     APRMLMASRAAAPSAAAYSPKVYGGDATKMQPRTTTTTGPERQTTIAPAEQAEVRFGT
                     LPTGPSAPGDWATPVVRGYRFVAQEPMPAGRATVFDPDMRFAGIADVSSTVPGEPVDL
                     VLGPSTDINVDAWVQVTTTYEPLAPVYNDIGDGPAQSIRSDDIRYSDDTDTGPDGPAA
                     RDRTRPVVARIVDRVAIRGTVHNGADRPVLLVLAYRPVFGGVVCASEPPYDRMTRGVV
                     EWQATVPPGSTDLEIDLDVDRGQRLLPGPSP"
     gene            complement(389465..390820)
                     /gene="pino_cds_235"
     CDS             complement(389465..390820)
                     /gene="pino_cds_235"
                     /codon_start=1
                     /product="Btb/poz domain"
                     /translation="MADRPDSDDQRRAKARDDSWLYTDGGRVFDSSVVGDLIEPARRV
                     LVGMRHRCCDCVVEMQDPRKEGVARVVAHRAILARAGYFDALFRHAEPDRVERRDPLD
                     DALVWRAVYKVDVACDPTSLAFLVECLYDVGHVKCVGDCDDPVDVVGAACFLQVPTEH
                     THRVLRRVLRMLLDDLADPNATEPRRPLAHFVRHVLASGLDTTVKTCLLGRVLGLLDP
                     SDRKAIIADHPDLVPKDYYRPRTEPGRKRAVTPDGRHWRLVRVSTDGTTRDGTTVVWA
                     DLAFRLDLHGWGRSSYAKVALSCLTAIEVLAPAPRRLRTLRASVCPRAAFIDARVYDP
                     SGPASLHEFQCDHATDDRDIWTRGAARYEQTARYEDLMGPVPKNCRLVPDPLFNVLSS
                     TDAQQFAHIKEAPALIFGDDLLACEVDVWVEINEDDGDRSKEAPTGGPWVGQRPRFLS
                     L"
     gene            391302..394032
                     /gene="pino_cds_236"
     CDS             join(391302..392456,392932..394032)
                     /gene="pino_cds_236"
                     /codon_start=1
                     /product="F-box domain"
                     /translation="MQAPPRQRADTKRAHIEALPLEMLVAALDKCRLVDAIRAAAVSR
                     SMATASGIAITDRRAAAATRWAHGRVLSDDMSDALTAAILHDDARIIEDMWLAGVLPT
                     TVGVRYRYYRERRSTRRGTTRRHVNQIISVHMSAPPPHGSGIEAVTLGLVYTALWTGA
                     VDVFTFVAVARRANGQLVNRTQALWFVRIGADAMPIKAVPDVRPRRMGHLYAAIASTT
                     TAPAVSVTANSRWSARSDDYEKPLTKAPLAVLIEVVRNTMVMCGRQSIDCDKGLPNQL
                     ADKLGVSVWQLERMVERAAGGRGLDHDAAVGILGAVASADESMGLLAGVHALVSMGWG
                     TRHCATARCTDTETVVFRAHGARPSGRPAHYRPGSRPRGAGLWRHPCCAVGMSTISLP
                     DEIALDIAQRLDARDLCVMSMLSRRFYLVARDPRLWRRLFRRDFPNLFIAPPGLSSAY
                     DALSVDDWPIEARAMYECASAGPHGLPTPSPVDAGLPPPFARALVSGKDWSWLYGAWA
                     GALRSHRRGRPICIGASSSTQGYHVLIKMPKDRSRVLRWIEGAPGWEVIRSAESDKYV
                     ISWFSLTHLHGGSCSWRGYGPCAIKAAGGPSNAETVEVYEMGQDARRVKFACTDPGRT
                     TLVRNHYANGDVQVIRYGRRERDRTGIEFTCSPQCSDPGFAGQRLDCTWRLHRVPDGS
                     PYCESILLPIHPSPDAPRFWRYVLLGHIGWTDAERQYALQCAGFPCVARDTARAVGAL
                     LGGASTVSVRD"
     gene            complement(394535..394867)
                     /gene="pino_cds_237"
     CDS             complement(394535..394867)
                     /gene="pino_cds_237"
                     /codon_start=1
                     /product="Hypothetical protein"
                     /translation="MKMATPTLFGTPLVLVALCATALLCMAASVDAKTCTFQKCFCTT
                     KDCDAFSSCTTHVQEEGACGSDNMICNCSTNKILKYANSGCTGSVTSYTSGSCYSKTF
                     CMEIISCV"
     gene            395239..396483
                     /gene="pino_cds_238"
     CDS             395239..396483
                     /gene="pino_cds_238"
                     /codon_start=1
                     /product="F-box domain"
                     /translation="MEVDEPTHATDLMGPSTQMPLNDLPVEILANIVGWLPGYALAAA
                     ACTCRALAALADDERLWKAAYRRDIDPRGPPLEHSDHAAYGMSARWVYGLMRAEPGRL
                     RMDPSGRLAGRIVASDGKQRKAGQFAPSIEADGKVALLPDGYTAMVTQYDNDDGRHYA
                     AEGRFTLDEQGKINGVATHRHRVKFDAAKHVSRCESTYRGPIAHGNYAGIGTIDYADG
                     TSHTAEFDSVAITGRCVSTRLAPTGAVYSGQCDNGHQVGYGVVRYADGAASVFCPSND
                     NDAVLRVKRPAPDAAPCRDSPSVLRARAHPEMQSRSLSFTIVRRRTPAHAVRHGHTRR
                     LVLGSRDPSRSRGRGRRKSIGLCRRVGPPSRPEPRWTALSRQLPCGKGSVGLGPRRTA
                     LFCRTRSPAQEEPRCDARYSRQ"
     gene            396491..397489
                     /gene="pino_cds_239"
     CDS             396491..397489
                     /gene="pino_cds_239"
                     /codon_start=1
                     /product="Hypothetical protein"
                     /translation="MDGNSAQVGDRGVGLPFGMVINGDAPCRGNETNAQDPTGGAWRV
                     RCFLTGLCVPAAECVLGHGGRFYHATMLSWWLALCNSADDDRPDQPNGWEPETGASTY
                     GTGSARIVWEPWMASVPPRLMAHCGAMAIRSMSASAWARGHVCQRTAQDLVRLSVLNM
                     AKVTSLLPCLDAPRGVEVLVAATATIAESSAMAGERPQSLSSLSPLLRSFDMVTLKHV
                     ELVHPTWDHVARGSMAHTRCPWMTQPWRRTSDGRAPTHPIAISPPTAFCALRSTGPPL
                     RARASRASFSLANALAKPLLSAPRSTGACLSAAFLAATVLSPARPCSIAICATART"
     gene            397931..398185
                     /gene="pino_cds_240"
     CDS             397931..398185
                     /gene="pino_cds_240"
                     /codon_start=1
                     /product="Hypothetical protein"
                     /translation="MNIDPCQQFTEVMADFLSIMREAFPDDGAPHCVRTLLPPPAKGD
                     APKPGRRHSMATEKVQTRRRRDTRHTRKATSIQKQRKKEM"
     gene            complement(398256..400436)
                     /gene="pino_cds_241"
     CDS             complement(398256..400436)
                     /gene="pino_cds_241"
                     /codon_start=1
                     /product="Hypothetical protein"
                     /translation="MQRRDQHDTILFGHDTRPPPSAESLCGLATVLKGLAKARAQNPH
                     MGSRMTIAKATAGAAEAIRRRFPGSDQVCDDWEQLVRRHAHVEADALGLRLCIESVKG
                     LGVAVHRVRVHDPNDLEGIKHLPIDLDDMRRRTASWRLPLATPSQPDPSQAESPAGQE
                     TQLVFSCSASSHSPNKGNHGRVADLPILAIEIRMGLGRRPWASAGHVINLATDTVIAL
                     GCTLVCDPDLDTHQDVRQDTICAQRHLLADFLNSGETAWRPFAARRYANVRRLPDALV
                     ARGWVVEDGDLAEWSWGGSAGPTAELSIGPSLSLSRPGVSVPVSVRMMDGHLIVSSDS
                     HWRYDDCTQIDWPTLFPSDPPAQAFYSDVGFVPLHECRAPTYYGEENSHFDAVAARQR
                     ERLVTMGLVSPFAISGRKARFLGRTPTNKEDALLQTDGAKSDGDDGSDGCFGAHVLVD
                     ADASTMVDAIEAYVAQNMFGEYGPVDANSGQRLHGGLCDDLLDQAVASGRLHSGLAHI
                     TGARHATAEAIVHVTSSAPAVESHAIDQPHLVVNWRAKCIPTVLSASGRGAHDLPPVV
                     WAAALVQSDGRGSACVAVSVPKALTRALLQDKSDDEDTTEDFDAPPEFHLPGYAEVST
                     LEYDIEDSTTRWRRVLDRCMRVDPGYPPDLHPALVAVLDMERECGKRACFVPWAYHVG
                     TTENPVDARSLASWIIDCIAEIFGALDNALDDMVNLGPEPFQDN"
     gene            400821..403169
                     /gene="pino_cds_242"
     CDS             400821..403169
                     /gene="pino_cds_242"
                     /codon_start=1
                     /product="Hypothetical protein"
                     /translation="MSHAAEHLIDLDRPARDLAVLLGAHLSAPFVQNSRASVLHGGHA
                     LDVADDADGGDEVSHPNFVVLDADMIDEKGDRREHHQDMDVNDGSDNVGDDENGPNHA
                     DRLGTPNPQPGEIATDTPTQVDERIWGLLSKCPEGAQRPLSTWEPMVSMCLSLLPTHG
                     PALDRPRDAPDDSPTVYSTGTAAAALARFLPALAAHGLDVYDVVLHPENMDTVTSPWD
                     AIQPIATAYCGVGSDWLAHVECGRLWYDDVREGQELAGGTILMTYIVHRQTHAIETCC
                     GARLGDGHDRDTAKWFAFMRSGYATFGAFADAHYGPLLRAARVLGTSFGWSLKRDDDK
                     DGDDDNEVKQADDSDTLWGVRSNPYPHSNAFVRASDSAVVHVDWQWGSLFVGAKPEPL
                     GLAPEAYTKPPCDHRPAKVPRPPADHVAPHQGDLRRQRKAALQREHADRAYLVDCGLI
                     DPMHVAPVLGRVRSRGRHGVESFWPCTRVAFDPLVAMDAETDADWHRRLAAQMDAVAD
                     LIQATGPALLVNNQDLREAIATEASPQLAVLATPALQSPARFDAETLTAWTWAMLSPM
                     VRHSWRVASMHRHVRCGSRYVDPSRGLYVNWNMRCNFVPTSEADGLTHARFYGHLLIV
                     DDKDMDTDGRHTRSTGTNATADGTTTDPACAPTVAAYYALSIRDPRANATYDESSFAP
                     HRSKYFTDMDDDERAAVEAAVDAVGPSRKSRFARDEPHHIIDHYRRREFTPVPVERFR
                     GILDEGALRLDPDMPVAVAIVRAFDWLREAFDRHAAVFATEC"
     gene            403830..405053
                     /gene="pino_cds_243"
     CDS             403830..405053
                     /gene="pino_cds_243"
                     /codon_start=1
                     /product="Ankyrin repeat"
                     /translation="MAAVTDNSIATLDNLPDEVLVGVLCRLSCADIYSTAPRVSHRWR
                     TLARDRSAMGPPVCLGGHTPQHFRPCHGDTVPSDWLTYAHSKDCPRYRHACRDAVAAG
                     RADVVAILYGCNYPLGEAVALAAAARGDVPMLTYLRGIHCPLPGKMCVLAAAGGHLEV
                     LRFAGERACLWGRDTCKAAARGGHLDVLKYAHENGCTWDESTCTAAAKGGHLACLQYA
                     RAYGCPWDHYATMRGAAKRGHLACLRWAVEHGCRLGRWADLVLCDSVRRGHIDVVRYL
                     VDDMACPLSANATETAAESGHLAVLQCLREAGCPWTPNVCRNAAAGGHLDILVYAHEH
                     DCPWDIIDTCRAARAGGHKDCLAYALRHGRVPTAAGTQNVLSGLSLLVAVGVGALGVV
                     FYFLIVYVLLAVRTP"
     gene            405671..406582
                     /gene="pino_cds_244"
     CDS             405671..406582
                     /gene="pino_cds_244"
                     /codon_start=1
                     /product="Hypothetical protein"
                     /translation="MGAAHPVPSTLHDSAGHGADDDDSDDMEISRLTGFGLEATRAFL
                     MSCVDPVAGAVDADALAKAEYQHNRRATLRALQSVPGGAILYVGTLCRSDDGLLYAQC
                     ASVRRTGNDRFEFAVAPTSRRGTTPSCWTEEDIARGIGALTLSHVLYVTSRPAREHKA
                     VTDAVRALMDNTWLATAARALSSHPASEPWTADCILCMGPLLAFLADTCARRIGDATG
                     DNRPRAPSPACPTRADAAVRAILDTEGRHEMGGSVDASWHRATYLEQDLRGMLAWIKA
                     LEQTDAVAEHLIGERYRRASSPATTAG"
     gene            406987..407856
                     /gene="pino_cds_245"
     CDS             406987..407856
                     /gene="pino_cds_245"
                     /codon_start=1
                     /product="Hypothetical protein"
                     /translation="MGTSQSTGAGALAEASKNTLASWIHLDEVREALLGCVDPSTGTA
                     DIADILALEYAHNHRIASEVVALARNGRRLNVGVTVLPAVGISTRLTAEPCGRMSSVT
                     REGWQSTWTRQEVADAIAAASVLAPMALSDSYHYGYSEYGVKACSLWRRCWLPTAMAA
                     LRAGPVCSVWTADRIDRMGTVLRALVDLCAQRDSGLDSTRAALPAGVVSDRALDLADA
                     VFGLSKGTEGFLNSRPQIIPHVTDVQENLRGMLSWIDAMERTDVLFDHLLKHHHAERI
                     ARMADSDPRLASP"
     gene            408637..411366
                     /gene="pino_cds_246"
     CDS             408637..411366
                     /gene="pino_cds_246"
                     /codon_start=1
                     /product="Hypothetical protein"
                     /translation="MDGSDNGENHTDNDASRRPAKRARRDANEDGDDAHNTNHIRDSD
                     HDQDAPVTVDIEFVASESVRIIASGANVAGLCARSAYFAAMLAGGFAEADVGARGGSI
                     SVRLPAGRIDTNALGRLAGILDGTIQPTAADALGLSGYLTFFEAAPCLRECKEALAAA
                     LCWTPSLSPSSSSSTTSSPLPSQPPSQQSSSSLSLPRPVGPDPYAILALYALAVTQDD
                     AFAQWADLQSGLFTVDSVWRALTKKDGADDGSDDTPGPQEEIDLVAYVGCGDALVEAA
                     SRVARSDINGSPCAAILLDLVQDTPGLLAAAVADAMSDWFAPLAPARALLNTPFALSA
                     CRVTSPMGALFETTRADPGGAVVPCFVPSHEAFVESLVGDSPRIVGAILSAGVLGPNT
                     VLAGGAVVNAMQSAPLRHRLGDSDVDMWIVGDDERDRRRAFARVIGALFDALPDAHVT
                     VRGSVVTFVVDTATPQPAASLDGISIAQADNAVATHVGNSTDGENRPETTETLQVIYT
                     DVQTAHDVIDDFDLTHACAYYDGTDVCASWACVWSVVSRVTLPLPGVTPNPRRLARAA
                     AKGFASASPDVVPAPLSDVNAGPSDEGASDRTRGDQGDGVIAMPVVDRDSVSDSHNDN
                     HNSNNDNNNEGNSVIGRDVNLPGNDYGSLWGMASDIQPVAYDSAAALLAHFTHASMVS
                     NSVDGFPRLEIALQTDVTPLAAIARVRVPPLRTRDPMVCSRCSAPDTSPNAWCLGGRR
                     CAGVSDDLPGDRAYAITVSVTDDNEAVRARACRNRLRAMDAEIEALHTRLANGTDMNR
                     WGESVPWVPMIATIGAMDDGGDDDAKPTMWRTHLRYTKYTTIRDALTGEALVPRRVGA
                     GSYIAARLAVYGVAHNDICTTPARHCRLLLVYPPHLWHVVGAIERALAPPTPT"
     gene            complement(411987..413399)
                     /gene="pino_cds_247"
     CDS             complement(411987..413399)
                     /gene="pino_cds_247"
                     /codon_start=1
                     /product="Fascin-like domain"
                     /translation="MYTASHAATSKTATALVALAVLLCCIAAPIDAYRYTVFVGASTN
                     WTAPVGATDISVTLWGGGGAGSSSLECGAGGGSGSAILNRTVGDAQWAVAPSDVQWIV
                     NVGKGGVPMDDANYAGGDGGNGGETFVVAVAPDNTELFRATAYGGGGARSVYESTTDF
                     CRGGGGGGASSSASGPTPGGGIPGGGIDNDHIGSPSEGALVGDVKAGGAGSGYGYLYG
                     DLTQPFADGAAWSSPGRYWPGGSGRRTGSGYSLQLSWGGAAGFNGRGGHGYQFAREYP
                     PANSGSGGGSGYVMAPVGANGMNGPGADGGVIIEYNHPVAPTPSSTPSRSPTPSRTPS
                     PSRTPSVSPTPSAQPLSQLITLVSPISGKQLTPQEDGSVASLWVGVSYKEKWTVARLA
                     SGKYTFKGFNNRYLGANPGGWVRAEATSVGAWEQWDVVINAGNQWTLKSAHGTYMGTT
                     AAGVIYLNDNADLYWTKTNV"
     gene            413954..415087
                     /gene="pino_cds_248"
     CDS             413954..415087
                     /gene="pino_cds_248"
                     /codon_start=1
                     /product="Hypothetical protein"
                     /translation="MKGSPRDGADAANPEIGLACLPPELIGMILGYVGDCDFCRCCQA
                     SALFWVPPTDAAIETRKRRWRGCREPHDFCATGNTEALALLSDRGVTFDVRLCVVNAI
                     AHGHGKRILALLRCIGLVASGTVADGGICDEWRQKACSVAARNGRTDILTAEWHPRLG
                     SDCIIDSAIQGDSLAVFQWACEASGISPRAADVWSALRNGAVTILAHCRRVLIDRAWW
                     VHIAETAAHWSFGIEAVLLCMGDDAPPHAQSDICYALCGSAPLCDVETFYGRFPDAFN
                     EWCLSAAVDAKRMDVARWLCERFPTLGDRLADRLVGLPPIDRMFAVPHDCGASVKWLY
                     GTDLIGDVPQLVRIAAERGATGLAAYITDHELPFADLSSACGF"
     gene            415516..416208
                     /gene="pino_cds_249"
     CDS             415516..416208
                     /gene="pino_cds_249"
                     /codon_start=1
                     /product="Hypothetical protein"
                     /translation="MRAEDSTTNIDHFKSVFPCTVDPADVGAKARAVALAKPAHIERT
                     RAVSLVRIDRVNYDPAPWRQSRPHWAVDVGGDLYHLLAEPQVKGDLQSASCALAWVLM
                     HGMPVSERHPLGTTEMDHEQIIAALGHVLAAFDGDVILYTLFWHCGAFLRAVVAALCE
                     SDIEAAADDRGGSVVVDETSLRDGLFALGRRDWVAPIRDNRQSDLARSTAIIARTLCT
                     DKPHESAGCILA"
     gene            complement(416480..417805)
                     /gene="pino_cds_250"
     CDS             complement(416480..417805)
                     /gene="pino_cds_250"
                     /codon_start=1
                     /product="Amidohydrolase motif-containing protein"
                     /translation="MRPVSLVVRGATLLTSEPKSRTAMRVVDVAIRTDGTVASINDTH
                     TIWNRKATPKVQTLDARGMLLAPGLVDAHRLLTNNMPSVRRGGTEPTYDIVVARSVAR
                     MAEMVKCGITTACILAVDHPDAVAEAAVRVGMRAIVAPVARDHAKRGDPTVLASDDLD
                     RIAAFARRWRPHPLIRAAVGLCDLSVYRQAYVGGIVWGARDRGLHVHACLNGNGSDDG
                     VTQPLRSGQQTPSVRLAMMALGARPGTVSVVVRDATTADDLHVMDTADIGIVYDAMHS
                     LAALDNHTSTTAKTATTSATMPDPAQDRATEKRHVRRLPTMALSMGAANPFARMRAVL
                     STRSSLDASQMVEMATAGGGRVLGLDRCGVGVLRVGGVADMLLIDPRRLGQSLANATF
                     CRAVASIVQRGHPTDIACTIINGRVVAMNGRAMLVDECIPDRDMFSCCS"
     gene            418199..419395
                     /gene="pino_cds_251"
     CDS             418199..419395
                     /gene="pino_cds_251"
                     /codon_start=1
                     /product="FO synthase domain"
                     /translation="MHSNKYKAGAAVKAMAASWRDSTMARIADAVLANRPLGFADGVY
                     LYRRAALDDVRTLAAYRKQTRHGDHVHYARSLRITVGHGRNRTPLLRRVQPPVASRTI
                     DIDAMATPADMERLLRPYVGVGLASVTISPHAAAQGSSAPPATASVDWWCALFKAIKH
                     TLPHVRIKACTPADIVNMADHHGITLDATLSRLCGAGLGLLAGGGIGDVSAAKNKNDN
                     DDDLGGVKHALCEWIAVHRRAYDLGLDSDAILDYDRTPACKQRVLHLLAIRDFQQETL
                     ASGRPAFGRLGLVRRRVRSYRARGASWDDDLRNYAVARLMAHNVEHIAVDPTLDMDRA
                     VGAIGYGADDLGGYASAGAVDALVRRLYDIGAVPIQHGRTEGPSKQHQHPHDPLADTM
                     WPIVPS"
     gene            419711..420897
                     /gene="pino_cds_252"
     CDS             join(419711..419904,419994..420897)
                     /gene="pino_cds_252"
                     /codon_start=1
                     /product="Ankyrin repeat"
                     /translation="MAQHLDLPDEVLVAIFSRLPCVVLRQTVRAVCTHWAAVSGDRAV
                     LVNATNCFDGDRGGLYKRGRCGGKRRSHGDAGAPAQRHCAWDSRVCREAAAKGRIECL
                     DYARRHGCRWDESVSEAAAAAGHKGILERLLQDGCPYDETACVAAIAGGHSACLALLC
                     RYGAPLDEDVYGEAVRAGSLACLRVLERYGCPRDPAAMTVASGLGRVDIVDHLWRHGF
                     EWDRQMSVRAVACGHIPLLAYVHDRGGVQPWAYLCRVAAERGQVDMLAWLHEHGHSWS
                     ARTCAAAASQGHLDVLVYLYQHGCPWDGQVYRVALAGGHHACVKYARAHGCPDDVDGS
                     AHGAVLLDAAGTGSPQRKRRRRSVAESPHPG"
     gene            421296..422138
                     /gene="pino_cds_253"
     CDS             421296..422138
                     /gene="pino_cds_253"
                     /codon_start=1
                     /product="Hypothetical protein"
                     /translation="MDEAHGRRIERQGEITRALACDDAARLASILAASDLGFVDTIDL
                     PVVIAALAPSGGVAGIVSPARMSSVVARDDLSRACRSATSARLPQGDIAPLALALFYG
                     AHRCFDHLVANGAQMSAEDAESLLVHFCATHAWREAAVCWGPCRLLQSRRDRRLVSPM
                     IVAAIGGGDRGSGSRVRFLDPLPVAQRLMPFLAAVTPAEEPKMRERLMAAVHGSVVTL
                     VQEGGAAPDVAAIDRVLTLVDGATPPLSRLLSRSGDIAALSDNDAATADSDSTMERAH
                     DPNQ"
     gene            complement(422377..423942)
                     /gene="pino_cds_254"
     CDS             complement(422377..423942)
                     /gene="pino_cds_254"
                     /codon_start=1
                     /product="Kinase domain"
                     /translation="MCLRLCRLCMHGHPLVLGMRDYPLRMVALLVAVCASVLCAPSGC
                     RALDRSALGCLCPVRPFCRRRRTVRCAEIAINDTANNTACAACRPDLVLVDAAPASGC
                     ITNVVAPIVVHTKTDLVSRVANSSDHVDHESTQRDRLAIISHGDNETAVARATTYALG
                     LIHGPQRHRQRAVALVVCASDKTMTAISVERVGLGRHFIQRTTSPMFLGTAPISLTDK
                     TIGHNGPSDIGRNDGPNDKNSLNASLYDKSAHTTTNDEPVTDGIVCIRAHSHNYPESM
                     GFVRTAGGSALALQACGYLGSGTTARVFGVRGRNDLIVKIMGDPAVARHEARVLTRLT
                     AVADVPKLVRANAETLLLWPRAVDWAKEPFGIEHALQALDVMTAAHVVGIVHRDFRPA
                     NLLRTTDGGRVLVNDWGFAAEADAIEPHRGTLAYASDAVLAHMIAGHTQFGVARADDL
                     ESLVRTLYVMSGRSVAMGMPAGRDLAARAARLMRMWKKAMTRPWRRLQDLARAGDARG
                     LRDALPRVLTGAP"
     gene            complement(424538..426475)
                     /gene="pino_cds_255"
     CDS             complement(424538..426475)
                     /gene="pino_cds_255"
                     /codon_start=1
                     /product="Hypothetical protein"
                     /translation="MNIEQLPLELLCMILNGAAPRAGGSGAQLWRARPRRPRPFLDPR
                     WRFAARGVCRLWREVIEHPSPAQATAMGTYLRSDLDAPNIGDGRRVDCPKWPTGRVVC
                     ASAVAEWIAQRPDTWSRSRLDNVYTWCRDNAGATRHQVIVALVASDTPEAVAYALDVE
                     WSRLPFCATTKDDVASKSCSHADNPYWCQGRCSAECGLAQHVARAVLACGSVATVNVI
                     LSCNLHGWPGHLVRLIGPYGRTDVARHLKTDAPQRNDWIAAARVKEPNYFAYLIDTLA
                     KSTAEHRALYAPPMPYCPFTWTTGYAGGCVEGAAAAHGRWRFFALCDARGIKFDACAA
                     FGVAARRGRARLMDWLWRRDASGPRLLPLVLHDAALLAVRDDGPYPKNRCNRAADAIR
                     WLCEVADYRPGDRRQLAALFIGSDDRQGRTVPALLYLVERWPRTAMELDAALLRRLFC
                     QCAVAGGVAMSRFMRIVGLYCPRVDKPGVDGWQQVDPASFDLWGALTRMCTQHVSWSE
                     ALIRAKAMLRMMRVCRAVALGRPPHAVDVHGLQRPCACIVQPGWSHTLCALRHHTDAA
                     RLCADDNTPETDPAVRAGLTPLAHWCMPRPVCAADLFDSIYGHNGDWLRASPNTFAGR
                     LAGVFQCILAWLASEGLLIDH"
     gene            426927..428360
                     /gene="pino_cds_256"
     CDS             426927..428360
                     /gene="pino_cds_256"
                     /codon_start=1
                     /product="Hypothetical protein"
                     /translation="MEGTLAAPPIDILSDDALLYLVDNYLDDRSLGACLLAWRRFHVL
                     DRTRLDRRKYRLATLLSLCAAGDMDGLDYALKHLDVFGPPVRASRRIDCVRAAYHAGR
                     ARMVLSLMRDVGDSIERFSVQEWSALALKAALRGAADPELVWLCRQENQPAEPWDGAA
                     LVCACTHAVQSGFSAEAISTALGAIESLAGLSVAVSHDTWLRLSAQRAAHSKSNLDTL
                     LALVTNAAEPHHDIQGHEMERLVKRSDLALARDLIGDQRLARFLRKSHECLTVYRPLD
                     DVDVILWLYDHVDSVADFIDTRQVGIHHLVLAVAASGRANLFNAAEIRAASHENEEGK
                     PLSHMWERAYAEAAVAGHATAAEWALGHRMEPAHIRAAFWKQHWDNCLGLPARYGQTS
                     QSSRTLSPLVVHRDRHDLFDLLVDRRRREGIPQDETDARVDHMVDITVKDALAQGDLG
                     LVRRLYLVEPRLVQAAVDQERVYGARS"
     gene            428660..429610
                     /gene="pino_cds_257"
     CDS             428660..429610
                     /gene="pino_cds_257"
                     /codon_start=1
                     /product="Hypothetical protein"
                     /translation="MSLTLYHTDAYEDDVAMDGDFAAVWATALDEAKQEAADYDRALA
                     YRWLDDVTDAMVDPDGFAGDDDDGNNIEPDEWKWHADGYRSGDEDDDDFDEDPNDGVG
                     YVVDDWDEIEPDRPVTFIVGPAAGTRPRDARLVTLLDPGDPGRGTARRWAIWTSQGNA
                     TAPTGPPTALLGDEQVADAVVQRLRAGSDVRLHNAAKRNKRAVAGRTARFLLDAAPRP
                     WQTYALGLADTLSDAATSGLDSVRDTAPGINALRASLDDPDARLAAAARVVLARDALY
                     NSPRDIDRDARAAEKVGIVKGWLAAVRNMDPDIDAVLARP"
     gene            429854..431299
                     /gene="pino_cds_258"
     CDS             429854..431299
                     /gene="pino_cds_258"
                     /codon_start=1
                     /product="Hypothetical protein"
                     /translation="MHQGCDDDDTGIGGMFDHEQPPPIDILPDDVLIYLVDGFLDDRS
                     LGACLLAWRRFHVLDRGALYRRKYRWTTFFSLCAAGDVEGVDYALGRPDLFALHDEPS
                     FWRKCLELAATGGHTDLARRLALQSANVWPLDRDYWLRVVLRLGQSGKDDTVAWLCRK
                     DNRPAGSNDSRWLTAYMVIVIVYGYHPGDAHRVVRAAWTGATDVPGETAWTRLHDVAQ
                     GPDVGLVAAAITRILDGSQTTATSRTVILCLERGHFGLLKDLVGDARLTAVLAHITRR
                     RHALPLVPRDDIDSALWMYEHVPSASQDLVPDDAVRLLDAASHFGRIDVLDRVLPFMR
                     LRWPETATGALARAYATAALMGHDTFVERVLTYDMDTTNLDTVFEACSRIIGTRFDGA
                     RSPLVIHRGCDDLERVLLDRRPPVGIPQVQVDARVDHMVALTVHDALDQGDIRTVRRL
                     CARSDRDRAIVEAMILRLQTDAITEADAVAT"
     gene            complement(431681..432064)
                     /gene="pino_cds_259"
     CDS             complement(431681..432064)
                     /gene="pino_cds_259"
                     /codon_start=1
                     /product="Hypothetical protein"
                     /translation="MEHETDSFGVWMRAMLARHRNTLETMGVAPPPNNNAPSPYGAMD
                     ALASHVAGTGRTALSAAGTSGGVDFAHSTVMRAGLDALDALYADPTATRSTPGHSRRV
                     LGQRPTVLFDHRRSRRPSLRTIAKK"
     gene            432506..432796
                     /gene="pino_cds_260"
     CDS             432506..432796
                     /gene="pino_cds_260"
                     /codon_start=1
                     /product="Hypothetical protein"
                     /translation="MTCAPDDDDPQWWMTPIDILPDDILVYLVGTYLDDRSLGACLLA
                     WRRFHVLDRRALSGASTDGRRLRASVSQATGTASTMPWNAPMSLGRLIPIHG"
     gene            432847..434001
                     /gene="pino_cds_261"
     CDS             432847..434001
                     /gene="pino_cds_261"
                     /codon_start=1
                     /product="Hypothetical protein"
                     /translation="MATMPGTPWPLHFNMWVELLFDLARCDHSDGLAWLCRADNRPCG
                     WDDALLASNCANAMRDAKHDAATVIERVFGAIEAAGESRGAQQHQTWRLVAAKCRDHP
                     PQPRPPLKAAVLVEAVREIFCLAGQEEACFTDVENVNELVAQGHVCLLRDLVGIDRLV
                     ACLRYIDSRPPDPDDALWVYDLVDRPNNNRQPKCLFDLLDVAARTGRLDLLDRVESDL
                     ALLSSPYKGRAKRAFERAYEMAVASGHALFVDRILAHRLAADAERCSFGGPALSGFRF
                     VSMSPPFIHHEQSDLVRVLLDRRPRPGIPQADVDARVDWMIAAAVEDASRAGNLAAVR
                     WLYSLAPAVVEDRILRLRGHRRRANVHPERPDTVFVQLGGLEDADAAVDI"
     gene            434417..435910
                     /gene="pino_cds_262"
     CDS             434417..435910
                     /gene="pino_cds_262"
                     /codon_start=1
                     /product="Hypothetical protein"
                     /translation="MACLPHDDRPVGPAPIDILPDDVLIHLVDTYLDDRSLGACLLAW
                     RRFHVLDRRALFQRKYRWATLHCLCLAGDHDGLDYALEHLAVYGESDDLATTSSLVAM
                     AGAGHVTMIERVVRMHDMEWPVDCKLWSSLVFALAKGGHGDALAWLCRADNRSHEWDE
                     DAIATECRNIVGHAHGAEVIDRVLCTAEASGDSRATAQRRIWRLVATQQQPPSSSSSL
                     PTTKQMAAAVHGVIELCNQVQNFASCGYDGICDMIREGHMGLLRDLVGPDMLISSLRH
                     IRPRPPDPDDALWVYDNVDSCDRKRDGPFHLLHVASRTGRLDLLDRAEADLPLFSSRP
                     GHAERAFARAYVHAAVSGHAAFVDRVLTHAIDMPTVNGSFALYRHTPPVVDPPGRSPL
                     LIHRGCRELVEVLLDRRPRDNVLQDDVDDRVDHMIALTVRDALRTGDLAMVRWLWPLQ
                     SDVVEAMVALLRGQDMDCVSQRVRRVIDPFCQDAQTSHDERLYGGFY"
     gene            436636..437505
                     /gene="pino_cds_263"
     CDS             436636..437505
                     /gene="pino_cds_263"
                     /codon_start=1
                     /product="Hypothetical protein"
                     /translation="MGKGGDKRYTCKIKVVLPAQRVPCPRPCPCPPFIPPIIPPPPPP
                     PTPVTTVITTTSTFVIPAGATAATVYLVGGGGAGGGSPIDSTGGGGGGGSGILAFSNI
                     TAALSPVTPTVLTIAIGAGGVGTAGDGAAGGATTVSFAGTGGPVLLQASGGTGGASGA
                     TTSNGGSGLTAAVAAPTRRAHLVALASWVWAAPTANQARQRRAATAAAQMRAPASWPE
                     VVAVAAPPSHPLRPSRARAALARAPRPRATAVPAVEARWSAAAPRAVRARPAMWSWST
                     RPPPSRRTRLSLT"
     gene            complement(437896..439673)
                     /gene="pino_cds_264"
     CDS             complement(join(437896..439441,439567..439673))
                     /gene="pino_cds_264"
                     /codon_start=1
                     /product="Ankyrin repeat"
                     /translation="MATFDDLPNELVAAVLAFLPCLEARRAAAPVCRLWSIVRTRRHG
                     DKAVAARAARPELWRYATEPVCARGRSTSRAAIRNGSLACVRYLDEHGCPWLRDACRE
                     AAACGHLDILQYAHSRGQKWDAKACLWAAAKGHLACLRYLHENGCPWGSEAAHMAAKG
                     GHLACLRYLHENGCPWSYKQMTVAATWRRLDCLIYAHEHGCPWNKNIDCLAAVRGDMA
                     LIEYLCAVGYTWTTHSRSLVSRYATTSAACNGHLDALRYLHDHECPWDATTMEYAAMH
                     GHLDCVIYLHEHGCHAGARALAGAAGNGHLAILRYLHEHGYQGDATVCRQAARSRPDV
                     LAYAHNMGCPWDEQATATAARCGNLDNLRYLHEHGCPWNETTTTDAARCDHLDCLVYA
                     HEHGCPWDEALCTQAMYTPASMPCIEYALTNGCPCDAQVLAIACARGLPLIERLRDLG
                     CPWDATFCAAATLRGKVDVLAYARQHGCPWDSTAWRGAFYQSNIECARFLHEHDCPWD
                     VALLSAVRPFAAVEAYLVEVGRMRPRDFSVHLPATTPTNSPG"
     gene            complement(440241..440477)
                     /gene="pino_cds_265"
     CDS             complement(440241..440477)
                     /gene="pino_cds_265"
                     /codon_start=1
                     /product="Hypothetical protein"
                     /translation="MPRKRHDLDLPGSRANDGPPATTIQIIQNNGYRTEFTSDGKTHF
                     VLLDGDRFCLEDDDLDLDAVLVARDQYLSSNPTT"
     gene            440977..442374
                     /gene="pino_cds_266"
     CDS             440977..442374
                     /gene="pino_cds_266"
                     /codon_start=1
                     /product="Fascin-like domain"
                     /translation="MHAPYSSMGAITLLLVLVAIASPAWAYRYTVFLPASTTWTAPAG
                     ATDISTTVWGGGGGAAVSGNCGAGGGSGSAVLNRTVNTAGWGIVPSDAQWVITVGQGG
                     AALPLDGGTGGQASDGGETSVVVLAPNGTQLYSVVAYGGGGAKAVWPATTGCRGGAGG
                     GASSSAVGTIPGGGTPAGAADSDPLAAPAQGAIVGDVKAGGAGAGYGHANGNLSVPFL
                     DGASWTSPGRHWDGGKGVAFYGRLLSWCYAWGGAAGFNGNAGEANSAGTVYPPPANSG
                     SGGSSAVSCGSGSAGSNSAAGAAGGAIIEYNHPVGPTPSPSVTPSRTPSATPTRSPTP
                     SVTPTPSAQPLSQLVTLVSPISGKQLTPQEDGSVASLWVGASYKEKWTVARLGNGKYT
                     FRGFNGKYLGANPGGWVRAEATSVGAWEQWDVLINAGDQWTLKSAHGTYMGTTVAGVV
                     YLNDNASLYWTKTTV"
     gene            443292..444741
                     /gene="pino_cds_267"
     CDS             join(443292..443819,444047..444136,444292..444741)
                     /gene="pino_cds_267"
                     /codon_start=1
                     /product="Wd40 repeat"
                     /translation="MALACPPTTAARTGLLSLKFNQDQSCLTMATAKGFRVWNADPFT
                     LRYERDLGGGTGLATVLFRSNIVALVGGGPNPRFRNDRVMLWDDCRNQSIAELCFNST
                     VRGVEMVRDAIAIALDERVYIYALANLDLVKRVPTVNNPRGLVDIRASEGGTDNVIAT
                     LSTKAGYVEIHHSDNRRPLVVRAHEMPIARLSLNADGSLLATASEKGTVIRVWNTTSG
                     DKRAELRRGKDTASINNLVFSLDSRWLAVSSDRGTVHIFDLDTPPPDAPSGLLQYIGS
                     TGVLGAGVSEYSTGWFSKAKIHLPDSAPACVCAFGRTPGRVFVVDAEGIYGVYDFDVD
                     KGGEARVVDRFLFGNEDREQA"
     gene            complement(445116..445820)
                     /gene="pino_cds_268"
     CDS             complement(445116..445820)
                     /gene="pino_cds_268"
                     /codon_start=1
                     /product="F-box domain"
                     /translation="MDLPDETLLVIMEHGGAHAVGYLAQTCWRLATLGRDDALWRRLC
                     SLDPWELAWEALDCVPHPRRGWRWLCRAHAICVDLTAPFTVGWGYIPYALETRYAGEW
                     SYGTPCGYGYGTYQLHDGLPRLLQGRWKDGVPHGFCIQYTKGREVGRGCYRNGERHGM
                     WKVEFAPGWSYEGPFRNGLHHGMGVERYADGGHYRGHFYKGFRQGQGTLTLPDGTTRC
                     GRWKGDSLVVALPPAL"
     gene            446504..447784
                     /gene="pino_cds_269"
     CDS             446504..447784
                     /gene="pino_cds_269"
                     /codon_start=1
                     /product="Fascin-like domain"
                     /translation="MTMRKSYGAPLVGLALLALLCCTATVVVDGHRYSVLLGASQTWS
                     LPADATNVVVSLWGGGGGAASTVQCGASGGSGSAIIARALGDASWDLAASDAVWTVDV
                     GQGGAALGSILDLVAGNGGQTSVVVKAPNGTALFSATAYGGGGGRLLMSGGRRGCQGG
                     AGGGAASSASGVVRGGGTPSGAANDNPLAASAQGAMIGDIKAGGAGAGFSFVNGDVTK
                     PFARGASWTSPGRQWEGGTATYSPPCYTWGGAAGFNGNGGNGYVASYQVPAANSGSGG
                     GTAYVCPPDRYNADSPGAAGGVLVDYTHPFSPTVILKSSVSNKYLTAHSYGGVSANAA
                     VAQGWERWVGVRLDDGRYAFRSWQNKYLKVNPTGSVEATAAAASDWEKFTVVYFSPTT
                     WSLKSYHGTFLVAAADGFVNAVANPGHYWTVTEV"
     gene            complement(447988..448878)
                     /gene="pino_cds_270"
     CDS             complement(447988..448878)
                     /gene="pino_cds_270"
                     /codon_start=1
                     /product="Hypothetical protein"
                     /translation="MRAGAKVAAGHAICRREIGSASAAVLGRTSDNTLAYLTLTAVYD
                     YRPSSDRTEYLDAQGRPHNEARGTLCGKEHATAAKHAWWPKHLHVSTTVGTLCRVVAL
                     TTLLGFDARAIEARGGGRLLAGAAHERASCVLPLWGVTSIAMMARAWVAHGANSPSAL
                     LCPRLWCPLWSPSTTMRWTVSLGCPIAPTPLAWSTFAQRRPCATTRSDTPSPPAWTPT
                     LCKARAIWPPTSGAQRPCVGLPAGRTRPTARSPSATTFATPEPFRSLFFVSPSGGSFF
                     PCKKRETCESVRLKTCAATG"
     gene            449392..450387
                     /gene="pino_cds_271"
     CDS             449392..450387
                     /gene="pino_cds_271"
                     /codon_start=1
                     /product="Hypothetical protein"
                     /translation="MDSLPSEILWFLLNGALSTPRIATRGWVPPGRRDRVRPFLDPRW
                     RFAARAVCRLWQEVIETPTPSEIARLHRHRSKRHGADGEDGHSWHCPKWTTGRLVCAS
                     VVAQWIATDTGPWSHGDADTVWAWCTAHARASRKHVMAALVASDASWAVDAAMTTGWI
                     CASFADDHGDDANVNGDDRALHSALDCVDQRGDWWDDQRGDVQGLRDALWDIAARHAS
                     YRTLLALAAREPASHRAHALGRALDRACRAGRADTVRALLDNGVRPESAAWTHAARAP
                     NPDCFVVLLDHAPDGPPPMTTTDAGDDVQSVNGDWLHDPSPPDAGAFSTYARRAA"
     gene            450399..451355
                     /gene="pino_cds_272"
     CDS             450399..451355
                     /gene="pino_cds_272"
                     /codon_start=1
                     /product="Hypothetical protein"
                     /translation="MTAFLGAARARQTKVLAWLWAHALTRPLAIDLAVVAIHAVGPHN
                     RPRTRSADSLAWLCEVANYVPSELDLAALIARACANRCVECALYLAERWPHEALSLDA
                     ATLGSFFRACVCGGLSTLGRFLAVVDRHGTTLGADAAHRIDLWGALAMARIDTSRRWT
                     HMPFVVACMRAAHDIAHGRPPRAADIAQIDSLALSTTTPLPCACMHTKPTEAAAGTTS
                     HGLMRNTHDDAILCTDTAVLTALAPLATWCRPRPVSPDDLVPGWRRTSTVPAGTLDRI
                     THGGLCRRAVDWLASVGLLVPHLPPPPQTRAIFPPPVQIVSS"
     gene            complement(451575..453629)
                     /gene="pino_cds_273"
     CDS             complement(451575..453629)
                     /gene="pino_cds_273"
                     /codon_start=1
                     /product="Hypothetical protein"
                     /translation="MLEPIASEAVCDKGDGKTKSPAVAMPRVAAAAALLSEEIARRIA
                     AIPKKQTAEGRRIEEAAADAALLRRCESATAHPVSAWTHVIDAFVAWLSRDRTDEKPN
                     ALSIAHGKDSDDNENDDGDRDIDDKDDETTNMLLSNDGARQPDHPNSAASTASHFLGP
                     LACMGIDIYAITTHPENAHLIGCCTHTPPLIEADCAYGDWRFVVTMERDVDCTGGILF
                     IERAVHAPTGASIDARCDLRCQNTSRGVRALLFSFFQSGQTSLSAFLHGVFAPFDRVA
                     DVLHAHGWDTGGKRFLWSDAHSALYPQRLACTRNDGVVVHLFWWSGTLVASGLEYGPH
                     IKLAGQPASAYGPDPDPDTALQDGDDNSDGDDNHDDDDGDDDFGFVGTKRDDDRAWLV
                     DNGYIAPWVFAARWGKRPRDCPTLLLDPTRGTDGDLIMRMNAVADQIVRQHGINAGDT
                     AARAYGHSRLNGWTCQAALNGMLTGTRSWRLAMRDRSIVSGERYIDPDRGLYANWMVV
                     CDMLPLGNRDDVTVRTTGLPFVRFQGHLLGVDERAASGPVQQAHVVVLLCEPLEPPVY
                     AHQQRPMPPADGEQSRVAAMIHALSDNGKDPKVPASDVALHPITAYYRQRCVDSHGGP
                     KVLAVLHEASSTPQGAGTSGEFVAAIEWLMAEFETCAQTFGANAADAVPSSSAWTLEI
                     AW"
     gene            454052..456007
                     /gene="pino_cds_274"
     CDS             454052..456007
                     /gene="pino_cds_274"
                     /codon_start=1
                     /product="Hypothetical protein"
                     /translation="MDRPEDRPIEVAGTFLRAMAGARLNRHTIDTGHAAATEADKILG
                     SFPPLDECVDDWAYQLHALAAKHIMPPTTADDSESEPPKGTTAVPAQSMRQERRLCER
                     VEIAACINAISAIGATVHAVSEGPDDAPVVRLHCSDGAHTFVLAIEIVLARRLPSDRL
                     GAHVAEPLVFLGTRRLLRIAGPLCVPVGKWKMGGGWRSTMSTNDAALRLLTDLVHGHV
                     DDWAAFADARYAQLAALPNALVRHGYTVVPDDQSAHRRWAWTDHEKPAELGGMALGIV
                     CPDGLQLWVVWKSGTLLITNQGYFSISCTALPVFFDQTPSTPIAMDDDDSDDNLDVDG
                     ASHATQDWWAEQDGADTDISLVHERTWMIRRGYISGNVVHARAQDLYDKGERPPLILG
                     RLGDMADEMALRVGAYVNYVRGAYGPVDETTGAHLNGPWCRRHLADTLAAMGSQGRLS
                     HYEGPIDPAGPVVTNSIIEYELDHVFGRVSGITTHDIPHVVAHAHLVGESSGMASAYF
                     SVIIRHCRYHDTFDSDDDDDNREDHGRRDRNKERSDTVSKSTTSTKPRRSSYRSLVAA
                     LKEIATGPAQEDDNNIDHAVIVHTLAERNARASRFSTRTAAVLYERHLSGDGVGLVRA
                     HAWMTDQFVTAIRLFEEASAAFVCPLP"
     gene            456748..458307
                     /gene="pino_cds_275"
     CDS             456748..458307
                     /gene="pino_cds_275"
                     /codon_start=1
                     /product="Hypothetical protein"
                     /translation="MTMTATPMELEDTPCERATSLVDMPVEILLAIARHLAATSVRDM
                     VVWSQVVGLDLPTQLLGTAMEDALLRLVARTPPTAKAVDLAHRKAVASGAPLCVVQHI
                     ARYDRSNDLLHAAVIGGRVDVLQWAWSHACPDRSVWYDGSWSDFRRALKDAMCHDRRE
                     VLIWLLERRSQDWYNMCEHYDDVMTFALDKGYADVADTVHRAAHEETKMRPFWKRCFC
                     GHAILDDAVRRGRVGVLVMLEQIGCHLTSTISDRHLAKTVRRGHMDAARWIAARLDTP
                     TISERDMGKAAVRGHVSIVAFVHDTGMGTCTPTVVERAVIGGHTNVLDWAIGRGQAPP
                     ARPIVPWYGPHLAYTAAEFGRQDVLAWMARQPDIAPTPTVGVARRAVARGHWSCAVAL
                     HDCGVIPLDTWDALGTVLRHATGTNVLDIHSIIKTLAEKGARCTPDVMLAALCHKTCF
                     VLPYLCERFGTSDLQAAVDAASGLDFGHESLAWVAANVRSVCVAQLAHQQRGMLNVTA
                     HTCCQCARCTP"
     gene            459009..460564
                     /gene="pino_cds_276"
     CDS             join(459009..459649,459718..460564)
                     /gene="pino_cds_276"
                     /codon_start=1
                     /product="Ankyrin repeat"
                     /translation="MACVDDLPDELLVLVFSFLPCTVAGLAPRVVDRRWRRVLTDPRS
                     TKKTPCVSGVDLGNTTTWCRAAAAAGHLLCLRHAHDSGHVWGRATCVAAAAGGHLDCL
                     KYALEKRCNWDRKVCYAAARNGHLDCLDYALDKAHVCLTSDTSLCAEAAAGGHVDMIK
                     YLRARGYEWDARTFAAAVAQDHLDMLAFVWSQDCPRRREGKAIAASRGNLDCLMQIDC
                     LAFLRQNGYPWEPSAVAGSYDQGPRRILGIAAKCPPLPPQRRGRAYDAAAFDARVTEM
                     VREDAARRLHSLRDVVIACCDAAALGRTSFLQLVASLYPRNRGRGVCEAAALGGHIET
                     LRYAHGEGWPLAPSEKLVAEVAGRGHLNVIRYFDEHGWIGDSKACQAAAAGGHAEVLA
                     YLRRSVFSDGWHPRYWWDQVACLGAAASGGRLDVLRYAHECEQCPWFVDLSRRAAAGG
                     HIRCLRYLCETGCPYDAETYASAVRYQRRACLDYLDHMDCPKSAH"
     gene            461205..462743
                     /gene="pino_cds_277"
     CDS             461205..462743
                     /gene="pino_cds_277"
                     /codon_start=1
                     /product="Ankyrin repeat"
                     /translation="MDVRWIFLKETHNETTATHTHTHTHTHDKHPLAFVGLEQNQWSE
                     LPDEILGLVFFFVPCSILSTRVPLVCSRWRRVSGDSAAVGRASCLDRLQQKRGTCWMA
                     ASKGHADCLARARARGRPWPLGAYCEAASGGHLAVLDYMRANGCPPDVRALAAALAAQ
                     RVEAVFWLCDRGHAWGEASCALAAPYADLALLVRLRERGCPWGKGTTSALAARGLLDC
                     LRYVHEEGCPWDDDTCVRAAIAGHFDCVRYARQGGCDWSDGHNLAVKAADAGDLAMLM
                     ALHEAGCPWYEPTASAAAASGSIECLVYAHTHGCPWGKSTTASAARGGHLDCLVYAHS
                     NGCPWDESTCRGAIQHRSFDCLCYAVVKGCPWAPCSSEITVMLKARVGMSVIKHAFER
                     SKHQNWAKQAASCGRLDVLKFVANGRNLDANVMEKAAAKGNLDCLEYAYRAATGWAVT
                     TNVAAKAAAKAAKHGHLNCLIFAHENDLGWDRKVIIKAMDQGHRHCAIFALENGCPLD
                     RQHE"
     gene            complement(462957..463214)
                     /gene="pino_cds_278"
     CDS             complement(462957..463214)
                     /gene="pino_cds_278"
                     /codon_start=1
                     /product="Hypothetical protein"
                     /translation="MSSGYGFDIATPMCVMWSLGSAQITYVACGPATFGLGLCRTVKM
                     AFTKRARRRHTLGGSWRPFAVATVTLVPVAGGIVAGKMINA"
     gene            complement(464218..465186)
                     /gene="pino_cds_279"
     CDS             complement(464218..465186)
                     /gene="pino_cds_279"
                     /codon_start=1
                     /product="Hypothetical protein"
                     /translation="MTDPDLWAMPADVSRCKPSWPATAPHLFDSGSAAATTSYPLCAL
                     VVLPDDDGRRFYGVLVCRPLVARRVIMRHCVVASADVTRGGHIFLPDPFDFGRNYRNL
                     WCLADTLSPPGARCSLSDLCRTTEALVRLACNAVACGLAPTLGTMPVLGRDLSGQLDV
                     NVIQRHVPLDGPSLYTVFLSARDIVMLSTKVLPARQRLYDWMTASTLENCAAQSLADR
                     LVDHGDADKLACIPMHLRCLVVTHALSQACTSNRLDPLCAIARLLDVDPTNKSPVALA
                     VTLALAVGALLPHPNKGLAAVLRQRPVLSVFLCRPRFLNCIFLDFY"
     gene            complement(465938..467608)
                     /gene="pino_cds_280"
     CDS             complement(465938..467608)
                     /gene="pino_cds_280"
                     /codon_start=1
                     /product="F-box domain"
                     /translation="MTTYDAGGYGWADLPDELVYRVALHSPLAAIAALARVDHRTRNV
                     CLDDRLWRRLYQRDYGSCRSTRGREETCHRAAAAGFRGGDILSCASPWLGITLSSDDM
                     HSGDAQPTTDVIDLGQWRPHDGSAIAAADAGAPHVWQRTPPWEWWALFDQGPPGCLCW
                     WPPDAPADHRWAYASLQTPGGLFGRFPDMPLRRVGCMREVKIGDWGRGYPRDVKMTYR
                     GEFDDECNPHGRGMAVAVGRCGIAPTVHRISGHWVRGRLHGWAAVWRGCSYFEGHHDS
                     GQRHGPGLNIEGMHEVVHGRWEGGLRKGSGVARTYTGTLIHYGPASGDRHDDFGVVRR
                     HNGSVAFAGRFAYDRPCNGRLYDEQGRLLYEGDVTSNFDARDSGTVYVDDYGVTVAAS
                     DWTKSPLPATITYGGGGGDMIDCTVPSFSMIAQHDPIVIRRFTYSAAADPGLAGTVLD
                     GPWQILGTTLVDSHDDVENQDDHRRWQQQAQQLQRPLDIVVTQVSDMPGASPYIKAVR
                     SLCDMVFWPRSDGTAARDAARRQFLDYMAGRYGSRWVTCREVAYATGW"
     gene            complement(468314..469813)
                     /gene="pino_cds_281"
     CDS             complement(468314..469813)
                     /gene="pino_cds_281"
                     /codon_start=1
                     /product="F-box domain"
                     /translation="MDQSETTIDALPHELLRSILVEFTDRRDRLVAWCVCRAWRTQLR
                     VGRRYCWKQTNWRLSLTKLARRSIAKGLCDIGFWLYDVCAKKNVTELARLLPQTVSAD
                     GAHQALRRQLRDRHCRWTVDDAKRAILAGDPHAIDEAIDDGHGSDAGVLYALLATGNS
                     ALVDTRCNAGNVPIPFDGIVVEAALNGRTDIIGWVRSRTGLDHHASICKGISWAAYER
                     NPSVIDSIEWATTDGAIKWTANDIKHFLGFDNVAVAEWIWTHQRQGAPAQDFFRSIVS
                     QHGNVNTTLWALDHGIGDPTVIGKRSCEWFMFRGDYVWADIAVGQLDRLYAAGLIDPV
                     PYDYTYMAHHKDTAHLAWLCEHVSEAYRDYWQNDHIWREIAMNGHPAHIALLLSKGFI
                     ISRSMLADATHFCSPSNDAIATIACVELLVNAGCALTPKSCLTLASLGAADLLRIAAH
                     EKKVPWDARACMKAALSRDSAACRRTAAWIGQEIGVDVGAFEQDLLRGT"
     gene            complement(470248..470799)
                     /gene="pino_cds_282"
     CDS             complement(470248..470799)
                     /gene="pino_cds_282"
                     /codon_start=1
                     /product="Hypothetical protein"
                     /translation="MSITITYPNGDKIECIAPSEPAGHPWVPASIVRFAFSPTCPDPL
                     LAGRTLDGPWHILQVGPRSPLPSLGYTSVSGPDDSPSDEPPAMIVHPVPLYTAILSAL
                     DVALDDLCAGDDDDIDNGAADGDDPYEIAPMRALDDFVFWPRAVGLDDDRGIDGNRFL
                     DHMATHHGRHWLTCRAFAAALPW"
     gene            complement(470880..471932)
                     /gene="pino_cds_283"
     CDS             complement(470880..471932)
                     /gene="pino_cds_283"
                     /codon_start=1
                     /product="F-box domain"
                     /translation="MRLQERQHRPPCSWDDLADELVFHVASFLDEVSVARLGCTDHRT
                     RLVCLDDRLWKRFYVSRIAASARFCAPKRCMVHRHEAHRDTVDRVRRWIADPSDTSCP
                     LDEWDRFVGASLSRCGHHMREPVDHRWACAMDRALSDRNSHKYSRRGFARYPDLPYRA
                     VGAVFKIDPFLLDFAASGLVKPVLDRDIRFMASYTGDTDPATGAPHGYGMAYAFSSKS
                     GEEACKMAGEWTQGRPNGSLCVWAKYKRGHIYYEGGYADGRPHGDGLLVTSRGAYDSH
                     WSKGERCGAGVARTPYARITYAATSRVASAKRPLFTATTDRSSSRASATSTAPPLTAS
                     CLIGLVSRSTGETFHS"
     gene            472337..473569
                     /gene="pino_cds_284"
     CDS             472337..473569
                     /gene="pino_cds_284"
                     /codon_start=1
                     /product="Ankyrin repeat"
                     /translation="MDFPDEILVSIFRWLTCREVAWRVARTCQRWRAVALDPMVCRLC
                     VLGFPYDGDAWPPVNKRDLCCRAAAAGHIDCVRDLTKSDTQYDCVLVSTAARHNRVAL
                     VRWLVDNKYAVNIDACRRAARHGHVECLAILDDAGCLWGEETLWAAARGGHIECVDYA
                     IAHGRNQESHGFKLCRGPSTPAHVACVKKILAAGGRADDYSEELAVRAGHHDCMRLFG
                     TGRSKWDDEMCAFAARHGDLDMLCYLHENGWPWEGYTLRAAAASENRDCLAYVLDNAC
                     PWAPDDLCRTVRSVLWTRIVTAVRPESGDTRPTTAAASMGRLDLLSDLCEKGYACGPE
                     TCTAAARRGYVDCLHYLYKRHCAWDHETPRAALTGKSRCCFEYAVSRGCPLMPVDRET
                     ADAHGWKTKVRLSLARPH"
     gene            474695..475438
                     /gene="pino_cds_285"
     CDS             474695..475438
                     /gene="pino_cds_285"
                     /codon_start=1
                     /product="Hypothetical protein"
                     /translation="MSSSDPITVPSTSLEPSADAILDSAAQSPLISAEPRPLPETATQ
                     VATREGQDASATVVDETIRNDNVTRARPMWACNCATISASVVATKSSTPVHPTPKPYN
                     LWGRVLWTVLLLAVLAGPTLVRHSFAPDSFTLATANQMPTVEPALGVGPLDASSAVSN
                     GPVDDDNDDAHRCYCMCPAKKTPVVGTFLVGPDGSRCVCACDQPRPAQSLFEDLMVWI
                     HAGAVAHGPLVLGIAAVSVLCYAATALVF"
     gene            complement(475552..477126)
                     /gene="pino_cds_286"
     CDS             complement(475552..477126)
                     /gene="pino_cds_286"
                     /codon_start=1
                     /product="F-box domain"
                     /translation="MRKQDQRSDMNDRCDWSDLPDEVVYEIATHCTVAALVVLARADH
                     RTRAICLDDRLWKRLYWRDFQPCRSTPGHETLCLVDVARAYAGGDILSCALPWLGDGR
                     VDAKHPLGRYQLSPSWEWRDLFAGGLSLCLHHWPPEALVDHRWAYASLLAPKGLFGRF
                     PDTPLRRVGRIRANPRAFGFADYEPPHEIIYIGDLDAEDKPHGWGTAIIVRGRSTVVH
                     RVSGQWSCGRVDGWASTWSNHSGQPSYFQGHHAKGEPHGRGLLITHSRIYDGDWQRGQ
                     RTGVGVARTTEALTRYGPESDSSRTRGVIYRHDGSVAFVGRFDGGRLCHGSLYDPTGA
                     LLYQGDLSTRLQITGHGTVYLADGTALSGYMGSADDRYRATITYPNGDTIHCLLPCIY
                     DNGPRDPVVVFQFVYSPVADDDLAGKVIEGPWQVLTTPHDASITGGPFGGRLDRTNVD
                     LSNHADIVTQFTDGTYKPDPQLDTMRALCDFVFWPQSDGTAERNMVRRRFLDHMAAHH
                     GGRWLVCRQIAHASQW"
     gene            complement(477830..479152)
                     /gene="pino_cds_287"
     CDS             complement(477830..479152)
                     /gene="pino_cds_287"
                     /codon_start=1
                     /product="Ankyrin repeat"
                     /translation="MADIDCLPPEIFYDVFGWLDRFDLTAAAHVCRAWGAMARCVRRG
                     ECQIAPEPFEYVWEMAGRGCLDALRWAWPMTPTGPSDHWICIEALKRAAAGGHLPTVT
                     WLVKACGAYGPDVPSSAARHGHLGVIEWLHAHGCPWDSYMCCNAAQGGHLHVIKWAMA
                     HGCQWNPWAAAQAASGGHTAVLDWLHDAGHAWDARVIQEAACQGHCDIVRWARARGCD
                     WARVTTSALADNAAWDTLKWAVNDGCTWNDRAAAAIARHGNVSMLQWAHAHGCPWDET
                     TCEAAAAAGHLDVLEWVRSEGCPWSEAVGDAAARTGGLRMLQWVLARGCPHSSRLFDV
                     AVLGGRLDTVQWLRANGHGWGETTFAAAVRGGSAAVIQWLHENGCPTAEHADGHVRAA
                     ARAGHMDAFRWLVAHGFRWDRRECIEAASKGNHNNILGWIVHHAGDCN"
     gene            complement(479683..480162)
                     /gene="pino_cds_288"
     CDS             complement(479683..480162)
                     /gene="pino_cds_288"
                     /codon_start=1
                     /product="Translation initiation factor eif-2b-like"
                     /translation="MGSIHSRRKHRPRWQGYRVDMWQHGVPEESVHETMSTDEAIAWM
                     LSKEVEAVFVFGGFGAFLLSASGHANKVAFFADFYRILGLSEEDTSDRAVYAAYNSAD
                     QEKKARMAQAYGTRMDARRWLDKVARWCAEHPGDPFDLRGNESLMGTIISRKEEWPK"
     gene            complement(480524..481084)
                     /gene="pino_cds_289"
     CDS             complement(480524..481084)
                     /gene="pino_cds_289"
                     /codon_start=1
                     /product="Hypothetical protein"
                     /translation="MLDTTILHAPAIRTDQDPVKDALDGHVGHDRNTVPRANDDDDLG
                     DAPRLGSDSCAGSPDLAEISSNHTLCHHATRATPMYAPSNKTVDHMYRHGLWLAVHVF
                     VVTAVVLLILYALPASFKTEWNLGVADRDTAGQVPRSSCECICDSPLPQASLWTPTAI
                     WHHVVTTLAQGNHKSVWDLFPFFYAK"
     gene            complement(481694..483494)
                     /gene="pino_cds_290"
     CDS             complement(join(481694..483232,483354..483380,
                     483456..483494))
                     /gene="pino_cds_290"
                     /codon_start=1
                     /product="Hypothetical protein"
                     /translation="MLLVIDWLLKWSKDMSRICAQFACLWRLGAATADFDLVRFAERR
                     LDRLLDPAIRERPFIADDEVSQCALSALTQKVSGSCPHHGRSVIESRGYRWAYASAAG
                     PPRLFAAYPDLPPRLVGHVRMARCSCTGNYRGDIKVSRIIPGAEPKYRAHGQGTLVGG
                     VYYHRESSVDAVRTGVTGKWRDGHVHGRAAVWYKWHHYDDGNRDGNDLNPARQEPGRR
                     YFEGVHVDGDPHEFGVLIGTDIIYAGTWSRGDTHGPGRSWRTIGHHCPVGVTARNASG
                     VGTNGLFAQAGCGRRVGLVRAEDGTVAFSGDMEDGRPIQGQAFDSQGLLVYSGNFGRH
                     GIDRGGTLYLPDGRIVRSDSWHQRWELTDGPNGSDDDNGNAIAPCDRATLVYPNGDTV
                     AYRWTVDTYEYTNVWRPVVRQFGYSEDGAPAAVAGSQLSSALGWQVVVPGRHNLDDLD
                     DQDSDADAKVALVHTHLWPLCSGHLNRRRWMHDFLFWPCVYPRGRDYPEVDDAIQFVQ
                     HMTSQHHSDWAPYRHAIYAFYGIPPP"
     gene            483668..484228
                     /gene="pino_cds_291"
     CDS             483668..484228
                     /gene="pino_cds_291"
                     /codon_start=1
                     /product="Hypothetical protein"
                     /translation="MGVELADDVTSALVRKGHFDSKAMRTQRFNEWARLPDPAASLRI
                     ERAPYNGSYDALFALYCKNRATRYNDGWGHEFDTAISQARSISADIVPSTAKHHIKAA
                     KNRLVHKNDHLWVYTTASKDKVILYQDEGVDTTFYYFDVFFADEHDAYITFLEQWEYR
                     LFVFVYPRTRKQSVYHTWFRKKVDRK"
     gene            complement(484283..484570)
                     /gene="pino_cds_292"
     CDS             complement(join(484283..484450,484523..484570))
                     /gene="pino_cds_292"
                     /codon_start=1
                     /product="Hypothetical protein"
                     /translation="MSNESGTSYMPDVEAKCSYATPEDAVPDPADVFSPERWEQIQSI
                     LQKFEKGNATLALAMKKQNPLGGLSDD"
     gene            complement(485139..485435)
                     /gene="pino_cds_293"
     CDS             complement(485139..485435)
                     /gene="pino_cds_293"
                     /codon_start=1
                     /product="F-box domain"
                     /translation="MDTDALCRRTRPRREPPTDVYVDTPACALTLADLPDEILFHIAS
                     CLPLGAVAALGCASHQMRAVCLDDSLWRRFYERDFPPCNEIATTKGACLTLGNG"
     gene            complement(485834..486490)
                     /gene="pino_cds_294"
     CDS             complement(485834..486490)
                     /gene="pino_cds_294"
                     /codon_start=1
                     /product="Ubiquitin-like"
                     /translation="MYGRPGADVGDVVLLTKDSQTKMAIITAFDTHTYTLAPHVGDSR
                     KCPRQSTDMQWSICIDTPDGNDGWVNAPLPPCCVCLDAAADRVLACRCTVPCVCAGCV
                     RKEQLDRCPQCRHPVASDPHGPHHIYANLYSIESYDDRPRITVFARTTAGKSMILRVH
                     RRRTVRTVKVALFVENGIPVDQQRLSFAGRAMNDAQTLADLGIKECATLHLVLRLMGD
                     "
     gene            complement(487016..488329)
                     /gene="pino_cds_295"
     CDS             complement(487016..488329)
                     /gene="pino_cds_295"
                     /codon_start=1
                     /product="Hypothetical protein"
                     /translation="MTDEMRALVLAATFAVGTLVACFAPIEAFLDDEIPCAIVAAAAA
                     FAIYLSWILSASLARRRKTKSASRRSHADTLRPAVDTPPCTTADIVVDNDNVKDCATP
                     QCPPVLPLQRVDFAVHPFGFDPYLDTVPRGALGMCEPISPQVDARWWRTFKASIIARY
                     GGPLSFALACEVHPDRLDQDRWARHVNINDDRDGDQLQVVIGKVVHGGTNWIVTRGQF
                     VKSDGQLVPHGYAVRQWHTGMTNEGLWHRGTWVQGYIYSPPVHGRDATTHRVHGGLPD
                     KIDFSVTWHTWDAHGRPGRHVRYCGPSTARRSGMPRNQWPHAQWPICYGGWTPTGACA
                     TCVCRFHNGDQYVQVSDGNGVPTILYYYIDSLPRGQLIGNCAWTIIAAQPDAAYGGAV
                     FYPADVGSPQFEAMAHYVLSGRSAEAFSPAQQAAFVAAIRTAQTV"
     gene            488798..490024
                     /gene="pino_cds_296"
     CDS             488798..490024
                     /gene="pino_cds_296"
                     /codon_start=1
                     /product="Ankyrin repeat"
                     /translation="MDLPDEILASIFQWLTCIQVATRAAQTCWRWRAVALDPTVRRLC
                     IGEPIQQDPCVESPGREACRRAAAAGHIACARNLLVSHGRYGSLVVSTAARHGHANLL
                     AWLINGAYDPERDADACSEAASGGHVDCLALLHKARYPLHTRVLWTAARYGHIDCVDY
                     AIAHGCQPDQFYVCQSRSTPAHLACVKKIVAAGGRTNPIGDMVAVRAGHVDCLRLFGG
                     SSRRDGWACQSAAYRGNLEMLHFLHKNGWDWDARTFKAAAESDNHDCLAYVVKKRCPW
                     TFDDLCRVARSGRWANVMAVIRPESGDTRPTTAAASMGRLDLLSDLCQQGYACESDAC
                     LAAAFNGHVDCLHYLYKQRCAWDARTLDAALAGANRRCFEYAVARGCPLTPRTRKAAL
                     ARGWKTEPRLPLSRPP"
     gene            complement(490559..490966)
                     /gene="pino_cds_297"
     CDS             complement(490559..490966)
                     /gene="pino_cds_297"
                     /codon_start=1
                     /product="Hypothetical protein"
                     /translation="MEPNPFSQAAATAADDNAAASKHSIMPCCDIPQNSLGRGAECCV
                     SAAHPAGASNLESAPADAWDLDQGVADKLVAGATNLRVYNFDDDVLGTTDIAAANNRL
                     AASVYRTCARLGYHITGASERMPQTFYIEKTES"
     gene            492411..493909
                     /gene="pino_cds_298"
     CDS             join(492411..493127,493211..493909)
                     /gene="pino_cds_298"
                     /codon_start=1
                     /product="F-box domain"
                     /translation="MATVTTMDDLPDEIIELILGELDHIADRIAAHCVCRLWRERLAQ
                     ARRYAWALCRTDLWAVAVCALENGKWSATSWLLDGRDQLSARGNRLLLAATRADNADA
                     VAWLRARGCKWTPDLAAKAIAEGHKKVIDQARDDGHLCHKDVLCALAQIDDTETLQSI
                     ACLSSSALASIIRAATASGSVAVLRWLYERDASWATPDEAKRIILKGHLRTVVWAVEE
                     AGMTIDSQWLYTALEKERLDTAIRQGRADIAESVIDHPTVQFHIANSRTPWNAAWWPG
                     CATESHPDQDQVIRVLAPLLSTGFVQVGSTAYCDAARRNHVTLLQWLDARQCPKPPGD
                     RLGARCLESGYVGVVAWALSAGVVLPADPIRRAIRPRTHDDWDVDRHALVVMLTQHGH
                     PWSAGACEDAACQGMASALALGVAHSKDGWYPEMCLRLALSTDTPQHRKTAMWIAHRV
                     DIDVVAFERNLVRSTGAGGRK"
     gene            494335..494733
                     /gene="pino_cds_299"
     CDS             494335..494733
                     /gene="pino_cds_299"
                     /codon_start=1
                     /product="Hypothetical protein"
                     /translation="MSDGSGRGLGDLPLELLALVASHMRPDDLLTFCIANSTLLAACE
                     THVVEWRTATPQLVGVLPPRMSTRRFLSPLEVAWYRSALDVLCRKGALYAIMLVVDRA
                     RAFRAFVDACMASISARAVLQASLTGSRST"
     gene            494781..495311
                     /gene="pino_cds_300"
     CDS             494781..495311
                     /gene="pino_cds_300"
                     /codon_start=1
                     /product="Hypothetical protein"
                     /translation="MEAWARAQPHLAGFGLGYRQRAGDAPIKIIGPLGPHAAPVSCPP
                     ALLPVLDDAGAIGWDGALSAARNQAFVAETHTRINEALTKAIDKAVGVRADKRPEWLD
                     LMDQATAANRETPDTRDGSRPLDMCTKIAAFCEDVDLQRAYPGVALYLVKAGSRWAVG
                     IALPLFGNHDKDTPKD"
     gene            complement(495562..495996)
                     /gene="pino_cds_301"
     CDS             complement(495562..495996)
                     /gene="pino_cds_301"
                     /codon_start=1
                     /product="Ubiquitin-conjugating enzyme e2 domain"
                     /translation="MQGSYEEMRERRLAKEEQKLREAGIPYTRQPDKHTCTVHFSGPE
                     NTPYAESRYTLEIVPVRGWPFKAPEAFFVGPAPQHPFYAFDADDEHRRTRTTNLANTD
                     FGIYHERWNPRFCFVDFVERVRHSMTPPGEDEMRPFMCAQTK"
     gene            496313..497476
                     /gene="pino_cds_302"
     CDS             join(496313..496864,496943..497476)
                     /gene="pino_cds_302"
                     /codon_start=1
                     /product="Hypothetical protein"
                     /translation="MDDTTLLTSLPGTSPLRSCPCCPWPTLSHCRLRARRCTYWRHVQ
                     ACGGGFSCATLPTSIGRGSPPNRGPITTIPTTRGTRSPSTLEGHRRPRPYAAAVPAPG
                     PPSGTLCACPCGRQGLALALPGPRTDDAARETRRFVFGTSRLLCQPNDDRQVQLGKWQ
                     AVRLRVESDHGYRRHQRRQVDRMDRLPRPYLFSFCRDGHRRWTTFDASGSGIFTELCS
                     LGMRRDGEINGAGDVTSLTTHCADGRTMASICNGKVHGVQQSFWHNGDTMSVRYDYGE
                     FVEVTEFVCSPTCPRPEFAGAKITGCTWRPTSSAVAVDGKRYNVFVPIDDSDGTRLFW
                     RYVKDGLVGWDPRIRRAVLDAGTAGLA"
     gene            complement(497692..499218)
                     /gene="pino_cds_303"
     CDS             complement(497692..499218)
                     /gene="pino_cds_303"
                     /codon_start=1
                     /product="Ankyrin repeat"
                     /translation="MTDADTVAESSIQDLPHELVQHILVEFTDREARIMASLVCREWR
                     RQLWAGRRYRWRHANVAEGLSGMHRILSMACEAIARGHSAVGLWLLDNLSTAPPPGGG
                     QPSRPCPLMAAVHARDPNARRALRTRGWRWTAASAYDAINQGDADIVDEVINDYGSNE
                     RMIWRALASKGDIDRLRRLFDCGADVPSDRYTIPKALCNGHTDLVEWLMEHGADASQV
                     RACITSKRFSHQMPLATIIWAIDRGLINVYAKTVGKFVRRGRLDIVQWAHTNKGTPLD
                     SALLRKAIRSGQTTVVTWLLDQGAGVGPHEERECCWELVKDGCVDDATLKTVVERLFA
                     VGIVGLCDADFGSAAACDRPLTLRWMVERVWGSADSASAQCRLLLWLKCLTWASHRVG
                     EWMLSVGWAPPADAIHRVMRLRLIDIPCARFARLLANHGCAWAANDCLALARCGHRDL
                     LRRAVVDHGAPWSPHDCLAQALATDSPGHRATAEWIAQCAGIHVGAFERDLLNAHATA
                     "
     gene            499516..500544
                     /gene="pino_cds_304"
     CDS             499516..500544
                     /gene="pino_cds_304"
                     /codon_start=1
                     /product="Hypothetical protein"
                     /translation="MHFLHRAQSTRRQAAAAMALCETIDRALKAIRDGCDIATWVNIA
                     LGDPKQRGAVANAVAAAFWQEIRLGHHAEMSLEEAEQCRYIGMRRYAEDVAAGVADLQ
                     YAEGRHPADISVQGVLGEYAFIVTLFRLISRLDDTTPRGFLTETVFDAILEAEGWTVD
                     IKTTIPFEQADRARPLMVHVSKSRNPPHAYALIEYANYDPAMPLRTAITTPPRMRFNG
                     FASSRTVFEGVGCHSLIYERAFGLAGGDHREISYMCVVQPERLTDRAGLWHEHAERGC
                     LRYNKRDEDQRTYDNRLLQEPQALALDMARLHFYIEKKEARSGHPLKRGARIALLLTN
                     QWLRSAEV"
     gene            complement(501276..501800)
                     /gene="pino_cds_305"
     CDS             complement(501276..501800)
                     /gene="pino_cds_305"
                     /codon_start=1
                     /product="Hypothetical protein"
                     /translation="MRATGEEPPGHLTTGEGAILDLLDAVRDSNAAGELTQDVALAEL
                     IMAVALQDPDTLDELRGLFDEETMMASVLAIESLLLGPALLPDEPLLHDDDTEEAALA
                     IFGVFVQDDWSVYEDVLSAAARSDAQTLAKHIIQERLNNGLSPGFANALADEAEMNGH
                     DELAQLFREASGYY"
     gene            complement(501842..502477)
                     /gene="pino_cds_306"
     CDS             complement(501842..502477)
                     /gene="pino_cds_306"
                     /codon_start=1
                     /product="Hypothetical protein"
                     /translation="MQDATFSHSLYGPVGMALLEADGPSLDEVANVLALPKRIEGTEE
                     PLVDDDSTTAEPTASAYVRTIDPPCRLLPPGSAYDAAYDFYALDTDTMEFAENVPEPY
                     RTVMQSGTTTTAYDARQLIKGVKINANTQDLPLGSRAYVLNTEKGVCALDRRQYVDLR
                     QRAREAWWVPMGKNRSMTKCCGKRQWTNWPSGCPSLRSPSVLLPTTARGAL"
     gene            complement(502781..503350)
                     /gene="pino_cds_307"
     CDS             complement(502781..503350)
                     /gene="pino_cds_307"
                     /codon_start=1
                     /product="Hypothetical protein"
                     /translation="MNREDTYDSVNGYNAPFYVDRAGIDRSVPRSEVWQFDDIAETAP
                     LLTGDQLEDLDQSTAHIVIPIDAVMALGDAGSTNLSDHGLELAPFGSVGGKIVYQPAV
                     AERLTDEAGLRRDDRLYAGVIIYDRWTNNNEVTPWAAIGSRDQPIRGFYNGMPTRYAL
                     LSDLLSSALPATDAISTRTAATRFGRSHL"
     gene            503976..505384
                     /gene="pino_cds_308"
     CDS             join(503976..504877,505030..505384)
                     /gene="pino_cds_308"
                     /codon_start=1
                     /product="Ankyrin repeat"
                     /translation="MSAEGLPNEILGLVFSWVPCLDVRRTLALVSRQWASVAGDTRAT
                     GRRSCVGPKSRRRTLCHQATAAGHVDCLAYARSRGRSWGVNVCAAAAAGGHLTCLEYA
                     HTNGCPWDEHTPKAAAAGGHLDCLEYAHHNGCPWDGRTCDEAAKGGHVACLAYATSMG
                     CVCGRDTWMAAAAGGSTECLDIIESHSHWTINEVNGGSVYAAAAGRGHIVAIDWIMSR
                     GAWDAFALLKGAARHGRIDCLVYLLDRGLGADSCYGDDDADVVAAAARGGHVDCLRVL
                     IDNGFSVGVSVSTAAARGGHIDCLRGHLDCLVYAIQHGCPYDDYAIEAAARSGKLQCV
                     RYLYSIGAPVRGRALRAAAKAGKPGTVRFLLDNGCPIDSTTCADAAHPDVFLLLHSAG
                     CKLSRNWSWGTPLFYSGWQRSVYNAV"
     gene            complement(505859..506591)
                     /gene="pino_cds_309"
     CDS             complement(join(505859..506268,506489..506591))
                     /gene="pino_cds_309"
                     /codon_start=1
                     /product="Hypothetical protein"
                     /translation="MGHQTKTRRRGRSSIARSPTTAWWRRFWVRARPTDDKAAQTTTL
                     CRRLADCMAKDRHTLAGAFGALGCAKVLLVEPGDIDALHPWHFYVGPRRPALIHLAGS
                     VHSTRALLEEDGASHLVVSGDTNRDERFRVILSMGADDTMTAKIYRAPHNRPFGAPIS
                     CAVHAHPVPC"
     gene            complement(506989..507606)
                     /gene="pino_cds_310"
     CDS             complement(506989..507606)
                     /gene="pino_cds_310"
                     /codon_start=1
                     /product="Hypothetical protein"
                     /translation="MATAKRSNAGDLRRFLVAKRAKTGDAHEDHNKETVSSTQGRGPV
                     PATAAPLCASSDVPHSNGPTNGQRDQQPMATTRLTRVVRLRRRGGHIVQDCDVYIGRR
                     WTVGGWDLPQSEWANPYTVRQVGSAAEAVRLYEHEHLARRPDLVAKVGSLKGLVLGCW
                     CKNRPDDPCHGDVLARLADAASHIPANEEETTVPETCTPRHGDGV"
     gene            complement(508001..509134)
                     /gene="pino_cds_311"
     CDS             complement(join(508001..508923,509110..509134))
                     /gene="pino_cds_311"
                     /codon_start=1
                     /product="Ankyrin repeat"
                     /translation="MSRTTPRAGDIKTVGRRDLAACVAAHRCFYDAARPRLSKTRKIA
                     AWIRRGPLWACTTNNVDALTALAQRGVVFRQAHLTEAARCGHLDAVTFLCQKGIVTDD
                     PTDDANKTENNVAEDDAKPIQCTMPNPTALDVAAANGHGAIVRFLHANLDGTRVATTA
                     AMDQAAEHGHLDIVAFLHENRTEGCTECAMDWAAANGHTAIVAYLHEHRTEGCTPWAM
                     NAAATNGHLRTVAYLHEHRNEGCTTDAMDGAAANGHEDVIIYLDRHRDEGCTGNARID
                     ACLGGHDHIVVLMKERGIVPGRRHGRRRRRYKPAAGPGT"
     gene            complement(509382..511094)
                     /gene="pino_cds_312"
     CDS             complement(509382..511094)
                     /gene="pino_cds_312"
                     /codon_start=1
                     /product="Ankyrin repeat"
                     /translation="MRHTRTHARRKRMLTAMPSEIAACVLAYLNDIDFCSARLAHRWF
                     LVHTDDEITQQRRLAVWGRHDLDLCRKGDTAAVAALAAAGHYFTSRHLGEAAAHGRIE
                     LIDLLLSDAVPHARYSPRAMNRAAEAGRLDVVIHLRSIGTGQHPLDDADDNEHPVAMD
                     YAAAGGHLDIVEWLHNNTRQGCTTAAMDWAAVGGHVEVLQWLHSHRSEGCTVRAGSTS
                     CGGNVQAVEWIFGHLPQQHIDPVRIFRDAASRGHMDVLCWLHANHHIPNYLPSMGDSA
                     AAHGRLDVLQWMVTHVAGVQFDASTTHAAAQNGHLGVVEWLCDNYPDAQPTPRVLTTA
                     LHGGHMNVVNYLCERHPDLAVLDSAIDTVVSCRCFAILDRDAPQDCFDALEWLRVNRP
                     DIVPSQSAMGAAIFTGHLDVAQWLHAHYGTGCTTESIDTAAATGRIDLIDWVHDIYGH
                     ACTVDALHSAAEQGHVAILEWLHDHFPHLTPTTSTLDAAARGAHLGVLQWLHQNHPDV
                     RASDSTLALAIHGGNLAVVRFLCETYALEVTETLIAEADRREHFAIVDYLRSVRAAHD
                     SLSA"
     gene            complement(511635..512804)
                     /gene="pino_cds_313"
     CDS             complement(511635..512804)
                     /gene="pino_cds_313"
                     /codon_start=1
                     /product="Dna methylase n-4/n-6"
                     /translation="MQDDSEEVLSCNPDDRQCSTPNVPADVVDDIGQDSKPSLPERRR
                     CVLVNGDARHLDFVDDASVHLVLTSPPYWNLKEYNVAAEVEGQLGHVADYADFLKQLD
                     KVWTECYRVLVPGSRLVVVVGDVLLSRKRHGRHRLVPLHSDIQIACQKVGFDCLAPII
                     WHKIGSAAHEVNNGRASMLGKPYEPNAIIKNDIEYILMLRKPGGYRSPTPAQRDLSRI
                     PKADFHAWFRQIWTDVPGTRSKDHPAPFPRELAARLVRMFSFDGDTVVDPFAGSGTTL
                     IAALDARRHAIGAEIDPTYFALARKKVSSALPSIRWRERHCDASNDNHGQDRGDDLVA
                     DNEPIAKDCIADFIPDKSDNSTARLCFVGTLAAVGGVPRGANGKSHKRKRSPTDI"
     gene            513451..517196
                     /gene="pino_cds_314"
     CDS             join(513451..514940,515685..516575,516674..517196)
                     /gene="pino_cds_314"
                     /codon_start=1
                     /product="Ankyrin repeat"
                     /translation="MAMTMPTPGDSGIDTLPPEVIAAITAHLGDRDLCRARAAHRCFR
                     ADSDETMAKRADRWRGDKTPEHFSAVGLVEALEILHSRGMAMGPACAKAAASAGHVDV
                     LAFLRRVGTPFDVLETLDIASLFTALAGGLRTKRSTMAAGEIISTVVMTLAFGAPSGA
                     ENASRRVMSLADVAARNGHLHAVVWLVRVAGVTPTTMAMDCAAARGHLDVVRWLHDNC
                     DHGCTTTAMDLAAIGGHLDVVAFLHQCRTEGCTTAAMDGAAAGGHLDVVAFLHKHCTK
                     GCTTTAIDSAAVAGHMPVVRFLCENRTEGFTRPTIHEAEAAGYPYVAAYLARRTGPSG
                     AEEHTISGPFASYVDGDVVDDNGFGVAGGTTGMQPTDDDGTRTNLRAEFVNAVLKGDI
                     AAMEQIHARGLALDGPTPRAHNNYWMMAAMIGRLDIIQWLDAHDASGRDCGAVTVALV
                     TERLDIARWLYEHGVRDDLTEMIDDAAASGEADPVRAVYDQYASILGSNTDGRTAPTR
                     RAGRARRCRGDRRGAESSRRCHGPRGQRGARADRPRPRRVGGAPGLPHMDRPRARAPM
                     RPCSRPAWGRLGPAADHGPRFAQPRRPVASRAVAATETEFQPHQPNASRRAHRASVPQ
                     GCRRFATILLCLARLDASTCHDLGMIYLGARESDGRRAVCYAFDVHATAPATEWQGVA
                     QIFGAGGLAWFLGDPAVPKAVCGAGPGHDSVAHAATRDIAQLARAPTADGAAMTNLLV
                     RSVVRALPPAGLTPGGGKDSAGAAQDAAIKALHAHKFDMALWAQAGEASSGYRCAIHE
                     PGPPSATTPVTAVTDSLLCVAAAQTAACNRWPLSVAPRGDDRQPNRDSDITLACPLDD
                     LYGKVDDRRAAVHAKQKPIKTDADARCDVEGDNDWDDLWRAVQHYMAVSATSDSYAAA
                     AADDDRDRRQGVQQGDHWHRDVGEDDDRDFWPVLLPSPLWAYDEPWCPNGQNI"
     gene            complement(517700..518629)
                     /gene="pino_cds_315"
     CDS             complement(517700..518629)
                     /gene="pino_cds_315"
                     /codon_start=1
                     /product="Methyltransferase type 11"
                     /translation="MSTLDSKTTEDGTTTASADAAATGTPSKNLATDVGALYSSDMSA
                     LPAFVGPFINFGYWDANRASARGEPPADAPVPTLAERQASSRALYERVFEALDPRGAV
                     AEVGCGLGAGCRALAAQHEPHVLTSVTGIDLSTDQLARAKALCSATDARLGFAQGPAE
                     RLPLGDASVDRIYTVEALQHFDSPSAFVTEAARCLAPGGRLVVCTFLADRALTAQEHD
                     VIASGVRTVAVGIDKLVVVGDLQAQMRAAGLSVQSPARVGDRVWDAFGAWCALAQPTW
                     TWPAAWLRAYHAGWVDYFIVVADKPCAGPTTPP"
     gene            518709..520991
                     /gene="pino_cds_316"
     CDS             join(518709..518740,519122..520991)
                     /gene="pino_cds_316"
                     /codon_start=1
                     /product="F-box domain"
                     /translation="MGTRGRHQQRRERGSTGPRQSKRARTDGTVVQQDRRTQCQWAAV
                     SAVCRQRRHRQIQEPSHVVWDVLSDEAVWCILVMCAQRDICALAGTCRRMHRLCMDDA
                     LWRHIYRRDFPPCRGACLLTLGDEVSRSSFSPLDYARRRLDRLLDPGDDLSMPDPTPD
                     SAALWTVERLTSAAIHGVSQCVHHWSDVIAARGYRWACASMIPLAHRTMPQRPFYPVD
                     EPGAKGTVVGYCTVRSASIYKLRDDVEETCDLPYKASYRGEVEIRPCGFVAHGLGTIN
                     DHDAAAFAWACRSGAWNHGRDHTSGTCALWQRAIGRYDAVVFVHCSGRDLVHHAEMPG
                     QPDCGIGVLLSSDGDVAVGCLYHPDGDPREGTTRLAAIARIGQTERHVGSVTAMATCA
                     SLRRVTDRLCGILPGGIDGRGLLRTPSQRVAFAGSFVKGEPHIGKTWDADGRLLYAGF
                     FEHNALDRGTLYTADGTTLQGSWVRGWRDIDSPACRIPATYLTASHPDGATVLWKDWM
                     RVDRRTRPRVADFWWTLSTNGIVTTDRPWPSADWDVLVLPPDLAQPTRAPARPLLADV
                     GLAVARSMHLLHADDIVDDLAFWPRPNPPDGPRPHLDDVLAFLSRMVATRPRWARCLG
                     VVSAMYGPL"
     gene            521721..523664
                     /gene="pino_cds_317"
     CDS             521721..523664
                     /gene="pino_cds_317"
                     /codon_start=1
                     /product="F-box domain"
                     /translation="MDSAGIGADATECLPDELWHMILNGADTRGLSFLPQSSRVAARM
                     TCRRWRLVVSTPSATDRIRLVGDDDDDPDGRLAVGAVITVDAIRPLFAQSSTFAEGLA
                     RVRAAYLPADHGDVNLDTVCVDDRRDEIHSVLLAAAAATGMAVHLDAVAALLGKPPRD
                     AFSAWLHDTDTHRMADVADAVYDACVRADVDRGVDLAAPFLGLDRLVAPAKYAICTDD
                     LTMLPVAVRHARRAILLACARKKAEKAARFCDSRLWTFVLKRGTPTTARLLVHAGVSI
                     DSLSTNDDYHEPDDILFFESNETFFSYYATARWHESADHDHAPDRALNDALWLMCWAI
                     KHGDSLLCAWTARCYATRYGRGFGPLEADEAITLAMRSGTYRMLSRWFFAYASQDITP
                     QTIATVLSEAAAMPYDETLRQNVEQFFRNWAREIAQADDGRIATDYLFHRPSWTLAWI
                     VREHAPPVSGPLALVDGLWARHAGRVHWALAQGSLSAVSSALAKLCRAARRWGLVSDT
                     IIAAEAAALGRHRPDRHPWARTLVHLQAVTPDARAWHPWTGTVVPLSSAACDAIAALS
                     ARPTCGARPTTDGMEIDTDNQRGADGKDCGDDDDGADGVHASAHKQRARDPKHRSSAC
                     CALGAVRLLARADLALPLPRDVA"
     gene            complement(523823..524850)
                     /gene="pino_cds_318"
     CDS             complement(join(523823..524020,524401..524850))
                     /gene="pino_cds_318"
                     /codon_start=1
                     /product="Hypothetical protein"
                     /translation="MACMDACSLWAGGPRLGDMPTEMIDVIASHLARARDAVSYYVAL
                     GQSPTRALTERVLADPVAFLAAGAPLDITQTLLDRMRTGVPVPFDWVEAAVYGGQQGV
                     ITTVWTRASVDTAADLYRFEWRRVGRGRGGASLVRRDAFWRPPSTVAETILDLGDRTH
                     LIIHGGLNRVLLATAVSNLITLATEQSVAWMTTSWWPFLLGIIVGYVATVMVVTE"
     gene            complement(525502..525657)
                     /gene="pino_cds_319"
     CDS             complement(525502..525657)
                     /gene="pino_cds_319"
                     /codon_start=1
                     /product="Hypothetical protein"
                     /translation="MKWRVPVIKTKMLVRGKEDVMASSSWRALGQWRVGFLLPMRLMG
                     TSKPGRT"
     gene            526339..526638
                     /gene="pino_cds_320"
     CDS             526339..526638
                     /gene="pino_cds_320"
                     /codon_start=1
                     /product="Hypothetical protein"
                     /translation="MDLVTQIHSAAEFAQVRQQLGLTSDSTAQFVYCLFEEGACPRVP
                     HESVPQPIEVDLPQTKGHTPHTLRFVAATALHEQNTRDEPPVAGSPAHDQRQAAS"
     gene            complement(527088..527884)
                     /gene="pino_cds_321"
     CDS             complement(join(527088..527419,527500..527884))
                     /gene="pino_cds_321"
                     /codon_start=1
                     /product="Hypothetical protein"
                     /translation="MSTCVAARPYQRSLRPKAQKTSATAIACCVGDACALDVLHALVG
                     MKETAMDVEDNGLGTWIGAARRVDEHIAKIVAEGDALLCKAIALGPQQKADAEAVVTA
                     RDIWYERLGDAWYDARYSASEIDRTLGWREARHLAAMAPAVDRTVKRLEAAAMSLGGI
                     VHASAAIRDTYAFMRSNNFGHIMAALAGSSQHSALALLFAAVTGDVDPRLNIIDILDQ
                     AVERVCLPAACAVVDDSPTD"
     gene            528255..529319
                     /gene="pino_cds_322"
     CDS             528255..529319
                     /gene="pino_cds_322"
                     /codon_start=1
                     /product="Hypothetical protein"
                     /translation="MDEICTGAINAALPPEILYEVFLFVGHGHTPCMARMVCRWWRDV
                     IVGSGDPDVRLLRAPQVLDLLDEASTSESLVARAAWIMTLVAPPNGDCVAVFQHMCAV
                     LGSAGRWQQVLVLVKQTPRALDDGAFVSVASGAAACTRADDILGCASAANARRMWLCA
                     GLCGALTSGEAMATAALCAHLGSCEGALGDDLCVQRVDAATTTAAQRGHVDAVLAQQF
                     CPGLNVALLWDSVVESCDAAGFAKLVEAVRAAGPDAMASARRASWTSERSRSVADANR
                     RSLALGWYGGGWTRPAAIAGWATPFDLATDVTDDMVLGHDHPYRVSSSCLWADAAALA
                     AQHGHDRLAAQLMAAAARSP"
     gene            complement(529697..530869)
                     /gene="pino_cds_323"
     CDS             complement(529697..530869)
                     /gene="pino_cds_323"
                     /codon_start=1
                     /product="Icp0-binding domain"
                     /translation="MASAAQDAALDGGPARERTADTNVPALPGVVALDARGTRMSTAR
                     TTLANAPLGSLLHRIAAPSPECPFSAPECRPDGSYFVDVNPRWLSVVIDYLAHGVVTA
                     PKLTPGVLGGVQAAADYLGLGDLALECAARLARIETADAPPDHRIKVHLVTATDLHTH
                     TGALDVFSRHRHRGADSPFTIAFLRHHPMNVVGHIVCEALGRAPADVVFYACWMRRNT
                     TLRPLAPLDMTASTPLSNCWAECDREMWLYVMKRKPPVDLVPVPPCGPSIPKAPPRAD
                     VPMLVFVKVFSPANRYLSGPRHAFVTPCATVASALPALLEIFSNHTHTVRDGDAVVYE
                     EATNTTAYQVNMEGTFAEADIETGDILWLCSRTAGLGFRVTMADYDWIVAPGGPQW"
     gene            complement(531570..532409)
                     /gene="pino_cds_324"
     CDS             complement(531570..532409)
                     /gene="pino_cds_324"
                     /codon_start=1
                     /product="Hypothetical protein"
                     /translation="MADGGIDGGGGGRHLIEWIVELTELCTRVAGGAGTPDDIRTFTR
                     LLDGASFGALLGEDTVVGNGTGLVTRYRRFWAALDHDPDALAAVAREARGWPPSIVDA
                     AMAYVYISEWKAMAMMRALAEDEAQGTQRLSDASVPERLVTLLGNAIRHAAERAPLSG
                     RDAAVPRGGSYAGDRAPMGGRGSLGALAMYDAGAAIRRGVPPSPVVDPYPRVGMSPQS
                     SVSYDAYEHTDEPVVVVYRDRNYDHRRQHDDQAGNRGSPDCSQEGAWWARICHIPGQD
                     PHP"
     gene            complement(532947..534092)
                     /gene="pino_cds_325"
     CDS             complement(532947..534092)
                     /gene="pino_cds_325"
                     /codon_start=1
                     /product="Hypothetical protein"
                     /translation="MDKAQCTKETVATVERRPNYVRLNVGGTIRYVERALVGIAPLGS
                     RAHAYLTAAPDATDLHFVDERPQHFARLLDCLRHGDTALAFMRPYDAAIVRRLLTPIE
                     PKGVDDHGATHKGEDSAMSLAQEPTTTDRPLGTAPVGHDGGAEQPCAIDPVIRLLVDG
                     DVPMDVAQSTLTSPPDSLLARMARGDVGWSPAADGDRLCIDQNHRHFGLLLDCLRHGA
                     QVLQFVDDLYDLWGVRALGTYYAIDIARDAAQLAIGWRGYLEADKNDRCTTYIVEPGT
                     PASISGLYSGSPLAHFMHSSHVSLTEFVALAAAAVGAPSDAVVVYASPYGDKWYPVAG
                     DDDDTDYWDYTRCHHASGRISVAMVEARPLVALDDDAATTPPSTPYL"
     gene            534587..535204
                     /gene="pino_cds_326"
     CDS             534587..535204
                     /gene="pino_cds_326"
                     /codon_start=1
                     /product="Hypothetical protein"
                     /translation="MARGGLMTMTTAATALTATVIAILALLIVVMVPAPAGAAPGDPI
                     YPGTLVRIYAVGPSQWVRRSPTHSTSPVRAADNISQSQATLLVLEGGPTNATIPLPST
                     IINLFDAFTTRPYCEPRDGVSGWSDTTIMCWETSDGAWLRVVAVGSSTPNTALNSGDE
                     VYLYNVRNDRWCVAPTWPTNNAGLYCSFFTTGSMPSQARFRIESA"
     gene            complement(536014..536772)
                     /gene="pino_cds_327"
     CDS             complement(536014..536772)
                     /gene="pino_cds_327"
                     /codon_start=1
                     /product="Hypothetical protein"
                     /translation="MNNDASDPVTATAPVATATDHGPVDDHRQGADVACHHATVTTVP
                     LSPAMPSADDDADGFLSDALFACAPPASLQKPSASTMCHCNGQADNSDIDTGRRRRRR
                     YRDDDNSNLNGTWCQLASMAMLALAVSVLVVTVMRPSAASGVDGTLSNVTGPTIVDNA
                     TTTILAPCHCAGEPRPGPTVEGRLTISGSVATCTCTWPVERVTTTVYMTVPRWVHDFF
                     SNLDALAEPVRTALVIVALTVAAPLFFLHLFFGA"
     gene            complement(537202..540482)
                     /gene="pino_cds_328"
     CDS             complement(join(537202..538471,539212..539616,
                     539694..540038,540139..540482))
                     /gene="pino_cds_328"
                     /codon_start=1
                     /product="Fascin-like domain"
                     /translation="MQSMNRLFVLATLAALLCCAATTVDGHRYSVLLGWSQTWSLPSN
                     ATNVVVSLWGGGGGAASTVSCGAGGGSGAAILARAAGDAQWGLDSALVQWAPWTSPGG
                     RSAGDAQSRRRGGTSRRGCKGGAGGGAASAAVGTDPGTGNPTGAVDNNHLAAATQGAL
                     VGDIKAGGAGAGFGYKYNVDSQPFTAGAPWTSPDRQWLGGPGMHVELDARVGAAPLDS
                     TATAATTSMASPPGAAGGVLIEYTHPVSPIIALRSPTTNKYLTAHSYGGVSASAATVQ
                     AWERWSAVRMDNGKYAFRSWQDKFLKANPTDSVEASSTTASTWEQFEIVLKGYNRWSL
                     KNHYGKYVVAAADGFVLSTTDADHFWEIVYLICHEPSGCLLGPCGTSRCAAGPAHGHR
                     YSVLFRASQTWSLPSNATNVVVSVWGGGGGGSSTGFCTAGGGSGSAVMFRAAGEATWP
                     VPVNQVQWSIVTGGGGWGLNSPTYGGTAGNGGNTVVVATAPNGTELFRAAAYGGGGGY
                     SIISTTRRGCQGGAGGGATSAAIGTYPGGGSPGGAVDNNPLAAPQEGATIGDVKAGGA
                     GAGYGYAYNTLSMPFTVGAGWTSPVRQWAGGQGQVVIPCYGSGGAAGFNGNGGDSQTA
                     VPKSPTPNSGSGGGSAYLCYPTRLYGDSNGAAGGALIEYTHPVSPVVGLRSSVSGKYL
                     TAHSYSGVSAAATSIQAWEQWTAIRLDSGKYNFRSWQDKFLKANPTDSVEASSTTAST
                     WEQFEVIEQAPDRWSLKNHYGKYVVAAADGFVLSTTDADHYWTVVTL"
     gene            541390..543411
                     /gene="pino_cds_329"
     CDS             541390..543411
                     /gene="pino_cds_329"
                     /codon_start=1
                     /product="Hypothetical protein"
                     /translation="MTTDTTTTTRRRHGTAVGRAAIDLVAAAYLEHKIEWIASRLDCR
                     VDGGVATITLNGHTVHTGDADTLDRVRAELPTRLAATVDIDDPMFVQCVYLSEHAKRI
                     TDFWEQPGRRAVDWLGRSLCADDDVAHCALDIAALVGGPRWQAYVDTSANVRVGTTLP
                     WTPCLVAGARNTRRDLFLQDWDMLVIRDILDRVRQSHGPDGTVYAQRRRQPVVRHGGL
                     CAVEVAPGQWDLSRGPSGKSTGMPPVDVDCMAQMLASARLVTYHHTGVSLHIDGHMWA
                     CVEEAGKHVNIALAQTSLDRNVARTRAILSAAALTDDPRKGDFVDMASAVHDAVAIDQ
                     GSRHTVAPDSAWLAGLREALHHQESLRMRLWIGGVWDAAIPSVVIECVNESFVADAQG
                     VWRDAFGVVASWDDLMRAAQVKRRLGSSSITLMPHYGRDVNVRLTRDDPCAAPDFAHR
                     LCCDALAGDLYAIDWLGVLALASPQWAMLDATVCGVLTWVRDGALTTAPPSGNSAPWC
                     ESLIDWILDMAAGDDASARFAADEDAFNRSALCAIDEAGRRVSDRCMCTGPGSDNIPA
                     CCDGMCIWRTHDHIATLPKVSGGPGMKVLYKATYRGAVTRSLTRTALGIDGRQILAAD
                     AFCPLLGERGRGVEALVRALHTCEPLRKGIIEPAQLALADRVLTARSAQ"
     gene            544179..544667
                     /gene="pino_cds_330"
     CDS             544179..544667
                     /gene="pino_cds_330"
                     /codon_start=1
                     /product="Hypothetical protein"
                     /translation="MWSGDDDGRRRVARRDSGARLLVGPCVRLVSTVPLVSRRWQRVG
                     QDAKAIGRPLCVVRQAADSKEKSGYCALAAKSGHLDCLIYARARGRPWGRGVYLAAAA
                     GGHLDILRTRTYTARPSMMAPLRRPWRRVTLPVYGGSAITATPGARPRVPALDLPLTL
                     YD"
     gene            544736..545356
                     /gene="pino_cds_331"
     CDS             544736..545356
                     /gene="pino_cds_331"
                     /codon_start=1
                     /product="Ankyrin repeat"
                     /translation="MDCLHYAFENGCPHNRDAALVAARIGWLDGIQYMESHGITSRQD
                     AMCREAAIGGHAHALQYMIDKGFTWKPSLDDLRAALGFESLDVLCLLLQRADTDLPVA
                     RKAAHLGRPDALRLVHQAGWRLDKLVCDAAAAAGDLACLTYAFEHGCRWTHLTTHYAL
                     ETDSVECLAYAHEHGAPWHYDMINECIRNKARRCLAYAVEHGCPMP"
     gene            545733..546638
                     /gene="pino_cds_332"
     CDS             545733..546638
                     /gene="pino_cds_332"
                     /codon_start=1
                     /product="Hypothetical protein"
                     /translation="MPKETRRQCTKRHATLDARRAEALLPHHAEGTQQALWPTDVDVA
                     ASLPELPATFSFGSAALPDTAPSPTAFDPFMPSPPPLLPSPPTPLPLRAMVSAPTTTA
                     PSSPLAWPTRVSTATTIVVVLDARGTRMSTSQTTLANAPAGTPLHRISITAMTAIDQD
                     KDVDCTESTNADEPRVFVPCIAQADGSYFVDVNGVWLSIILDYLAHGVVTAPEFGPTV
                     VMGVQAAADYLGLYGLSSECAVRLARAQAAEESIDEVLRKLTARVARVEANATAQETQ
                     HSTFWQAALRPRSNLVPILGERLFS"
     gene            547381..547964
                     /gene="pino_cds_333"
     CDS             join(547381..547523,547679..547964)
                     /gene="pino_cds_333"
                     /codon_start=1
                     /product="Hypothetical protein"
                     /translation="MRRKDTITTSTALVALTLYVLATVTIAMADDAIRPGSLVKIYAV
                     GPSHTGPYCDPRLGVAGWFDSTIMCWDTTGGPWLSLVSASTGPNTVLNNGDEVYLFNG
                     LNHQWCGAPTWPTNNAGLYCGISDVGGTDNSILRFRIYLA"
     gene            complement(548757..550156)
                     /gene="pino_cds_334"
     CDS             complement(join(548757..549138,549240..550156))
                     /gene="pino_cds_334"
                     /codon_start=1
                     /product="Fascin-like domain"
                     /translation="MCGRRRAYVALFLLACCLAAPGLHAYRYSVTITGSQAWTAPVGA
                     SNVSVTLWGGGGGSSTSIVCGASGGSGAAIIGRSAGSNAWDVPVDSVQWSVTVGAGGA
                     GVPTSFDTEFYGATAGNGGETLVVATAPNGTELFRAVAYGGGGAKSTGFGQPRACQGG
                     GGGGQASSASGPVPGGGTPMGGVDNNPVGASTEGATMGDVKAGGAGAGYGFVGGDFLN
                     PFGRGADWNSPGRTWAGGHGSATTACFAWGGAAGFNGDGGWRQDFIPQYPPANSGSGG
                     GSALICNSGNSYATKYYDDVAGASGGVIIDPTAQPLSQLVTLVSPISGRQLTPQDDGS
                     VKSLWYGASYKEKWTVARLSNGKYTFRGFNGKYLGANPGGWVRAEATSVGAWEQWDVL
                     INAGDQWTLKSTHGTYMGTTTAGVIYLNNDSTLYWTKTTV"
     gene            551373..552626
                     /gene="pino_cds_335"
     CDS             551373..552626
                     /gene="pino_cds_335"
                     /codon_start=1
                     /product="Hypothetical protein"
                     /translation="MHGDAPYVALSLSVVALAIHVVHASLSHRRRRRQSVYRDAKPCD
                     GALCQCDAVDRDSSDVAAAQAIGVVSSGDGMPLQHVPVPAPPIPARRLHPAVHPFGGD
                     PVVDAVPRGVLVICDPIAAQIDASGWQRFKWSVISRHGGPLSFALACEVHHDTLDQER
                     WIKGGDVNKDGGNRDGPVVVGKVTDSQGAGITVSGQLVWHAGALRPHGYAIRRHPCGM
                     VYEGRWNRGSWIDGYIYRPPTSKCDSVTRRVCPFSPDKIDFTVTWRARDAEGRSRCHV
                     RHPCPPSARRVAMSLAEWPYPNYSVYCGRRAPAGACATCICRFRNGDSYAEARDGDGP
                     PTILYYYIDAAPRGQMIGNCEWTIVPAERGAVYTGSVFYPTDVKSSQFQAMADYVLSG
                     HSSHAFSHAQHEAFARAIHAGTQAP"
     gene            complement(553391..553783)
                     /gene="pino_cds_336"
     CDS             complement(553391..553783)
                     /gene="pino_cds_336"
                     /codon_start=1
                     /product="Fascin-like domain"
                     /translation="MTPTPSAQPWLQLVTLVSPISGKQLTPQEDGSVKSLWYGASYKE
                     KWTVSRLASGKYTFKGFNNHYLGADPGGWVRADATTVGSWEQWDVLINNGDQWTLESV
                     HGTYMGTTVDGVIYLNNNAALYWTKTTV"
     gene            complement(553792..554796)
                     /gene="pino_cds_337"
     CDS             complement(553792..554796)
                     /gene="pino_cds_337"
                     /codon_start=1
                     /product="Hypothetical protein"
                     /translation="MPKGRACALVLFVAWSLLAATTDAYHYAIFIGQSGTWSAPVGAT
                     NVTVTLWGGGGGAATSLYCGASGGSGAAIIGRITGSDGWSVAASDVQWNVTIGQGGAG
                     LPRVTPTGWYGGTAGDGGSTLVVATAPGGVELFRAVAYGGGGAKATGMGSVRACQGGG
                     GGGQNSSAAGPAPGGGIPAGGADDSPQGPPTEGAMADDVKAGGAGAGYGFVGGNFTNP
                     FVRGANWNSPGRAWVSGSGLSGDYPNGWCYAWGGAAGFNGDGAPGQVSTPQYAGANSG
                     SGGGSALLCIGGELAGKHSADTAGASGGAIVEYDHPAAPSPSPRAHPRPRRRGRPRRR
                     "
     gene            complement(555390..556815)
                     /gene="pino_cds_338"
     CDS             complement(join(555390..556563,556622..556815))
                     /gene="pino_cds_338"
                     /codon_start=1
                     /product="Fascin-like domain"
                     /translation="MQRPSTIKSGGRPSIGAVLATAVALTALCLLAVPPAASLYHYTV
                     FVPTSRTWAAPASATNVTITLPFRAAGSEAWGLPIQEVQWAITVGQGGAPRDAGEYDP
                     IYTGGTAGDGGATIVVATGPDSTELFRATAYGGGGAKVTDPDEPRACQGGGGGGASSS
                     AVGPVPGGGTPMGGVDNNPVGGPTEGAMVGDVKAGGAGAGYGSVGGDFVQPFNQGAGW
                     TSPGRSWGGGSGHLTYACYGWGGAAGFNGNGGNAVFASATRPALNSGSGAGSAITCTN
                     GFVYEEGDATGAAGGVLIEYDHPVAPSPSTTPTPSVTPTRSPTPSRTPSMTPTPSMQP
                     FSQLVTLVSPISGKQLTPQEDGSVASLWYGASYKEKWTVARLASGKYTFKGFNGRYLG
                     ANPGGWVRADATSVGSWEQWEVIINPGSQWTLKSVHGTYMGTTAAGVIYLNNDSTLYW
                     TKTTV"
     gene            complement(557599..559007)
                     /gene="pino_cds_339"
     CDS             complement(join(557599..558169,558247..559007))
                     /gene="pino_cds_339"
                     /codon_start=1
                     /product="Fascin-like domain"
                     /translation="MKACATNPMVTTAALVTVVAIISLLALGAPTTEAYRYSVSFGAS
                     QAWSLPAGAANVSVTLWGGGGGSSTSLYCGAGGGSGATIVGRAVDASDWSISASDVVW
                     SVVIGKGGAPSPVGAQESVAGDGGATSIIAMMPNGTVLFNATAYAGGGGKSSSRVDRT
                     GCQGGAGGGASSSAVGPQPGGGNPSGGADNDRQAAPKEGALVGDIKAGSAGAGFGYAF
                     DSPITRGAPWRGFGRTWSGGAGRGSTLCWSSGGAAAGAGAGSAEACKTYVLSQDVAGA
                     DGGAVIEYDHPVAPSPSVSPAASVTPSRTPSPSRTPLPSPTPTAQPLTQLVTLVSPIS
                     GRQLTPQEDGSVKSLWVGASYKEKWTVARLPSGKYTFRGFNGKYLGANPGGWVRAEVT
                     SVGAWEQWDVIINNDNRWTLKSTHGTYMGTTAAGVVYLNDNADLYWTKTNV"
     gene            complement(559512..562103)
                     /gene="pino_cds_340"
     CDS             complement(join(559512..560893,561638..562103))
                     /gene="pino_cds_340"
                     /codon_start=1
                     /product="Fascin-like domain"
                     /translation="MDSAPPATSQPPLPSQEAHVGVAQLMRLLEIAETFRTMPPMPPR
                     QQLSTGSFNPLLGIPPGRVRAGSMAVRPLPVTRPQRKSSTPSCVANDAPAERVDTVSS
                     DCPAAPLLDDRTDIAQADTDATGGTDRQHHQVPCTVNTQEAPPQTAPLDSVDCDNDNI
                     GCDGALGAVRSGACSGRLPLHCLCARIASVGAAGQRYQHCSDAVGWRWRLSHHALLWC
                     GRWRRCHHSRPRVDSSGWPVSVDGASWVLTVGRGGLPPAPVFTQAVSGNGGATSVAVV
                     APGGAVLFNATAYGGGGGSASSAVERDGCQGGAGGGEASSAVGPIPGSGNPSGGADDD
                     RLAPPKEGVQVGDVKAGSAGAGYGHVAGNTDQPFRTGAGWHAAGRAWAGGEGRWSAGC
                     RSWGGGAAYNGAGGPGLNNALGPGVFPRLPPASSGSGAGSAEACVIYKTGYDAPGADG
                     GIIIEYDHPLGPSPSASPTPSRTASPSPTRLPTPSVSPTSSAQPLPQLITLVSPISGK
                     HLTPQDDGSVKSLWVGASYKEKWTVTRLANNKYTFRGFNGKYLGANPGGWVRAEVTSV
                     GAWEQWDVLINAGNQWTLKSTHGTYMGSTAAGVIYLNDNASLYWTKTAV"
     gene            562171..564032
                     /gene="pino_cds_341"
     CDS             join(562171..563118,563268..564032)
                     /gene="pino_cds_341"
                     /codon_start=1
                     /product="Hypothetical protein"
                     /translation="MFYSPSLVCGRQKRQTQCHWSYTRPHRILPLVVQSTLQARSGVG
                     CAVCTQKEPDRMDAACAEAADNCSNQDGMDPTDILPVELWTNILNGCDGRRGLLFLDP
                     RFRFAGRATCALWSAIVQRPGPDFAAALNAPPWRCQTACTWPKHPWYLALADGRAVCM
                     SALVTMLARSTGGWREDPAAFMMWFASHVTRATQHHAALVMLMSPAARAVEHVCEVEA
                     HRADSASEGQLFDVLARGLERLKRGHVYRAFQTRLCDEQSRITVALARLADAAIENEH
                     VSLLQQIATLGRVINSRHRELALRASGPACFACIVDCHPVGAVSSLDWLDDRHGEWMN
                     ASIVSLTITAVKEPSFHDERSFGWICDRMHSRFGPADRGALFGAIVAGRHVRLLLFLL
                     DKHFFRVNAKVITRAFAVCLDERDHLRSVCALLRTADALFGGRPSDVDLWGRVIVPNL
                     YDKRNIRANCIARILCAIALGARADHGDVHVLEGTHEACAEYADGQCWCNPQTEAAAS
                     QVEQLLRWCRPVARFAGTGAERLYDRGRLPTRTYMALHRLAVSNSTHPFRPDPRDPDP
                     PLFY"
     gene            564993..565889
                     /gene="pino_cds_342"
     CDS             564993..565889
                     /gene="pino_cds_342"
                     /codon_start=1
                     /product="Ankyrin repeat"
                     /translation="MGSTTSMLPRPAEHATEEQLPLANDNKRLDVTHLSDDTLREVLA
                     LANELKVPATQLVDRRARACAPCIDDAMVGDPAGWFALLAGGGHIDVLRWARANGCPW
                     DEVAPALAALGGHLAVLKWLHANKCPWDARTCANAASAGSADIVNWLRANGCPWDEAT
                     CEYAALGGHLDILKRARADGCPWSKRVCEYAAGSGDTEVLEWAIANGCPHTATTCSAA
                     ASNGHLEVLKRARTLGCMWNTDTPTEAARGGHAEVLIWAVSNGCPLIQANIDRAARES
                     GNAAIASWREQCPPPTNPHLWM"
     gene            566226..567803
                     /gene="pino_cds_343"
     CDS             566226..567803
                     /gene="pino_cds_343"
                     /codon_start=1
                     /product="Ankyrin repeat"
                     /translation="MDDDDMQQDRQRGAHVKLRSGHRPVARAQTWCQHLLATALWAAL
                     PRFPRALVHVLYQVRAQQHGARHEQASVAAPRPIRRRQRDLPVPDEILCMILAACDPV
                     DLVTARCVGRRWRACAPAVPRFQRRYVGELAFRGYRSVLVWARASGYRWRHADALTCA
                     SAAEGGHLALLQWLRARGCGWDERTCARAASGGHLDIVKWAKANGCRWDKWTCYGAAL
                     GGHFEIIKWGHTWACTCGTSDWPREAWDGAIKGGHLNILQWAYEHGHRCDGKTCSTAA
                     GRGDLAMLQWARAHECPWSAGWGQVSWSTESHSPSPCAEAAAGGHLDVVEWLRANGCP
                     WDARACAAAAKGGHLGILQWLCDNGCPWDWRTCAGAAKGGHLDILQWARTRGCPWDEA
                     TCAGAAEGGHLDIVKWARAEGCPWSKDACAAAAGQGHLDLLQWLRANGCPWGALVCQA
                     AATGGHLDVLKWLRANGCPWDVWTWRWARPHHHVVEWIEANGCPRDGNGHRCNLLWRR
                     ADDAGVSPPPREGHPSP"
     gene            568280..569392
                     /gene="pino_cds_344"
     CDS             568280..569392
                     /gene="pino_cds_344"
                     /codon_start=1
                     /product="Ankyrin repeat"
                     /translation="MTSLAEDGRPIENECQTCLCGTQNGQPSIWPDQTTTGYQMIDIH
                     GRDMATIEKACGNDGHLSDQLPVPDEILSAVLALLKPRDRWRAAGVQRRWYACSGRPD
                     LLEYAKSHACSAGVWWCAVLTGRLDVLKWAFGRGVALGQRSLACEMAARAGHLKVIRW
                     LRRRGFAMGPGTCARAAKGGHLKVLKWALRKGCQWDSSTCTEAASGGHFDILQWARRK
                     GCPWDKWTCIAAAGAGRLDILQWARAEGCPWSKKVCVAAAQGGHLKVLKLGQGQRVRL
                     AQRRVDAGGARRPPQIAQVGASQRVPVERMDLHPRSRMRPSQGTQVGATQGHAVGRTC
                     VCARRRRGPPRRAPVGDAQGLPLGQRRQKGRQVWTN"
     gene            570181..571503
                     /gene="pino_cds_345"
     CDS             570181..571503
                     /gene="pino_cds_345"
                     /codon_start=1
                     /product="Morn repeat"
                     /translation="MTEAPNYNGVAHEMRQACEQDLNIPATIDNLPVEIQVMILNGLT
                     TAPDLLALRATSRTWRLLMSGDRGPVLALCRTLLSDEAVNRLATHPHADVLACIVYGI
                     LKTAGASLRSIEPIDQYCTTYWVPRVMGTFCGWGLSLSKVRVYGAASLCTWSVGRWSD
                     GKLVDGMTRAPHMVPEVPGCRWRPHVLPAGSPCARCRMRRTMWTGRVVGGVAHGHGVW
                     KISRCQECRTNGGRLCKIRCAGRWTKGTLVRGTMAWGGNCVYNGEFKDNFFHGLGTLD
                     ADDHGAGTRYVGHWSMGRPHGHGTLVYERVHDNDPDPVPWHYVGDWDHGVQTGTGTKE
                     WADGRHYTGEWHDGRPHGRGSLKCVDGSRYDGEWHRGARHGQGNQFGPDGRLQRRGYW
                     IHNSLSSRLAFRHAMETGHERSIARCALTAVTHPFHVVSGWLDTPLNF"
     gene            571939..573329
                     /gene="pino_cds_346"
     CDS             join(571939..572188,572257..573329)
                     /gene="pino_cds_346"
                     /codon_start=1
                     /product="Ankyrin repeat"
                     /translation="MQTIQHAVGTLLDPSDGQTRSGGLVTIACNAAQTSTAEQLPSTA
                     TMTTEPATEEDLPVPDEILCMILAACDPVDIVAARHVGAGYRSLIEWTLSSGYRWLCV
                     DVYTCAEATSGGHLALLQWLRTNGCPWDESTCSYAAGEGHIGVLKWAEANGCPLTKCA
                     CGRAARCGHLAVVEWIHARLCPSDRTERLKAAWDGAVIGGHIHIVQWAYDRGHRCDGW
                     TYVVASRRGDLGMLQWARDNGCPWPNAQWPDSPCSEAARKGHLDALRWLWSNGCPWDH
                     HVYIDAAYDGHLPVIEWAHANGCALPSYDRGTLSHVAAAYGSLDTLVWFYDHGCPLTA
                     SVFTSAARRGDLNVLAWLKEHECPCNESACASAAEAGHLDALQWLRANGCQWDANTCY
                     GAANGGHLRVLQWARANGAPWDGGVYWIDHTHTHTIEWIKANDGPDPY"
     gene            573695..574873
                     /gene="pino_cds_347"
     CDS             573695..574873
                     /gene="pino_cds_347"
                     /codon_start=1
                     /product="Hypothetical protein"
                     /translation="MGRSNWGLRTCKKASCDNKPTSALNDGMMHGKQNDVFERIPPEV
                     AFLILAHLDNRSFWMARRTHRVFRLEHDASEVQRRKAYWWLRTSPEQAIARGRSNVLL
                     FLKEHKRIPPNFLPWKAVVEAGHVRALETALTVFPADFGQSAVDGAILRGHTDLVLRM
                     HSLSELSIANSISTALREERTGMVLALCRAASVRYWGLWSLIAARHGHLACLQLFLDR
                     SRGPPQDPVHLAIEAVHADAHNDGGARTLCFLRGRFPDAVNCDRLFDAALYRGYADIC
                     AKILTRASPPLDLQSKIETAAASGVGVGVRILLNLDSALCLQRALDRVARRVKRLRMV
                     PPSTEKPARHDALCALVEADPARALDPRRAFKIFLAGGMLGHAAYLAEHYPSCCCCEQ
                     "
     gene            complement(575037..576323)
                     /gene="pino_cds_348"
     CDS             complement(575037..576323)
                     /gene="pino_cds_348"
                     /codon_start=1
                     /product="Hypothetical protein"
                     /translation="MASRPQCVPFGGRARKRARTATTTTADEPPAVDLFEPAVYRLPA
                     EMLAEIVQRLPPADLVATTASGAPLASAAQAVLGRRLEAALKVAGVSDVDSDPVGAVA
                     ILNNAISHDDATTLKAILNVGFRRAINEPLPPITSMVKWDTPTAMVSYLHGRDRIECA
                     DGLRNRREDGAHREICDVAHDVSMMARDGYTWRVLPHTPLVRAILCGARRCVRTLLSA
                     GARPHPSPEALLGAAVERIFLTHVAIVECRQLGMWHRCPDRATDRVGIVEDLTGAFAR
                     TPPPLPLMDANPLSMLRCGATASAVYDDGPAKSDILFRVLKRIADVGYSPDEPVTQLP
                     SPHLSWCGVPWRTAAAPGGPHATYGSLCLRDRPGDGGPSHRFPTLTERLTAAVIACDH
                     EDIGIPPYEMEIAEAIFAIYEHISPRTAVADEKPVS"
     gene            576864..578063
                     /gene="pino_cds_349"
     CDS             576864..578063
                     /gene="pino_cds_349"
                     /codon_start=1
                     /product="Ankyrin repeat"
                     /translation="MTSTPTAVRTASMRWMRKSTNACRCPNEILEMILVNLDDADAVS
                     AACVNMRWRRALRAPNAPPYRSMHTCAWTLAERGHLATLQWARANGCPWDWKTCAKAA
                     GGGHLDVLEWARSNGCPWDADTCNEAAAHGHLEVLKWARANGCPWDWKACAEAAAHGH
                     LEVLKWARAKRLPMGHIDVLWRARGGHLSALQWARENGCPWTKTFGLGQAAEHGHIDI
                     LRWARAAGCKWGASVWCLCSEGCRIDVLEWSRANGCEWDQSTCAYAAMNGQLETLKWA
                     RANGCPWDDTTCSFAAGNGHIGVIEWARSQGCPWNAKHVRVGGMRWTSRRAQVGRGAN
                     GCPWNDFVCVAAAVFQHLDVLRWAVENGCPCYKAMWSKREQSDLEWVRRHGYPWGRHI
                     PDFPEGA"
     gene            complement(578797..580211)
                     /gene="pino_cds_350"
     CDS             complement(join(578797..579018,579126..580211))
                     /gene="pino_cds_350"
                     /codon_start=1
                     /product="Hypothetical protein"
                     /translation="MFGIDNWSKSDATKIGESIGHILNDRNNNNTRPKKRARTDAYEL
                     PPDPSGTVVDCAIGVLPNEMLQAIVGCLAPRDLPAVAASGAPLSNAARAALDRRLLQA
                     LAVTGVEDADDDPLGALVVLHNAITRDDPVTTKAILDAGLADSINEPLPAIKNMPEWD
                     TPIVAAFYLDNDGTMERSASTPWDGDGKHGYDMAFDVSTSTSSTLRSWRILPHTPLVR
                     AIQCGARRCVRVLLAAGARPCPSPEALLGCALDRIFHTHVAIAEYHSHIHDDYWRSHH
                     AWVPRPHRATDRVGIVEDLVATFARTPPPLPMFDINPLSALRYGTLFSCRKGHGTAGT
                     DLERVLGAVLGAGYSPDERMSRLPPLWARRETDMDTEREAAAIMCVQWIRESGGNAKS
                     MLIPMAILAMYGRLVPRDVALVEPRASTAETVDCGATPCPDGK"
     gene            complement(580796..581899)
                     /gene="pino_cds_351"
     CDS             complement(580796..581899)
                     /gene="pino_cds_351"
                     /codon_start=1
                     /product="Morn repeat"
                     /translation="METHIQIGHAADAQPNFFDILPDELVLAVFAALGDDPRSVLDLG
                     ATCTRHHALSIDDRLWRDMCQLRFGHPLHTRAHLYGKDGRWVYRAQACAGDMHTPVGA
                     VDISTGMYCGDLVDGLPHGYGASMQRPALGRLEGQSMHRRSAADVRASNCCEGEWHKG
                     QAHGYVIYNNADGSIYRGMWENGRRHGHGTTINAHGSRHVGIWHAGECPFGTSTAPAG
                     NIWHTGEWKKGRPHGHGTARLESGATYKGGYADGVRSGYGVYTCTDGTTIQGIWEADT
                     LTGYAICTDHVAGTRYEGMWANNMSMGFGVQSYGDGSRLAALWDGVKHSCGNVATHRS
                     SDVTGDRCANDPCAACVALVVGTSNLPLGRNTP"
     gene            582127..583152
                     /gene="pino_cds_352"
     CDS             582127..583152
                     /gene="pino_cds_352"
                     /codon_start=1
                     /product="F-box domain"
                     /translation="MDSMLPTELMCMVFGHLPLPWWVSAAYVCRWWRMCIQTTWTLRR
                     GPLKTGPRLHFSDTLDDAVRCGHVGAAVWVAKIIGADMHDTAFTAAWMGFGSVRSWEE
                     AAIEAARTGRHNVVLWMARHATSLRESPAVEVTALYGHADCLGRLLSCLPLRIMRAGW
                     RQRIVACALASGNAECADLVLADRRFDVGLSSALLAAIALPRCVEPILSRIHADKYDA
                     HSVRWLAAQNMRPFQTDAPRAYQRGCAADAQGGASEMPAIQTGGDTQVAPWPPMRSLT
                     VDDFLHGGALSDYGMSDDVRQSSRHLMHALMTPLVLKGGSYIALCKCIEMGIPTLPIP
                     YLRLRRP"
     gene            583855..584109
                     /gene="pino_cds_353"
     CDS             583855..584109
                     /gene="pino_cds_353"
                     /codon_start=1
                     /product="Hypothetical protein"
                     /translation="MFFVINIIAKEERGLVYVLCAFSNITFDGGKLCAARRTRVYGVW
                     SSTSAMVGICRVRMPLVAHVHPDGVDATPRSFCVGAARVR"
     gene            584214..584999
                     /gene="pino_cds_354"
     CDS             584214..584999
                     /gene="pino_cds_354"
                     /codon_start=1
                     /product="F-box domain"
                     /translation="MASHRACSWTQALVEATRAGRDDVILWTACHVLPAKESFAVEVA
                     AAYGRTVCIEKLLCAVRTEWSPRIVACALASGNIECVDLILADRRMPVALPSVYIAAI
                     TLPQYVGTLLARAGLPPHIDDASAVRWLAAQNLPLDGSSSTMVVHPRPRRGAPSHPDV
                     DVHAWPPMRRLHAADLLPGGALAGYCQLTYWSTSIRTYICDLLDPLLVGDASHHAREC
                     IFRDALPWLPTKSLSRRRRPNCMGMRVNFTARAVIRPDNDELG"
     gene            complement(585365..586555)
                     /gene="pino_cds_355"
     CDS             complement(585365..586555)
                     /gene="pino_cds_355"
                     /codon_start=1
                     /product="Hypothetical protein"
                     /translation="MDHTIEATSDPCAGRSLPDVPREIIDVIISYLGRGRDVASCCTV
                     LGESASYAMLRHMRTKPLAWVRAGAPLAVVEALLAKAAPCGWVSFGWVEAAACGGCLD
                     VLVHLHTLANVDVAQGIGFNGWAYVWRERRRLIFERMRSLLKAAVVGRGGSDVIDYIL
                     NVYDAPRFRSRRLFNSGMLIRLSRHAINHRSDAVDVLEALHSHDKRGKCGCTKKLAYD
                     AARADRPDVLAWMAEHGCCAIADAVQTTCRTVVEYITRRGHNGLFTTTRRYTYPWSEE
                     YDDIALAAVRAKAASAVAWFGPKTDRRRIVDAVCRLDDSTCQDRINLIMGGSLAKTAL
                     IMFLEALVALIKCRCLVWMTTPWRLLLLGTMAACAASKCAKDCRRPDLMPSTSAEPEP
                     PKAF"
     gene            587241..588395
                     /gene="pino_cds_356"
     CDS             join(587241..587566,587714..588395)
                     /gene="pino_cds_356"
                     /codon_start=1
                     /product="Ankyrin repeat"
                     /translation="MQMETVTKNDFEPQGDMPVPPEILTMILARTDPVGQFVSRWVCR
                     HWAAYAPAAMRPWYGFTTAAAACGYTRVLEWARANGCPWDTTTCYAAARAGHMRLLVW
                     LNKNECPRRRCPWDAETCRAAARGGHLAVLQWARANGCAWDERACDEAATAGHLDVLK
                     WLVANRCGLLWTTPRHAAANGHLDVLMWIKDNIHSNAYGRSVCTAAASNGHEHVVEWA
                     QNNGFLWDDDTCIKAARQGNVRLLVRAKRAGLPLNGRICEVAAKHGHIAVPAWARENG
                     CPWDERACRVALCRSDLSVLKWLRGNGCPWDYGTRIMARVFYGDEVPEWRLPLPRSFH
                     V"
     gene            complement(588511..590544)
                     /gene="pino_cds_357"
     CDS             complement(588511..590544)
                     /gene="pino_cds_357"
                     /codon_start=1
                     /product="F-box domain"
                     /translation="MDGNLSLLDLPSEILCSIVALLDHPRDVVACACTSAALACRSPL
                     DAAASYYRGRSEAALAAGLPLCITRALFEQWNMAADYRHLPAAARGGRVDVVRWVCRL
                     IRHLTTPGLWTDLTVAGPTFVPEYAPAPDAPHRQDGHCEEPPAPRQIHQTHARAARRQ
                     DYAGPLGSTRDDGLVGPSRHRGLTNPATDDGAVGLLSPRGQDVIYGDDQDPAVSPGNR
                     HPMPFPPLHLQGSLNVVPVRRRGGPPQGPPDGASGTLQLTGDITSMPYLLQAIYEAAC
                     AGHIDVLRYLTTACPLAKMPCALDVRVVVEAARRGTLSTVVYAHDRWPFVVVVAAGRG
                     GTVCSCPVDIADAAVAGDQRHVLDWMHAVGCQAFACTTEQLARAISAGNDRLVDAITR
                     TLVDEARAEGQDVSDTQSNTSLLCAGALIEAAQNGHVRTLAIAHARGFVRLTADVMGA
                     AARAGHLDVLRWAAGETVSGVEACFAPTTRLPWDDPVVAWSAAECADLGSTAVVDWLV
                     ARPETRHHFTGVMARTMLARNRPAIVLWMHDTGILSLSDWEGLEIAIKSGLTCLGDMI
                     DRGAVCSSRTMAAALMHSPNADAIALLCERFGYDDLYEALQMHVHATDNAALRWVRDN
                     VPNADVEHIIEAGKAHASARRPERSPAVRHATIQHRPGIVLIPPRANLPEPAG"
     gene            complement(591079..591995)
                     /gene="pino_cds_358"
     CDS             complement(join(591079..591284,591461..591995))
                     /gene="pino_cds_358"
                     /codon_start=1
                     /product="Signal peptidase"
                     /translation="MKKSDPRKMHTQKHTELSPAAEATHRPAESTQRTLSTLATVFWA
                     VYPILWTCGVCLAISCFLRCTMPLASVTSGSMEPQTRRGDMLLVVGPDFDGEVRVGDI
                     VLYRLPHRPDTPIVHRVVEIVDEKTAARGNRRVAHDAGSATTRLWYRTKGDNNDVDDA
                     GLVSPSFPSGLVPHDALVGKVRGQIPLLGYPGLMPVSAKIAVALGCVTWLLHGLWIGT
                     AASKPDDNNEKDQVSAWRWALLVLCPLF"
     gene            592483..594594
                     /gene="pino_cds_359"
     CDS             592483..594594
                     /gene="pino_cds_359"
                     /codon_start=1
                     /product="Hypothetical protein"
                     /translation="METLPREVIFHILCAVADDRDFLACLCAARLFSVYLRAHVLERQ
                     CRRCRTAVAAARRLSPEALDYFRRTRAERFDSAAIYAAASVGRVDNVRWLHEHTDAPH
                     RPAIAQGTVACGTRILDAAVKSGSATTVAFLLDQGYQAPRRAFVEAARLGRVDLLRML
                     DTAAPGCDMMHVARTAAASNHTDAFLFAAGRIDTMTRARVRSLFAYTTLGPAGGPRDA
                     HFALLLIEHATLDSEALSAALQVAECGADNPNVRARLEHAVAAVCASRPRDRRIALGC
                     LPLDAANYVDDPALLRVGPAHITDCMLMRRCKERAIQVDDVEATRMARTMWDVVVPDA
                     LVSLVARWFSEGGDLAGAAAVGWIFETSRPPRAHTLGGASLADVLAFALVHCRERCDL
                     DAWMSDFAATDEVDLLRTVLNISGSSIPDDAVRVAATYGSMGVLRLLTECGYRGWPRD
                     LVVCAARSRRLDVVQFVHGLAISADRSALTCAISMGCDDIVLFLCDHYDRVCPQMAMR
                     MAIIKNHHGLVHLLCNRKDTGTCRGGPMCCAADIVATPRLYEHVDVDAFFTHCADMRP
                     NWITLRDAAGLKNGSLLCRLVAKHHYDDDDVRYALAGAVDAGDSRAIRRITAHFGGRL
                     ASMPWPSRLVDICQSEHTPMVSMDALIRHAPYLCTAEDLVRRARPHTECGIDGRCAAE
                     DQFLQMLCERLSQSSDAMVAT"
     gene            complement(594897..595250)
                     /gene="pino_cds_360"
     CDS             complement(594897..595250)
                     /gene="pino_cds_360"
                     /codon_start=1
                     /product="Hypothetical protein"
                     /translation="MNKRALLDRFLPLPEPRIRKGLMVTNNPHHTPCAPGLAIGPQAI
                     TRLDYDGDSVLDRVAEWFSINFASYAVEHERALWEEKCERYWHASAAADRDLFPIHME
                     NIRGRRRLPEIYRDR"
     gene            complement(595500..596294)
                     /gene="pino_cds_361"
     CDS             complement(595500..596294)
                     /gene="pino_cds_361"
                     /codon_start=1
                     /product="C1q domain"
                     /translation="MDNHHARQCRAPSVCPGHAAALTTDDTSAHAAHARPCCMFVSRV
                     GTRGPSGPPGLLGPTGPAGAQGTPGSVGVTGAVGPRGPGGPGGLPGPPGAVGQQGPPG
                     PAGPLTLSSVAFRADGVATQVVTATVFVTVAYENEIYDLQNGVAADNYDPTTSTFTAP
                     LAGVYRFSAMASGTLVTGNPTVSLVLTTSAVGQGPTRALFRVFDIADADDNYSANVVG
                     DFQLAVGDTVTPQISLSPDGGNFTLAPATTVTRTFMGSLVFETPPP"
     gene            complement(596398..597147)
                     /gene="pino_cds_362"
     CDS             complement(596398..597147)
                     /gene="pino_cds_362"
                     /codon_start=1
                     /product="C1q domain"
                     /translation="MKKAATDCVTVACVAPCAGAADSRVCCTIAVPGPRGPTGTQGLV
                     GASGAQGPPGAVGAPGPVGPAGPSGPPGEAGPAGPPGDVGPMGPPGPPASVVGVGFRA
                     IKTTDQTGGPGTTVVIEYVNEIYDLQNSATVDNYDPVTSTFTAPVAGVYRFEAPISPT
                     WAVEGTNAIVSLVSDSGAPPIERWLAMPALGEAFGTAVEATVSGDFLLAAGQTVAVQI
                     ANIGPGAFAVLGVSPPTQTSFTGALVAPVEP"
     gene            complement(597776..599455)
                     /gene="pino_cds_363"
     CDS             complement(597776..599455)
                     /gene="pino_cds_363"
                     /codon_start=1
                     /product="MORN repeat"
                     /translation="MRMRRSRRSSEGDRVALVKGHPMGDCTDTDNHDTADIDLEASTT
                     VADHAFRLTSPATWCAGVRACLHRRRRCGGDKVGANTQSLIEKEEAVPPKDGDVMASD
                     GNSGRAPRVPPRPAFLRQRHDGWRRVYADAYGVDPESGDPLDHAILRRIGASWRFLWE
                     CRQPINDVPHAPGAIVQGQCLMPNGDLVRGTFCVRRLPLAPDMSTCPLGPTLSPTGRS
                     LVCDDSWAVAHDAVLPPDPSSGGKDNGNDSLQPDGTDSVRCQQGDDAINCDNDDNYGV
                     DRDTDGQHPCVSCARIVCPLHPAADGDSSNNNNNDGDDKDRNADSMDDTSRCAGDAPK
                     PGDKQSTDMVLVPHGHAAAVGVDGSLIEGAFCMGLLHGHGRTVDARGEIFGRWRHGRL
                     HGRAVATTPEGWFVCATWRDGRLIGDYFAQSSYGEQFRCASCVDGRFDGRCRRGYACG
                     DWAIEQWADGAFVGIEQFRIAPVADMDGLVDGALLADCAWTCDRVRGGGGSRYDGHIY
                     YPADPASPEFALFCAYMTSEASSRAFTAGQREAFVWAMWMAQRRAAAASSV"
     gene            complement(599949..600782)
                     /gene="pino_cds_364"
     CDS             complement(599949..600782)
                     /gene="pino_cds_364"
                     /codon_start=1
                     /product="Hypothetical protein"
                     /translation="MTSTKTKTQATLCARALLPMAVLAALAIMAVSSVAALSPNLECP
                     VSPAYPKNYFRDNLPAQNGYMGTTAFRAYVQVDVTGEADLVKVGNFTGVYLRYYSRTQ
                     RRPIDEYYINVDGTPVKVIEITTTPLGDAGQFCLNFDPPTDVSEYVEGVSLNDKFFFN
                     AYYNKTAKGARFGEITRTEFFNFARNGVDSLNILTVLDNEDASIESMTFFRFHKTSNH
                     VVERCEETDEVKAIDGVATRSVHDYAQTFELGNGMRVPLDVIKPAYRGFFPAAAAAAA
                     A"
     gene            complement(601542..602662)
                     /gene="pino_cds_365"
     CDS             complement(join(601542..602459,602528..602662))
                     /gene="pino_cds_365"
                     /codon_start=1
                     /product="F-box domain"
                     /translation="MQGMDQTTNLPDEILAEVLSPRRMRPSDIGSARLVCRRWGVAGG
                     DWTAMAALVDAFVNDSVPMLLRVMDTDIIGPNDPIDVGGWRRLGVGVATVDIGLAHGS
                     DDRIVGLSPILAEDDLVMSIPPLVVTPLVAAFAYGALRCVRTLIGLGAYPMPHVDALV
                     TFMIERCAWASASVMAIAPGDSTAATPRPRNNIAVVMPRRPMDIAPALRMILDAFPRD
                     RRTPSGIVPPLHALVRAATDASLMTAEENLDETTLTDAVLQVAHVLLDAGYNPRAPWR
                     CRGPYEMSHVYLYDSCPASTVSVNDARLAAIDQMTAETPYDAALRTKDRTRSRPVGQY
                     GLLDALAAAYAAHGAS"
     gene            complement(603065..603706)
                     /gene="pino_cds_366"
     CDS             complement(603065..603706)
                     /gene="pino_cds_366"
                     /codon_start=1
                     /product="AldR family transcriptional regulator"
                     /translation="MQRQKAQMRRGPAPVDDTYGHPPARSVVAHTRAPRHRKPDGDAD
                     RDRHPVRRQHDDEHDDKRPRRRHQRRNSDGGCCYSIPTPAFAAGDVDRLGYAQAVRCG
                     DAVWVSGTIGRDECDRLVTGGMRAQAEQVFDNLTESLKAAGCRGLEDVVHLTSYVVDI
                     RRNIGAYVAVRARRMPDADFASATVGVVGFPTLGALVNVTCMAIPHRGCHHRA"
     gene            604719..608048
                     /gene="pino_cds_367"
     CDS             604719..608048
                     /gene="pino_cds_367"
                     /codon_start=1
                     /product="Cathepsin c1-like peptidase"
                     /translation="MRMSITISTTTNNNGCVDRGGHEDASSDSDYERRDNAPERDRRR
                     DDRRGTARRRRRAPAVASLLPAYTPPPRIPRAVRVSTMVVGRSATATLSNVVQGGAFY
                     APPPLPLPFGSGDVSDPSWGPPAVSPAPSWPTQAPLGTGSPMYGAPAMMSSSMQPPPS
                     PWPMMASPSPIMDQMRAALGAWPPVGMEVPPSTQPPSSISVASYGGSWQPQSFSPYPA
                     QSTATPAQSPMQLSVQSPMQPPVQPTMTQPPPASSPPAALGASPTFVVGQARPSLTLL
                     RSSPSVNAQYRQLLAVNSGRLIDEVKPPPNFDGRKQWNGLLSPVLDQGQCGGCWAFSS
                     AGVLGDRFAIHTKGAFGLALSPEHLILCGFSKPLAVGNVSEADRATVQAEIATLQADL
                     AQAQQLNATFQGQVACYGNTLPLVSEYLYRYGTRSLRCDPYTLGNIQPGQPLPSCRSL
                     LPTIPPYERCKTEDRIDRIYRAIGRYFVSSDEGAGTNLTGQGLQMQIEAIKAEIYKFG
                     PIMAGYEVFRDFMQPGPTNPSWATGIYRYDGVSPKDGGHAITIVGWGTDPDSGETFWI
                     IRNSWGVAWGEAGYFRMYAGQCGVEHNTMAVIPDLPGLTVPAEYVERFIVDEDSVTVR
                     ALVDVDPSGLPASYVRSLTPAQRATEAQPIVHPSALPEYGSFMAGRIENPHAIPGAEN
                     PIPVGATRFVTPPALGTALAEAAADPMGGGGGMVVPLSHHTTAPSVPVTMVQPASSSS
                     AAPSSLGAAGPTTSSWAPQTPMQSGPTYPYPPAQAQAQTTAAGAPTGYNGTPVPMPWP
                     DPYDAWGSPAMAYFGAEQQQQQQTMTTVATTTTVDYGAPHHNQHGGRPQGGCGCGPCG
                     PSAPQGPTPAPACGPCSGGAGPFPMINALMLAEACAGHGKPTARPCPLDAVTTPTAPA
                     HGVDYRVDIDGHQGAGTGCAPETGHHQAAEGVPCACATCQPVARPRARGRKDKGLATA
                     AAYILVPVPEPRPPKSARHARPVGSSVATKGRHQAPTDTEGHSSSRSTTASRSTASGS
                     SSGSSSEDDSVSYNPSAFSLAPSSPSSIRLDAMSGDQSSGSRHSASDSGSDSDSATKD
                     ARARRKSRRHRRARRVSARRR"
     gene            complement(608720..609739)
                     /gene="pino_cds_368"
     CDS             complement(608720..609739)
                     /gene="pino_cds_368"
                     /codon_start=1
                     /product="Hypothetical protein"
                     /translation="MDAHSTPICTDDAGATSAPLTDATRADDSDADTATGQVLSWESP
                     ALSLALERVFEFYLADENDLHLQGKTSAVARAEAARWIAGCAAGPLRGSPEFCAAARR
                     AIRTLSLVCKRIAGVCVHQWAGLAGLDRPPDDARRGTRGSITLVVGGAGSGKTYASSR
                     LCPAMSQAMARASGRPDAPCIAADSMIVNPHSDRPLDEEIDRNLSSRPHARAALVHAE
                     GRDYMGNAEKLCARGFHVVVVGYPTVDAPRPDAIDRVLVALGPRDSRCRYDALSANVA
                     TIVDVDPAALKVLFDAAPDYAYLWFEPVDGRFGPDRTVVTLSRASDFETADGSAPPLD
                     PCQVL"
     gene            complement(610437..611222)
                     /gene="pino_cds_369"
     CDS             complement(610437..611222)
                     /gene="pino_cds_369"
                     /codon_start=1
                     /product="Alkylated dna repair"
                     /translation="MQQHQCDSVPGPVRPRRTIQTLLGVKRARDNTQSPTAPNKAPPA
                     TAAPSAKRARPNLDPSACVTHMRSVLTAAEAREAMDDLRREVTTSQGQVRVYGRVLDE
                     ARLTAYHVRRQWADPRLADRPYIYSGKAMAGAPDFTPVLERLCCRVEDALGKPRGTYT
                     AVLINEYRDGDDHISWHSDDEHSINRDDIASLSLGATRDFKMRDKTDHTLQVTFPLGS
                     GDVVHMHGACQDLYQHQVPKRARVHSARFNLTFRQLADHSTPK"
     gene            611717..612142
                     /gene="pino_cds_370"
     CDS             611717..612142
                     /gene="pino_cds_370"
                     /codon_start=1
                     /product="Hypothetical protein"
                     /translation="MKITAALFCLGALLFFAATVDAYGSAAITCSGVPFSLKWDTAGA
                     TDKIQVVSLTATVPSYVPGAIQCDLSALPAAFQSNLGTRNFAHGTSVNTYGAVGTAFF
                     AKPAGGNYAVTLYSDITIGQGSGFQAGTKINDSFTWISA"
     gene            613077..613583
                     /gene="pino_cds_371"
     CDS             613077..613583
                     /gene="pino_cds_371"
                     /codon_start=1
                     /product="Ankyrin repeat"
                     /translation="MHYEGVCAAWTCRQWRALMRPILDRGISFWHVNRRAIFAEHAAG
                     EGRLALLKWARARGCPWDASVCAAAARHGHLGVLQWLRAKGCPWNDLTCANAARGGHL
                     AVLQWARANKCPWDNWTCGFTAASAATLDILQWARANGCPCVDDCDVCVLCLSGARCA
                     PGADIFYP"
     gene            613643..614272
                     /gene="pino_cds_372"
     CDS             613643..614272
                     /gene="pino_cds_372"
                     /codon_start=1
                     /product="Ankyrin repeat"
                     /translation="MSAAGGGHIEVLQWARANGCPWNSTTSSSAARNNHLTTLEWIVV
                     NGCELGKNACSYAAHEGHFAILRWLRKHDCPWGKETCTGAAQCGQLDMLKWARANECP
                     WDATVCATAAAEGHMDVLTWARANGCPWDRRACAWAAKHNRVDVLSWLRAGGCMWDDL
                     ACQLAAEMGALEALEWARANGCPWDRQKCRAKALRRRDKTMVAWIDAQP"
     gene            complement(614816..615676)
                     /gene="pino_cds_373"
     CDS             complement(614816..615676)
                     /gene="pino_cds_373"
                     /codon_start=1
                     /product="Hypothetical protein"
                     /translation="MIATGHDGMLRAVLAEIDPALVVPSVEIDRALHSASTLGSLATV
                     TIAMEAGLVPRALPLFVGAGAGGHIALLDYAEARFGAPRDALRGAVMMAAETWGQGHD
                     TVRWVAARRSDVIDASIMWVAIARGPVGVVRAIDDALAGSFDWQRAAYAVLKSQNMKL
                     LRYAVEEKGMVVDAMSIQGRLRLTPKMTKYLISRYGVERMQPVLDTVSILNADRERGG
                     WSWLDAVSGACTAARYVATSAHALCSGTYSGESKPCTCRRCREPDGPRSTKRRCVDPS
                     AAAEAPPFCS"
     gene            complement(615691..616545)
                     /gene="pino_cds_374"
     CDS             complement(615691..616545)
                     /gene="pino_cds_374"
                     /codon_start=1
                     /product="Hypothetical protein"
                     /translation="MADTDITDAMVGEPRTPTTLADLPNEVRDGILRLLTKPRHLAAA
                     RCASHLFDGGDMEALTVRWATRHLFVLVKSRAPLALVAAAVNANVHVVEFDTLFDAAV
                     GGRIDVVRLVHAALEVPLPLLPKISKAAFSFCCFVGGSPLTCAFCFSHSFFFGGTGTH
                     THTHTHTHTQRRAPTPVPRAPCDLLSIIRAALRRGHVGIARYLINRAIAGVRYDPRDD
                     VSLVSAAASSGHAVSVAFAHDRLPPGAGSAPCACASDLGDRAWWATLPDAALWLKSMA
                     ALATRRPP"
     gene            complement(617002..617756)
                     /gene="pino_cds_375"
     CDS             complement(join(617002..617588,617738..617756))
                     /gene="pino_cds_375"
                     /codon_start=1
                     /product="Hypothetical protein"
                     /translation="MCSERAKTNSNVDCRLSLFFLGGHVDHASRHRRVLGEDHVLPYV
                     ALEWDHTVGRPAIKWLVVRCADIRSKCGGPQSLAPRADVAYFCHPETAEADARLFARL
                     KNAAEPWPPGAPVASPPQALQEEEEKEPATDLARHCVFAWDHLLLNEAPLRWAVLEWV
                     GAEAVASAPDIAHPRTDVAYFLDPATAEADAHLFAGARDRR"
     gene            complement(619137..620426)
                     /gene="pino_cds_376"
     CDS             complement(619137..620426)
                     /gene="pino_cds_376"
                     /codon_start=1
                     /product="Hypothetical protein"
                     /translation="MATWATLPVEMRAAVLDHIDNSHDFVACMLASRLFYEAATDTQR
                     RMWRYAPERPNAPNVFNSDEPLGVMAVVWNRWAHRLDFDYEQIIWGPAQSGRVEVLRF
                     VCAIVGPAGGAQPTGPCLCSRWRKCACDDPKRIDEMRCNAFRDAVYAAAASGHTDAVL
                     YLMNVIAALADLRPHALCAAASHAQIHVIEALSMDRPPDRKTIVQMVDNALDHRHPHV
                     ALWLHDRGWADAEVIAPFLARLLLVAASCACASDFEGAWRLVVLREPDSVRRWWRTAM
                     LKAGPSGNVSALGWLLGSPPDTGMPPSSSSASVASHALIGAIEHGHLDAAVLLRERGA
                     TVSLPMFQCALQWATTNGHADAVVPLCEAFYDPALMKGGHRLVECACEMDHVGLLAFA
                     CERFGAHLAVHAWRWARASANCKGGRVLLWLNKHFPS"
     gene            620799..621915
                     /gene="pino_cds_377"
     CDS             join(620799..620806,621009..621915)
                     /gene="pino_cds_377"
                     /codon_start=1
                     /product="Hypothetical protein"
                     /translation="MARHWQPDERRPDGQRQSQWQHYGGNGLVVGAPTVARLPGFAEH
                     HAPRFSVSAECARRAGRSARVPPEEATCANLVPLYTSFWSALNDLDAAVPGGIVGLRQ
                     PLTPLSVLATAGRAVPPGYRGAALAAYAIDTLMRLSAGETWSSSPPPPDIFLDGAASD
                     GDNAVGDSDGSSSLYTIHVHAPDGAGTRVSFDRAGQRVATMQLDPYGSVVGFEAHGRR
                     RLPRRVRGVAEAAMRALMNDAVRQVVDSGATSGAVALSESQLVWDLLSRSRPGATGRR
                     GRHRIMERSYGGVNGDYASEEEGETGDD"
     gene            complement(622339..622869)
                     /gene="pino_cds_378"
     CDS             complement(622339..622869)
                     /gene="pino_cds_378"
                     /codon_start=1
                     /product="Hypothetical protein"
                     /translation="MGVATSSIITTDGVTVVARGGDSAIVIGGGSGTRVNTSSTSLCR
                     RGASTITNGNVSVISRGGAVSMSRNQGSGSHPVDIITTSGDETEIIYNGPPAVYVTVK
                     NTGGDCTLTQVVEGERVVTRIAPNTTVTVSPKGQPTVVPLSPRPRPWRRLFLPVGVAL
                     ISLSLGFYLFSQQQQQ"
     gene            623551..624258
                     /gene="pino_cds_379"
     CDS             623551..624258
                     /gene="pino_cds_379"
                     /codon_start=1
                     /product="Hypothetical protein"
                     /translation="MDILRLGDLPDELLAAIVEDNHGGRVGSVERPLDDCDVFALTAS
                     DRRLAAAASRPHRAARKRAALTRQRTLLLRALCTAAADGEVTYVYVGEALYGGIPTYV
                     GLHLTLHRHGSPPSVYWTLRGDPLGDTPSAPADVIGATGLWPRLDDRDFVDRLFDTWL
                     TPTMGSPRTTRVHAVVRPATSGPPADMHARLCALFGGDSWKPGAIGLVGVPLDDVWAR
                     WCASTAGGDNVPFGTHR"
     gene            624665..626507
                     /gene="pino_cds_380"
     CDS             join(624665..624980,625273..626507)
                     /gene="pino_cds_380"
                     /codon_start=1
                     /product="Hypothetical protein"
                     /translation="MADTCDTHMTIEQDPGAMVGSVVSPHAPSNVCEDVTLADLPREV
                     LLHIVQFIEHPSDLLATRMAARLFDMIDVARRAAEWATCPAHAGAVIRSRAPVTSSQS
                     RCLFIRCDAPCRPSRHHICQALCGAARGGHVDIVRYLLTRRIGDVRGKDCDWGVADAA
                     ETGNVDLFVFAHDVYSLPATSTKPCRCDAEVGRAAWDAVQPDVALWMRDFGCAGYCRP
                     VFDQLTEALIQGHDSIEAIVRHMEPITDPELVQRLNNAVVSVNAGHHAAMMAAIDHGL
                     PIDPTALFMGAASSGDVKALALVSDRFPLTRHMVRSAIVAAMAYNDESDSVHWLTQQW
                     PDAVDATLVTACIIEGTLGKVRALESVLDPPYNWQRAAGAVLASQDERLIAYAIEQKG
                     VVLDESIVLTEGFVPYAPAVAYLIRHYGREHTQALYDMGAALWRRQQSCEVYCLEEVA
                     EQGDLCVAAYAAMFWAHDRDHESQSAPTCACASCRGPDGSRPLKRRRVEPSSSPPPQD
                     PLDDAHHE"
     gene            complement(626811..627803)
                     /gene="pino_cds_381"
     CDS             complement(626811..627803)
                     /gene="pino_cds_381"
                     /codon_start=1
                     /product="Hypothetical protein"
                     /translation="MAGRLQEGDTSMIAQARFRAPRMNGGNPTGAIAYDGNDNKRDDK
                     KVPVDIDPLNMVSLIDPYEPRPLGGDGPFDVPAAGECLRLLEEAFVVALHDDLVAEIV
                     ARTADALGAREPTALIGAYARLGAQCDRRASTHPCATQPRCAMCTRAPTVSAVAAALE
                     RLDALDGGLGARASIVDARRRAMSRALAQPDDVPAAVDAYRAYLHATYATLYARYVAF
                     WTLVVRQPTLARASACRPHHVGLSRSAPGWGGARMLTPRDLAQALSASGQHALGRLLM
                     AQQRMEPVYTLDPPLTLSPLPGAAPMCRRWAALPEAGIVACAVDIHEDLAMDDA"
     gene            complement(628357..629301)
                     /gene="pino_cds_382"
     CDS             complement(628357..629301)
                     /gene="pino_cds_382"
                     /codon_start=1
                     /product="Hypothetical protein"
                     /translation="MDSPSATDNTLGDQAPHERTFDEALMECARFMGVEWPLPPTHDL
                     ATLMDNTYDPAPCQTAPMFQLSIDNDVPRGATCDDNLYFGNDTADRVKYKNNQDGDPA
                     HAIRDGGNGRDNAGGHSNGHAHAVPTLDTGDTNDVIILGESAGAGPHQRRRKRVEVND
                     TNVGSTSHYAGDNHNHKRLCIRSTQPNSGPDSTDSEGDDDGPSSDYVNKNGPCFHGGC
                     KIRRSFMCTHGMCAVHCRARQRLLGPTRGRCASVLHSVVVWMERCAVAGCRGRTSVGC
                     RSRLCTRHCVALTPAAATCDNTPHHKAIRKSRCAVAIR"
     gene            complement(629909..630523)
                     /gene="pino_cds_383"
     CDS             complement(629909..630523)
                     /gene="pino_cds_383"
                     /codon_start=1
                     /product="Hypothetical protein"
                     /translation="MASTAINLAPTVPQQPPHTEHDLFARAIAGLAMWPPAPAWDPIA
                     MRRRCALYALCAVWHASHRIDAPSWRASTLDQLDRWAASLVVPVVVCPGGVARSTTVS
                     APCTAADTAPAADDDKSPYCAGNTDGLRHLTDDAIADARTAINERLLHALVLAAVETG
                     CAVDVAAVCPRVGVDLFAAWPTAQVVAVAGSHSPAVDLAVVLAP"
     gene            631955..638418
                     /gene="pino_cds_384"
     CDS             join(631955..632023,632144..632888,632980..633825,
                     633918..634282,634354..635466,635578..638418)
                     /gene="pino_cds_384"
                     /codon_start=1
                     /product="Ser/thr kinase"
                     /translation="MMTVPLARRHSGDMLFSRCRILWGSVAVLVLLLLLALASRTVIA
                     DSVCLPLDRTDDTSILPVTPTKTLLFEGTTVGGDALDVVASAYVFTVDDVAVQRLGTD
                     AATATADYDSGRCDAVLLAAQVPRALAGRVQLPVAAYGVEIVYSFAGGSSMVIFDGLP
                     LFAAVWAGTITRWDDPAFGAGSGATPDMLPPGEVLIAVERFPSGSLETEAASLGATFA
                     QALSSASPAFADAYAAHDGDLAALVLATAGTNRTLLVDPVTWSVVVPAGTTPAGGDPA
                     TAPYMRPMLTNRAGRFFFGWTPSAAHAAMDTYDALSLRSIPYNTGVTGLSNLEDPNTW
                     PVVGLVTAVLRTDAIPPGYDCQYAEAALDFLAWLEINDGALAATLASRGLVPLSSNFR
                     QRATDMMGAISLCASFSSSSELSGDMVPSSTLFNATTETDSTGKRRQDTRTTTGVTAA
                     ATGTTRVAAAALVGAGTTSLVWSGWMQAYASRVGAASAARLKLAEDGEDEAISRLALY
                     GVDFAVTSAGATDLTPVLAAACVDCMSVPMAVRPYAAIYNVPEIAPNPSLAELLPGRQ
                     IIVALAKGGTASGAGGPLGGVANRFAAEYMAADVAFRAEVLNGSATTLGVASIVYPLE
                     SSAPARILVAGDRVAAAVKATVYSVGVDAIEAALAARNPSLADLRDANGNRSCASCRG
                     LFPNGTIIVIIIVTVIHRHPLYRGRHRIVPRANATLPFAGLPAMMVGAATAGAWPVLG
                     WHHAYMHRETTPDCAKAAGLVDAFYWTQTSNDGAAVAAAQGLATSARVAGPGAAARII
                     SALAGITCLSSAGVPVQVLGVAPCLYVPPDAADPAVAVTVCANHGQCLASASPSTPPT
                     TTTTWSAVACSCDAGWTGAHCEMVDAGSASGDDGVSTSVIVGIAVGVAVPVCCGIVLL
                     VLLVAALVVVRHRGARRRALDSGCEIDPEEITILRDLGAGATSNVFEGTWRGTRVAVK
                     RFHTPARGWDRTALAHFSEEVRVMCTLRHPHVVMFMGASTRPPVLAIVMEHMALGSLR
                     DVLDNELLVQIPFKLKTKVSDFGLSSMRGGNNNGNNGKGGGLIGGGVDAQHQQQQHHN
                     LGTVHWAAPERIRWASGGDEADVQAADVYSFGIVLWELLTRDSPYRGCSPAAIQVAVM
                     RDGMRPDAHVARAALAAEGVDVFDAARYEPEDDISAAVENEAMPRAIVAAYVGLYRSC
                     WDTDPSARPTFLGVLSELGELAGLVQDARPYGASDSSSSSATHYMPGATGSTNRDVDG
                     SRTTDTLTGASGSEATDGGGGIDSGMLGMAGRRKAGRAPDGMVVIALADVAHAATLWE
                     TAPAAMATATLTLFQTLRRLTARYGGHESAQAGRTTASLFCAVFVDPCAAVAWAAASQ
                     RLLASDETSWPEDLLACPEAAAEYPSTASTADIESGRARPVYRGLRVRMTLHHGHVRR
                     VSCDPGRRPEYDGEGLKEALRLTPRVRGGHVLVTEALCRCLLERPRPVAERCLAAAGR
                     IERAAGGAFGDSRARRRARFAAGAADIISGTTATSGGPLMDGDGRTLSASPLTPVRHA
                     SCTVRALADTDDAVAQAGALAWRNIEWMLKTCDEARRRRGGSSGLHRSGHLLNDKSDA
                     SPSDSDDDDDGSDADGSDQEQESGSRVRTLLCQIRVAQLDGRWTDHALLATGTDPHHR
                     DDSDDSDDSDDGDDQEGRGQRGGHAWGDGQQGELRYVDSANMCRPVINPSTLNKMGPV
                     IGAGSFATVHRANWYGAHVAVKRLARWRLTEEDILRFRAEVMVHSKLDHPNVLPFFGA
                     CLQEGNLCLVTEYMPRGSLRDLLASPEGGRLGWDVRLRMLRHAARGVAYLHARSPPIV
                     HRDLKPANLLVADQGDRIVVADFGLARIKEEGATVTACRGTRAYAAPEMLLSRPCTEK
                     VDVYAMGLIMWSVLTRREPFADRGAHDAEVYADIIGGTRPQVPSDAPEDFKVLMARCW
                     NNNPSRRPTMQAVVDALTDMIGDGHAVDVEMGLV"
     gene            639105..639467
                     /gene="pino_cds_385"
     CDS             639105..639467
                     /gene="pino_cds_385"
                     /codon_start=1
                     /product="Hypothetical protein"
                     /translation="MDNTSDDESVECGGRVFERNPQARTTGQAGTSDGFNSRRPPDPT
                     PEQIAAQMAFYEQYNAVAYASRHDGDEPGPDKPEFDWEAFDQSMGDAPECDGLLADFL
                     AEKAARRAPLEGDLPHRE"
     gene            640011..641876
                     /gene="pino_cds_386"
     CDS             640011..641876
                     /gene="pino_cds_386"
                     /codon_start=1
                     /product="Hypothetical protein"
                     /translation="MQEGANANDNNNNDGSTLWALLPIELVAMIVNGRDRHGRAFMDP
                     RWRPMARMACRLLRRAVEHPTLWDSDALGDPQRLFRYPMVGTGDHYVEFDVTHARRRR
                     WRHGMIVCASAVAEWLRDAPMDLTGQYADAVAERMVAEWGASRAQAHVVLLATGRSDA
                     VAYALDPTTSASFAPIPPVPARPARALRPHSAGWQPDGQELAYTMLDVAVRRCSVNVF
                     EAVVGAIDALYPDGDPNLVPRRPIADDDDGAIEDDIDAYTLPERRGLWRSSFHMSVIA
                     FDRADVARITGRDCARLKGTAYAIAYGAADCIRHHLEINSLNPESAHRLLAEGITLWA
                     EGRYCAEDMGVGPSVSRAMAAIPADVITPEHCKAIALAAIRDDDVALIMWALGARDHE
                     RVSASALFNATGLTATELVCHALSPLGTGRSARQSRSGVGHLAGMARAAAWLCDVLAY
                     TPAAKDMAALARACIEQDRHKEPECCAARVAFALARWPRLLWESGRGVPLVRDAFVSC
                     VTGWGLTPDAMILIDAIEIWCDALGMERKTMRDTLALFCSMIGRGGSFECVRQIGMAL
                     HGSPWTKACSAVCHGTCRGSASDTSQAFCNGRSRSDADTIAAWCMPTFSLERWPV"
     gene            complement(642195..642539)
                     /gene="pino_cds_387"
     CDS             complement(642195..642539)
                     /gene="pino_cds_387"
                     /codon_start=1
                     /product="Hypothetical protein"
                     /translation="MQNRQVGWLRASTTPGQSGWTVLIKACLCVYSGVALVAALVEAL
                     LWSTRRATGLVTACRGIGIWPREPAPPTPGTGDTLWSSRSACLLVGLGTGALWPVWAA
                     AALVILLRRRRQ"
     gene            complement(643100..645333)
                     /gene="pino_cds_388"
     CDS             complement(join(643100..644081,644174..644932,
                     645056..645333))
                     /gene="pino_cds_388"
                     /codon_start=1
                     /product="Hypothetical protein"
                     /translation="MEQPSGAQPPCDEKATAETQPGEQPAHKPTPCVRVSLVAAVDRN
                     DIVCRQGQPPDIYKTSGQMDAVRRLVADHTTVIGYRAALAMDDPPRGRTDLLYVVGDG
                     STLAAFLPYASTVHRFVLRKALYDRNAPGCEVFPRLPSTPYHRWTGLDVEGPSGLWFW
                     QQTEWLRPTITPSGIAVPPLESSAGLRPAVGLSGAAAPTQIDQGDADLARALANSLLS
                     PEIAQDDIILYHLLNQDRIIGEGMTTKANNHTTDNDDDDDWFYADGDPVDTDDEDVDD
                     DGNQEDSDDDSSGDDNVYDNDGNDKDYGDDKDDRDDDVPQDRLEMGLCASADAEFADD
                     DDDDRIASAPQGASPRVPRILANILDLALPQPAHTRHRRSDSDNDGTDSPSDMALRLD
                     PIVLPCSVPASVARVARAWLTLVAIPVRRWPRHPLAPCFDPAVASVSGATAYYCKYLS
                     VAVHATLVGCTRVLADCLHWAIEEDPVADTCLPATALYEEAVGRDSLPLYCVASMVAS
                     DNARRGRLFRQSPTMRPRDPRVPVRILCSAVTAVARAGVEGTLSADSDTTKYERFLVC
                     SVDALLDGVGALVSGEATPPPLIIDALCESCQALIDGVAFIHRHAPNGSAYEKCVVTS
                     LGHIVRAFPQLSGLGPQTPSLVAYAVRSTSGMDSTVTDSLLDALLWYP"
     gene            complement(646130..647863)
                     /gene="pino_cds_389"
     CDS             complement(646130..647863)
                     /gene="pino_cds_389"
                     /codon_start=1
                     /product="Pqq-dependent dehydrogenase"
                     /translation="MARVQWNNRPRCGHGVHLSTMVVIVAALMACAIGTLPASATYGS
                     NHGGHPQKKAWWPNWGGGLDNDHHAAQERDIAACNVGSLMPAWVKAMHGDVASPPAVD
                     KRGVVYIPDLGGSLWAVDGDTGATIWEADVGNFTGNVGIYNPATGRSNGTTIRGTPAL
                     YGDALFFGDAASGHVFAVSTRTGQLLWKTLVEIHPAAVITMSPTAVDGLVLVGVSSIE
                     ESFAGFPNYACCDFRGSLVALNARTGVIVWKTYTTVAGYAGAAIWGSSPSVDLDTRTV
                     YVATGNNYKVPADVQACVDAHEGDASQCPSDPNNLAEAVIALDLDTGAVRWSISFTAQ
                     NGVDVWTVGCKPERVGLPGPPNVNCPAFPGEDADFGQAPLRFYYESGAAKVPLMGVGQ
                     KNGLFFALHPADGSLAWVTVVGHGGDIGGLQWGSAFDGTRIYVASSNSHFANETLHDG
                     RTTRGGSWVALDPATGSVLWQTPVPEGLTVVDEADGVAKWPLAWSSLTVANGVVYAGT
                     GSRSSAVPTMFALDAATGEILWTHVTGAATLNGPAVVEGTVYWGTGYGYMSTPGKAFY
                     AFRPALSVSHC"
     gene            complement(648363..649487)
                     /gene="pino_cds_390"
     CDS             complement(648363..649487)
                     /gene="pino_cds_390"
                     /codon_start=1
                     /product="Morn repeat"
                     /translation="MTARRYRDLATDATVWRALCLGRFGAPLHRGFLEAGKGWRWLYR
                     AQACVGTLTTEVGATTHLGRIYWGDLVGGLPDGYGLALSLPTPHRDGSTARRRRCDDD
                     QSVGAHYEGQWRAGRMCGHGTRTYRDGSRHEGLWEDGFAHGHGWRSTTHWTYTGDWDR
                     GLRHGRGRCVWTAGDAYEGEWQRDREHGHGVYTYADGSRYEGGWARGDENGYGVLDCA
                     VSGRRYACEWVDGDRCGHGTVFYPRGGVYRGQWWANRPHGWGIHVSADGCLYMGQWSD
                     GRMNGACLYAAPPRVLREDASVTARHEGMWIDDESVGYGVCTFADGSRAVGTWSGDRC
                     VQGFVAAHRVACADPPCDAQECMACRIIGETRLSPHEYNT"
     gene            650516..651958
                     /gene="pino_cds_391"
     CDS             650516..651958
                     /gene="pino_cds_391"
                     /codon_start=1
                     /product="Ankyrin repeat"
                     /translation="MEDLPDELLALVFSFLPCVVAGGAPKAVSRTWRAILGDRRAMAR
                     APCVSGVDLRNTTTWCRAAAAAGHLGCLRHAHEGGHVWGAATCAAAAGAGHLHCLVYA
                     RSHGCNWHRDTCLLAAEHGRLDCLRYALDRGCPHDRIGHDLCAKAAAAGRIDVLEFLR
                     GQGFLWSATALVEAAARGHMDVVTFLWSRGCPRSSDSTMRAAASKGHIDCMRFLHANG
                     VQYGWGLDQHAFAGGHIDCMRFLHEHGCSSIGWYQYINAVEAVDLDVFRDVLAGRTHG
                     PWMVGAMCRRAASKGRADVLGLIASLYPDHDHSWDADVCSAAATGGHLDTLRYAHTNG
                     WPLCSGPKTMAIVAAAGHLTTIQYLCDNGCTGDKRACLVAAANGHVDVLRYLHNVGCV
                     WDRHACIEAAAAGGRLEILRYCREEKGCPWHPGLALRAAAGGHIRCLRYLCDTGCPLD
                     AETYAAAVASGRRACLAYLDRRGCPRPANP"
     gene            complement(652470..653942)
                     /gene="pino_cds_392"
     CDS             complement(652470..653942)
                     /gene="pino_cds_392"
                     /codon_start=1
                     /product="F-box domain"
                     /translation="MGEYGHADVVPIECLPSELLHIVFFEHCNVIDRAAIGRTCRLWR
                     AIGRRRRRCRERYERPSWIDIVGALCEGKPGVAMWLVDQADSWHCPLLPPDQRACLRV
                     IKRGRPDLVPWLRQKGARWTPKARAKAIARGYAAALREGSSDGLEATDIDLLSAARSG
                     NVGILEHLRDRVSGNSMAARFLARRALMAAAHAGHVPVLDWLASLGHVHAGNVSAVIA
                     RERGHMHVVEWFSARGIRPMHAAPLLEAIAQGNLVAVEVLAPTDPIKCCTLVAAAARA
                     GHMDILEWLVVDRCAFRFGACEAAIMNLANDRVMEFIVRIDRRLMSADMRAQVRRRVM
                     PAAINRRLADVVMWMVDRGCRWPRQARLVDWAVVQLMDGACTDATSTTLARALSDGWV
                     PPAGSLLRLMRAAAHMRAHSFATEPVDAVAQMLVRSGRCAWTDEARQIAVSACAVDTA
                     LAALEGLDPDGLEPYLRLARSVRPHGDDLADRLAAAALHR"
     gene            complement(654593..656356)
                     /gene="pino_cds_393"
     CDS             complement(654593..656356)
                     /gene="pino_cds_393"
                     /codon_start=1
                     /product="Hypothetical protein"
                     /translation="MDNGFGSCSDHADVLPPELWDRILNGVDRRGRPLLDPRFRCMAR
                     MTCSRWRAVTSATSKADRVRIASTVSRGRDFCGHKPETGKPTDRVVYASAVADIFARS
                     GQAPDLALSTVCRALRPDNPRDHAITLAAMAASGVGDYFQHVLALVAGDAPCVDPTHV
                     GCTGRASCVDGGRLRRAIYIACLHRGCVYEEGQVEALVTELELLMCVGDIIRADRPHS
                     LGAVLVHLGCRCASHDPTSLHNDVRPRETAMGVWDALANWGTLSTTRVVLAIQRGTSF
                     FDVGAVLRRYLVGVWRYGHWVKEVVRCGRREVIEAHDGSAGMHMAEALREAINMDRLS
                     MAQWLAAHHRQHGGTGICGMAPEKVRTLLADCMGGKSTAWWRWLVAYGCDPTDDDARP
                     ILARSWCRDADALDFIDLRTRQSALTDGGKHVVDLLCADPPRLAWSVRERTMRTLAPH
                     MPVGSAPVSNGLWQQAMVQVTERIVKAHPRASDVSASLLWLCRKAHRWGLLDDALALS
                     AICAGVTADDLDHPRALLAHVPSEPEVWSVWVGKVTPLPVPVVAVARTFCGPPTDREV
                     VATISGLLRLLDEAGLVSLLP"
     gene            complement(656755..658193)
                     /gene="pino_cds_394"
     CDS             complement(join(656755..656877,657069..658193))
                     /gene="pino_cds_394"
                     /codon_start=1
                     /product="Ankyrin repeat"
                     /translation="MTIGHLPDEVLAHILKWLPCADRATAASVDRRWRSLCEDAHLLG
                     RPLCVGPFAAQSGSEPFGALAHNRQVCDTYIHARDCPCHDNRLVDAAKHGRHDLVKPL
                     LQGWAPRYNVACLVAAARGDDNVLMALARDRGFHTFPAVEVAEVAARAGHIHVLDHVV
                     DFARYVVPSLCRNAAGAGQLETLVHLRQRGARWDKETFEAAADSGHLGVFQYLYENGC
                     PREDWVVTCRLAAKGHAHLVDYALEHGLEWSSQADYAAALGNHLDVLLVARTRGRPWT
                     AKACSGAAAGGHLDLLKRVRADGCPWSGQTCMDAARNGHLDILTYAIENGCPMGEYAA
                     KAACKKGHLDCLVYAHQHGAPLEDNDCWVAARKGHVDCMEYGSLSAVASAASIVGSAA
                     VFASCIGIVCVGRAVSWLSSRRH"
     gene            659070..660323
                     /gene="pino_cds_395"
     CDS             659070..660323
                     /gene="pino_cds_395"
                     /codon_start=1
                     /product="Ankyrin repeat"
                     /translation="MEALPDELVAHVFAFLPCVVLLGRTSVVCVRWRRIASDAAAIGR
                     RPCVSHWPTPDWLHGPAAAAGHHQCLVRADERGQVPGPDTLAEAVRSGHRQCIAHCAL
                     YAGQDITGAHTAAVESGDVDRFQFVIQCTRSPWHLLDDLPHVAASAGHLDMLAHLHQS
                     GHDMWGVCGAAAAGGHLACLAYGRKHGCRWDTYDYANAAAVGHLGVLCYMEDNGLAPH
                     PDAMALAVSKGHLGVIVHLVERGHPLHDRACDDAAMSGRVDVLEYVRSRGCPWTEATC
                     ASAAAAGHIDMLVHLVKQGCLWDAQTCAAAAAHGHLEVLTYARDSGCPWDARTTHLAA
                     QHGHMACLEYACERGCPIDSGALAGAAKRGKEACFHYAHDLACPLATSHSTFQCYLQH
                     MVSGDDVVLGMPYRRDAGHAFWRPD"
     gene            660902..662482
                     /gene="pino_cds_396"
     CDS             660902..662482
                     /gene="pino_cds_396"
                     /codon_start=1
                     /product="Ankyrin repeat"
                     /translation="MQGNGDGMAQGLRLVDMPPEIIFCILAWISIRDRGACAIASPLL
                     VGEHLATAAARCPSLSLPTLLAAGAPLDIVEKVVTWRGAIIRPVLLKPAVRGGRLDVI
                     DWIHARLTGPIEVHCKSDDESEDEADYREHDHTNKEHPLIIPERKKSRSRESVRERRQ
                     KRRHRGHSTHKRRTGPTIAALYEAAYYGRATVLASLLDRYQVDFGGKHGQRLLYALTI
                     EACTGPASGTDAIALLHSRSARLAMSGPCECSRDAGRAAASAERVDVLAWMRAVGCNA
                     RLNTKWEWPHHNTPLSKAMRGGRVATAQWLIDVMDVAAWPKHDEDLDDAMVAAAKRGH
                     VHILDMFHGLGEQTCPHQAIIEAARHGQLDIVQWVLRDPSPDVDPRKATATICPPLTI
                     GWAAAENGCGRVVRWLAARPDARRFLGVGAARAALIKGHAGVALFLHQVGIARFDQWN
                     ALATAVASRNVNAAEMAARHGAQSDIAALAKATNEGQVDSVVFLCDYYGTADVQAAVD
                     MTAGATSHAPWSRDRTLR"
     gene            663069..664184
                     /gene="pino_cds_397"
     CDS             663069..664184
                     /gene="pino_cds_397"
                     /codon_start=1
                     /product="Hypothetical protein"
                     /translation="MDRPDSESGGDKQPAGAAPSLPHELWSAILDAAGAAPEDRYIQA
                     RVCRLWRSLVLARSQSRTHIPLRIGAEVCRARIARAAIARDDQRLFAWAIHESLAAPL
                     PPTLVHKLWRRIAVHDALGCAVAMRMAGPWPLPCDRTGCQCEADPAPTIGVAPQCRAR
                     VHDSCRRVRLVLAALKHKSLAVASLLLSWHVSHPMRWVKAAAATALAGGHIGVLDLMW
                     ANGVAPYLCASAKCLDALRPSTWAQMAAEANQVASLAWIVDHLQPDDSLLGLCLISAA
                     RRGASDTVLWLCRQQHFDGFAQALVAAAATGHMSTLASMAPHVAAVRAHFEARGLCLD
                     HCVTTTKEYNALATGKRMPLTNLLRGRPLDRQSAQPS"
     gene            complement(664720..666045)
                     /gene="pino_cds_398"
     CDS             complement(664720..666045)
                     /gene="pino_cds_398"
                     /codon_start=1
                     /product="Hypothetical protein"
                     /translation="MTDRASTMPHRLGIMCARLRIWRIVPPRPTPSTAALLTRLMHDA
                     LMLSCHTDAEAWTRVADVYVPFYETLLAIEAVEPGCVPDRHDLPTPASACVLYQQFAA
                     DSPWRHMTEDDRVAAWRSTARLDLGIGTDVMVRTALSLYNLAAFCARAARERQVLVKD
                     DTRLPPTQTDFTDGAFLVVSRPHPQGGRTVALFTGSSEPTWLMRLADNHTCTSLYHGQ
                     APHAFWSDCCDTQAVRRKGTLCGALLGLAARLVALGGLDLIGLRRIATDHDLPAPVAI
                     ALYDDRRGIYQHRDPSGKVRRWTDSADDDPWMPGKGLHYGMLLSWPRLDALLACTPSM
                     LAVMRWFAATRRLDHKAARVLAQTATVTTIHAPMQRLDGETRKLFAAYAVLDSCDEAD
                     PRRLVDIAGAFGIDARAATYRADPALLGADIGVAIVNEYASRSSDFAFL"
     gene            666485..667843
                     /gene="pino_cds_399"
     CDS             666485..667843
                     /gene="pino_cds_399"
                     /codon_start=1
                     /product="Ankyrin repeat"
                     /translation="MDDLPDEILGLILFFVPCAVRLTRTALVCKRWHRVANDHAAVGR
                     APCVDKSLKGKGLCWMAASRGHADCLARARDCGRPWGDAIYHVAAHAGHITVLDYAHV
                     HGCPPDARALEGAIDGGHLDAVRWLCDHGHSWGAVSCARAAPHGDIALLVLLRERGCP
                     WGYNVTRILADRGALDCLRYAHENGCPWASGTVSNLAAKGLLDGLRHAHENGCPWDDD
                     ACDRAAAAGHWACARYAHDNGCAWSHVSNLTAKAAAAGDLATLTALCKCGCPWDKSTT
                     RVAAEAGHPDCLRYAHENGCPWDEVTCTRAIYADSFECLRYALEHGCPWRTSIHDFGH
                     VIAVADLTIIKVVYAHTLSQSRWAAEAARNGRLDVLRFLVKRATILMARSWKRRRPPD
                     TSTVSSTRMRMCRGTPTRTRSPKRLPNTAPSTASNTRTTMALSGTAKWWKRPWKEAIA
                     TA"
     gene            667931..669397
                     /gene="pino_cds_400"
     CDS             join(667931..668018,668238..669397)
                     /gene="pino_cds_400"
                     /codon_start=1
                     /product="Fascin-like domain"
                     /translation="MAGPDACMWTRAAALTKALLPNGAAGKDPLPAVADDRHTAFIAS
                     SGAWVPPPGAYNFSATLWGGGGGAITSYFRCVAGGGSGAAIIDRVLDTSTWPSNAQWQ
                     ITVGQGGGAGSNGQPSTMTVSSSGGSAVLYTMTAYAGAAGGMNCVGGGGGGANGSASG
                     GAGGAGVPSGANGNPPAQGARIGDIKGGSSGGGDGGVGGGGWDTRWSGGIGQDDGTCK
                     SAGGAAGFNGNGADGEIGGEKRQVVSLDAAPNSGAGGGSGLSCDRYLSGYAGGGGSGG
                     CIIAYWLPPPSPSPSPQPLAQLVTLVSPISGKQLTPQDGGGVASLWYGASYKEKWTVN
                     RLSSGKYTFQGFNGKYLGANPGGWALAQATTVGSWEQWDVLINNGNQWTLKSVHGTYM
                     GTTVDGVIYLNDNASLYWTKTTV"
     gene            complement(669840..671315)
                     /gene="pino_cds_401"
     CDS             complement(669840..671315)
                     /gene="pino_cds_401"
                     /codon_start=1
                     /product="Morn repeat"
                     /translation="MDRGVKRGLIDEHPSASWPQDLHRATTNKRPKTCSFDWPDNPFD
                     RLPDELVVAVLVATADADAVVQWALTCARHYRLAMDPVVWRGLCHARFGPHLLHMRFQ
                     DAGKDWRWLYRAQASVVYADSGPAIGACIIDVIARPPETAKKDWVYWGDLVDGLPDGY
                     GVCLRLPSVHCADAPHPTRSAVYSLPMADARGVARSYEGQWKAGRYHGHGVHIDGLGT
                     RYEGGWKEGQRHGYAVARYDDGGVHEGHWQNNARHGRGASRYANGSTYEGEWRSNKYH
                     GRGVRTEPCGTTYDGQFEDGFPCGQGTQRCADGAAYEGEWNGGRHHGRGVCRYADGSS
                     YEGEWRNGARHGRGVYTFVSGNAYDGGWVDGRKQGRGTYRYAAGTVYEGDWHHGRKHG
                     NGVCRYATGGVYTGQWQEGTCSGQGTLVYPSGSRYQGEFRNGRRCGQGTYCWTDGTRH
                     AGTWVDERVCGFGVRVFADGRQIRGAWRDRDGPGRSQPSSE"
     gene            671980..672939
                     /gene="pino_cds_402"
     CDS             671980..672939
                     /gene="pino_cds_402"
                     /codon_start=1
                     /product="Ankyrin repeat"
                     /translation="MGNRLFSATPSFADATGTPSQTIAGSLQDAINHDRTFTPSTSGA
                     TKTINVRIPYMLDNPLITMLATEGVAALKALLVTDSADINLANAQGLTLLHVAAIRDD
                     AALIETLIDAGAHINATDRMGNTPLHIAQFMRMCNAVKALERRGADDQVVNDAGVKPS
                     GMPALLADPAQVTEPYVDFIRRFDARSFGQSIVVSTSRPMSVPLSLYADDLNLLINSG
                     LSSATLAPGGFTLLHTAAFFGRSDMAVAAINAGLSVDARDDKGNTPLHIACALGHGML
                     AQTFVARGADVGIQDARGKTAFELTRATDTYRLYADYSHPRRS"
     gene            complement(673055..674769)
                     /gene="pino_cds_403"
     CDS             complement(join(673055..673723,673840..674769))
                     /gene="pino_cds_403"
                     /codon_start=1
                     /product="Hypothetical protein"
                     /translation="MGHKSHERHRRGLVGAQGAININPKTGIGVQEATIARPRRTSKG
                     STVDSAPPVPLGVVAPITLEVGTVASTVGVACAGRTRCGRRRWTQWRQWAGQRVWATA
                     AWMAPAAGLYWLAVLVPNFVVFALTTEIADRPGGGPYTTKALMSIARAAAMPLGFATT
                     LITPARRRLTAGWWRPVGLVGFALVFSAMPNLFFAIDDVSVRVVSRFVGSFFFSWAFC
                     VYIGYAQGRRASDALMPAATACMLVSSAISRAIAPAIKDHLLGGGGDDNDDNETAYRW
                     MPATVSALAFMPMLVAAVALAASPRATEADAAARRRLCHGPLGRRCPMVALDRRRRAT
                     CLGACLLYAPFVLVGGNRRAFMAVNALGLVAGALMILGGVLGLVDAVPPLPFLVVSGL
                     GYYLGVVPFAGGGVIIERLVAASATPVDAMLLNVVCQLPGYTGALAVLLLVPAADDVK
                     AYFDWTTLAGGGVMLAAYAWTLGAAWFVLPADAHPDRTLTDSCEMTSVAPAHALSASS
                     LAAPLGGATSPTNPYVSSQPKRTS"
     gene            675275..675673
                     /gene="pino_cds_404"
     CDS             675275..675673
                     /gene="pino_cds_404"
                     /codon_start=1
                     /product="Hypothetical protein"
                     /translation="MTSSQRVLVLWAVAVGLFVIVASAEAPPRTGCGARAYADSCVDA
                     CCAWCPATAANVTVIALARLCPATTNNNSDDKQKADTRFLSGPTAPLRRRPRARVTSA
                     AARRADPTPSCVRPRNATLNWSCPSGRYLA"
     gene            675685..676041
                     /gene="pino_cds_405"
     CDS             675685..676041
                     /gene="pino_cds_405"
                     /codon_start=1
                     /product="Hypothetical protein"
                     /translation="MAACYARRRWMRRRAARTQATRQRQKSGRAHSSHHKTLTMVDIP
                     YAQSADGTVVYYASPYAVARDVEYATVLIDEEGGDNGDGGAGCSPCRVRVDAGHYCMW
                     RWPCAFVFIFGRARQT"
     gene            676341..677261
                     /gene="pino_cds_406"
     CDS             676341..677261
                     /gene="pino_cds_406"
                     /codon_start=1
                     /product="Hypothetical protein"
                     /translation="MPHKHHTPRCHPSSSSSSSCSSSSSHRHHDPPVVVVNVERDDDS
                     RRSDDDDFDRQDRRRAFGGNGNGGGGFPRTGGNFTVIPVPGPIGPPGPAGVGIAGPPG
                     PAGPAGPAGPPGLPGTAGPAGPPGTPGTVGPPGPPGVPLAAIGFSSLIVPGTDLAIPV
                     AGTTPVTPFETASRPGLYNTGSFDGTTFTAPVASTYRFSAAVFIPDVVVTVLGATLTL
                     NLLLTPTVGAPVVVRSNSIPTVGGLVGVTLGGITLSVEGTLDLVPGDAVSLTLTNATA
                     LAIALTLGDAGTETATWFDGNATGQPAVAP"
     gene            677728..679007
                     /gene="pino_cds_407"
     CDS             join(677728..678723,678807..679007)
                     /gene="pino_cds_407"
                     /codon_start=1
                     /product="Morn repeat"
                     /translation="MAAIECNGTDDGSLFQRLPDELVLAVLRATRDPRAIVRFGETCR
                     RLHTLAADDGLWKDMCAQSAHGLPPHVHFAAFGKNWQWLYRARLPLSVTKRKQRPSSV
                     GAANRDDGDAYQGDFRRKRRHGYGRAVINTAHGAYVYEGQWAHNKQDGRGAAMWPGGT
                     CHRGGWADGKRHGRGTVTYGDGHIYEAEWVKNKVVGPGVHILPDGERRPCERVDGKWR
                     DTVQHINDKQEGQSLPLAPRYATAVAALFQPGRYTICHDEEYGMRAEQWENQRRTRGF
                     DDTETWSLDSVLAQFAIDLVDALATDMEPAPRPGLADRAQAMQKALAEFAADGVEPAF
                     ARFAVPRLDAFDRARVGYVDGMTDAQWSRCIQAIAESIQHAAAHGGPPPDNFALLREH
                     FFSLWH"
     gene            complement(679477..680725)
                     /gene="pino_cds_408"
     CDS             complement(join(679477..680528,680659..680725))
                     /gene="pino_cds_408"
                     /codon_start=1
                     /product="Endoribonuclease L-PS"
                     /translation="MSRDARKSADGEKKKEYSRRKATRARQQRQGRQEQEEEGPRRRG
                     QEKVQAPRMRRDATAARTAGVGTRRAALLRPVGALRQGGDPVGHLGLRAPRPFGGRRR
                     RQPFGRGPCACPDQAGVSQHPRRLVAAGCRGLEDLTSMSVALVDLAATAEPFFEERLK
                     IMGDHVEYTSSVVGITGFPVPGGLVHVDAVAVVGRGCPLPRGHLDAVAKANSVKAAHG
                     GISTSGCVVAYPKPPHLSMALPGGKPRSALAVRYGKEVTLAGIWAYTERSRLVRGGVR
                     EQARQTFRNVHAALVEAGCRGLEDVVSINASLVDVEDTWEAFVEERDRALAPNTDYTS
                     SIVGITGFPVEGGLVYVNVKAVVGRGCLLGVAANNARA"
     gene            complement(681344..681703)
                     /gene="pino_cds_409"
     CDS             complement(681344..681703)
                     /gene="pino_cds_409"
                     /codon_start=1
                     /product="Hypothetical protein"
                     /translation="MGGGDFHPDFDDRPEALAAMLVAAVKVDTKPLFAPLYDSVYAAV
                     RRSADRRAYADALRAAVAHALANSAEGNGCGGDDDDAASQSRSVRMLADILSPARCAL
                     GLGPEAFATLLTMSPSP"
     gene            682331..683399
                     /gene="pino_cds_410"
     CDS             join(682331..682546,682737..683399)
                     /gene="pino_cds_410"
                     /codon_start=1
                     /product="Hypothetical protein"
                     /translation="MNTKGWYSRRTVVAIRATSDAITVPRSARGQAPDQDDPLGVEHE
                     EAPASGNRSPVESRSVMPPRKRPLSRSAYRNRQIMAPCMSMATAQCGTTLSAAKTPMA
                     VATAMTRRQTMGEATTLKPNSPGTASSATTALATPLSGHTATRDKGAHGVTTAIMMLM
                     MPTTSMKTNPGPNAHTASARTLPHAIDSDLDAQSDDGGDLDAHKDADSDLGTQGDDDD
                     DSDHDLQSDREYEPTAEDWQRSPATAVAVGLRAEARRLERLCRVNEAVWRQTESALEG
                     VRYAIKRLDRMAACGR"
     gene            complement(683763..684407)
                     /gene="pino_cds_411"
     CDS             complement(683763..684407)
                     /gene="pino_cds_411"
                     /codon_start=1
                     /product="Hypothetical protein"
                     /translation="MNIDRDEAFDIRADALTAQICNLLDRDPNARRIWVNAESDDDYD
                     LFEAVAARLFDQCGVLAVHAMQTTPLVFEIGPRARDVWGPAIAQVLDDPAGEALLDTH
                     AIYEQTAQTWRNLLRLQPLSMTPYEERLMVRLAEDMALARGLALEPVDRHRFRVHAPR
                     AVAVYSDRTDWVDDYLDLDEGNDVPIVEDAEGEYVLDVDGGDNGALWSWQSVLV"
     gene            684998..687486
                     /gene="pino_cds_412"
     CDS             join(684998..685762,685858..687486)
                     /gene="pino_cds_412"
                     /codon_start=1
                     /product="F-box domain"
                     /translation="MERKRTNRIFWRLPCRKRQRRQRDARNHRRLPSSVMSGCANNRR
                     NDDEGGGKGNTRVPSVSHMPDELIERVLGLLSGRDLAAAACTCRAVARVADCERLWKA
                     VYRRDVCAAGPPIEHIDHEAHGKSTRWLYGLMAALVGRVRIGPTGRLTARIAGPDGIV
                     THSGEFVVVVSEKTGYAEFVLDGYGAKRQFYGSAYIHEGQFVRGVLGGVGRSALMHKA
                     IDGGGVHMTSRGPFVGGLPHGLMRVEYDTGTVEFAEYARRGAPASGVLRDYIANGCGE
                     PMALIERYNRALPRTDEPTMRCASKHKDGTHIIHWTRRQMPGAAPACVCLCARGVVRA
                     NYKGDVVVADADGLCFLAIGHTHPDPRLAGRRWIARADVKRASHDAASDILDSLSPSG
                     TQTGEEPTEVRVCRFAARLGQVTADTLVPLLEGALLKPTHTFCAAWADCRDRADLHNG
                     NSCNHDDASPFGIAVVVTGDDVKGDDARAATDSEARPYVHCFLAGARVPADECALFAS
                     GRFYRADLLRDWIAFAPCDPETGDSVAPNIAPIAWRPWMQHAPPAILNWAVRDLSTWL
                     TLAGKSVVLFDRAIADLLRAAVVGALGTPMRSGLDVLTAAATRLWKRKQCDSDNNGNS
                     DGDAADDAAGASITRDDNNDPCHSTLIPGFDHITLDHLELRHPQWDPRGPWTFGPPVM
                     PIEDPTMGDHERDPDGPPDRFVQSHDILRVALDGPSVSFVGARLRNVFFFGHAFRGAS
                     FAAASLDRCAFVGCTFDDDCVFARAVLTDCGFYACRNVRDRTVDASVIKAAFHAIVL"
     gene            complement(687977..689670)
                     /gene="pino_cds_413"
     CDS             complement(join(687977..688282,688402..689079,
                     689198..689237,689312..689670))
                     /gene="pino_cds_413"
                     /codon_start=1
                     /product="Carboxylesterase"
                     /translation="MASANVAGCLLLLLAIAATTTFAVDALPRLDAPNASPGCPSGRT
                     AVSTRYGRPQGKRIDGVVQYLGVPFAAPPVGVLRLAPPVDPLPWRGVRNATAIGPVCP
                     QSGSAYPVSEDCLYVTFTQKTDGSRLVERTGVLKWLRANIADFGGNPNSITLSGTSSG
                     AIATQMLLTSPMAYGLFDRAVVISGGVAELGDRASMRNITGRVLAQLGCPDAGSAALD
                     CLRAADASAFMPIQNALPEMSKPRPPIDGVVVVEHPMKRFREGNYQRVPTIISNAINE
                     SNSLQVRSYGFNATRQDYVSFVTSQYGAHRIADPDSLYPIGEWGTWYHAISAIDTDNL
                     YVCTQDNTYQQMDHAGGSVAYRWLFVPVPTMPAGPEHGLGTAMMDLLTGFMHDGTYSP
                     DLVAAPYGTQANYTSLIIDVEPHFALEDGTDVFRREACDWWQSVWVICGGRHCNPSET
                     ATSCPSDCAA"
     gene            690761..692305
                     /gene="pino_cds_414"
     CDS             690761..692305
                     /gene="pino_cds_414"
                     /codon_start=1
                     /product="F-box domain"
                     /translation="MLVSPHHGDKHALPPALDRLPIEVLFEVLSWLPGRALAAVACTC
                     RAVEAVARDSRLWEAAYRRDIAPTGPPAEHADYAAHGKDIRWLYGLMGAAPGRMREGP
                     TGVLTGRLIAADGVTRRSGEFVVVTSDNPEGDAALRLNGYGAIVTKDVGNGDADTLCT
                     VQGKCFAGQFVGRLTIQWLDASAYASGLAATRVYRGDVSRPGDDVAKGEDAVGKIRVL
                     GGRALDGRCVSTVTVPGCVYAGHCDDGVPGVSGAVRLPDGTVREYWSHVPSDRDRDAH
                     WCIERPADKCAPVTRVAYDILVDDREDDGKAATRVTLATHHDVNRRRRRRNRSDFEAT
                     AALGDGLFVTGPCTRVCYAGHVLVWIGSEPLFLAVSHRHADRRIAGLRLLGDNLIIRS
                     LLELVDGTVPSLNHLVALDRANADAVTKSLARASFARECPRYLQGRCLSDESFGVVIN
                     DSTSRPWVRCFLTGRRVEAKRCAFFTTGRLYESRALERWMDATEHRPSDPESGESVVG
                     NALRIM"
     gene            692315..693139
                     /gene="pino_cds_415"
     CDS             692315..693139
                     /gene="pino_cds_415"
                     /codon_start=1
                     /product="Pentapeptide repeat"
                     /translation="MASVPSDVLETTAAHTISSTQISTTFGNKSVADRAANDIVRLRL
                     LHATHATRNLPPRDIALVVAHAISSWESRCSAAANDDERTDASCIESIDDNRDHSDSD
                     HHGGDDNSDEAVRGFDLVSLRHVELHDPTWDLRGDWRGGSVPTSFDDPTLAEHERHLS
                     GSVCADGAPDATVRPDAHLDSHGVLRVALHKPSFVGARLVGVFFVGHRFDEASFVGAV
                     LDRCAFVHCTFHHCAMTAATLLHCGFWNCGRVVGADTKPLTARKVDKIVRTHGGLL"
     gene            693598..694902
                     /gene="pino_cds_416"
     CDS             693598..694902
                     /gene="pino_cds_416"
                     /codon_start=1
                     /product="Hypothetical protein"
                     /translation="MAAAAPTATTPLDALVQEQLEQDRHTQSSRRTGIIVAIVLIAVL
                     AAIGVAVWLYGRRGTNGGGGNNPTNPDDCTPPCGSGTVCIDKQCKANALSCTSDSQCG
                     TCMTCVAGKCTPKASCCGGVTCGAGQTCDAKTNTCVYVKGYCSADHPCGSGFVCDTAK
                     NACIAEPPYGPGTGPHASGCVRGFGNWVATRDPATGDAVWACSCVNGSLYNPGHGCSP
                     LADQTVCTAANLDPSALVPASKVPAFSNYGWGDHPVLINPSTAGDAVPSPLGGGVCPC
                     KSGWGGGTCTTDQSCSGNGRWTGDTTGCACFSCHNSGGRENGATYRWTGRRCDSLADC
                     KIIINGGPASGQHFRKLQRVRGVHAQRVAQCALLPGRRRDPDGRRLCARAPTKDTGPT
                     CRGASRAKSRRTALFLIPMGSPAFFFPPYQKKRNSAPTMSPM"
     gene            695357..697414
                     /gene="pino_cds_417"
     CDS             695357..697414
                     /gene="pino_cds_417"
                     /codon_start=1
                     /product="Ankyrin repeat"
                     /translation="MDCVHRGHKNRTAPIDILPQEIVGAILWMLGGRDLLSCLCASSR
                     FHAWSEAECDRRRYSSLAADTAATCYGPAALDHLAHRRGVVFDSSHLLLAASHGRTAN
                     VDWLLDHTDAADRVAFVNGQLACPHAVIHAAAATAGSAPLVRALIERGFPLFGQVFVD
                     ASKAGHIDVMEAVYKAAPALCTTCGLGKAVRDGHTNAVAFLLDHFCTDDNRPRIFDAI
                     DKVNDLATAALMHDHFCGTSGLPPVDSVTDARTAMTFAYARDGHGWVVCNAPNDAIVA
                     RLFGDARTSATVVTDAGRRQKRNPLAAVPLDVAREMANVLCGPHCEMLDVCSAWFAAG
                     GDLGGARTIYSMGSKHGIRMTRPTSEAEDAFDEPMLFAIAALRPTTTTEQRARWAREA
                     ALIGSVEVIKALLNDDDVDTKCTSRLIGIIVDTASVYGQLDVLRYLGGRGGVDWTQTS
                     LLQPATRGHLDVLVFLHERGARATTHEMDWAASNGHLKIVEFLHWNRTEGCTTSAMDG
                     AAEDGHIEIVEFLYQNRTEGCTPRALWRAIDRGHADVVAFLLDHDRRPPTGHALMAAV
                     DPQSGLDKALVDRVLGLCDGSALCDALCSAIVYRRHDIVDALLRVAGDRQPFDEHVFG
                     AAVHWQSMHVLSEMATRTPQLCNRRAVESMSVQCAYMDHCPMAERARQQIVQMLSMTS
                     SSV"
     gene            697603..697989
                     /gene="pino_cds_418"
     CDS             697603..697989
                     /gene="pino_cds_418"
                     /codon_start=1
                     /product="Hypothetical protein"
                     /translation="MIRASSVVASHVRAGWRSIAADTKNAQPSPPAGAFRQALLEAIN
                     KALCARNLVDHDVDEEDALSAALCAIDAYLATPEARLLCPVVACLSARNFETLARDAV
                     RAHRWRAILTRPRQNTDDANADDETH"
     gene            complement(698192..699174)
                     /gene="pino_cds_419"
     CDS             complement(join(698192..698905,699016..699174))
                     /gene="pino_cds_419"
                     /codon_start=1
                     /product="DNA-methyl transferase"
                     /translation="MSRARKRSRTVAAETDTVPCAEAARVAAAAWSVVEGDCRDKLAD
                     YADGSFDCCVRRVLRDTGTLWLNLGDAFTSGGRATRAPDKKNAGREMAYRPPTPSGLK
                     AKDLIGLPWRVAFALQADGWYLRSDIIWHKPNCQPESVRDRPTRAHEYVFLLSKSATY
                     HYDHEAMREPAVGGSGTATRSRRSVWSINTKPYPGAHFATFPLDLVDVCLAAGAPPSA
                     TVLDPFCGSGTVGVACRRLGHAFVGIDLNPEYVALARARIASALDPPPSSSSSKSEPS
                     SSSPPGSGSPDVSG"
     gene            699931..701445
                     /gene="pino_cds_420"
     CDS             699931..701445
                     /gene="pino_cds_420"
                     /codon_start=1
                     /product="F-box domain"
                     /translation="MKKSRKHSTDHDQASNAQNSDGSDANGKRRAIHKLAKGRHSLID
                     PADPLFGLLPEELVIEILAATGDASSVVNWSVTSRRHRRLADDPLLWRRLYEARFGPP
                     LHTGFVQRGKGWQWLYRARACDGRATGTSVGGISTTMDGTPALYWGDLVDRVPHGYGF
                     LAACGAPTQVTTDCYEGDFYKGQCDGYGIRTWPDGDRYEGDYAEGERHGRGVYSWPDG
                     HRYEGGFADDKRHGHGVYTWLNGHRYKGTYISNKRDGHGVYTWPDGAQYKGAYDGDMR
                     HGYGVLTRPDGRRYEGGYANNEKHGRGIRTWPDGKRYEAYTPMVSCTAAASTHGPTAP
                     GARSDTEMARCAARQCAPIPTVHAPEAIGTTAKMRTTWWWNTAIGVNPRDHVRRAPSP
                     FLVAVPAPDTVIVGLSFFLFRAQKYFFLFFDDNKKCGLLSVLFMQSPVFGARAQIVAW
                     SPVLFVPWKSNGGQFESRQAQTRLCSSPLVARATACRLACGGHPARVDRTQITD"
     gene            complement(701729..704052)
                     /gene="pino_cds_421"
     CDS             complement(join(701729..702770,702884..704052))
                     /gene="pino_cds_421"
                     /codon_start=1
                     /product="Hypothetical protein"
                     /translation="MDRQLDTALSPPNDSALKVLSLALASAARAVDNQDGQDGYRQQY
                     HGRPATTAADASAIRDRFAGFQSVCADWRQEVQHRAAGCDPRGVRLCLAAIDALGADI
                     HGVRTIPADVISSKAMRGVPWNRDEGPHINRTVGFASWDLDPVDDDDDDHHHNNDADD
                     DAGNNNGDQGERQNERDHSTNQPRGTEGQDQDDAADSLCAPALARAQANDTSTPVLRE
                     SGCQPRSPSPPYHLLLSCSRPVASCNGDNSGGDNDGDAFRRPRVGDAATTTMTATHFV
                     FEVRIGFCPDVWATVGPMVNAETGACVDLGCLLVARSKPSAWDYGGVPWVERRRLLDF
                     LASGQSTWAAFAARRYDDLRRLPATLATRGWLVRDVRGERWTWNPDKGPMSELDSRRA
                     GNTPAGPCDSMFYRDTPGFPLDPCRDARARGHWAVHAEADIGLQRSRLVGMGLASPCA
                     VHGRTQGFASRQCDQQQQESTNPFLFSNIASRALPPVGADAMADLIDAYVTQALFGEY
                     GPIDAVTGQRLNGGLCDHLLDAAVASGRLWSGLRFIRGLDTCAYECPIDCDLAAVDGA
                     GDGPRMLVNWRARCKPVCAVAALSQGTARRRRSLAKHRIRVWMAVAVQTDGRGGACVA
                     LVVPAGCKALLDERVDDPTADDDITWRTVAARCVKGAPDPPADLHPALLASLEVERAC
                     RRTACVVPWAYHTGADGPVDARLLTDWALDRVAHLLKAVDDAVPCARVEAINTK"
     gene            704459..706699
                     /gene="pino_cds_422"
     CDS             704459..706699
                     /gene="pino_cds_422"
                     /codon_start=1
                     /product="Hypothetical protein"
                     /translation="MLCDNAPPLIDQPVSALAVRLAAEIIARTPGIQAPPAQMHPDGD
                     GDNITTFASPSPSVTKAECDESSLYATDATVIVATDNANADVDTNVDNTQKDDDDDDN
                     DARALLDRCMETEAKPPSAWQSFLKDYAATLPTRGPALRDIGTTEAPVYSTGTALAVV
                     QRIIPVLDAHHLDVYAIRLRPEHATVIASPWEHVKPMAEIDCGTGDWLIEVACGRAWF
                     DDVREGQELAGGVFSIRRAIHRHTGTVIETNGAQLGDGYRVATDALFAFFRSGCTAPQ
                     DFVDARYGRIDRAPAALASLGWTLDASQSAHNLDHRWSIASAGDERLGVFVRAGDGAR
                     VHVVSYRGSVFVGVQPTSICLPTAAYPPRLERDGGDYPFAVPRPPADYTGDTHSDDGD
                     DRVDAKEADHVHGDRDHLIAQGLLDRMHMASGWPEDHIGATGVRWPYTRFEFDPDVHM
                     DATAEADWHRRLAARMHLVADLIRGDYGPPVDDDTLAAAAESASGLTTHMPMICQWAR
                     GPHWVNARTCDALMEHLTSIGAARSWRIWRKDLHMRHGTRHHDPARGLYINWFVQCDF
                     VQCRSDFAPATVRFYAHLVGLDSGATAHSSSSSLSTPTLSTSAPSPAHAVAAYYCLTA
                     RKHMPNCDDDDDHQGWRLAWAAAEDLGEPALTAAVDVAIDAAQPALTSRFTADQPHPL
                     VAYYRDREFALAPGLFRGILDEDALCTDTGVSARSAGAAVICALDWITTAFDRHATLF
                     ALPADL"
     gene            complement(707134..708951)
                     /gene="pino_cds_423"
     CDS             complement(707134..708951)
                     /gene="pino_cds_423"
                     /codon_start=1
                     /product="Hypothetical protein"
                     /translation="METAPEDILPPELWTLVLVGDNDGDIDEYGGVEPKWRFAVRTVC
                     HLWREIVDRAAPSFYGGPPCTRACLWKPACQILWGRGRLVCASTFVQWAKMHCDSATW
                     DTVAVDHLIAWINKTSSVPSHDIKVVLAASGVPTLVDYVLTSSHADTGRQSDAQRSCE
                     STNIDNESAVPHDTPWPRMSLNQAAEHHPFVVAAAAVRSGCHEAIARIAKVMPYALMS
                     PLAIEAAAANDDPAALEMLLTIGKVSHIEAVCAMEQASGPNIATRCLEAAKDPHGRPW
                     ATDLAAFLASEANRNALVCQCIKRAGCTRPDAYNVNNAVPAILDVYVAHGMTALMDTD
                     GVIDLICKRRSIGGARWILRRAERSDNPQERSHILGRLAKGACRLDDNGDRMQCSDDS
                     DALLSWLCDGPPKYNPLRKGDFSQLDSLFLAACVGGRADPGCFAWLCERWPDEVARMN
                     PSWVASMMRVACDSADDSHDIGTVERLVAALDAVAAPSSALIDAVDVWSLLFERLERV
                     AANADPDAYTAAADLIQYAWSRCKTTGMHMWHSALPLSARWYSLRLTSMPAGRGDVRG
                     PMPPHGAVGAACARCASPDPSICIPRPQPFWDGSPSTVF"
     gene            complement(709584..710213)
                     /gene="pino_cds_424"
     CDS             complement(709584..710213)
                     /gene="pino_cds_424"
                     /codon_start=1
                     /product="Hypothetical protein"
                     /translation="MGARMTAGLPAYDALGDDHETLANLADDILEARGGSAAQRPGAD
                     AVDTLRGLYRRPQVHFCCTTAAMRRIETLALRLPIDHVCAATGAASTDPDAMSWWVAM
                     SPCSHQLLLASQVVVEVDRNRRHVPASTPSPSPWTNQTVCSSSSSVDTVVVQEDTSAM
                     QAITTAIATPSATTHKGAEAQPKRRRRRRQRGRSKSALGGRLAVPPLSV"
     gene            complement(711097..711492)
                     /gene="pino_cds_425"
     CDS             complement(711097..711492)
                     /gene="pino_cds_425"
                     /codon_start=1
                     /product="Hypothetical protein"
                     /translation="MSNTDDATGILQGEMLTDRNKREHYVLCFRFRYVTADGVDVQFS
                     IDPTPAEPIEWSALVDAIKTDQQFGISMCSEADVSVDYADGQVKIYIQSTGCDGNLDM
                     TLPAAKCVAAFEKCAMAYAAHNPSLDPSS"
     gene            complement(711862..712266)
                     /gene="pino_cds_426"
     CDS             complement(711862..712266)
                     /gene="pino_cds_426"
                     /codon_start=1
                     /product="Hypothetical protein"
                     /translation="MSNTDKTVGTLKGKMMTDEDGKEHYIVGFRFEYTSTDGISVRFY
                     IDPTTTDAGKWSTLVDAIKTNQPYEVSTCIMNGDVSMGHASGQVEMRAHRTGAGGDGD
                     LQVTLPAAKCLEAIEKCAAAYAAHDYSLGASS"
     gene            complement(712701..713138)
                     /gene="pino_cds_427"
     CDS             complement(712701..713138)
                     /gene="pino_cds_427"
                     /codon_start=1
                     /product="Hypothetical protein"
                     /translation="MADPSDNSAPARGLLRGNAVYDDYDGTRAIERFDFCYETEDLTV
                     PISISLDTTTAAQWRAFADNMRRGVDCSILACDCRGSVGIDHRGGKVEFYAEKGGGDG
                     DGSISAIFAAPKCIDAIEACAAAYEAHTAEARARVPDDFVSIG"
     gene            713525..714562
                     /gene="pino_cds_428"
     CDS             713525..714562
                     /gene="pino_cds_428"
                     /codon_start=1
                     /product="F-box domain"
                     /translation="MTAIDDLPDELLVHILRMGFDGDGDDDEDPDQRDVFLAAVRLAA
                     RRWRDVATVFWRPVAPHAYGRCLQFLKDTGDDAWLAKPRTRRLTKRRIVAKAVADIPY
                     AQAEAGPALDSVVLRPYDYATALVEAFGTNAPKVAHRECLGERCPACDQRPDQCVCLC
                     GDECWPCKGRQPLSQCRMVLGDVWDGDYPGDWDGDSDNGGRLASEYPRLDKAIERHRA
                     GSMSCRSRMRHPTLAHWSATRTSPTDAAVYIARCAQTLVSHALADAVVIGRMSCSAVI
                     RRAHRLNDYRVCARDPLWDIGGCDCGQWTFGEKRCVCDNVKYYYGADDVEIDGAYSLD
                     LTARHGSLRRG"
     gene            complement(714771..716168)
                     /gene="pino_cds_429"
     CDS             complement(714771..716168)
                     /gene="pino_cds_429"
                     /codon_start=1
                     /product="Hypothetical protein"
                     /translation="MVRSRQPLAATGSDEITRGHLTAAVHSAAEHGRLEALKYLTANN
                     TPLGRRGQRAVNSWLLAIAARAGHVPVVAHLHRLLAPDDSVPCRCKRRVGDAAWKAPT
                     TDVALWLRDNRCKGYVQPDAYVIAHAVASGRTADVTRMLAARAGLRALFGARTTASLA
                     TRPCTEMPAIEEGVADAARKGDMAMMDLAVRLLCRHSAPILIGAARGGQTDLLAWATA
                     ADGLCVTGLGAPTATMMRAAATAAAMCNQPASLQWIAQHFPDAITPTLVWTAATSDAP
                     DAVRALAALVPASLIAWDSVLSDAMASDSLAVVRLLVEERGVALTPLVVTSSDSTHDG
                     VVDYVCQRLTRDQLQVIVDMVCARASPLRHTIVTHLRDRVPDLCMAVANAVSANMPGH
                     WLSDADACSCTRCTVSPKTSSSMTAALPLPHGLSTTVAPTEDLPLSLPPPTKRQRLID
                     PQRSLADGYITRPIL"
     gene            complement(716259..716621)
                     /gene="pino_cds_430"
     CDS             complement(716259..716621)
                     /gene="pino_cds_430"
                     /codon_start=1
                     /product="Hypothetical protein"
                     /translation="MASPLFWHISVERFAARWGAGRLRCLIEAGAPASVVRAAIACGQ
                     QAPLPDLIQSAVLYGDVRVLDVLCRALQARLLLLPHCRCPTVSVSFCRLCILHPFCCR
                     PFLAAAQLPSSQLYKHYD"
     gene            717181..718299
                     /gene="pino_cds_431"
     CDS             717181..718299
                     /gene="pino_cds_431"
                     /codon_start=1
                     /product="F-box domain"
                     /translation="MASMGDHADGDGTADTPRQRRNDTRPAKRRRYKSEQKAHIPVGR
                     TTDDGDGDNPVTIDCLPNEIIAHILFLGSTDLHAQGAFVAATRSVSRHWHQVALVLWP
                     RVGEHAYGRCLQLLKDVDPSTVRECYTKWQVVAAARRLAHQRPMEAGPDLVGVELRPY
                     DYATAMDDAFYQSAGMDASQICAERCPGCRRAPPSCTCMCSVDCRQCDGGPLTNCDAR
                     FDQWLGDHIGGPRGEPECPHLASHYVDSDEDCARRRRDASHSPCARWRGRELSHWCAA
                     ADATKAKIYVGRCAQTLVTHALADADDIRRMSCAAVIRRAHRLHSTKTRLAERPPTAT
                     CACVRRQVAIFRGQGYTCDGRCISTSAEMATTLDLDHA"
     gene            complement(718380..720237)
                     /gene="pino_cds_432"
     CDS             complement(join(718380..720046,720234..720237))
                     /gene="pino_cds_432"
                     /codon_start=1
                     /product="Hypothetical protein"
                     /translation="MATQETGRKDDEERRHRPGSLGTALARRWSHWWRGSTQQKGTDA
                     ERTAHPAEAPTVTTPDAPTLGTTVIDDHDHISHIDHNDHNDHGGNDMDVAHDGGAGDL
                     MTAMLPIELLVLIYGRVHREELEAAAAARWSGRWDRVLGGDAEDDHERGGAPGLYRML
                     TLSRAHHDAVASALADWRSWPDSCYGPGAAMWALGVACTNGRADRGASFSNDNINNEI
                     KSRVGTYRQIHATPTLHPVLADRRLHGLWFGAGMHLPPCWLGAPARRLLDVATDLARH
                     AAPADEPMRTRWRDALADLLATPAWSTPCLVLCVQVPHGCNFALDRAHLFPTLTLTRT
                     YLCAPGGSAGARFTVGGIVDAVADFYAGSPLQPFEMERVRAHARSVWRAYERHFGRPW
                     DPHDFGPGGGGRPLTADTDRGSYEGGYVIDTAAFEHAPRVMYGRDPYRGGWGPRNDAH
                     ERRFASWFSDRWLGEGDLPPAERRLTRQFADPERYGRPRLIDLATPPTYDDPPPRLPS
                     GALAPGGVGACASCWWALAACPKTLDFFKKIRDGGEKKRVCVSCLHFF"
     gene            721117..721737
                     /gene="pino_cds_433"
     CDS             721117..721737
                     /gene="pino_cds_433"
                     /codon_start=1
                     /product="Hypothetical protein"
                     /translation="MGGRTRRRWRFWRRALGVALCWRHDGYEPCESGGDDGSHIGSRS
                     DTSTSPRSAVGSIVGSTISSGAGSAVIIDSDVGNDGRGRLQDRHDSEQTPDDRVCWPS
                     AILGRLAAMEQPIVVRCPDSGDVLYRVDLGEETLECLRAGRRRSWSLTFFMDHEHTDD
                     RRVTLYGLMAPGCEAWTMAFVSDDSGGACKAHVRIEPDHAMHTLDE"
     gene            722229..725222
                     /gene="pino_cds_434"
     CDS             join(722229..723987,724681..725222)
                     /gene="pino_cds_434"
                     /codon_start=1
                     /product="H2b histone"
                     /translation="MDGPTYDQDQDQDQDLHGDAEYDDQGGDDNNDYDHASWQGNNDG
                     GLQANEAVYEMPADSGAACAVEDASAADMLADCTDQATAVRLFCTRWSRLRAQLDPLT
                     AATRGIRLEQAALRRDLAAYMEGARTRRAVIQCRGGDGDDNATIVVRVDPMRPTTSMK
                     REVITAAVYEHVTPDLVRACAEAAAAAAEKRSAKVAKAKAVAARKAARAAAAAARPAK
                     RRRRTAKHDDDDDGDDDKNNEAKNDAGSANTPMVDNNPVATDDNTVNAGDVGAIDDAD
                     GTGDAHLGNTPALAEIMGAAVVEATRAAQRRATAQQTSLTVGRYDPDRDDESAMVDMW
                     DASPSSTKGACEVSCPDARDVQVDGKSQEPVCALAAYAWSTEDVPVEVRAWATRFVEL
                     ADVASGLRDRMRPIEVAIEALTLDLPPPPSEPAPRRTPAKGTARNRPSARAIEAAAKR
                     ERHEAFGPMRDAVARHLAEVGADKRGVPVRFPTSDALYRLRESVRTRAGTLTRTDYVP
                     LAADAAAAAMAQVEIDPAVPIRPRPPPISWRMMNSATGSLMPSPAGSTNTASAAPYAR
                     APSRSSAWRLAATLSSRRPMRSRRGSHRWRGRCDRDRNRSRNRRGAQARAGRQKDQKE
                     GRQAPKEDRPLNQDRGDAGAVGKRGRGQRKKNYASYSSFIYKVLKQVHPRTVGPSSNK
                     SMSIMNSFVNDMIDRIGTEAGRLARSNKRNTIGTREIQTAVRLIMRGELARHAVSEGT
                     KAVTKYNEAVNAAAEANAAAIDTTAA"
     gene            complement(725462..725815)
                     /gene="pino_cds_435"
     CDS             complement(725462..725815)
                     /gene="pino_cds_435"
                     /codon_start=1
                     /product="Hypothetical protein"
                     /translation="MADRDRRTLLGRLLAGETDHLKLVHQEDRQRVWRLVLDPLELRG
                     RINHSITATAILDRRDKFIVRGTLDNGWMDHATVDAAEEEGSHTLQAKLEFAAPDKDY
                     CAPVPFCAERKEKGK"
     gene            complement(726227..727075)
                     /gene="pino_cds_436"
     CDS             complement(726227..727075)
                     /gene="pino_cds_436"
                     /codon_start=1
                     /product="F-box domain"
                     /translation="MNEIRLPAEMWCAILGHLDGPWAAVAAHVCRAWRSWTPLRLRAL
                     DAVAAQRPLHAIQMVDDNDVLDWAIAASRSVAAVACVVDAASGRAIDPQVRLDDALVA
                     CALIGTPAMVLNALPVLLRGAVPTPGPGAPAAVNAVLAALCNERLLVVAAQKGNVTFI
                     ASMARCVVALFPRLCGDAGVPCPRTFLATMARDAGTRALMAALGADDICAFRTLMEAG
                     CQIEPHVLAAALADRAEDDKSAWAKAGPHCPHCGGNALAVTVTPTRLSMTVCCITCMR
                     PWRVGF"
     gene            complement(727206..729385)
                     /gene="pino_cds_437"
     CDS             complement(join(727206..727272,727725..728277,
                     728469..728840,728992..729385))
                     /gene="pino_cds_437"
                     /codon_start=1
                     /product="Hypothetical protein"
                     /translation="MTDAYDDDHGNAERPLRGLGDMPIEVVQMITRHLDTPHDLGAFA
                     IAAPHLVADPVTPQVAKMIGFARASDVLVRGAPLSAVIALFAKWDEPVAVHMLGDAAE
                     GGRLDVVQWIYDCIADGLRDVKDCDAPPRDLFATRYVPRFSDHCGSTPTDDLLWSDAL
                     ARAARKQHLNVIKWIVKGPCYGDVSTWRIKTIYHSVMIEAAARGHLDVLKTLHKTVAQ
                     QTRHGSSRCQCPREIGIAAVQADRPDIVDWLLDQNCAGGARSGAGRSLAVLQWAAGDD
                     PSAPRAGAAWRPTDVAMLAATNRRVGVISWMRTRQDGQRALTVGVARAALAAGCIDAA
                     AHIRPFGTWDALEAAVVSGNVDTVRAVADAGGVCSPAAFVGAIRHGGSDVLAFLCERY
                     GVAFVEGALASLAGVACAAPCLDWLGATRPRLTCVWPRPMRPTWAAASPRHGSTSRAA
                     AKVPVQAPQIL"
     gene            complement(729670..730032)
                     /gene="pino_cds_438"
     CDS             complement(729670..730032)
                     /gene="pino_cds_438"
                     /codon_start=1
                     /product="Hypothetical protein"
                     /translation="MDKVGSALQGATTMITDGVSNDVRYAWTNDLRSATMAMMMMTAT
                     RCRLWSPPGLRARGRAWTPWVPMCMRTPGRGHGERRLSARPTDSCGTHRPSIPICSPT
                     VFLRELSSNKKTSRDKKD"
     gene            complement(730426..732069)
                     /gene="pino_cds_439"
     CDS             complement(730426..732069)
                     /gene="pino_cds_439"
                     /codon_start=1
                     /product="Ankyrin repeat"
                     /translation="MATAAVEADGSAMIDHLPDEILVCCFRFLVCVDRRTRAAAVCRR
                     WRHVAQDATSMGRVDCTMRAAVLRRRRGVARRLLAAAKAAAVEGHCDCTRYILGGRQR
                     LRDPLHRITCAAARAGDVANFVYVCSQGYGQYHRALEEAARHGQTAIVAHVIETAAAI
                     GNAFYPQTIEKALVHAAYGGHLDCATYLSSRSSIKSRGACSHAALGGHTDILRVLIAD
                     GHPPHNRACMQAATGGRLDTLRFLHEAGCPWDARTCAAAVAAGRIDILDYARDHGCPW
                     DTGTCAAAARTGRLDVLIDLRDRGCPWGDDTLVGAASIGRLDILTYLCDNGCPQSVYA
                     CDAAARGGFIDALVYLYSRGCPWNGSVCAHAAANGDLAMLTFAHEHGCLWSASTCTEP
                     AKALCRIRSMIYGRDRVPTADKDEGRLACLLYARAHGCPANTMPCYYAASVGNLALLR
                     RLRDVGCPWTDDVTHAAASGGHNAIVIYAHENGCPWSPYAIMAAAERGAYNILRYALA
                     HDCPYDATEATRAARRGGHWRCVRLLEWAALPSATDLFD"
     gene            complement(732663..733631)
                     /gene="pino_cds_440"
     CDS             complement(732663..733631)
                     /gene="pino_cds_440"
                     /codon_start=1
                     /product="Morn repeat"
                     /translation="MAPLGDPMPPSDAFLVLPDELIVAIVVATRSASAICRLEATCHR
                     MARICGDDSLWRQLYTVCYGEPLHRQFANYGKDWRWLYRAVASAPTAPAATGPHRSVR
                     ESVVFMGDLVNGEPHGYGLAVEMADEAAACKTEQILNRHGVLSTFEGAWAGGREHGHG
                     VEEIAGQGVYTGTWAAGARDGHGACAWAGGENYIGEWRAGKKHGAGTYTWSNSDRYQG
                     MWQDDLEDGFGQMIYANGVRFVGQFRKGQRKGPGVQTECDGRMTVGRWRGLGRGGGAH
                     IDTDGTFYQCEWADGRLTGDVTRIKPDGAITNNPLAVDQDDAQPLP"
     gene            complement(734280..734879)
                     /gene="pino_cds_441"
     CDS             complement(734280..734879)
                     /gene="pino_cds_441"
                     /codon_start=1
                     /product="Vltf3-like transcription factor"
                     /translation="MDEITPLTVRRCLDVPALKDHCDFAVYVWCCVTGNEAPQLGSAK
                     ERHCWQMFEHIQASYARVKVRLASTPIDPHLSGDYVPYKFCQLLGRHECLDHLSLPRS
                     RLQLAKFEAIWKGICEDLEWPYLTASARPCMPAPMRPACCRHSLPMPIRSTRDRAMPR
                     STSPRARLPACVLLTTRACLCGRAMKHHHRRRLSLARRK"
     gene            735494..735946
                     /gene="pino_cds_442"
     CDS             join(735494..735765,735922..735946)
                     /gene="pino_cds_442"
                     /codon_start=1
                     /product="Hypothetical protein"
                     /translation="MRAINLILARKAERLACALHDDRKRFAHAITDREYPEVYAFLRY
                     RLPSLLRGKVSRIDLCLNRATCRRFLHDLAGRFGVLHETVNRYPRWRMLCAVGT"
     gene            complement(736079..737503)
                     /gene="pino_cds_443"
     CDS             complement(736079..737503)
                     /gene="pino_cds_443"
                     /codon_start=1
                     /product="Morn repeat"
                     /translation="MKRARIAVARGDPLSGDNAGQPQKRRRTDADYASAPDSLFERLP
                     DELILHVLMAMDNIAALAAWSLTSRRHHALAMDQSLWRHLCLAHFGPPLHEPPWPEWV
                     DWRWIYRAQSHPASPKGADVGAVQIKCGRHNYWGDVVNGKPHGFGIAVNAHGAHCCGV
                     LRARPSSAAARRARPLIVKRQGHWIEGKMHGDCTKTYPNGTRFEGRWQRGKRPLIGTL
                     RSVSGDRYDGECKSYRAHGHGTFAYAAGGRREGQWRHGHACGEGTDIYDNGDVFTGEW
                     TDDEPYRGTYIWSDGSRYDGEFNKRGQSHGQGSMIDTVGDRYEGQWHEDRQQGHGIMV
                     HSNGDRYEGDWRDGERHGNGVMVYADGDRYEGQWHDALFHGRGTMAYANGDRYEGEWN
                     GDHRHGHGIYTWPSGDEYRGTYDKGRRRGQGTHTFADGSRLSGVWADTVCIEGKVEIH
                     RLTPPCLPGEPCLACAARSAPKSA"
     gene            complement(737989..739449)
                     /gene="pino_cds_444"
     CDS             complement(737989..739449)
                     /gene="pino_cds_444"
                     /codon_start=1
                     /product="Morn repeat"
                     /translation="MSDSLFGRLPDELILHILMLTDDVGAIAAWSLTSRHHYDLAADQ
                     SLWRHLSSTHFGPPLHEPPWPEGVDWRWIYRTQSHPARPEGADVGAVWVKCGRRVYWG
                     DVVDGNPHGFGILLDAHRIRRCGVLRARVDPAGALHAPTTSGSRRQGHWIHGKMHGDG
                     IKMYSDGTTFQGRWENGHRSLVGTLRFPGKGYYDGECKSHRPHGHGTYVYASGARREG
                     QWDYGRACGEGAETYDNGDVFVGKWTGGGPYDGTYIWSNGQRYDGEFNSKSQSHGRGS
                     MVYANGRHYEGQWLKDHRHGHGIMTYPDGTRYEGQWREGLWHGHGAVAYADGSRYEGQ
                     WVNDKRHGPGVMVYADGRRYEGQWSDGERHGNGVMIYADGDRYEGQWRNALFHGRGTL
                     AYANGDHYEGEWCDDKKHGYGVYTWPSGKEYRGTYDKGQRRGQGTQTFADGSRLSGVW
                     EDTVCAEPKVDAHRTGRPCLAGDLCAACLFVRQTST"
     gene            739841..740905
                     /gene="pino_cds_445"
     CDS             739841..740905
                     /gene="pino_cds_445"
                     /codon_start=1
                     /product="Hypothetical protein"
                     /translation="MEKACATARSECNMDGAAIVSMQNATSRILRRTCIPSTTNKGRK
                     HGQARWRRKRRGRAATNITNDSVTDRQCPCLLDLPDEILATIMRWTLPRDPKAVGEAV
                     ASLQGCCTRFDALCRMTCLEVTDMDLRFAPFACCDTDEYDPAIASSSGRLASAVGLVR
                     APRLRRQFMQTCVRYAIYSLIASKPDAMAPRLDLVPFSSFVRPESAAHLMARALAPVC
                     GIFVYNYIKVYTHSDGHIDVRACCGTTAKILGPEPTVLDGWARDTINGALVAAATRAM
                     NATDPLCKHKIVVDSIDLFEAYPDCAGHFCATRPGFTCPVGQNRNLTMKCTLDISSAV
                     YPARREATCDPVSLSHRPPA"
     gene            741784..742530
                     /gene="pino_cds_446"
     CDS             741784..742530
                     /gene="pino_cds_446"
                     /codon_start=1
                     /product="Hypothetical protein"
                     /translation="MSHTAFQTDDIDPRLASFACCDTDEYDPTAISGDGCLISAAGLA
                     RAPRLRRQFVQTCVRYAIYSLIVTKPGTGVSNRGLDLVSFSFFARQRPPFPLLIHALT
                     RGAYAPDHIGVSEYDDGHTKVHIDDRIVARVHGAKPVDLDDHTRSVINRALAAAAICA
                     LDSAQAPPHERAIVADSVDLFEAYPDMVGRLRSTRSGFSCQAGHTRYLTRHATLDIST
                     IVYPKHWDAIRHRARYSPKPLTDFSFLSDE"
     gene            complement(742938..744581)
                     /gene="pino_cds_447"
     CDS             complement(742938..744581)
                     /gene="pino_cds_447"
                     /codon_start=1
                     /product="Ankyrin repeat"
                     /translation="MNDVLPVEILQTILHWVGRCPQLGRVCRLWRDIAQDEAFCRARR
                     SSTETVQGINYAVHLARHGLVDLLAWAHGKGCPVDDRVMAAAVYKNQAAVICWGLGVP
                     AMVGPRAVSTAARLGRLDLLQQLYIRGCPLDRDACRQAAFGGHRDVIVWLRQYDCLWD
                     ALACSHAAKGAQWDLFDWLIGQGCPYDHRAMAAIARHGDLARLVALRARGCAWDAAVC
                     EAAARGGHLKVLQWLRANGCPWGRRSPRAAARGGHLAVLKWLRRQGCPWHPRLGGRAH
                     RSGDRDTIKWVIKHGWSTGWLNRAVLALDFDLIEWSIPVVWPMARRHYGNGPWRIAEM
                     AALCGKQEVIEWFRGRGYEWSHNVMGSAARNGHLGLVKWMHANGCPWGDETCNSAAAG
                     GHFETVQWLIANGCPHDRSQMCGRAAQVDQFNVIRWLAPSAQEATPYVCQMAAYHGRL
                     DVLQWCRTVGLPWDKNTTKEAAQKGHNDVLDWLIENGCPGHYEVILSFIRRKCYDKAR
                     WAYSMGCPLWRQVCLREAQRYGADASFLAWIEEQPDWAP"
     gene            complement(744984..746036)
                     /gene="pino_cds_448"
     CDS             complement(744984..746036)
                     /gene="pino_cds_448"
                     /codon_start=1
                     /product="Morn repeat"
                     /translation="MWHNGRMCDRGVYTASDGRRYDGQWHDGQRNGTGTYTQPDGFKY
                     EGHWQDDKRAGHGTATWPDGAHYVGLWADDVRNGHGVCTYPNGGRYEGDWAHDKSNGR
                     GVYTWPNGERHEGNWKKGKCNGWGVRIYPNGNRYEGDWKDGARGGWAVYTWANGERYE
                     GQFRDGKRSGYGVCLYVSGARYMGHWEDDRFDGHGVHTWPDGGCYNGEFHNGKRNGHG
                     ICVYADRGRHEGRWERDVPHGQGMTIFGNGDRYDGNHVDGKFSGCGVYTWADGRRHDG
                     QWQHGKADGTGTRHYPDGSCARGQWLDGSLVSGEVVCHRVGGSAPCCNGPRCAACMTV
                     MVAQLRRDLLQSTQEQ"
     gene            complement(746170..746820)
                     /gene="pino_cds_449"
     CDS             complement(746170..746820)
                     /gene="pino_cds_449"
                     /codon_start=1
                     /product="Morn repeat"
                     /translation="MDQHIPGPLHFLRLTKYSFFFQFFVSGTCAFQPKKKDANHSLFI
                     VSQDLRLSMKRPVVVCSPITDGLCKRTSMKKRQRDVHKSADLEGHTLDNLRALKKHRS
                     QRRGGVHNVEASSFDRLTDELVLAILAALNNPRSLILWGQTSRRHRGLAGDPCLWRQL
                     CESRFGPLLHRQFLELGKDWRWLYRAQAHVARMVGATSVQFLCAPASTTMCIGVIA"
     gene            complement(747097..747351)
                     /gene="pino_cds_450"
     CDS             complement(747097..747351)
                     /gene="pino_cds_450"
                     /codon_start=1
                     /product="Hypothetical protein"
                     /translation="MPTGRVCEASGATRASSRVRSSATVAAAPLCVPVIRSALRAPLS
                     PRHKHHHTTKKLSLRRTPRRSAARLSKACRVVSLFIFFKI"
     gene            complement(747356..748639)
                     /gene="pino_cds_451"
     CDS             complement(747356..748639)
                     /gene="pino_cds_451"
                     /codon_start=1
                     /product="Morn repeat"
                     /translation="MKRHRDSHKSIDPAGSTPDNLRAPKRHRQEDAYGIEMPFFDGLP
                     DELVLAILAALDDPRSLAMWAQTSRRHYGLVNDPFVWRRLCESHFGPLLHRNFAKWGK
                     SWRWLYRAQARVAATVGTDVGALMVKVCGNKCVYWGECHDGLPHGYGLALLLPTPHCD
                     RWCTLVRAWTNPAASETINNIGYEGDWVDGRMCGHGINTAGDGSLHNGQWNNDKRNGP
                     GVYVRADGFECDGEWANDKHVGHGTATWPDGTYYVGLWADDVRNGRGMCTFSTGCHYD
                     GEWTNDHFNGRGTYTWPDGEHYNGDWKYDKRDGQGTSVYANGDRYEGGWKDDERNGHG
                     VYTWPCGMRYEGQHKDDERNGHGVSTYPNGSRYEGNWRDGLFHGEGLLVKRDGSRYQG
                     DWAEDGRHGHGLCVKADKSRYDGSWRRNKRHGAGI"
     gene            complement(749164..750771)
                     /gene="pino_cds_452"
     CDS             complement(749164..750771)
                     /gene="pino_cds_452"
                     /codon_start=1
                     /product="Morn repeat"
                     /translation="MKRRRDICEGTDLQGHTATHPCVSKKRRHNRQEGSRAFATPFFG
                     WLPDELVLDILAALDSPRSLISWGQTSRRHHELADDPLLWNRLCVSRFGPLLHHQFLA
                     SGKSWRWLYRAQAHAAASTGVDVGALVVTIGHHIHVYWGDCVNGVPHGYGLALLLPTP
                     HCHRERGLARVWTDPSGPTATKADAGYEGQWQRGRMWGHGVQTQPDGSRHDGLWVDGR
                     RNGAGMYTRADGFKCDGTWTDNKRVGLGTATWPCGAHYVGQWAEDMRNGRGMQIYSAD
                     TCYEGDWADDKRAGRGTITWTEGRYEGDWKGDKRNGWGTCVYASGDRYDGQWKNNERY
                     GHGTYTWPTGQRYQGQYVSGARSGYGECLYADGICHKGYWKNGLGHGHGTVTYPNGDC
                     YAGNFSRDERIGYGVYVWADGMQYRGEWKADKPHGKGLLIKPNGARYQGDWAGDNRHG
                     HGIGVKPDGSRYDGQWHDDERRGTGTWQYADGSSVRGEWRHKSLVSGKVVHHRDGAIV
                     CISDSVCVACAIVAAAQPPPEDTIMAP"
     gene            complement(751218..752312)
                     /gene="pino_cds_453"
     CDS             complement(751218..752312)
                     /gene="pino_cds_453"
                     /codon_start=1
                     /product="Morn repeat"
                     /translation="MKRHRGICEGADSLCDKANGPCASKRRRRNRRKDSRAFATPFFD
                     RLPDELVLAILAALGDPRSLVSWGQTSRRHHELANDPLLWRRLCESHFGPLLHRNFAK
                     WGKSWRWLYRAQTHEVAVTGPDVGAILVHVRDHQYIYWGDCRNGLPHGYGLALQMPTR
                     HCDGSRGLARVWTDAADAVATTDPGYEGEWSNGQFDGYGTYAWPDGKHYEGDWRDSRR
                     HGQGASTYANGDHYNGEWRNGERYGHGVCTYADGSRYEGEWDGGILWGKGLLVKPDGW
                     RYQGDWRDKRHGYGVCVEVDGSRYEGRWRGGKRHGTGTWHYADGSSARGDWHHKSMTW
                     GEVIQHRADAESCITDVACKACAVVANKWF"
     gene            complement(752824..753600)
                     /gene="pino_cds_454"
     CDS             complement(752824..753600)
                     /gene="pino_cds_454"
                     /codon_start=1
                     /product="Morn repeat"
                     /translation="MCGRGINTWANGSRHDGQWNKDKRNGPGIYVRADGFTCDGQWLD
                     DKHVGHGTATWPDGTHHIGIWADDACNGHGMRTYADDDRYIGGWKADVHHGRGIRTYA
                     NGDQYDGDFIDDERAGHGTYTWTDGTKYNGEWKGDVSHGKGILVKPNGTHYHGDWIGD
                     DRHGHGVYVKADGSRYDGKWRHGKRHGTGTWHYVDGSSAQGEWRHKILVSGEVVHHRT
                     GAKSCSADSPCTACTVVAMAQVPQKDIRSTCTAPSGGSFD"
     gene            complement(753766..754179)
                     /gene="pino_cds_455"
     CDS             complement(753766..754179)
                     /gene="pino_cds_455"
                     /codon_start=1
                     /product="F-box domain"
                     /translation="MKRPRDIDESAGSIDKTDGPYTFKRHWQNGRKNDRAVGTSFFDD
                     LPDKLVLAILVALADPRSLAMWAQTSRRRHDLANDSFVWRRLCELRFGPLLHRNFARW
                     GKSWRWLYRAQARAAAATGTDVGAVMVELCASNCV"
     gene            complement(754682..755389)
                     /gene="pino_cds_456"
     CDS             complement(754682..755389)
                     /gene="pino_cds_456"
                     /codon_start=1
                     /product="Morn repeat"
                     /translation="MPTRHCDRSRGLVRVWIDPASAPTTIDAGYEGEWNNGHFDGRGT
                     YAWPDGDYYTGEWKNAKYSGQGIYVLFDAGECYEGEWKDDKRNGRGVYTYSDASRYEG
                     DWVDGRRNGFGVYTWPDGTKYKGKWEDDKSNGEGLLTKTTGARYKGNWKDDGRHGRGV
                     CVKKDGSRYDGQWRHDQRHGIGTQHYADGSSVRGRWRYKRMVSGEVIRHRRSGALCSL
                     DSTCMACAAVVALQTMP"
     gene            complement(755411..756073)
                     /gene="pino_cds_457"
     CDS             complement(755411..756073)
                     /gene="pino_cds_457"
                     /codon_start=1
                     /product="F-box domain"
                     /translation="MPTQYHELKAKAEVGESLILSGRRCADLFPVIGHSVNGKTTGHF
                     LLVVGRRRARWRAICTRQKNSVYPQPMGQRRHAHKVAGSANKRDRTGGSRMQKRRRRR
                     PLKGNHVTKISGFDWLPDELVLAILVALDDPRSLASWAQTSRRHHSLADDPSLWRRLC
                     ELRFGPLLHCNFVGWGKSWRWLYRAQACEAAATGTDVGAVHARVRGRKYIYWGDCRDG
                     LP"
     gene            complement(756555..757826)
                     /gene="pino_cds_458"
     CDS             complement(756555..757826)
                     /gene="pino_cds_458"
                     /codon_start=1
                     /product="F-box domain"
                     /translation="MEQDGATALHDQGDRTARPSKRRRRHADHDAGASDPFGRLPDEL
                     VLAILAALDDPRTLALWAQTSRRHYALANDPSLWRRLCESHFGPLLHRRFIKSGKCWR
                     WLYRAQARVASSVGVDVGATIVPACGYDHCVYWGDLKDGLPHGYGLLLQLPSRHCDPD
                     LLPTRIKVGATDANVLTTVDIGHEGGWHNGQRHGYGVGVLAGGNQYEGDHADDKADGY
                     GVSLWDDGSTYEGKWRDDKLDGYGVHTWPDGESYRGVFLDNQPHGYGSRSYVNGEIYR
                     GVWRCNQRDGYGIHIDADGAAHKGEWRNDVANGFGIFTKTNGTTYRGDWLDGLRHGYG
                     IYTKANGSRYSGWWQNNLREGRGMWEYADGSCAQGRWLHRRLDHGTVTRHRGGESPCP
                     LDFLCAACTTIAASSEPYPMDVYQEDVDADA"
     gene            complement(758270..761011)
                     /gene="pino_cds_459"
     CDS             complement(758270..761011)
                     /gene="pino_cds_459"
                     /codon_start=1
                     /product="Hypothetical protein"
                     /translation="MDTTEQERERELAKDSGACETATRQPGQLFELSSSKLCAKNDMA
                     VFSDIATLGTDDASGTTPTSPASTAAIRLGACPLRWLAAREIALALKLADCFGPSDVA
                     HMASVSKGTLTTLADVVVRSEGAPVSLDLARSVVAYLPQAKIMADAVTVLINERCGLV
                     DWSRGSGLCLLPMPLATAILEGRHRWPFRALWDIAARAFACGRHDIVAHCRGLQEAIC
                     TCMRAYVRAAMNPWSWSQGVALGSWLCKAAGLALPLDPSTGSLAMVLTDAKSMAALQM
                     AHKAGKWGHVQLVDFAIDAARESVRAGIDLVRGGRLVMRILGPEPRDRCAHAAAVAHV
                     LVRVVAGIGLERFQTVDDDHITRFDALFERMTTLTLAVIIPALEEVETPRATPPATPS
                     DPLYRASVATTFGKLCRALLRLLDSAVEAHTMHAARLIGTSCAIKTHAMRAARLIGVI
                     VSAGAQPPQCTRWLECIWPALCRPIFSGGRPDRVALSTAAVQGMAVRMAAAQDRSLCG
                     GLHLRKTIDADLFRDLSTALDPCDPRATLVDCLAHFDPALTARVFFYLDPEDLGALAV
                     CSRLALTLVASMVIPPATGGPQRALIAGLCDGGDVPQRLGIDAPETVVHLTPTAGPTG
                     PDAVETMRLVSLACFCLPAIRSALCRMEVELPYGFVHESSEPPRGLYTGILPALATAI
                     GNAVACGSGAAAGEALALGEHAALLGERIGADIDESTHIVHGNGKGKCADSWDTLAIY
                     DTDEKRSWMVDGRAWHVAAAVARVAGLRCDVRVLRLACAMAAQKMGSALTGLVEVRTV
                     GSRKHITVRRHSADETRVHVMAILLNAVTSRLAFVGADQRDAPTAFVVELCSHLCRDI
                     LAAHALARNSARNTAMPAVVAACRTASLRITLFCARAGVPVCPEWTHTVCRLAAVST"
     gene            761480..763099
                     /gene="pino_cds_460"
     CDS             761480..763099
                     /gene="pino_cds_460"
                     /codon_start=1
                     /product="Hypothetical protein"
                     /translation="MATIDDLPLDVLDLLLNGDEHAPPALDPQYRSTARLVSPQWRAV
                     IETVGCGAKKALTAANPTGADAAAWMGGRLICASTLVDRLVHAHSVDDMDAAVARFLD
                     AWAPIVSWLHAASVLALLEPPPPFSTRRASPLATITMRAVDCKIARHVLDRVCARICA
                     DDSTSAHIAVPSWRRTFDRGVLAAVAADYSETAMAFLFRTSTVLACDPTLTLDSTESL
                     VEYIASQSWDHIAGHDAACTAQALLGLIKPAPFHNTGDAHDLWRWMVCVIGWTYRKGA
                     WTNVVARTSRFKVYDLCAPRQDFSINAKAAAHYANVDLLRRLPDHLYQGTKIIADVLD
                     GPSSRGAAAAALDWLAAERAFVPTHIQADHLFGTQPWLSGGRRRLSLLLDRWPHLVDV
                     ARQCTHQTVASVLGALGANDLAEAEHMMEVLSPHAPDLDEGLWPTLVDRLHHRGYCQV
                     VDLHIACALAVQRDHSPLWQAWCGDMTKPIDKRRVLACTTRIGPGALDARAKEELDGL
                     FACLASHGLLIECASFACDDDASHGYARALE"
     gene            763791..764744
                     /gene="pino_cds_461"
     CDS             763791..764744
                     /gene="pino_cds_461"
                     /codon_start=1
                     /product="F-box domain"
                     /translation="MGTIRTRLARSYEGVARDCDESHDGSRTSGLKGLDDLPDELLAA
                     IAQSLDCVDNYSTMRRVSRRWRTIVSDRTLLGPPLCMKTHHATEAISEKKKKTQLYAH
                     SPMCPRHRLACADAIRAGAGSDTLRGLKSMGHKFNGDAVMAAILADDVPSLAVLTTSK
                     GCEIEREHYNAAAQYGRINVLARFLAAKGATWRNSEVPKVAARHGHLDCLKLAHRSGI
                     KWDAKVAMAAARGGHLNCLAYAHEYGCPWDASALYAKARSHGHKACCDYIARHTTKRL
                     PNEPRDTILAMFAVLVMVSIFVGVVVLVIAVVVRHGSKTSE"
     gene            765592..766398
                     /gene="pino_cds_462"
     CDS             765592..766398
                     /gene="pino_cds_462"
                     /codon_start=1
                     /product="Hypothetical protein"
                     /translation="MARLHGQLLDHGRVSRRWRAIVSDRALLGPPLCFRPYLNKALSD
                     TKKKKKKCLYAHAATCPRRRLACADAIRAGAGPVVLRSLKKLGHKYNNDAVMAAILAN
                     DIPCLALLHTSKVTIKCAHFEAAARHGRTDMLAWLFANEVAYSWNSNILAMAARYGHL
                     DCLKLAHRSGIKWDEKVALAAARGGHVDCLAYARDYGCPWTPYTLYKEAKTYGHKACC
                     DYIARHTTERFPNEPWDTILVVLGVYAILVAVAGLIFLVVVVAINYTKGE"
     gene            766912..768054
                     /gene="pino_cds_463"
     CDS             766912..768054
                     /gene="pino_cds_463"
                     /codon_start=1
                     /product="Hypothetical protein"
                     /translation="MHAATTPSTLGGRSTRTDAHRSLATGREAHIRDLVCAIYDDDVD
                     QVRRLLTAETVSLTKILITRDSMRALLPPNVASALKSGVPCDVRYNGWTLFDVAVHLG
                     AVRTIAMLGSLQKPARTVEAYLHHAVRRAESGLCSLGDNTDKDADSDMAAADCLATYP
                     LDDVIRALIVSIPRADRLSEDDPNPLTDLRHRAIWGIVQSLSNNVTAAICWIKDEGGW
                     TLSEADQARVLSAAGDEAITLLQRIAKAIERGLSTDDMAPHIDRLAALVVARRFGEAK
                     ITAALNVLLEAGYASDSDLGAVPLVWIEEDWQSIPKDWLDNLSEQAFAVYDAQRFESW
                     AKACKDKADLENWFVERVSWEINAAFLRAYDRVVATAPSFDSTLVA"
     gene            768434..770526
                     /gene="pino_cds_464"
     CDS             join(768434..768800,768857..770526)
                     /gene="pino_cds_464"
                     /codon_start=1
                     /product="Hypothetical protein"
                     /translation="MKRQHENSLSQDGLAHAQGTNKRARTEDPPRSYPLVENSGITQL
                     PAEILSLIANGTDAHGRPLLDPRYRFLLRQVCRGFYDSVSSPSAVDAARLRAFGQTGD
                     AWVSGRAASLALIAEKIRSEGWITAWMASASVDSIVADFARVAASLLPACPDWFPLAD
                     AWPRSWDCWKQATSTLITTRDIARRNLVCTACCLGRLDVVDALLGWFGIADLSTLCPA
                     IHVAAHRGRITTVATLLSYMKQRLDLQIGFHASTMRLLFDTAAAAAAQSKDLQMMRHL
                     VGWCLQEHNQRDPTEALVGPEEGGAGFRPPILDDLLAAARVRGVACNDVNLDPYWVSM
                     DRLDVLLQVIDTSRFDAPSVLLMAVVSGSLGIEDALASCWDEPGFVVSDLPLLLYHAF
                     KRVNESVRAPACDAFARGMERLRDRCDIPCHRMLYLADRLNGHLDLATCVLKLWPPVP
                     NTTKSSALLKGNLVYVAVRQGHWGVLGKIIRAYGLVDDAPSGMWSQSDSPYDSDSSSD
                     SDDDDASSDDGSSNDDGDGSDHIKCDRTRLAVHGRRQRHWWRTVADGVSFARRTDRSL
                     TVCMKKAKRSLAALGLICQMAYVTGRITPDAVSAHTDASLFSSIPEDDCKIDVSDWSR
                     WCDDMDILPRRVYQSTETVGHGETNKKQDLVDRCDRLIALLGGAGLVRCLPASR"
     gene            771245..772341
                     /gene="pino_cds_465"
     CDS             join(771245..771436,771523..772341)
                     /gene="pino_cds_465"
                     /codon_start=1
                     /product="Hypothetical protein"
                     /translation="MDVDADRSQYGTNTMSALPPELWEAILGGADQVGGLDPASLLAL
                     ASTGRVGQQIVRAATQPFYAPLALFLAMADCVLRGYITHGMNALANGEDLAALFEQDR
                     FGNPDAQHPYYTARRYDWATRAPNDTRHMMITSPRHQEVAAAFRAFTQASKTLKRVSD
                     GGPSGRHAIDILLIGPDSPGPFDAQGQLADDAQVRMTAVATLDPQQISLLTGGVVASD
                     AIARWLRDLQLTTQETNMQYRQLAAALRMPEAYARVMALIGQVAADASPSAPCRRALA
                     RSPLMPRFDQLFTASPFLAVVSPTVVYLVLRDLGSVPIEWAIKEAGIERREGTLGGCV
                     LS"
     gene            773051..774001
                     /gene="pino_cds_466"
     CDS             773051..774001
                     /gene="pino_cds_466"
                     /codon_start=1
                     /product="Hypothetical protein"
                     /translation="MVKTCRGHLSVRGIREPTCTALNSTEDGLLIATRSLTESPFMRP
                     MRTHIRRVDPANAKTPMAAASLGMLPRELVDMITRRITAIRDMGAWSLATGLPTTALQ
                     VDAARRGLLQNGERAVMAGAPVPIVEALLAHNVGDAYALLPVACKSGRTDAVKCLLVS
                     LYRTERWTVDERGQCTVRLSTAYDRAVKAAINHDHGPALGCLMDGAGLFTLDDFVSGN
                     VAKAAAFCEASGRLRCAAVLCRRLKARTVGACPIRSHQFVVAVLPPVLFYDALQCAAA
                     IFEACPALWTDAPLVIEHASAVGAFGVARWIIGNRPKYNA"
     gene            complement(774295..775999)
                     /gene="pino_cds_467"
     CDS             complement(join(774295..775615,775722..775999))
                     /gene="pino_cds_467"
                     /codon_start=1
                     /product="Ankyrin repeat"
                     /translation="MEATSGENINQVLPVELLSVILNEHLDRDYDDVCAESACRLWAD
                     LLADARLHRQRRGKWFADMAAVNGHLNVLKWARANGRPWGTLTTGFAARWARANGCPW
                     DTETCARAAKRGHFKLLRWAHGNGCPWDATTCYSAAKRGHFEILRWARKRCCPWDQRT
                     CYGAAKRGRLDILQWARAGGCPWDQWTCTMAATNGHLDVLSWAYEHGCPWSGDVYSVA
                     ALGGHLDILKWAYEHWGIRGEHDVCALAAKGGHLEVLKWVHERGWPWDARTCRKAAKG
                     GHLDILQWARANDCPWDARTCHVAARNGHLDLLKWAHADGCPWDKHACNAAAIKGRLD
                     VLGWLRANRCPWDEWTCACAAAGGHLNVLKWLRGSGCPWDQRTCTRAAKSGHVGVLRW
                     ARANGCPCDDEDCLAGAAEYGHLAVLDLMLASGCALPKAVCTEAARFGHLHIVKWARD
                     NGCPWGSAVPFAAAMAGRLEVLKWLVMNGCPWYPQSCRDRAAWIGHDHVVAWVDPYVA
                     DIQARPEPARRRRSRRRRRSHKRR"
     gene            776257..778862
                     /gene="pino_cds_468"
     CDS             join(776257..777186,777957..778862)
                     /gene="pino_cds_468"
                     /codon_start=1
                     /product="F-box domain"
                     /translation="MEPTTEADLPDEMLCAIMRHIPTKWVWLVALVSSRWCRCAVTVT
                     QEAAQGRPRVHRATIDELATSWPGKEYMDEAARKGHTSLVLWLRTCLGIHWRTHTVRC
                     AALAGRQHTIDHMLASQWFLPVDEPCLVAALIGGQGLALARSIHRAGQPWTPMARAAA
                     VALGDPSIVAQLNDQRRRRNGDDLAVTLAVACGRPDLLHAMRATTAEIAHAHRVIARQ
                     KEWSDTPFDHQALLDYATIRGLCGRDLAMMILIRFDIGWTLMPRLDDCGASLCKPVVD
                     HHQTAATAHSHTRRERDLQPPRGAPEPLLPPPDQVIERLADISTPAVVALYETDRNAR
                     ALIDRMTHVLYAVRDDGTITQSRVPLIEYARIGTTLGKSQPLRILLAAAVCTLKGLAN
                     VEIATDMEVEFGQFDDDAAYTLVEDYSVPITAGGTGSFTTLVDANLLPMLLDFDADGV
                     ALTFPARDLDSIRNLVEGPIPGDLPQVARQWYDWMTGLVDVLAETLPPIAARYQMILR
                     GRRDYDDERSTEPFMAEAVDFEGSVQVGAIMSNADGEPFIDGNDDTAPYLCAFWPPSR
                     CRRWRRPSGSTCHRDFCKNGPHCNREPAILLHDPFRMLSRRTNMP"
     gene            779355..780521
                     /gene="pino_cds_469"
     CDS             779355..780521
                     /gene="pino_cds_469"
                     /codon_start=1
                     /product="F-box domain"
                     /translation="MPSADSRHWSLCRHQKIRIGFADNNGKLLHQDTTTPRVTRQAML
                     IDDLPEEMLCAVMRQMPPRWVGLAALVSSRWCRCAMTVTQETRRAWPKMRTTRLLWVH
                     YVLVGKKFMDGAACDGHTSAVVWLHDQLRIPWRRGTLRKAALAGHKHTVATMLARRNE
                     VSVDESCLVAALIGGRGLAIARLVHRTGQPWTPMARAAAVALGDPSVVAALNEQCGPR
                     NADNFDVALAVACRRRDLLRAMQATGTEIVHAKRLLQRGAEWGRMPLAHQSVIDYAIR
                     DKLRERGLALRILAHVTVKRRKTRNSDLFDLPWMGRSFWLHNDLHDFERLSPVWRETI
                     DWDLPAPPKPAHAPEVVQCIQRKGRDDQRRAYRGKMMPPPPRRRGRPLGSRHRH"
     gene            781188..781577
                     /gene="pino_cds_470"
     CDS             781188..781577
                     /gene="pino_cds_470"
                     /codon_start=1
                     /product="Hypothetical protein"
                     /translation="MAELFAYIGATGYGLAGGTLAGTTAALLRRFIPYRYSLPIVVAI
                     PCVGAAKTIYDLKRRPHYAYPDQDSLDSVHATILGILVGGYAVSCLGVLKVSSAWRRS
                     LWLARRSYAATARDARRQQLTSTRPRQ"
     gene            complement(781846..782581)
                     /gene="pino_cds_471"
     CDS             complement(join(781846..781980,782072..782189,
                     782268..782581))
                     /gene="pino_cds_471"
                     /codon_start=1
                     /product="F-box domain"
                     /translation="MEGVDQNCGKKAKDNGSTCATDDETDLDDVDNINERLPAEIVSM
                     ILAYLDADYEAVIAERVCRMWASLSAPIRQARQRREIWRRRWLTMSTPQGRRQAAEWH
                     QKCGGFSAALGGHLHVLQWARANGCPWDELTCDEAAQSGHLEWAVANGCPWSADECLT
                     EALHHGHKEVGAWIQAFRFNSDLGWRPK"
     gene            782891..784066
                     /gene="pino_cds_472"
     CDS             join(782891..783742,783806..784066)
                     /gene="pino_cds_472"
                     /codon_start=1
                     /product="Ankyrin repeat"
                     /translation="MSIDGLPDEFLALVLYHVPCIVRARDCAPVCHRWRAIVADDKAL
                     EPCIHPRHLCLGKLAEAIGTWGNSLSSLMGADSLPRSSPVLLAAVSKEHLHCMSYARS
                     KGHAWGPDACDDAARCGRLAALKWLIDAGCPHLVALYEGDSCTGHNLVGSPRGVHYYL
                     HRRNIKPLMKEAIKGGHVSCVDWLVSRGMRLDPTTCHMAAKRGHLDMLRYARANGARW
                     DESVVCDSAVRGGQLDVLTYVQADPLLLAKCPWYVAEKDGLPPRFCCGIGCKMSGLGC
                     SMPSTTRTIVETDHMGLLVFAHTHGCPWNTGACKEAAYIGWLEALQYAHENGCTWDWS
                     ACMDAAMRRRVGRRCRPYLKEHQLCASDGTCRFKSV"
     gene            complement(784217..785320)
                     /gene="pino_cds_473"
     CDS             complement(join(784217..784398,784492..785320))
                     /gene="pino_cds_473"
                     /codon_start=1
                     /product="Hypothetical protein"
                     /translation="MQTPYNDFAGFALLPNELLADIVKRIGAFDPFDATGLCDVDPRM
                     RMWCQEPVFDAALLPPTIQRNLDAPAADEHGPRKISIAEAVRARTRRDTMRRCVLYAL
                     YSLYAYMGQAGQGPVGIPVLPFDPAKTDDDWAALLFPAMYDEYGSRSFAVVYVTLHDM
                     GVQLGNQIARVNRQLVRDPPRDQHYAAVNNVLTQALIRAVVDTRSEADTPSLCASIDI
                     QRAFFDDADLPNGHYTKSGNAAVGLNVLRVGHLWYPWTDKYETYESAIEGEPIHVYND
                     ALRRARWPRTAPPGKEEEKEGDSMDWIMDQIMSEHTTINIPDEDLAAFDSSDSLPWLE
                     YD"
     gene            786360..787064
                     /gene="pino_cds_474"
     CDS             786360..787064
                     /gene="pino_cds_474"
                     /codon_start=1
                     /product="Ubiquitin-like"
                     /translation="MSAAGATTASDDQRPGTTTQVEARPGVGHGGVVFVTQGQRSILA
                     VVKYMGDRKFRLAPWPHNSLYGAEDEWTVDVATVAVDGDWVDAPLPKCCVCVNRKAGC
                     ALPCRCTAPCVCIWCAGRLDACPQCRTPFQSKGVPWASLGYQGNIGALRAPCAWETIR
                     VFVRTLTGKTLTLRLRLNSTVRHLKEAVQDREGIPPDQQRALFAGTEMDDRLALTFYH
                     LRDECMVHLVCRMHGD"
     gene            complement(787285..787758)
                     /gene="pino_cds_475"
     CDS             complement(787285..787758)
                     /gene="pino_cds_475"
                     /codon_start=1
                     /product="Ankyrin repeat"
                     /translation="MWGAARGGHLAMVKWAVGDGRSYNESTCAAAAQGGHLDVLEWLR
                     NQGCPWDESTCAGAAAYGYLDLLRWARANGCPWSKSVCFHLAGDGHFETLKWARANGC
                     PWGRDACWHAAQAGHLDILRWARANGCPWDRDHCAKGAAAMKRHHVCTWIDAQPY"
     gene            complement(787771..788478)
                     /gene="pino_cds_476"
     CDS             complement(787771..788478)
                     /gene="pino_cds_476"
                     /codon_start=1
                     /product="Ankyrin repeat"
                     /translation="MDTLPVELVHAILRHVALFTHARFVCRVWNDILTDPTIAPPAER
                     LLGKAYTALLAETGAENVVRWARKNGCPWDVRACEGAARGGHLALLQWLVGEGCACSD
                     MARAWAAVRGHDDVCQWLDARGCPNRSDYQWHNAITGGHIEVINWLLSLGHVWPDGAC
                     SKAAKHGHLEVLQHARRHGCAWDATVCTHAAHDGHFHIVQWALDQGCPCDCYPASKAA
                     RRGDLALLQRLVELGAP"
     gene            complement(789104..791380)
                     /gene="pino_cds_477"
     CDS             complement(789104..791380)
                     /gene="pino_cds_477"
                     /codon_start=1
                     /product="Hypothetical protein"
                     /translation="MATPTSLATATVEMDDRPATVNLVLVCASRPSTTWTVADVDARA
                     LCLASPVFAAMLDGDFVESRLDTVRLALPLDPDTPQDTVEVFVKRCQGHAVLDAITTL
                     WLWDTLAFVFIDMAPFIGPLVDALLHPDKDARVDPALVIGAHALHACPVRGQTLATSM
                     VASAATGHFGATEPFTSPSDGVARRVTEYMSCGETIVRTWFRLSAGDCASMVEKAAAW
                     IVDARRGLLAAAATTALADWHRTFARLDAYTRVPFAWGVCRPLTAGPAPCDGMTLCAL
                     AACEDDSNALVGSHDDFLEALANDSPTIVQALRACALLGDPAVVIAGGAVINAVQAPH
                     LRKRLDASDIDLWIVGPSDVDRRYALDRVVRTLFDALPGCRSSHRGSVVTIETVPPTP
                     GACKPDARTERIQIISTGARSGAEVISRFDLAHACGWHDVDGVGLSWDAMWAIVTRRT
                     RALPGVEPIASRLSKAVEKGFAPDDVKADADGEPMYMVDGATIEWHPTADAILADFSF
                     MPIQRNGDPKPRPPVASPFVPHARHYDRCDDGYDAFDKHLHWRGFAWAQPTRVETPLL
                     TVLTTEARTARFSSTVKTRLTLALEQNSPLWEVISNADARFFERANRHARGSEYRVEP
                     DDGPEPLPRDPMAALAVSLLRHCHPDPMAATRQRHVEDFRAQCHPTLTRCSDGTGHSF
                     AIDCTLMSRMVDGLTGGTYDANAIVVGSHVAVTMDIAGGMWNDWGGGRLMIRLISARI
                     YPSHMHEVFNALSSLPSP"
     gene            791916..793883
                     /gene="pino_cds_478"
     CDS             791916..793883
                     /gene="pino_cds_478"
                     /codon_start=1
                     /product="Hypothetical protein"
                     /translation="MQHGNNGDDRFWVDDSDSDSSSMLSDSDSSSAPSNSDSDDDDNE
                     SDSDMAVGPRLRPANPKKLGMFVHLLLYCRPAAGEAVCAYLHTTTDVEKTPSAYELDG
                     LAQSMLSTLPRHVADNKSRRLDYTGDMSEHINAFFERLDRLADAHRQERERLHASNRS
                     LAIGKVFSTWPTPDRERKDDISTPSYLLVTGTVNRKRTAGLFAVRFGPGPVESSRLIG
                     KIQLDGCAPVLDADAAGPVLGPYADLVPTLLRNLLLDLDPYRLDSRSPPGLVTRAIAK
                     RLVHAKPPTLPPHAYAALWPSDDTDYPTSYWPNDQDIEAAWFTQTQLQAALGTIAAYE
                     AAETVGLDASRYGRTDPLGAASLCDRERQLEGLGLLRSDGGGPLSLFQTAQVRIVEST
                     WGVPLVDLPDDIAAPLALAIWQRACRALRAKDGTTEHASRLLDVAHYWGVQPTEVQRQ
                     QPEWLCQDLMTEAVVRATSLGRQRAVPLAWEDTFRPLFPSRSEHETMWCVEMASIQWD
                     DETDEDDHDETDDHHRPRCHETIEQMHAHVAKAFEHIYQRPPRTPDEPLLAKAVDIAM
                     GARAVSTGAARRAPTSAKEQAVIALGIVRARLDVEPKQLSDPSMFPKRLCGAFHQLPL
                     VTDIPFHRYRFDNDSDDVGFGDQEDMLRDLL"
     gene            complement(794324..794665)
                     /gene="pino_cds_479"
     CDS             complement(794324..794665)
                     /gene="pino_cds_479"
                     /codon_start=1
                     /product="Hypothetical protein"
                     /translation="MNRIAPTRVPKRPTSRFDPSWRASMRKACPIPETWARTTWVVYK
                     NGALWLTTTGTACSFIYGVFLGVHEGHGPVNQSAVGVSCAIRAFPYCAVAAMNWPLIL
                     GYGCWYKYIAD"
     gene            complement(796136..797374)
                     /gene="pino_cds_480"
     CDS             complement(796136..797374)
                     /gene="pino_cds_480"
                     /codon_start=1
                     /product="Morn repeat"
                     /translation="MPDEVIMCVLMALDDPRALVLWGLTSRRHARLAHDDSVWRRLCE
                     LRFGPLLHREHARWGKDWRWLYRAQGRIGATTGADVGGADVDIRAYGRHVYWGDLKDG
                     KPHGYGLALKRSTGHHQKGSFVRAKNESADPAPHSCIGYEGQWRRGCMHGHGVYTYST
                     GGHYRGEWRDNDRHGFGVDTLPDGSCYTGQWCSDDWHGYGLRISVPDGRTYRGQWVHD
                     VKHGFGVFVYPDGARYHGNFRHNVRDGQGQYTWPKGKRYDGAWCNDVRHGPGVVVYTD
                     GAYLCGEWWEGNIRGHGVLHRPDGSYYQGECADDMRHGHGVWVKADGSRYDGQWTRDR
                     RDGEGTWDYPDGSRAQGRWAHKEIVSGHVVWHSGGANSCHPGSPCKACTVVGSKAPAQ
                     DLMTLAGDAQQPPSLAETDD"
     gene            complement(798327..801208)
                     /gene="pino_cds_481"
     CDS             complement(join(798327..798477,798560..798712,
                     798792..798893,798970..799312,799406..799480,
                     799588..799739,799824..800023,800116..800285,
                     800357..800433,800502..800644,800724..800943,
                     801021..801110,801183..801208))
                     /gene="pino_cds_481"
                     /codon_start=1
                     /product="Ring and ubiquitin domain"
                     /translation="MHAQRTQPMELEERFQVLWVNEDRMLSVTLPCADIGSLKSVLAE
                     QTGIKGHEDLIQLCMGDVDMTRKTDKALDYFLVTDEPWFVRTPVEVSIIGFMSANGES
                     LIGGDVPQINAKAMVRAGSNYRAIKNALKQEGLPTNDYRLVTADGARLSSIRDVGPLT
                     MTTAKLGLYLRPKGEVINITVTNPPGFAKSGKAPSVLLTPRKDSTIASVKSRIISQMC
                     MGRQNVCLFYRGVPLGGLGDASPEDNRTLGDYGVEDGARLNWQLACAPQSGHDGSLDQ
                     TEDDPIVIVITRPDGGLVDIKTVDRTKPIATFRAWARDYLLALNGETLDQDKTWAEHG
                     IEAGTWLSIVPKPSRCFQLFITTLTGKVITIAAGPANTIEEIKVKIQDKEGIPPDQQR
                     LIYAGQQLEDSRRLVDYAIRADATMHLVLRLRGGGGGPPTSAMFVDMENTGAMRRQQW
                     SKSAPDWRIVRPGLCVEGRCKNVDCAAYGRMVIVNKGFDTFDLLLDTDTCVCPACGDH
                     VQPETCAFNNCWWKWNGIKKGDSHKYAGKWTLADDNYHCFAEDEAGSVEWTRLLIRAR
                     SIPSAGKSGKPAPKAFDMFCCICISSIGTSGDHRVLDCGHAFHSACIDAWVARSPTCP
                     HCRHKVAEA"
     gene            complement(801736..802857)
                     /gene="pino_cds_482"
     CDS             complement(801736..802857)
                     /gene="pino_cds_482"
                     /codon_start=1
                     /product="F-box domain"
                     /translation="MAHCKIDRPFDRLPDELILKVLQTVGDPKSLGAWSLTSRRHHRL
                     ALDESIWRRLCEIHFGPSPFGPPLPPHVNWRWIYRAQSHPARRRGRDVGALKMRGPGV
                     YWGATRNGRPHGFGVDLDGRGITCNSILRKRLDAPGPRPVDINKVSRRFQGRFVAGKK
                     TGLFIDTHDDGCRVERDWNHDDRAAVTLPSGIHYEGGSQYDEPYGRGLLTLPNSTRFK
                     GHWHFTGSPHGNGIIIFANGDVYSGSWRYGARLPVGTYMWAHGGRYDGELDNADRMHK
                     IHGDLHQDPSGGCTLEVFATKVDGWRTKRSAGGDTCHYRRCGQGTLTYADGSRLTGMW
                     DGAVCTAGQIIDHSATGESCPDPDVACMACKALAKRLPA"
     gene            803175..805178
                     /gene="pino_cds_483"
     CDS             803175..805178
                     /gene="pino_cds_483"
                     /codon_start=1
                     /product="Hypothetical protein"
                     /translation="MHQDDDPCPSSSDIEFESSDSSDNDDDDDDDEWLFLAPASPCVA
                     ATKQDDHAVDWTGILRDVRDPVVGMWSALAARPVACEAACAYLRRPLDGKPHKGTGNV
                     RNLALDLARALQPLYKFPSPLPVSVWDLDADDLATLLTNLASAQRKQRARLYGSNCAI
                     AIGHMFDGWPIPDHLYQPVSARAVGPQTMYLMVVSKAPQEIVALFAVHFGPDGSEWSR
                     LVAKIHFYKHAPDRASDIDADAIGPGSRSYADLIPTLLRGVFACWNPGLSHRIGDRQR
                     GPPDAIIDAIARRLLSTHPVRLPPQVYAALWPASDVARKHKDWQPDKQEIAMVWLTEA
                     QLAVASGTIAAYEAAQIVGTNAPRYGRTDPLGTKSLRGCRPQSGVGMLRSDGTGPLSL
                     LQMAQVRIVKSTWRVPLVDLPDDVAAPLAVAIWQRVCTAQRAKDGTVDDAARLLDVAR
                     YWGVQLTEAQEGRPEWLCQDLMPMALARSASLSGGRAVPVAWEATDRPPFPSGEEKDR
                     RFRIGMDVDHDGRHQFARATPTEMCTHVESAFATIYGRPPVLGDGPLVQKATDLAVRA
                     NALGAPPEGVGGVCASHQAIIALGVVRGGLDVGACVLSDPYTATDVFNGIKTQRPEPS
                     VCLFRRYRPRRGVDGSDANTDDEDDAEDSSDSPTPFWSTPEFL"
     gene            complement(805335..806632)
                     /gene="pino_cds_484"
     CDS             complement(join(805335..806012,806135..806632))
                     /gene="pino_cds_484"
                     /codon_start=1
                     /product="Morn repeat"
                     /translation="MDGMDCEHKILQLGDDAPFDRLPDEVLLRVLMAIGDVASLRAWS
                     LTSRRHHQLALDDLLWRYLCETHFGPSPFEPPLPPHVNWRWIYQAQSRPARPTGTDVG
                     AVRVQGSNARIYWGDVVDGQPHGFGVCIYGLCLIHDDGLSRKRIDIAQPTPMPMTSVD
                     RTQCEWYEGGLVASLPYGHGTLTLLNGSTINRKWHYFDRIQTLASGEACLVFKKKKAT
                     SHTGIYVWADGTRYDGECDEHGQKHGHGVMVYASGSIYEGGWCNGKKHGHGIIAYSNG
                     NRYEGDWHDNARDGHGTFTWPSGQVYTGHYRQGRRHGPGVMNYADGDKYEGAYHEGRR
                     WGRGTMSYTDGSRLSAIWDDTACTDAKVILHRTGDDTCSLSSEPCRACVALSTGNSK"
     gene            complement(807164..808390)
                     /gene="pino_cds_485"
     CDS             complement(807164..808390)
                     /gene="pino_cds_485"
                     /codon_start=1
                     /product="Morn repeat"
                     /translation="MEHVHAMPESMTDSPFDHLPDELVLCVLMAVGDAKSLAAWSATS
                     RRHWLLALDESLWRHLCEVHFGLCPFGPPLPVHAGWRWIYKAQSRPACSIGVDVGAAV
                     IEGGCKVYWGDTMDGQPHGFGLSVDQTSQMCDGHLRARLVAGGRADDSPLPDRSQGQW
                     AHGTMHGPGLTVYCDGTRVEVLWEHGVRGDHVRITYVNGNRYEGKAITCMPHGEGTKT
                     YLDGAQHQGAWEWGVPHGDGTTKWPNGDLCTGYWDNGELHAGTYIWPSGDRYDGDFVD
                     KQIHGLGSCTYADGSRYDGQWVRGTKHGHGVMTYANGHKYEGVWAHNQRDGHGIYTWP
                     DGGVYEGHYGMDRRQGRGTRTYADGSFIAGVWDGAVCARDPVVGHRTGDMPCSVHGLC
                     LACVALPQACPPDATS"
     gene            complement(808837..810129)
                     /gene="pino_cds_486"
     CDS             complement(808837..810129)
                     /gene="pino_cds_486"
                     /codon_start=1
                     /product="2-isopropylmalate synthase"
                     /translation="MECAHEIRQLDSDALFDRLPDEVVLCVLTELDPKSLRAWSLTSR
                     RHQQLALDDSIWSHLCDAHFGPSLFEPPLPPHVTWRWVYQAQSRSARSAGADVGAVWV
                     EINGTCRVFWGDVVDGQPHGFGLCVKDLDLTEDDGPARKKKKLGLAQETPTTCIAARI
                     QGDWVQGRLDGATVEIRADGLKVERRWNQGESETHGALTYRSGAQYQGGLGWCGDPHG
                     RGTLTLRGGATIEREWHRLDSITVRPNGNVCVHFFRSRQLPRTGIYMWPDGKRYDGEF
                     NKYGQKHGHGIIVCANRTIYQGTWRNDKQHGHGIMSHSDGDRYEGDWAGGMRDGHGTY
                     TWTSGQVYEGHYSQDRRHGPGIIKHANGDVYDGTYHDGRRRGRGTMSFVDGSRLSAIW
                     DDMTYTDARIIFHRAGNDPCSSAGPCSACVALSAGASP"
     gene            complement(810692..811567)
                     /gene="pino_cds_487"
     CDS             complement(810692..811567)
                     /gene="pino_cds_487"
                     /codon_start=1
                     /product="Hypothetical protein"
                     /translation="MTTHARPLEALPQETLWSIWDHCSFFDLVRLSRVSTPIETSVFA
                     FVAERRLAADWGIHFGSGRGVVQLLTWWLRLARHVVNADGYNGGIGSDHVVVVECYDA
                     IQCGAIHAWARRFGCASERAVYARFQPQHVYACECGLVSPLSSLTWTQSECDDSGYKR
                     ANCKRCRSFVTASPPDLGLLPADDGDPAWTLTHNAVIITADPGRMPLSVGPSVSDHHR
                     PGGRAQWAEKNRAPADDAKTDDQIKAWVAYAFGRAAQWLPLETSICATRTERLFSTNY
                     ISGLRHTPCPYQEKD"
     gene            complement(812049..812465)
                     /gene="pino_cds_488"
     CDS             complement(812049..812465)
                     /gene="pino_cds_488"
                     /codon_start=1
                     /product="Hypothetical protein"
                     /translation="MPCLYVDPHTPLPPRHSCNTITTLGAADRTMGAKESRTSATRHV
                     CVAQDGAIYGKECSYCNDHAYEHIVANDDSGRSFLRCRDCWRRHPDWYYQGYTWTCPT
                     SGCKICGTRPPPNTDDSDATMRMVGEALLLGIAKHL"
     gene            complement(813324..813776)
                     /gene="pino_cds_489"
     CDS             complement(813324..813776)
                     /gene="pino_cds_489"
                     /codon_start=1
                     /product="Hypothetical protein"
                     /translation="MSTKRAKTPEPADSMPSAKKACRRDYNERAMTRMAKRGDLDGIK
                     KVIDDVGYIPWHGIVQYKAAAHGRLPILELIYDKYVKGTRGSTCFRDEDEMSAAVLYS
                     GDDDTIRWMCCTTGNCFALSMPTITAPMKTTRSDPTILAGRTGAPSRG"
     gene            814119..814490
                     /gene="pino_cds_490"
     CDS             814119..814490
                     /gene="pino_cds_490"
                     /codon_start=1
                     /product="Hypothetical protein"
                     /translation="MSTAQTTANTRTRRPSRYIVQFSVTGLLEYDQSYIVTREQLAVV
                     EGCNITLDRLCTCDGCELCMAGSVDIKFVRSLPLARVTPQLLLLESQAINLDTIIEAV
                     REEQKRQRLSTKLTSVAQEEQ"
     gene            complement(814773..816058)
                     /gene="pino_cds_491"
     CDS             complement(join(814773..815460,815523..816058))
                     /gene="pino_cds_491"
                     /codon_start=1
                     /product="Hypothetical protein"
                     /translation="MECMQTTSGKRPAAVTFALESDPSTDGVGLAPRAPKLARLVSQH
                     FNQVKRAAIETTPNVPSRAVRRRVEHDHDNNDDDDSTDSYGRTINPDLQYKIMQTLFQ
                     LDPLDALRLAASDRRHQSILESIKSSIVRTSVLVPTDVPITAHRLLAANIGLSQRLGG
                     GTSPQDWEAAVAASLMEAFIAHARYTEYDTTYAGDAVTAVLDEMTLEERVSTLYEWIM
                     KGSFGAFLDRQRQSKFRTWLSRRGVRRGYFNRHDYQKYLLYLEAVGVRVGNHPRRPWT
                     GTDGKDLSTEDRALQPILSFPQTYGRIFPDAIDRAERPLAWRDQASGETRTAPIDSSD
                     AEATIRDYLDDMVRQHMVGPCAQIQAAGDLDVPTFSDFFPGAIYLANMVPDVALMMDL
                     RSSRIERLLGQEFKY"
     gene            complement(816760..817954)
                     /gene="pino_cds_492"
     CDS             complement(join(816760..816909,817014..817390,
                     817489..817600,817715..817954))
                     /gene="pino_cds_492"
                     /codon_start=1
                     /product="Hypothetical protein"
                     /translation="MNTFRSDSLCAAPALGDATRAFESEPVQLTTVDAVNQLNVFDFD
                     GTLFRSPEPNADKWTRASIATIKNGPKAKGLGWYQDVITLSPPYVPAVPTSDWWNTSL
                     LDRVRESMADPRSVTVLLTGRSESYRQRIEALVGTVGLVFDVVGMILLPVCVCVPCIW
                     KKSMILTLALPPSPMPPCVRVCAVRLKPPSGPSTIDYKTDFIGALIAKHMPRQVNLWD
                     DRVEQIKGFGAFLETHKRSSGIVFEIHRVEIPPLFLSEDDETQLLSLLTAKHDNHADL
                     AVRQDYARRGALSPSA"
     gene            818952..819791
                     /gene="pino_cds_493"
     CDS             818952..819791
                     /gene="pino_cds_493"
                     /codon_start=1
                     /product="Hypothetical protein"
                     /translation="MRLPMTTATEGLLPLPVTTPRKIKHTYVCRTSKAARWLFVCNAA
                     LLFIIVTVSLRTCNRHHRNTSQDAFADIKPTIDPHDAQERTHDPAVLAHVKAEPVQAD
                     IESSPRETPVADIYSNSIDNSGTAAVAKSGRPLRQAPSSVEYEIVRFVPDDRSDGPKR
                     FALAVERVGRRRLQDNIKCIAACLRSDAKCGWYSAKMARTEGAVLYSQWAKDVPHSRR
                     ADDDETRNVQVTLVAAPDEVADSVGWDDLRCVGPVRVFLGPSDDPAELLSMAPPRSDY
                     FVL"
     gene            820532..822388
                     /gene="pino_cds_494"
     CDS             820532..822388
                     /gene="pino_cds_494"
                     /codon_start=1
                     /product="Ankyrin repeat"
                     /translation="MHLSNSGAPPAKRLCPCSLPAIAPPRPHLDALLHIDDLPDEILG
                     AIFCRLTCLDTTTSVPLVCHRWRRVNGDPLVVPLRTCDPCADDSTPRKRESACVPTCP
                     LAPVRTAFRLAMGRRLPDDFGRRERPSDAVFCLYARKRDRFSDKWAAFADRCRPLDSR
                     PCELSAQNNGSLNVLDLANARWPCSPRVFAWCVARGTALSRLVALISAGCPIDNPVVG
                     MACAWHGRTDVLSLLVGFGARHAVDARAWQVAAMRGHIEWMAAARTRGFPRPTQACAQ
                     AAAWAGHVDIVAWVQDECDLCDPSVVTSAVASGASVAVLDVLVNSGWAVAPEAVTKAA
                     SRGAVDRITYLWSVLDKRHVPTGQTRSVFCCIEAARQGHLDCLAYLWVHGCPWDERVC
                     AAAASNGHLDCLQYARENGCPWDVFTCRAAAASGHFMCLTYAHKNRCNWDETTCEAAA
                     AGGHLACLAYAHQNGCRWDQKTTIAAAANGHTVCLAYAHEYGCPWDATVVEAALLGGH
                     RDCVAYAHRHGCPCRPSACHLYCARERWDVKVLCGRPCSLTTTAARRRQRHRIAMAKA
                     GPPPWAVACTSRPFGANHDPWNRLLASLAHQQQGTTPNAASTSAAIDLCRRR"
     gene            complement(822667..823809)
                     /gene="pino_cds_495"
     CDS             complement(822667..823809)
                     /gene="pino_cds_495"
                     /codon_start=1
                     /product="Morn repeat"
                     /translation="MDMDIDNGHSHAAPTTTTGLDVLVDELLVAILVRLPHANDIGRC
                     TLVSQRFANLIARDPDQFVWKRAAECRAQLIQLDGPWLAFAASTKGWSWIRRALDPWT
                     GCDCTIGFVCNGPYTDLGEYREDGSHGDLISIRNNGGWRFAGPVDDDGMSYAHVFHAN
                     GDTYRGQLLGRRRHGHGVYTYAARRDFLSPLRIEGGWVHGMRHGTVTITNGCNCTGPL
                     HGGESEGAHADNPGHLQGSAVQPTLAAHGTDPSRPYGYVATETWSYGAPMGPSTRIYC
                     NGDLYECERDRDTTTTVRMVLSPHCPDPRFRSVEIKSPDWRWTTVERPGDQGSFTLSY
                     PDPASAWSDMFCLYHDYFKAGFLPVEERDRAIIGAILAEAALDLDT"
     gene            complement(824449..825429)
                     /gene="pino_cds_496"
     CDS             complement(824449..825429)
                     /gene="pino_cds_496"
                     /codon_start=1
                     /product="F-box domain"
                     /translation="MTGMDDLPDELLVIVVRAIETAERHASIMLRLVSCRWRSIVDDV
                     HGPPVDPWVLGRCLEFLVQRGQSSTSPQARYDAFPAPWFVVEAAQKAGCGADMLYGPL
                     AGVNMDAPTYADAVGDWPFDLYSCIVDVEAYRTPTDGDQVPDCRAYWTRLAAVERCLD
                     CRSPTRSPRDFERYCDTCFLWRPRDSLPPPASGDYAWLFGRHQSVFLDEAEGTRDRNR
                     WRWLARLARCYQTAVVFWWHKAPLRRQPCGHIIEEALQNTKEGTVHFYRMPTFRTLTL
                     YDVWWPASTLMAAPNIGPATTFVLDDMVYRQSRWPTLPLPTAVASLDALD"
     gene            825768..826604
                     /gene="pino_cds_497"
     CDS             825768..826604
                     /gene="pino_cds_497"
                     /codon_start=1
                     /product="Hypothetical protein"
                     /translation="MEQSRTMTVEPPVVPVHEIVMPPLSTPAGGRALLRHAFPQHHRT
                     SVFHDRTADVRRAPKTTKKKKGKDGEKRLDKPAKERPVRMPTDAVVDLLMDHLIDAGA
[truncated: 1,200,014 more chars]
